# Thermal exposure of adult Chinook salmon and steelhead: Diverse behavioral strategies in a large and warming river system

**DOI:** 10.1371/journal.pone.0204274

**Published:** 2018-09-21

**Authors:** Matthew L. Keefer, Tami S. Clabough, Michael A. Jepson, Eric L. Johnson, Christopher A. Peery, Christopher C. Caudill

**Affiliations:** Department of Fish and Wildlife Sciences, College of Natural Resources, University of Idaho, Moscow, Idaho, United States of America; Texas A&M University, UNITED STATES

## Abstract

Rising river temperatures in western North America have increased the energetic costs of migration and the risk of premature mortality in many Pacific salmon (*Oncorhynchus* spp.) populations. Predicting and managing risks for these populations requires data on acute and cumulative thermal exposure, the spatio-temporal distribution of adverse conditions, and the potentially mitigating effects of cool-water refuges. In this study, we paired radiotelemetry with archival temperature loggers to construct continuous, spatially-explicit thermal histories for 212 adult Chinook salmon (*O*. *tshawytscha*) and 200 adult steelhead (*O*. *mykiss*). The fish amassed ~500,000 temperature records (30-min intervals) while migrating through 470 kilometers of the Columbia and Snake rivers *en route* to spawning sites in Idaho, Oregon, and Washington. Spring- and most summer-run Chinook salmon migrated before river temperatures reached annual highs; their body temperatures closely matched ambient temperatures and most had thermal maxima in the lower Snake River. In contrast, many individual fall-run Chinook salmon and most steelhead had maxima near thermal tolerance limits (20–22 °C) in the lower Columbia River. High temperatures elicited extensive use of thermal refuges near tributary confluences, where body temperatures were ~2–10 °C cooler than the adjacent migration corridor. Many steelhead used refuges for weeks or more whereas salmon use was typically hours to days, reflecting differences in spawn timing. Almost no refuge use was detected in a ~260-km reach where a thermal migration barrier may more frequently develop in future warmer years. Within population, cumulative thermal exposure was strongly positively correlated (0.88 ≤ *r* ≤ 0.98) with migration duration and inconsistently associated (-0.28 ≤ *r* ≤ 0.09) with migration date. All four populations have likely experienced historically high mean and maximum temperatures in recent years. Expected responses include population-specific shifts in migration phenology, increased reliance on patchily-distributed thermal refuges, and natural selection favoring temperature-tolerant phenotypes.

## Introduction

Long-distance migrations are energetically demanding and risky, but they allow animals to exploit spatially-dispersed resources and satisfy life history needs that vary through time [1–3]. Unfortunately, migratory populations can be especially prone to decline and extirpation due to habitat loss and fragmentation affecting one or more life stages, to construction of obstacles (e.g., roads, dams) along historic migration routes, and to the ecological and phenological changes associated with climate warming [4,5]. These diverse risks have been well documented for the anadromous salmonids (*Oncorhynchus*, *Salmo*, and *Salvelinus* spp), a largely philopatric and entirely ectothermic group that includes some of the most iconic long-distance migrants. Anadromous salmonids express remarkable levels of life history diversity and local adaptation [6–8], and these traits contribute to species resilience at the metapopulation scale [9,10]. The finely-calibrated life history strategies of individual populations, however, can be accompanied by increased vulnerability to rapid environmental changes that affect critical habitats or life cycle events.

The phenology of migration and reproduction are among the traits most often identified as sensitive to climate change [11–16]. It is well established that adult salmonid migration timing and spawn timing are genetically heritable over generations [17,18] and that these traits reflect historical adaptation to river discharge and temperature patterns [19,20]. There is also accumulating evidence that recent phenological shifts in many salmonid populations have occurred in response to climate-driven warming and have been accompanied by genetic changes [21–23]. What is much less clear is whether population-level adaptation can keep pace with climate change, and this is currently a central question in salmonid conservation and management. There is also considerable uncertainty about how behavioral and phenotypic plasticity, as opposed to genetic adaptation, contributes to the observed phenological changes in some salmonid populations. Plasticity is critically important for how animals respond to short-term (e.g., within-generation) environmental fluctuations, whereas genetic adaptation is likely necessary when environmental change is unidirectional, as appears to be the case with recent climate warming [20,24–28].

In the Pacific Northwest (PNW), climate change is a potentially strong selective force that affects not only adult salmonid phenology, but also the fitness and long-term viability of some populations [24,28–30]. Climate predictions for the PNW are for reduced snowpack, a shift from snow- to rain-dominated runoff, more severe summer low-flow conditions, and river temperatures that more frequently rise above stressful levels for many fish populations [31–36]. Hydrological changes will certainly not affect all species or populations equally given the large within-region gradients in latitude, elevation, precipitation, and thermal regimes among habitats currently used by anadromous salmonids. Populations that evolved in highly stochastic environments, for example, are generally less philopatric [37] and hence may readily locate suitable habitats when historical sites degrade. Some populations will potentially become more abundant as climate warming occurs, especially in areas where productivity is expected to increase with warming [38,39] or where current cold-limited conditions become more favorable for life history requirements like reproduction or juvenile rearing [27,40–42]. There is growing agreement among scientists, however, that many anadromous populations will be challenged to rapidly adapt to climate stressors that may manifest across multiple life stages and environments [43–46].

Understanding the narrower question of how warming rivers affect the adult life stage of anadromous fishes requires better quantitative information on the thermal exposure of individuals and populations and how they respond to adverse conditions. The unique biotelemetry dataset summarized in this paper includes continuous, spatially-referenced body temperature histories for adult spring-, summer-, and fall-run Chinook salmon (*O*. *tshawytscha*) and summer steelhead (*O*. *mykiss*) migrating through the large and warming Columbia and Snake rivers (Oregon-Washington, USA). The studied populations all have long-distance freshwater migrations through a highly-regulated river migration corridor that has warmed ~2.5 °C over the last several decades [21,25]. The “runs” represent groups of geographically and genetically distinguishable populations defined by adult migration timing. Although the four runs have extensive spatial overlap, particularly in their large-river migration corridor, they have substantially different migration and reproductive phenology and encounter distinctive thermal regimes during adult migration and pre-spawn holding periods. Divergence in thermal exposure suggests that the runs may also differ in their climate-warming vulnerabilities, offering a compelling opportunity for among-run comparisons.

Our broad objectives here were to characterize the thermal experience of a diverse group of adult salmonids with long-distance migrations, and to describe the behavioral responses of these populations to potentially stressful water temperatures in their migration corridor. Thermal regimes encountered by the four runs are tightly linked to their migration timing and the degree to which their migrations are ‘premature’ (i.e., freshwater entry well before reproduction [47]). Among the Chinook salmon, spring-run migrants are the most premature, entering freshwater several months before spawning [48,49]. This strategy may allow spring-run salmon to avoid snowmelt-driven peak discharge and the warmer and more energetically costly river temperatures of summer, but requires long pre-spawn holding in or near natal tributaries [47,50]. Summer- and fall-run Chinook salmon encounter much warmer migration temperatures than spring-run salmon, but typically move rapidly upstream and have relatively shorter pre-spawn holding, a strategy that may mitigate against extended exposure to stressful temperatures [51,52]. Lastly, the summer steelhead strategy features hyper-premature migration, with freshwater entry 6–11 months prior to springtime spawning [53]. Summer steelhead migration behaviors are remarkably diverse, with some fish moving rapidly upstream either before or after peak summer temperatures [54,55], and others migrating in mid-summer but with extended bouts of energy-conserving inactivity while holding in thermal refuges along migration routes [56–59].

We used the biotelemetry dataset to test several hypotheses about adult phenology and thermal exposure during migration. First, we expected that Chinook salmon migration timing would be a good predictor of mean and total thermal exposure across runs given predictable seasonal patterns of river warming and cooling. Second, we hypothesized that the warmest temperatures encountered by the four salmon and steelhead runs would occur in different river reaches due to seasonal river temperature patterns, but also to population-specific migration behaviors. More specifically, we anticipated that Chinook salmon confronted by warm water temperatures would migrate faster than those in cooler water and that fish from all runs would use cool water refuges when river temperatures exceeded stressful thresholds. Faster migration during warm periods would reflect increased metabolic efficiency at warm (but not hot) temperatures [60,61], but also the need to minimize the physiological and pathological risks of warm-water exposure [62,63] and the imperative to arrive at spawning sites at the most advantageous time [64,65]. Lastly, given the substantially different reproductive timetables of the two species, we expected that thermoregulatory behaviors would be prevalent and extensive for summer steelhead but limited to periods of acutely stressful temperatures for Chinook salmon.

## Methods

### Ethics statement

The methods used to collect, tag, and monitor adult salmon and steelhead migration were approved by the University of Idaho Animal Care and Use Committee, have been detailed previously [66,67], and are summarized in the section “Fish Collection, Tagging, and Monitoring” below. Fish collection was permitted annually by NOAA-Fisheries (e.g., Research Action 994) and the state of Washington (e.g., Scientific Collection Permit 02–150).

### Study system

**Fig 1:**
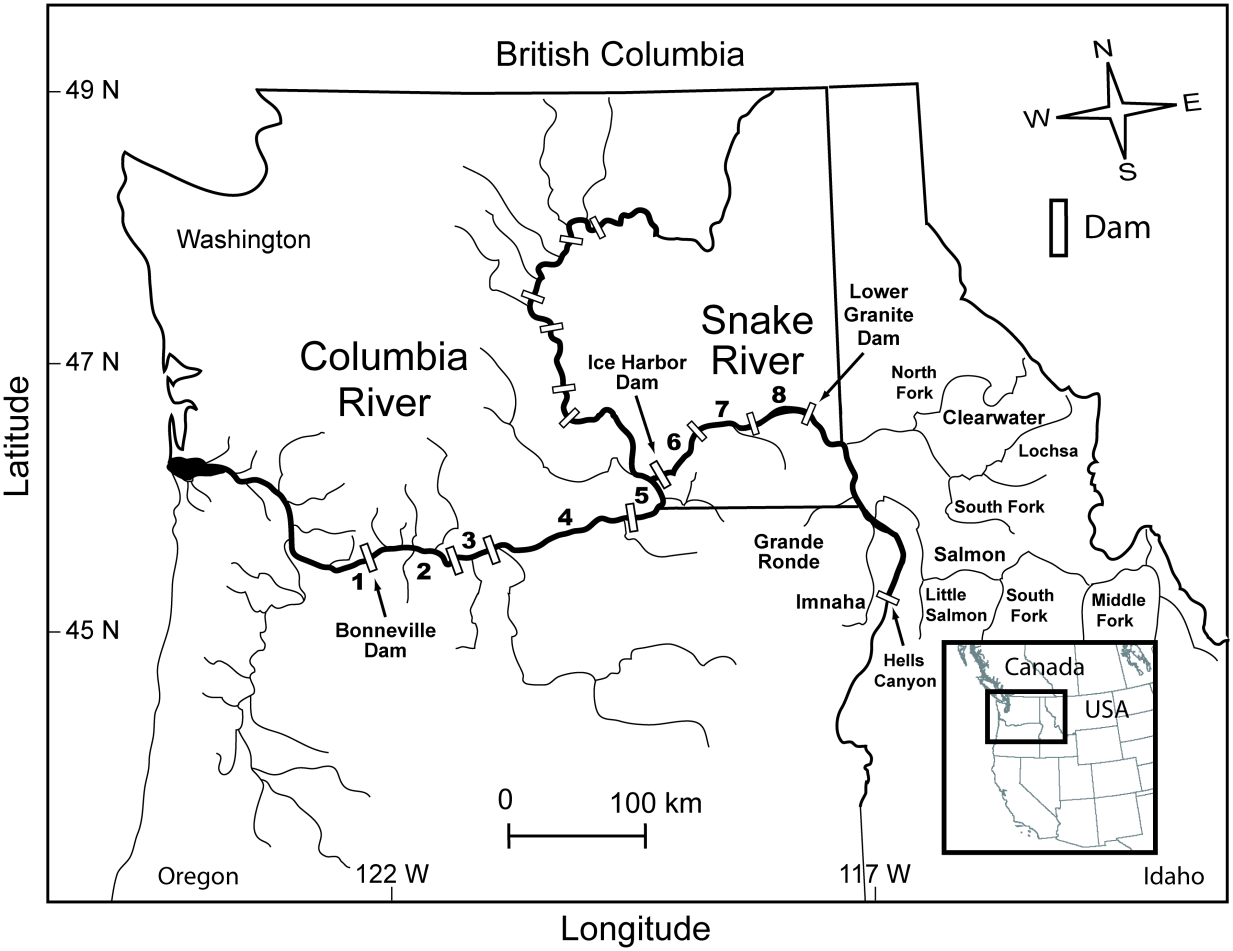
Map of the Columbia River—Snake River study area. Black bold numbers indicate the eight reaches where RDST-tagged Chinook salmon and steelhead were monitored, including: (1) release to Bonneville Dam, (2) Bonneville Dam to The Dalles Dam, (3) The Dalles Dam to John Day Dam, (4) John Day Dam to McNary Dam, (5) McNary Dam to Ice Harbor Dam, (6) Ice Harbor Dam to Lower Monumental Dam, (7) Lower Monumental Dam to Little Goose Dam, and (8) Little Goose dam to Lower Granite Dam. Inset map shows the study area in the northwestern United States.

The Columbia River drains >660,000 km^2^ of seven U.S. states, two Canadian provinces and has the highest annual discharge (>7,000 m^3^/s) of any North American river entering the Pacific Ocean [68,69]. The Snake River is the largest Columbia River tributary by drainage area and contains some of the highest quality salmon and steelhead spawning and rearing habitat in the region. Anadromous adults returning to natal sites in the Snake River basin must pass four large dams on the lower Columbia River and two to four more large dams on the lower Snake River (Fig 1). The Columbia River dams are Bonneville (located ~235 river kilometers [rkm] from the Pacific Ocean), The Dalles (rkm ~308), John Day (rkm ~347), and McNary (rkm ~470). The lower Snake River dams are Ice Harbor (rkm ~538), Lower Monumental (rkm ~589), Little Goose (rkm ~635), and Lower Granite (rkm ~695). All eight dams are run-of-river hydroelectric projects (i.e., limited long-term water storage) and water releases through turbines and spillways produce vertically well-mixed downstream reservoirs [69]. Our study area was ~470 rkm long, stretching from the Bonneville Dam tailrace to Lower Granite Dam. Upstream-migrating fish pass through three primary environments in this reach: (1) low-velocity reservoirs up to > 70 m deep; (2) dam tailraces with relatively high velocity and turbulence [70]; and (3) pool-and-weir adult fishways that rise ~20–32 m at each dam.

### Study populations

**Fig 2:**
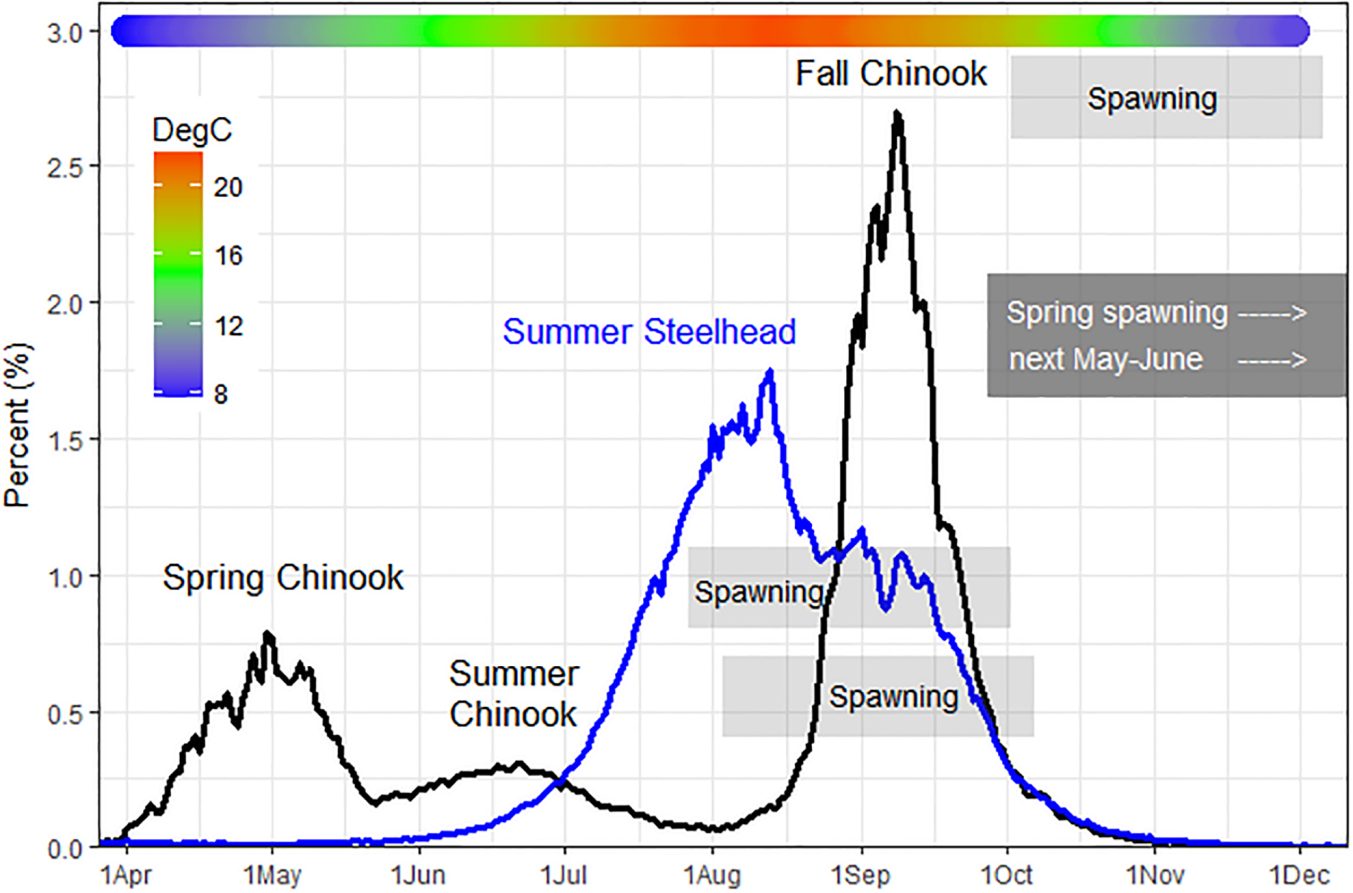
Chinook salmon and steelhead run timing and river temperatures. Mean daily percentage of Chinook salmon (spring, summer, and fall runs combined to show relative abundance) and summer steelhead counted of the total number of fish counted in each species in each year at Bonneville Dam on the Columbia River, 1996–2015. Gray boxes aligned with seasonal run names show approximate spawn timing in the Snake River basin; color bar at top shows mean daily water temperature at the Bonneville Dam water quality monitoring (WQM) site, 1996–2015.

The Snake River spring-, summer-, and fall-run Chinook salmon and summer steelhead populations we studied are currently listed as threatened under the U.S. Endangered Species Act [71]. Returning adults from the four runs migrate upstream past Bonneville Dam from March-May (spring Chinook salmon), June-July (summer Chinook salmon), August-October (fall Chinook salmon), and May-October (summer steelhead) (Fig 2). Most of the spring and summer Chinook salmon spawn in tributaries from August to September, 1–4 months after they initiate migration [51,72]. Most fall Chinook salmon spawn from mid-October to early December, weeks to ~2 months after migration initiation [73,74]. Snake River summer steelhead spawn 6–11 months after migration initiation. In freshwater, prespawn adult steelhead overwinter in large river habitats, including Columbia and Snake River reservoirs [75], and then spawn in tributaries from March to June of the following year [53].

### Fish collection, tagging, and monitoring

We collected adult Chinook salmon and steelhead at the Bonneville Dam adult fish facility (AFF) in 2000 and 2002 in approximate proportion to average migration timing distributions for each seasonal run. In the two years, we tagged 241 spring Chinook salmon, 155 summer Chinook salmon, 116 fall Chinook salmon, and 410 summer steelhead (Table 1). The tagged samples were 0.04–0.07% (spring Chinook salmon), 0.05–0.28% (summer Chinook salmon), <0.01–0.04% (fall Chinook salmon), and 0.05–0.07% (summer steelhead) of the adults counted at Bonneville Dam (~31,000–479,000 fish per annual run; count data archived by the University of Washington at: www.cbr.washington.edu/dart/). Salmon and steelhead with relatively high recapture probability were preferentially tagged so that we could more efficiently recover archival transmitters (see below). Samples were therefore predominated by hatchery- and known-origin fish whose migrations passed trapping facilities, especially the Lower Granite adult trap in the lower Snake River (Fig 1). Adult fish origin was assessed at the AFF by the presence of hatchery fin clips and/or presence of a passive integrated transponder (PIT) tag that was implanted during the juvenile life stage. The prerogative to recover archival data outweighed our effort to randomly sample, and we disproportionately tagged fish that were PIT-tagged as juveniles that originated from the Snake River basin. All data summaries in this paper are restricted to the salmon and steelhead that were definitively linked to the Snake River based on juvenile PIT tag location or adult return location. Importantly, passage behaviors are broadly similar for hatchery- and natural-origin adult salmonids in this system [76].

**Table 1:** Summary of Chinook salmon and steelhead tagging, RDST recovery, and survival.

Sample	Spring Chinook		Summer Chinook		Fall Chinook		Steelhead	
	2000	2002	2000	2002	2000	2002	2000	2002
Tagged & released (*n*)	126	115	87	68	80	36	181	229
Recovered RDST (*n*)	63	81	13	27	8	20	46	154
(% of tagged & released)	50%	70%	15%	40%	10%	56%	30%	67%
Survived to LGr (*n*)	63	70	13	24	8	13	46	130
(% of recovered RDST)	100%	86%	100%	89%	100%	65%	100%	84%
RDST data to LGr (*n*)	59	67	12	22	7	14	22	53
(% of recovered RDST)	94%	83%	92%	81%	88%	70%	48%	34%

Numbers of Chinook salmon and steelhead that: (1) were tagged and released; (2) had their RDST recovered; (3) survived to Lower Granite Dam (LGr); and (4) had continuous temperature data from Bonneville Dam to Lower Granite dam in 2000 and 2002. Percentages are of the number of fish tagged and released (Recovered RDST) or of the number of RDSTs recovered (Survived to LGr, RDST data to LGr).

Details of adult salmon and steelhead trapping, anesthesia, handling, radio-tagging, transport, and release protocols have been reported previously [66,77]. Briefly, migrants selected for the archival tag study were anesthetized and then intragastrically tagged with a uniquely-coded 3-V radio data storage transmitter (RDST; Model LTD_100, 9-cm × 2-cm, 34 g in air, Lotek Wireless, Inc., Newmarket, Ontario) and a full-duplex PIT tag if they did not already have one. In addition to transmitting a radio signal, the transmitters logged fish body temperature every one minute and pressure (i.e., fish depth) every 5 seconds. The settings allowed archival storage of approximately 40 d of data and produced data files of ~600,000 records per fish. The manufacturer-reported accuracy for the pressure sensor was ±4.8 kPa (±0.5 m). Temperature sensor resolution was reported to be 0.02 °C with accuracy of ±0.15 °C at ambient temperatures of 0–20 °C and ±0.10 °C at temperatures of 20–35 °C. After tagging, fish recovered in a 2,275-L tank of oxygenated river water and were then transported to Columbia River release sites on both sides of the river ~10 rkm below Bonneville Dam.

RDST-tagged fish were monitored in Columbia and Snake River dam tailraces, inside dam fishways, in reservoirs, and in tributaries to assess a diverse mix of study objectives [58,66,67,77,78]. Multiple radio receivers and antennas were deployed at each of the eight dams in the Bonneville-Lower Granite study reach. Additional radio antennas were located in most major Columbia and Snake River tributaries, and at Priest Rapids and Wanapum dams on the upper Columbia River (Fig 1). Detections at PIT-tag antennas supplemented salmon and steelhead movement histories, including at two dams with limited (Rock Island Dam) or no (Wells Dam) radiotelemetry monitoring and in some tributaries and hatchery facilities.

### RDST data collection and processing

The radiotelemetry data were regularly downloaded from receivers and assembled into annual databases by experienced personnel using established data filtering and coding methods. Fish temperature and pressure data from recovered RDSTs were downloaded and processed using manufacturer-recommended methods (TagTalk for Windows User Manual, Revision 1.0, Lotek Wireless, Inc.). Quality control screens included tests for temperature and depth values outside expected ranges and cross-checks with the radiotelemetry data for appropriate date-time stamps. To facilitate data analysis and presentation, we reduced the RDST temperature and depth data from 1-min (temperature) and 5-sec (depth) to 30-minute intervals for each fish by excerpting records near each hour and half-hour (i.e., ~12:30, ~13:00, ~13:30, etc.). The temperature, depth, and telemetry data were then merged to assemble spatially-referenced migration histories for each Chinook salmon and steelhead. In an additional post-processing step, each depth and temperature record was assigned to one of eight main stem reaches (Fig 1). Reach start points were either the release sites (Reach 1) or top-of-fishway antennas at dams located at the downstream end of a reach (Reaches 2–8). Reach end points were top-of-fishway antennas (Reaches 1–7) or the Lower Granite adult trap (Reach 8). On average, reaches were ~59 rkm long and ranged from ~10 rkm (release-Bonneville Dam) to ~123 rkm (John Day Dam- McNary Dam). Many Snake River fish used cool and cold water thermal refuge areas along the migration corridor where tributaries enter the Columbia and Snake rivers; RDST records collected while fish were in refuge areas were assigned to the adjacent main stem reach.

### Data summaries and analyses

Temperature data from recovered RDSTs were reviewed and evaluated at two spatial scales: (1) within each of the eight Columbia and Snake River reaches; and (2) over the full ~470 rkm study area. The reach-specific summaries included data from all Snake River fish detected in each reach, regardless of whether individuals ultimately passed upstream. In contrast, the full-migration summaries were restricted to the subset that survived to Lower Granite Dam and had RDSTs that collected data through the entire study area. To help characterize the diversity of individual Chinook salmon and steelhead thermal histories, we reviewed thermographs that were color-coded for river reach for every recovered RDST (S1 and S2 Appendices). We also overlaid the temperature data from each fish on reach-specific date×temperature plots to help visualize among-run differences in thermal exposure and fish behavior.

To characterize reach-specific thermal experiences, we calculated individual salmon and steelhead mean, minimum, and maximum temperatures in each reach. To test whether fish selected water temperatures that were warmer or cooler than ambient Columbia or Snake River temperatures (i.e., behavioral thermoregulation), we calculated the difference between each RDST record and water temperatures collected at nearby dams. Comparison data were the mean daily values collected at dam water quality monitoring (WQM) sites by USACE (archived at: www.cbr.washington.edu/dart/). WQM temperature data were available at all dams during the spring and summer Chinook salmon runs and we used data from the dam at the upstream end of each reach (i.e., at Bonneville Dam for reach 1) for comparisons to fish body temperatures. Several WQM sites ended temperature data collection in September or October, and so reference sites had to change slightly for fall Chinook salmon and steelhead: WQM data were from Bonneville Dam (fish in reaches 1 and 2), McNary Dam (fish in reaches 3 and 4); Ice Harbor Dam (fish in reaches 5 and 6); and Lower Granite Dam (fish in reaches 7 and 8). We were confident that temperature data from these sites were reasonable proxies because water temperatures are highly spatially correlated at adjacent dams and reservoirs in this system [79–81].

For the fish with temperature data over the full study reach, we calculated mean, minimum, and maximum values and identified the individual reaches where each fish experienced their warmest and coolest temperatures. Cumulative temperature exposure was calculated using degree days (DD, the average daily temperature exposure above 0 °C) for each fish from release to Lower Granite Dam. We assessed cumulative exposure to potentially stressful temperatures by calculating DD values above 20°C, denoted DD_20_; lethal and sub-lethal effects in adult salmonids can rapidly increase above 20°C, particularly after prolonged exposure [82]. The effects of migration timing and migration duration on DD and DD_20_ accumulation were explored using correlation analyses. Importantly, the 40-d RDST storage limit was exceeded for many steelhead because they had extended periods of behavioral thermoregulation in summer and early fall. The 40-d limit therefore introduced some bias into the full-migration steelhead sample by excluding fish with the long migration times and high DD and DD_20_ accumulations.

## Results

### Columbia and Snake River temperatures

**Fig 3:**
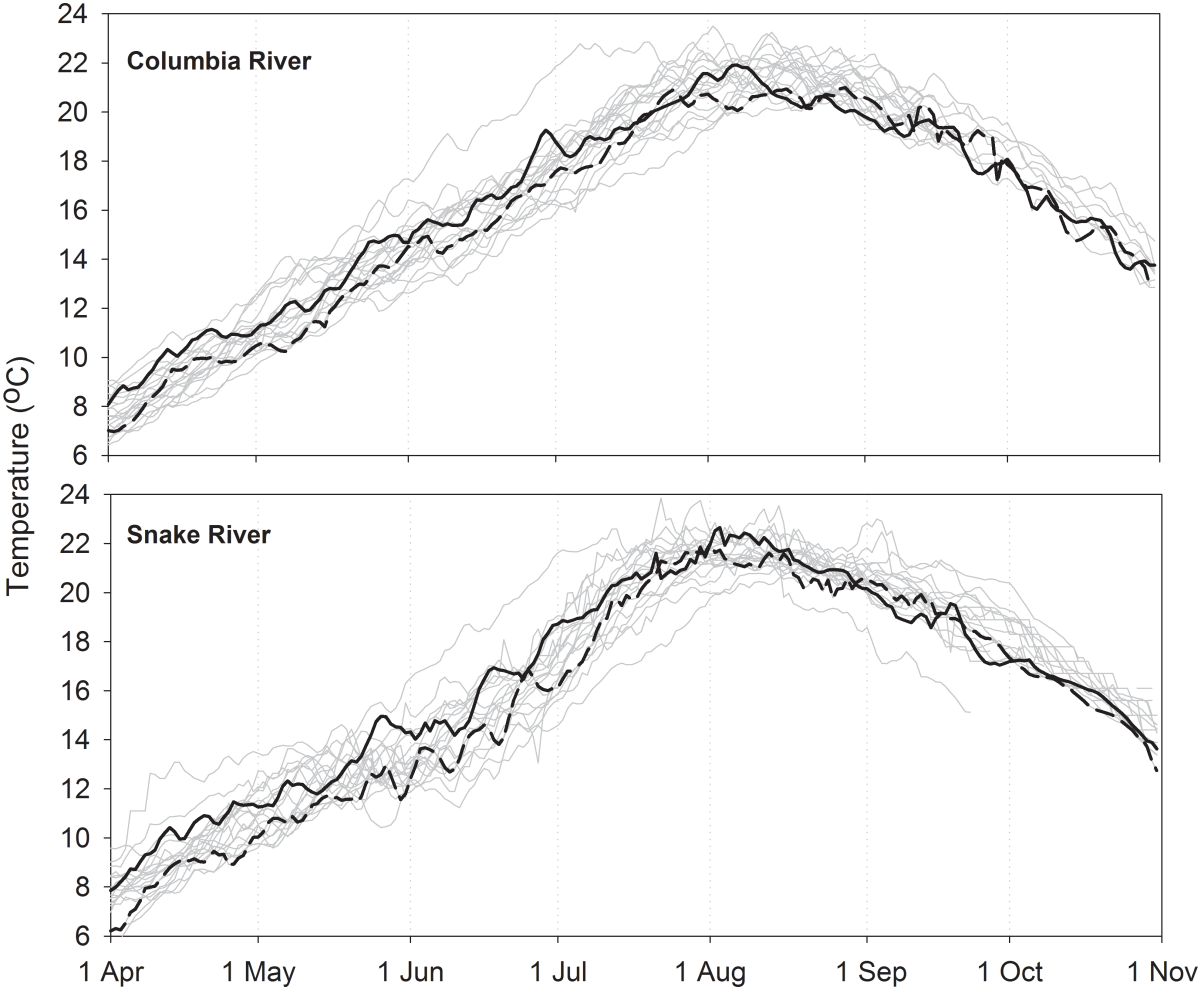
Mean daily Columbia and Snake River water temperatures. Data collected at the Bonneville Dam (top) and Ice Harbor Dam (bottom) water quality monitoring sites from 1996–2015. Solid and dashed black lines show 2000 and 2002 data, respectively. Data were not collected in parts of September and October of several years. Source: U.S. Army Corps of Engineers, archived by University of Washington: www.cbr.washington.edu/dart/.

Water temperatures in the Columbia and Snake rivers, as measured at Bonneville and Ice Harbor dams, steadily warmed from 7–8 °C in early April to annual peaks of 20–22 °C in August in both study years (Fig 3). Seasonal cooling started in August and temperatures declined to 12–14 °C in both rivers by late October. Mean water temperatures in the spring and summer months of 2000 were slightly warmer than or near the 20-yr (1996–2015) average in both the Columbia and Snake rivers. In contrast, mean monthly temperatures in 2002 were below the 20-yr average in both rivers. September and October temperatures also appeared to be below average in both years, though the temperature dataset was incomplete for these months, especially after 2005. Within year, mean daily Columbia and Snake River water temperatures were highly correlated (*r* ~0.99). However, in both years the Snake River was slightly cooler (~0.5–1.5 °C) than the Columbia River in much of June and slightly warmer (~0.5–1.5 °C) than the Columbia River in August.

### Fish sample summary

In the two years, we recovered RDSTs from 412 Chinook salmon and steelhead of Snake River origin (Table 1). The 412 included 144 spring Chinook salmon (60% of the spring Chinook salmon tagged at Bonneville Dam), 40 summer Chinook salmon (26%), 28 fall Chinook salmon (24%), and 200 summer steelhead (49%). Large majorities of the recovered RDSTs (~75–90% of each run) were collected at the Lower Granite adult trap, and the remainders were collected at hatchery facilities or from fisheries. Fish collected at the Lower Granite trap were re-tagged and released with new radio transmitters and ultimately migrated to a broadly representative group of hatcheries and tributary spawning sites in the Clearwater, Grande Ronde, Salmon, and Imnaha rivers and in the main stem Snake River (S3 Appendix). The subset that was PIT-tagged as juveniles (53–64% of Chinook salmon and 77% of steelhead) came from 43 Snake River locations, including juvenile collection sites at Lower Granite Dam (S3 Appendix).

Based on fin clips, 61–65% of the recovered Snake River spring and summer Chinook salmon were of certain hatchery origin versus 32–33% of fall Chinook salmon and steelhead. Fork lengths for the recovered sample ranged from 63–104 cm, with means of 77.9 cm (spring Chinook salmon), 78.4 cm (summer Chinook salmon), 77.1 cm (fall Chinook salmon), and 78.7 cm (summer steelhead). Migration timing of the recovered sub-samples broadly paralleled run timing distributions at Bonneville Dam (see Fig 2) for spring Chinook salmon (*mean tag date* = 3 May, *range* = 3 April to 31 May), summer Chinook salmon (*mean* = 13 June, *range* = 3 June to 28 July, fall Chinook salmon (*mean* = 7 September, *range* = 11 August to 19 October), and summer steelhead (*mean* = 17 August, *range* = 4 June to 17 October).

Across runs and years, 89% of the recovered Snake River fish survived migration to Lower Granite Dam (Table 1), with most non-survivors harvested in Columbia or Snake River fisheries. Because RDSTs had a 40-d data storage limit, not all fish that survived to Lower Granite Dam had archival temperature histories through the entire ~470 rkm reach. On average, ~85% of the Chinook salmon (all run-years) had complete Bonneville-Lower Granite RDST data versus 34–48% of steelhead. Percentages were lower for steelhead because many had migration times >40 d. Total RDST data volume varied by run and study reach as a function of reach-specific sample sizes and fish residence times (Table 2). In total, we collected data equivalent to 2,846 d (spring Chinook salmon), 680 d (summer Chinook salmon), 478 d (fall Chinook salmon), and 6,375 d (summer steelhead).

**Table 2:** Numbers of Snake River Chinook salmon and steelhead and total fish-days of recovered RDST data by reach in 2000 and 2002 combined.

Reach	Spring Chinook		Summer Chinook		Fall Chinook		Steelhead	
	*n*	days	*n*	days	*n*	days	*n*	days
1: REL-BON	144	656	40	84	28	95	200	631
2: BON-TDD	144	467	40	96	28	70	198	3047
3: TDD-JDD	136	323	38	150	25	58	148	1277
4: JDD-MCN	135	450	37	127	25	69	113	424
5: MCN-IHD	130	279	36	57	22	42	100	388
6: IHD-LMD	129	214	36	51	22	56	85	183
7: LMD-LGO	128	221	36	53	21	34	81	187
8: LGO-LGR	126	236	34	63	21	56	75	238
All	144	2846	40	681	28	480	200	6375

Reach abbreviations: REL = release sites; BON = Bonneville Dam; TDD = The Dalles Dam; JDD = John Day Dam; MCN = McNary Dam; IHD = Ice Harbor Dam; LMD = Lower Monumental Dam; LGO = Little Goose Dam; LGR = Lower Granite Dam.

### Fish temperatures: Individual river reaches

**Fig 4:**
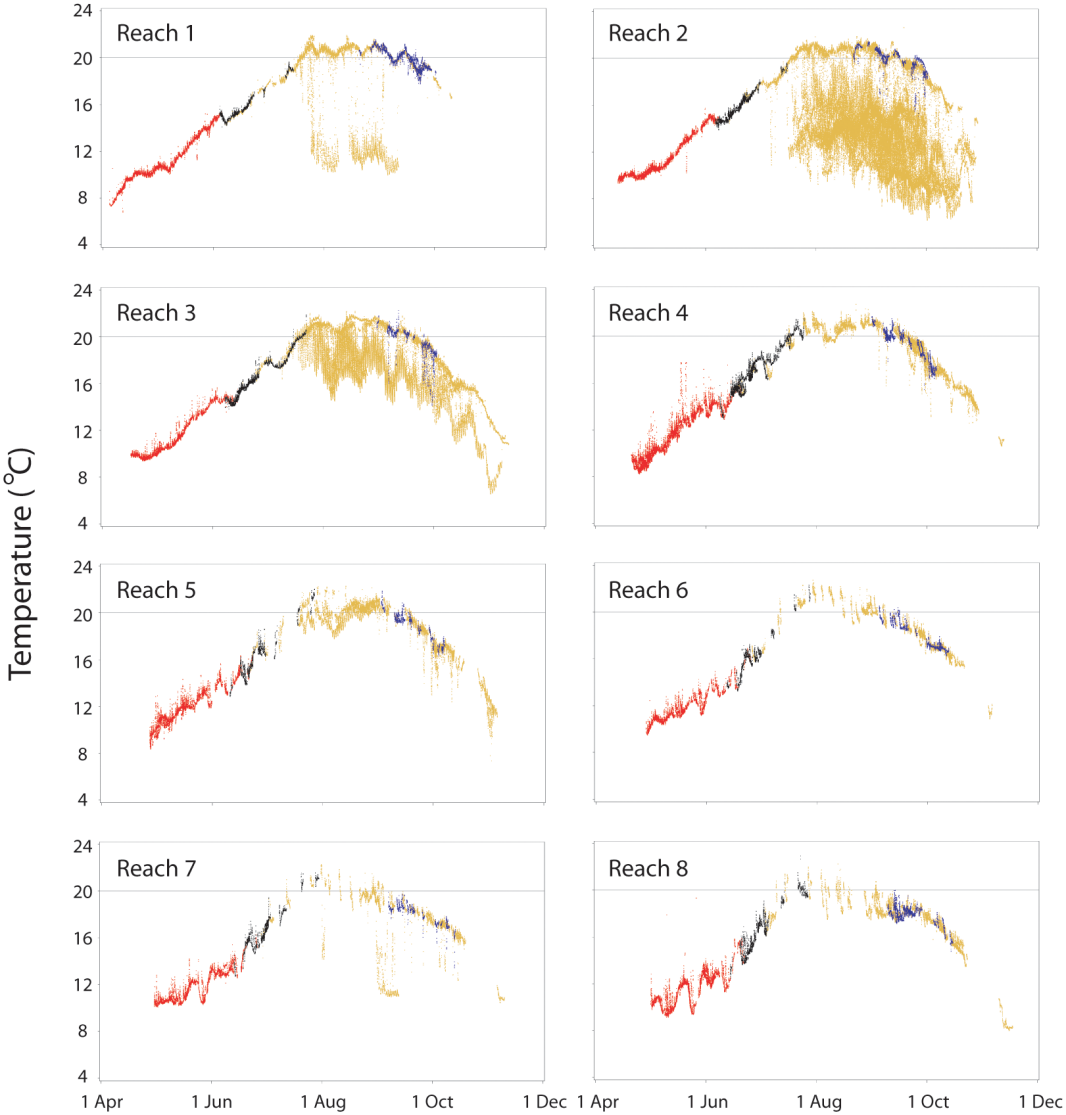
Body temperatures of Snake River Chinook salmon and steelhead. Data collected in 30-min intervals during fish migration through eight Columbia and Snake River reaches in 2002. Red dots are for spring Chinook salmon (67–89 fish, 105–502 total fish-days per reach); black dots are for summer Chinook salmon (22–33 fish, 26–110 total fish-days per reach); blue dots are for fall Chinook salmon (14–21 fish and 19–76 total fish-days per reach); and gold dots are for summer steelhead (53–167 fish, 123–2,543 total fish-days per reach). Horizontal line at 20 °C is a reference for potentially stressful conditions. Reach-specific results were qualitatively and quantitatively similar in 2000.

Snake River spring and summer Chinook salmon body temperatures indicated that most fish stayed within narrow within-day water temperature ranges within each study reach (Fig 4). Departures from apparent ambient river temperatures were mostly positive (i.e., the salmon was warm relative to the river), and these departures often occurred when fish were inside dam fishways or in dam forebays. Most of the fall Chinook salmon body temperature data also fell within narrow seasonal ranges, but relatively more fish had either warm or cool deviations (Fig 4). The cool deviations were mostly in reaches 2, 3, and 7 where some fall Chinook salmon briefly used cool water thermal refuges. Steelhead body temperatures were highly variable in comparison, especially after ambient river temperatures reached ~19 °C, a threshold associated with thermal refuge use. Long-duration refuge use (i.e., days to weeks) by steelhead was evident in reaches 1–3 and relatively short-duration use (i.e., hours to days) occurred in reaches 4, 5, and 7 (Fig 4). As with Chinook salmon, some steelhead experienced brief spikes of warm water exposure, often at or near dams.

**Fig 5:**
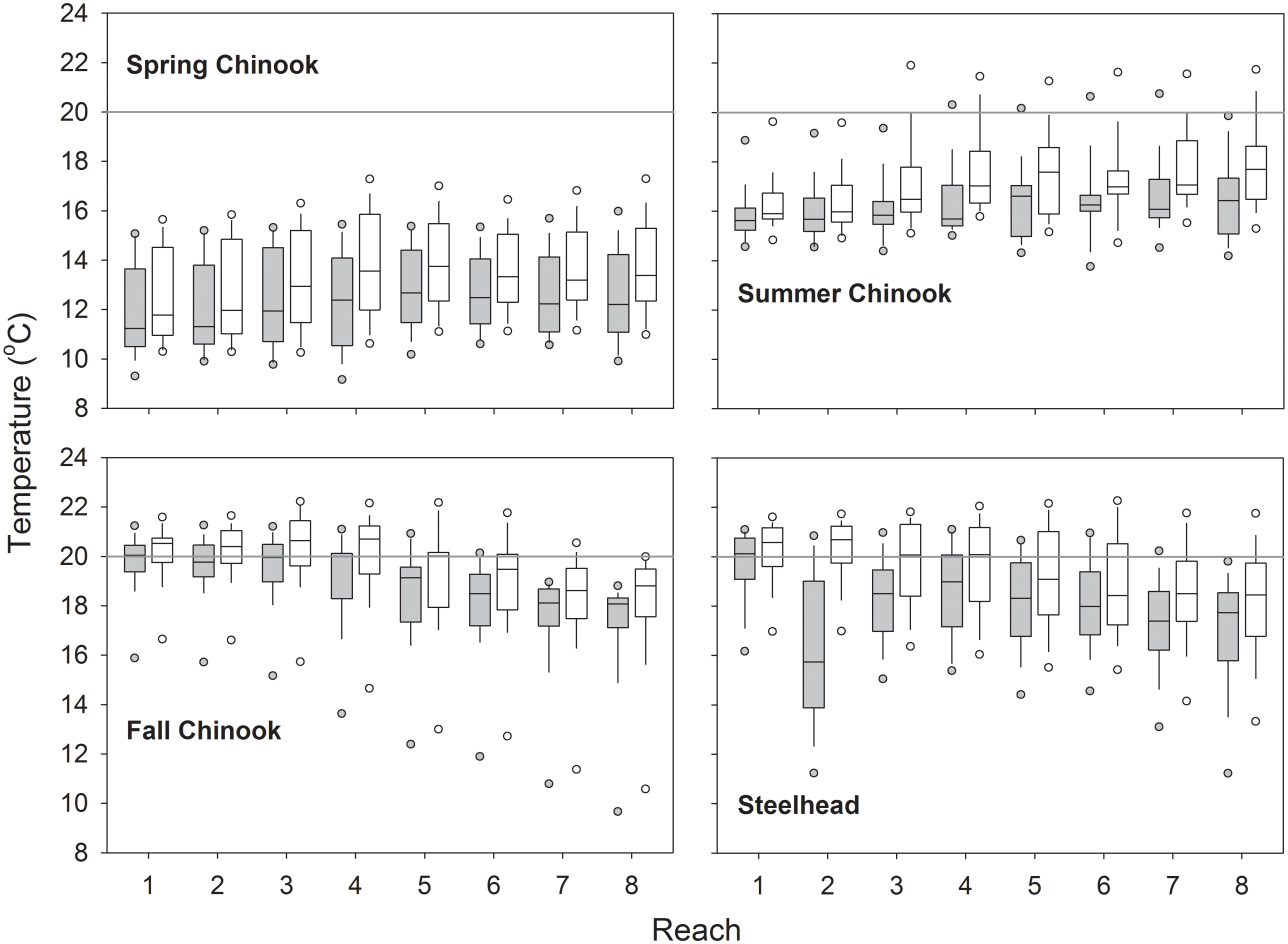
Reach-specific Chinook salmon and steelhead body temperatures. Box plots show 5^th^, 10^th^, 25^th^, 50^th^, 75^th^, 90^th^ and 95^th^ percentiles of mean (gray boxes) and maximum (white boxes) body temperatures in the eight study reaches, 2000 and 2002 combined. See Table 2 for sample sizes.

Individual body temperatures of spring Chinook salmon ranged from ~9–16 °C, on average, with relatively little among-reach difference in the overall distributions of means (Fig 5). Individual maxima were mostly 1–3 °C higher than individual means and almost no spring Chinook salmon had maxima > 18 °C. Among summer Chinook salmon, individual reach-specific means ranged from ~14–21 °C and individual maxima ranged from ~15–22 °C; maxima were slightly higher in lower Snake River reaches than in lower Columbia River reaches. Most fall Chinook salmon had means of ~16–21 °C and maxima of ~17–22 °C. The fall run also experienced seasonal cooling as fish moved upstream and a small number of late-migrating fish had very low mean and maximum values (Fig 5). Patterns for steelhead paralleled those for fall Chinook salmon, with cooler individual mean and maximum temperatures as fish progressed upstream, and a few late-migrating fish that encountered lower temperatures. Steelhead differed from the salmon runs most starkly in reaches 2 and 3, where thermoregulatory behavior was extensive.

**Fig 6:**
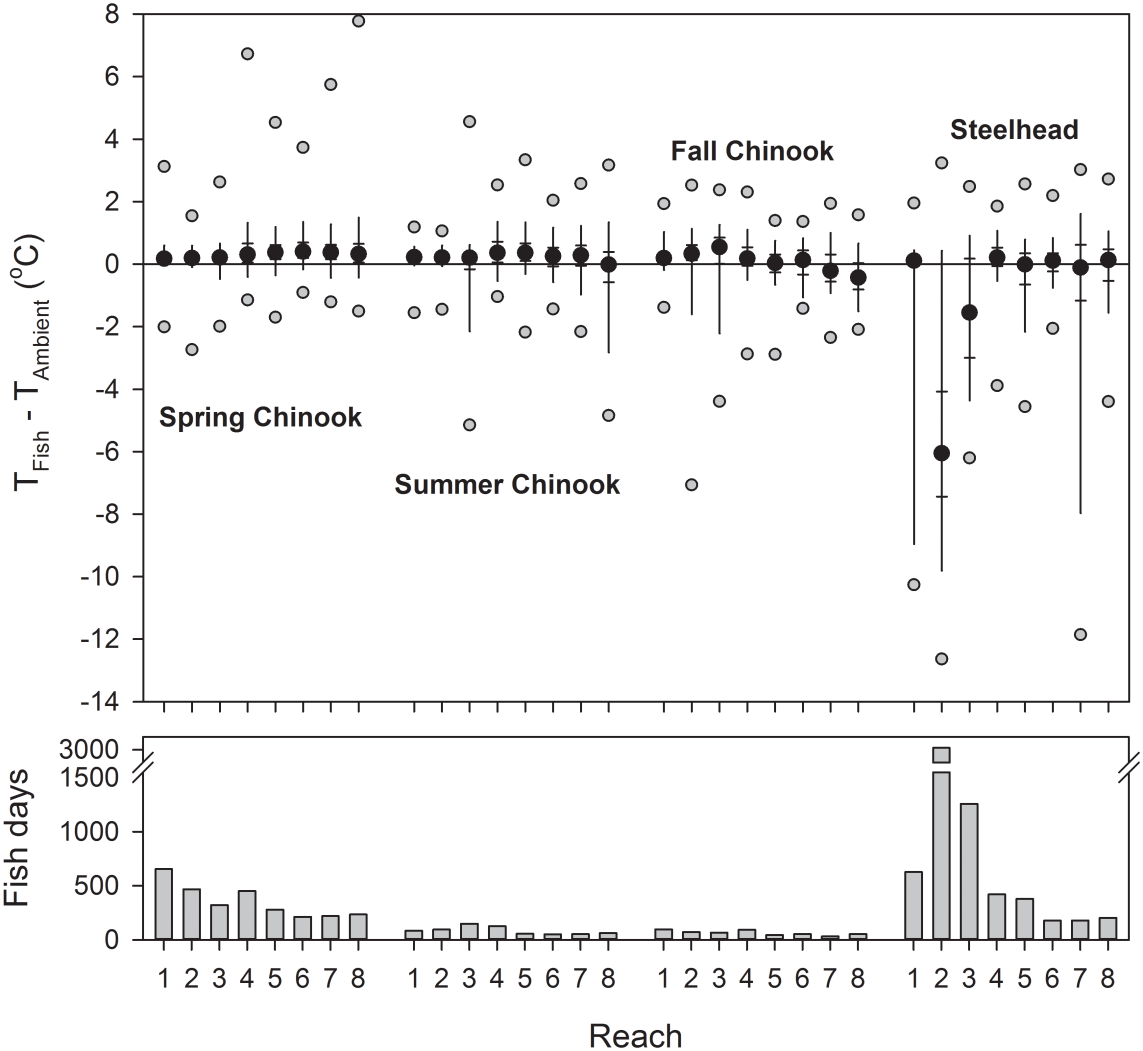
Fish temperatures versus river temperatures. Differences between body temperatures of Snake River Chinook salmon and steelhead (T_Fish_) and mean daily Columbia and Snake River water temperature at the nearest WQM site (T_Ambient_), by reach, 2000 and 2002 combined. Top plot shows 5^th^, 25^th^, 50^th^, 75^th^, 95^th^ percentiles (black dots and whiskers) plus minimum and maximum (grey circles) differences. Bottom plot shows the total numbers of days fish spent in each reach (Fish days).

Spring Chinook salmon body temperatures rarely differed from ambient river temperatures by more than ±1 °C in any study reach (Fig 6). On median, spring-run salmon body temperatures ranged from 0.17–0.40 °C above ambient, their 95^th^ percentiles ranged from 0.59–1.49 °C above ambient, and their 5^th^ percentiles ranged from 0.05–0.47 °C below ambient. Patterns were similar for summer and fall Chinook salmon, which had medians 0.03–0.54 °C above ambient in all reaches except 7 and 8, where medians for fall-run fish were -0.22 °C and -0.43 °C, respectively (Fig 6). Thermoregulatory behaviors by steelhead resulted in large body temperature departures from river temperatures in several reaches. Median differences were -6.06 °C in reach 2 and -1.55 °C in reach 3. About 10% of steelhead body temperatures were -7.25 °C to more than -10.0 °C below ambient in reaches 1, 2, and 7, where thermal refuges at tributary confluences were most accessible (Fig 6).

### Fish temperatures: Release to Lower Granite Dam (~470 rkm)

**Fig 7:**
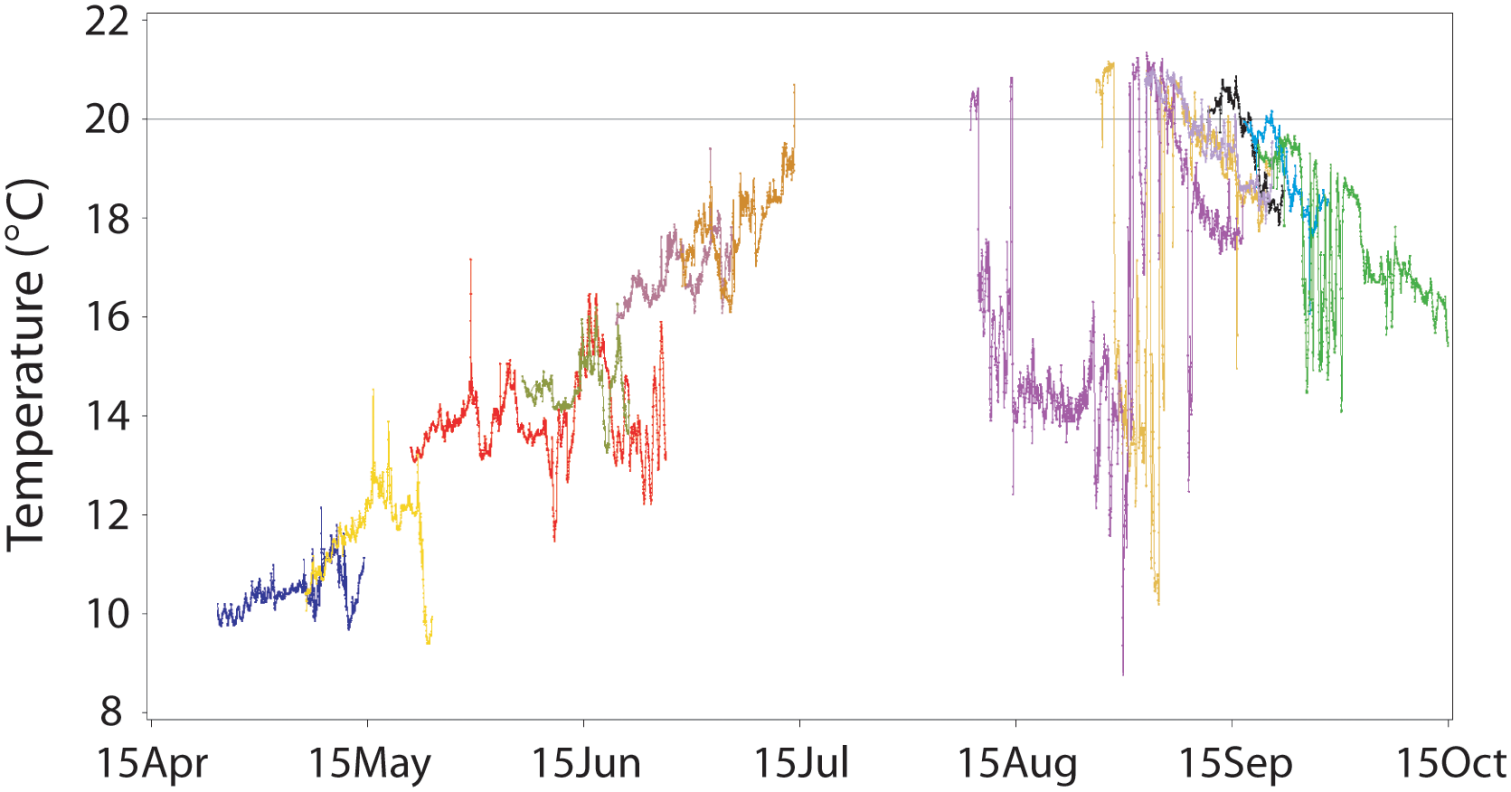
Full-migration temperature histories. Twelve Snake River Chinook salmon and steelhead body temperature histories (30-min data) from release below Bonneville Dam to Lower Granite Dam in 2002. SP = spring Chinook salmon, SU = summer Chinook salmon, FA = fall Chinook salmon, ST = summer steelhead. Individuals were selected using a random-number generator.

**Fig 8:**
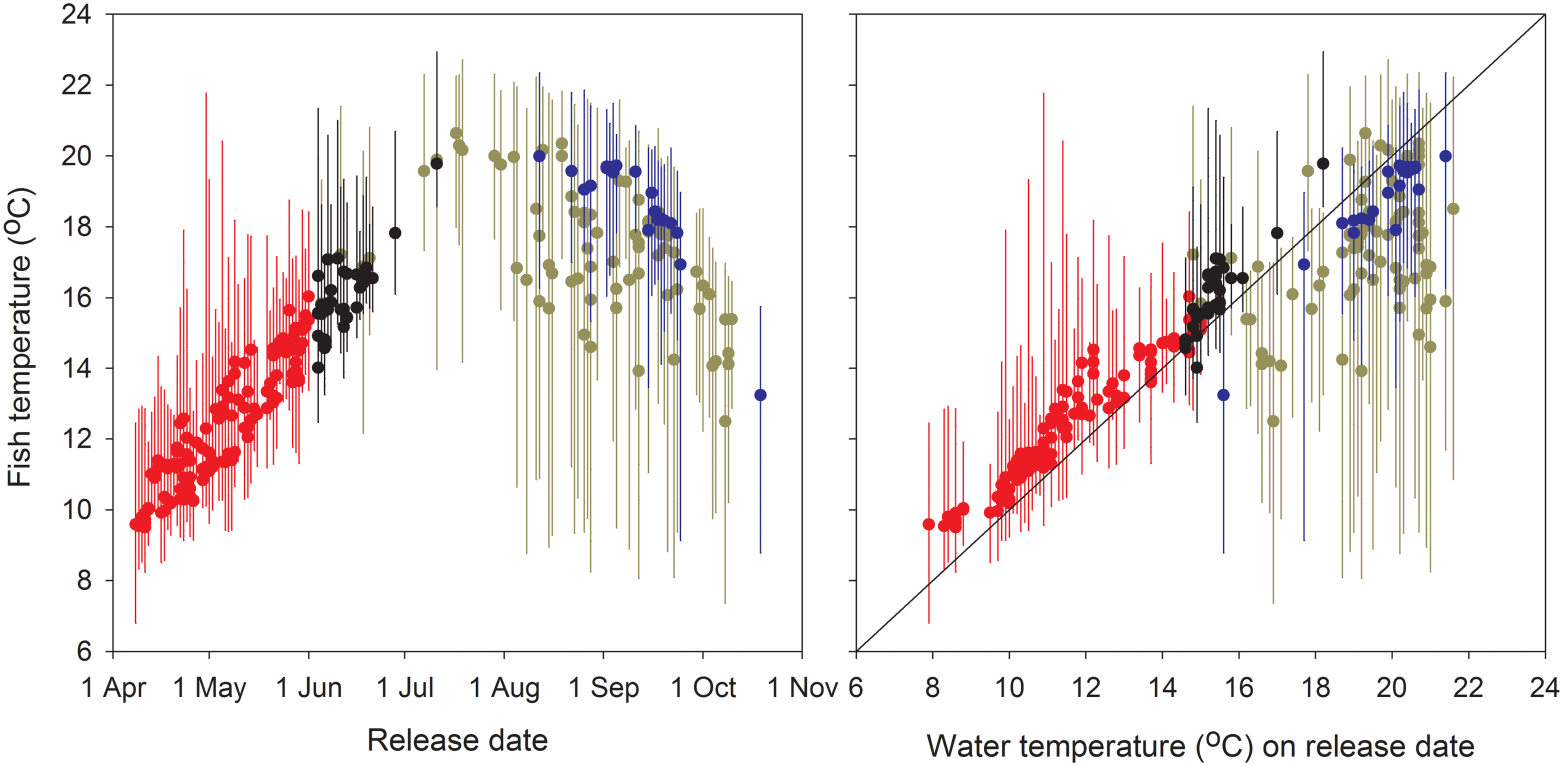
Range of fish temperature exposure over their full migration. Individual Snake River Chinook salmon and steelhead mean, minimum, and maximum body temperatures during the ~470 rkm migration from release to Lower Granite Dam in relation to release date (left panel) and Columbia River water temperature on their release date (right panel) in 2000 and 2002. Red symbols are for spring Chinook salmon (*n* = 126); black symbols are for summer Chinook salmon (*n* = 34); blue symbols are for fall Chinook salmon (*n* = 21); and gold symbols are for summer steelhead (*n* = 75).

**Fig 9:**
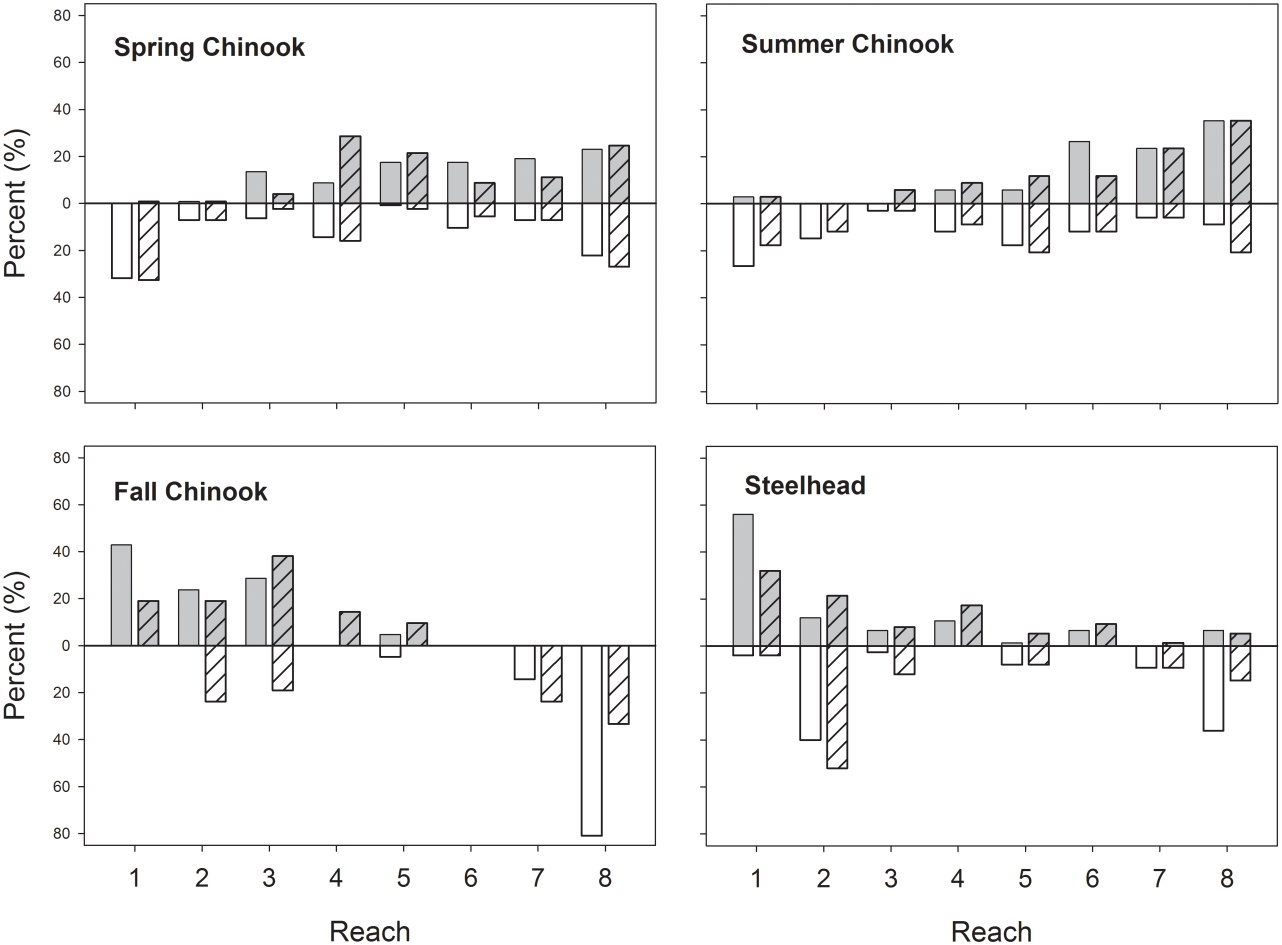
Spatial distribution of thermal extremes. Reaches where individual Snake River Chinook salmon and steelhead had their warmest mean body temperature (solid gray bars), body temperature maxima (hashed gray bars), coolest mean body temperatures (solid white bars), and body temperature minima (hashed white bars). Percentages are of all tagged fish with complete RDST histories within each run in 2000 and 2002.

The subset of RDST-tagged fish that had body temperature histories through the entire study reach included 126 spring Chinook salmon, 34 summer Chinook salmon, 21 fall Chinook salmon, and 75 steelhead (Table 1). Fish migration times through the combined reach ranged from 8.9 d to 40.0 d (the RDST storage limit), and mean times were 19.8 d (spring Chinook salmon), 15.4 d (summer Chinook salmon), 18.3 d (fall Chinook salmon), and 27.1 d (summer steelhead). Longer passage times for some salmon were associated with downstream fallback at dams followed by re-ascension of fishways. In addition, steelhead with the longest migration times were excluded due to the 40-d data storage limit, biasing the sample against early- and mid-summer migrants. The radio transmitters remained functional, however, and travel times for all steelhead that reached Lower Granite Dam averaged 59.9 d (*median* = 49.7 d, *n* = 130). Variability in reach migration times and seasonal changes in river environment resulted in cumulative thermal experiences that were highly variable among individuals both within and among runs (Figs 7–9).

Thermal histories of spring and summer Chinook salmon showed that many fish warmed slightly over the course of their migrations (e.g., SP2, SU2, SU3 in Fig 7; complete histories in S1 and S2 Appendices). Other spring- and summer-run fish had relatively flat thermal histories (e.g., SP3, SU1), particularly in June when river temperatures were cooler in the Snake River than in the Columbia River. Conversely, most fall Chinook salmon cooled as they moved upstream (e.g., FA1, FA2, FA3), reflecting seasonal cooling in both rivers. Steelhead thermal histories were much more diverse than those of Chinook salmon because steelhead migrated more slowly, were more likely to behaviorally thermoregulate, and had migrations that often spanned portions of summer and fall. Individuals from all four runs had brief periods of relatively warm exposure (e.g., SP2, SP3, SU3), often associated with passage through dam fishways and dam forebays and occasionally near warm-water tributary confluences.

Over the full study reach, mean body temperatures of individual Snake River Chinook salmon were 12.3 °C (spring run), 16.0 °C (summer run), and 18.6 °C (fall run), with values generally tracking seasonal changes in river temperatures (Fig 8). The mean steelhead temperature was 17.2 °C, with many summer and early fall migrants having means well below ambient as described above. Individual temperature ranges (i.e., maximum-minimum) for the runs averaged 3.8 °C (spring Chinook salmon), 3.6 °C (summer Chinook salmon), 4.9 °C (fall Chinook salmon), and 8.1 °C (summer steelhead). Columbia River water temperature on the date that Chinook salmon were tagged was generally a good indicator of the mean full-reach temperature of each fish (Fig 8). Mean salmon temperatures were ~0–3 °C warmer (spring and summer fish) or 1–2 °C cooler (fall fish) than temperature on the date each fish was tagged. Release date temperature was much less predictive of mean temperature for steelhead.

The specific reaches where individuals experienced their warmest and coolest temperatures differed markedly among runs, reflecting a mix of seasonal river temperature patterns and variable use of thermal refuges (Fig 9). The warmest individual mean and maximum temperatures for spring Chinook salmon were distributed among the six upstream reaches, especially reaches 4, 7 and 8. The warmest reaches for summer Chinook salmon were in the Snake River (reaches 6–8). In contrast, most fall Chinook salmon and summer steelhead had their warmest means and maxima in the lower Columbia River, particularly in reaches 1–3. Sites where fish experienced low mean and minimum temperatures were widely distributed for spring and summer Chinook salmon, and were concentrated in reaches 2, 3, 7, and 8 for fall Chinook salmon and reaches 2 and 8 for steelhead (Fig 9).

**Fig 10:**
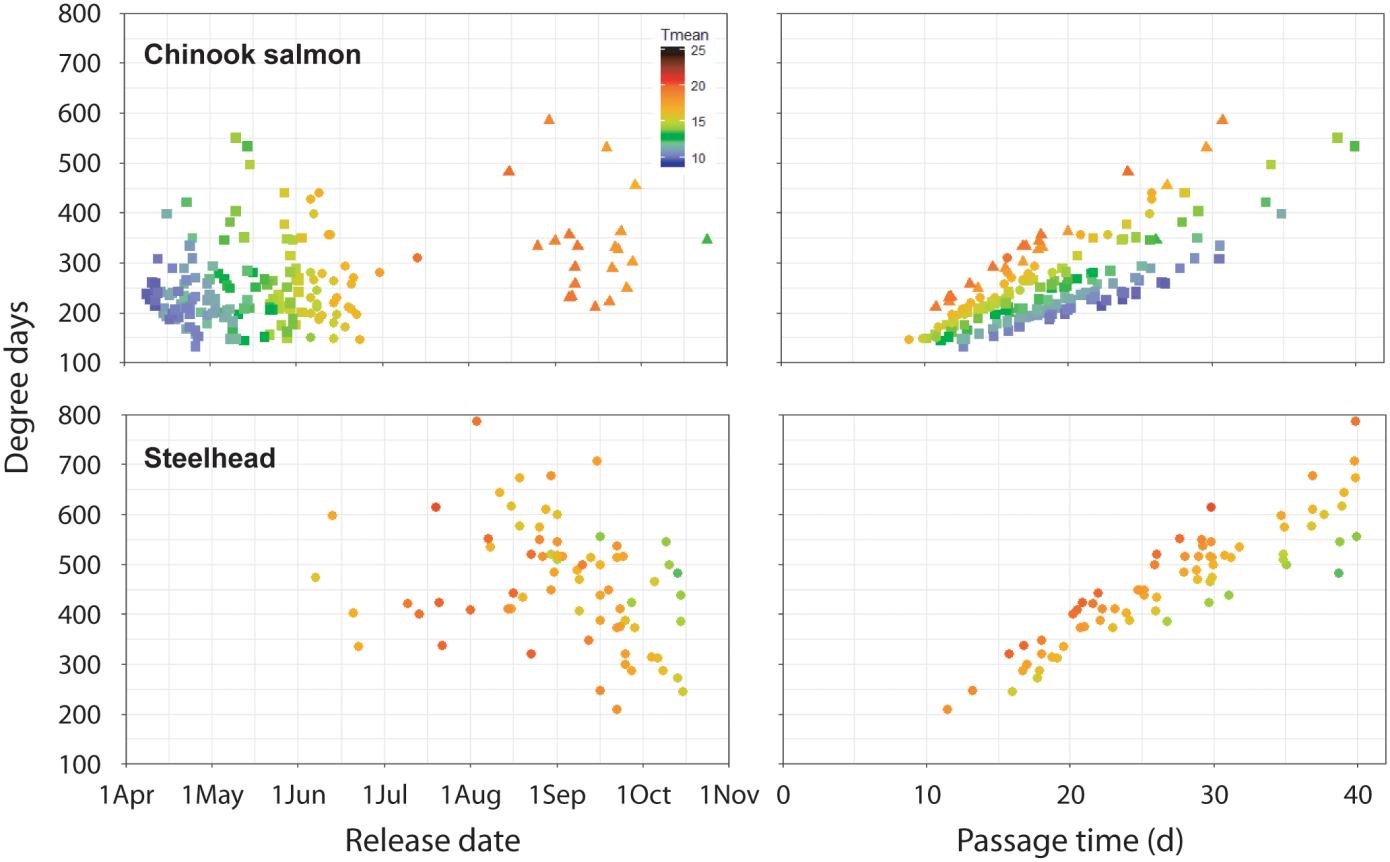
Effects of fish migration date, reach passage time, and mean body temperature on DD accumulation. Relationships between individual Chinook salmon and steelhead degree day (DD) accumulations in the ~470 rkm migration from release to Lower Granite Dam and their release dates (left) and reach passage time in days (right) in 2000 and 2002. Symbol colors represent mean fish body temperatures (Tmean) over the full reach for spring Chinook salmon (*n* = 126; squares), summer Chinook salmon (*n* = 34; circles), fall Chinook salmon (*n* = 21; triangles), and summer steelhead (*n* = 75).

Mean total DD accumulations during passage through the Columbia and Snake River migration corridor varied by a factor of two among runs, though estimates for steelhead were biased low due to the data storage limitations. Spring Chinook salmon migrated at the coolest time and accumulated 242 DD, on average (*SD* = 76, *range* = 132–550 DD). Summer Chinook salmon encountered warmer water than spring-run fish but migrated faster, resulting in accumulations (*mean* = 248 DD, *SD* = 76, *range* = 147–441 DD) that were very similar to those for spring-run salmon. Fall Chinook salmon had about a third more DD, on average, than the spring and summer runs (*mean* = 336 DD, *SD* = 102, *range* = 211–585 DD) because they migrated more slowly (on average) than summer-run fish and many also encountered seasonal high temperatures. Steelhead DD totals were highest, on average (*mean* = 462 DD) and more variable (*SD* = 115, *range* = 211–787 DD) than for any of the salmon runs, reflecting the protracted run timing and diverse migration behaviors of steelhead. DD totals for individuals within each run were strongly positively correlated (0.77 ≤ *r*^2^ ≤ 0.96) with total fish migration times, although totals still varied by a factor of ~2 for among fish with similar passage times (Fig 10). In contrast, individual fish release dates were weakly and inconsistently correlated with DD totals, with *r* values of 0.09 (spring Chinook salmon), -0.06 (summer Chinook salmon), -0.17 (fall Chinook salmon), and -0.28 (steelhead) (Fig 10).

**Fig 11:**
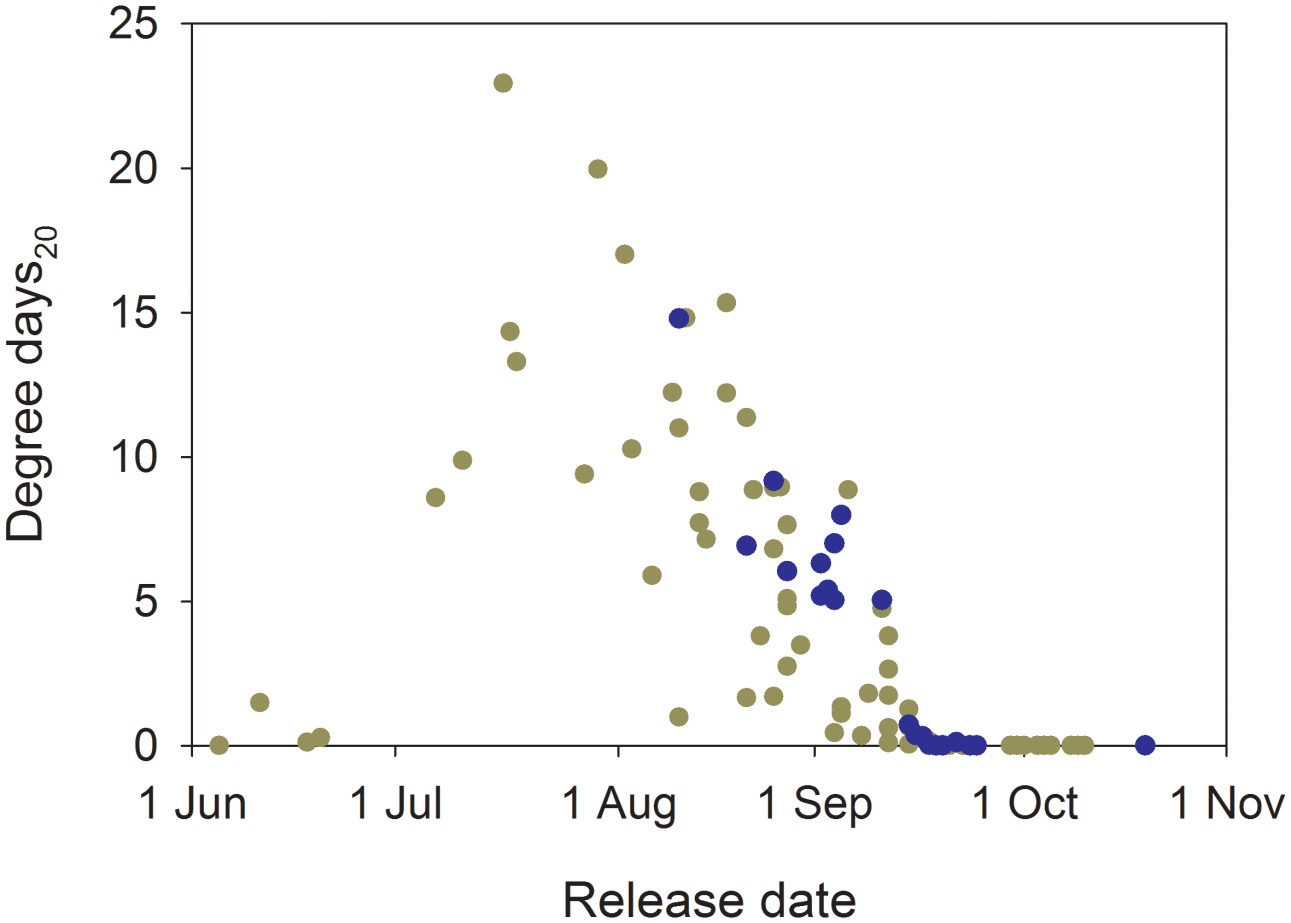
Effect of migration date on acute DD_20_ accumulation. Relationships between release date and the accumulation of acute temperature exposure measured in degree days ≥20 °C (DD_20_) for individual fall Chinook salmon (*n* = 21, blue symbols) and steelhead (*n* = 75, gold symbols) during their ~470 rkm migration from release to Lower Granite Dam in 2000 and 2002.

Cumulative acute exposure was very limited for spring and summer Chinook salmon. In total, seven fish (4.4%) from these runs had body temperatures ≥20°C, and six of the seven had < 1 DD_20_. In contrast, 76.2% of fall Chinook salmon and 68.0% of steelhead had exposure ≥20°C, with mean cumulative exposure of 5.0 d (*range* = <1–14.8 DD_20_) for fall Chinook salmon and 6.5 d (*range* = <1–22.9 DD_20_) for steelhead. There was a clear date effect, with peak DD_20_ values in late July or August for both runs, tapering to essentially no acute exposure for fish tagged in mid-September (Fig 11).

## Discussion

### Thermal histories

The 412 thermal histories summarized here provide valuable baseline data on the thermal regimes experienced by a diverse group of Snake River Chinook salmon and steelhead. The continuous, spatially-referenced body temperature data demonstrated that: (1) exposure metrics for many Snake River adults can be readily estimated in the migration corridor using a combination of migration timing, migration duration, and river temperature data; (2) migrant behaviors and their patterns of exposure became considerably more complex as river temperatures rose; (3) the risk of high-temperature exposure predictably varied among populations and among river reaches; and (4) the adult life history of steelhead affords more opportunities for adaptive behavioral responses to stressful water temperatures than the adult life history of Chinook salmon.

Temperature exposure during long-distance upstream migration is critically important for adult salmonids because temperature can mediate or accelerate a variety of physiological, disease, and mortality processes. Warm conditions more rapidly exhaust finite energetic reserves, which salmon and steelhead are simultaneously re-allocating to sexual maturation and depleting during migration, holding, and spawning [83,84]. At the same time, stress hormone production surges [85–87], organs atrophy, and immune function is substantially reduced [88–90]. These co-occurring processes allow the proliferation of parasites and pathogens, many of which become more virulent as temperatures rise [63,91,92], significantly increasing the likelihood of premature mortality [62,93,94]. Reliable, quantitative exposure data, like those collected in this and other biotelemetry studies, can help researchers address the complex inter-relationships among temperature, senescent and disease processes, and the factors that hasten premature death [93,95–97]. Future studies should strive to more explicitly link individual exposure histories to specific fish health, survival, and fitness outcomes [86,91,96].

The Columbia–Snake RDST dataset is one of the largest ever amassed for adult salmonids, but the logistical challenges of data recovery produced some inevitable limitations. First, the data were predominantly from fish that survived through the ~470 rkm study reach. Any that died from direct or indirect effects of their migration experience, including temperature-related effects, were excluded *a priori* because their transmitters were not recovered. Second, the thermal histories were limited to the low-gradient, highly-regulated portion of the migration route, and therefore fall short of documenting the full adult migrations of the study populations. River conditions adult salmon and steelhead encounter upstream from Lower Granite Dam are more diverse, with large diel fluctuations relative to those in the regulated sections. There are important run- and population-specific differences in exposure as fish near holding and spawning sites that may strongly influence prespawn mortality or fitness. Third, the two study years were characterized by near-average Columbia and Snake River temperatures. While this is a useful starting point, data collected using a similar study design in a warmer year—or in an exceptionally warm year like 2015 [81,103]–might provide more consequential information about exposure and behavior of these threatened populations in their rapidly changing freshwater habitats.

### Migration phenology and thermal exposure: Chinook salmon

Body temperatures of spring- and summer-run Chinook salmon were the most closely aligned with ambient river temperatures. These runs largely passed through the migration corridor during the ascending thermograph and reached Lower Granite Dam before seasonal peak temperatures. Consequently, just a small portion of the summer-run fish encountered water that was > 19–20 °C, a range associated with increased stress and altered adult Chinook salmon behaviors [82,98]. In cool or near-average temperature years, it should therefore be straightforward to use the relationships identified in our dataset to predict exposure of individuals or populations of spring and summer Chinook salmon migrating through the study area. For example, thousands of adult salmon with passive integrated transponder (PIT) tags migrate through the Columbia and Snake rivers annually and are detected at hydroelectric dams [37,49,81]. The PIT-tag detections have been used to demonstrate predictable, population-specific migration timing [49,99]. The detections can also be used to precisely calculate migration duration [81,100], and the combination of timing, duration, and river temperature data would allow estimation of many of the thermal exposure metrics described here. The fish in our dataset migrated in years with near-average thermal conditions, so modeled exposure estimates will require additional assumptions in warmer years when more spring and summer-run salmon encounter stressful temperatures that may elicit different behavioral responses.

Snake River fall Chinook salmon migrate earlier, on average, than some of the more abundant Columbia River fall-run populations [101]. Half of our fall-run sample was tagged before 7 September, and the thermal regime experienced by many individuals therefore included seasonal peak or near-peak temperatures. About two-thirds encountered water >20 °C and some body temperatures exceeded 22 °C. The fall Chinook salmon RDST histories corroborated results from a previous mixed-stock radiotelemetry study where salmon migrated much more slowly and increasingly occupied cool-water refuges when Columbia River temperatures reached a ~20–21 °C threshold [52]. One of the lingering uncertainties from our study is whether the fall Chinook salmon strategies of slowed migration and thermal refuge use will be used by spring- or summer-run Chinook salmon that encounter warmer future conditions. Biotelemetry studies in the Willamette River (Oregon) and Klamath River (Oregon-California) suggest that accelerated migration may be a more common strategy than thermoregulation for adult spring- and summer-run Chinook salmon that encounter thermal stress along migration routes [50,102]. It also remains unknown whether differences in maturation status, genotype, or phenotype are associated with population-specific differences in thermal tolerance.

In the Columbia River main stem, the ~2.5 °C increase in summer water temperatures over the last several decades [21,25] is a potentially powerful selective pressure on Chinook salmon phenology. All three seasonal runs have likely experienced historically high mean, maximum, and cumulative exposure in recent years [81,103], and ongoing selection against fish with low thermal tolerance is widely expected in the Columbia basin and in the PNW [23,60,104–106]. Adaptive responses may include a shift to earlier migration (spring-, summer-, and some fall-run salmon) or later migration (fall-run salmon) to avoid the most stressful conditions in the migration corridor. Indeed, there is compelling evidence for a 1–2 week shift towards earlier migration for Columbia and Snake River sockeye salmon (*O*. *nerka*) populations [21,25,28]. Sockeye salmon co-migrate with summer Chinook salmon and migrate similar distances to spawning grounds, and we think parallel selective changes are likely occurring for these two species. There is also some evidence for a temperature-related phenological shift for Columbia River fall Chinook salmon: in recent years run timing distributions have flattened, with proportionately more fish enumerated both before and after the historical run peak [52].

River warming in the PNW is likely eliciting a mix of phenotypically plastic responses and genetic adaptation by Chinook salmon, but it is uncertain whether these changes can keep pace with river warming or whether phenological shifts may simply exacerbate other risks [107]. For spring- and summer-run salmon, the benefits of migrating early to avoid warm water in the Columbia and Snake River migration corridors may be offset by early arrival at spawning sites and longer prespawn holding times. Prespawn mortality, where fish reach spawning grounds but die before reproducing, has been the focus of considerable recent research in the PNW [93,108–110], and warm temperature exposure has repeatedly been identified as increasing this mortality risk for Chinook salmon [63,94,111]. Hypothetically, fall Chinook salmon may have greater opportunity for phenological adaptation than their spring- and summer-run counterparts. A shift to later fall migration would reduce warm water exposure, while costs associated with later arrival at spawning sites (i.e., reduced mating opportunity) may be ameliorated by concurrent shifts to later cooling of spawning sites and later spawn dates.

### Migration phenology and thermal exposure: Steelhead

The Snake River supports a genetically and phenotypically diverse group of summer steelhead populations whose adult migration timing has been an important trait for structuring management and conservation decisions [112–114]. Historically, steelhead run timing at Bonneville Dam was bimodally distributed, with a passage nadir in late summer when river temperatures were near seasonal highs [54]. Timing of the aggregate run has dramatically shifted in recent decades to a more unimodal distribution with peak steelhead abundance coinciding with near-peak summer water temperatures (see Fig 2). The changes have been attributed to a variety of demographic and environmental factors, including loss of native stocks, proliferation and broad distribution of hatchery stocks (predominately early-run fish), harvest impacts, and development and operation of the hydropower system [53,54,114]. Despite substantive phenological changes for the metapopulation, many individual steelhead stocks maintain distinctive migration timing in the lower Columbia and Snake rivers that strongly affect their thermal exposure and migration behaviors [58,59,113]. The relative influence of temperature- or climate-related selection for specific phenotypes within populations is currently unknown.

Previous research identified ~19 °C as an important behavioral threshold temperature for adult steelhead in the Columbia River basin [58]. Most fish that migrate before the main stem reaches ~19 °C or migrate after temperatures decline below this threshold in the fall move rapidly upstream, whereas those that encounter temperatures above the threshold tend to dramatically slow migration and behaviorally thermoregulate in reaches where cooler water is available [56–58,79]. In the RDST dataset, more than 90% of steelhead encountered temperatures ≥19 °C and more than half had maxima ≥21 °C, patterns that were very similar to those of many co-migrating fall Chinook salmon. However, the hyper-premature life history of steelhead allowed prolonged use of cool-water refuges, resulting in dramatically cooler mean body temperatures for steelhead relative to fall Chinook salmon. This was most pronounced in reach 2 where there were many thermal refuge sites. Thermoregulatory behavior in reaches 1–3 significantly postponed steelhead migration timing at upstream sites, and total migration times from release to the Lower Granite trap averaged nearly three times longer for steelhead (~60 d) than for fall Chinook salmon (~18 d). Some RDST-tagged steelhead arrived at Lower Granite Dam in late October and November, after many fall Chinook salmon had likely completed spawning, and ~6% of steelhead overwintered along the migration route and had migration times to Lower Granite trap of 150–255 d. Remarkably, the overwintering group likely encountered both annual thermal maxima (20–22 °C) and minima (4–6 °C, [75]) in the migration corridor.

Understanding how river warming in the PNW has affected the migration phenology of Snake River steelhead is challenging given the large demographic changes in the aggregate population and their spatially and temporally complex migration behaviors. Nonetheless, we think it is highly probable that Snake River steelhead are currently: (1) more likely to encounter stressful water temperatures during migration than historically; (2) more likely to encounter warm water earlier and later in the season and further downstream than historically; and (3) more likely to routinely employ behavioral thermoregulation as a temperature-mitigating tactic than historically. Collectively, these changes may affect adult mortality risks such as energetic costs, disease proliferation, and harvest in thermal refuge areas [58,115,116]. The changes may also further affect steelhead phenology at upriver sites and reduce the fertility or fitness of those that survive to spawning grounds [27,117,118]. We expect that temperature-tolerant phenotypes will be favored if warming continues and that the demographics of the aggregate population will continue to shift away from the historically diverse mix.

### Spatial patterns of exposure: Among-run differences

The studied runs encountered thermal minima and maxima in distinctly different river reaches due to a combination of migration timing, seasonal river warming and cooling, and the patchy distribution of cool-water refuges. Many spring- and most summer-run Chinook salmon were warmest in the lower Snake River, whereas most fall Chinook salmon and steelhead were warmest in the lower Columbia River. Patterns were not quite reversed for cool water exposure: low body temperatures for spring and summer Chinook salmon were distributed among multiple reaches, were concentrated in the uppermost Snake River reaches for fall Chinook salmon, and were predominantly in reaches 2 (thermal refuges) and 7 (late arrival timing) for steelhead. Exposure differences among runs suggest that mitigation strategies may need to be tailored to specific reaches or dams to effectively address temperature-related migration challenges like passage delay or mortality.

Some of the warmest temperatures for RDST-tagged fish were associated with adult fishways at the dams, and the fishways are one of the few possible sites for temperature-management intervention. Warm exposure inside fishways was typically short duration, but adult salmon and steelhead that encounter warm fishways have been shown to delay passage at the Snake River dams [67], at John Day Dam on the lower Columbia River, and presumably at other dams given the likely universality of the reported mechanism (i.e., sharp thermal gradients that prompt fish to reject fishways). Concern over dam passage delays recently prompted design and installation of a fishway cooling system at Lower Granite Dam, where water associated with hypolimnetic releases from Dworshak Dam, a high-head dam on the North Fork Clearwater River [119], is pumped from the Snake River into the Lower Granite adult fish ladder. Similar water management systems may need to be prioritized at other dams if the Columbia and Snake rivers continue to warm as expected [33,34,36]. Unfortunately, cool-water sources are limited or non-existent near several dams because significant thermal stratification is uncommon in adjacent reservoirs [69,80]; alternative cooling strategies may need to be considered in these locations.

### Behavioral plasticity: Faster migration versus thermoregulation

The thermoregulation evident in the fall Chinook salmon and steelhead histories confirm previous behavioral findings [52,56–59] but provide far more quantitative details on how much body temperatures differed from ambient conditions along the migration route. As expected, steelhead extensively used thermal refuges in reaches 1–3, where cool- and cold-water tributaries drain the Cascade Range, and in reach 7 where a cold-water hatchery outfall attracts many adult migrants [79]. The coldest thermal refuges were in reaches 1–2, where fish temperatures were frequently 3–10 °C cooler than the main stem Columbia River. Salmon and steelhead in the Deschutes River refuge (reach 3) were typically 1–5 °C cooler than in the Columbia River. The RDST histories additionally indicated that the Deschutes River was the only significant thermal refuge in the >250-km reach from The Dalles Dam to Lower Monumental Dam. The paucity of refuges upstream from the Deschutes River confluence portends that significant thermal barriers to adult migration may occur in this reach in future warmer years. Similar thermal bottlenecks have threatened the sustainability of salmon populations in other locations [105,120,121].

The prolonged use of thermal refuges by many Snake River steelhead exemplifies the behavioral plasticity afforded this species by hyper-premature migration. As noted above, demographic and environmental changes appear to have made refuge use by steelhead an increasingly necessary tactic for surviving the Columbia River–Snake River migration corridor. It remains to be seen, however, whether reliance on patchily-distributed, cold-water habitats 100s of km downstream from spawning sites will be an evolutionarily sustainable response to river warming. The myriad risks of extended prespawn holding [47,58,122,123] may eventually outweigh the benefits of early freshwater entry and mid-summer migration. We presume that phenotypically plastic behaviors like thermoregulation are adaptive, but also that evolutionary pressures will result in selection for Snake River steelhead with higher thermal tolerance, altered migration and spawning phenology, and perhaps other temperature-related traits [14,24,107,124].

Chinook salmon behaviors in response to warm conditions provided a stark contrast to steelhead. Refuge use by fall Chinook salmon appeared to be a behavior of last resort, occurring only when ambient main stem temperatures were ≥20–21 °C, and then only for brief periods. A few spring Chinook salmon encountered water >18 °C and a few summer Chinook salmon encountered temperatures >20 °C but we detected no apparent behavioral thermoregulation by fish in these runs in the refuge sites associated with tributary confluences or deep in the reservoirs. We hesitate to speculate about the degree to which spring and summer Chinook salmon will seek thermal refuge along the migration route in years that are warmer. However, several recent studies of obligate summer- and fall-spawning Pacific salmon populations suggest that behavioral thermoregulation distant from spawning sites may not be a particularly viable strategy [50,103,121,125]. On the other hand, the well-documented thermoregulatory behaviors that occur near natal sites during the final stages of maturation [126–131] will potentially be increasingly important for these populations.

Increased migration rate is an alternative behavioral strategy that Chinook salmon could employ to minimize exposure to acute or cumulative thermal stress, but this may invoke unrealistically high energetic demands. Snake River Chinook salmon already move very rapidly through reservoirs, with median rates ranging from ~50–70 rkm/d (spring-run) and ~60–80 rkm/d (summer-run) [57,81]. These rates are likely approaching optimal swim speeds, where metabolic scope for activity is maximized and energetic costs are minimized relative to distance traveled [60,132,133]. Salinger and Anderson [61] estimated that swim speeds of Columbia River Chinook salmon were maximized at ~16 °C, and declined above this threshold. If 16 °C is a reasonable estimate of the physiologically optimal migration temperature for Snake River Chinook salmon, we think there is considerable remaining scope for faster migration by spring-run fish but relatively limited additional scope for summer-run fish given the temperature histories in the RDST dataset. That said, there is currently very little aerobic scope information for the diverse spring–summer Chinook salmon populations in the Snake River. Studies of other Pacific salmonids have shown that aerobic scope, cardiovascular performance, optimal swim speeds, and temperature optima all vary among populations within species [60,105,106,134]. Quantifying similar relationships among physiological performance and temperature for Snake River Chinook salmon populations would greatly improve our ability to predict whether faster migration through the corridor is possible. If these populations do reach spawning sites more rapidly, the benefits must be weighed against the potential hazards associated with longer prespawn holding in tributaries. Anticipating how climate warming will affect adult survival and fitness of these threatened populations will depend on how well we understand the complex interactions among acute and cumulative temperature exposure, the timing and location of exposure, and the many ways that population-specific impacts are likely to vary.

## Supporting information

S1 Appendix Chinook salmon and steelhead thermal histories collected in 2000. Graphs show individual body-temperature histories, color coded by river reach. The run (spring Chinook salmon, summer Chinook salmon, fall Chinook salmon, steelhead) and RDST number are shown for each fish.
(PDF)

S2 Appendix Chinook salmon and steelhead thermal histories collected in 2002. Graphs show individual body-temperature histories, color coded by river reach. The run (spring Chinook salmon, summer Chinook salmon, fall Chinook salmon, steelhead) and RDST number are shown for each fish.
(PDF)

S3 Appendix Fish origin and fate metadata. Five tables summarizing the final adult Chinook salmon and steelhead detection locations (Tables S1-S4) and juvenile PIT-tag locations (Table S5) of all RDST-tagged Snake River study fish.
(PDF)
