# Supplementary material for: Thermal exposure of adult Chinook salmon and steelhead: Diverse behavioral strategies in a large and warming river system
Source: PLoS One. 2018 Sep 21;13(9):e0204274. doi: 10.1371/journal.pone.0204274 (PMC6150539; doi:10.1371/journal.pone.0204274)

Fall Chinook
2577E

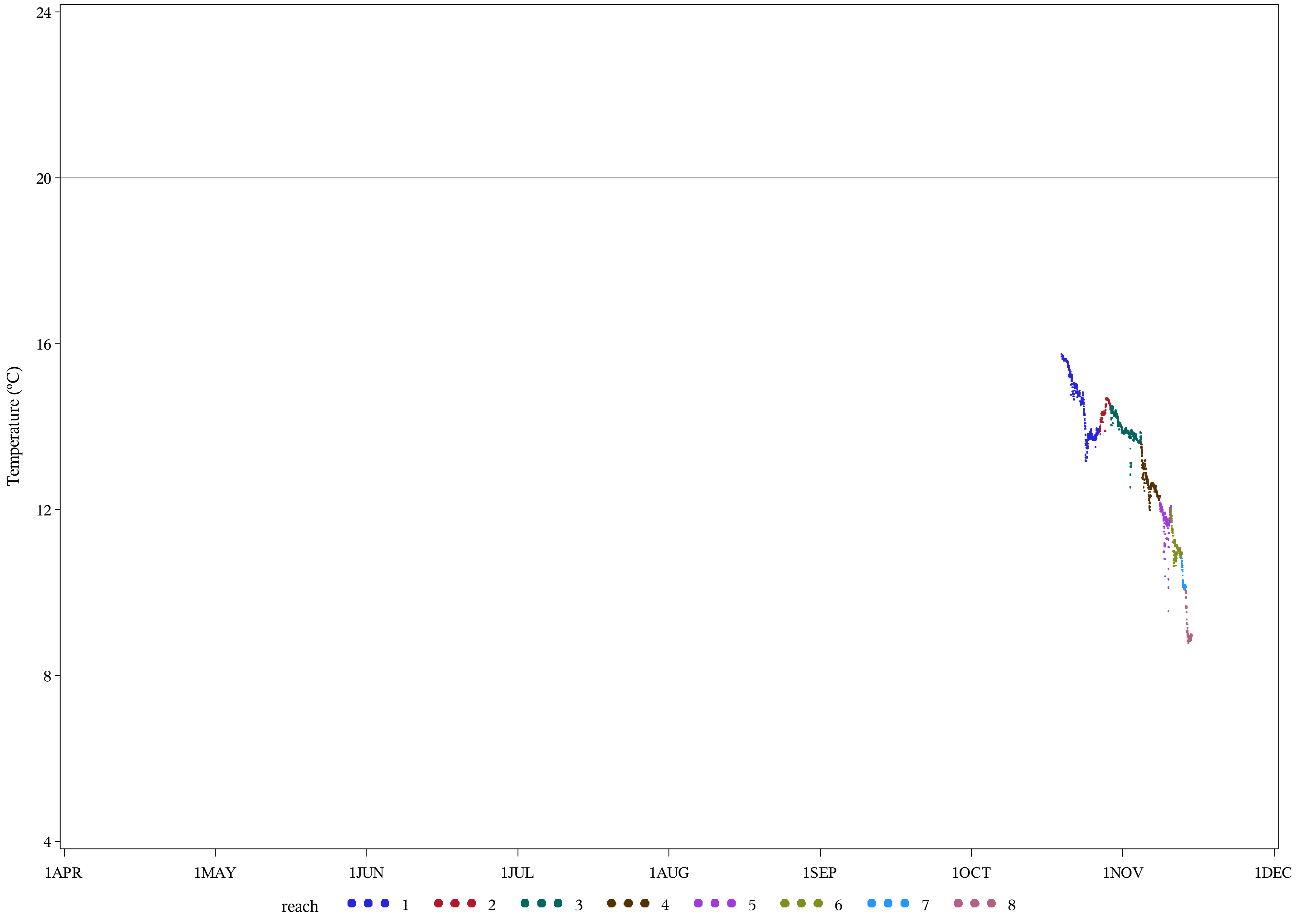

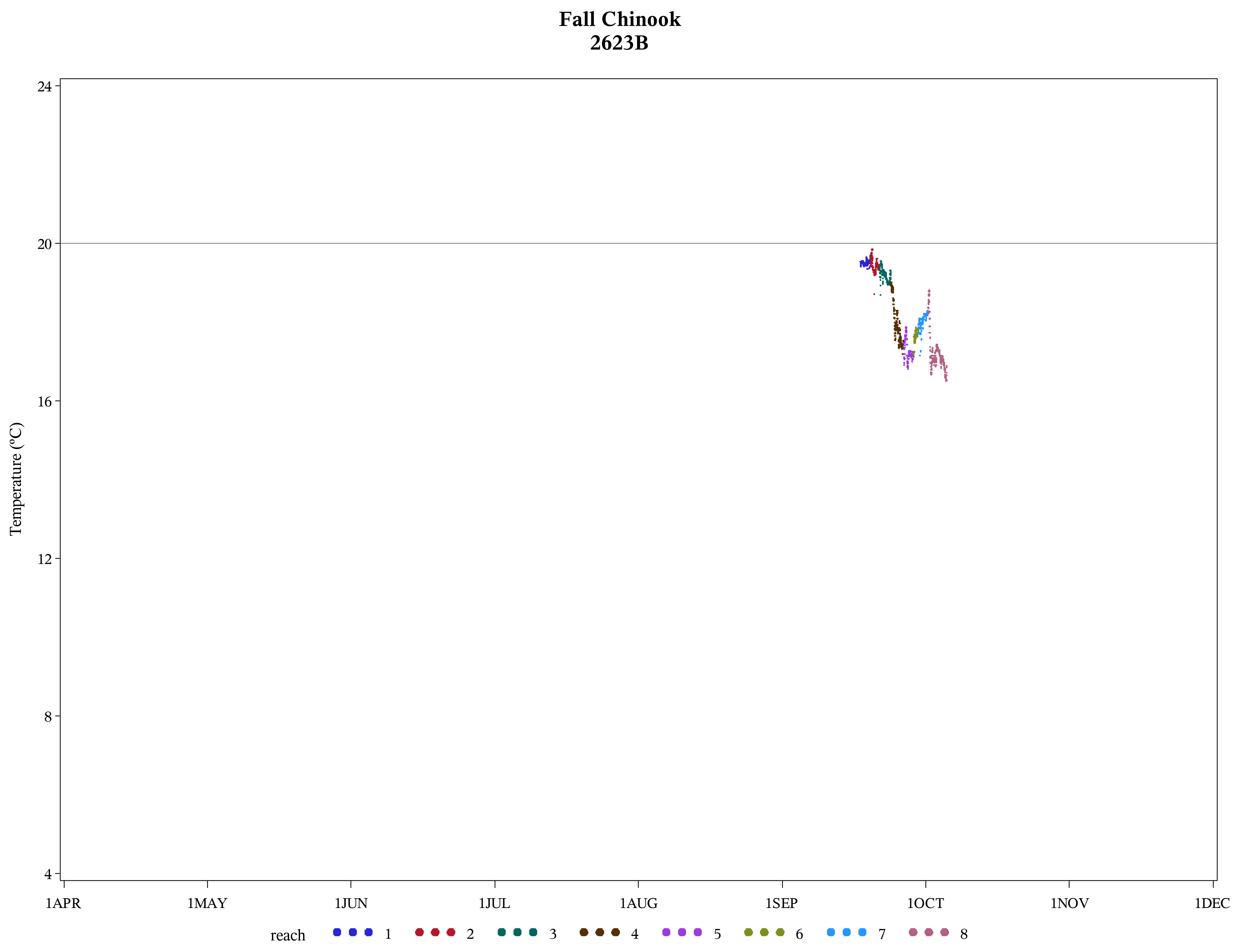
Fall Chinook
2623B
Temperature (°C)
24
20
16
12
8
4
1APR
1MAY
1JUN
1JUL
1AUG
1SEP
1OCT
1NOV
1DEC
reach
1
2
3
4
5
6
7
8

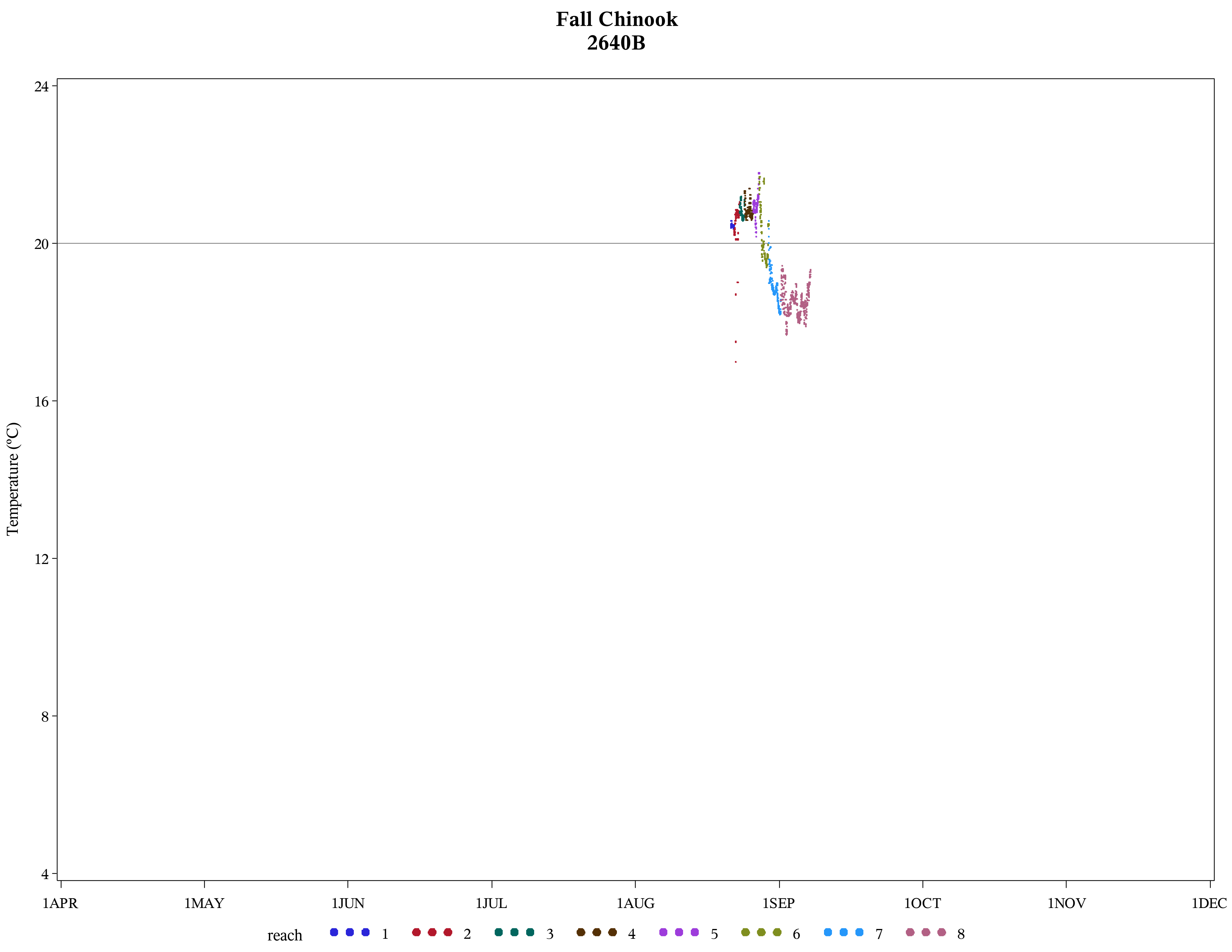
Fall Chinook
2640B
Temperature (°C)
24
20
16
12
8
4
1APR
1MAY
1JUN
1JUL
1AUG
1SEP
1OCT
1NOV
1DEC
reach
1
2
3
4
5
6
7
8

Fall Chinook
2676B

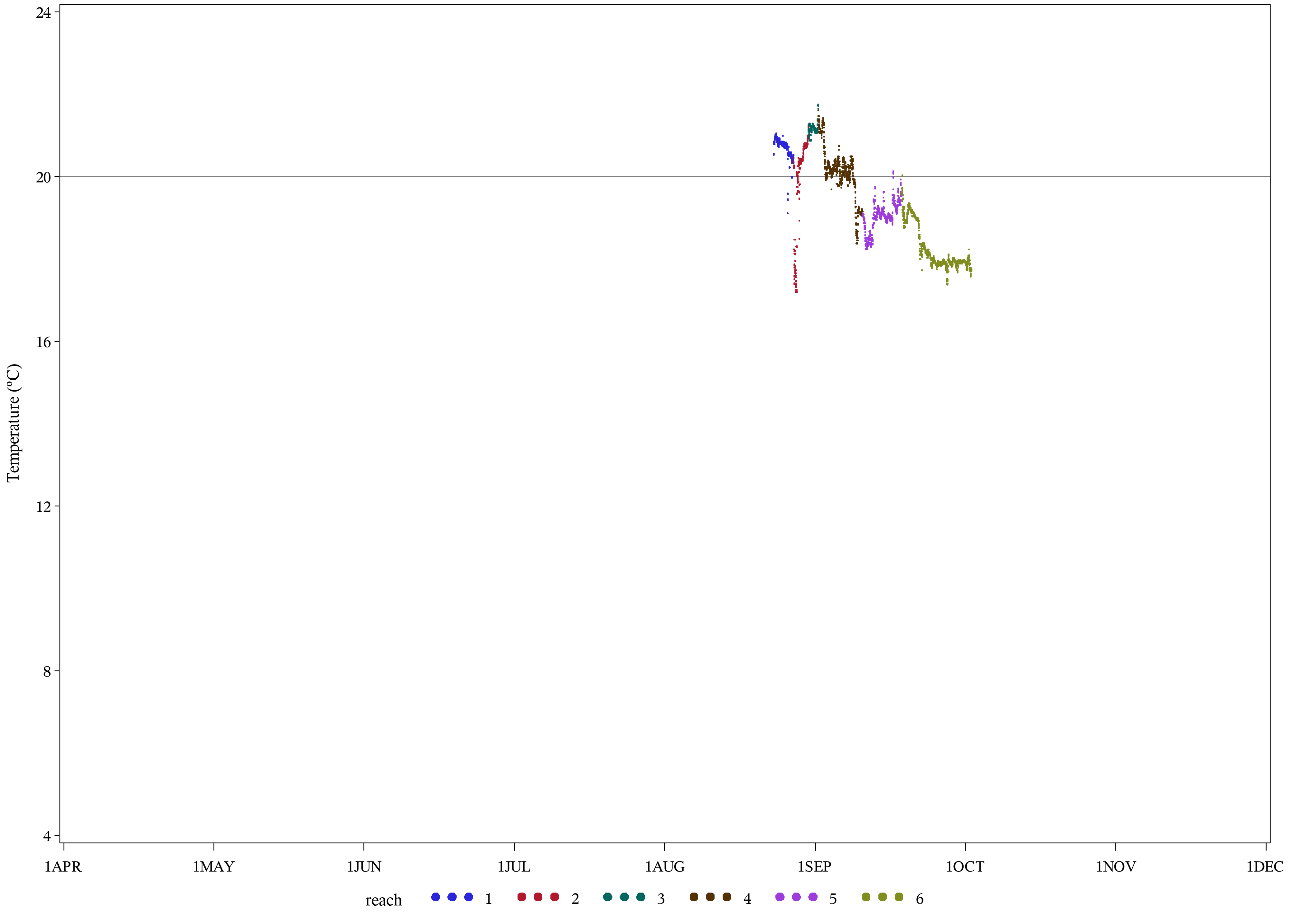

Fall Chinook
2725A

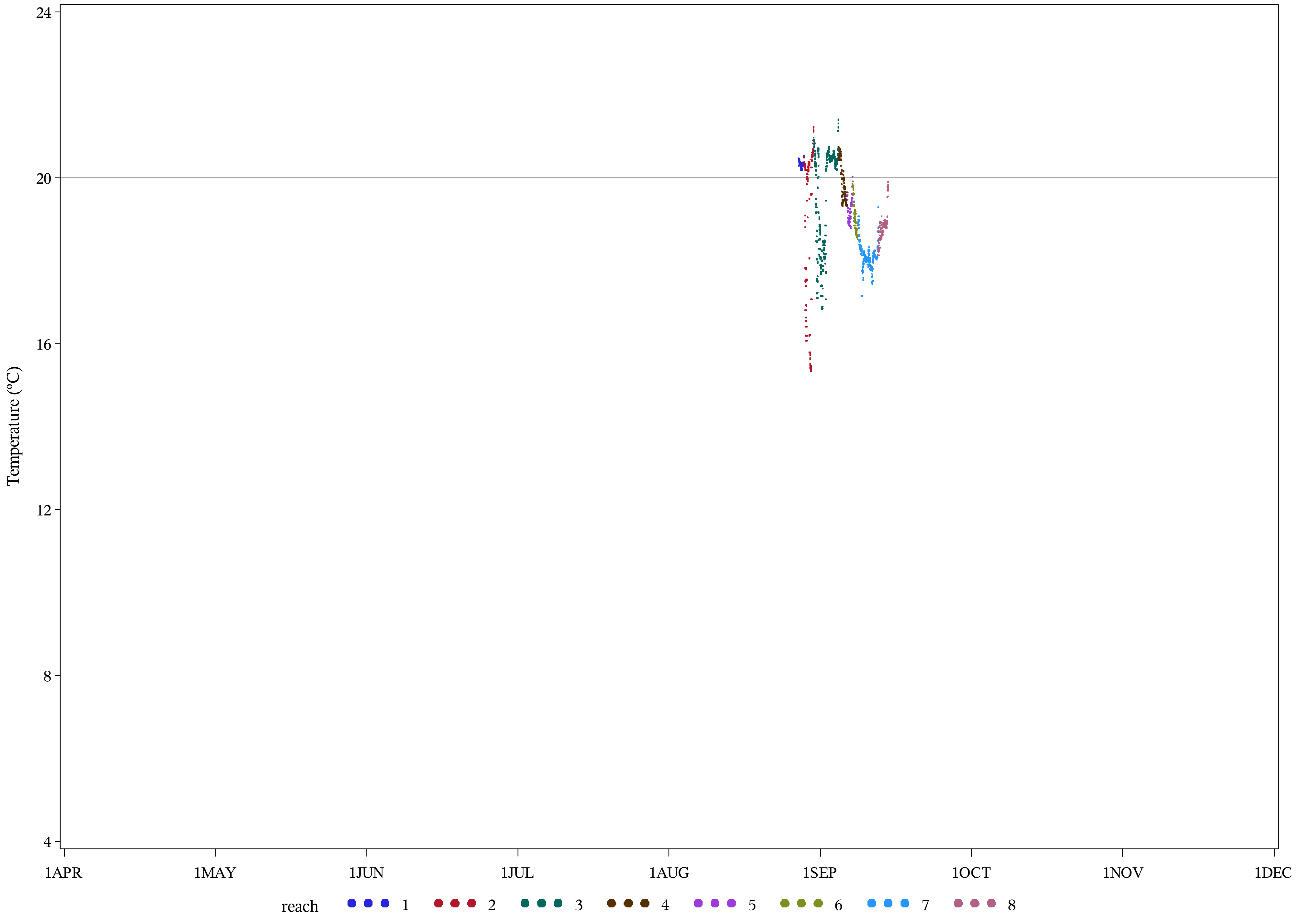

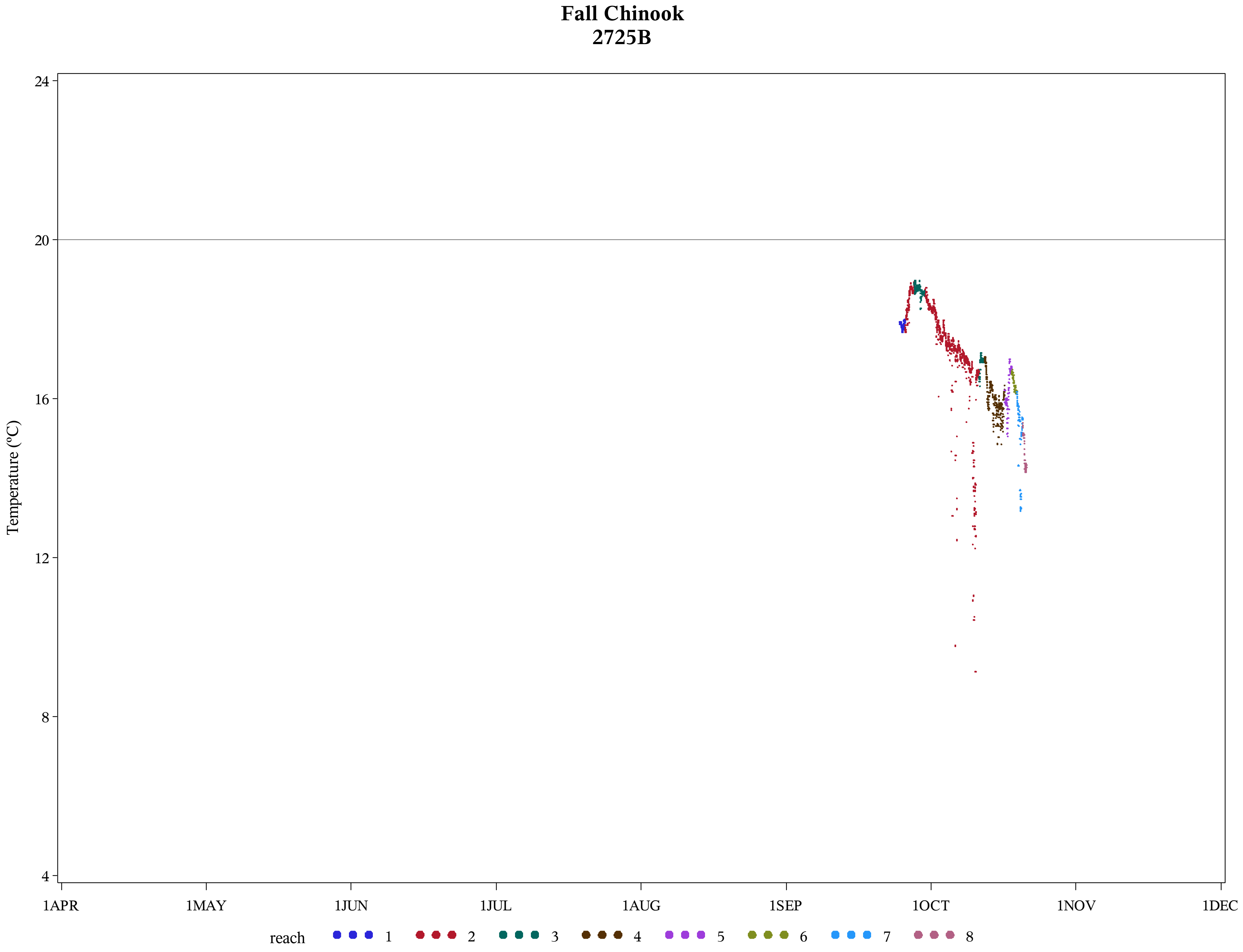
Fall Chinook
2725B
Temperature (°C)
24
20
16
12
8
4
1APR
1MAY
1JUN
1JUL
1AUG
1SEP
1OCT
1NOV
1DEC
reach
1
2
3
4
5
6
7
8

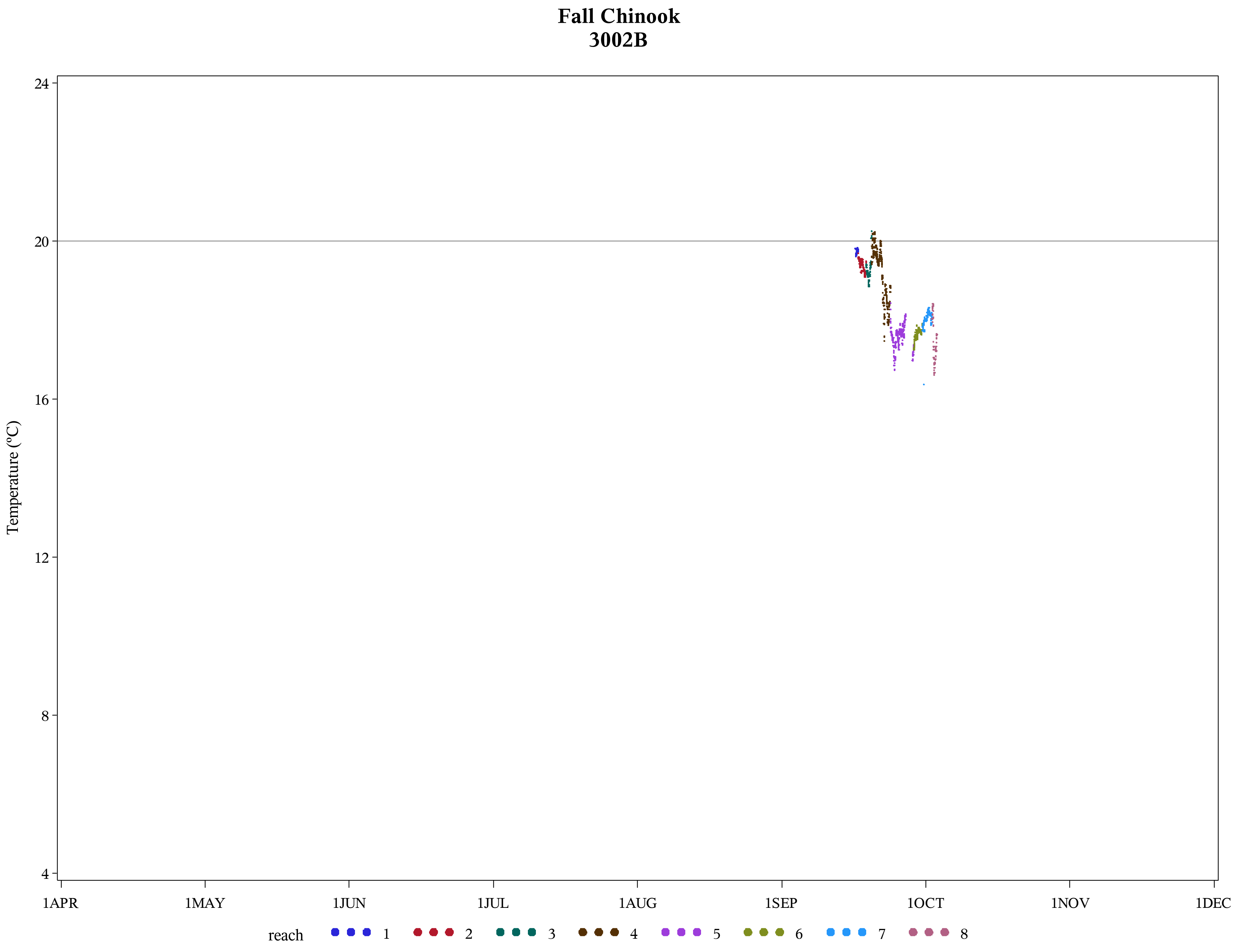
Fall Chinook
3002B
Temperature (°C)
24
20
16
12
8
4
1APR
1MAY
1JUN
1JUL
1AUG
1SEP
1OCT
1NOV
1DEC
reach
1
2
3
4
5
6
7
8

Fall Chinook
3596B

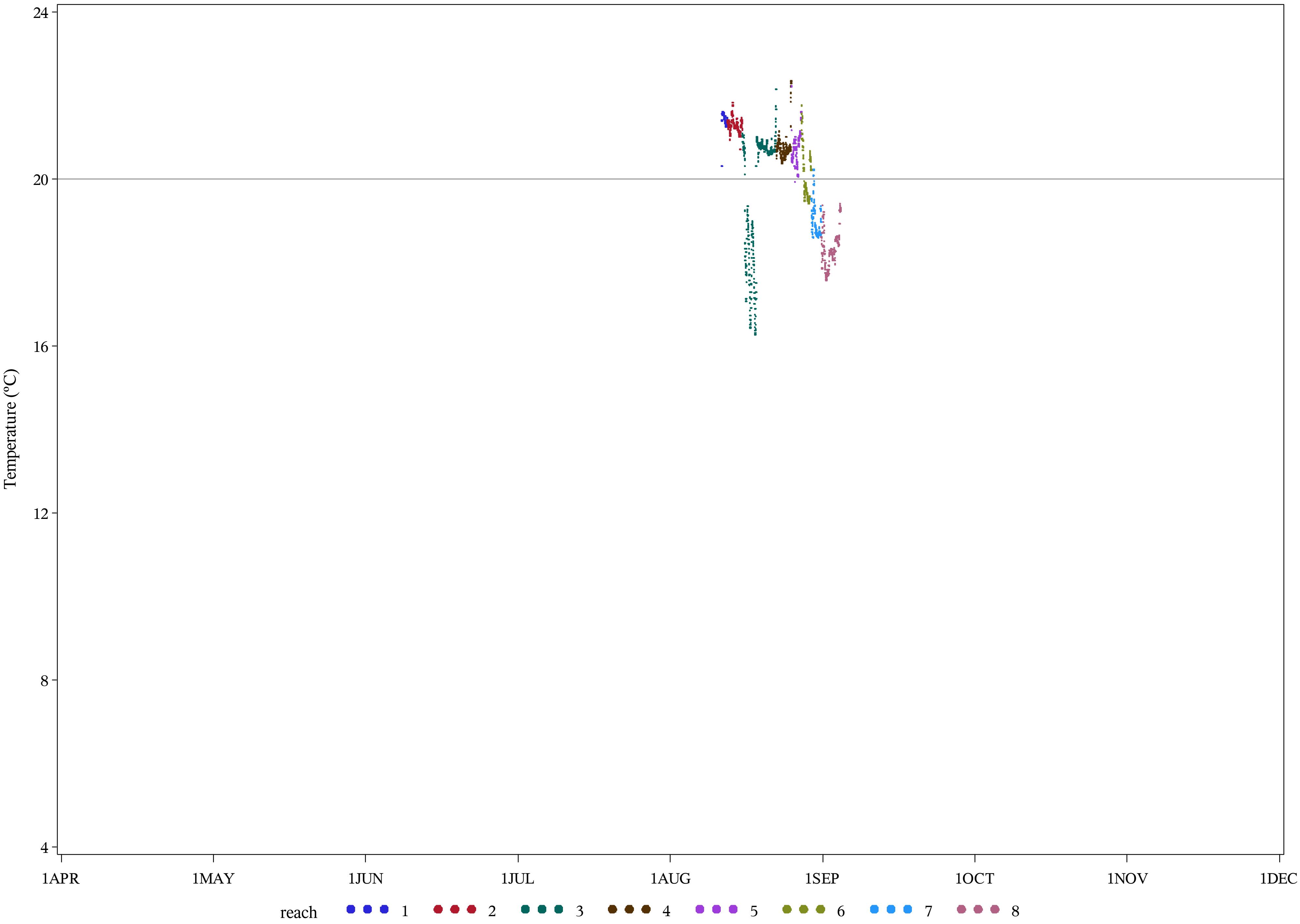

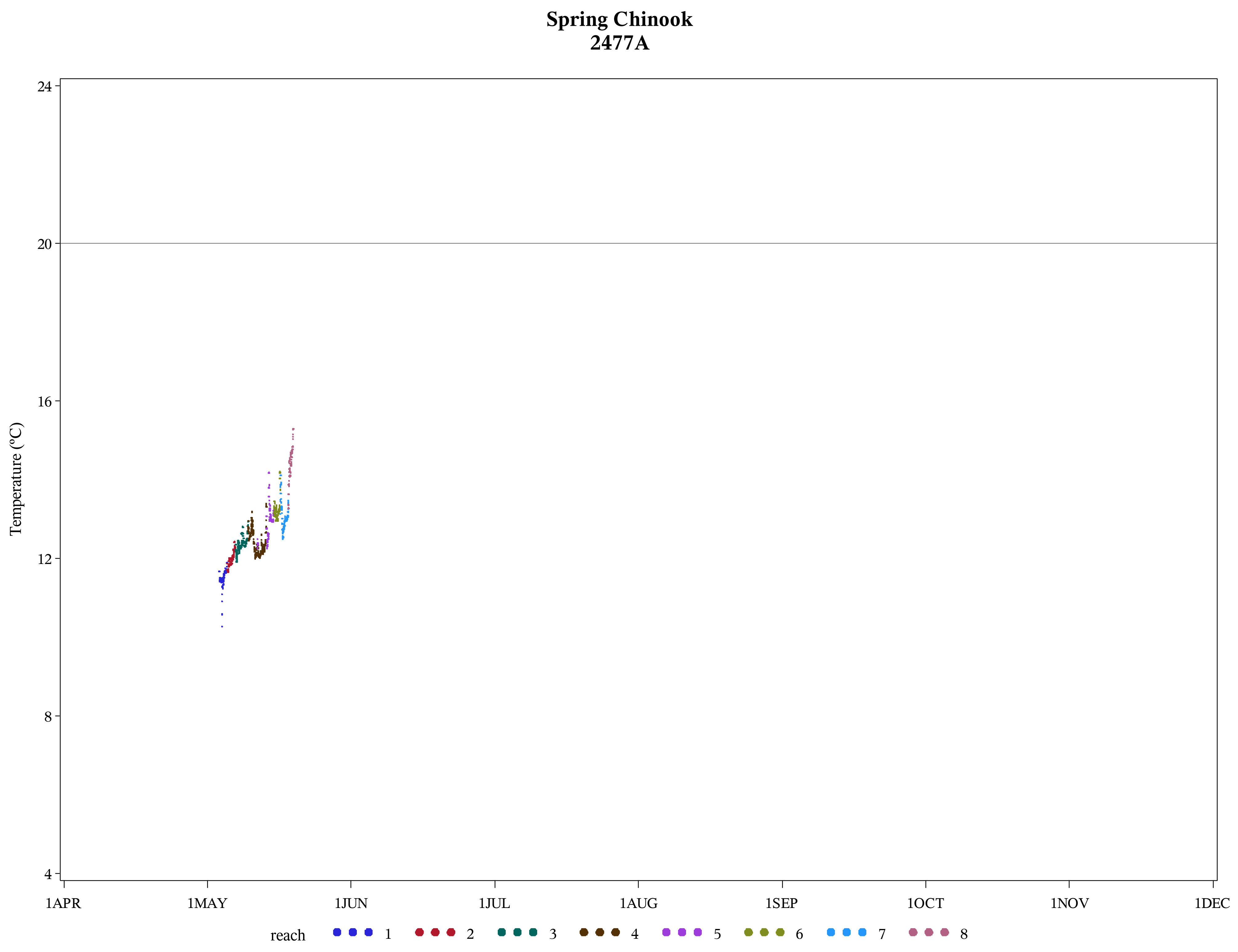
Spring Chinook
2477A
Temperature (°C)
24
20
16
12
8
4
1APR
1MAY
1JUN
1JUL
1AUG
1SEP
1OCT
1NOV
1DEC
reach
1
2
3
4
5
6
7
8

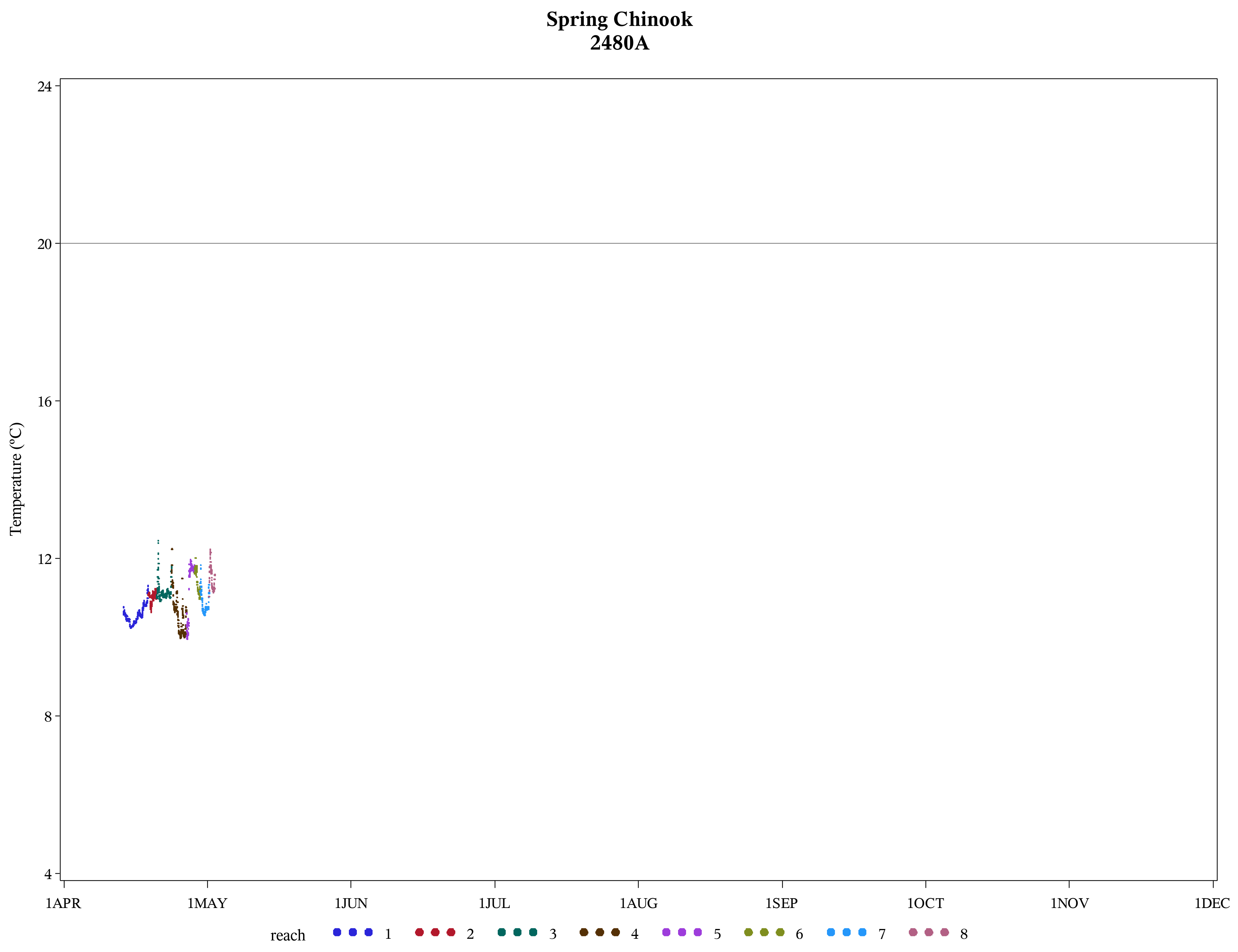
Spring Chinook
2480A
Temperature (°C)
24
20
16
12
8
4
1APR
1MAY
1JUN
1JUL
1AUG
1SEP
1OCT
1NOV
1DEC
reach
1
2
3
4
5
6
7
8

Spring Chinook
2480B

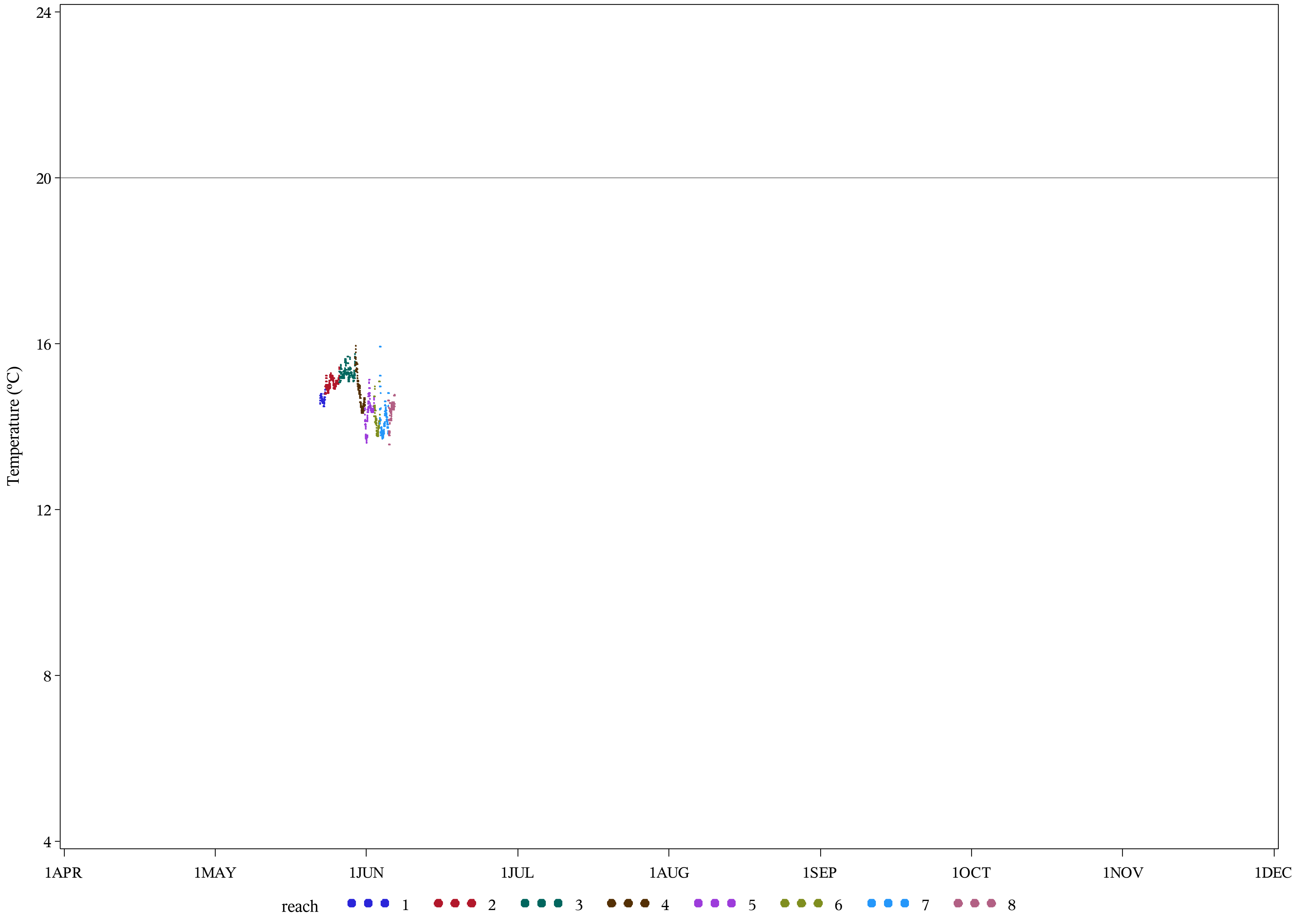

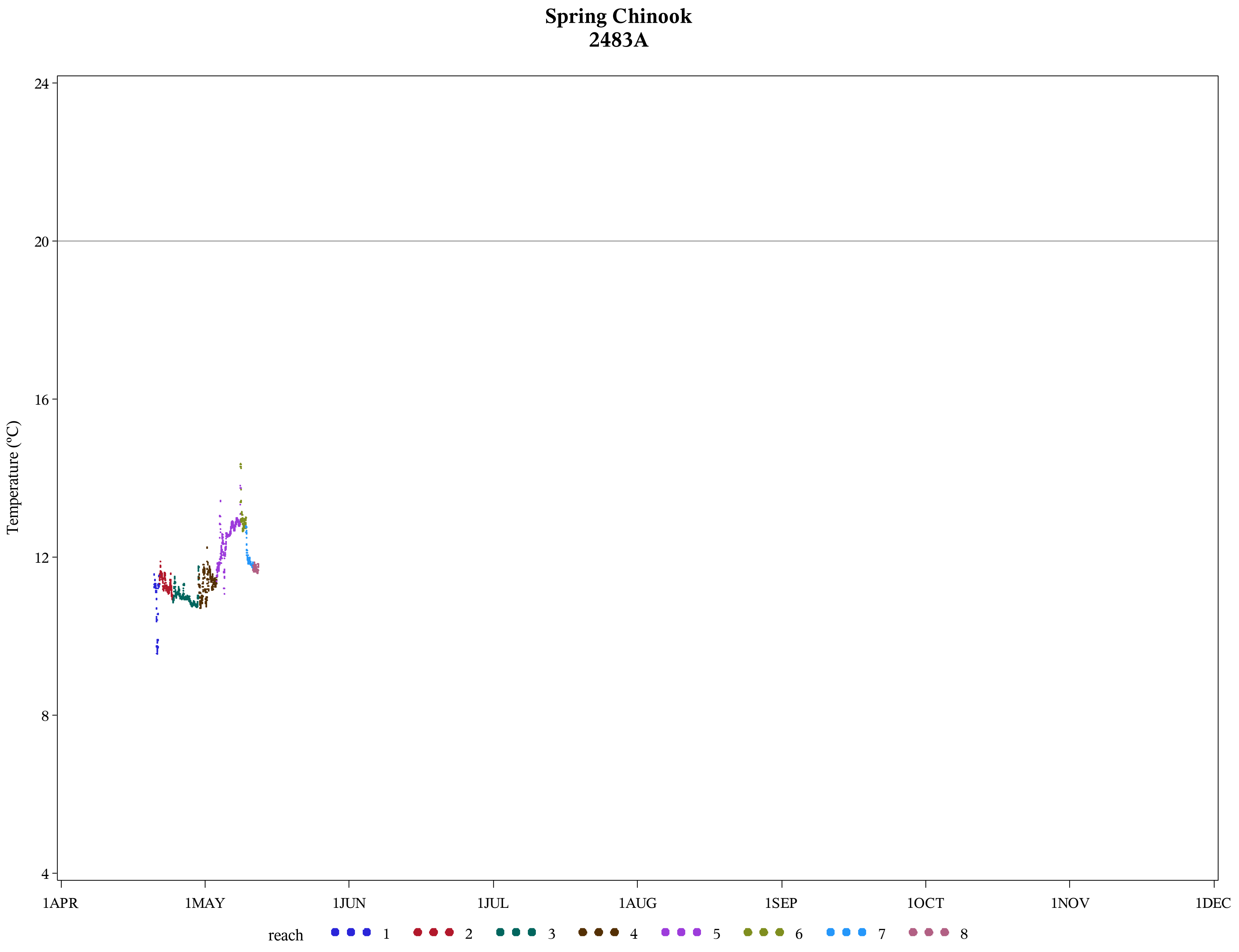

Spring Chinook
2483A
Temperature (°C)
24
20
16
12
8
4
1APR
1MAY
1JUN
1JUL
1AUG
1SEP
1OCT
1NOV
1DEC
reach
1
2
3
4
5
6
7
8

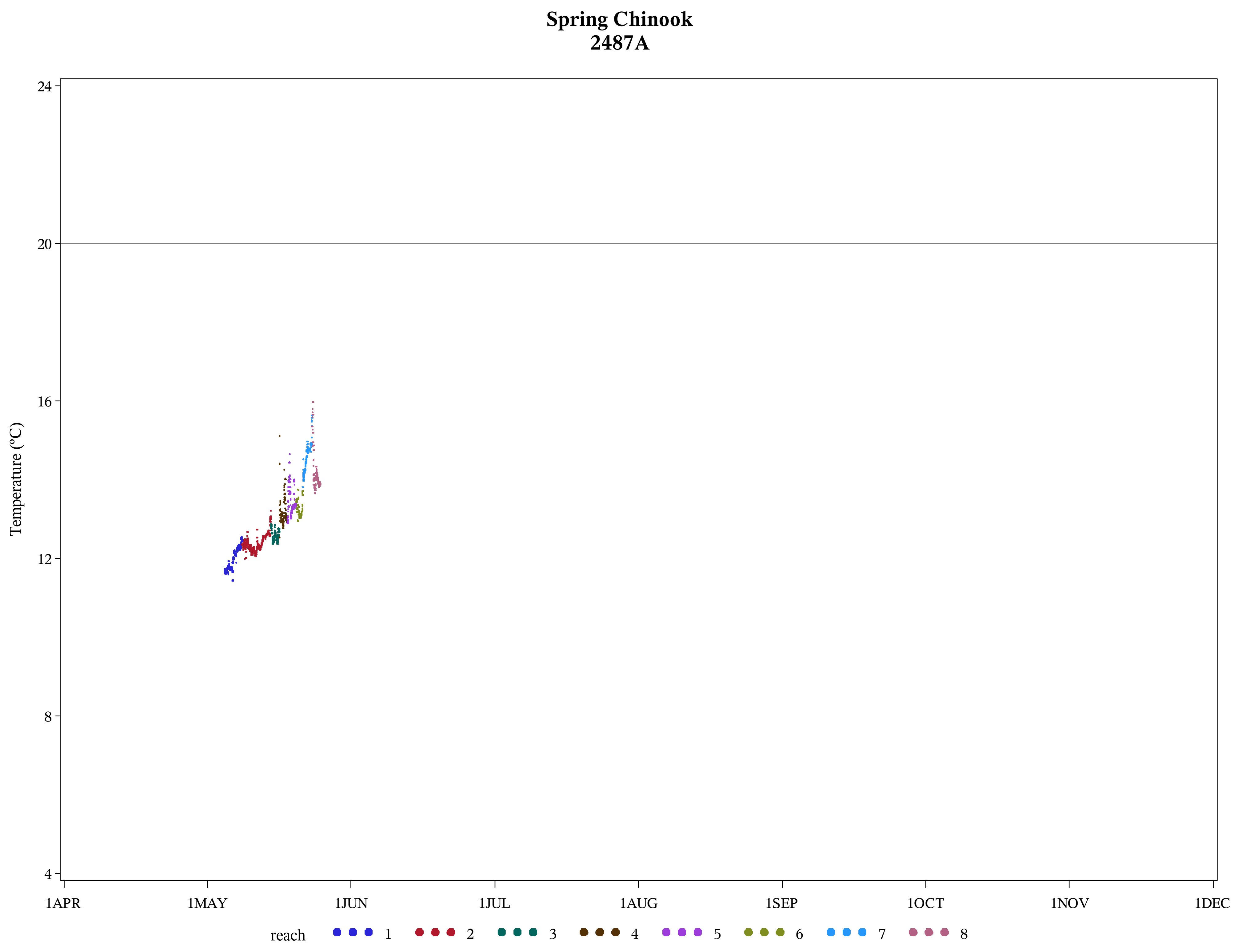
Spring Chinook
2487A
Temperature (°C)
24
20
16
12
8
4
1APR
1MAY
1JUN
1JUL
1AUG
1SEP
1OCT
1NOV
1DEC
reach
1
2
3
4
5
6
7
8

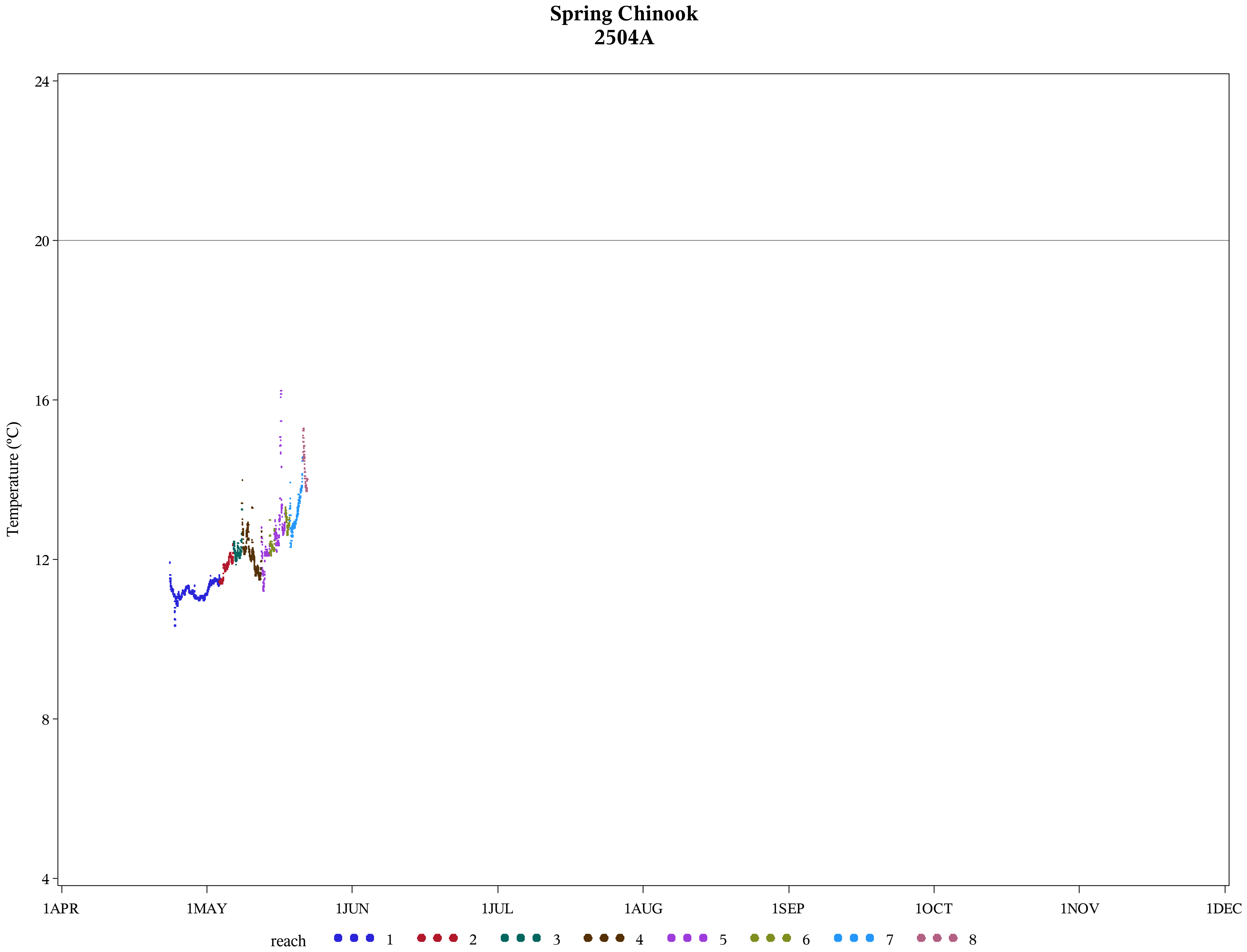

Spring Chinook
2504A
24
20
16
12
8
4
Temperature (°C)
1APR
1MAY
1JUN
1JUL
1AUG
1SEP
1OCT
1NOV
1DEC
reach
1
2
3
4
5
6
7
8

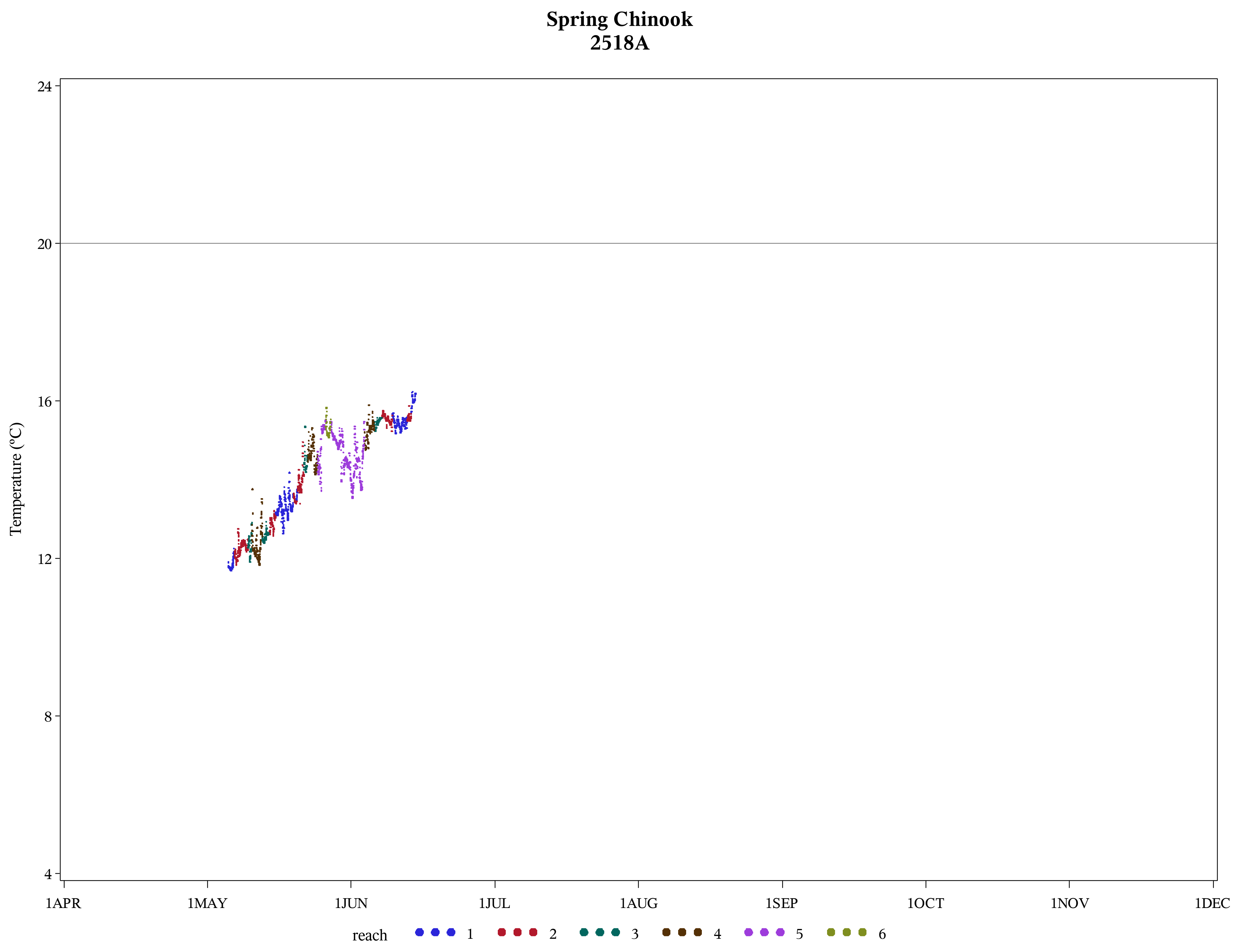

Spring Chinook
2518A
Temperature (°C)
24
20
16
12
8
4
1APR
1MAY
1JUN
1JUL
1AUG
1SEP
1OCT
1NOV
1DEC
reach
1
2
3
4
5
6

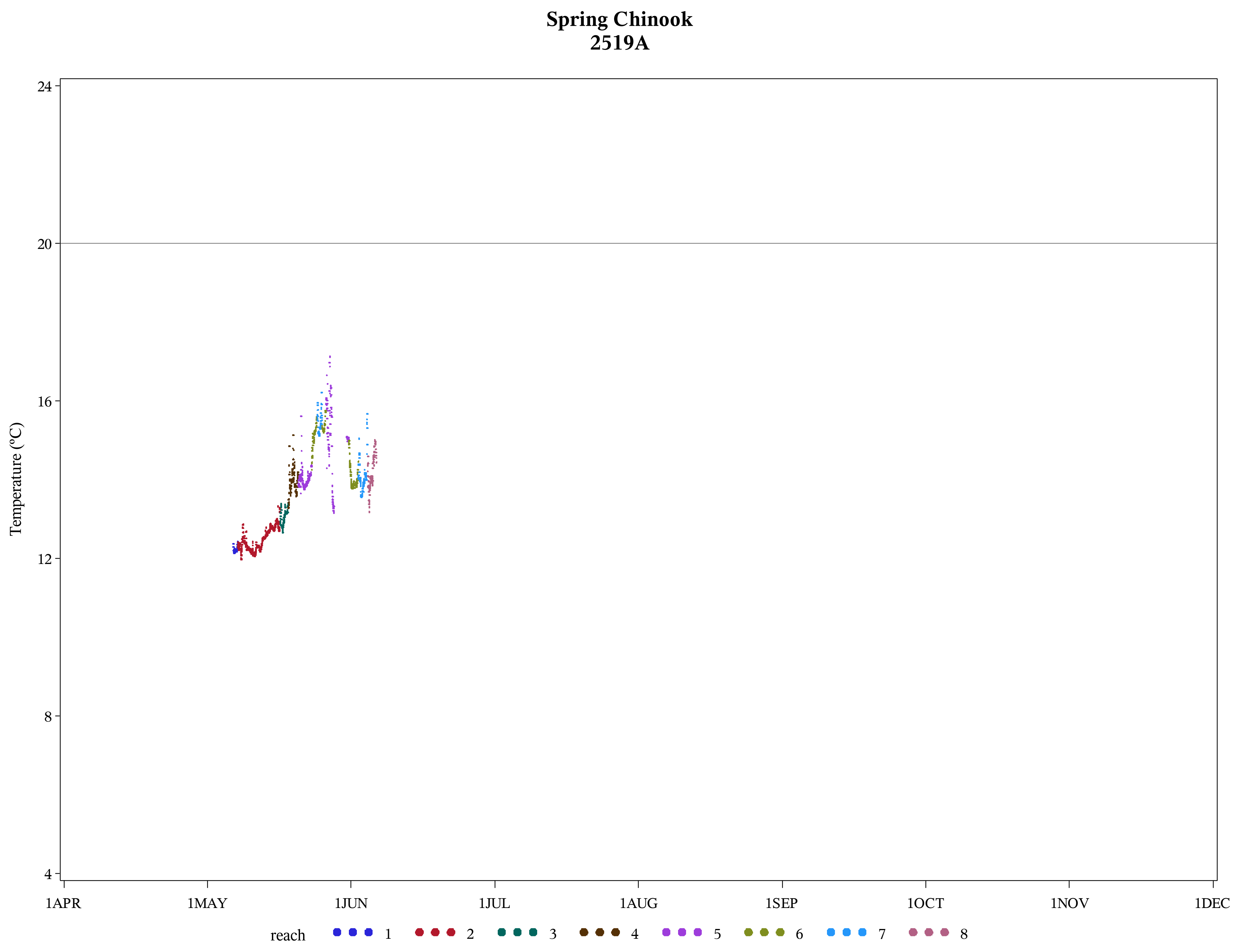

Spring Chinook
2519A
Temperature (°C)
24
20
16
12
8
4
1APR
1MAY
1JUN
1JUL
1AUG
1SEP
1OCT
1NOV
1DEC
reach
1
2
3
4
5
6
7
8

Spring Chinook
2520A

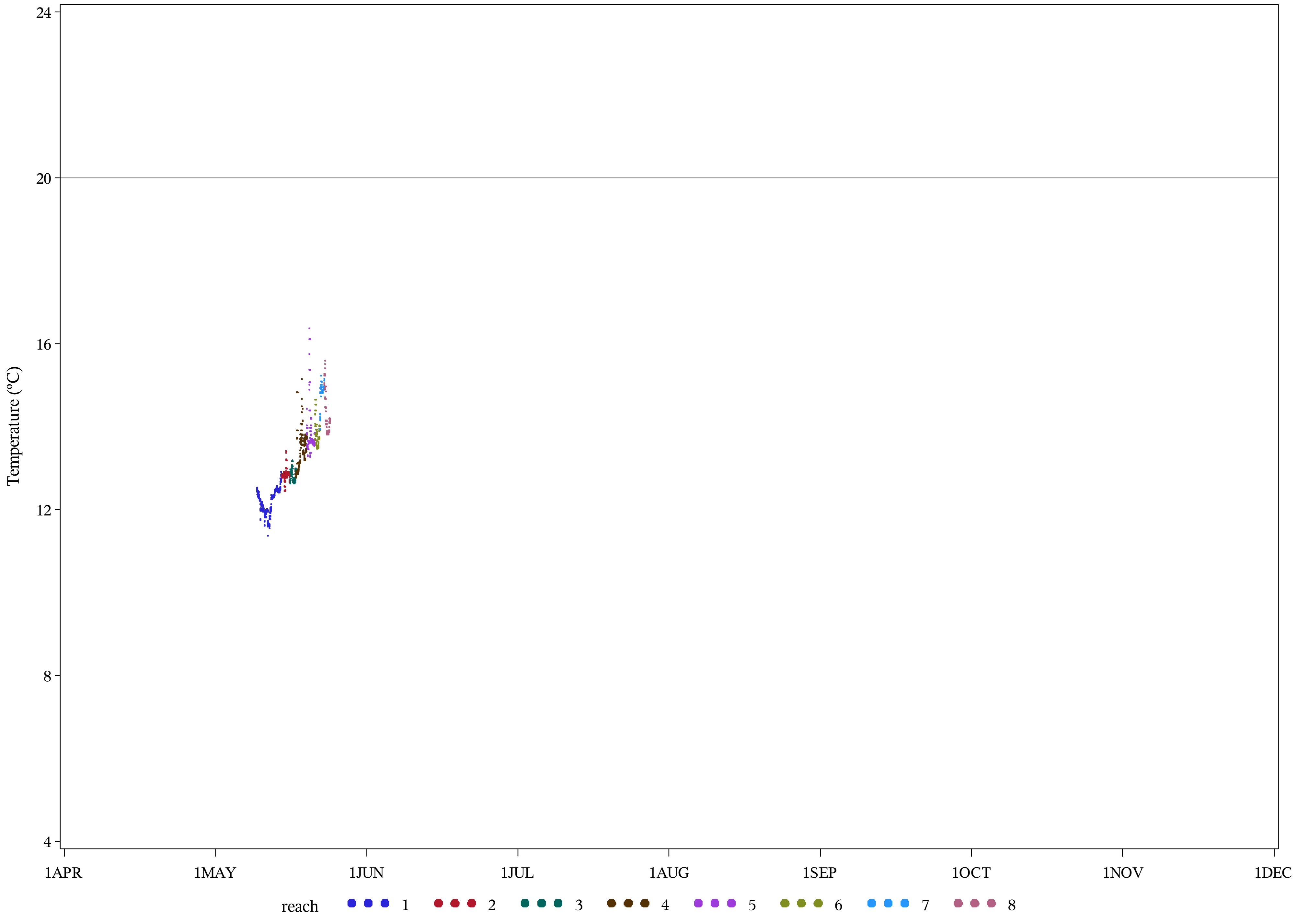

Spring Chinook
2530A

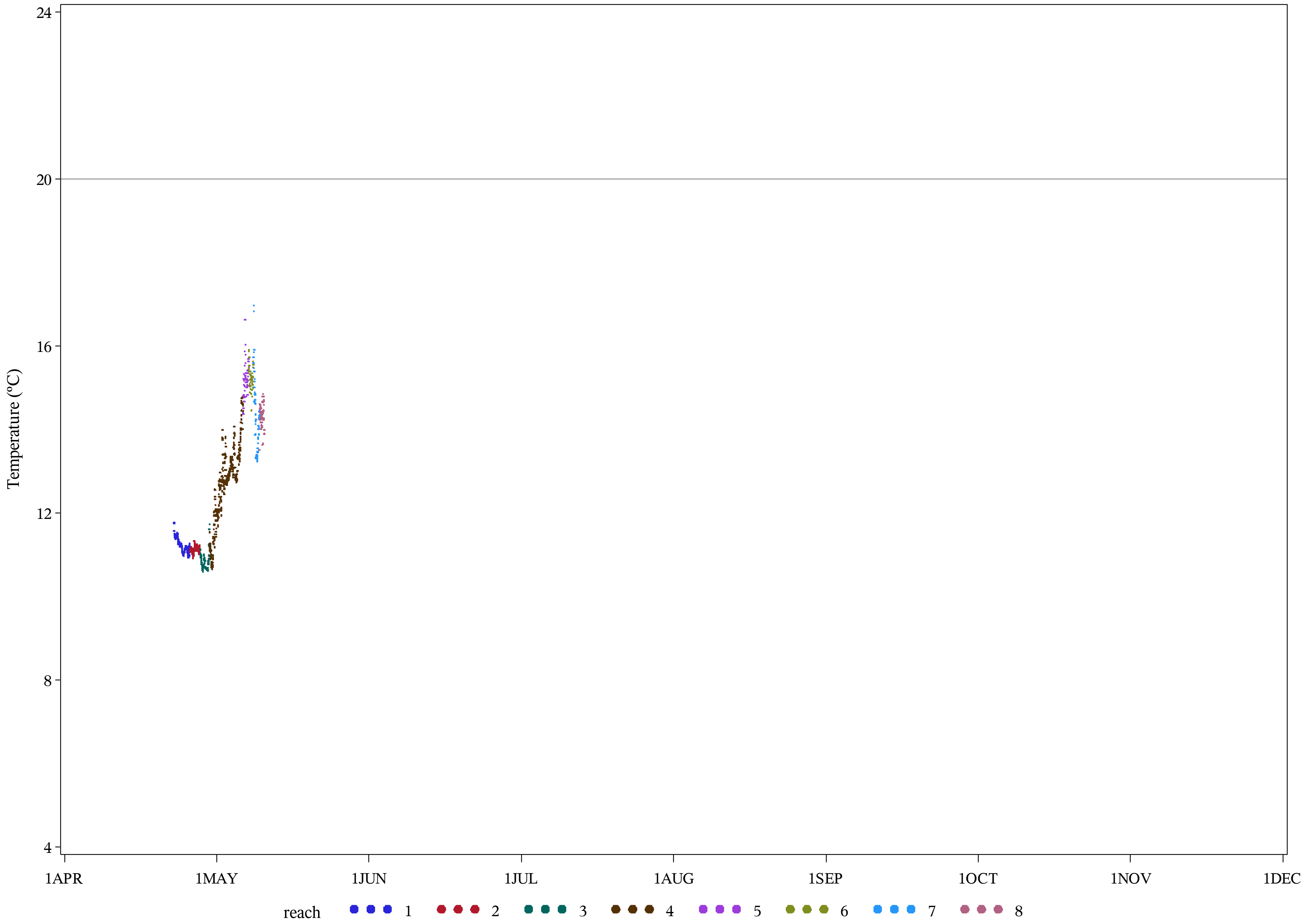

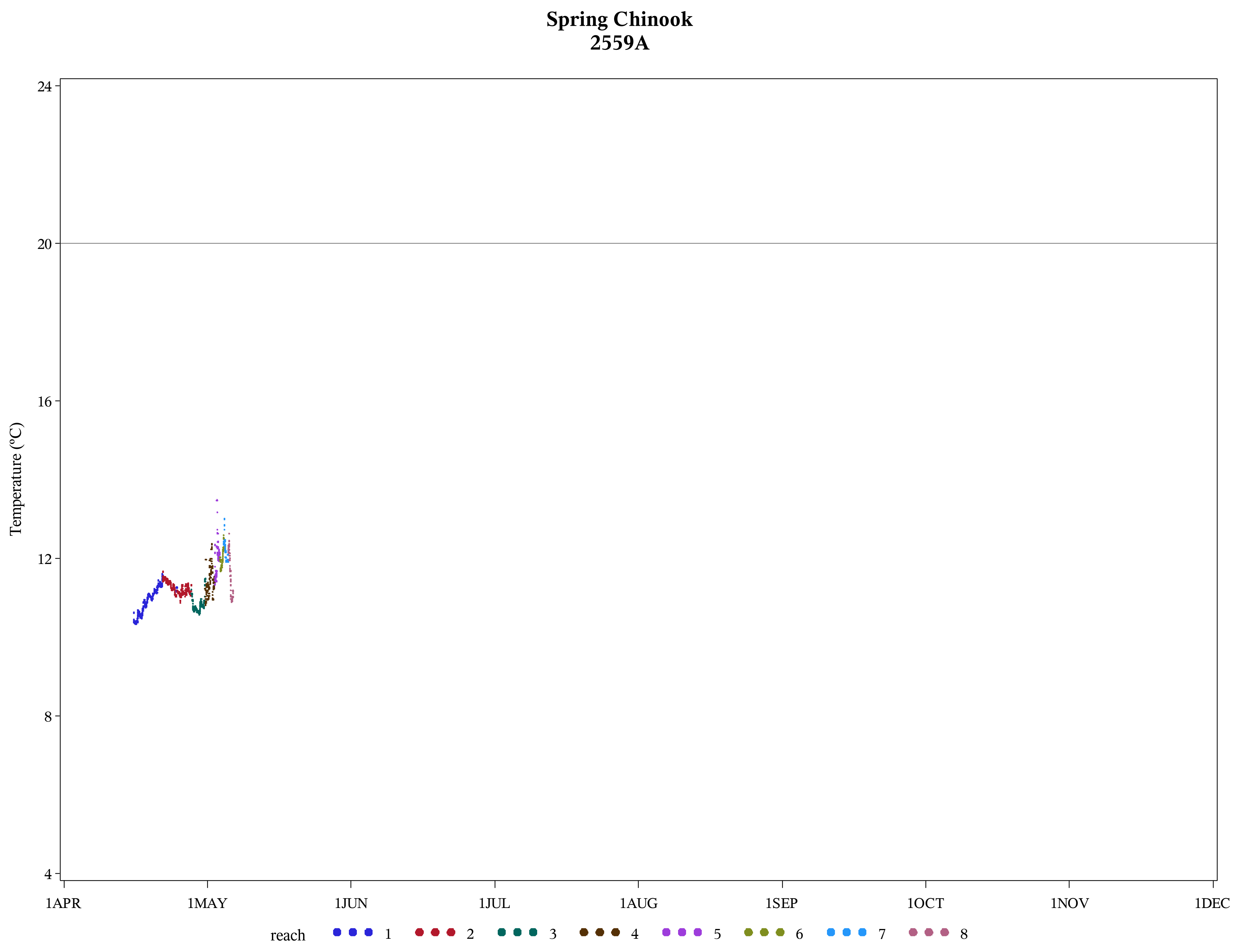

Spring Chinook
2559A
Temperature (°C)
24
20
16
12
8
4
1APR
1MAY
1JUN
1JUL
1AUG
1SEP
1OCT
1NOV
1DEC
reach
1
2
3
4
5
6
7
8

Spring Chinook
2570A

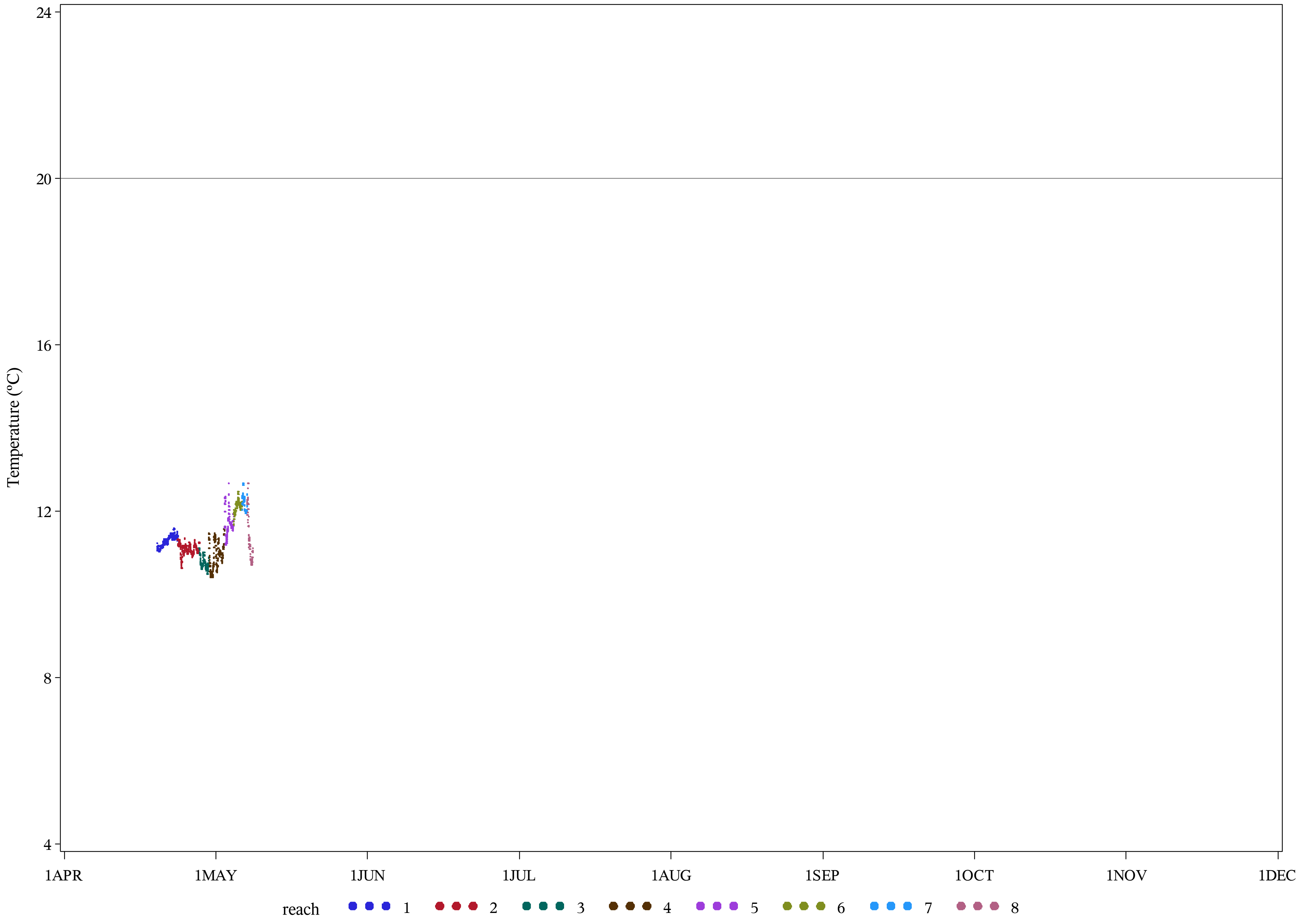

Spring Chinook
2577A

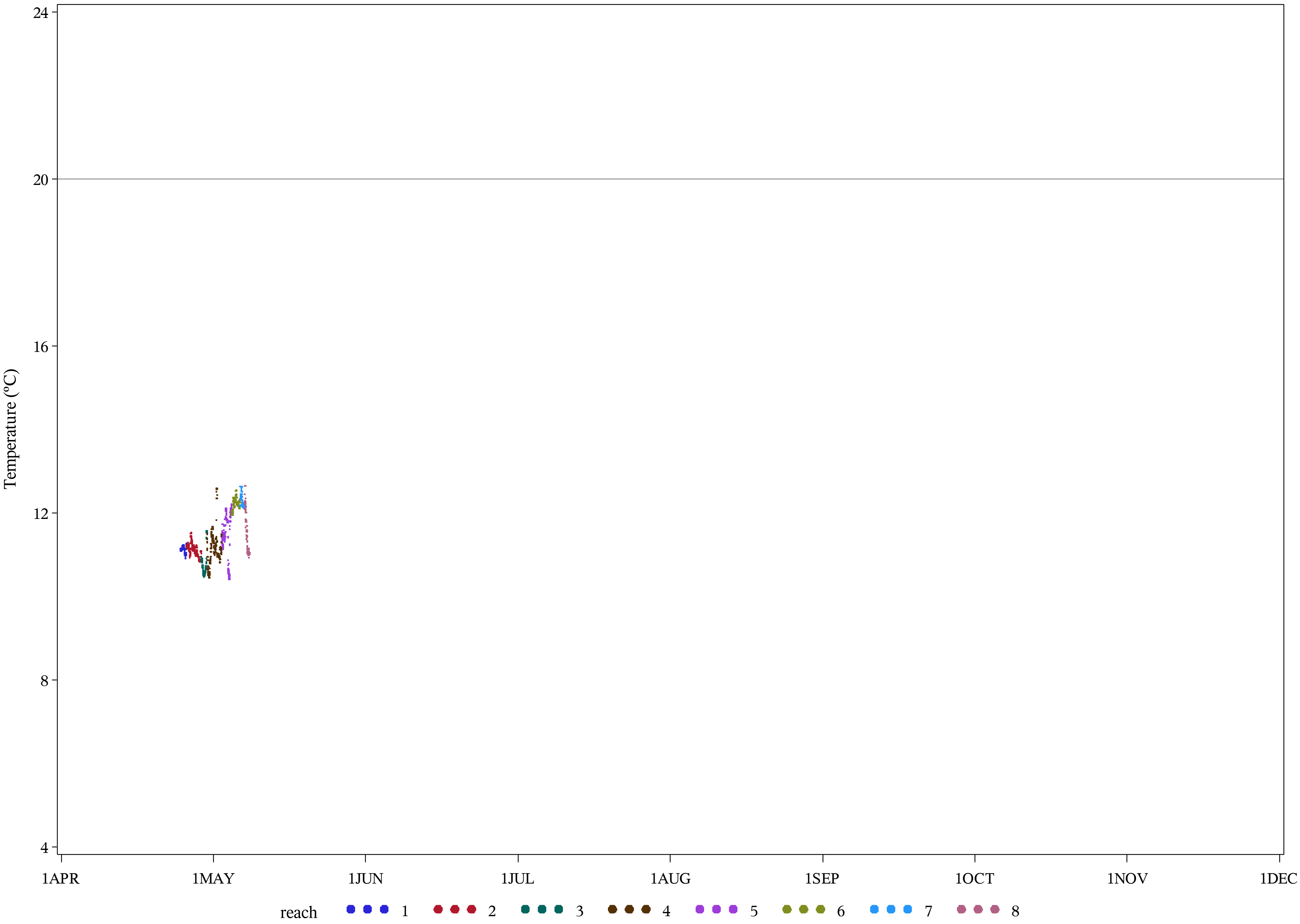

Spring Chinook
2577B

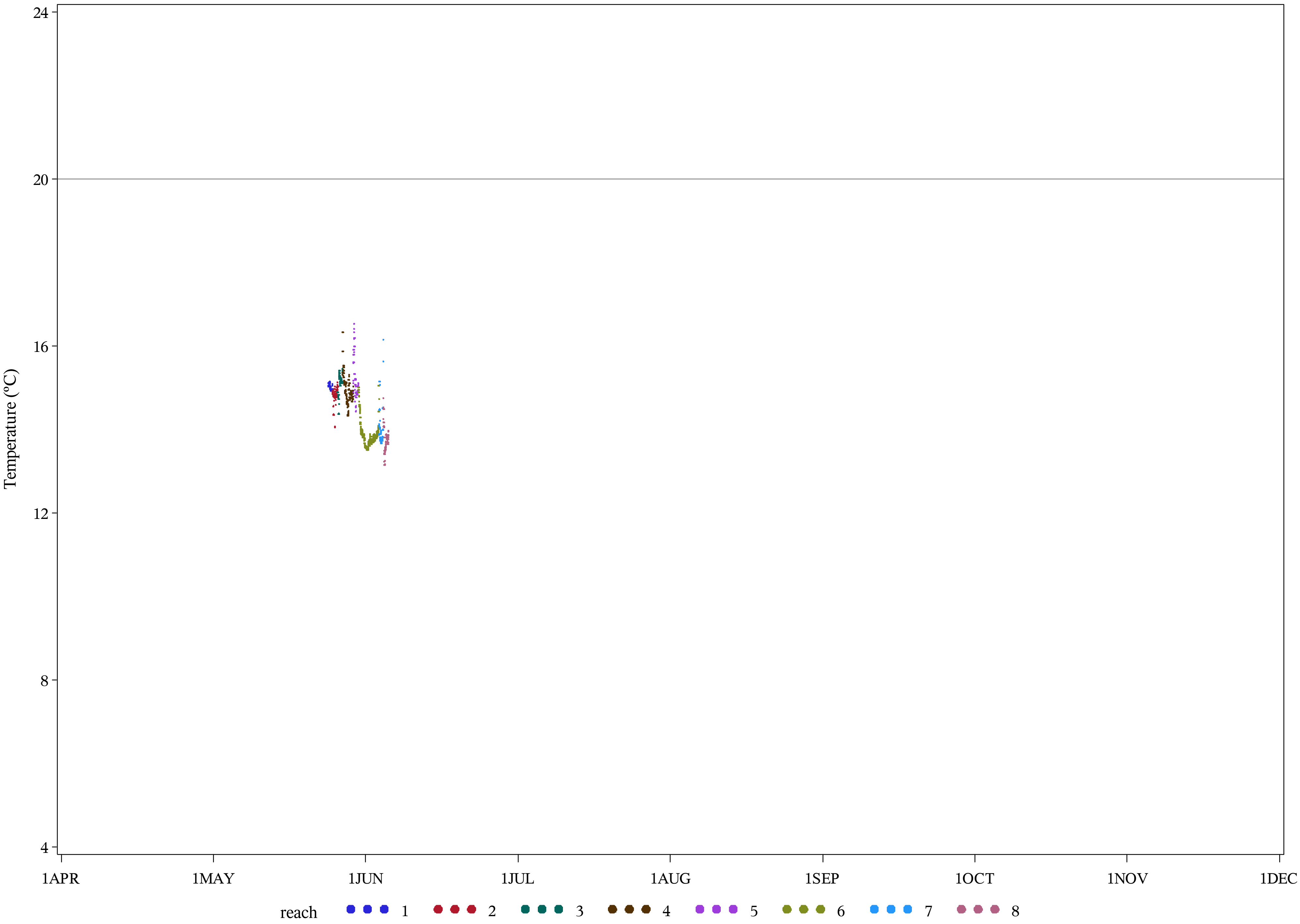

Spring Chinook
2592B

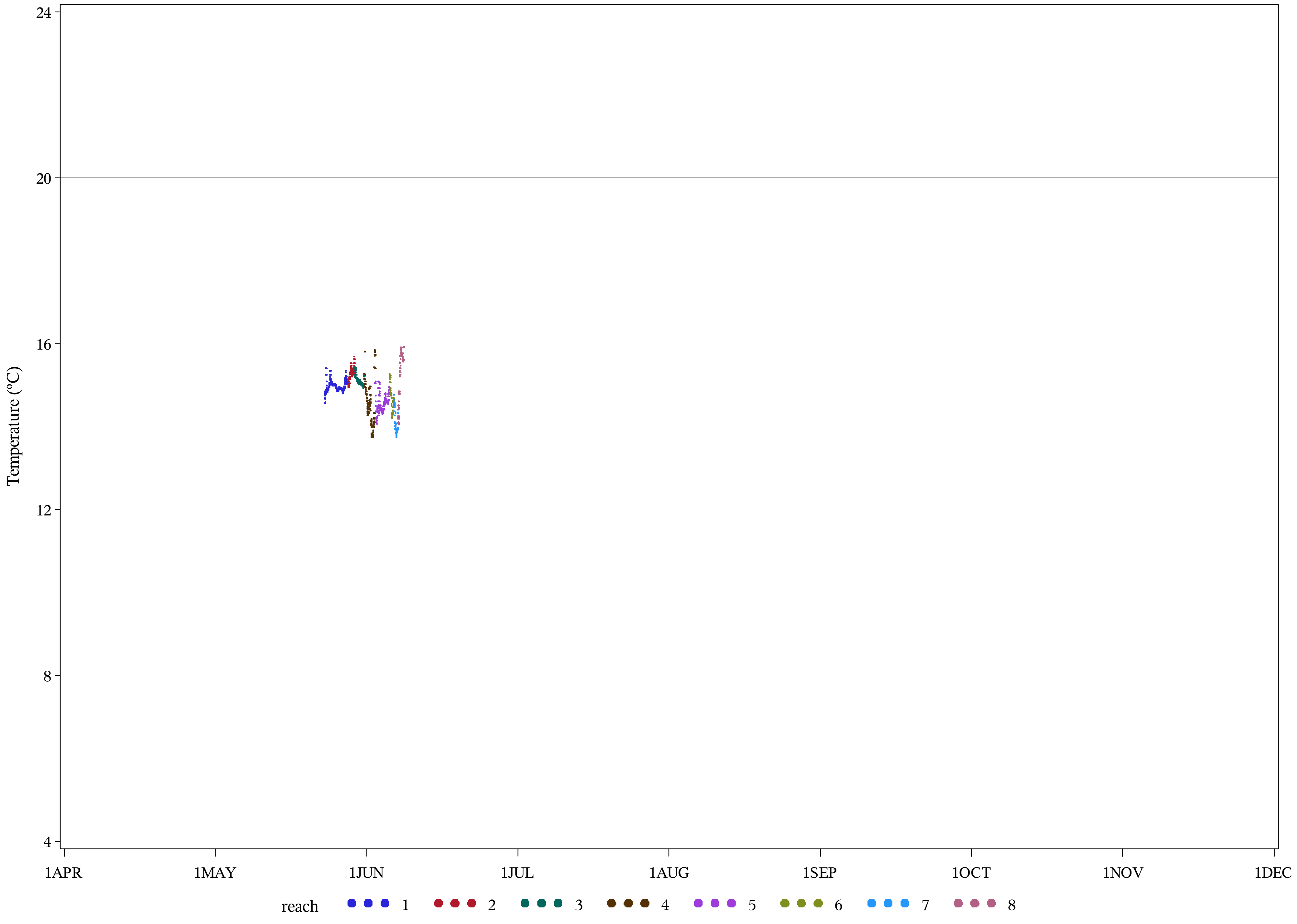

Spring Chinook
2594A

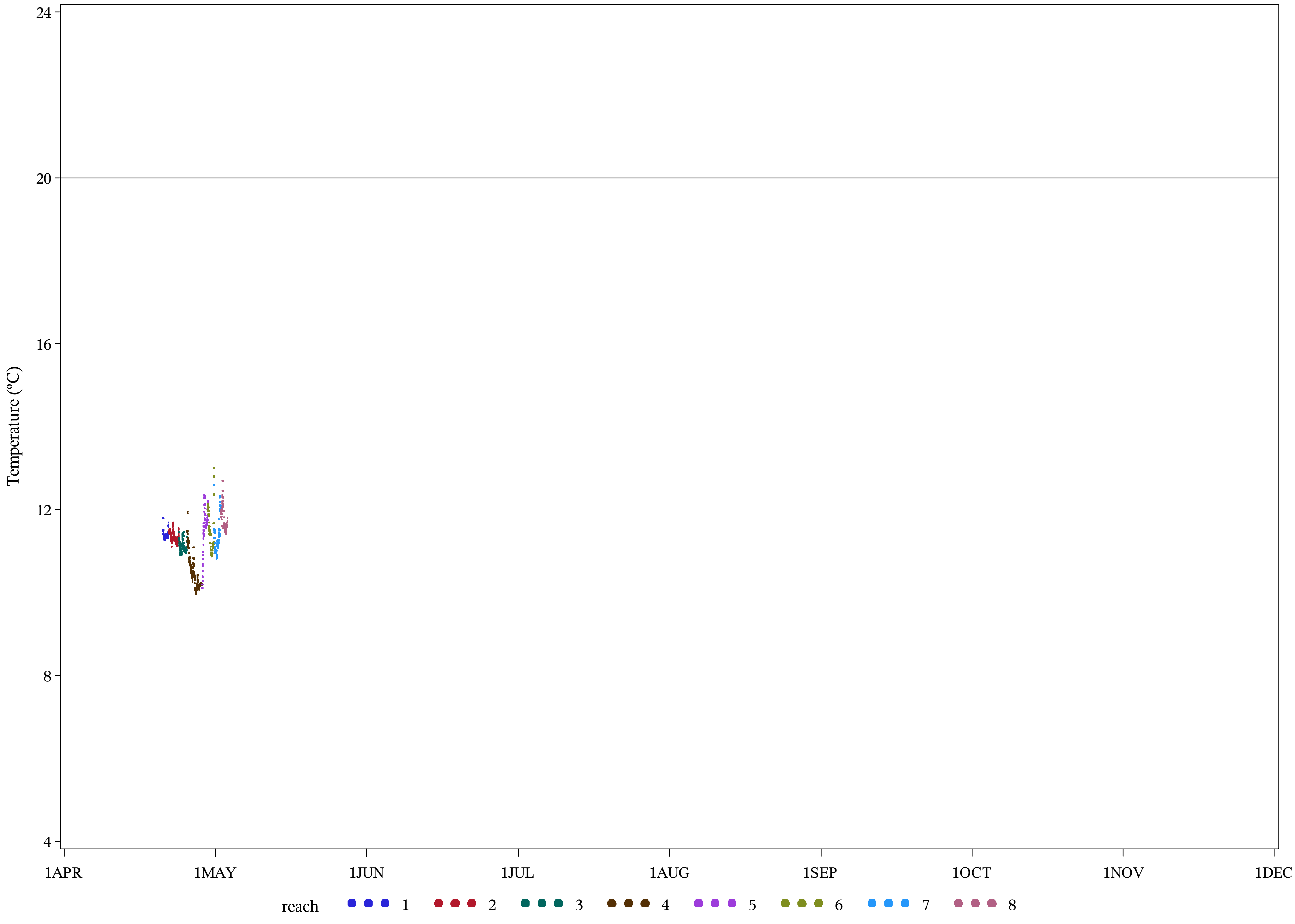

Spring Chinook
2594B

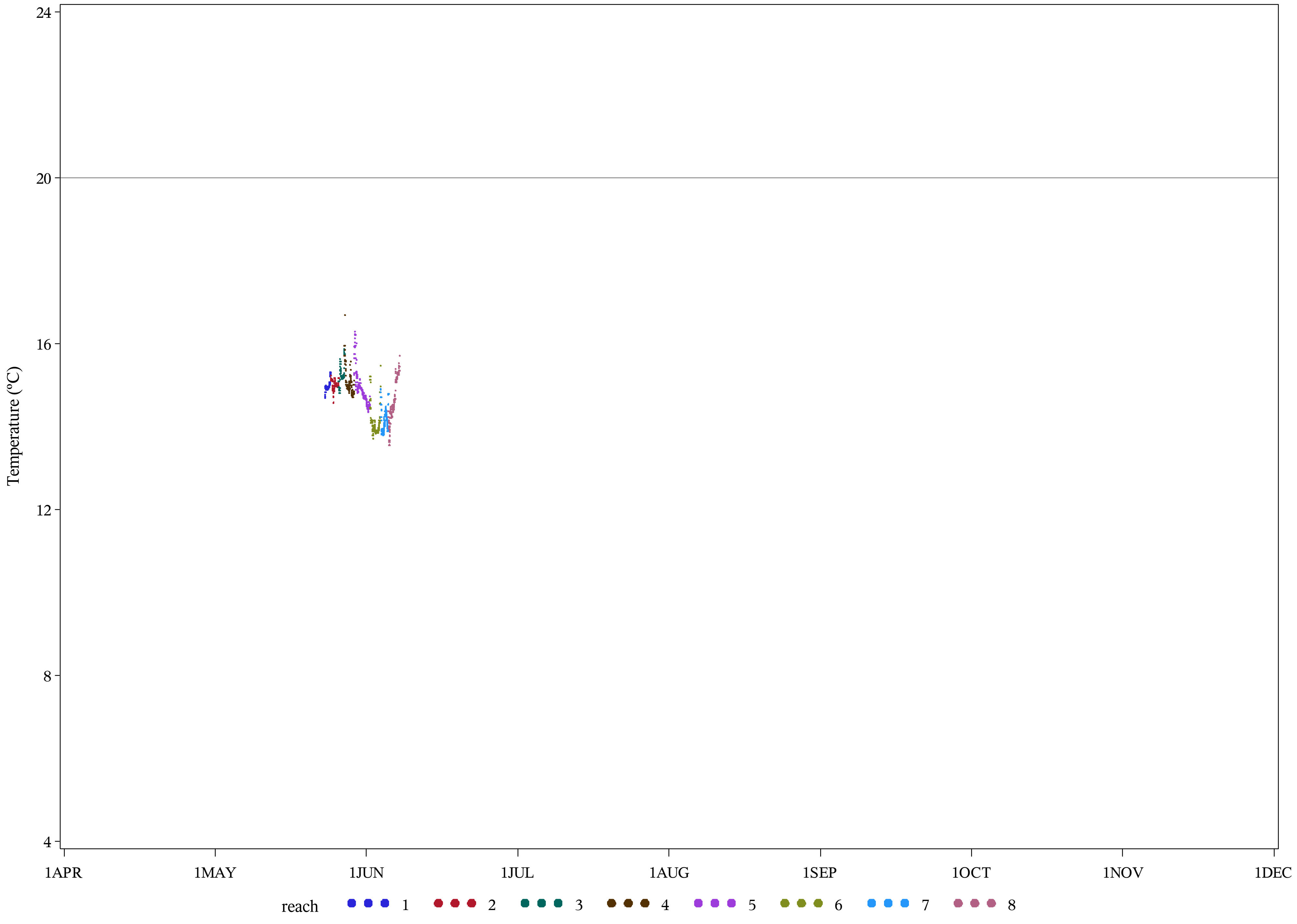

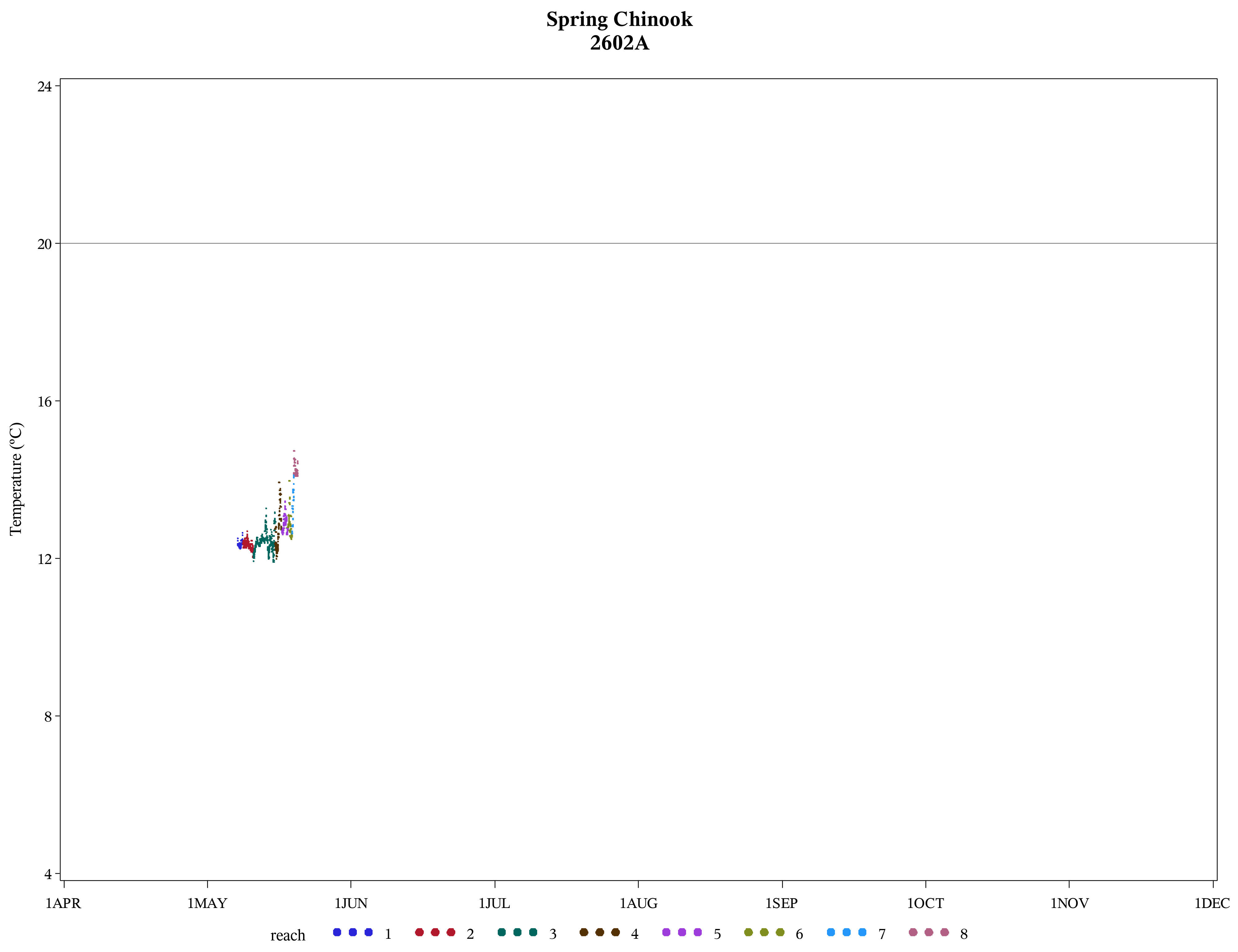
Spring Chinook
2602A
Temperature (°C)
24
20
16
12
8
4
1APR
1MAY
1JUN
1JUL
1AUG
1SEP
1OCT
1NOV
1DEC
reach
1
2
3
4
5
6
7
8

Spring Chinook
2619A

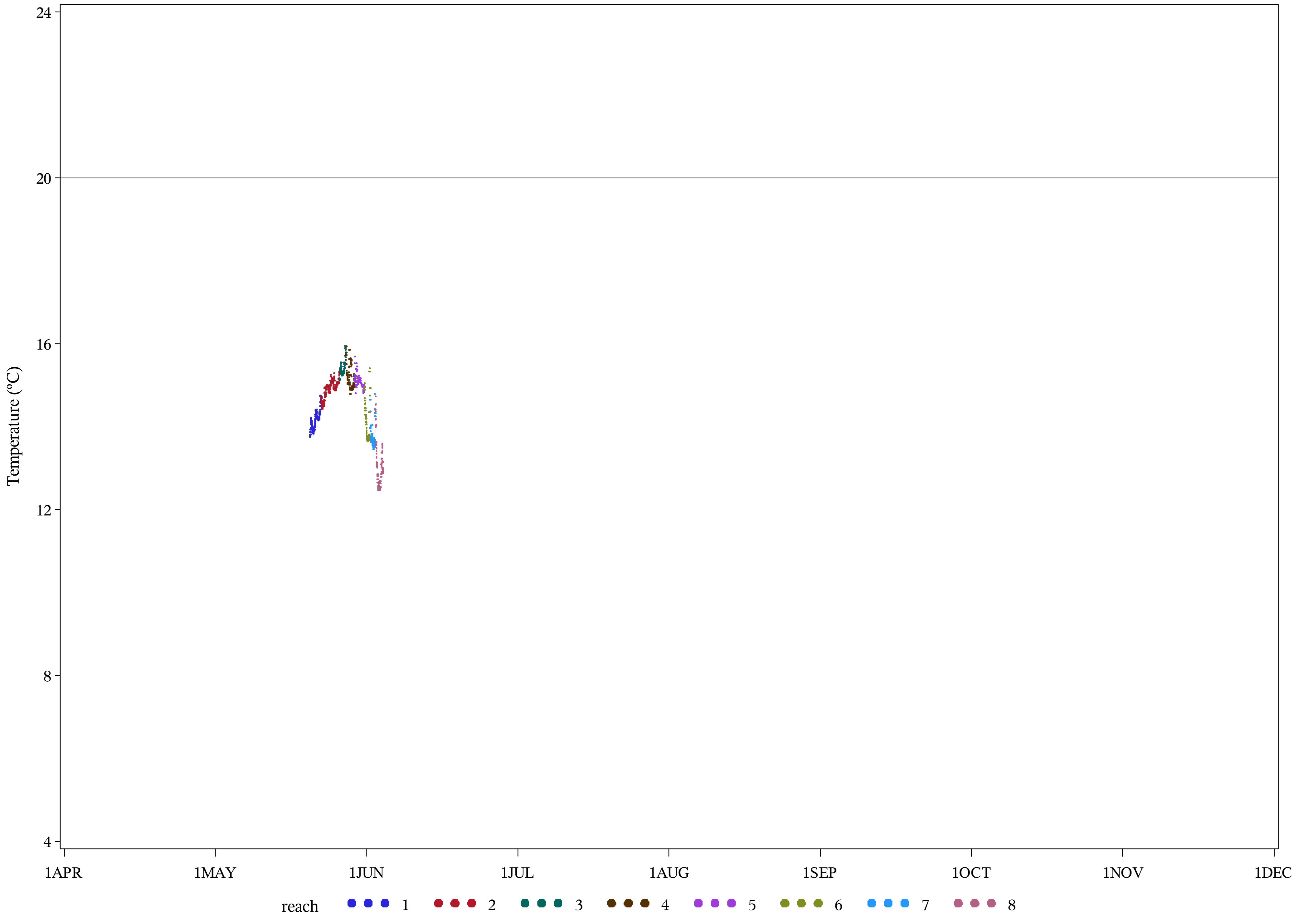

Spring Chinook
2621A

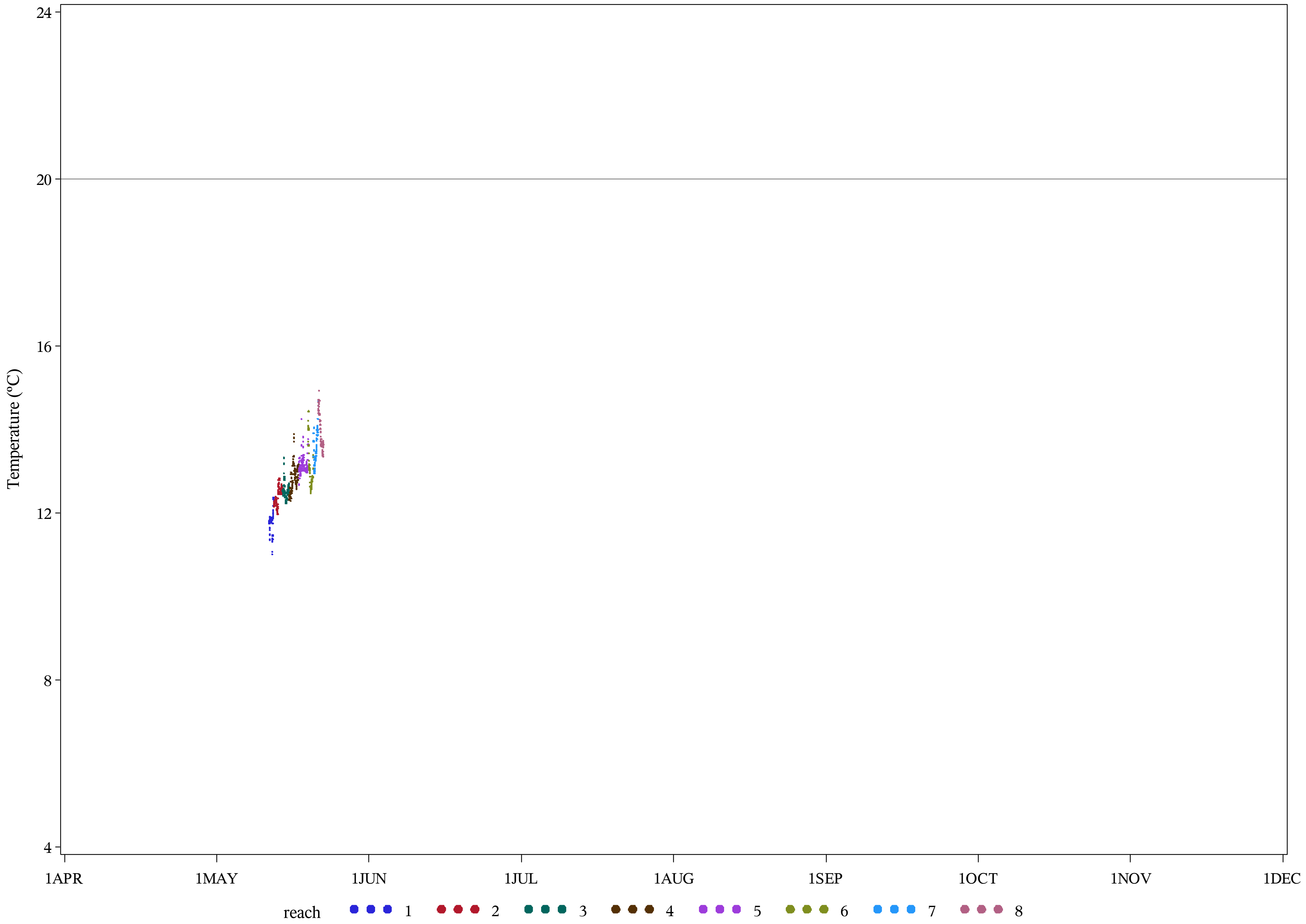

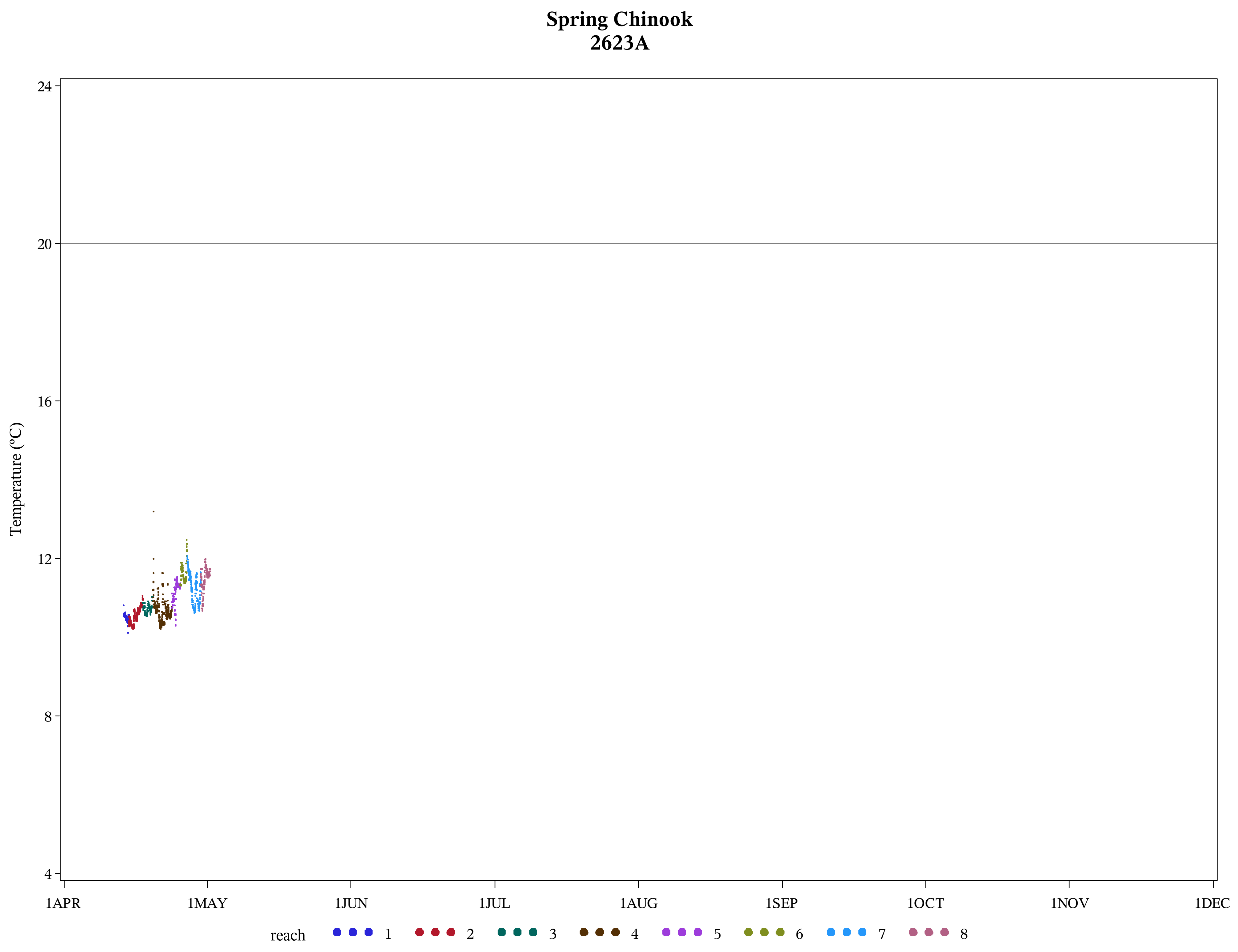

Spring Chinook
2623A
Temperature (°C)
24
20
16
12
8
4
1APR
1MAY
1JUN
1JUL
1AUG
1SEP
1OCT
1NOV
1DEC
reach
1
2
3
4
5
6
7
8

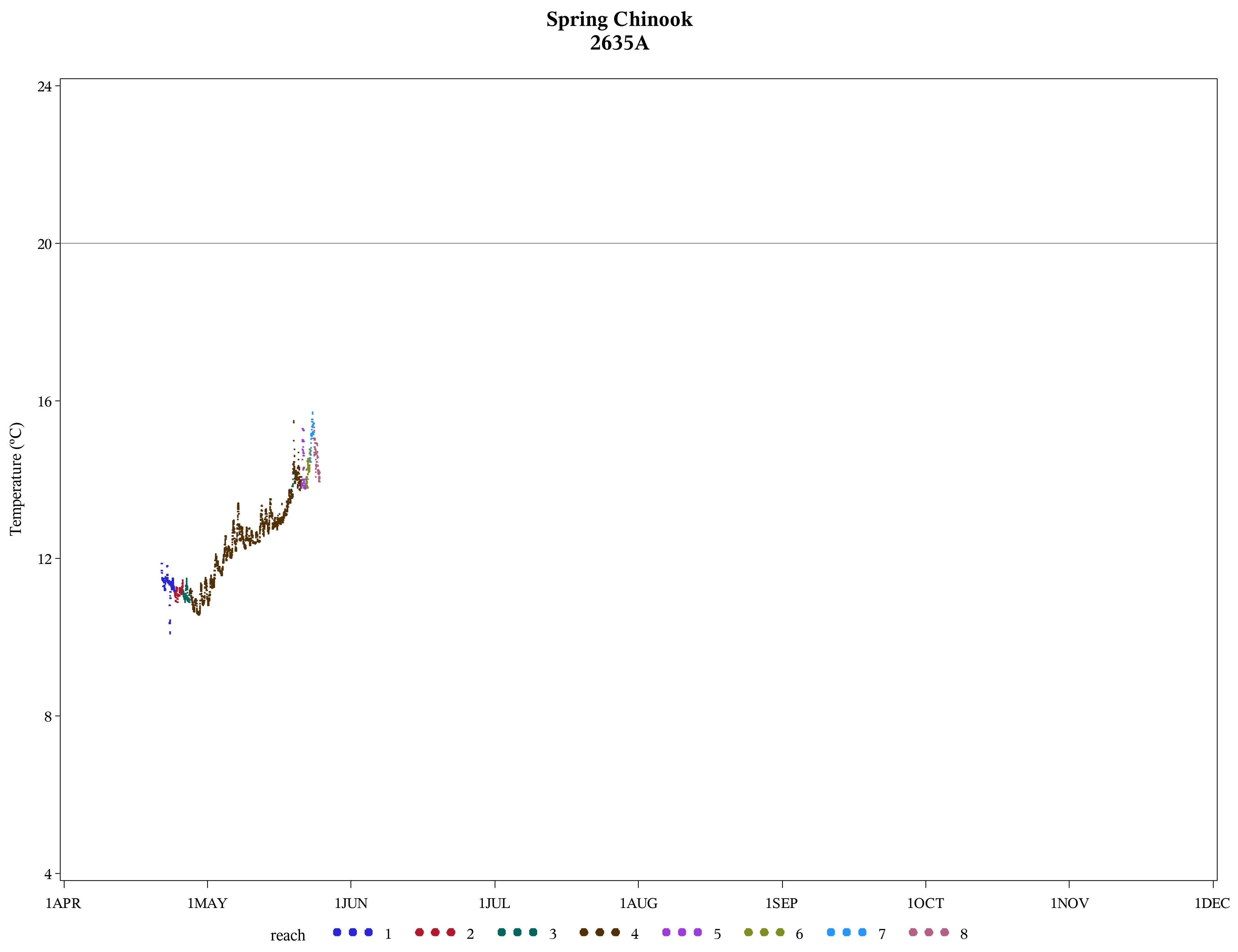

Spring Chinook
2635A
Temperature (°C)
24
20
16
12
8
4
1APR
1MAY
1JUN
1JUL
1AUG
1SEP
1OCT
1NOV
1DEC
reach
1
2
3
4
5
6
7
8

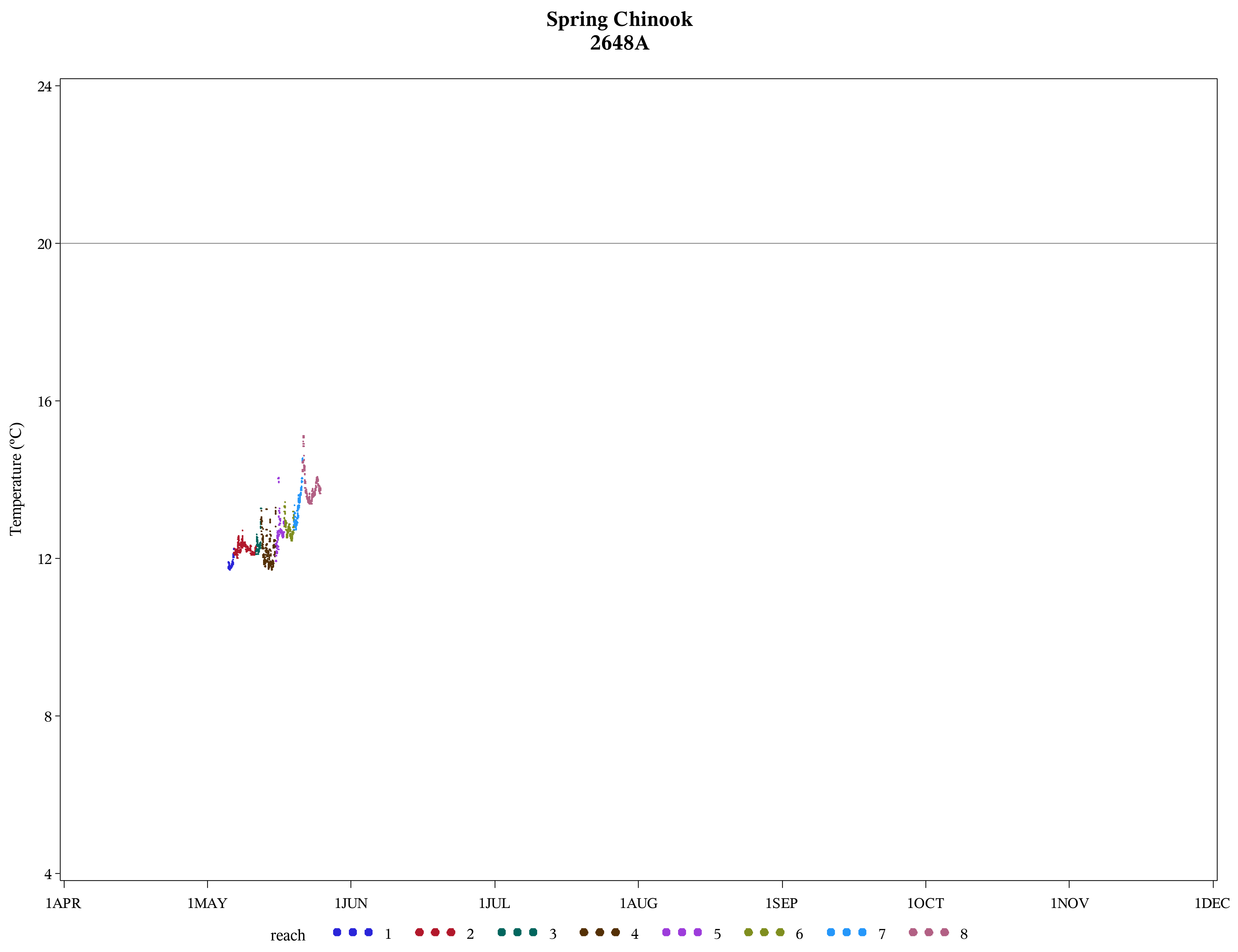

Spring Chinook
2648A
Temperature (°C)
24
20
16
12
8
4
1APR
1MAY
1JUN
1JUL
1AUG
1SEP
1OCT
1NOV
1DEC
reach
1
2
3
4
5
6
7
8

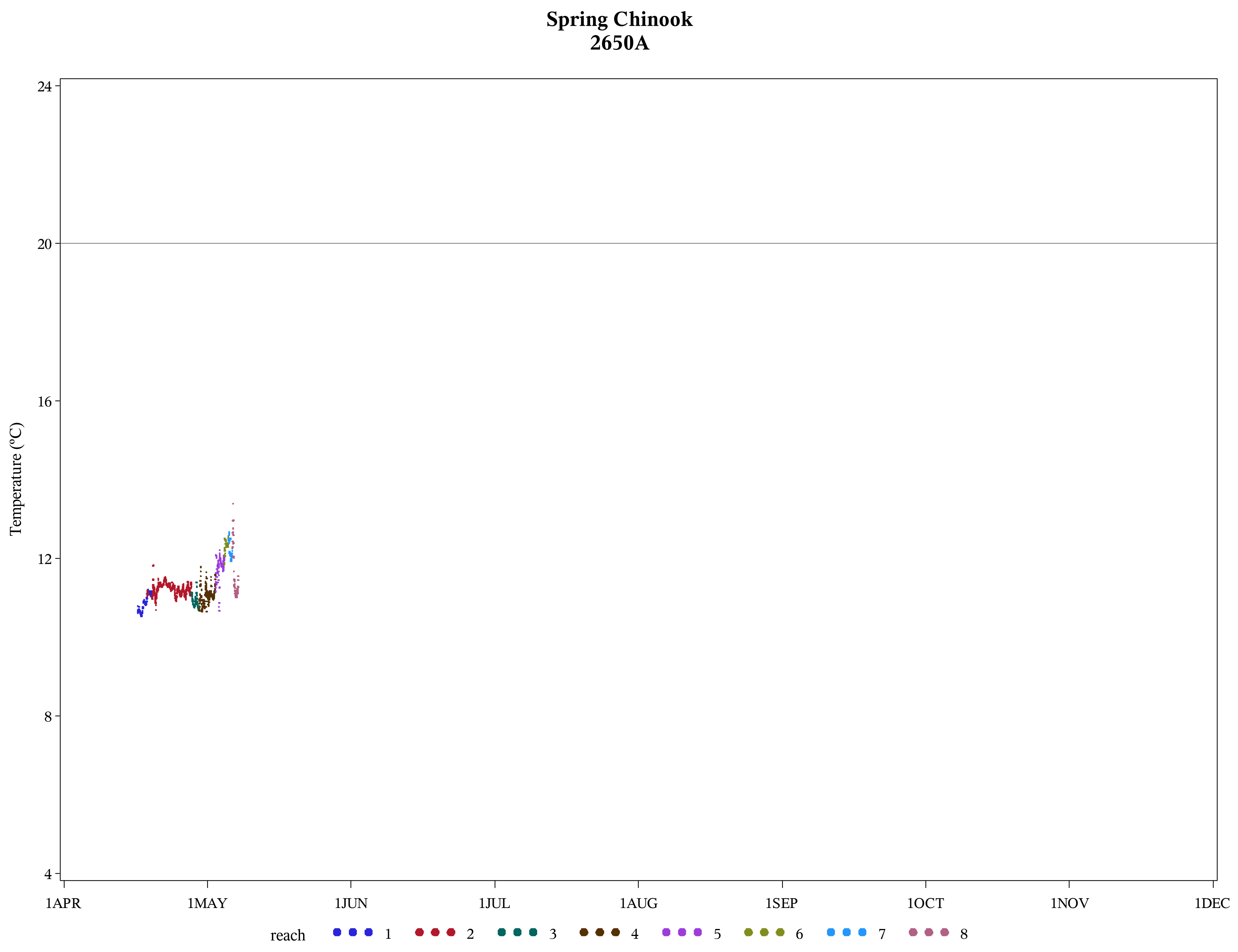
Spring Chinook
2650A
Temperature (°C)
24
20
16
12
8
4
1APR
1MAY
1JUN
1JUL
1AUG
1SEP
1OCT
1NOV
1DEC
reach
1
2
3
4
5
6
7
8

Spring Chinook
2666A

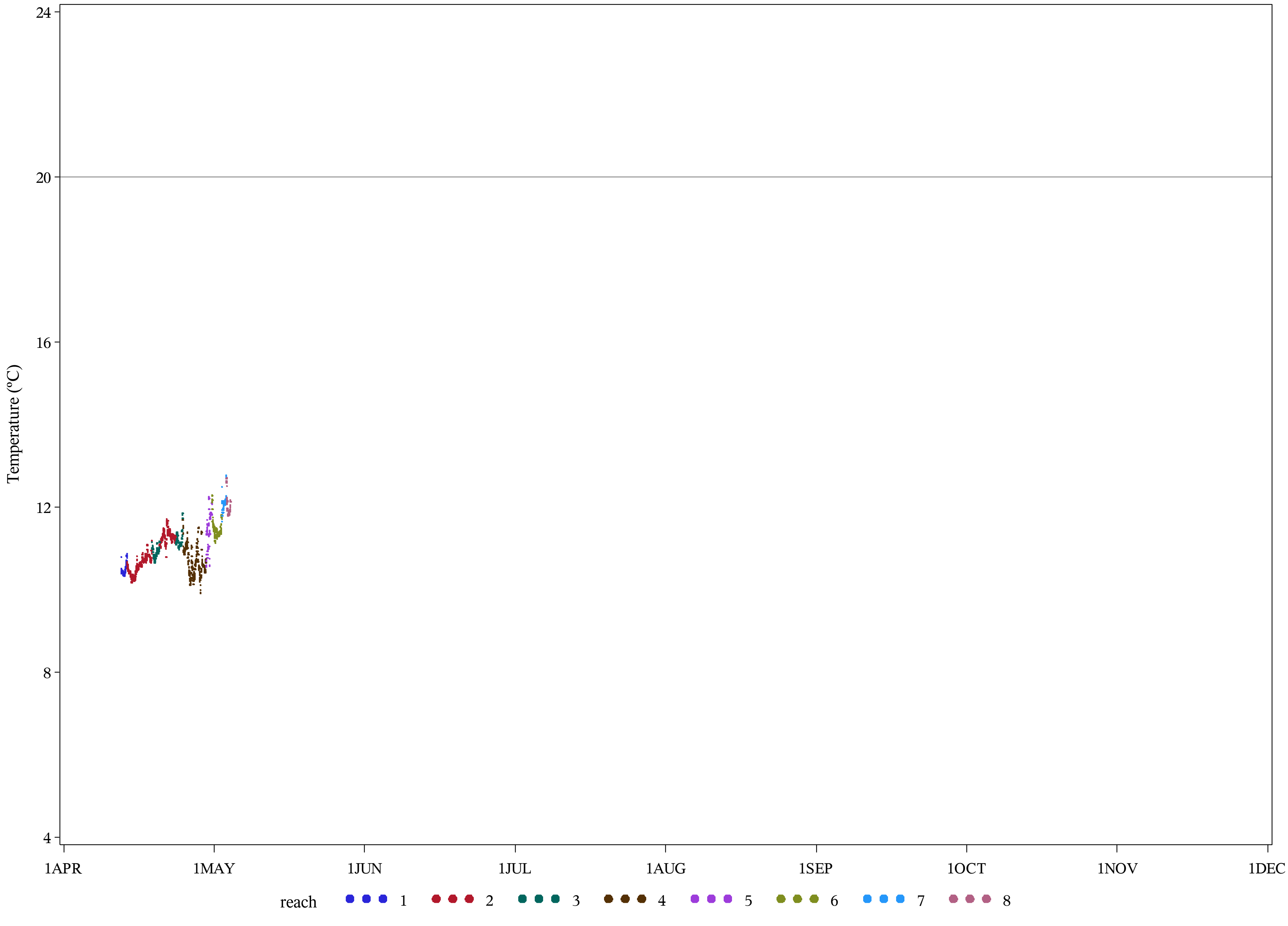

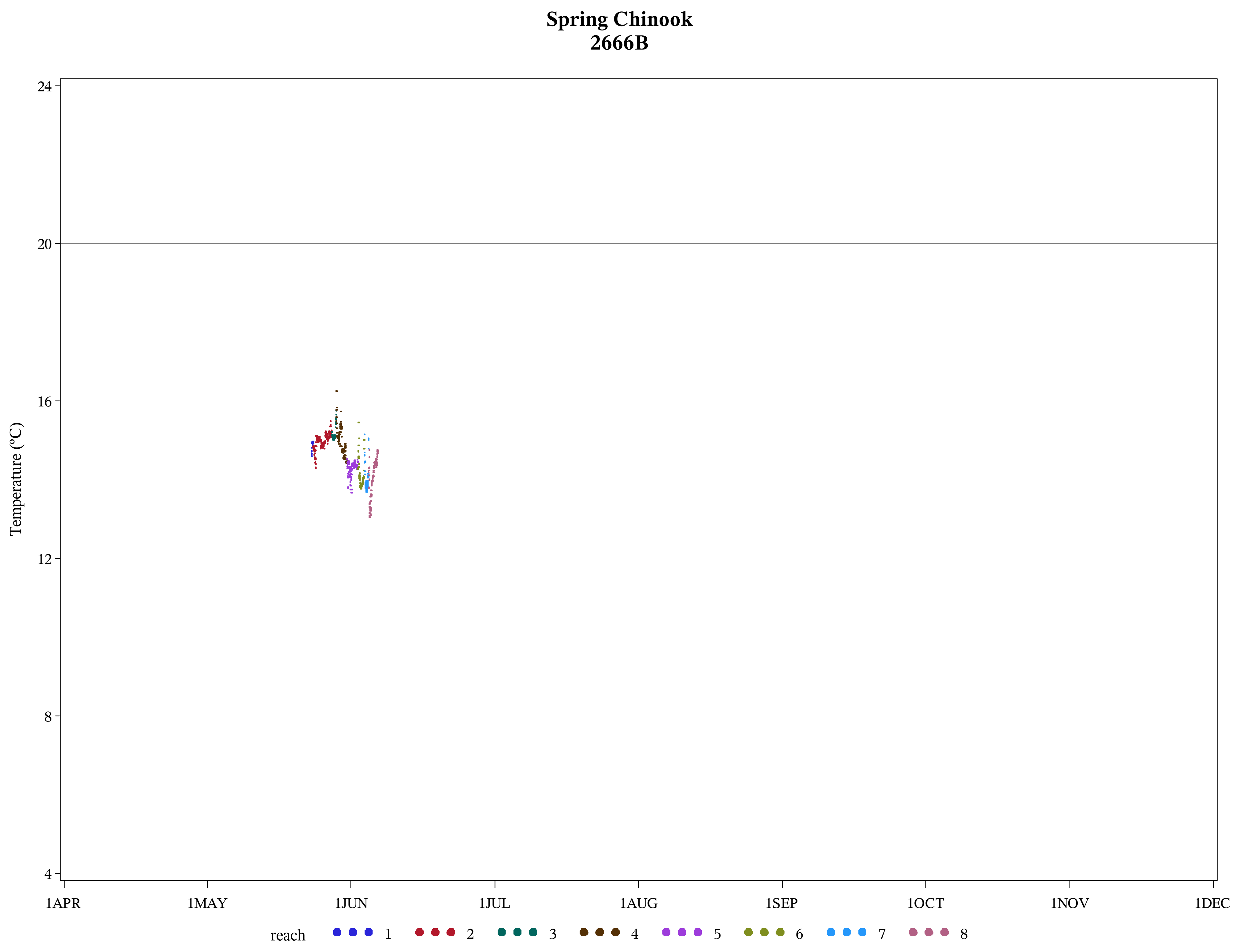

Spring Chinook
2666B
Temperature (°C)
24
20
16
12
8
4
1APR
1MAY
1JUN
1JUL
1AUG
1SEP
1OCT
1NOV
1DEC
reach
1
2
3
4
5
6
7
8

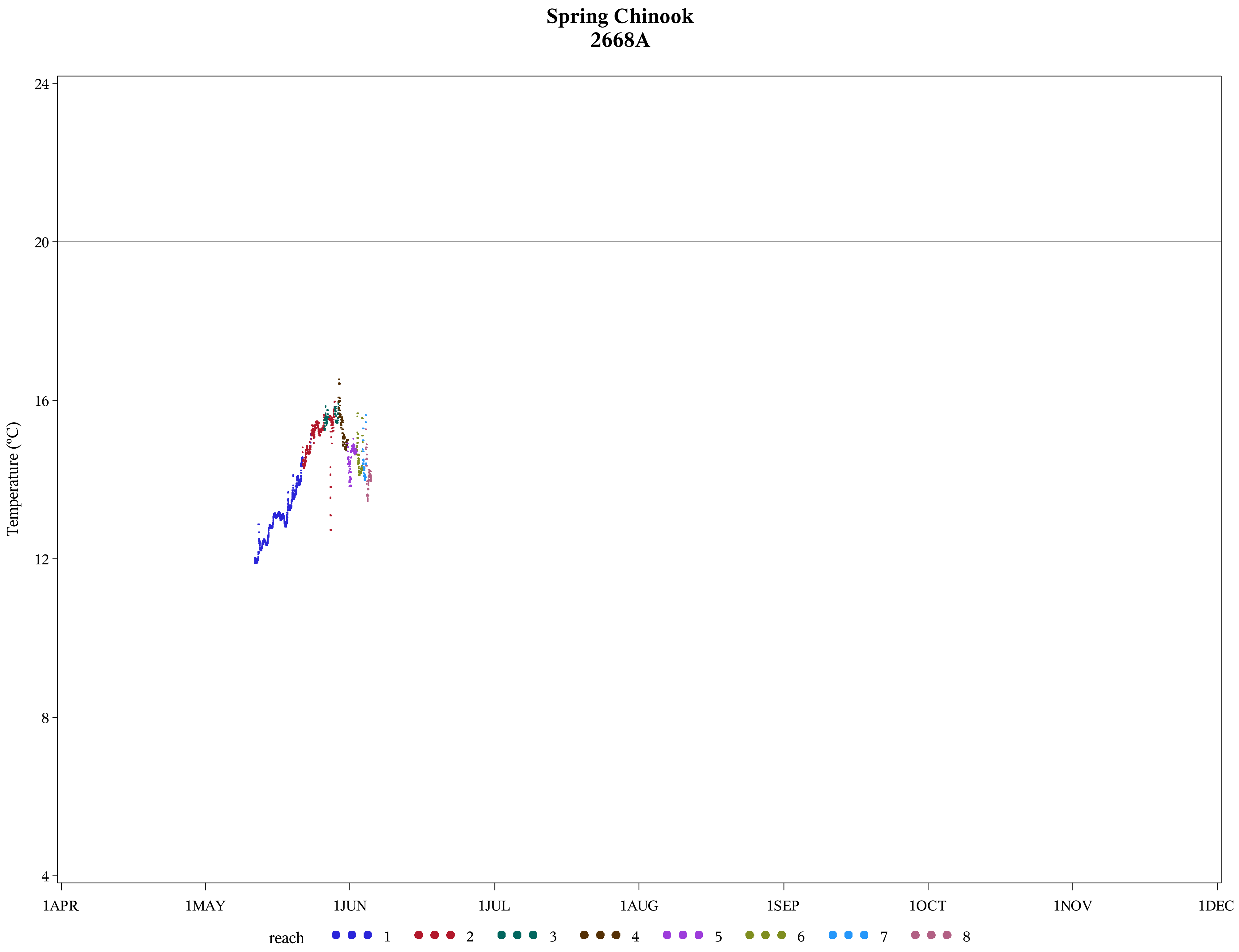
Spring Chinook
2668A
Temperature (°C)
24
20
16
12
8
4
1APR
1MAY
1JUN
1JUL
1AUG
1SEP
1OCT
1NOV
1DEC
reach
1
2
3
4
5
6
7
8

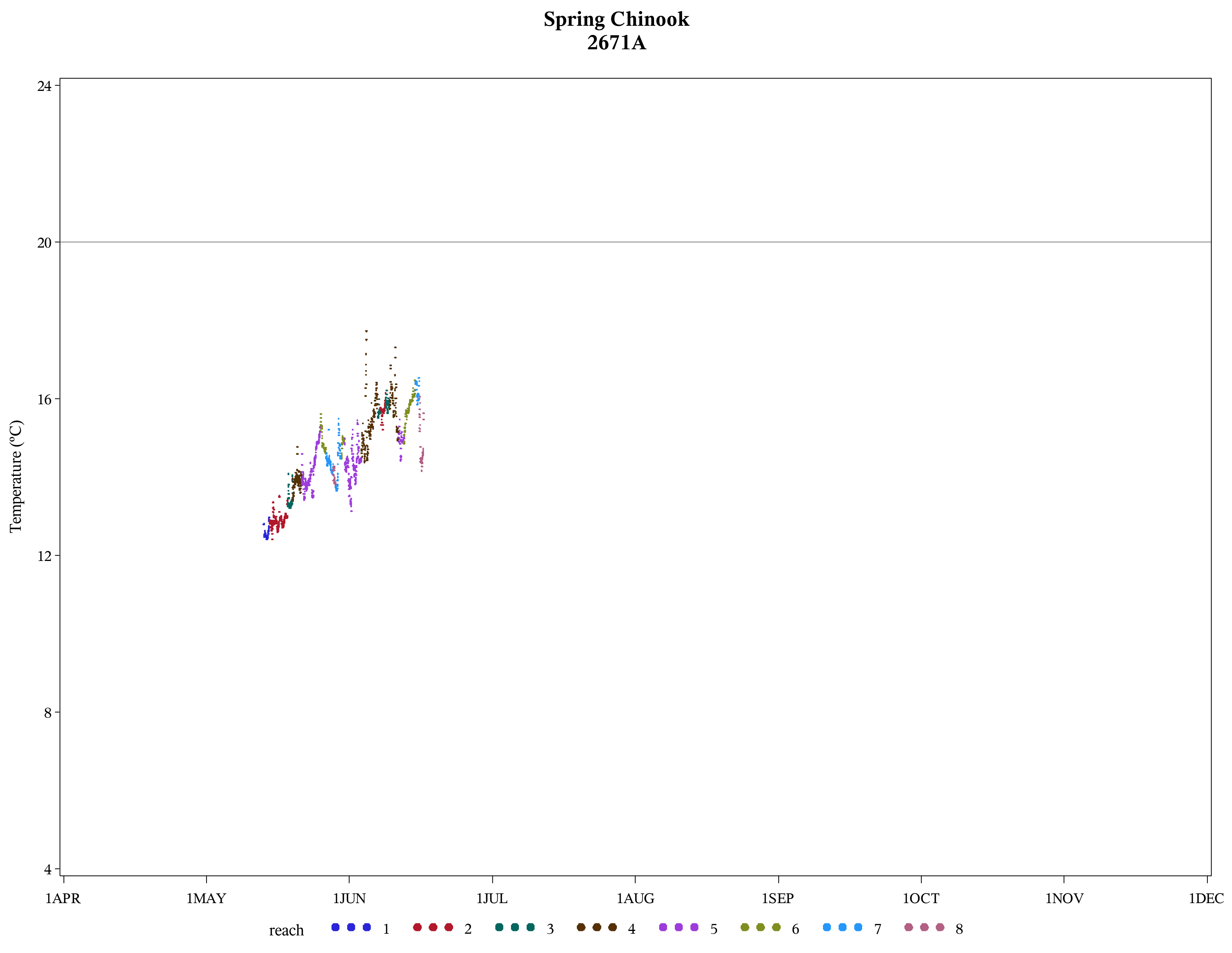

Spring Chinook
2671A
Temperature (°C)
24
20
16
12
8
4
1APR
1MAY
1JUN
1JUL
1AUG
1SEP
1OCT
1NOV
1DEC
reach
1
2
3
4
5
6
7
8

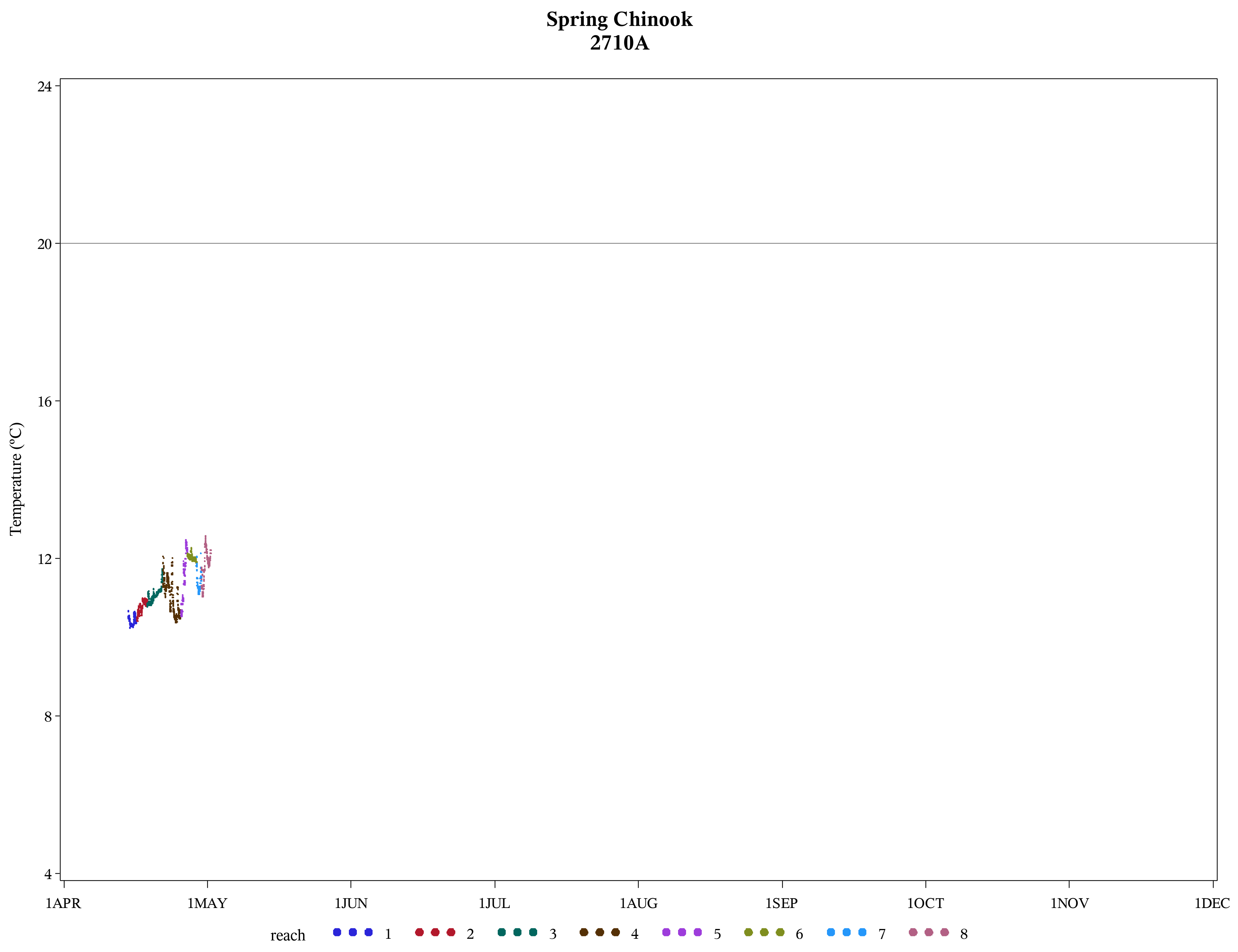

Spring Chinook
2710A
Temperature (°C)
24
20
16
12
8
4
1APR
1MAY
1JUN
1JUL
1AUG
1SEP
1OCT
1NOV
1DEC
reach
1
2
3
4
5
6
7
8

Spring Chinook
2710B

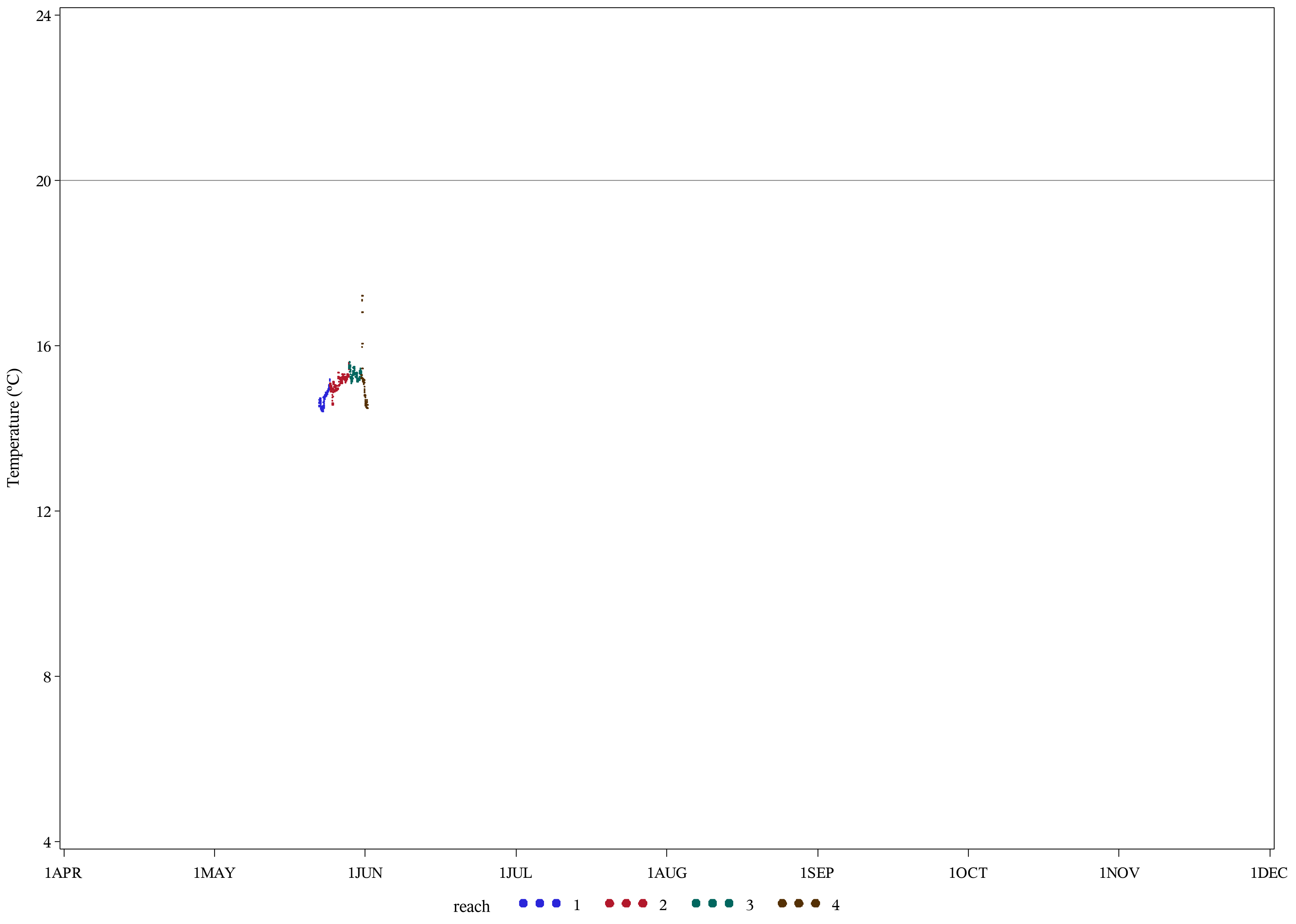

Spring Chinook
2758A

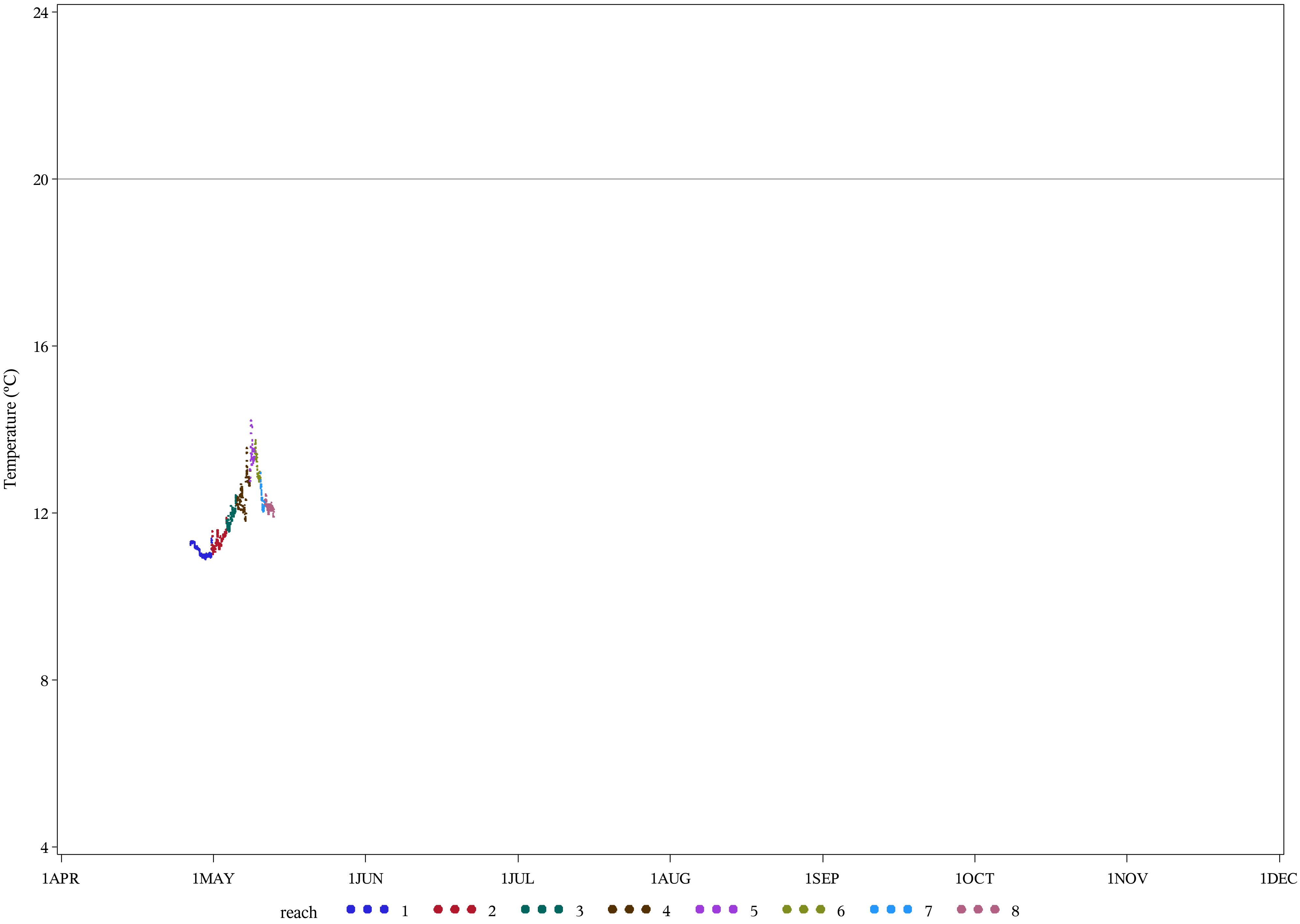

Spring Chinook
2759A

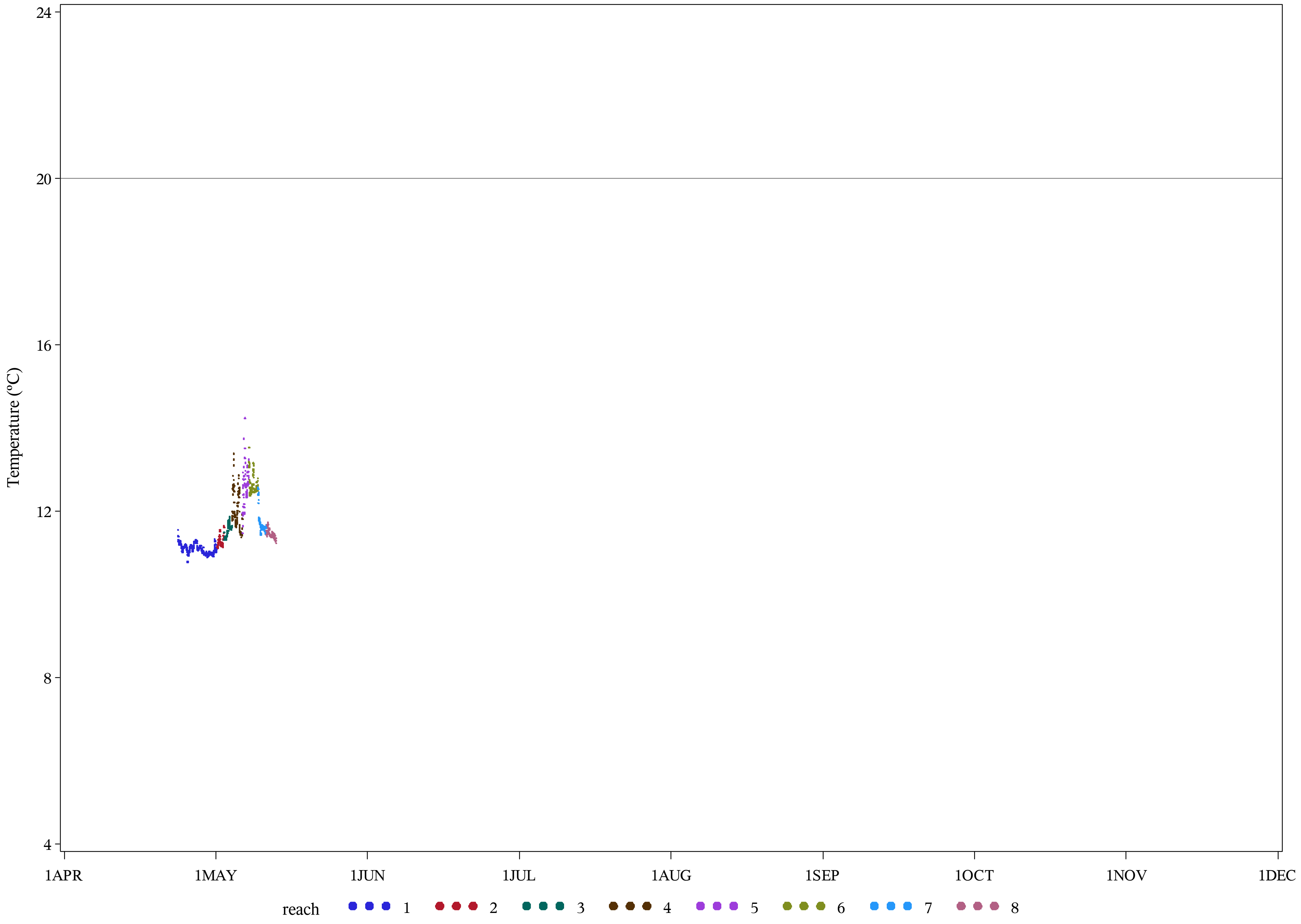

Spring Chinook
2770A

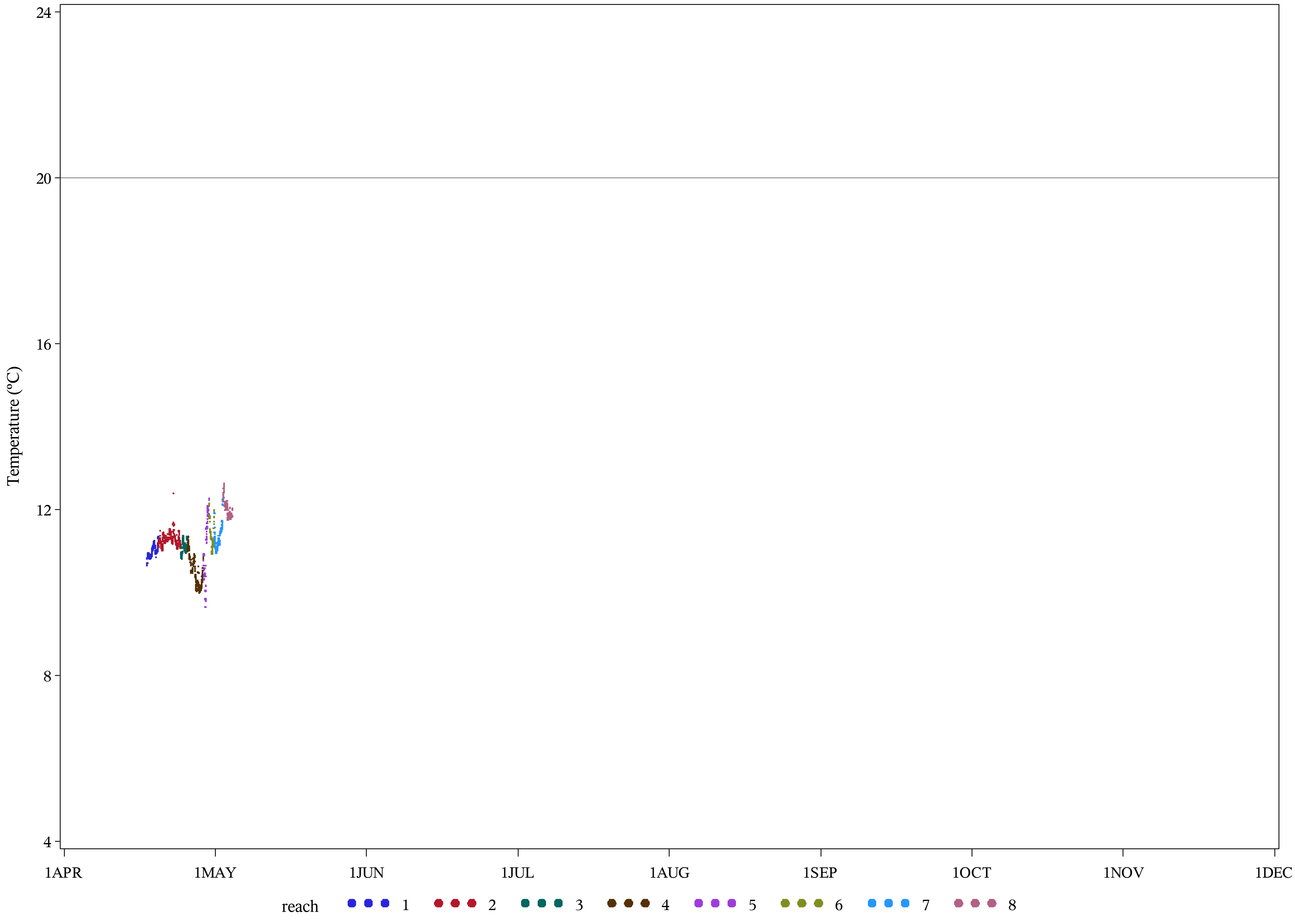

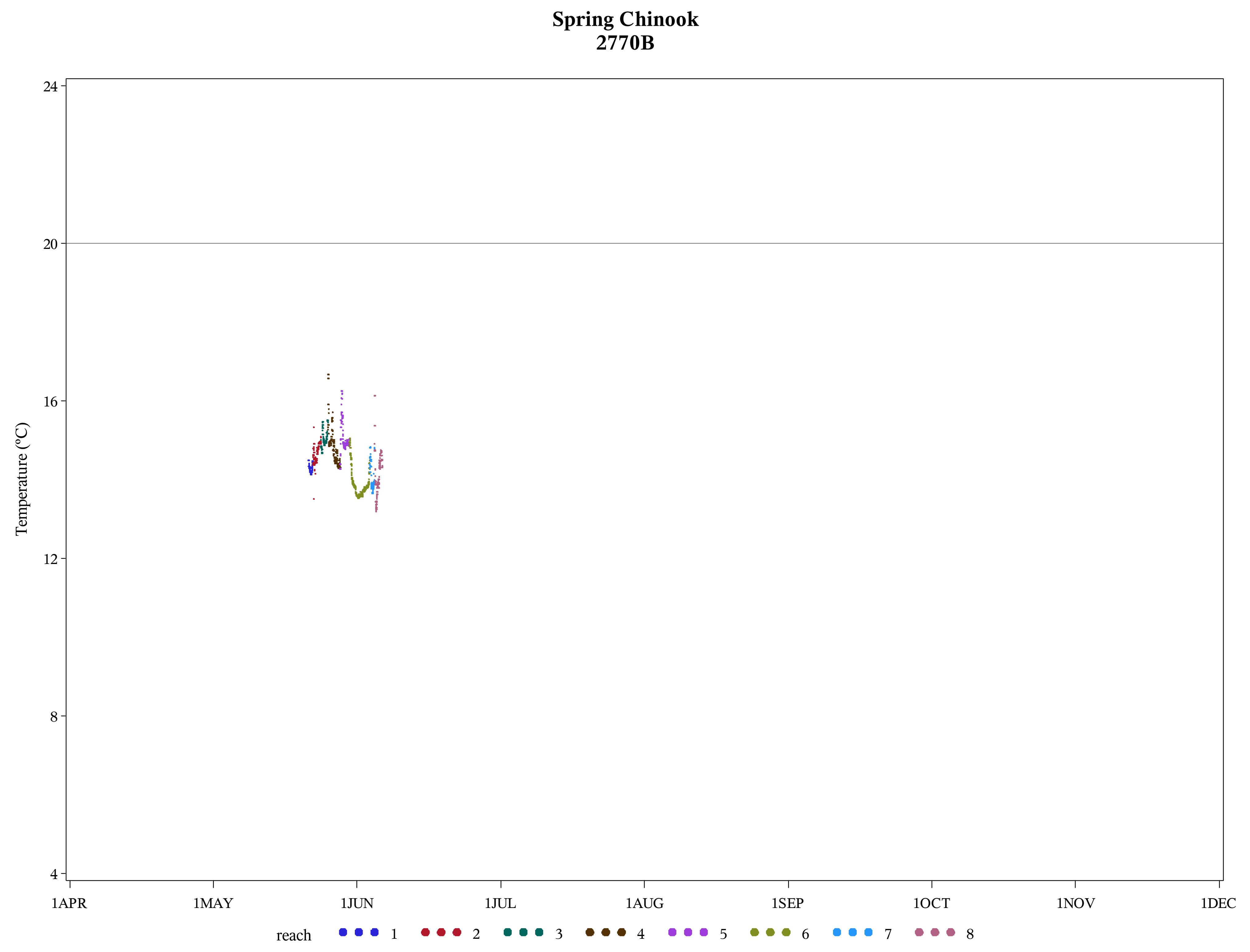

Spring Chinook
2770B
Temperature (°C)
24
20
16
12
8
4
1APR
1MAY
1JUN
1JUL
1AUG
1SEP
1OCT
1NOV
1DEC
reach
1
2
3
4
5
6
7
8

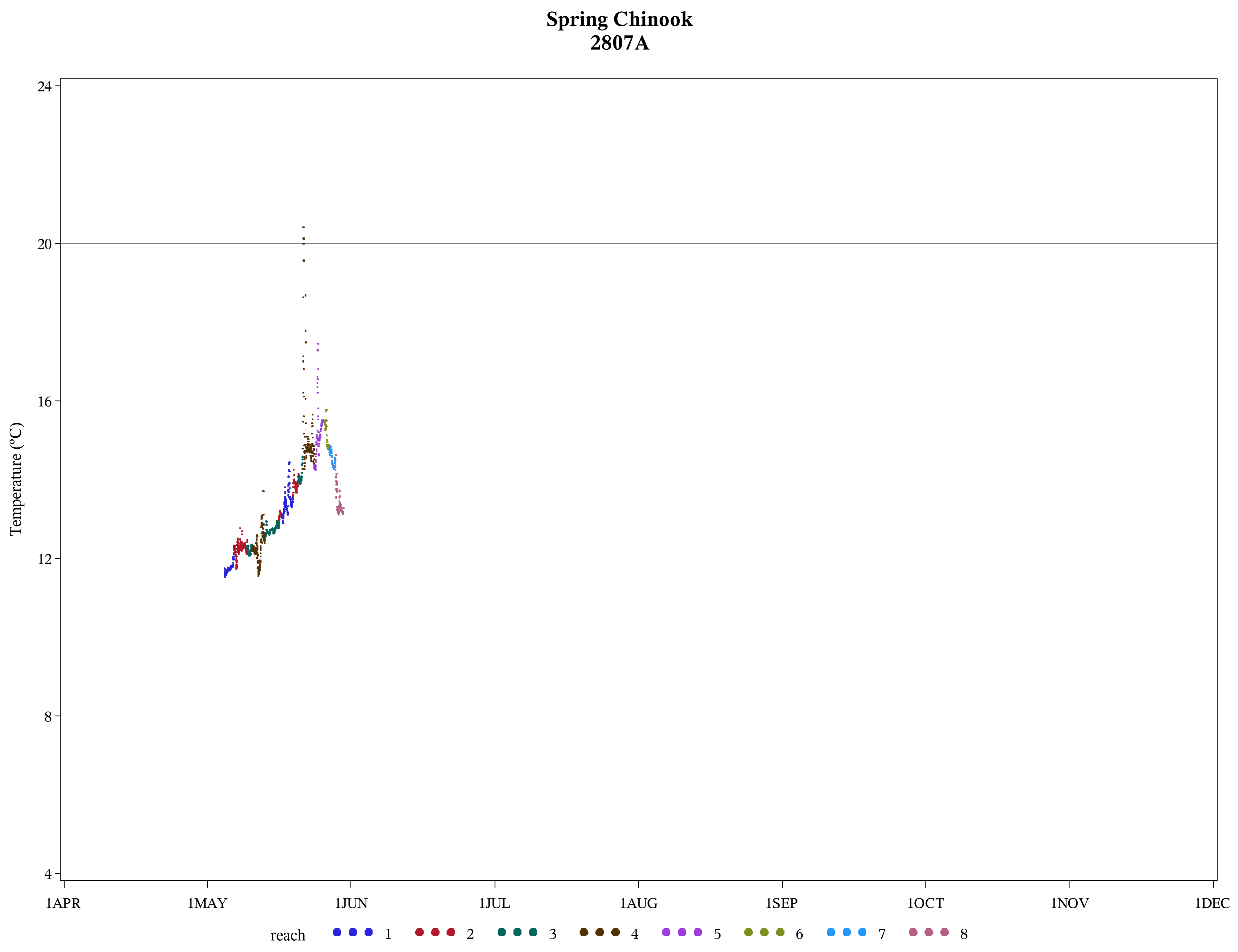

Spring Chinook
2807A
Temperature (°C)
24
20
16
12
8
4
1APR
1MAY
1JUN
1JUL
1AUG
1SEP
1OCT
1NOV
1DEC
reach
1
2
3
4
5
6
7
8

Spring Chinook
2999A

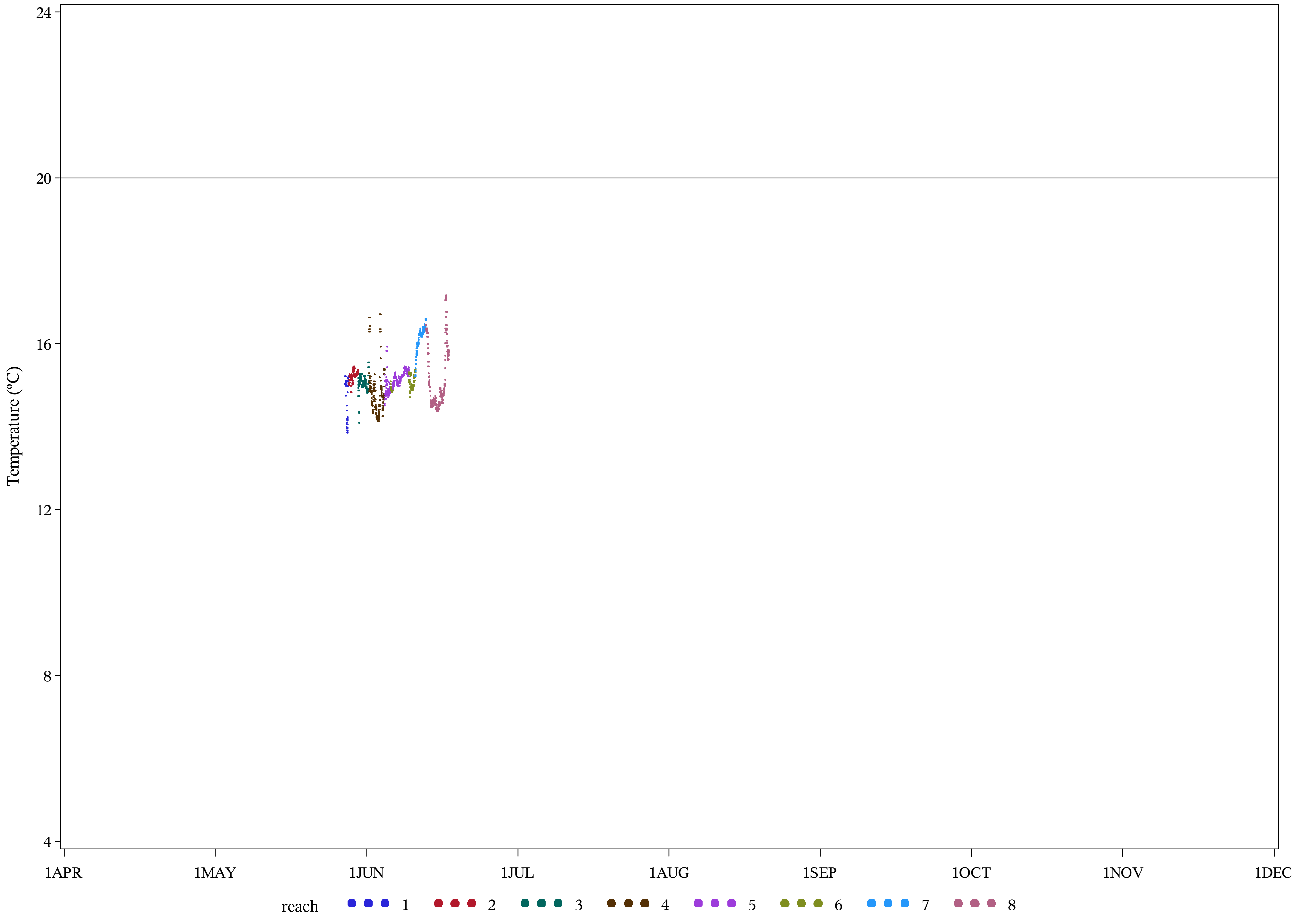

Spring Chinook
3033A

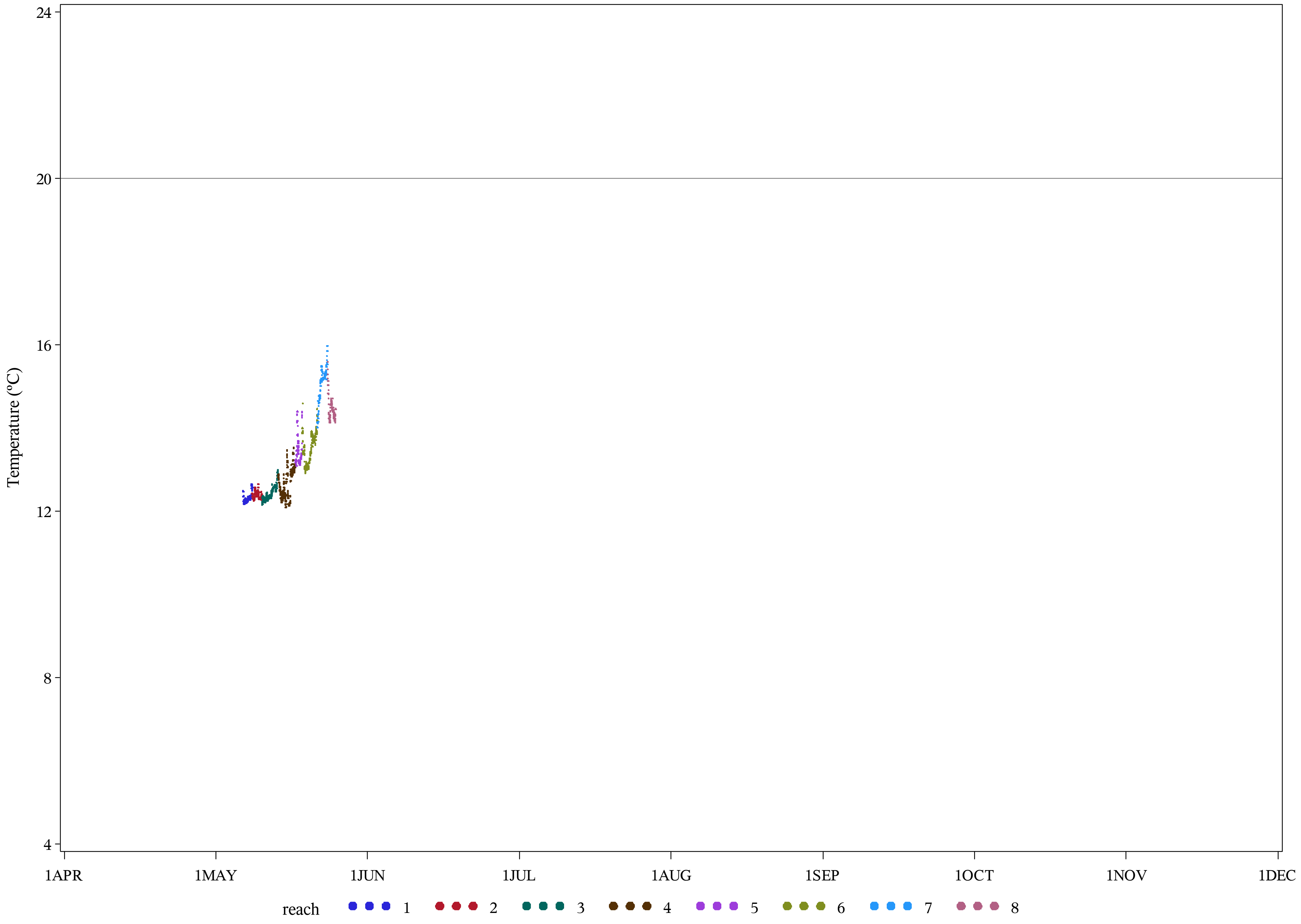

Spring Chinook
3043A

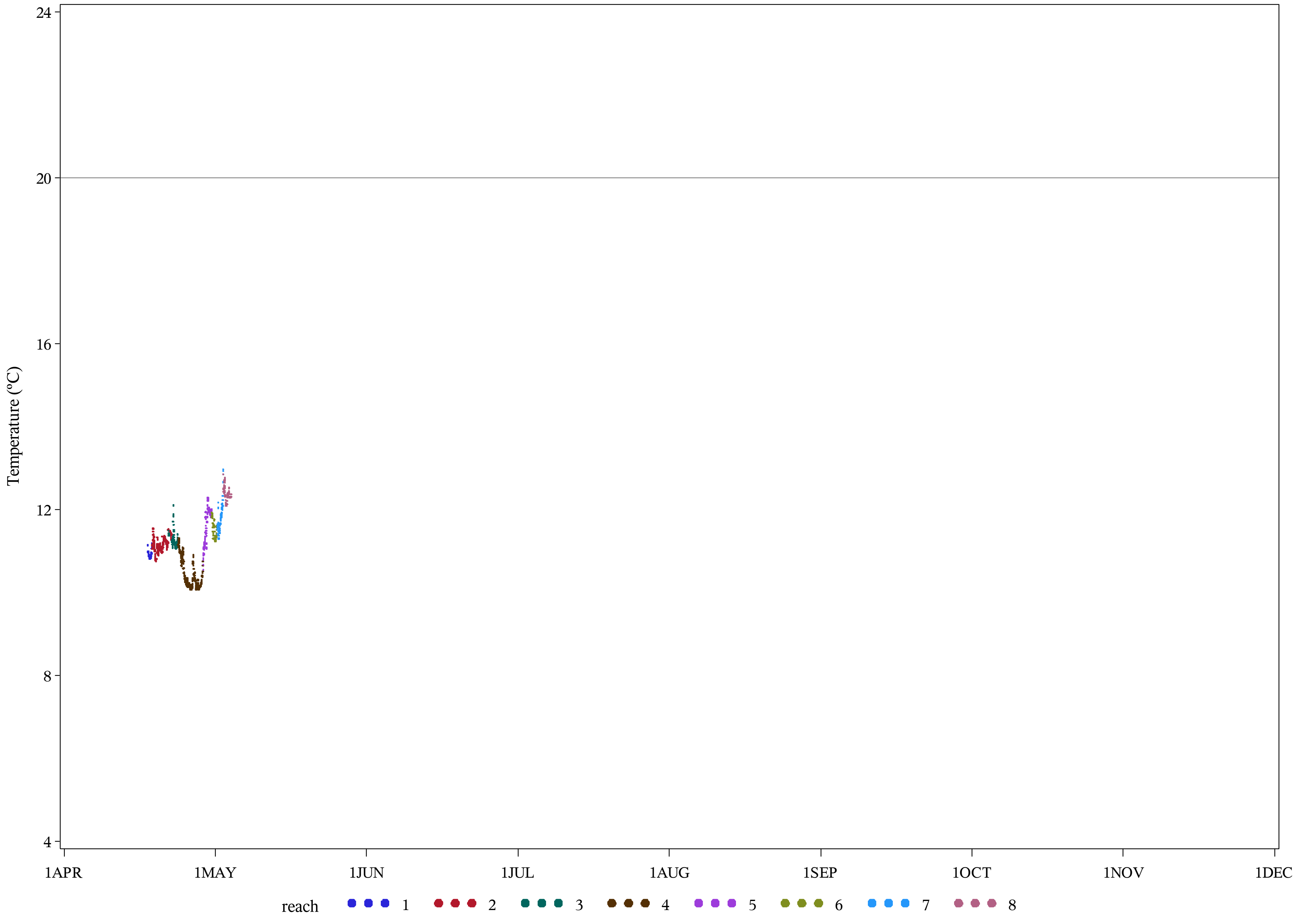

Spring Chinook
3047A

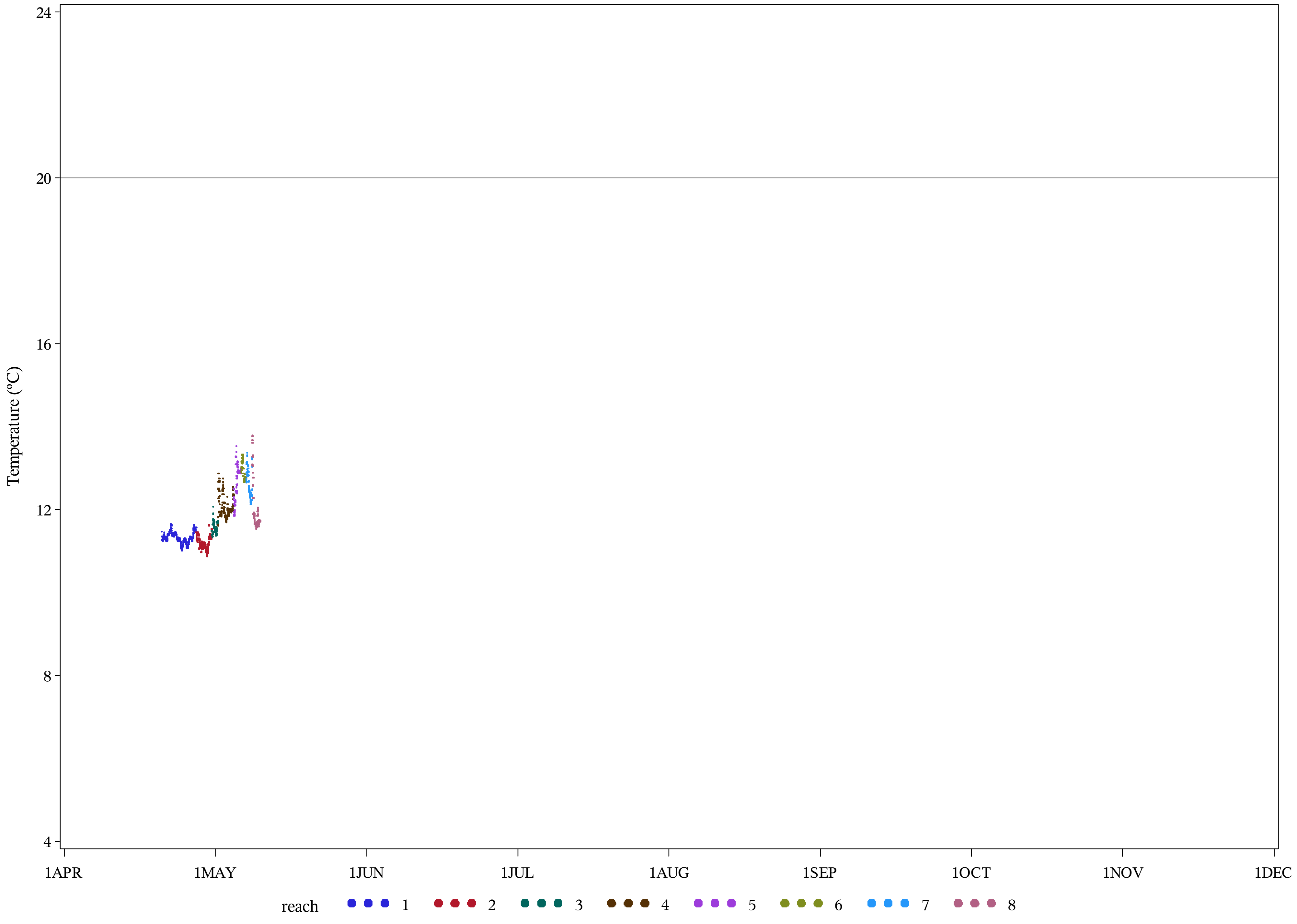

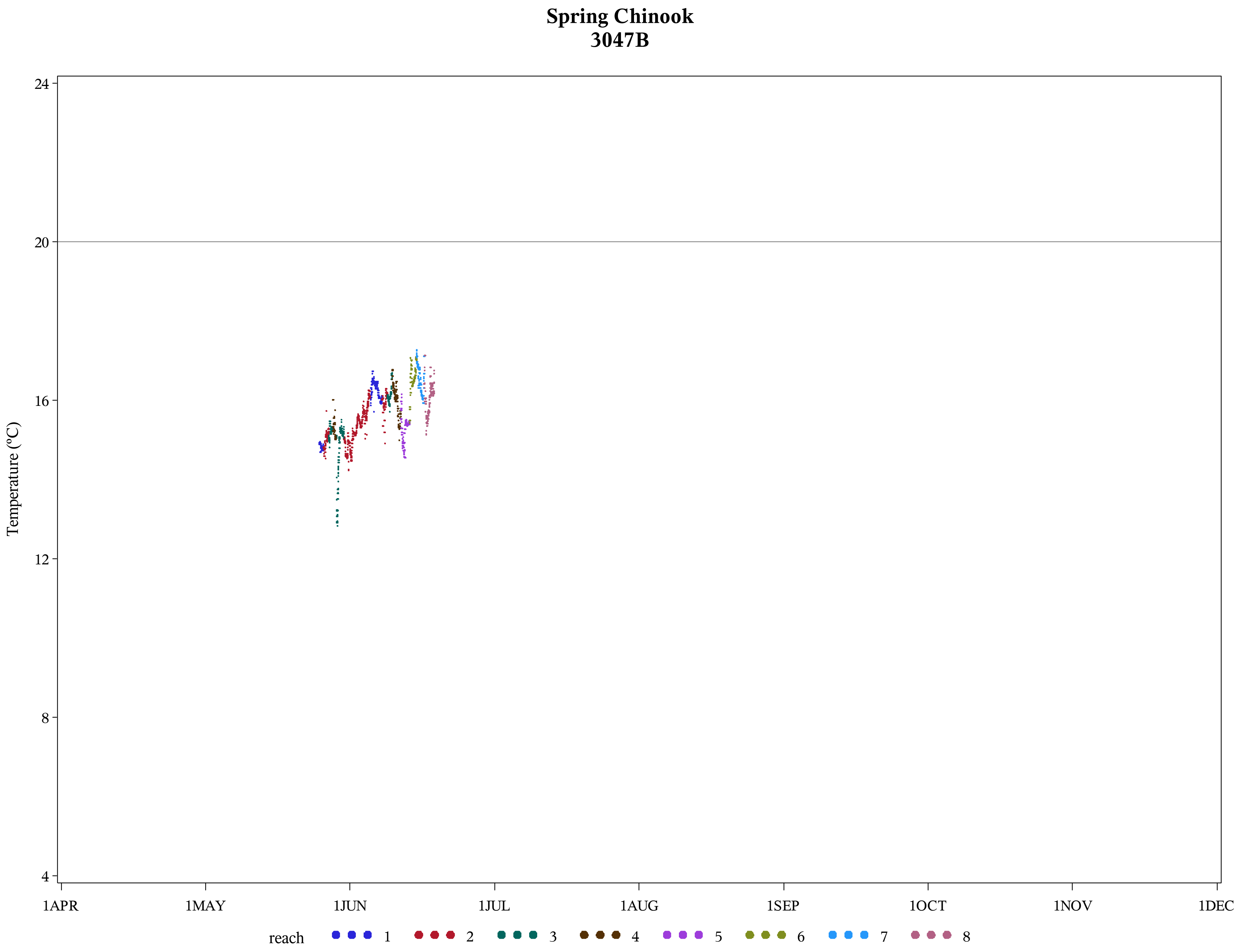
Spring Chinook
3047B
Temperature (°C)
24
20
16
12
8
4
1APR
1MAY
1JUN
1JUL
1AUG
1SEP
1OCT
1NOV
1DEC
reach
1
2
3
4
5
6
7
8

Spring Chinook
3051A

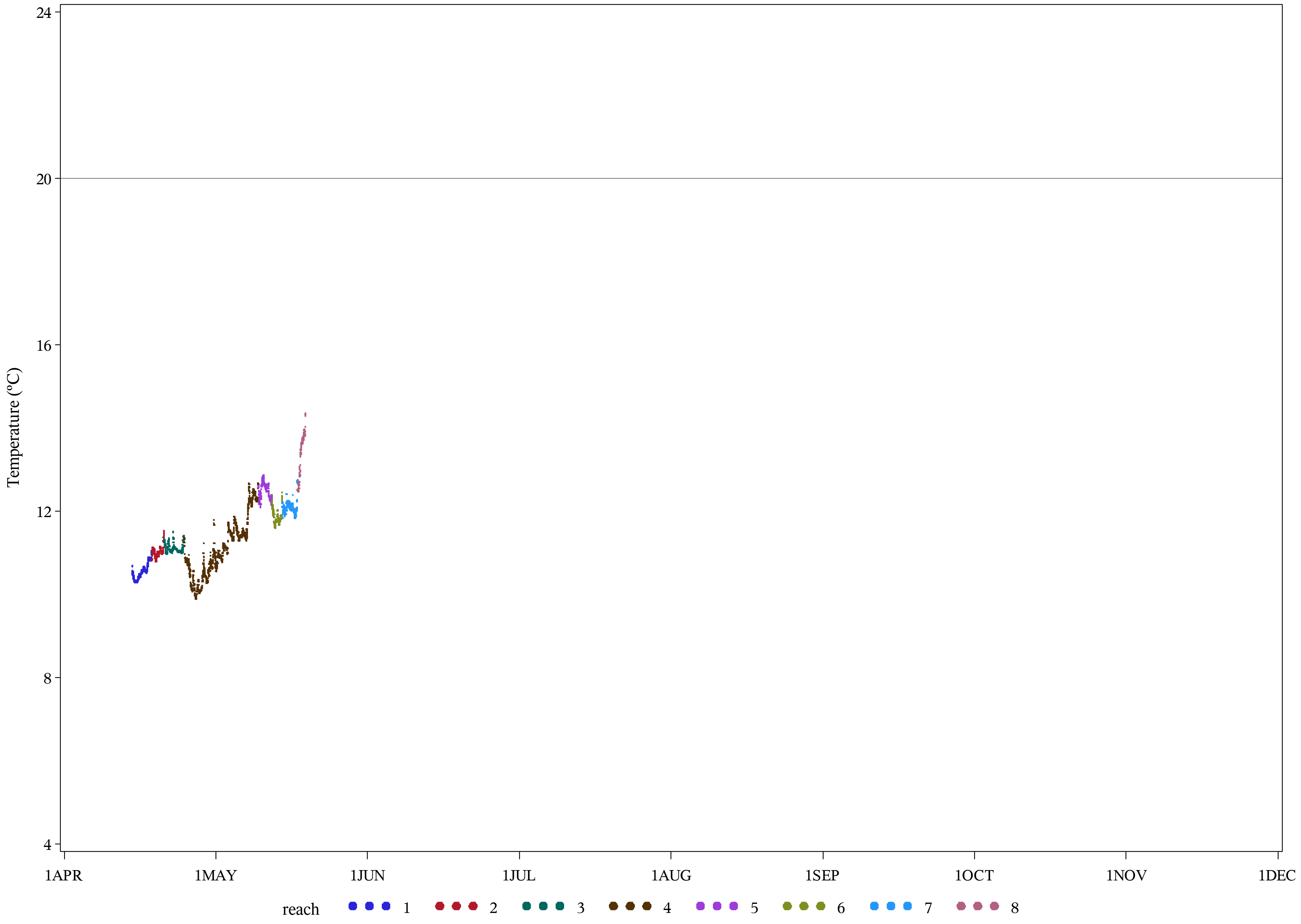

Spring Chinook
3056A

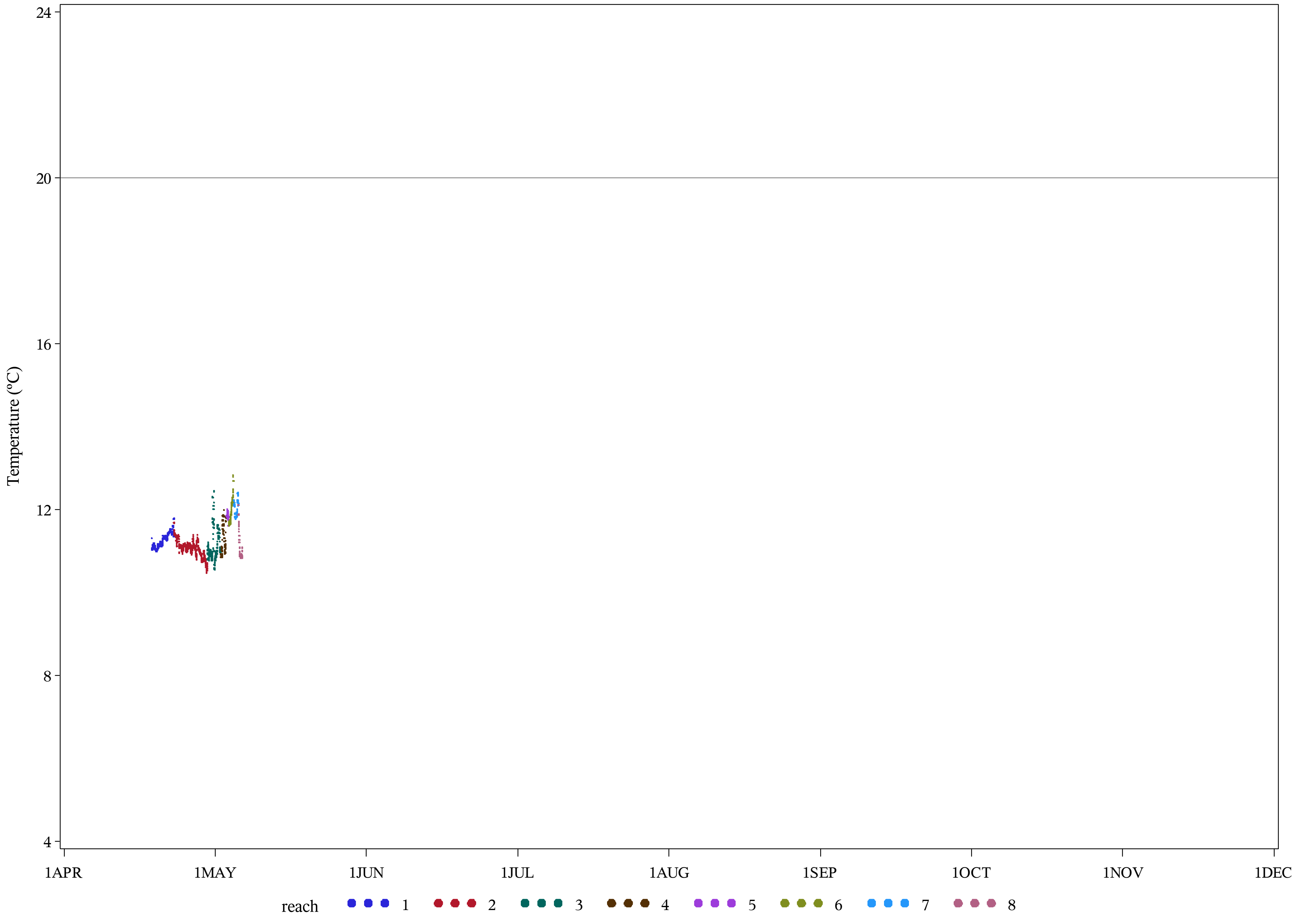

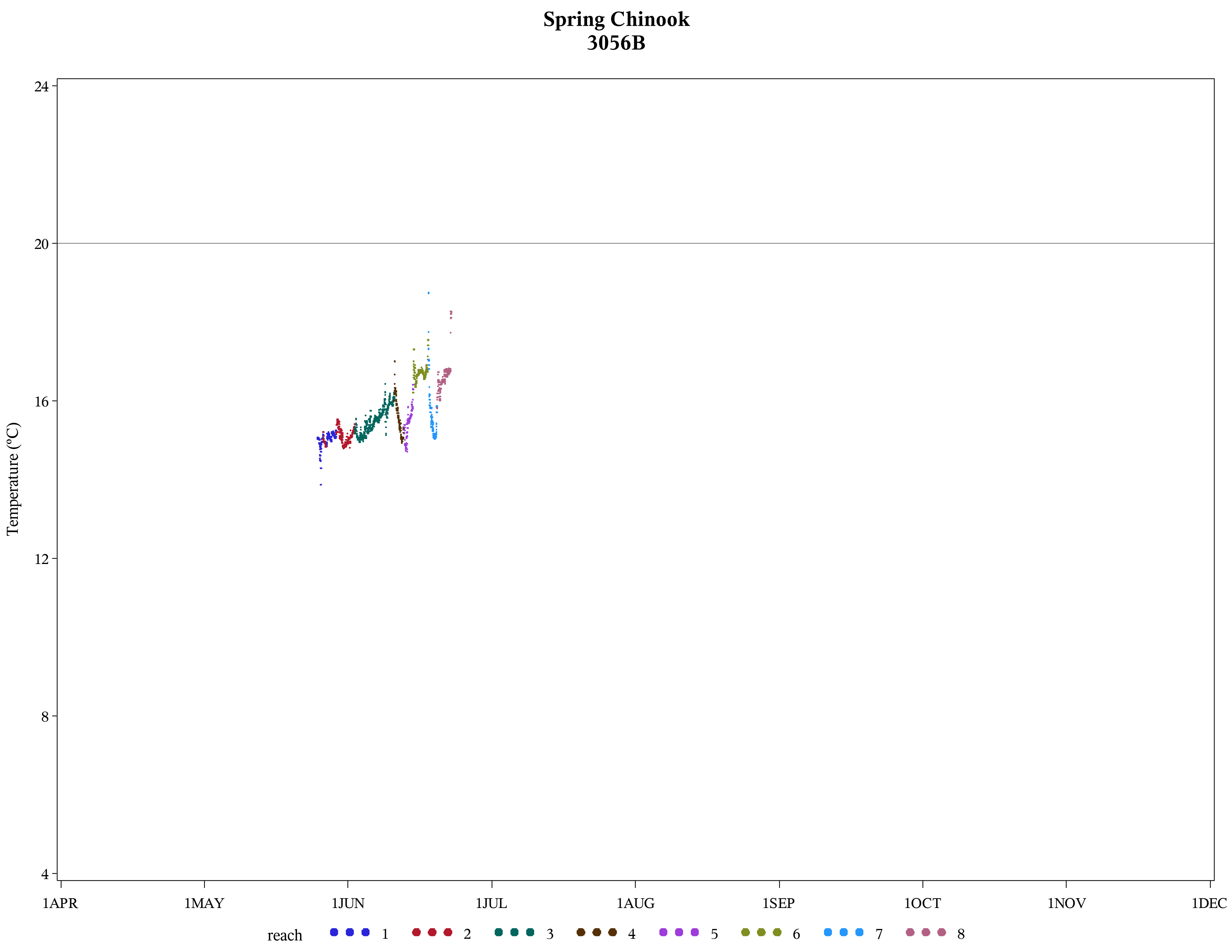

Spring Chinook
3056B
Temperature (°C)
24
20
16
12
8
4
1APR
1MAY
1JUN
1JUL
1AUG
1SEP
1OCT
1NOV
1DEC
reach
1
2
3
4
5
6
7
8

Spring Chinook
3058A

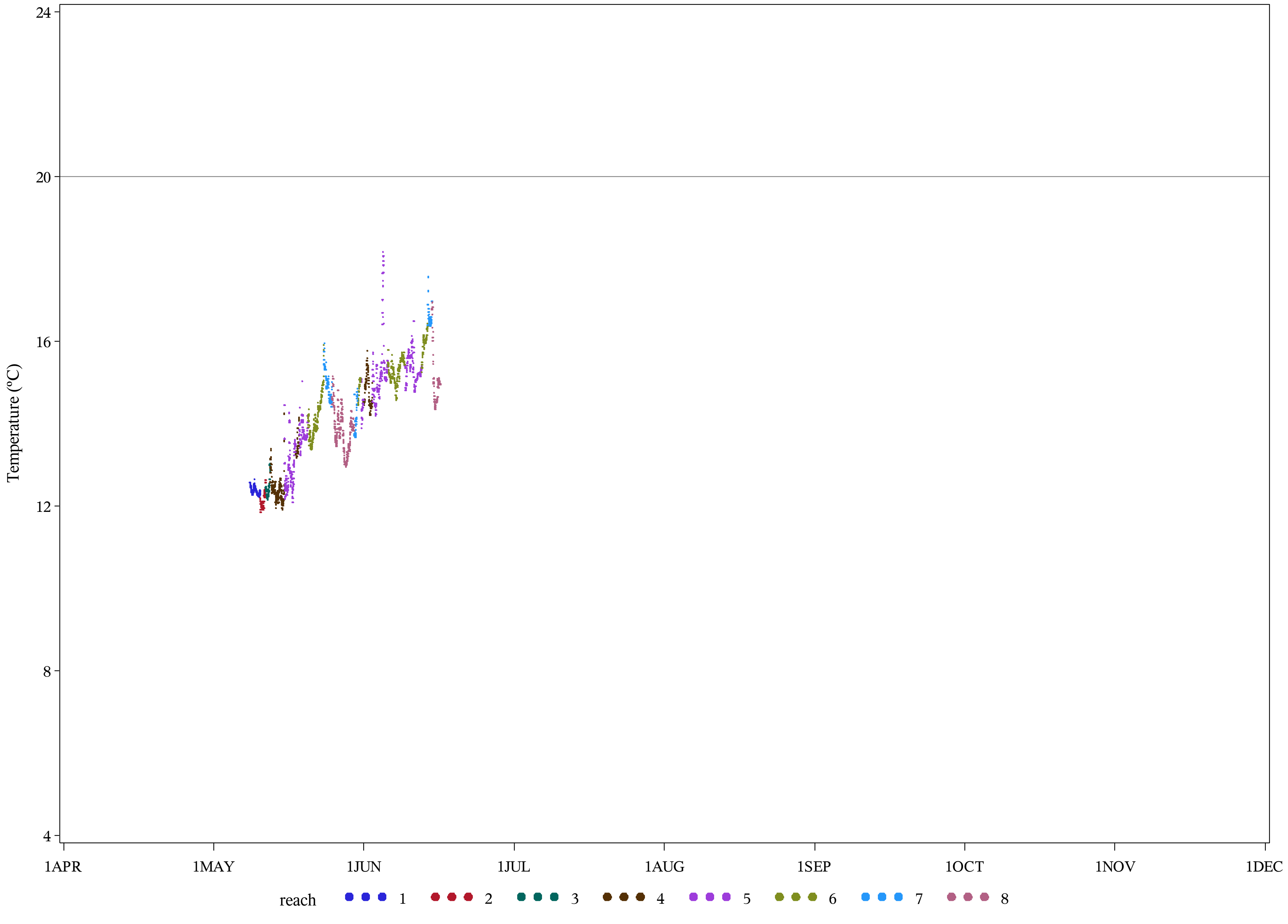

Spring Chinook
3061A

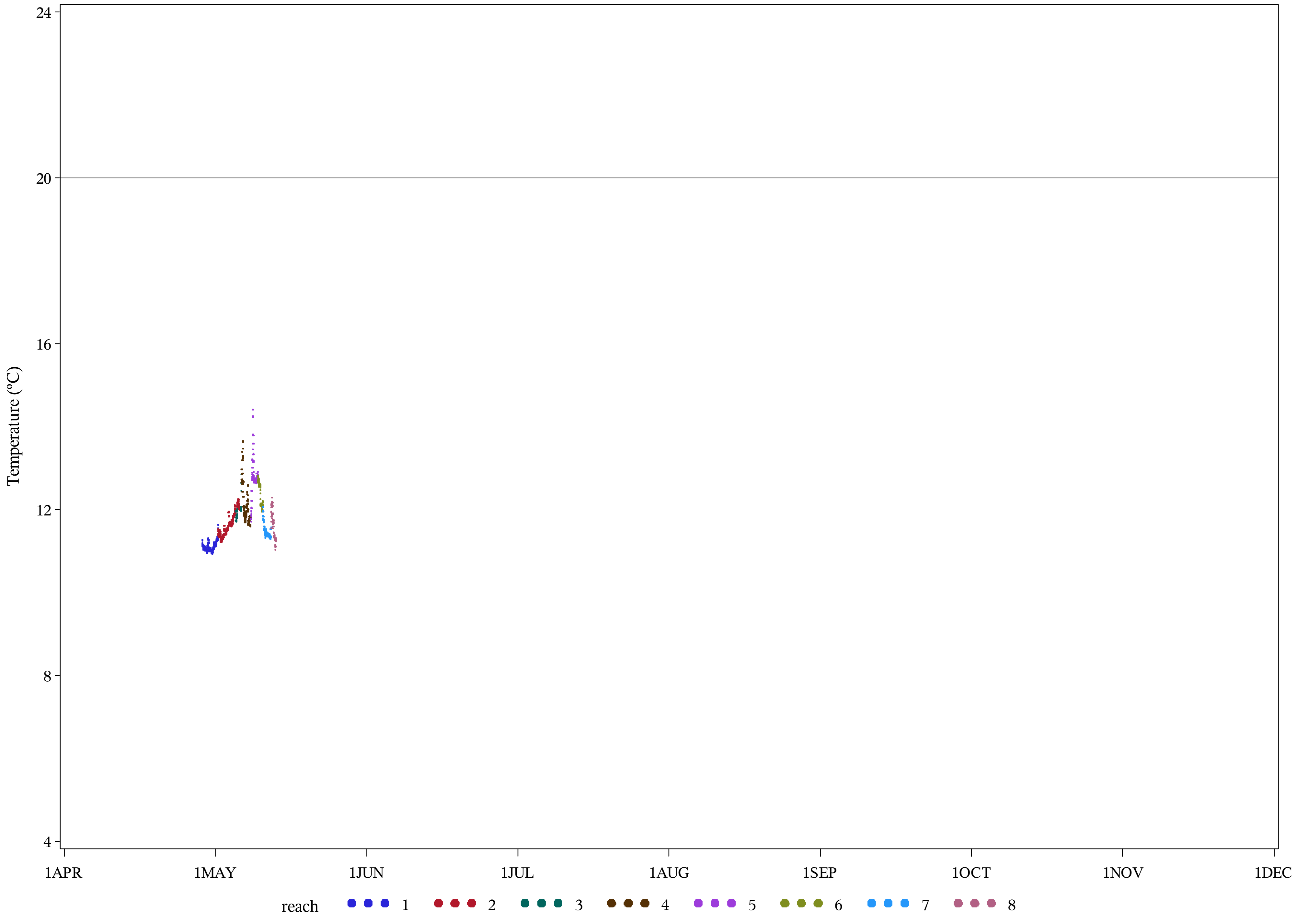

Spring Chinook
3063A

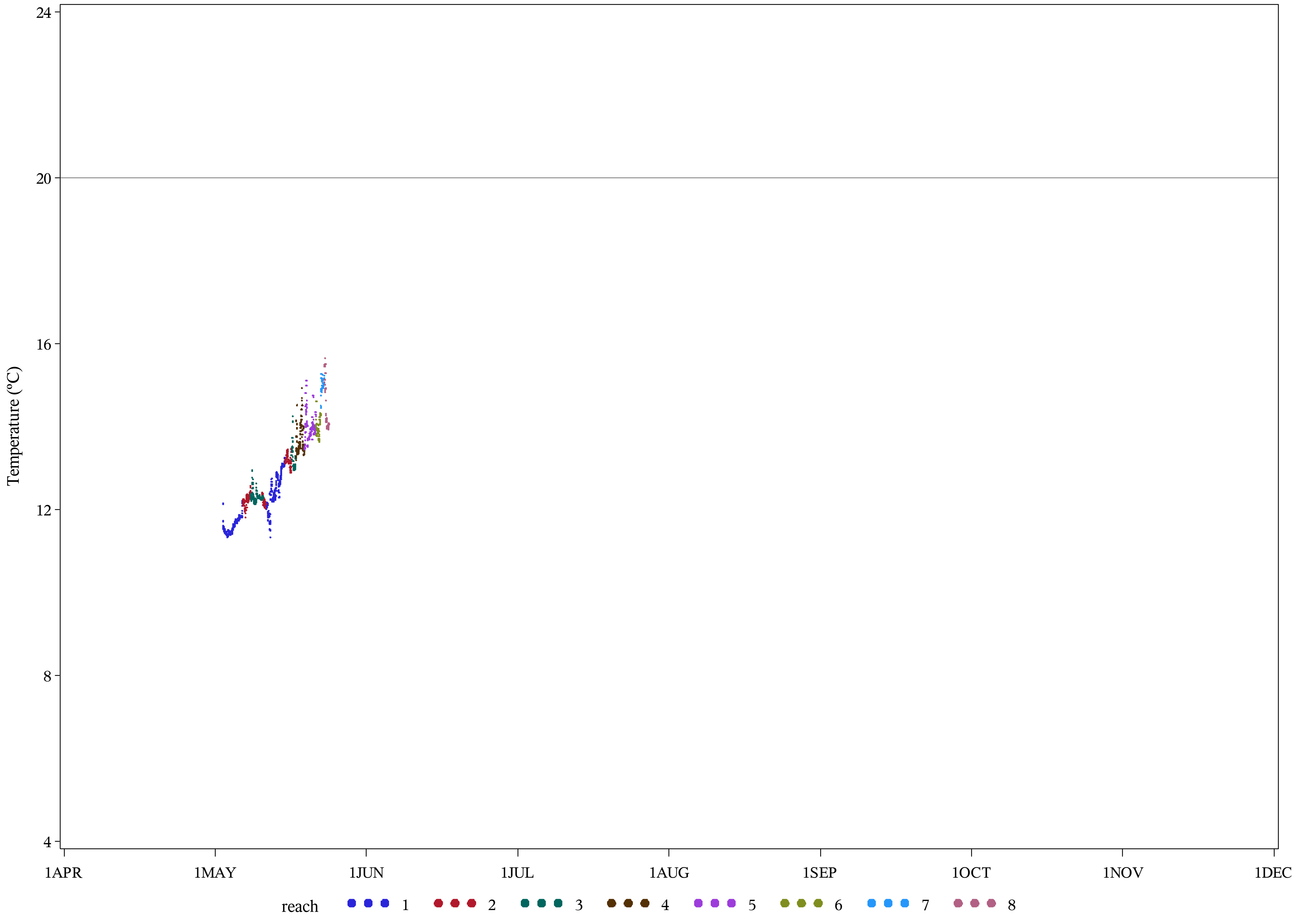

Spring Chinook
3069A

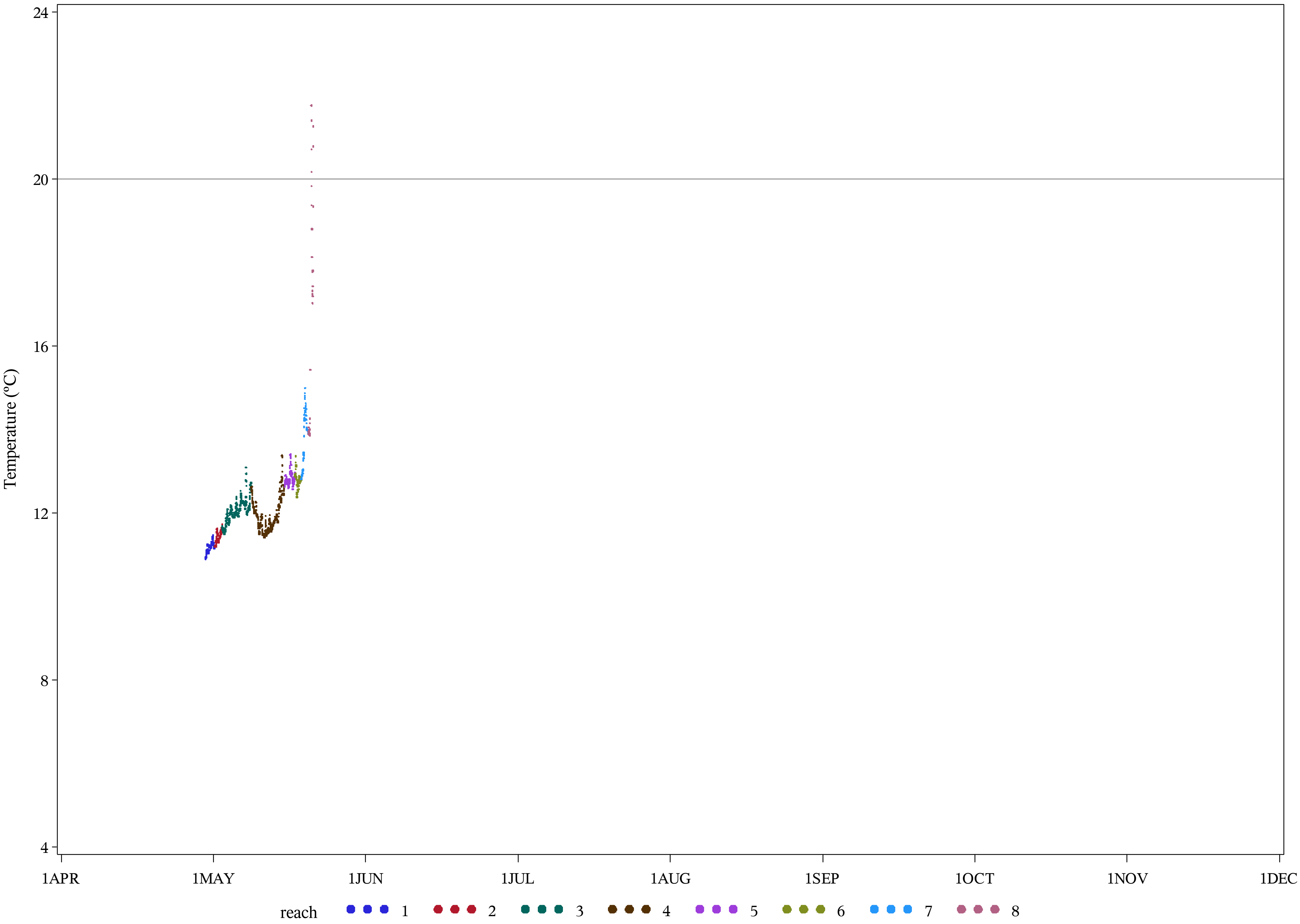

Spring Chinook
3072A

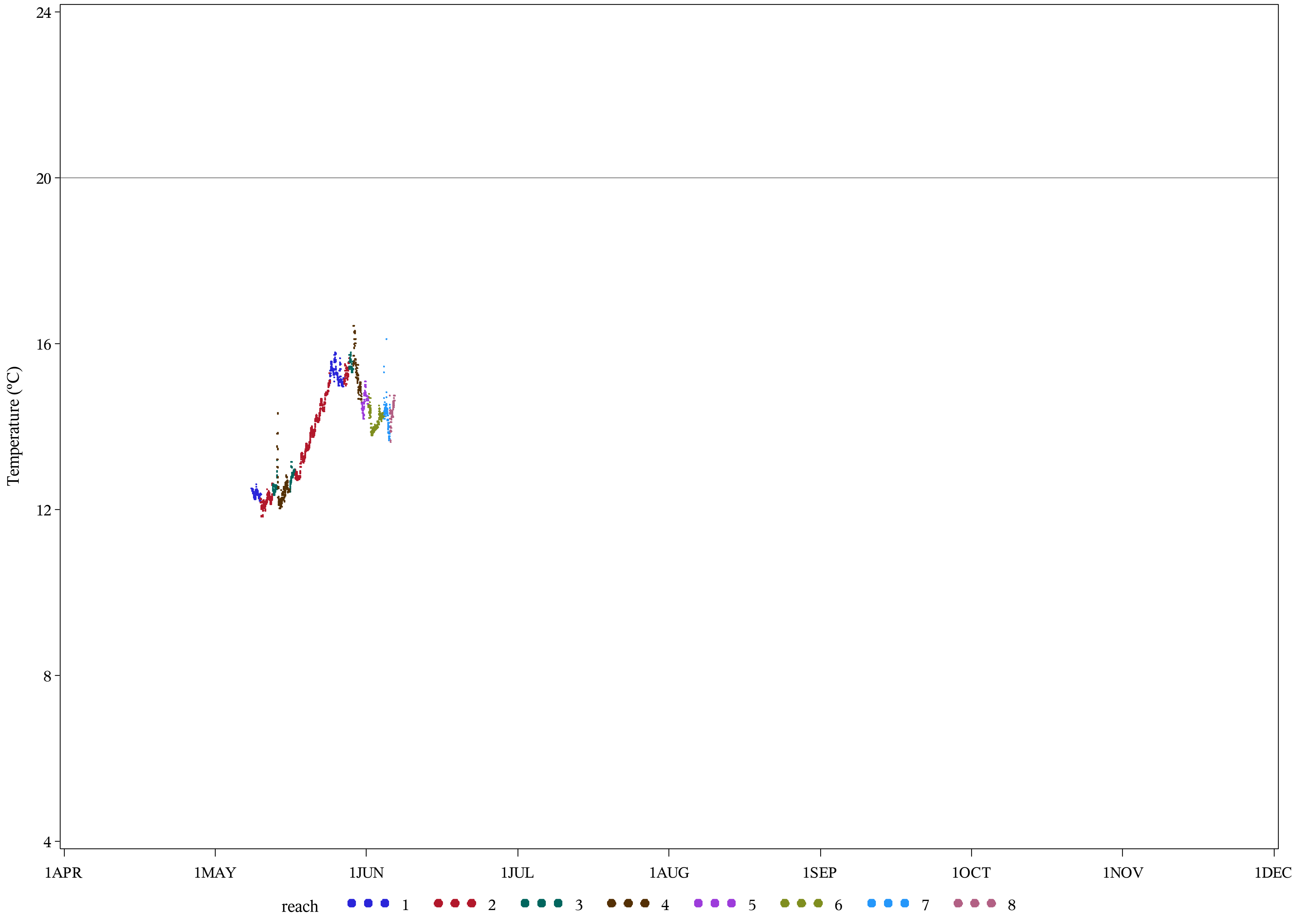

Spring Chinook
3076A

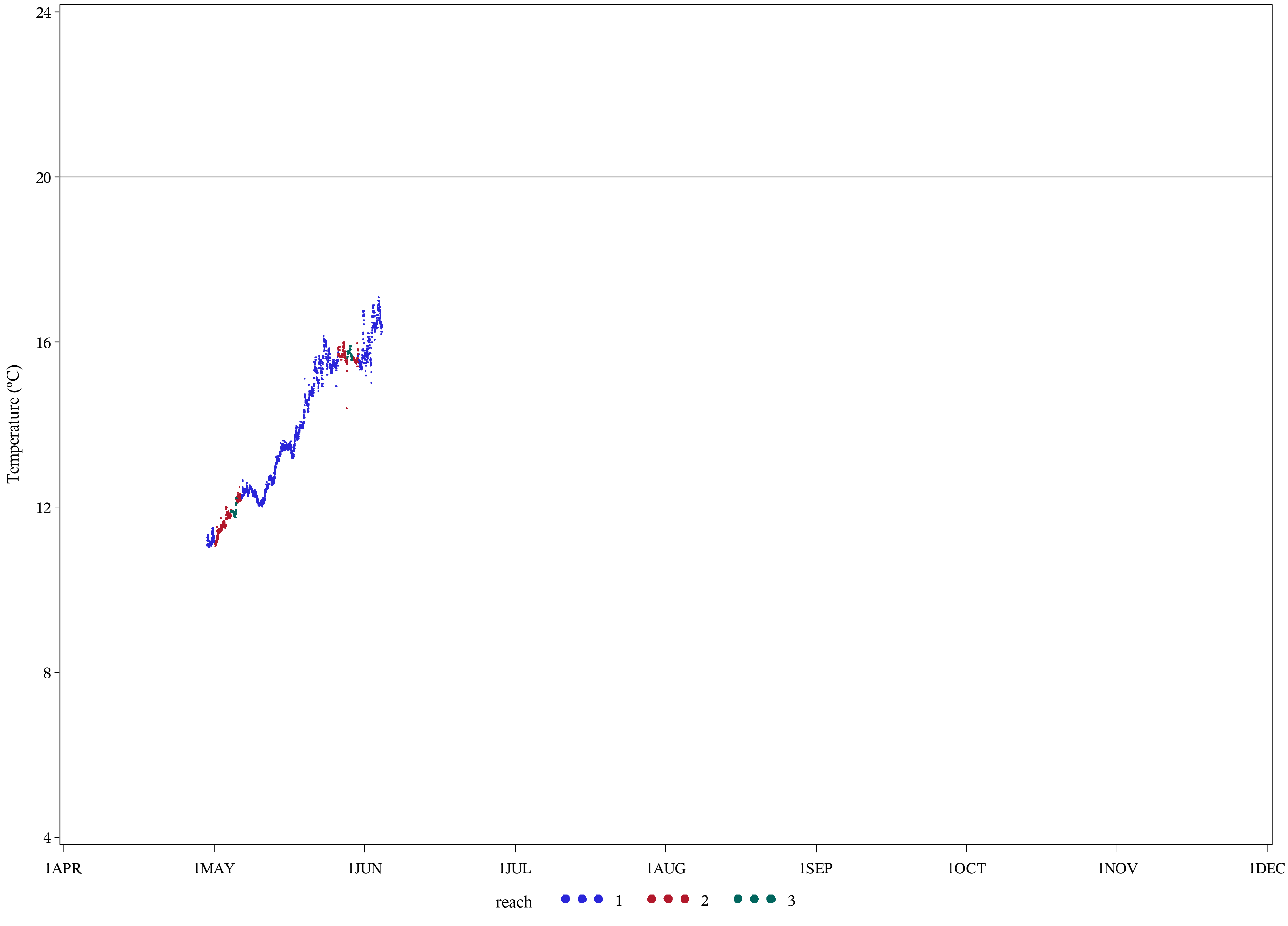

Spring Chinook
3081A

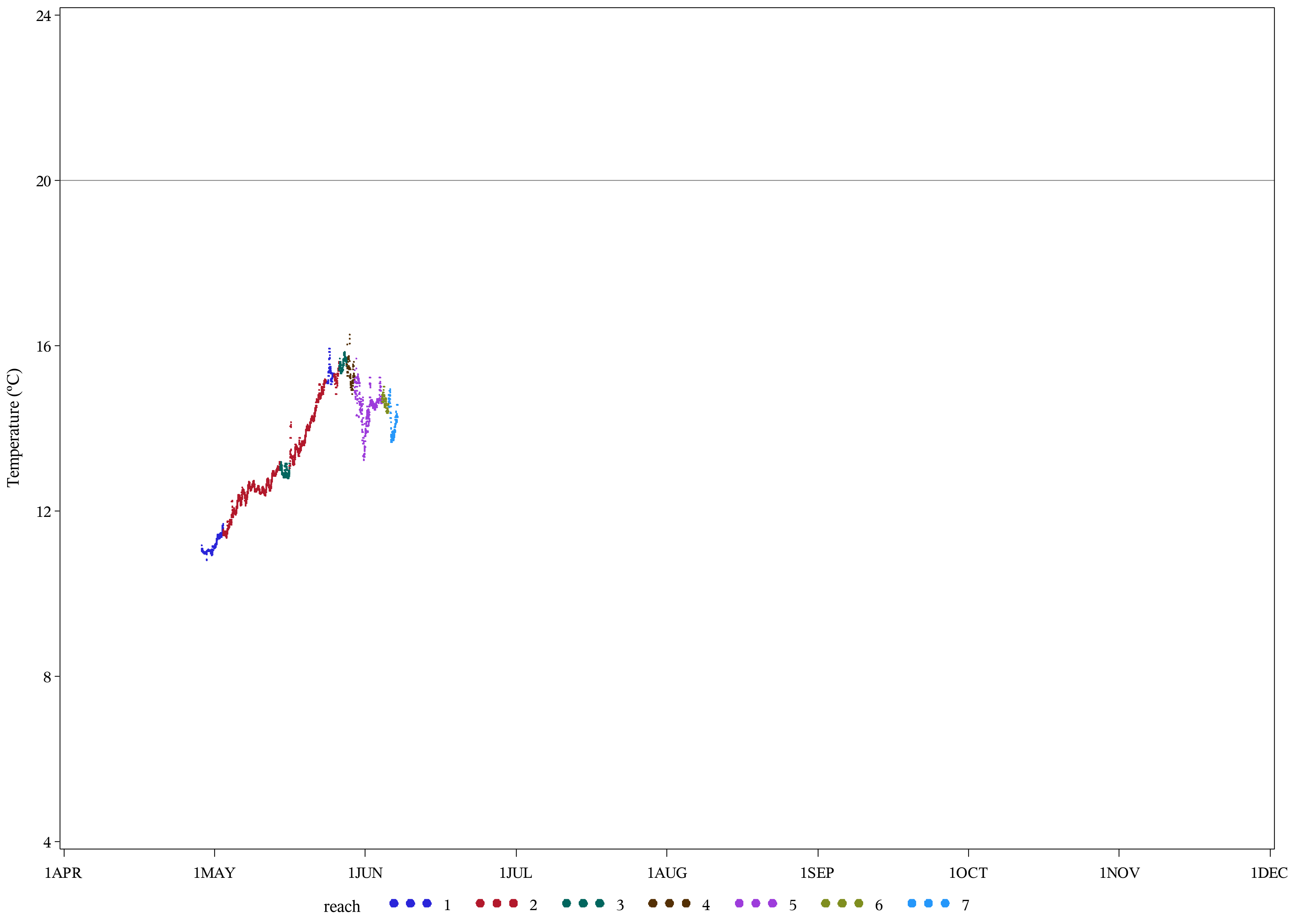

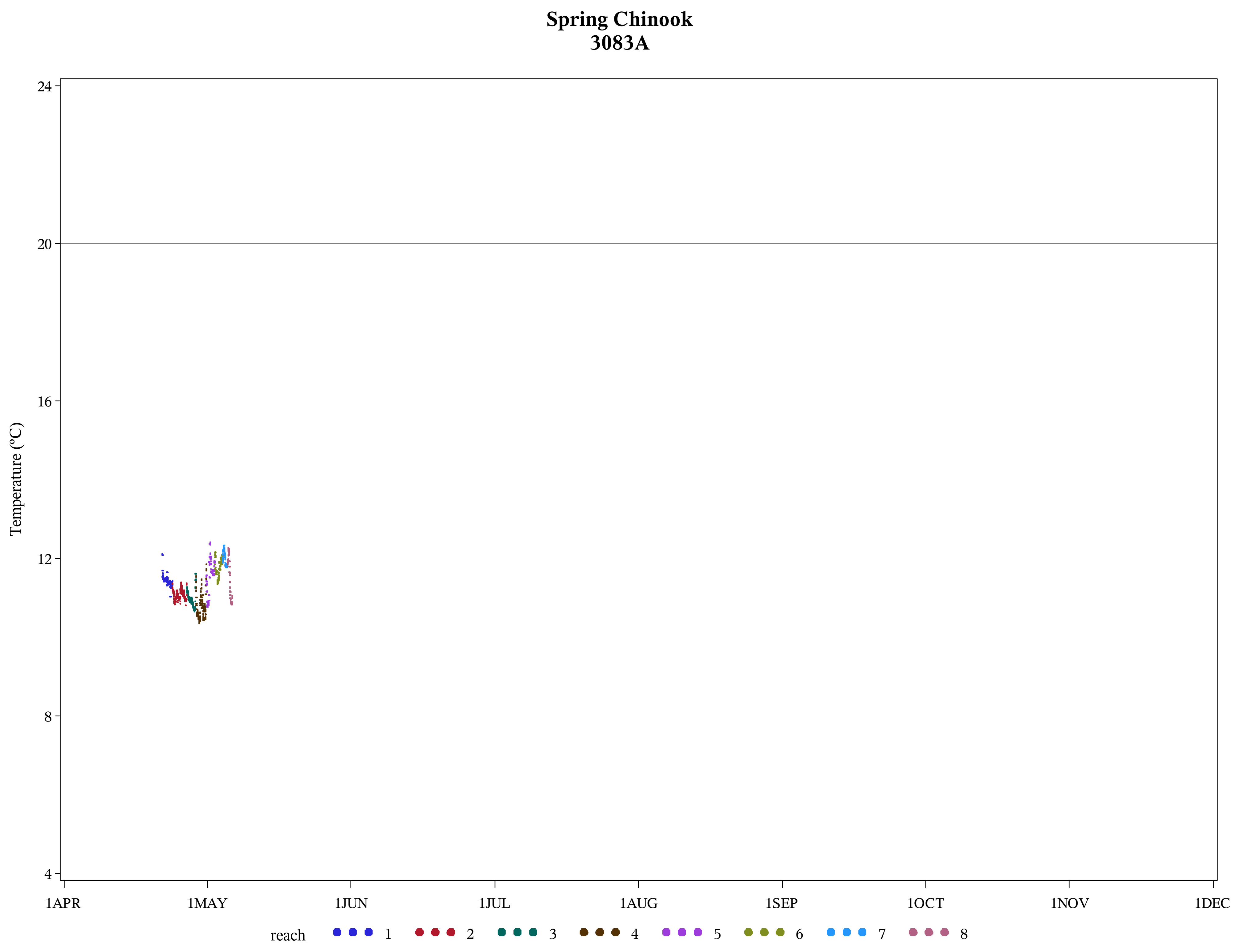
Spring Chinook
3083A
Temperature (°C)
24
20
16
12
8
4
1APR
1MAY
1JUN
1JUL
1AUG
1SEP
1OCT
1NOV
1DEC
reach
1
2
3
4
5
6
7
8

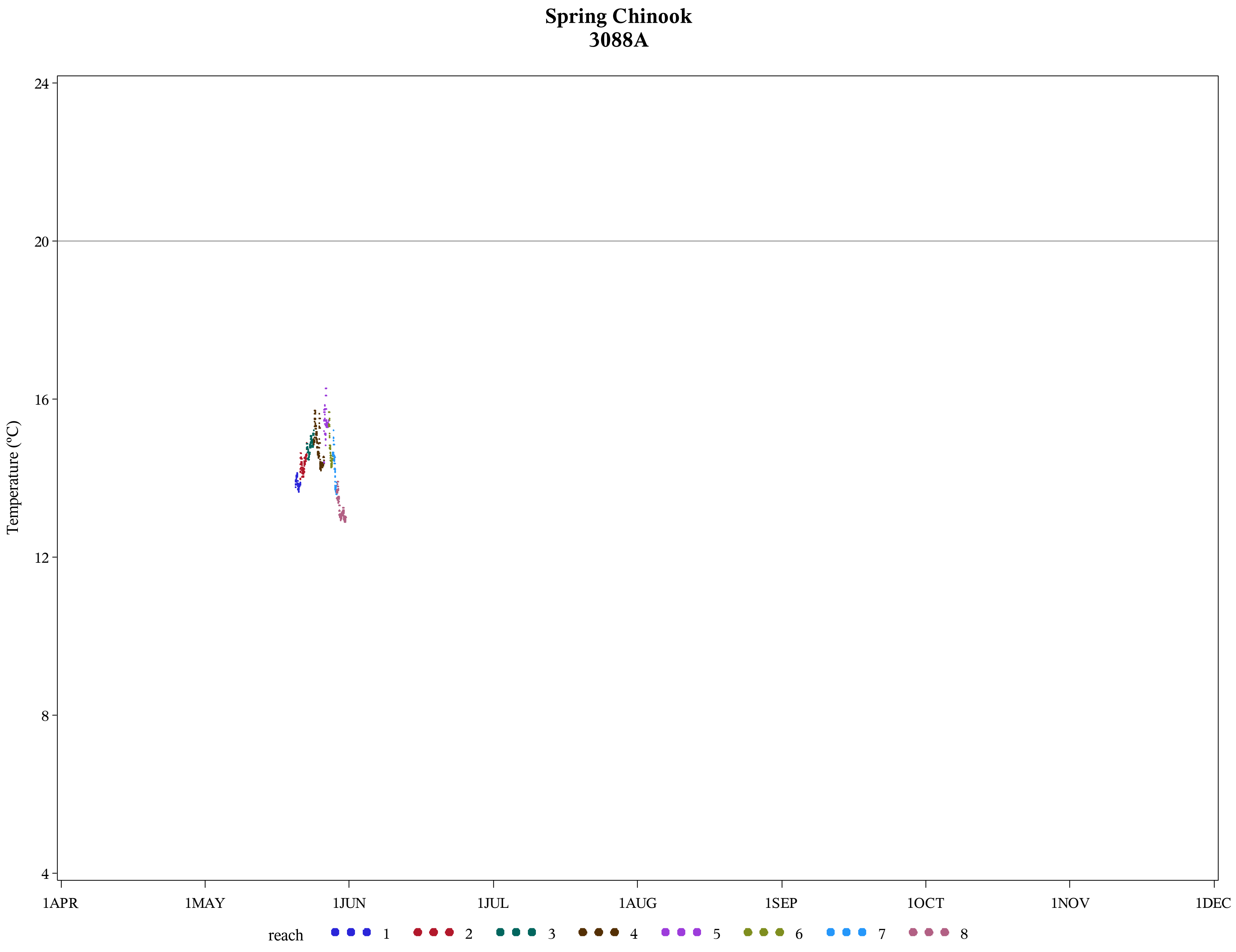
Spring Chinook
3088A
Temperature (°C)
24
20
16
12
8
4
1APR
1MAY
1JUN
1JUL
1AUG
1SEP
1OCT
1NOV
1DEC
reach
1
2
3
4
5
6
7
8

Spring Chinook
3111A

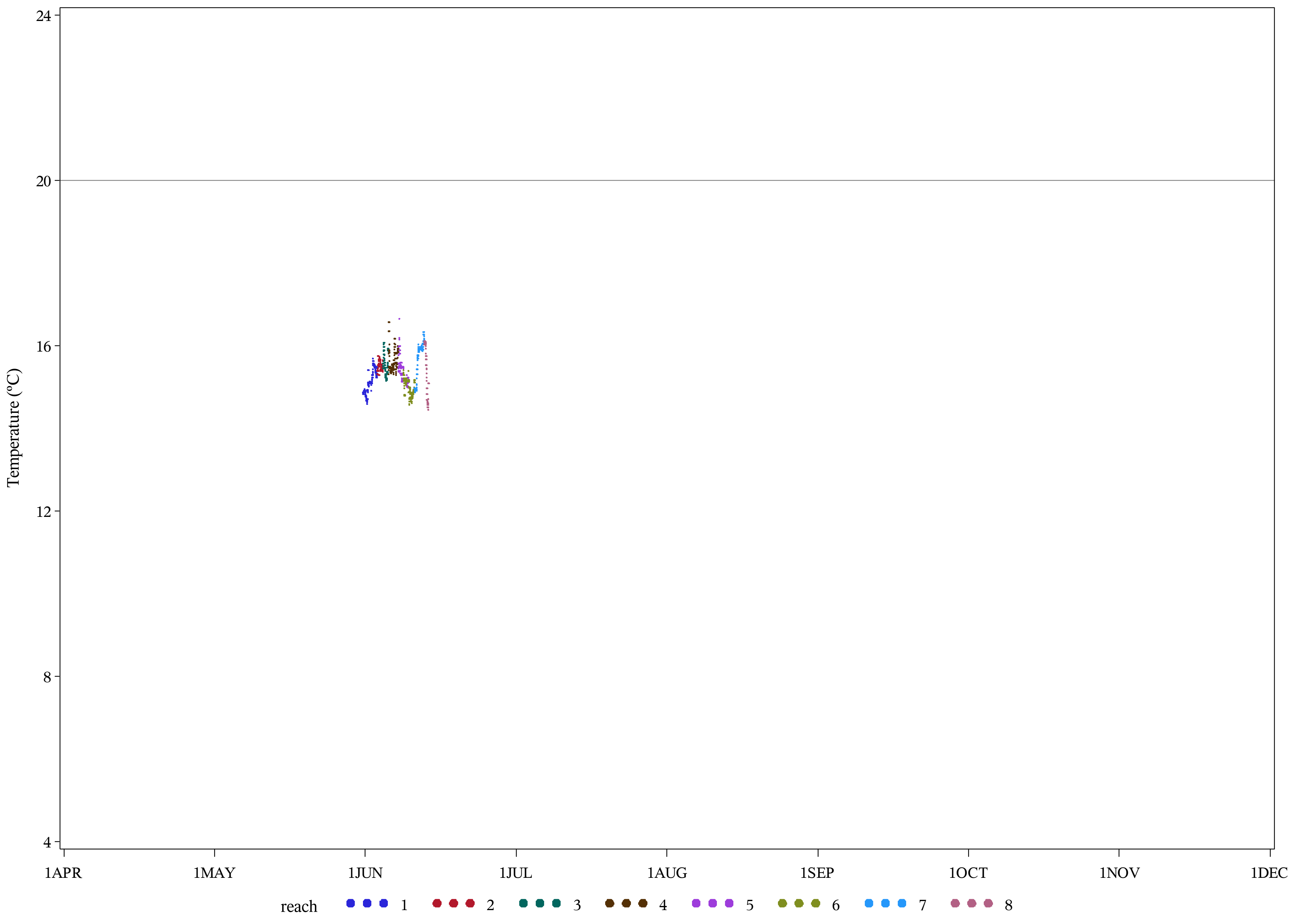

Spring Chinook
3254A

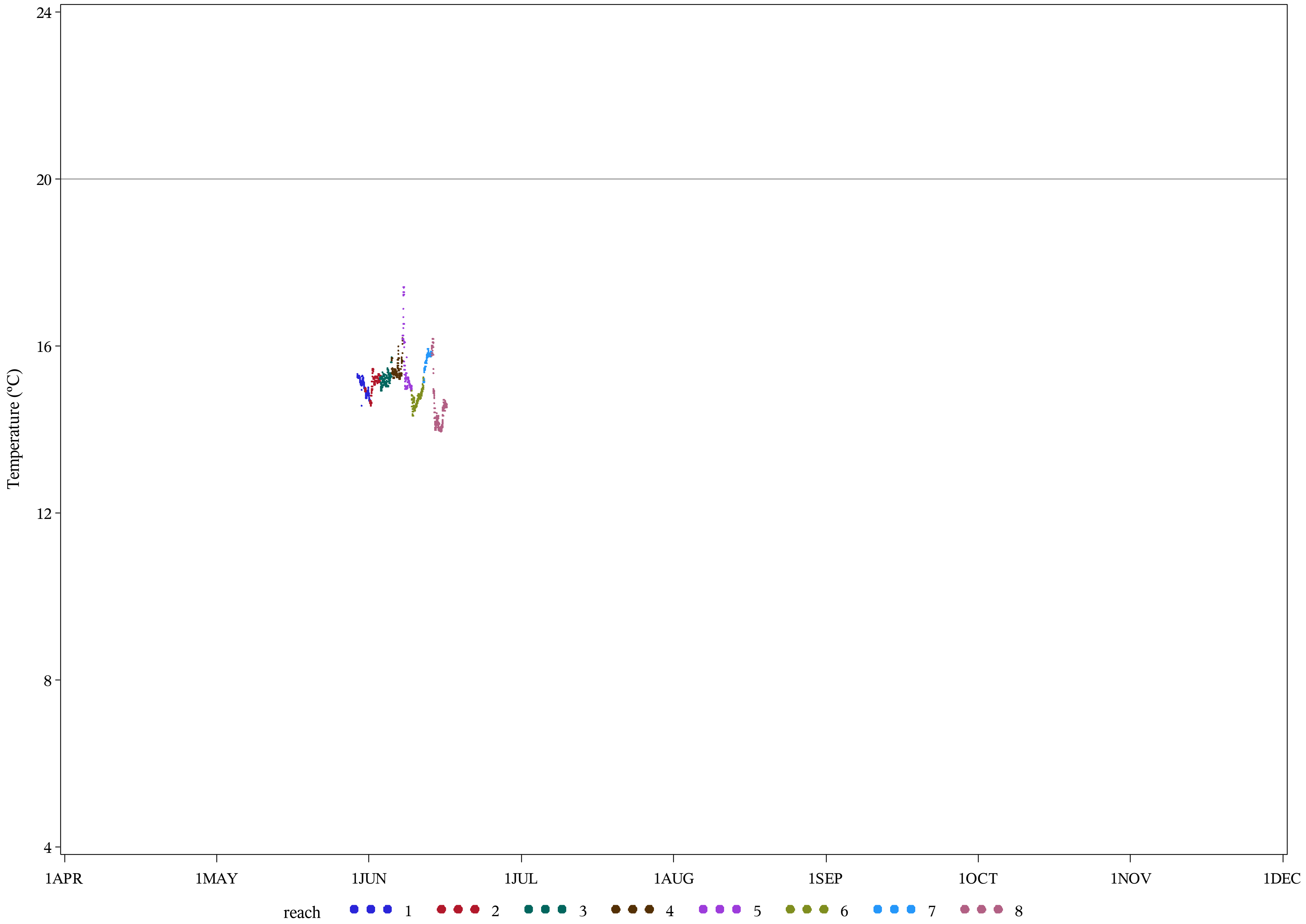

Spring Chinook
3536A

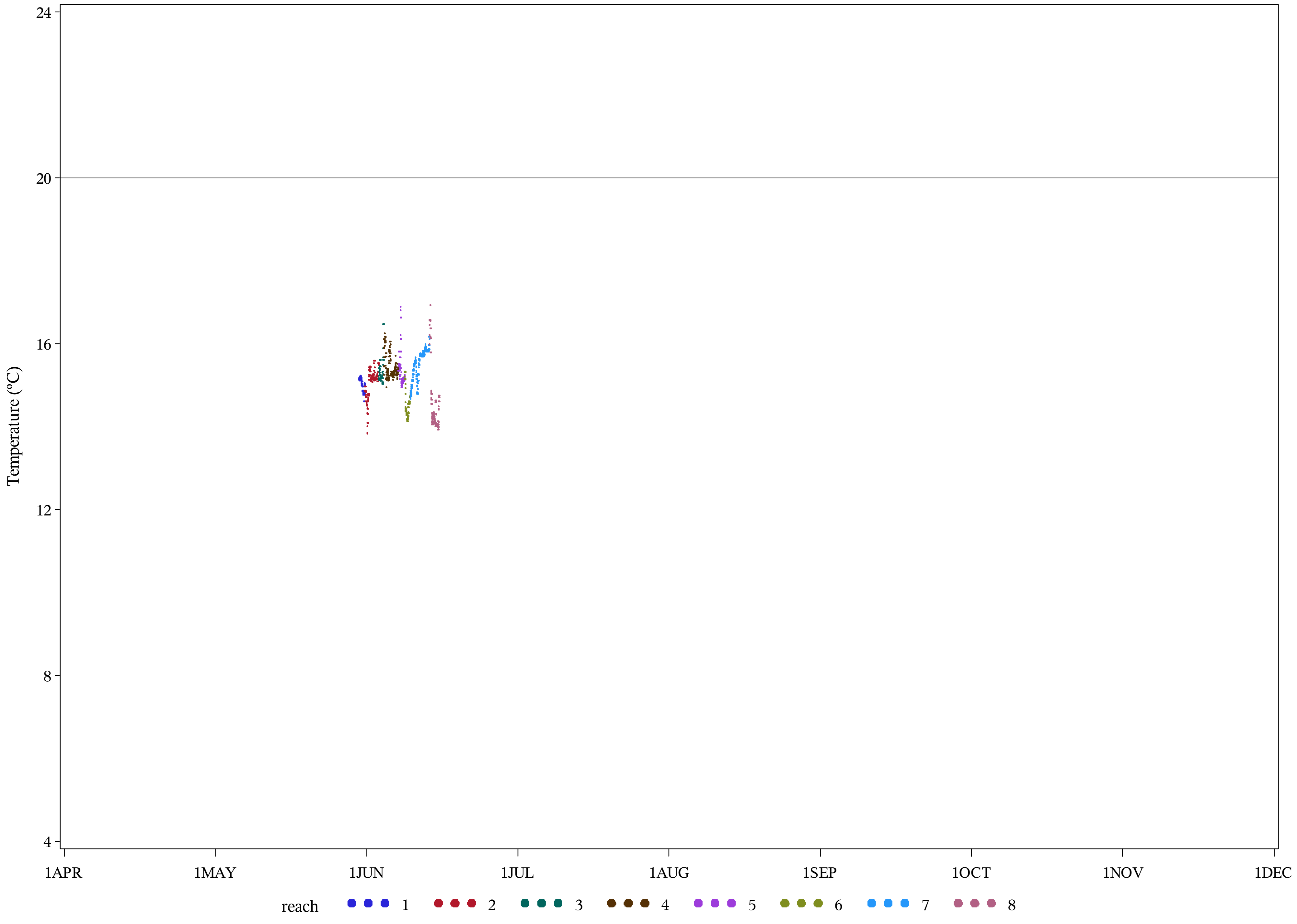

Spring Chinook
3563A

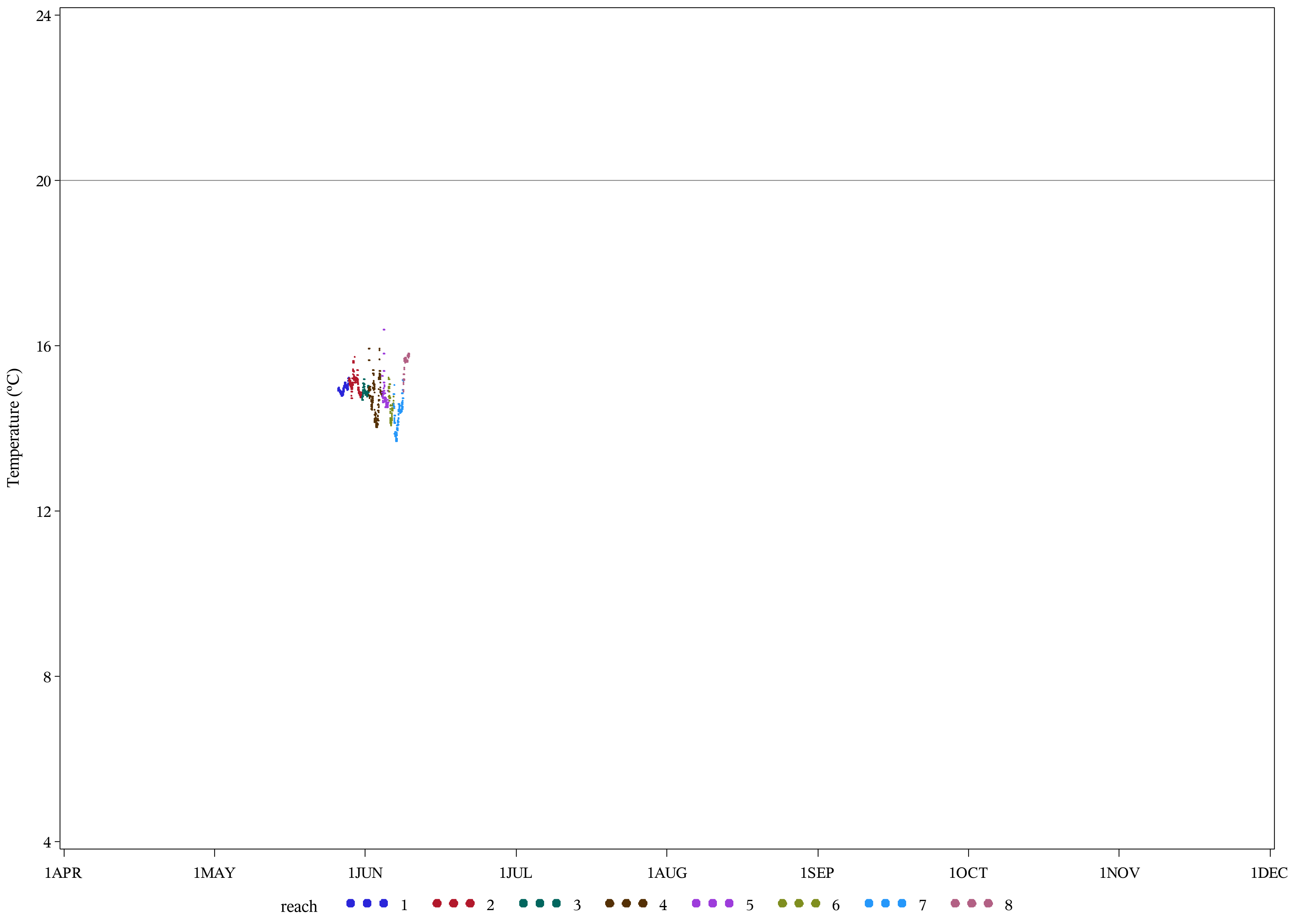

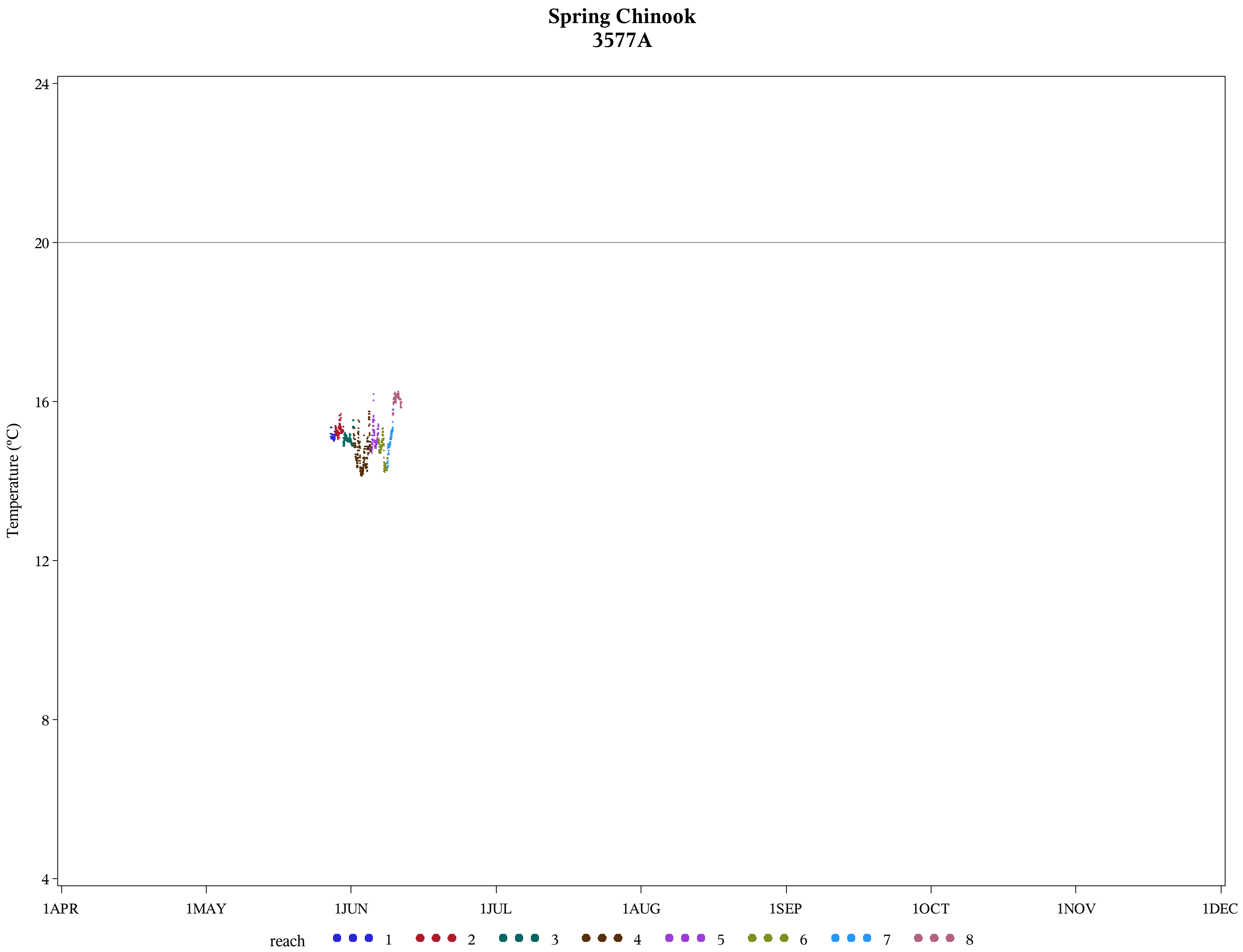
Spring Chinook
3577A
Temperature (°C)
24
20
16
12
8
4
1APR
1MAY
1JUN
1JUL
1AUG
1SEP
1OCT
1NOV
1DEC
reach
1
2
3
4
5
6
7
8

Spring Chinook
3610A

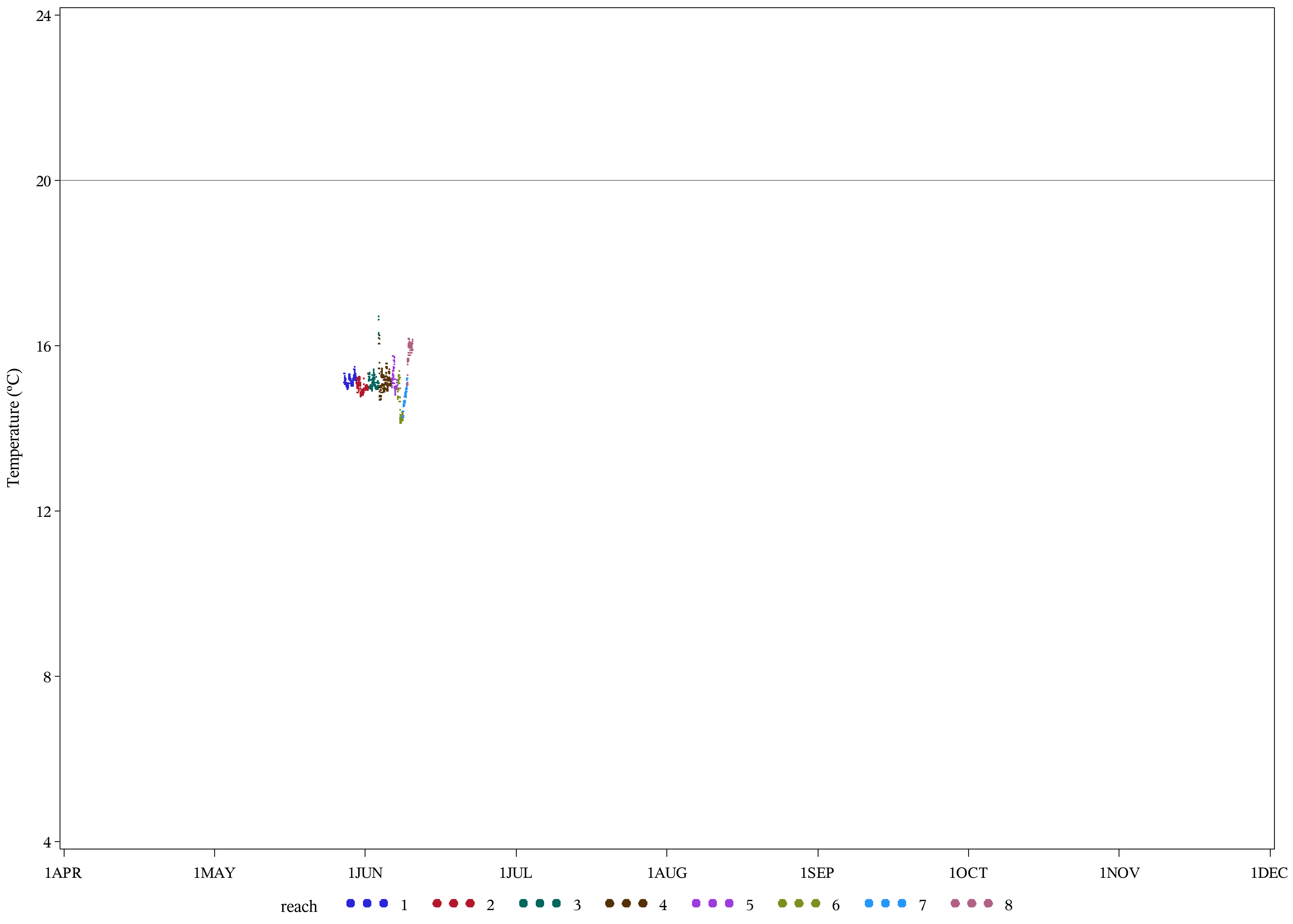

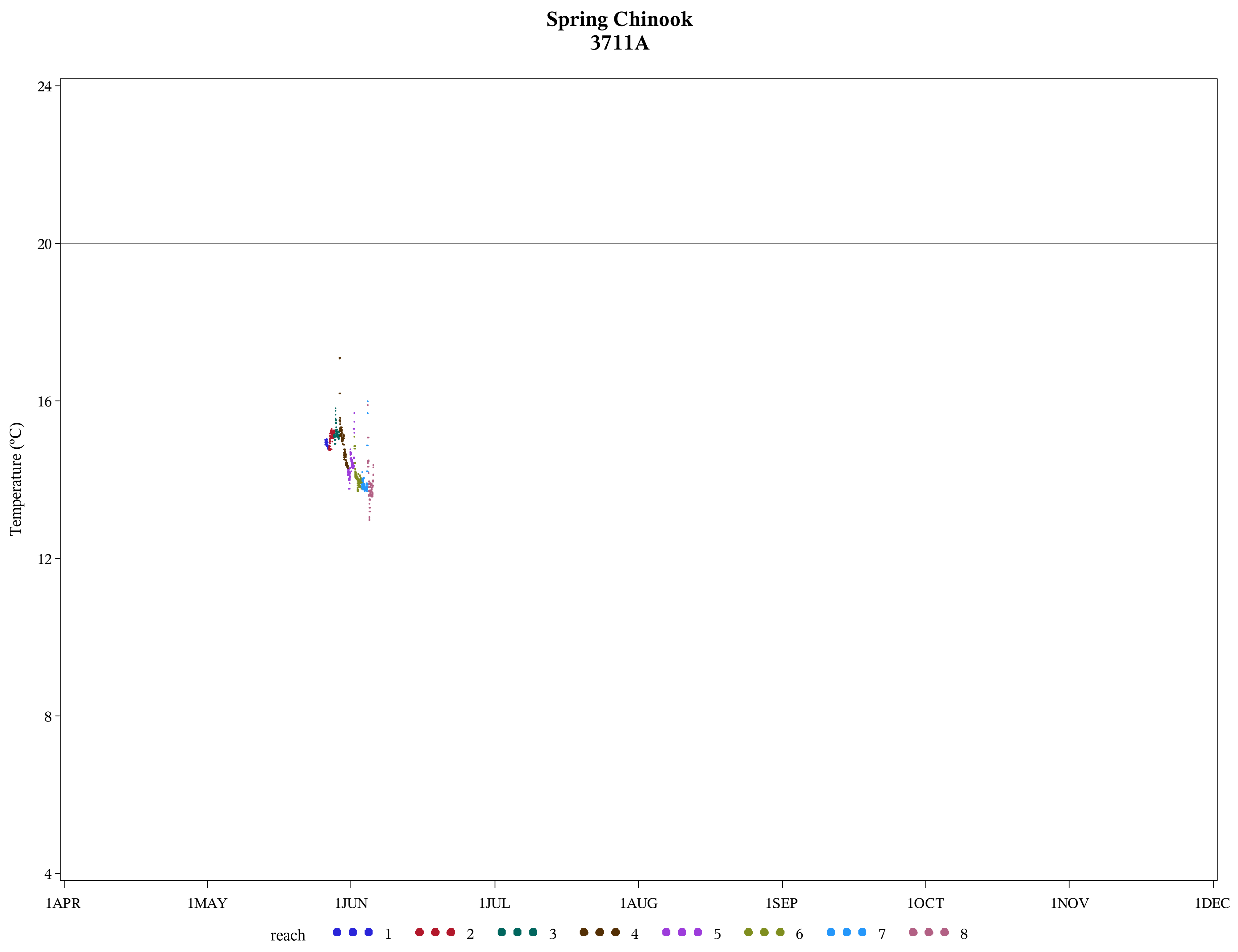
Spring Chinook
3711A
Temperature (ºC)
24
20
16
12
8
4
1APR
1MAY
1JUN
1JUL
1AUG
1SEP
1OCT
1NOV
1DEC
reach
1
2
3
4
5
6
7
8

Spring Chinook
3758A

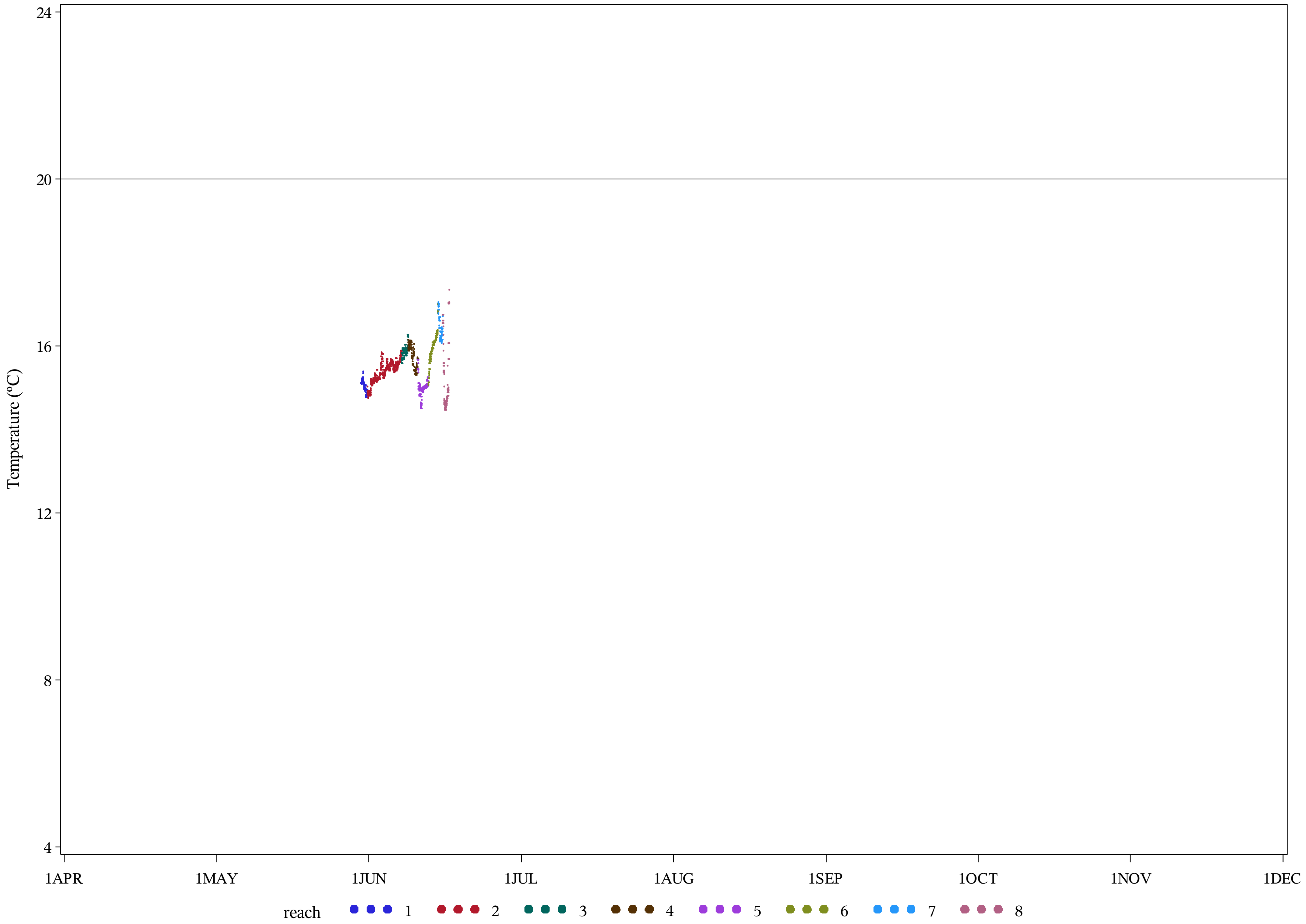

Spring Chinook
3761A

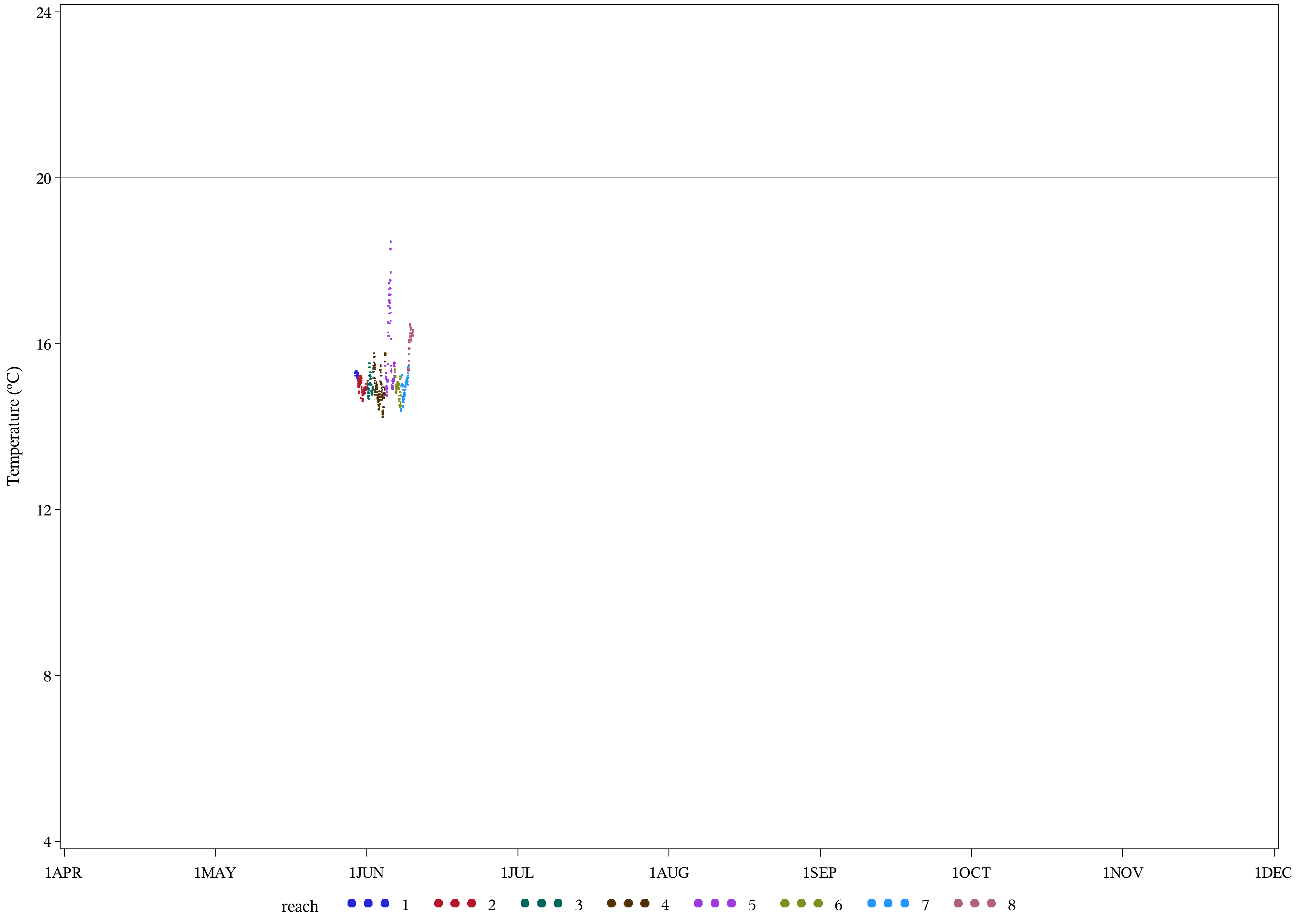

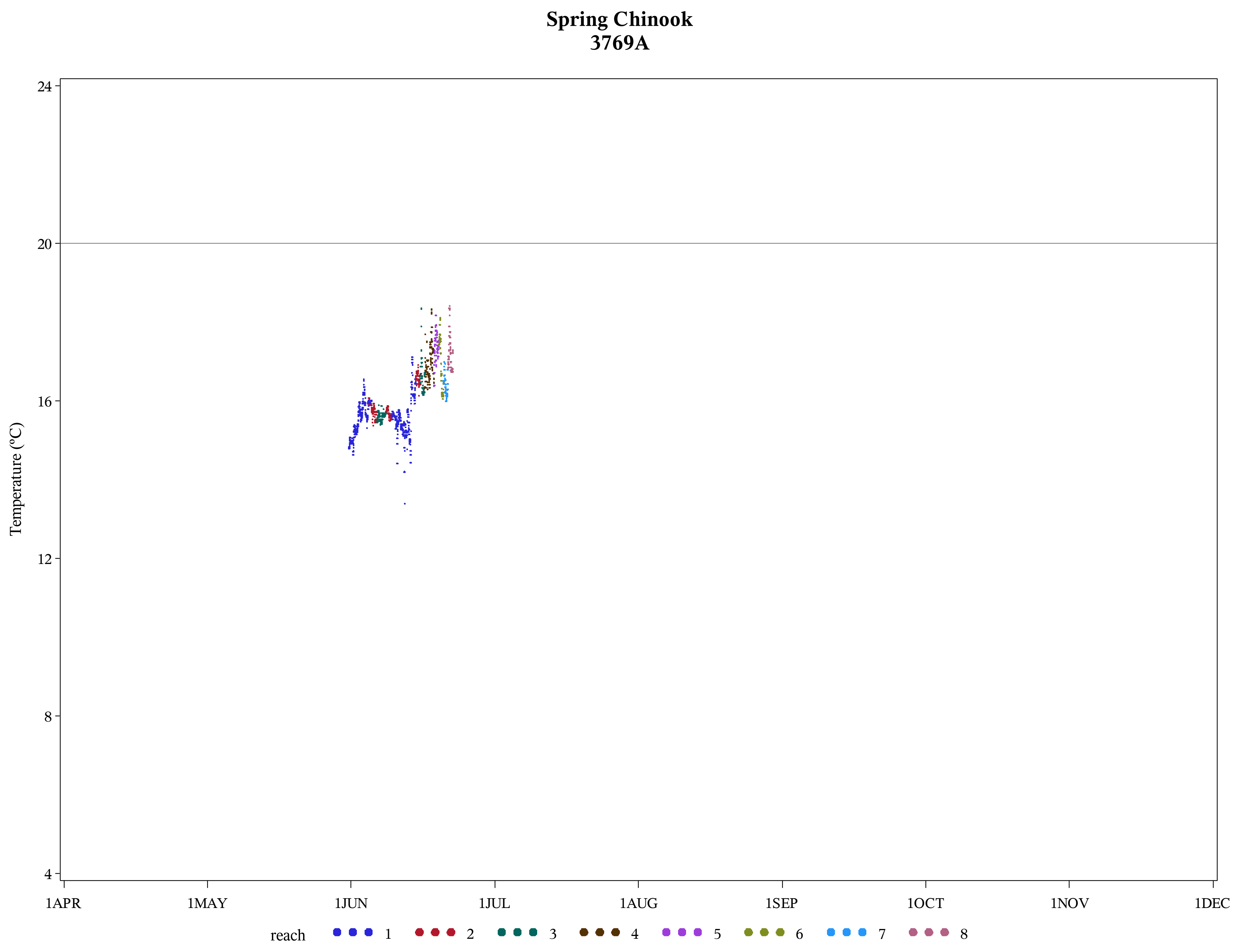

Spring Chinook
3769A
Temperature (°C)
24
20
16
12
8
4
1APR
1MAY
1JUN
1JUL
1AUG
1SEP
1OCT
1NOV
1DEC
reach
1
2
3
4
5
6
7
8

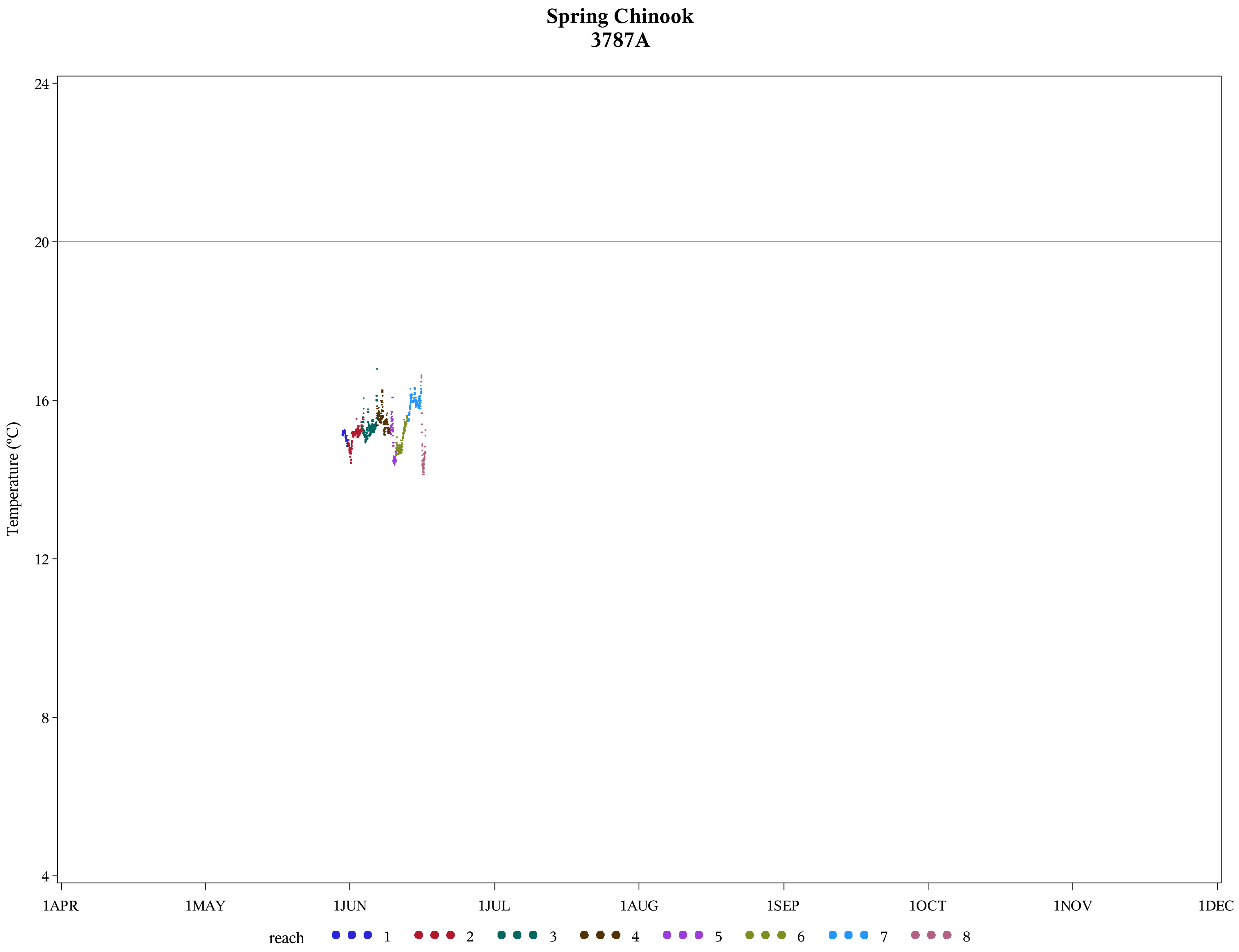
Spring Chinook
3787A
Temperature (°C)
24
20
16
12
8
4
1APR
1MAY
1JUN
1JUL
1AUG
1SEP
1OCT
1NOV
1DEC
reach
1
2
3
4
5
6
7
8

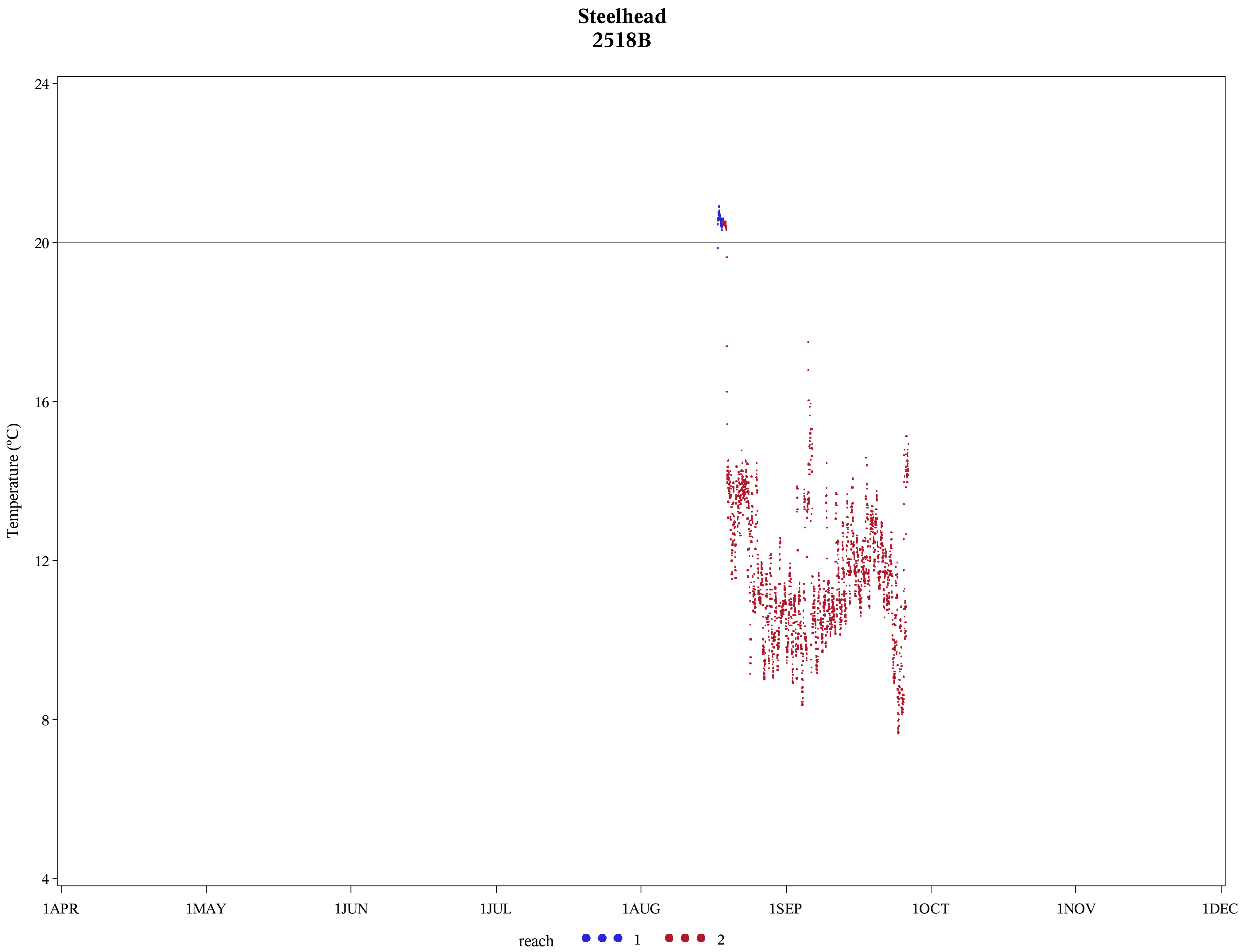
Steelhead
2518B
Temperature (°C)
24
20
16
12
8
4
1APR
1MAY
1JUN
1JUL
1AUG
1SEP
1OCT
1NOV
1DEC
reach
1
2

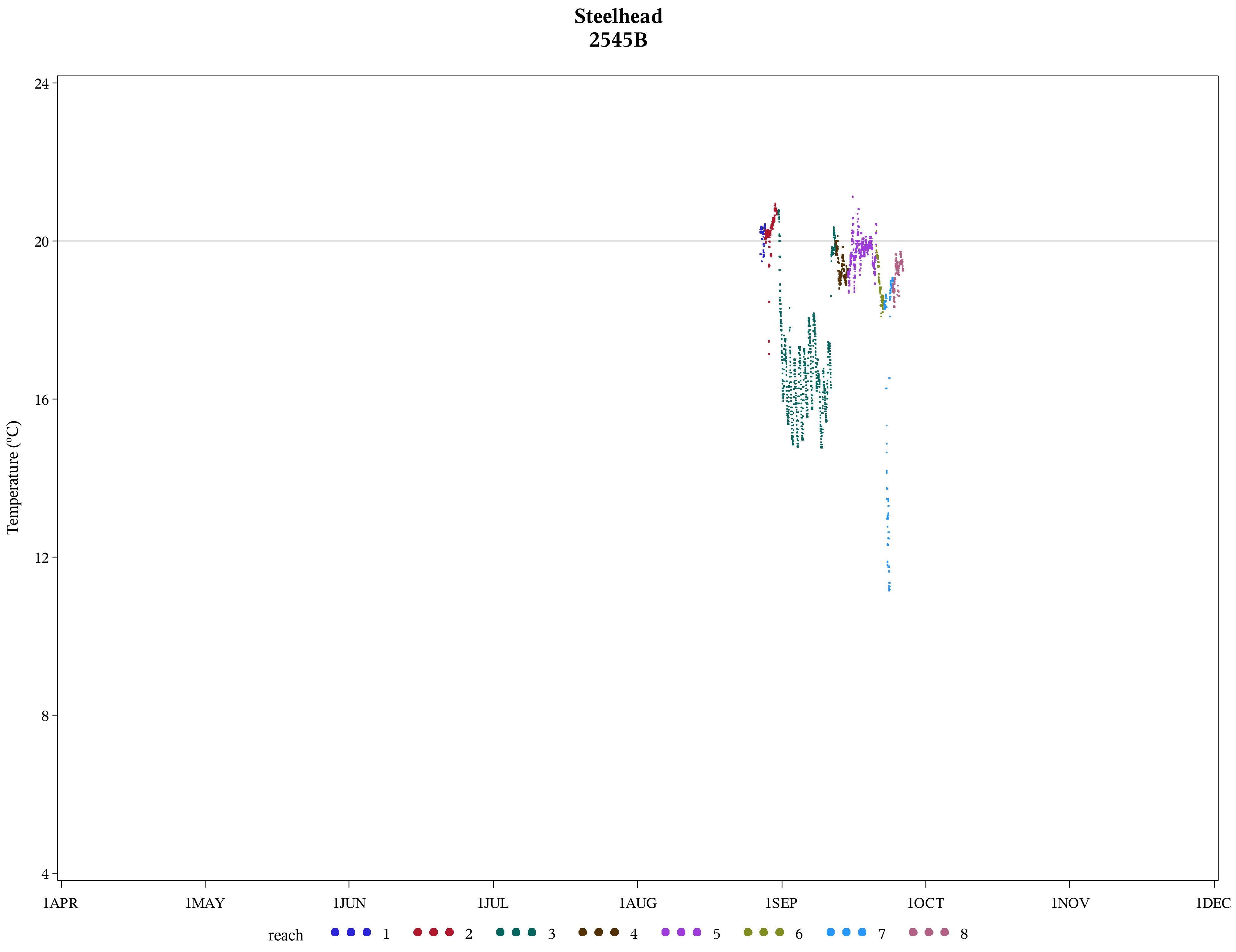

Steelhead
2545B
Temperature (°C)
24
20
16
12
8
4
1APR
1MAY
1JUN
1JUL
1AUG
1SEP
1OCT
1NOV
1DEC
reach
1
2
3
4
5
6
7
8

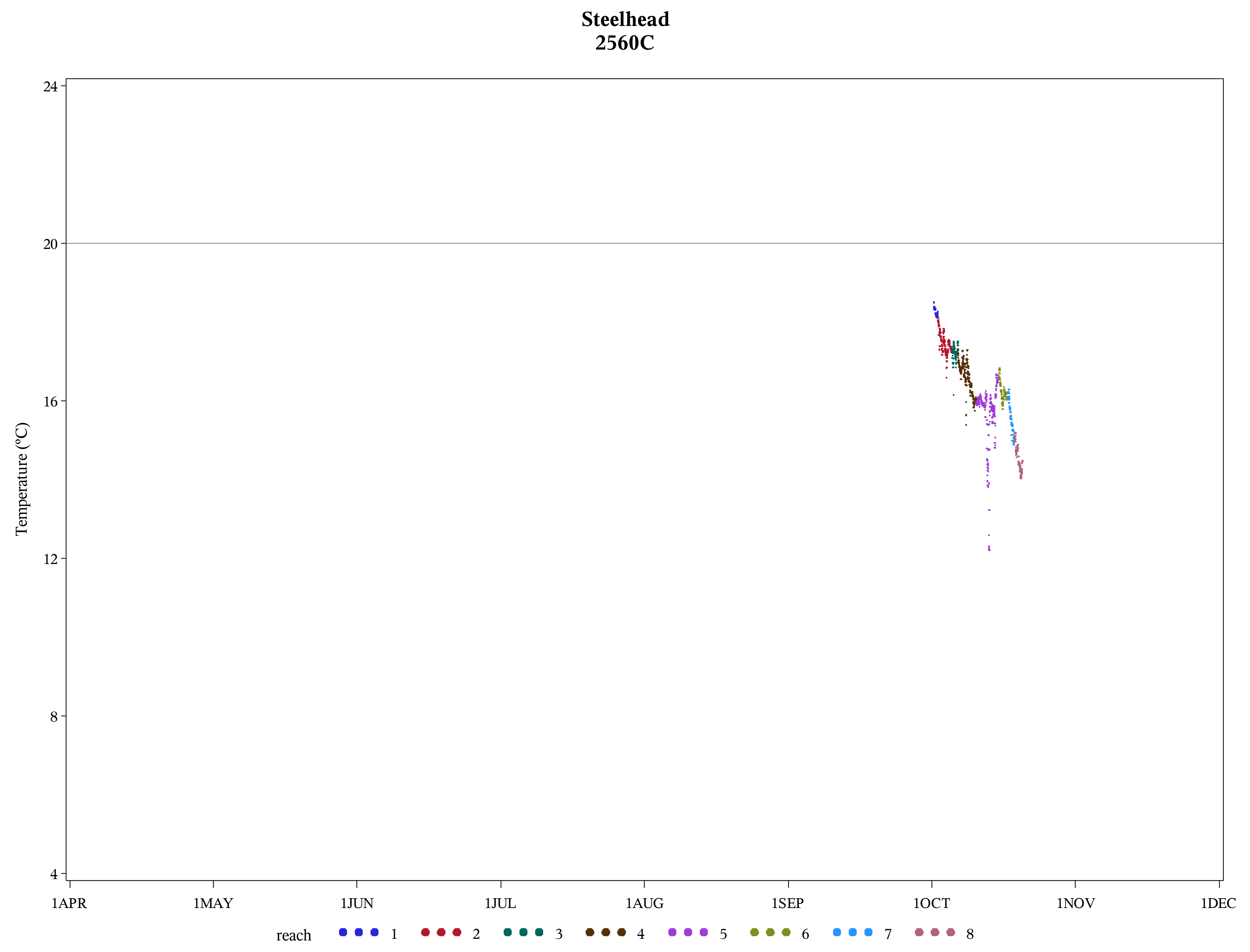

Steelhead
2560C
Temperature (°C)
24
20
16
12
8
4
1APR
1MAY
1JUN
1JUL
1AUG
1SEP
1OCT
1NOV
1DEC
reach
1
2
3
4
5
6
7
8

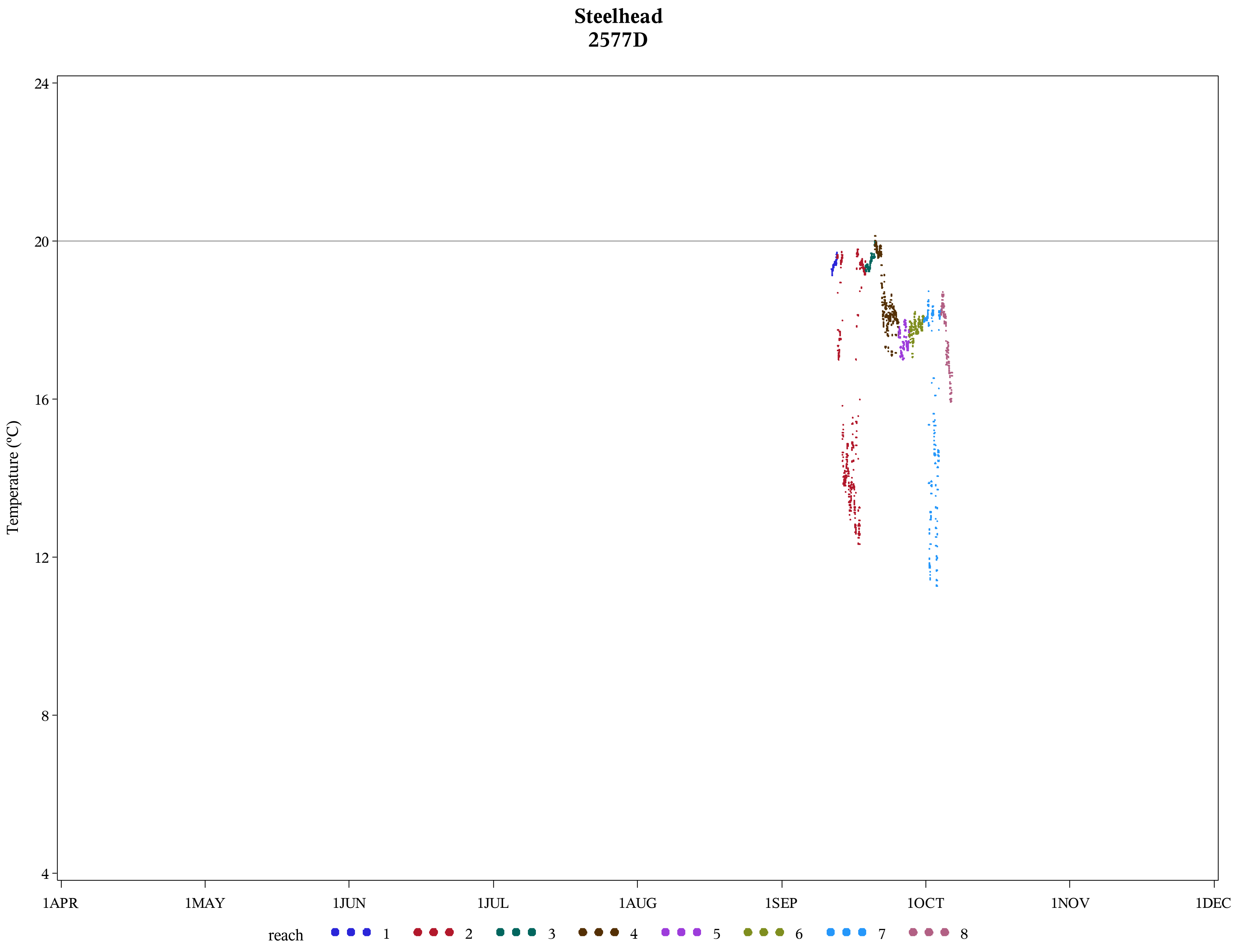

Steelhead
2577D
Temperature (°C)
24
20
16
12
8
4
1APR
1MAY
1JUN
1JUL
1AUG
1SEP
1OCT
1NOV
1DEC
reach
1
2
3
4
5
6
7
8

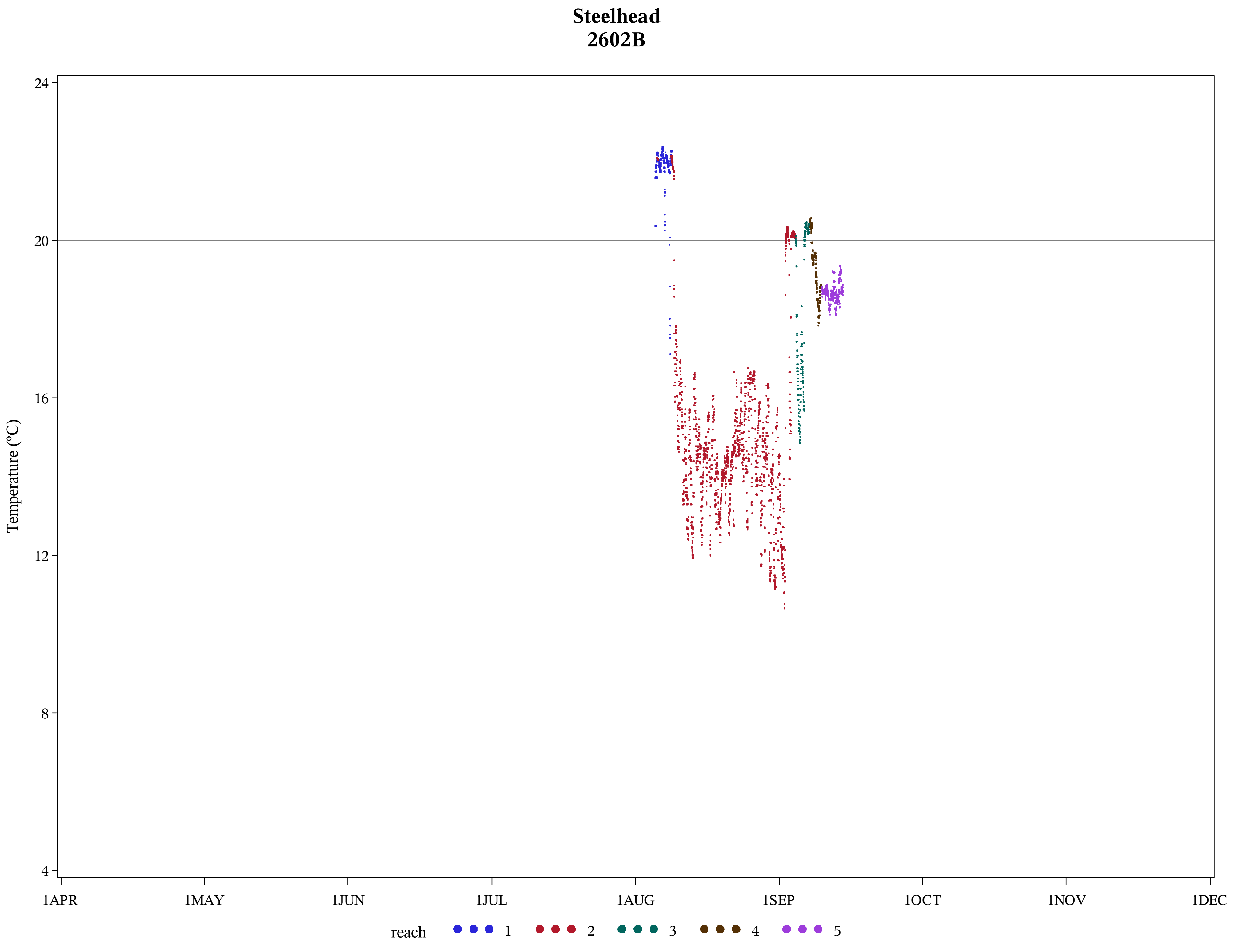

Steelhead
2602B
Temperature (°C)
24
20
16
12
8
4
1APR
1MAY
1JUN
1JUL
1AUG
1SEP
1OCT
1NOV
1DEC
reach
1
2
3
4
5

Steelhead
2617B

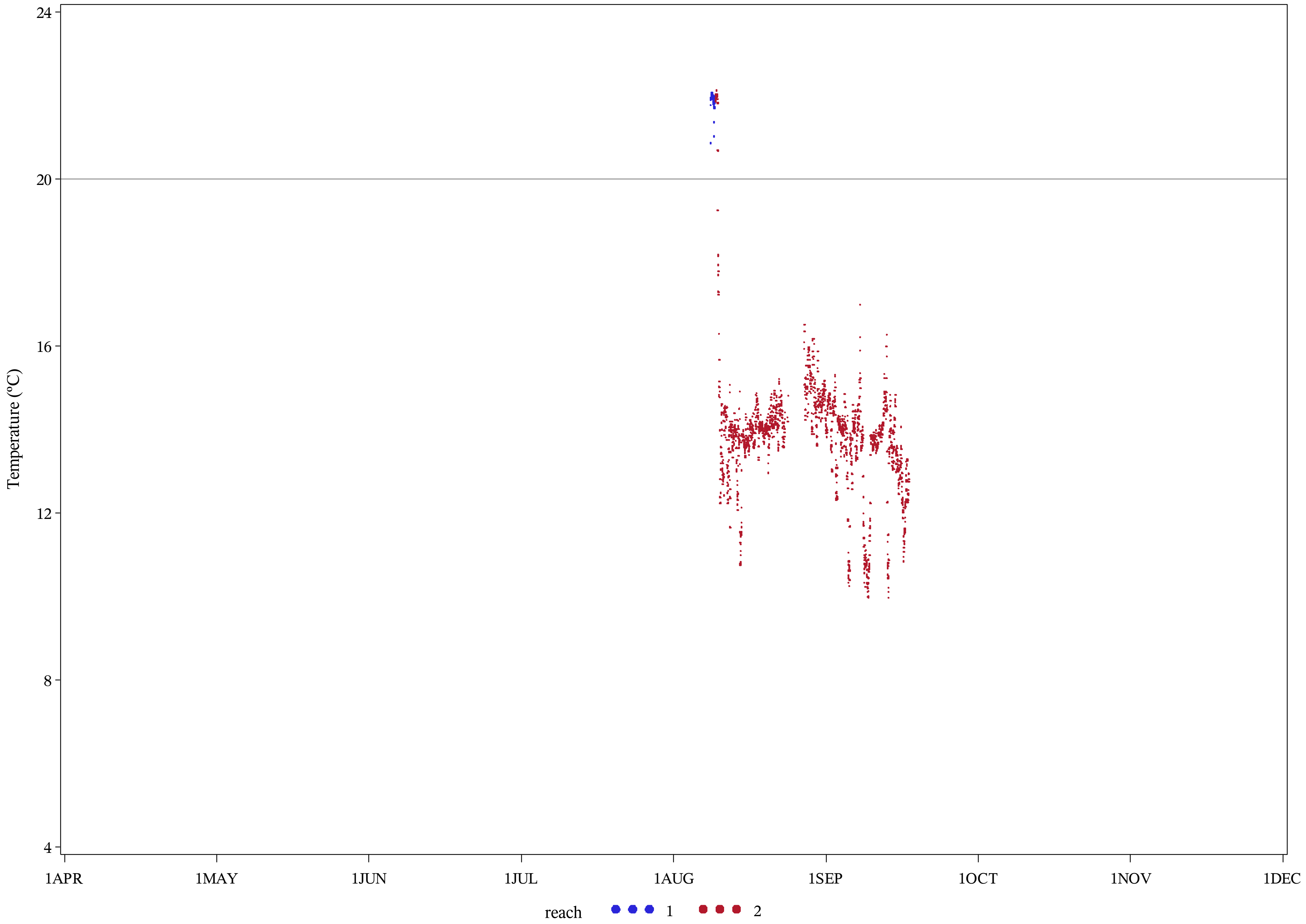

Steelhead
2621B

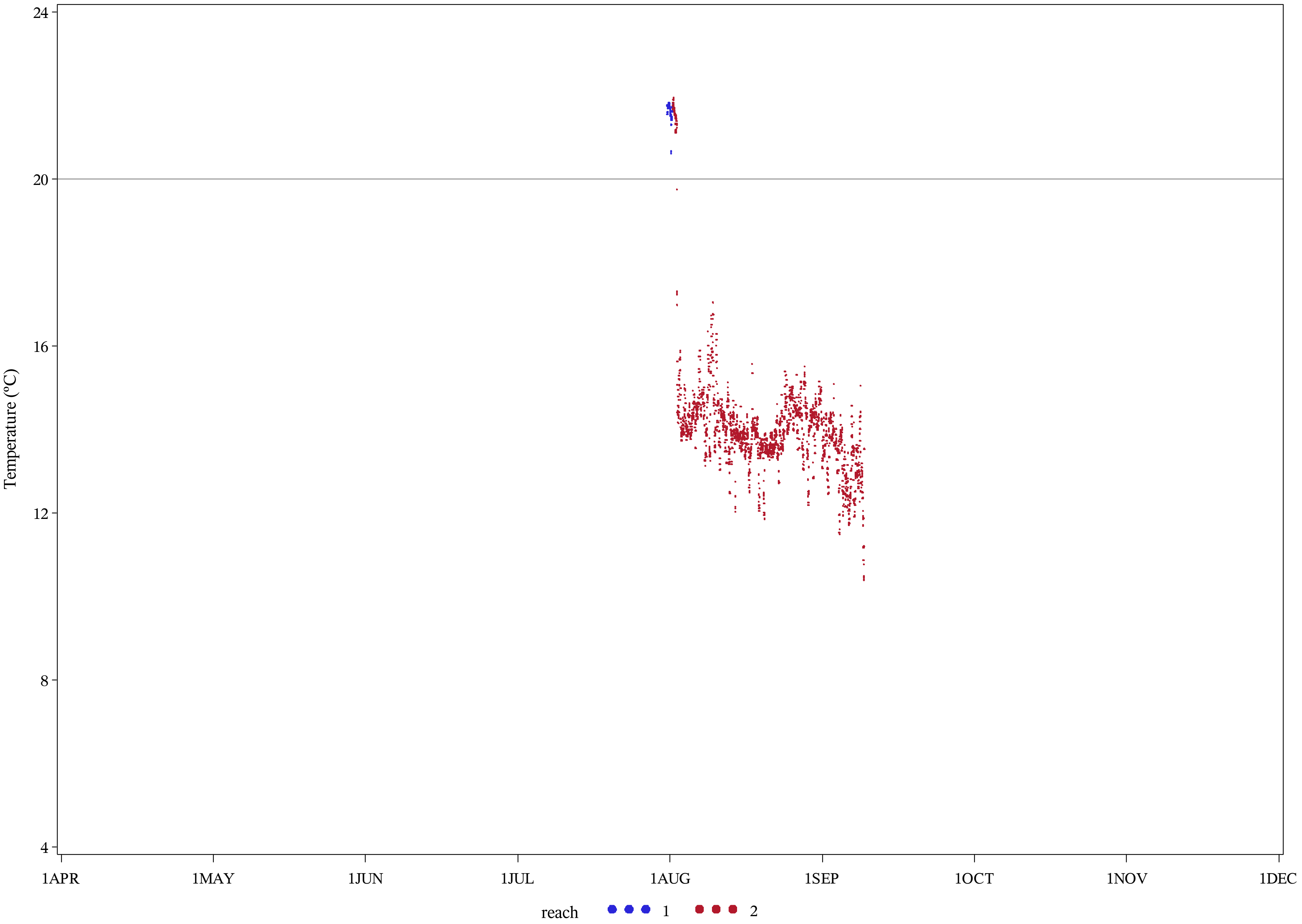

Steelhead
2640C

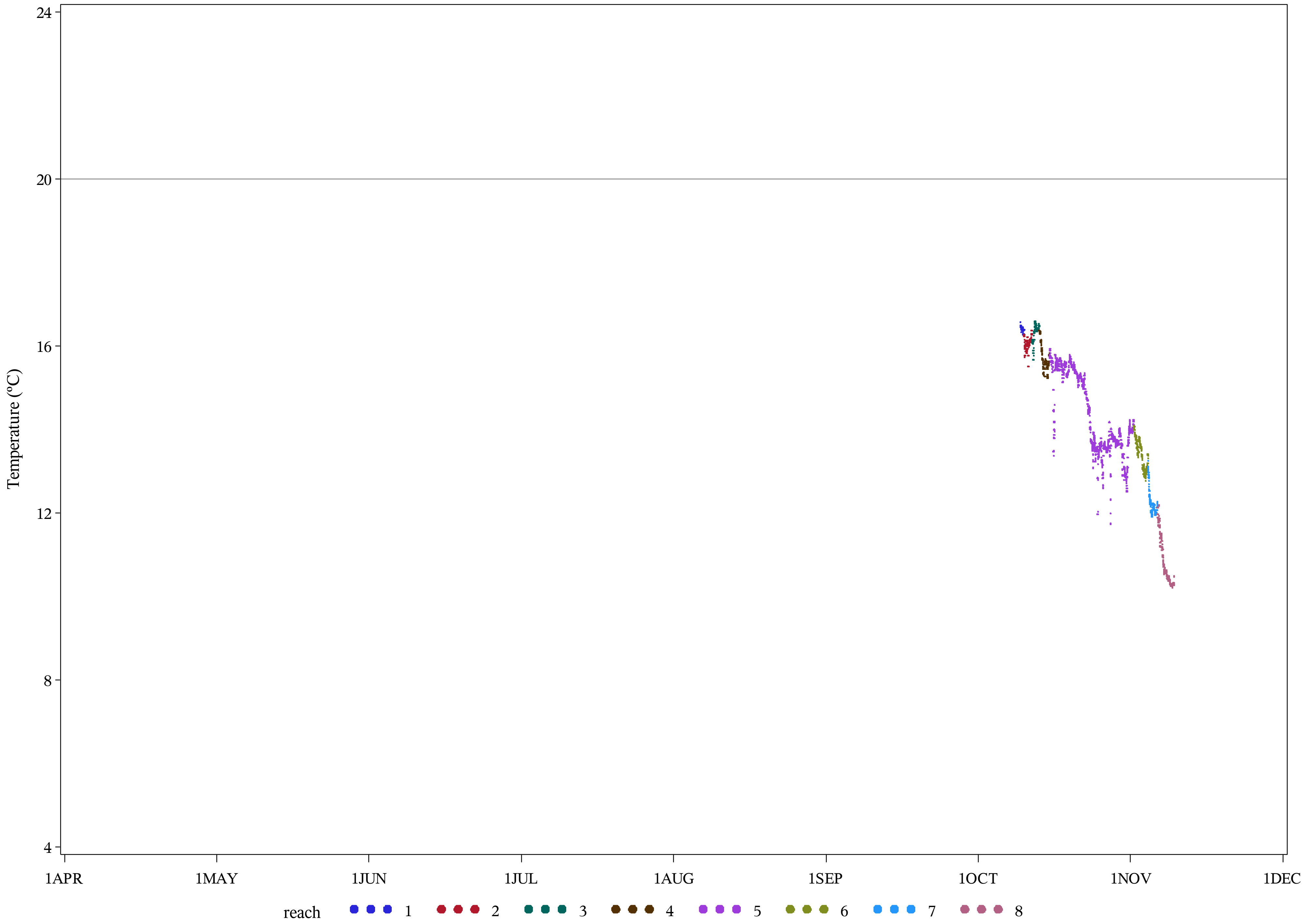

Steelhead
2645B

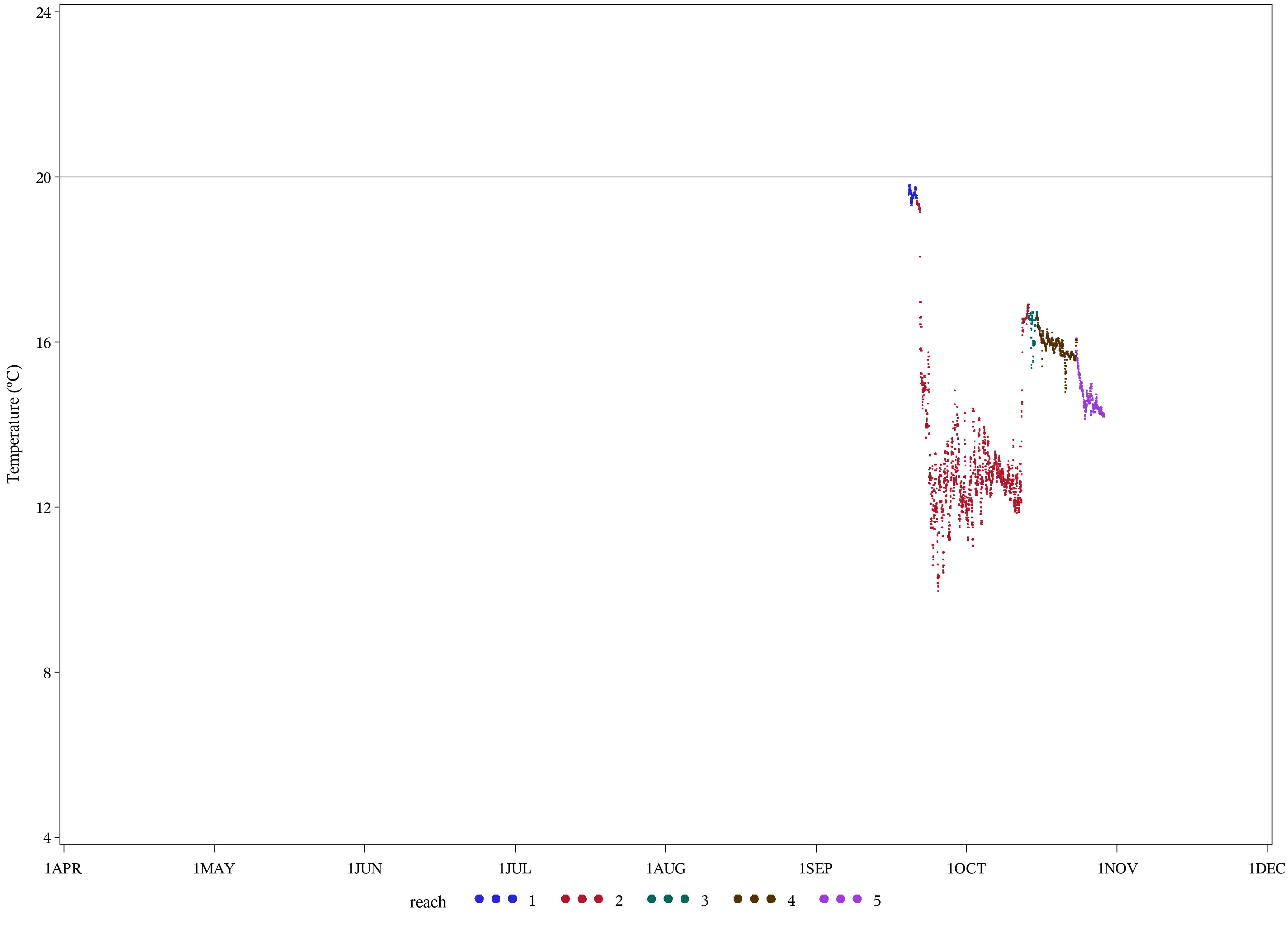

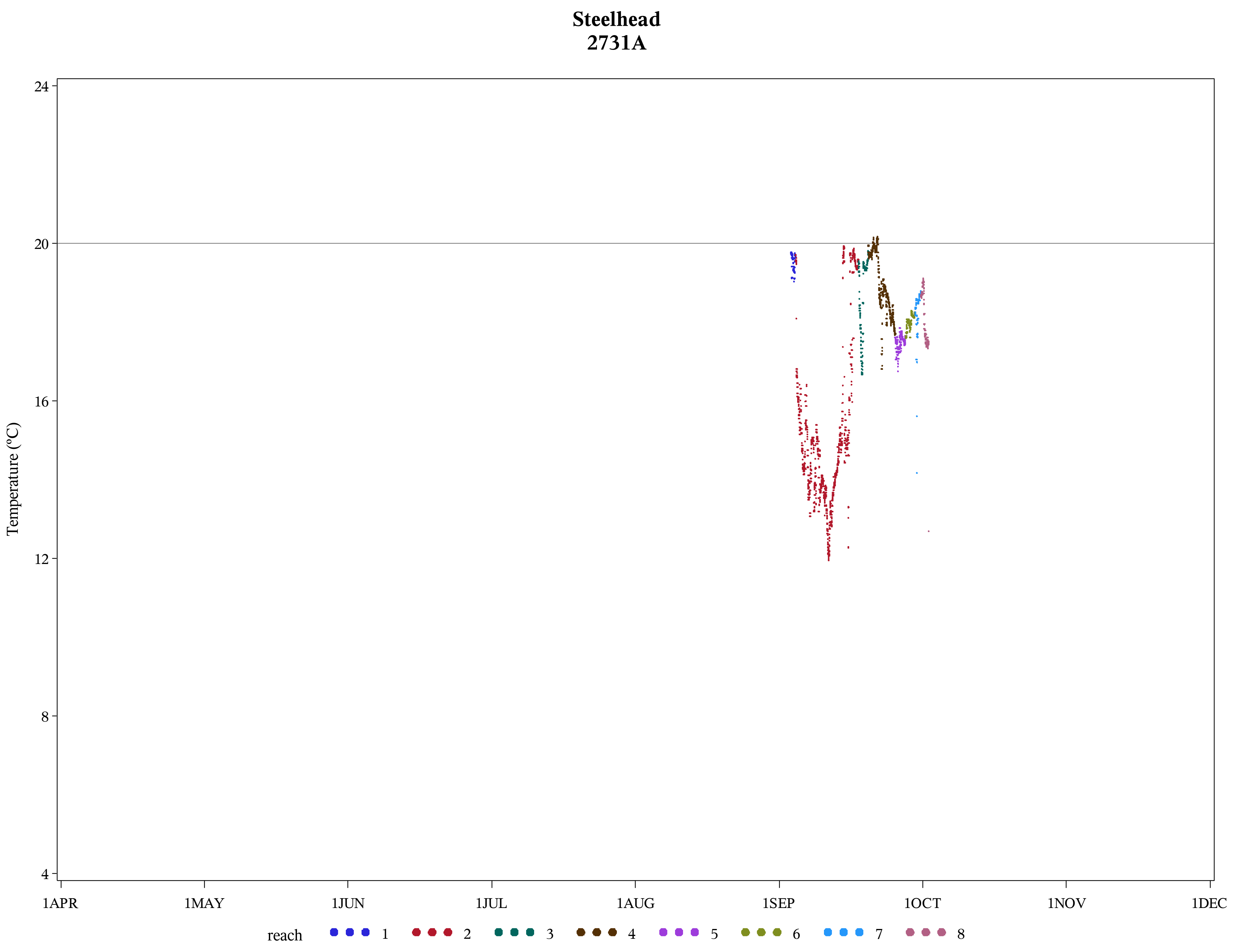
Steelhead
2731A
Temperature (ºC)
24
20
16
12
8
4
1APR
1MAY
1JUN
1JUL
1AUG
1SEP
1OCT
1NOV
1DEC
reach
1
2
3
4
5
6
7
8

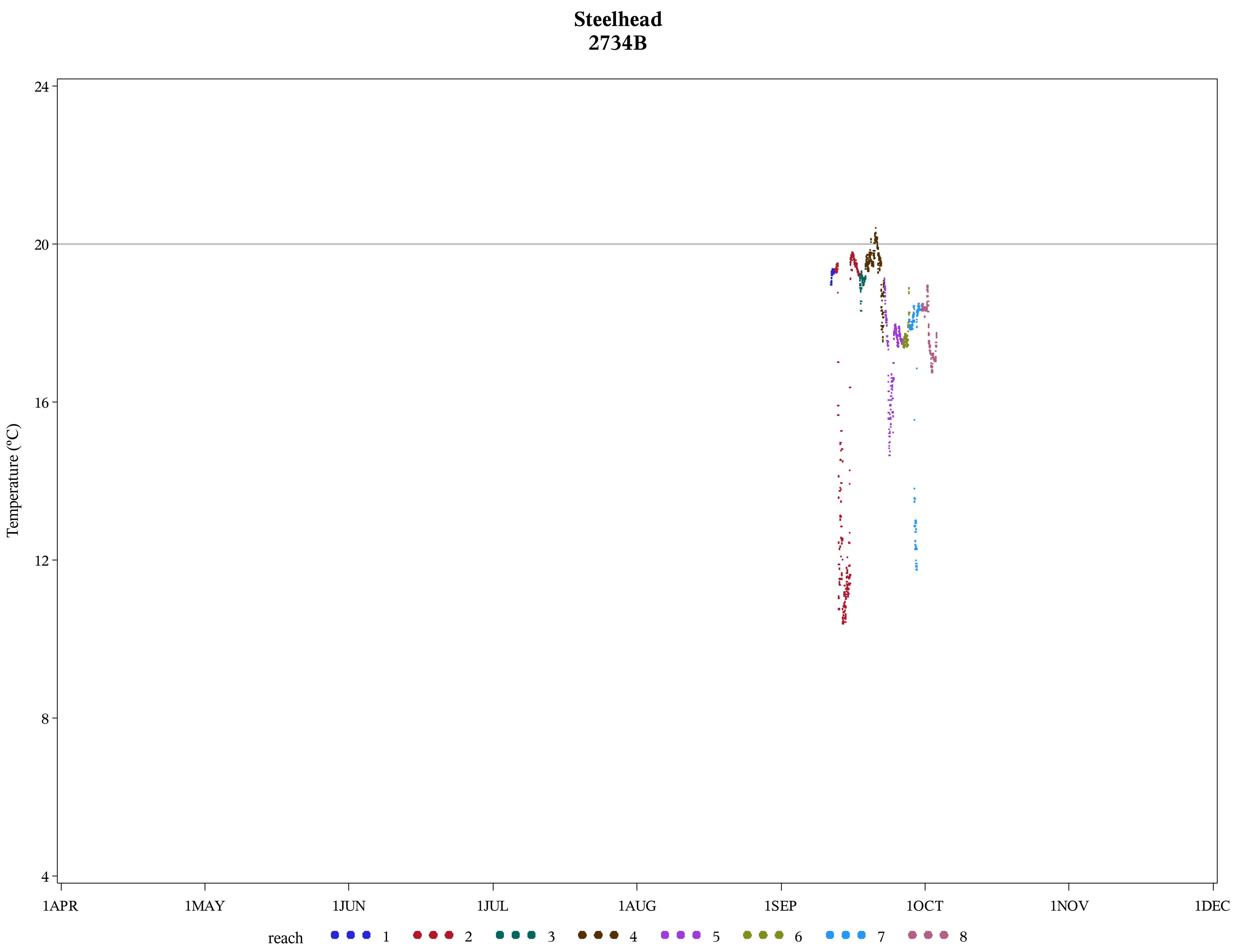
Steelhead
2734B
Temperature (°C)
24
20
16
12
8
4
1APR
1MAY
1JUN
1JUL
1AUG
1SEP
1OCT
1NOV
1DEC
reach
1
2
3
4
5
6
7
8

Steelhead
2758B

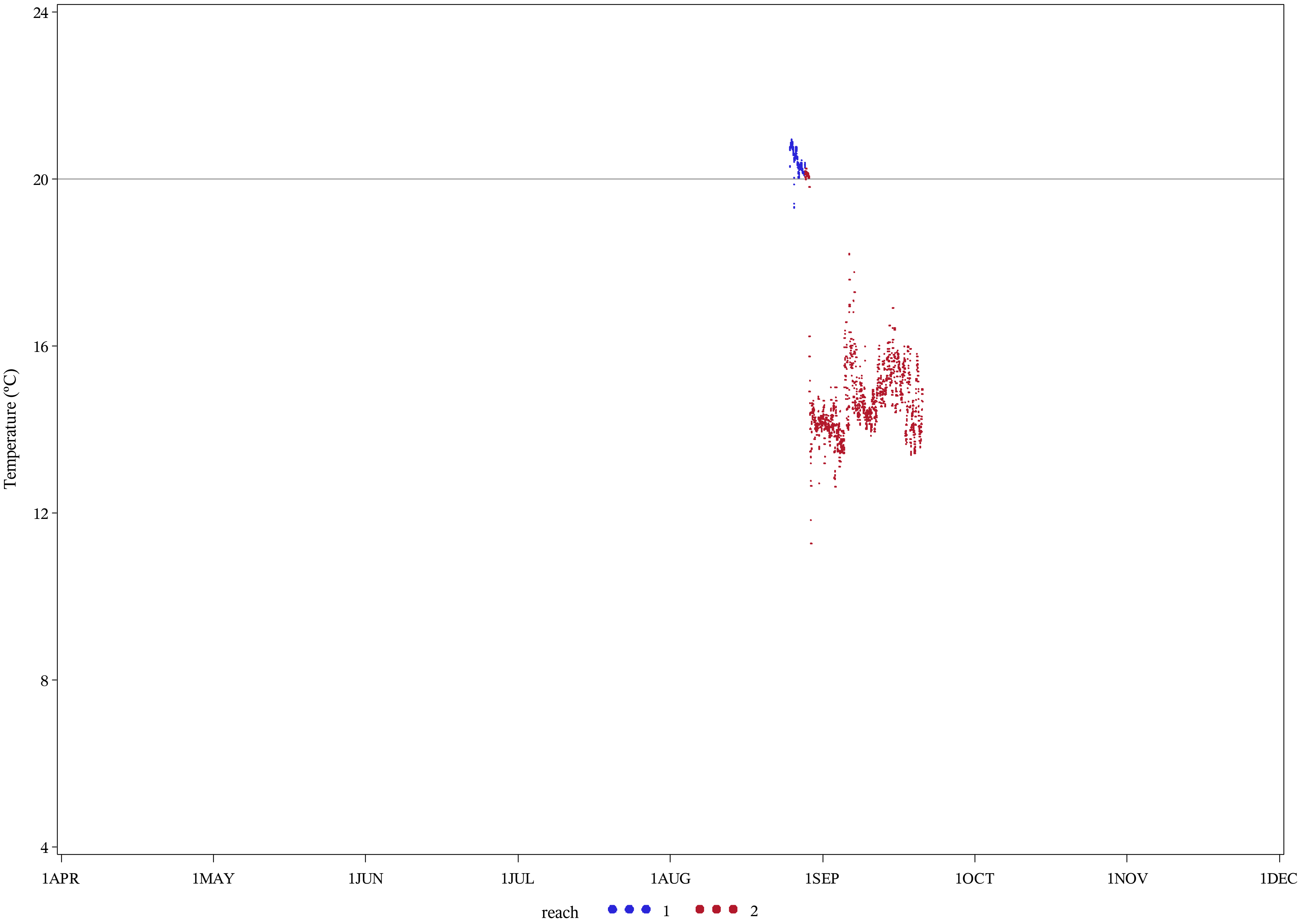

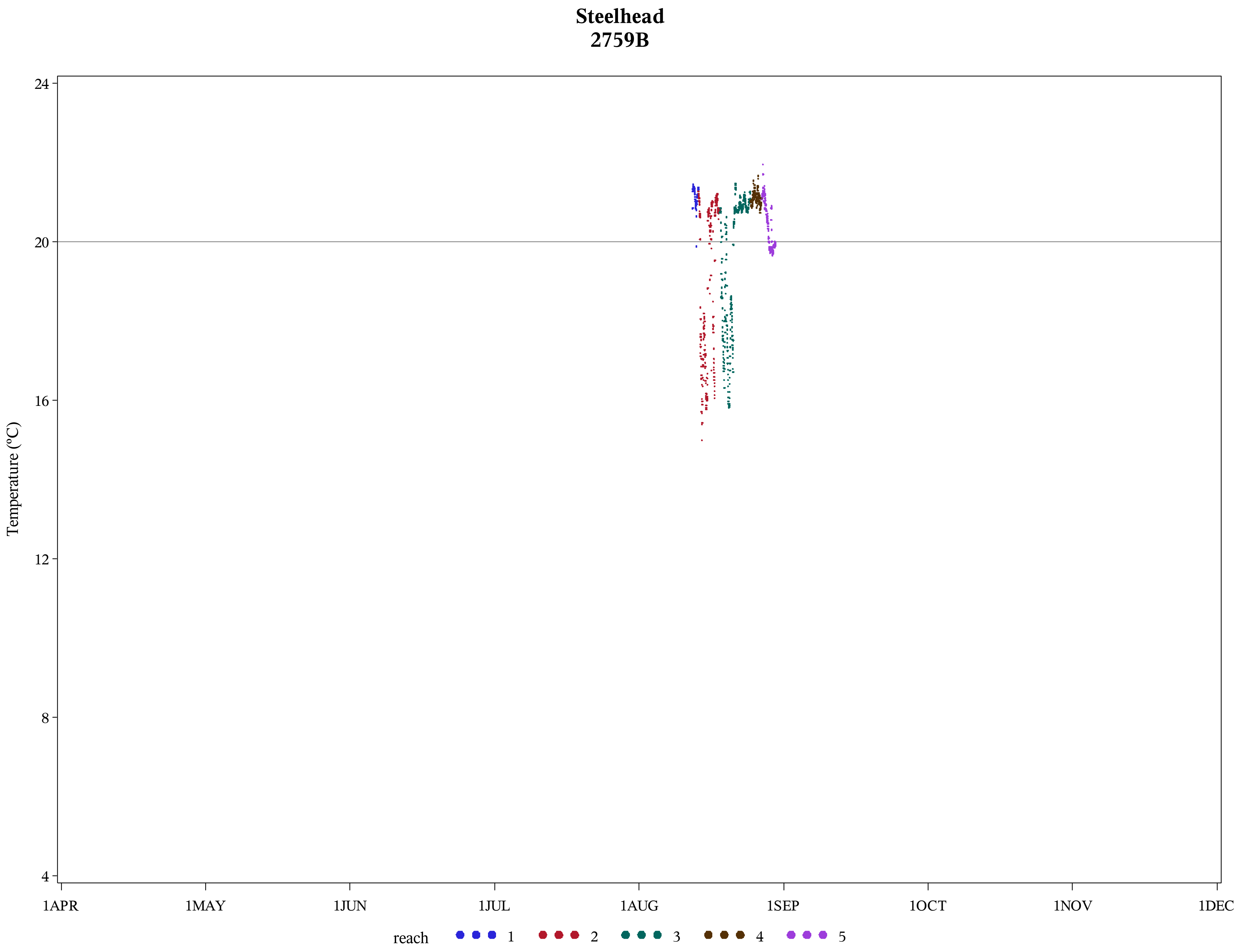

Steelhead
2759B
Temperature (°C)
24
20
16
12
8
4
1APR
1MAY
1JUN
1JUL
1AUG
1SEP
1OCT
1NOV
1DEC
reach
1
2
3
4
5

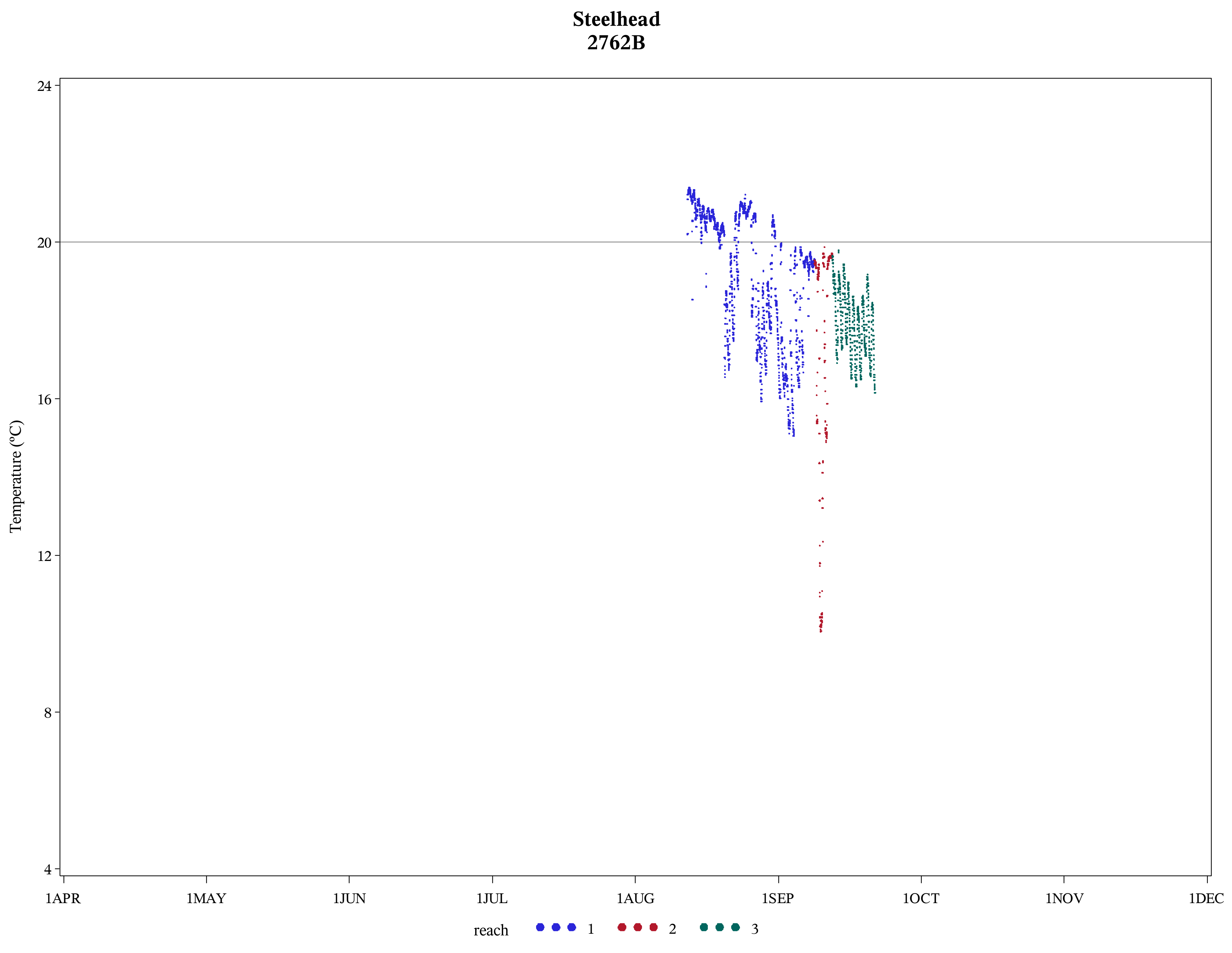

Steelhead
2762B
Temperature (°C)
24
20
16
12
8
4
1APR
1MAY
1JUN
1JUL
1AUG
1SEP
1OCT
1NOV
1DEC
reach
1
2
3

Steelhead
2787B

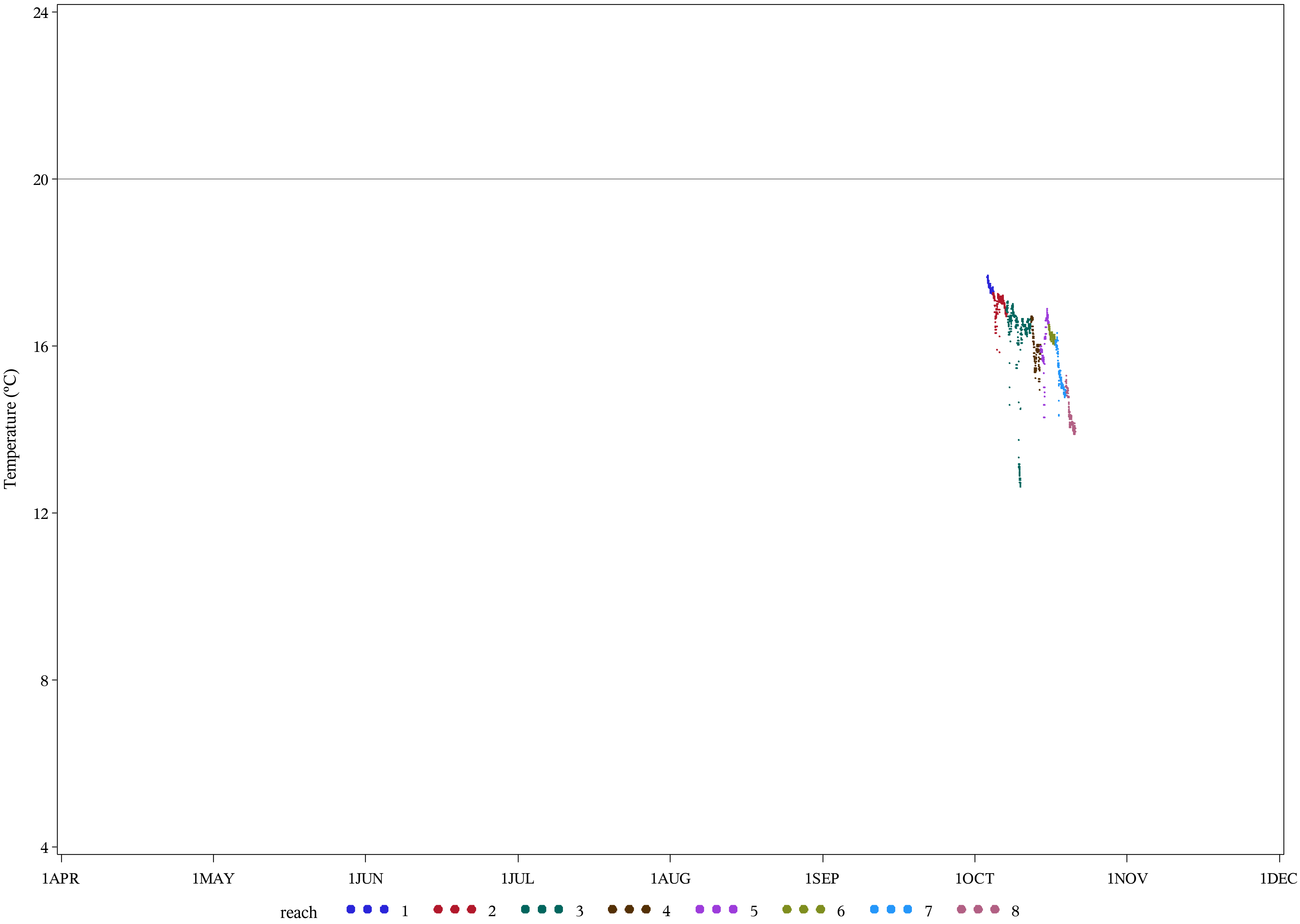

Steelhead
2797A

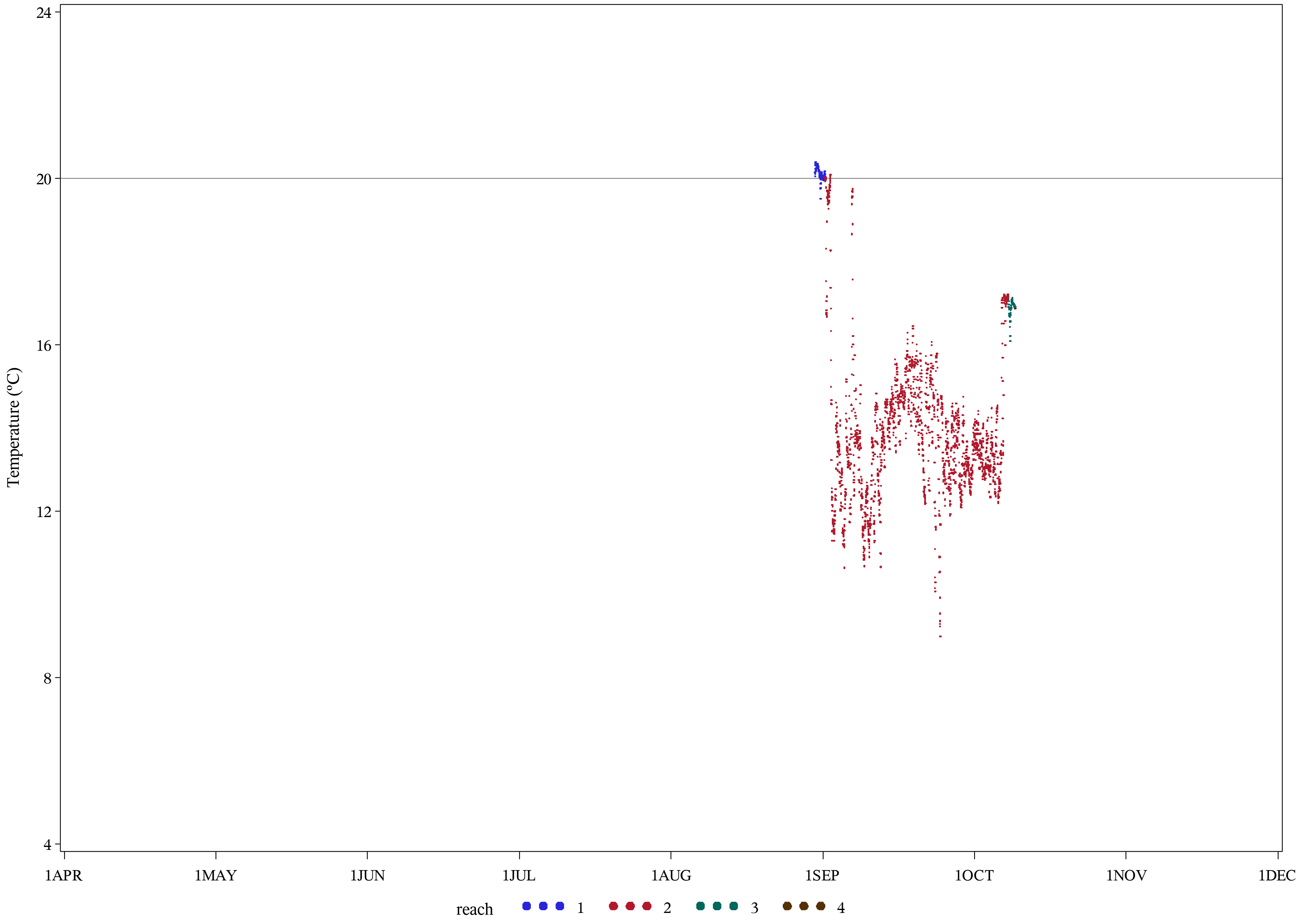

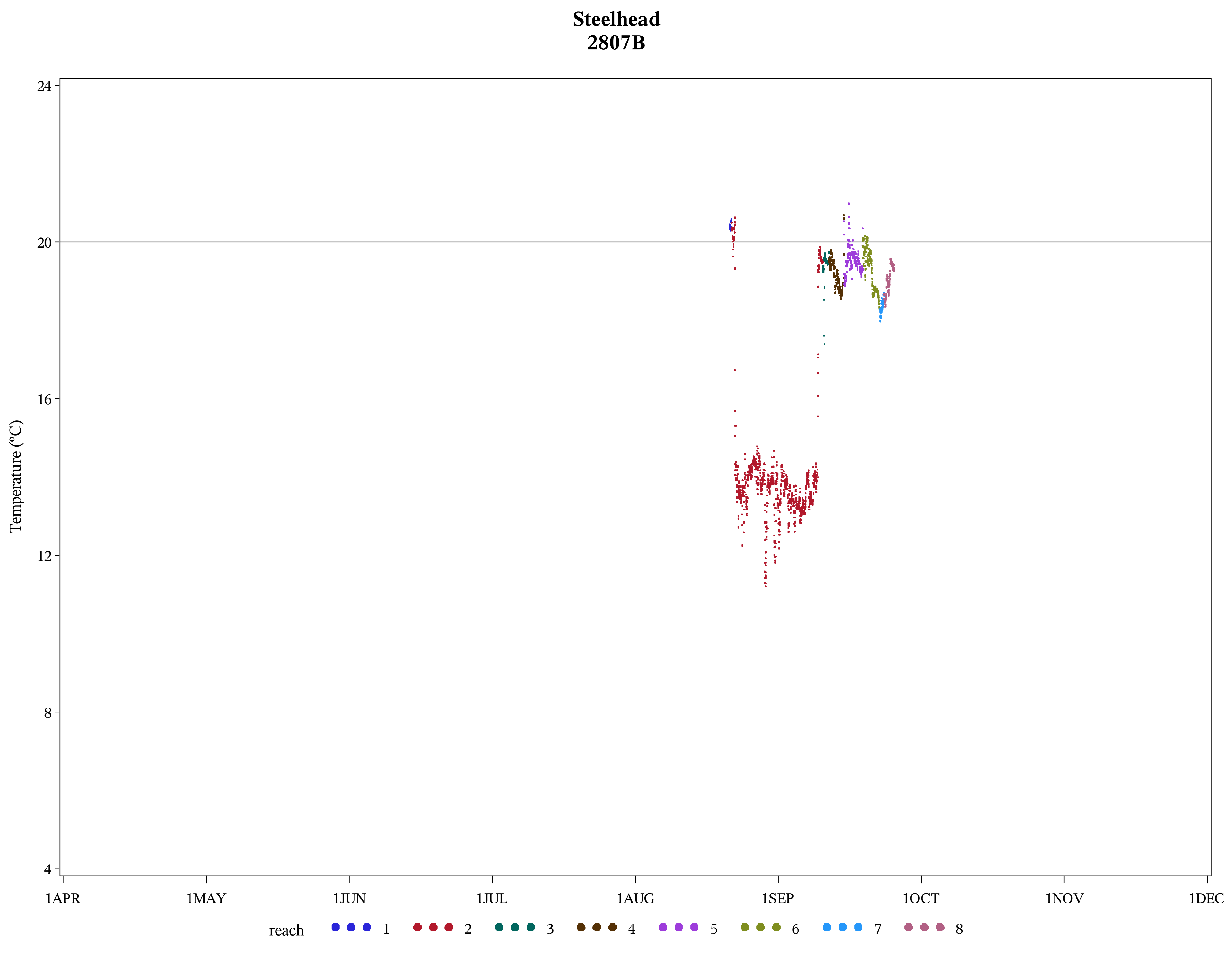

Steelhead
2807B
Temperature (°C)
24
20
16
12
8
4
1APR
1MAY
1JUN
1JUL
1AUG
1SEP
1OCT
1NOV
1DEC
reach
1
2
3
4
5
6
7
8

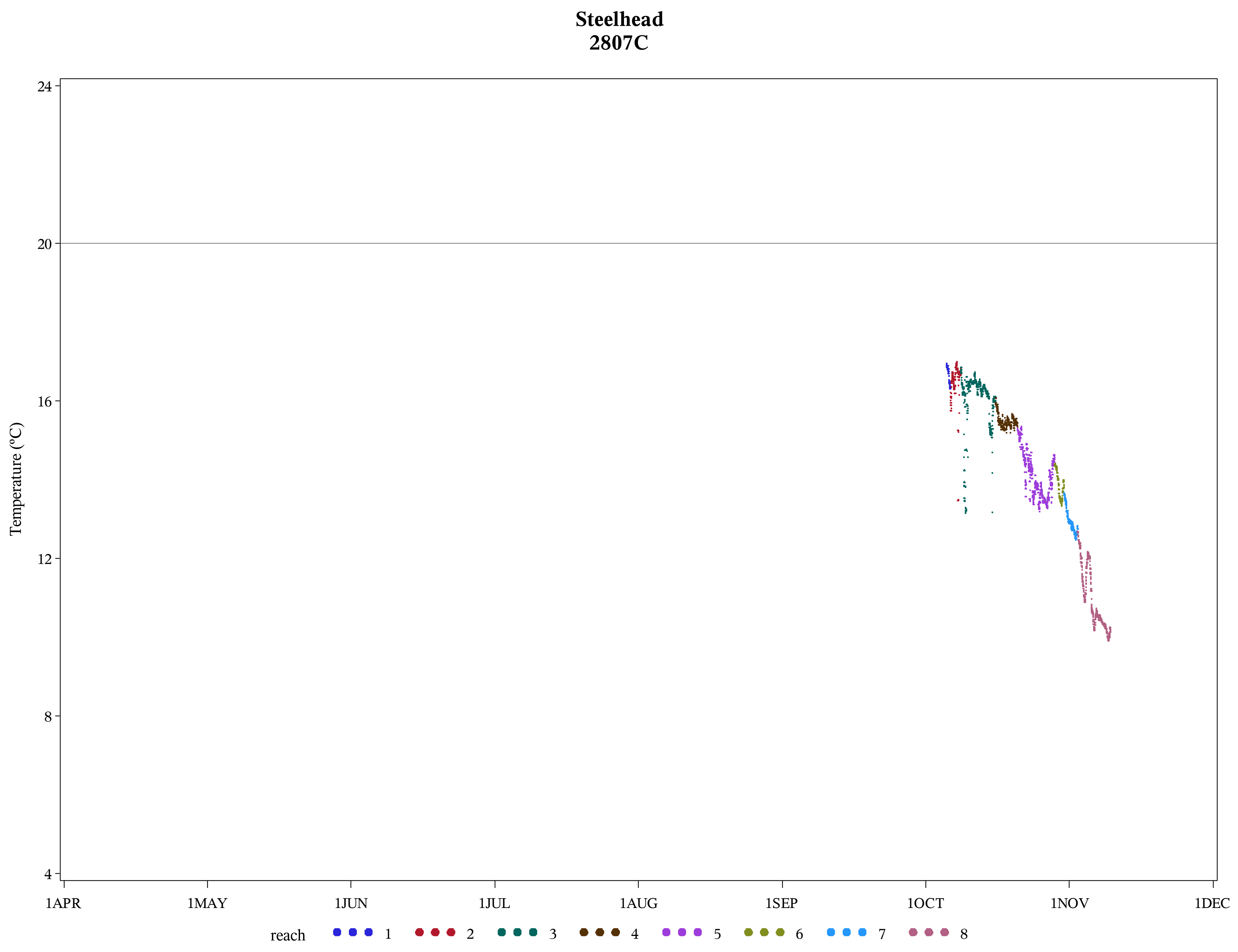
Steelhead
2807C
Temperature (°C)
24
20
16
12
8
4
1APR
1MAY
1JUN
1JUL
1AUG
1SEP
1OCT
1NOV
1DEC
reach
1
2
3
4
5
6
7
8

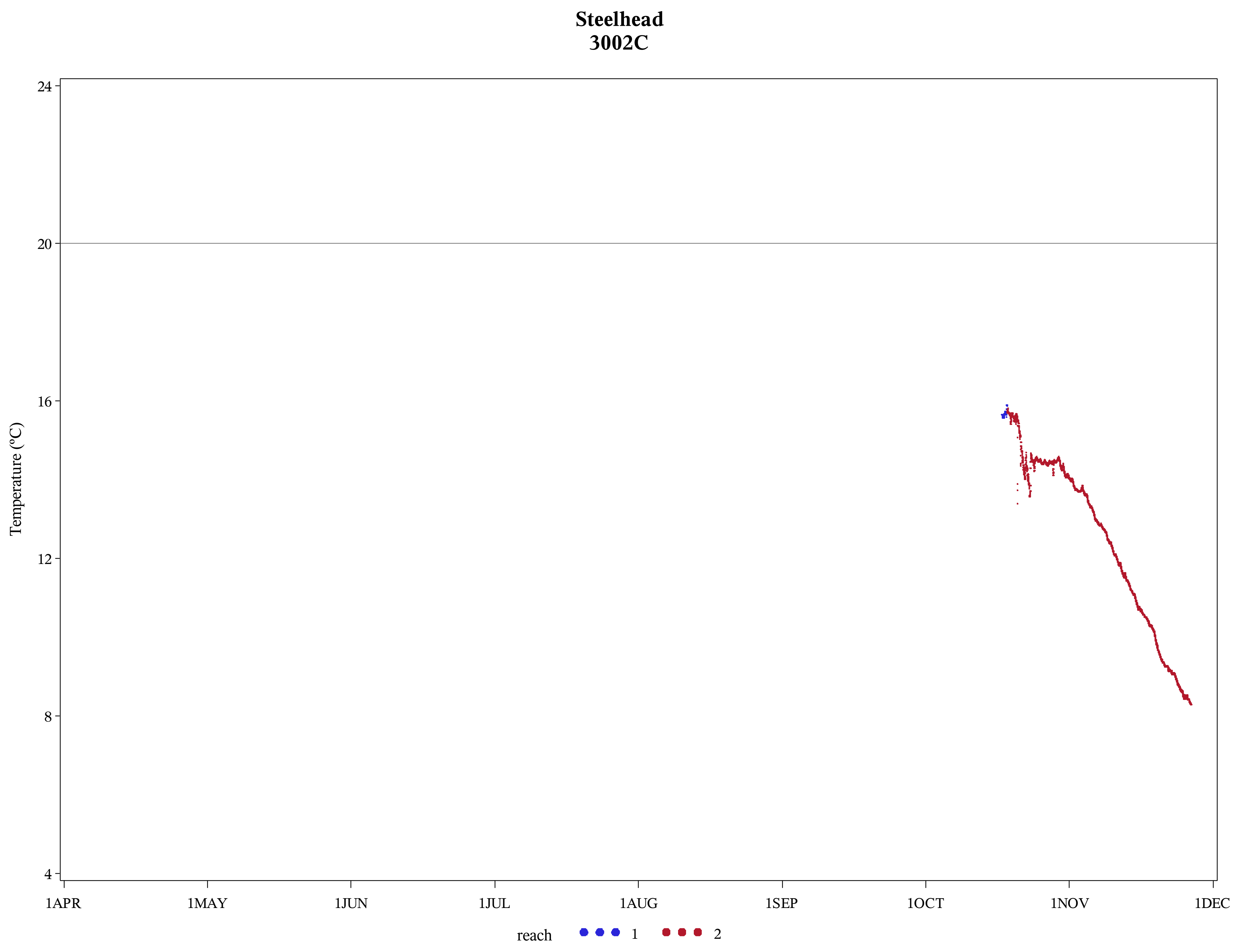
Steelhead
3002C
Temperature (°C)
24
20
16
12
8
4
1APR
1MAY
1JUN
1JUL
1AUG
1SEP
1OCT
1NOV
1DEC
reach
1
2

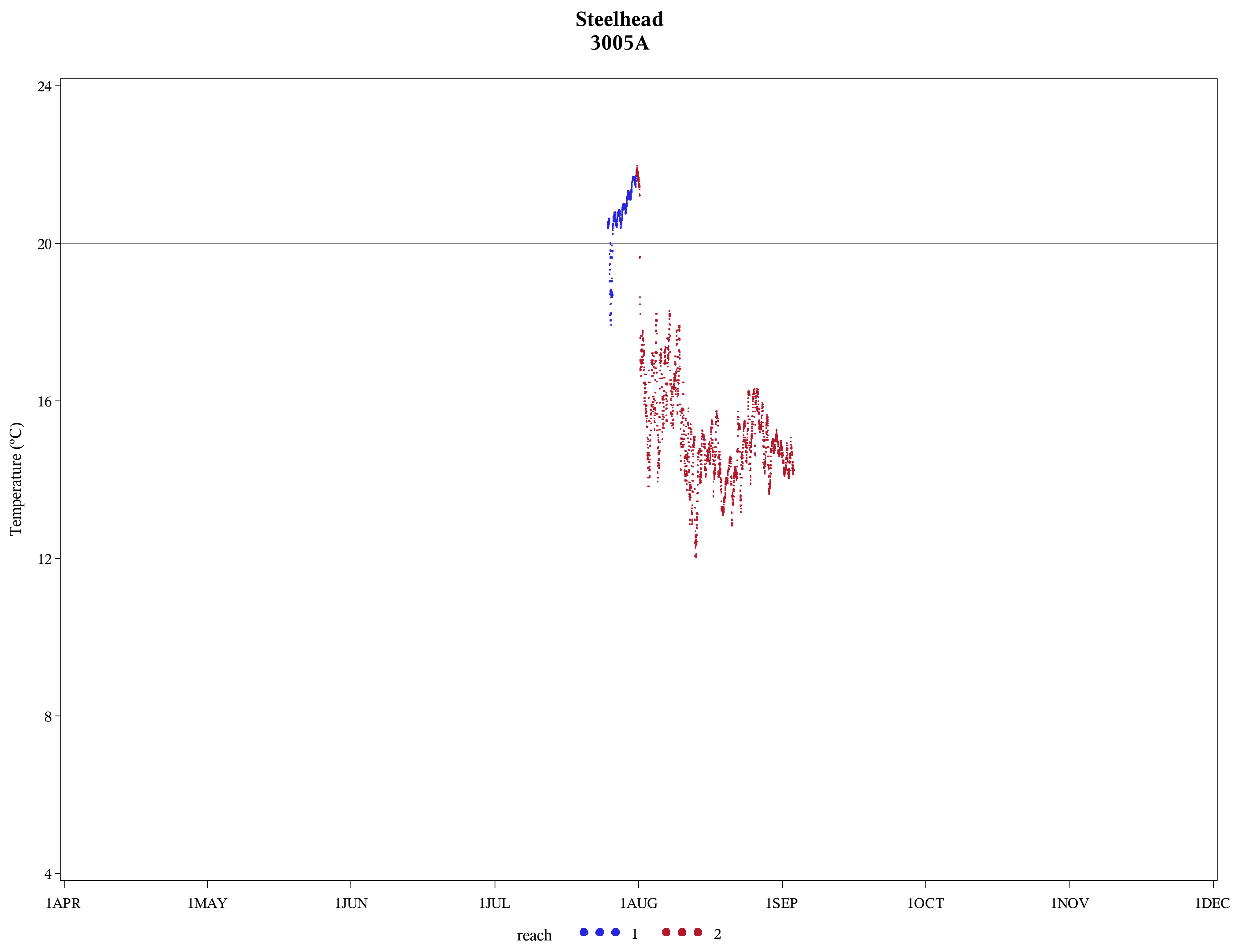

Steelhead
3005A
Temperature (°C)
24
20
16
12
8
4
1APR
1MAY
1JUN
1JUL
1AUG
1SEP
1OCT
1NOV
1DEC
reach
1
2

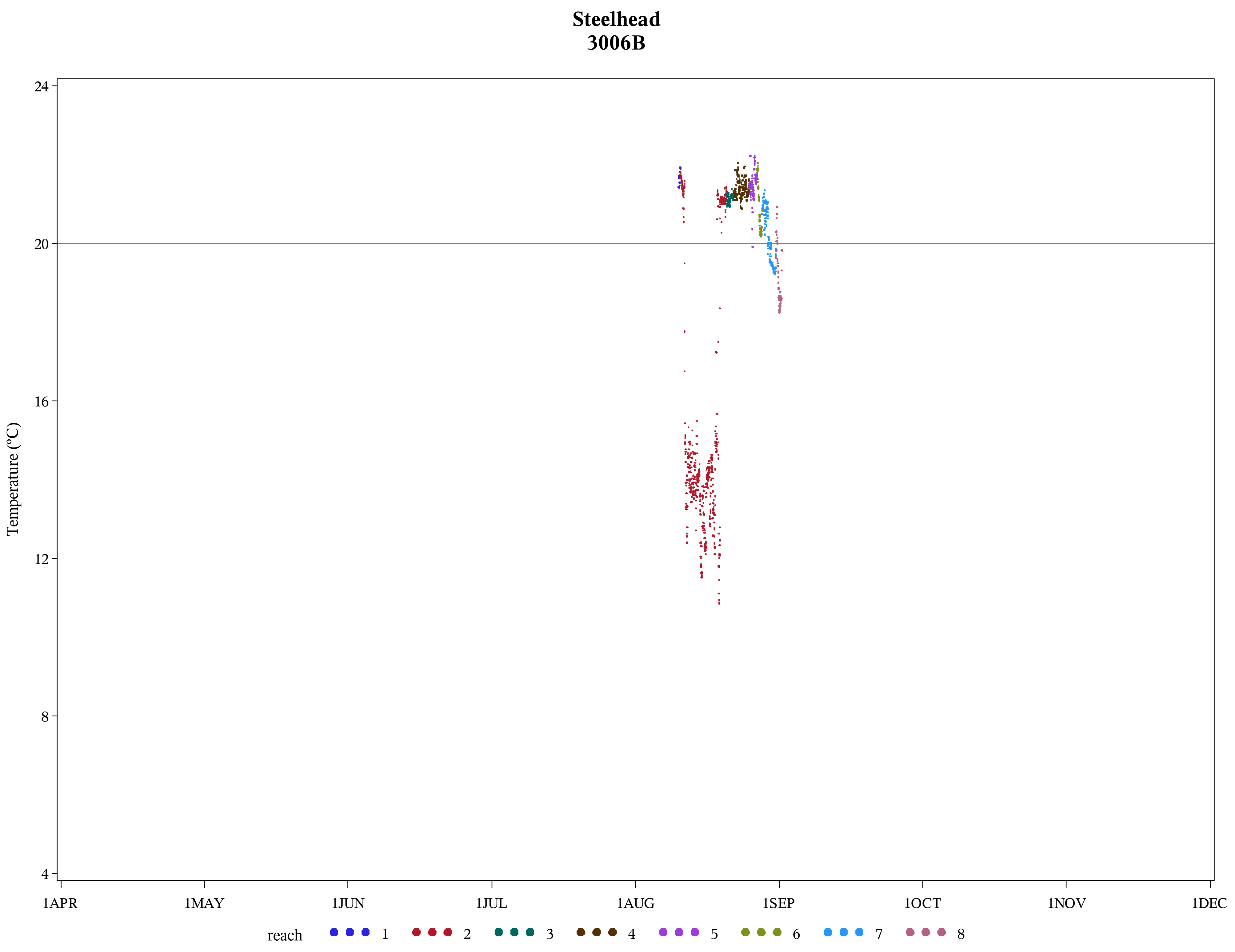
Steelhead
3006B
Temperature (°C)
24
20
16
12
8
4
1APR
1MAY
1JUN
1JUL
1AUG
1SEP
1OCT
1NOV
1DEC
reach
1
2
3
4
5
6
7
8

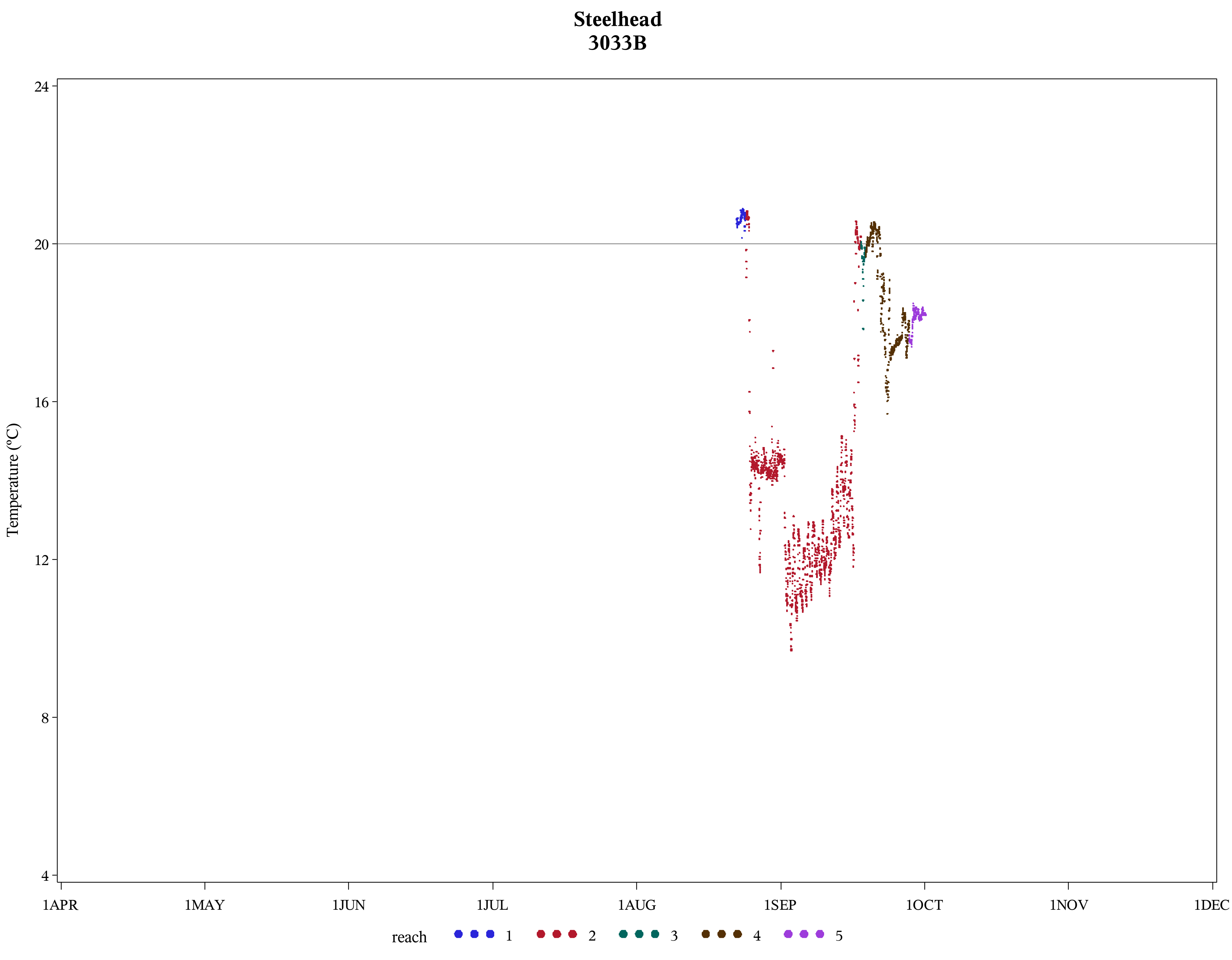
Steelhead
3033B
Temperature (°C)
24
20
16
12
8
4
1APR
1MAY
1JUN
1JUL
1AUG
1SEP
1OCT
1NOV
1DEC
reach
1
2
3
4
5

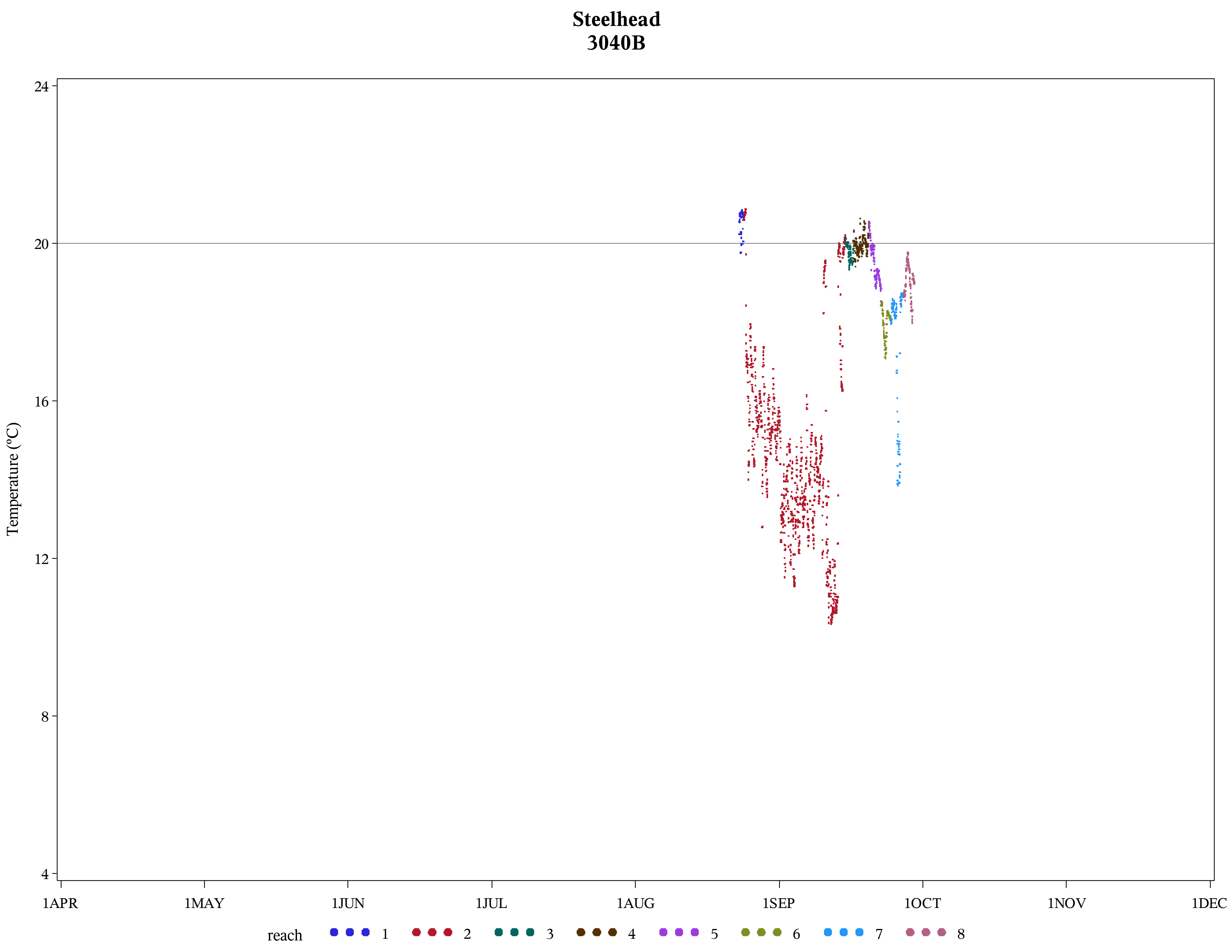
Steelhead
3040B
Temperature (°C)
24
20
16
12
8
4
1APR
1MAY
1JUN
1JUL
1AUG
1SEP
1OCT
1NOV
1DEC
reach
1
2
3
4
5
6
7
8

Steelhead
3047C

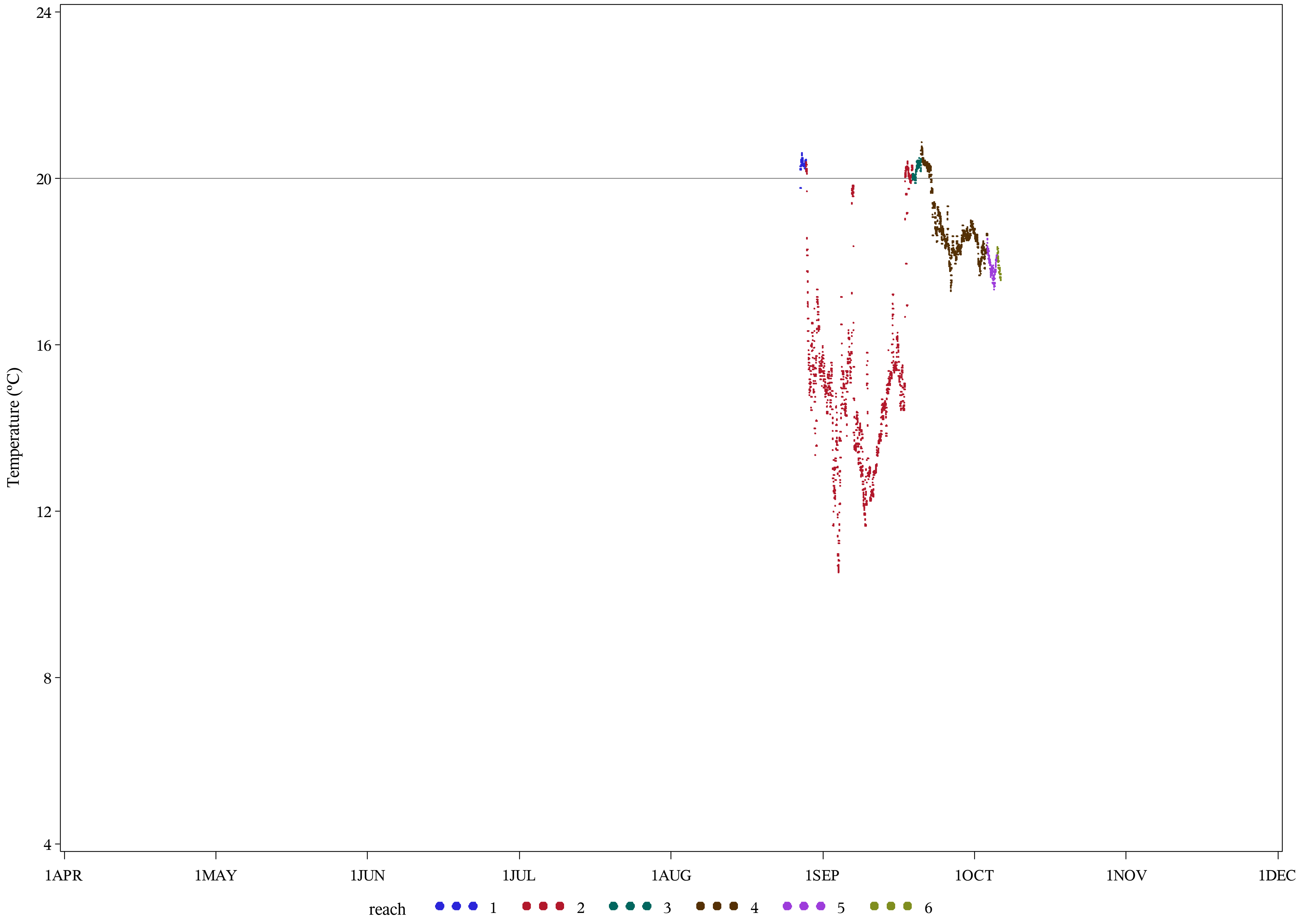

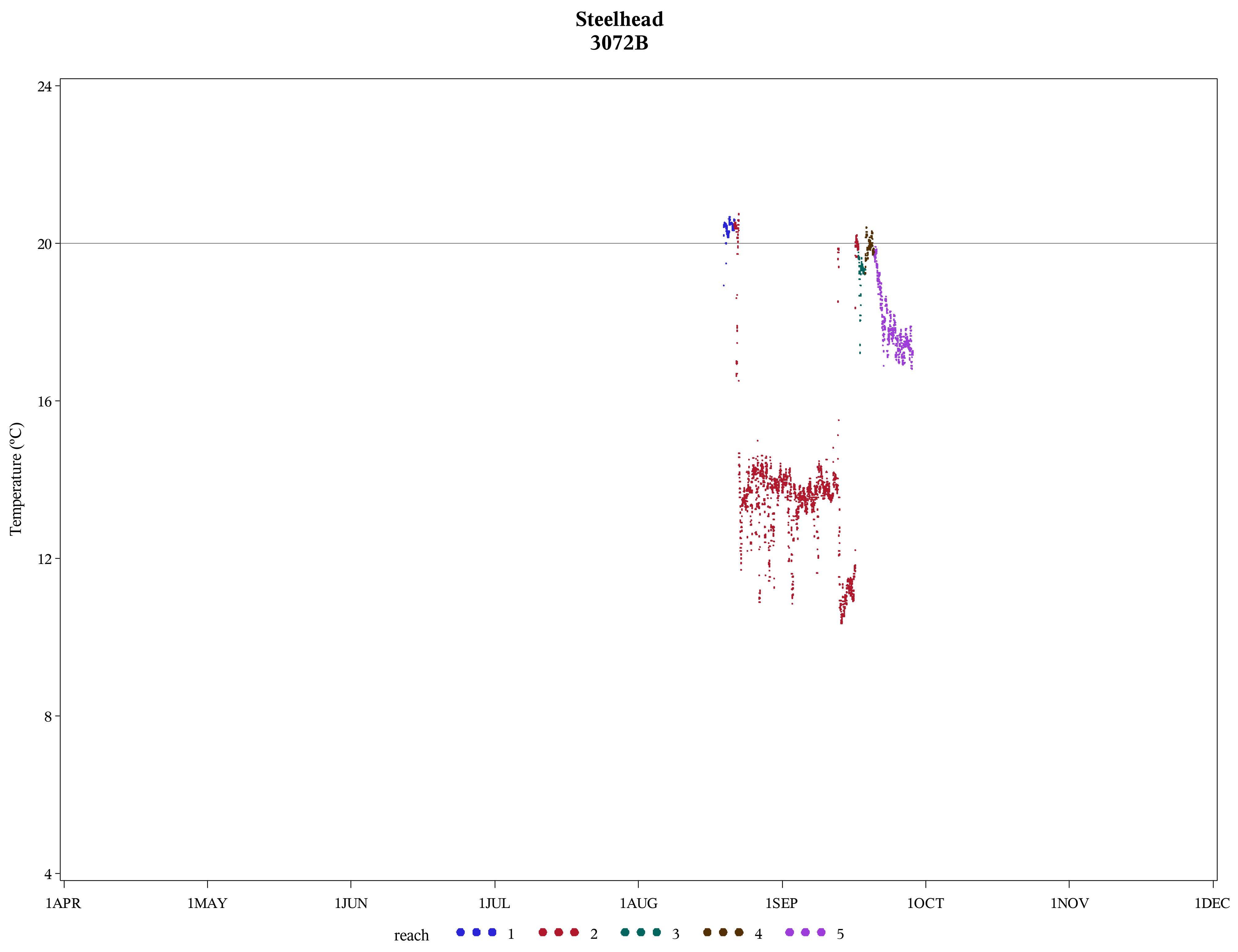

Steelhead
3072B
Temperature (°C)
24
20
16
12
8
4
1APR
1MAY
1JUN
1JUL
1AUG
1SEP
1OCT
1NOV
1DEC
reach
1
2
3
4
5

Steelhead
3081C

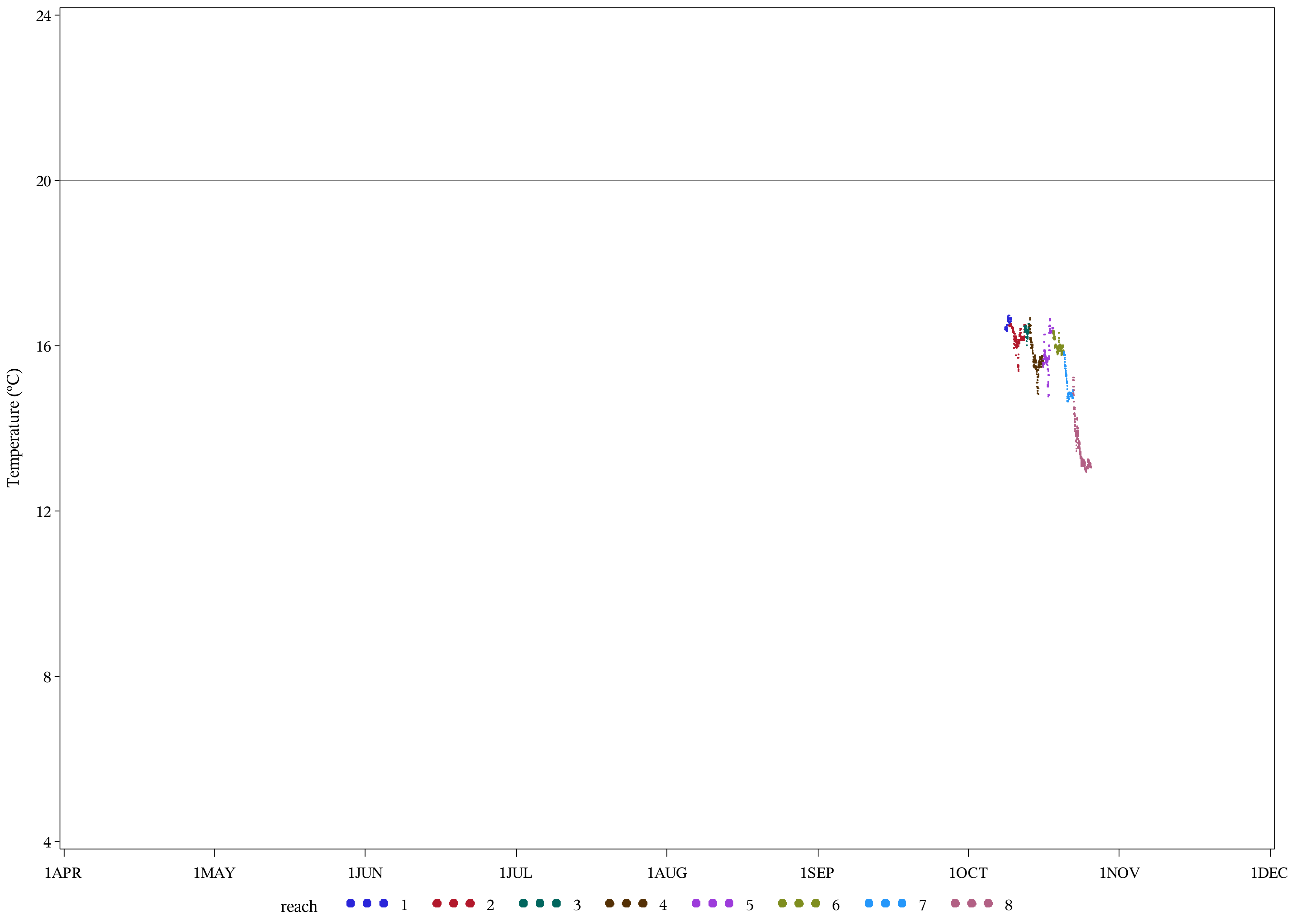

Steelhead
3088B

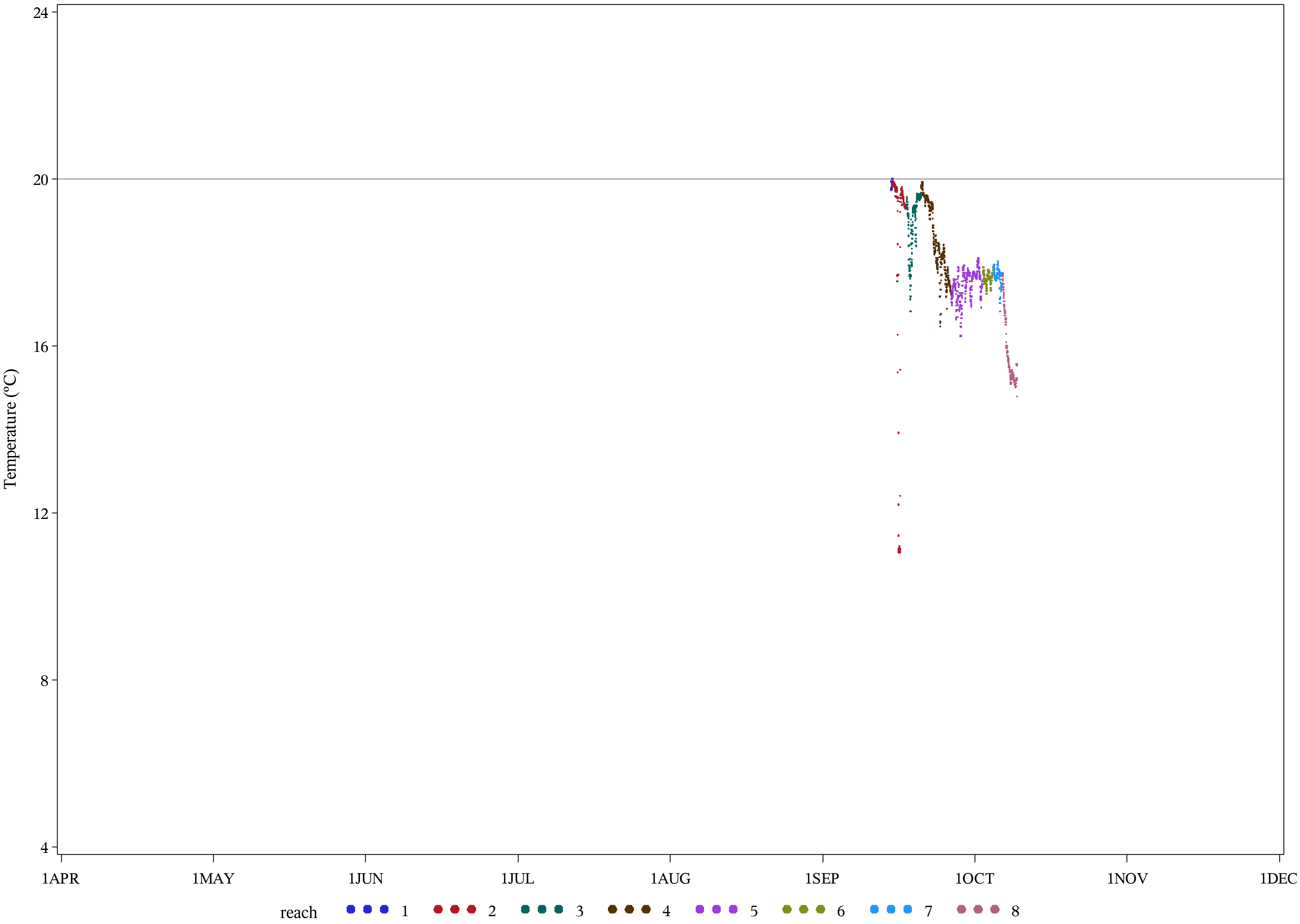

Steelhead
3570A

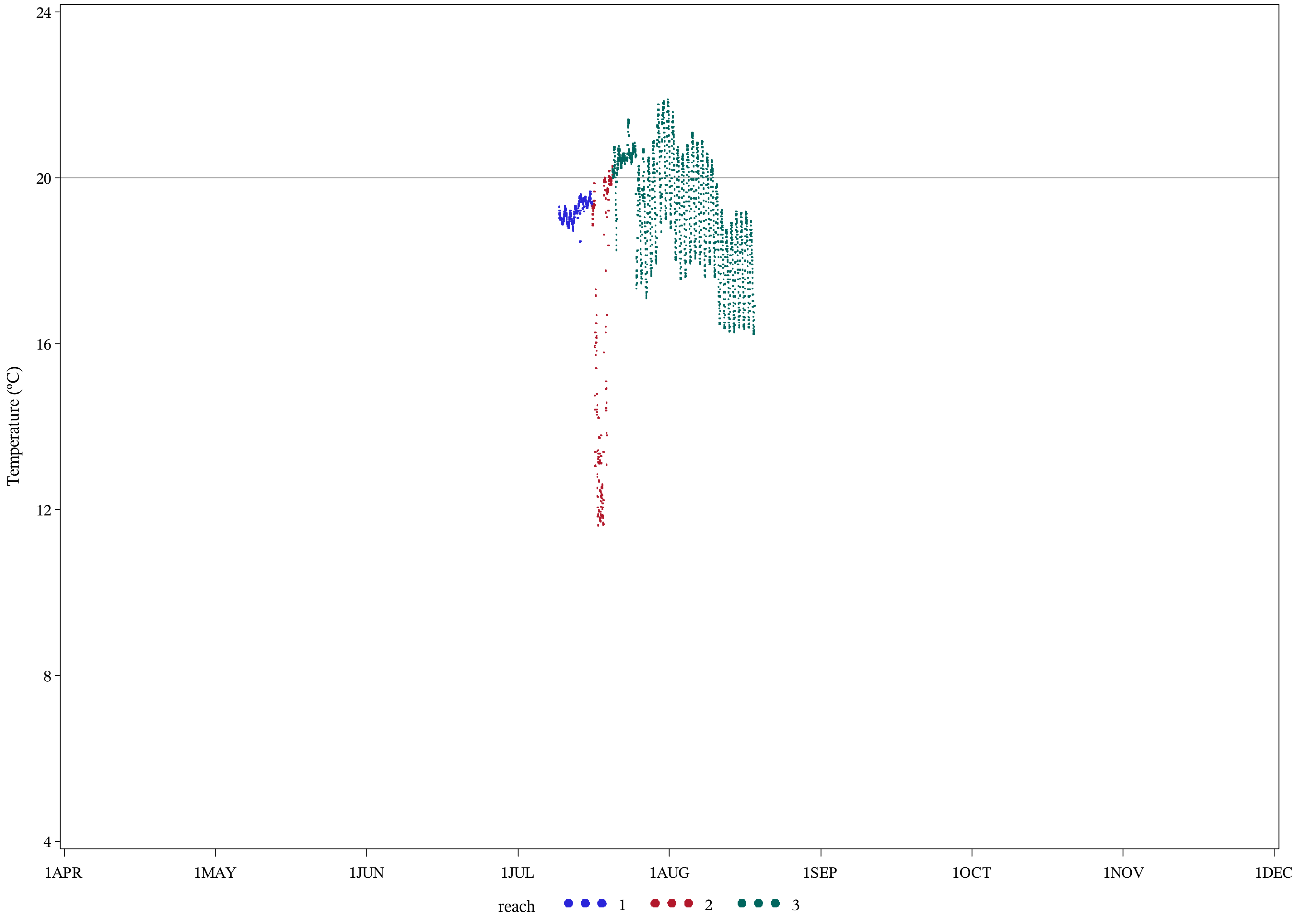

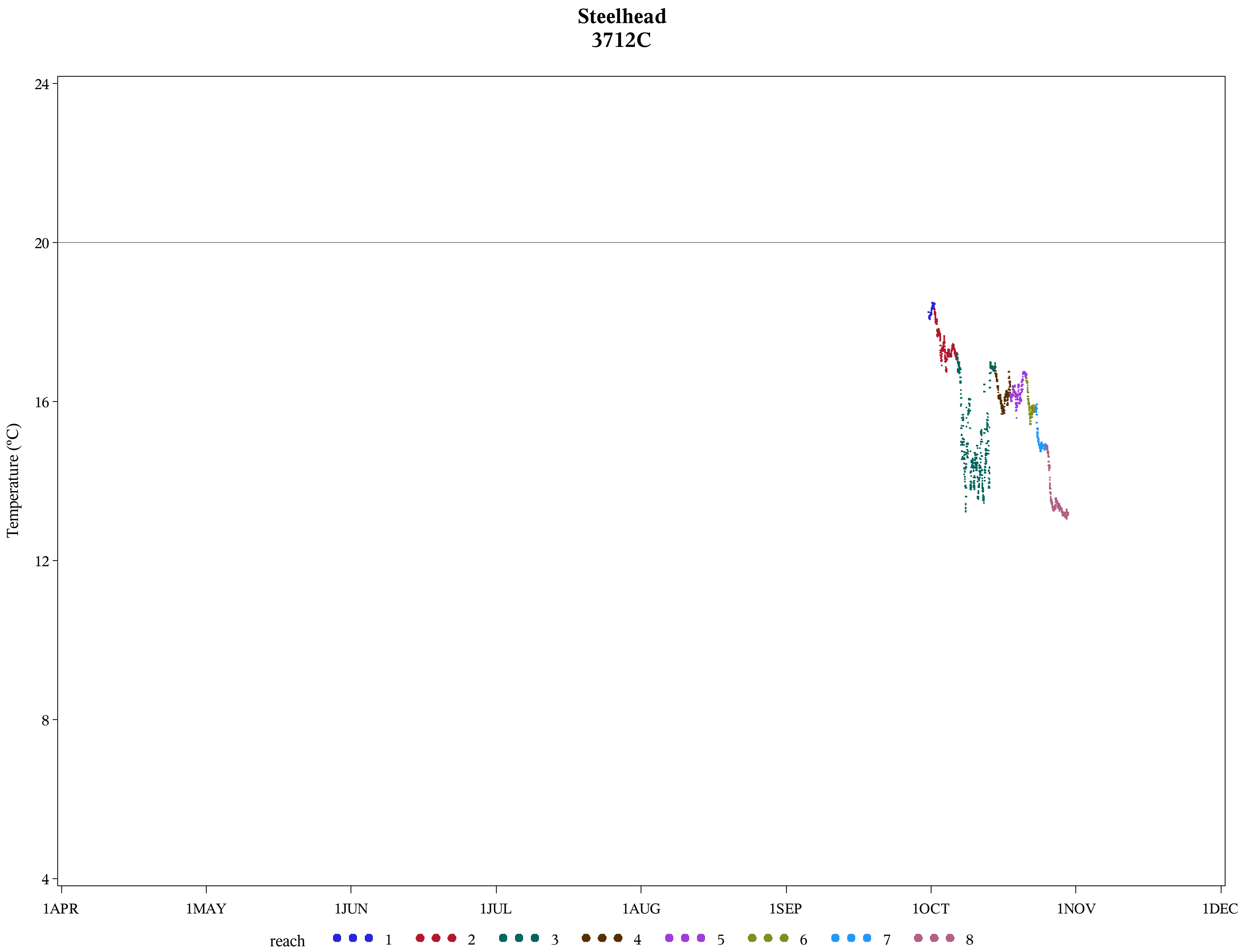

Steelhead
3712C
Temperature (°C)
24
20
16
12
8
4
1APR
1MAY
1JUN
1JUL
1AUG
1SEP
1OCT
1NOV
1DEC
reach
1
2
3
4
5
6
7
8

Steelhead
3718A

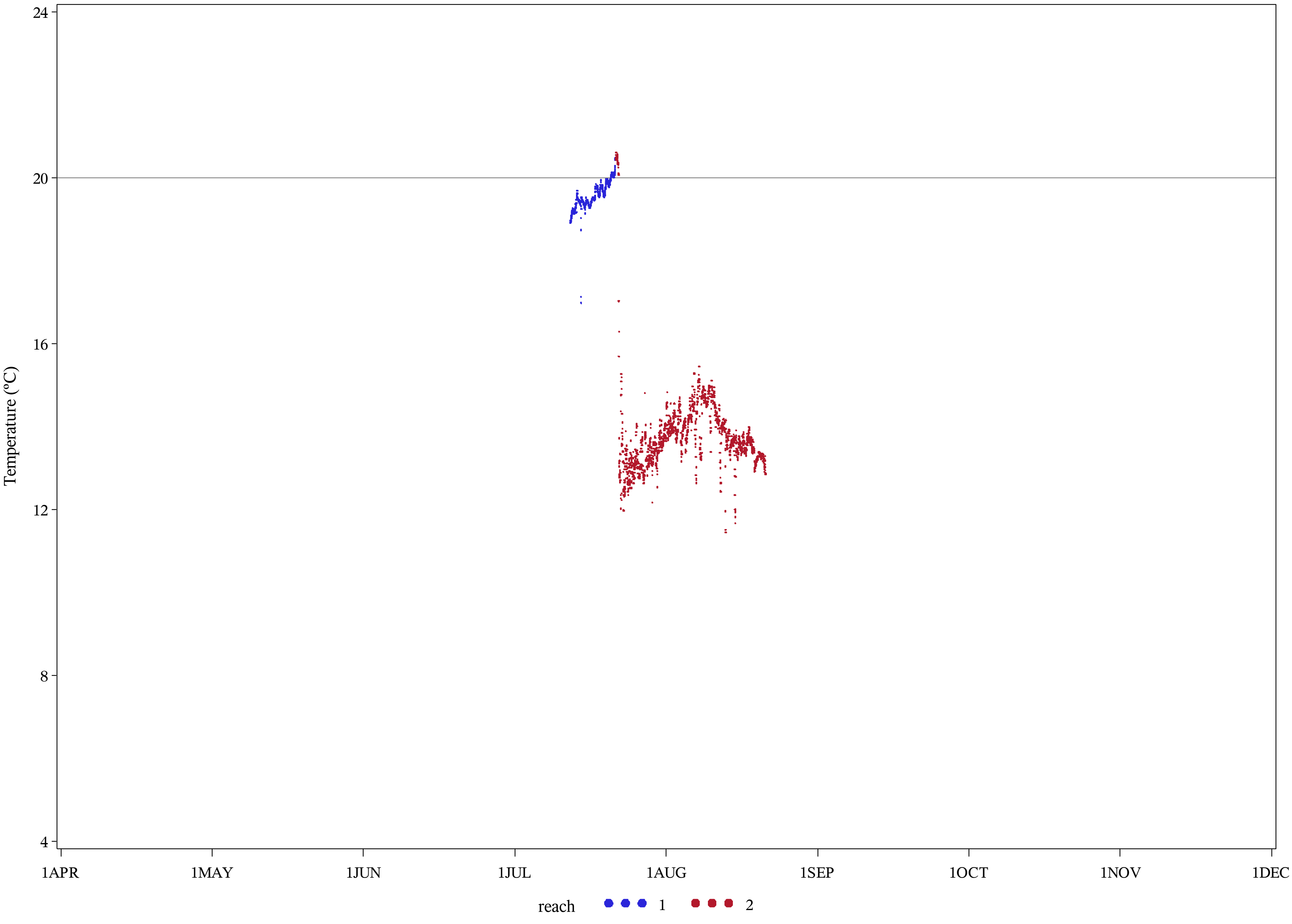

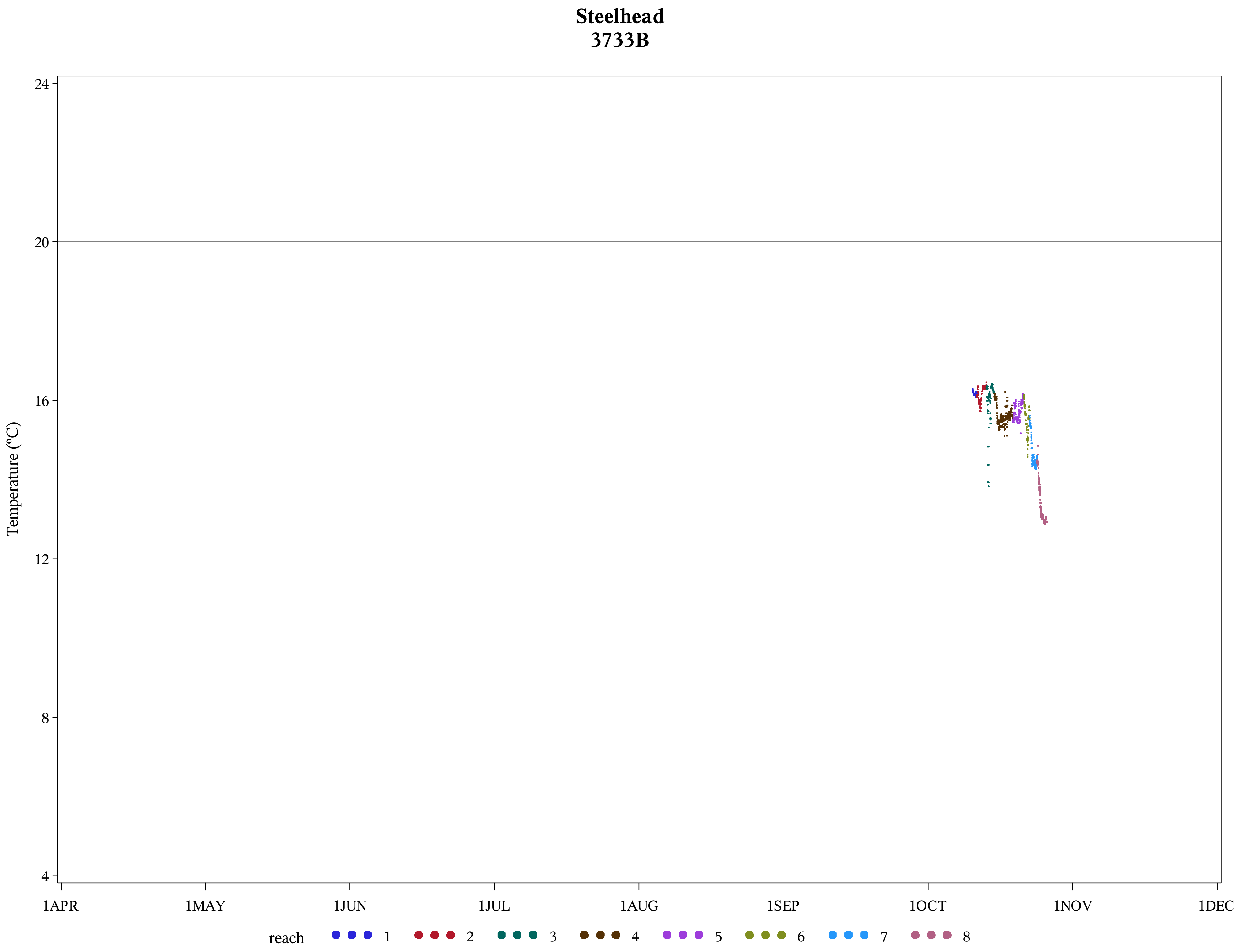
Steelhead
3733B
Temperature (°C)
24
20
16
12
8
4
1APR
1MAY
1JUN
1JUL
1AUG
1SEP
1OCT
1NOV
1DEC
reach
1
2
3
4
5
6
7
8

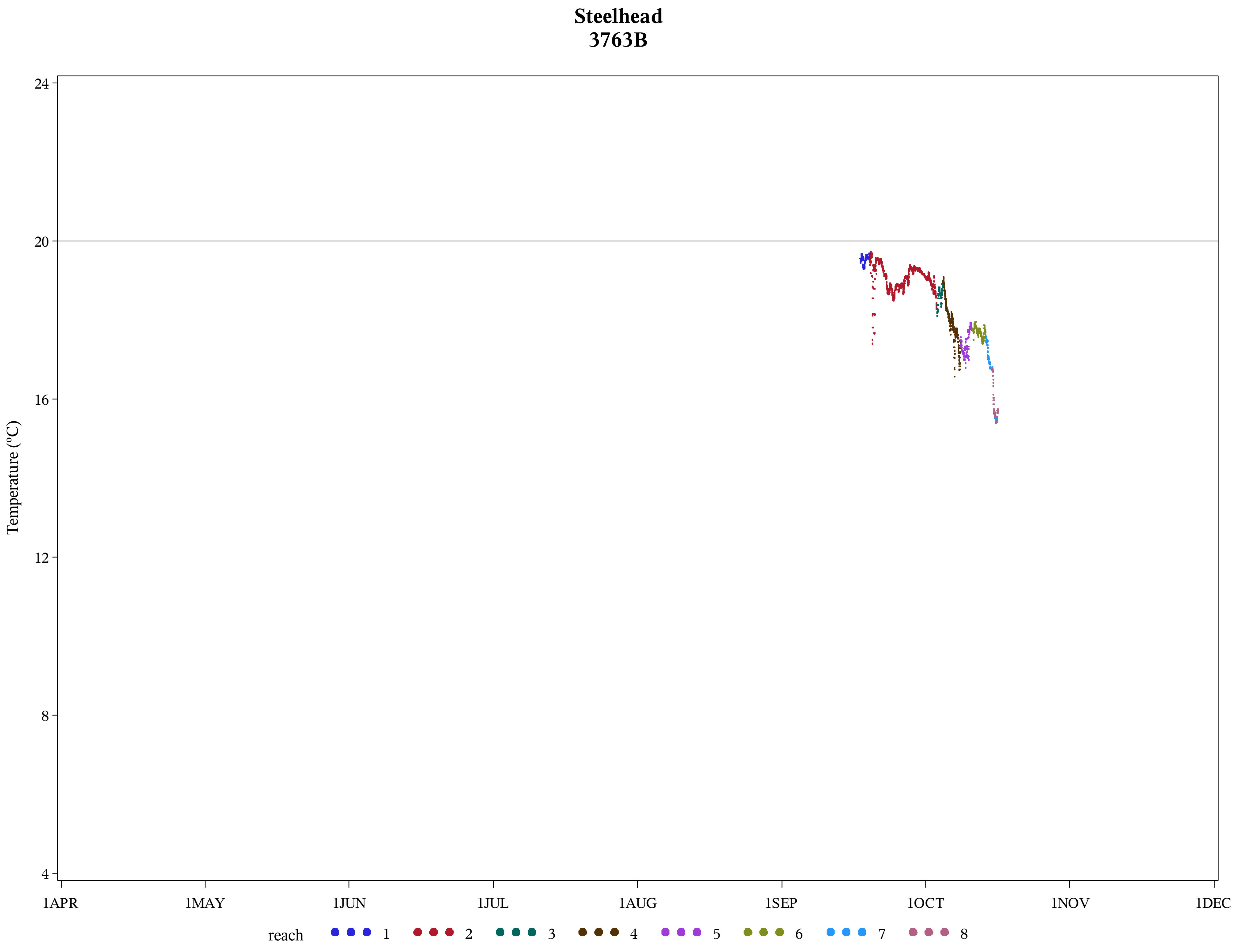
Steelhead
3763B
Temperature (°C)
24
20
16
12
8
4
1APR
1MAY
1JUN
1JUL
1AUG
1SEP
1OCT
1NOV
1DEC
reach
1
2
3
4
5
6
7
8

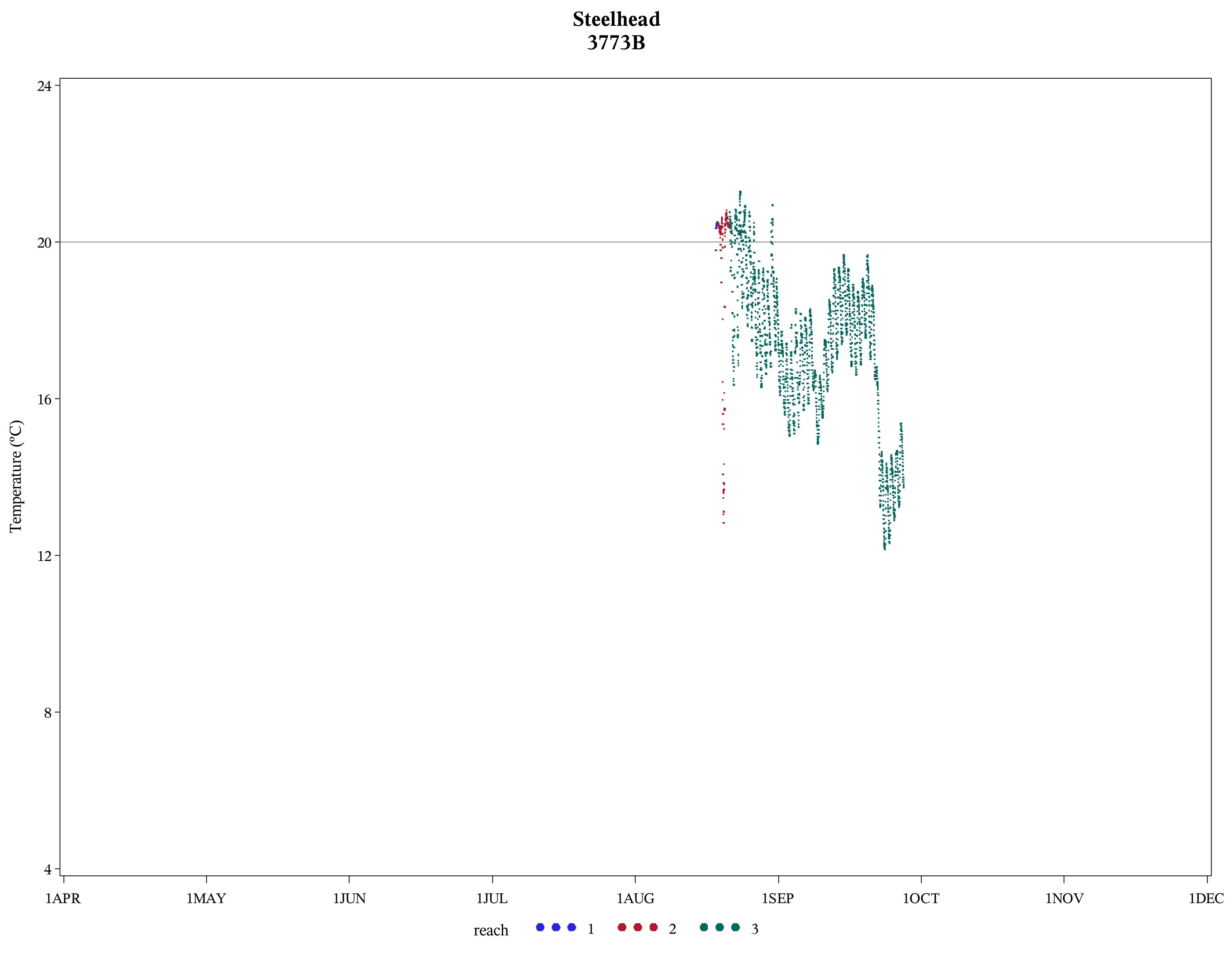

Steelhead
3773B
Temperature (°C)
24
20
16
12
8
4
1APR
1MAY
1JUN
1JUL
1AUG
1SEP
1OCT
1NOV
1DEC
reach
1
2
3

Steelhead
3781B

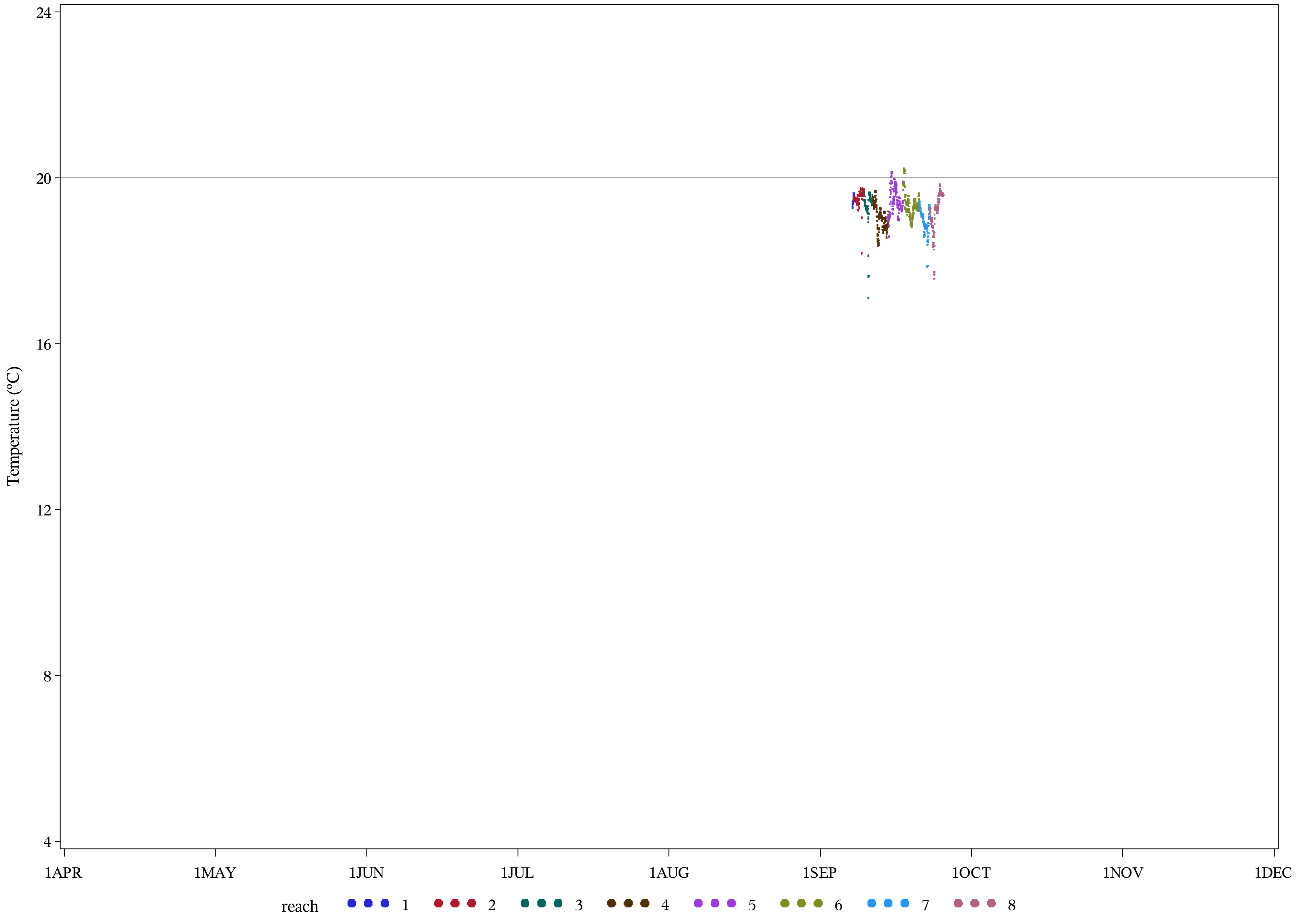

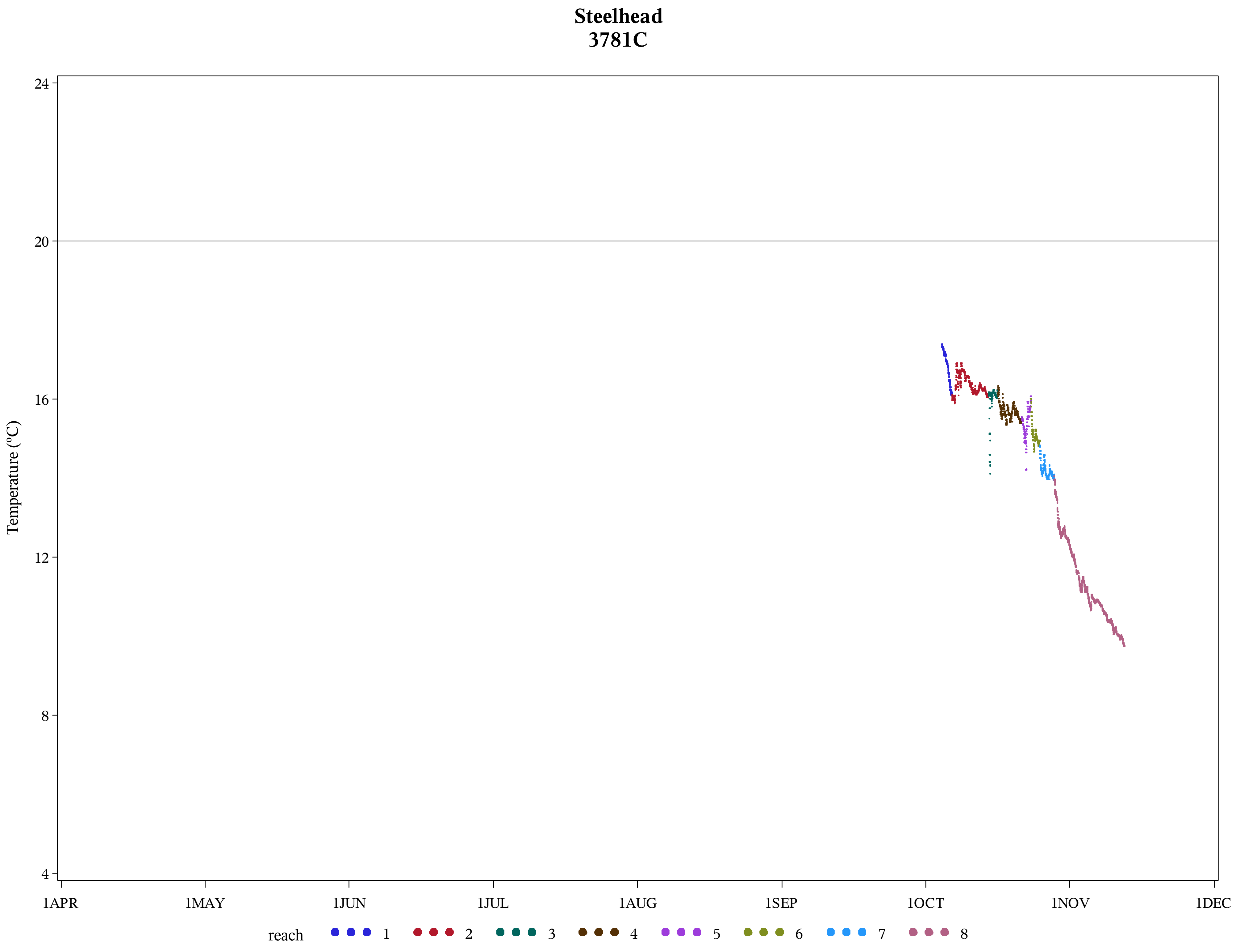

Steelhead
3781C
Temperature (°C)
24
20
16
12
8
4
1APR
1MAY
1JUN
1JUL
1AUG
1SEP
1OCT
1NOV
1DEC
reach
1
2
3
4
5
6
7
8

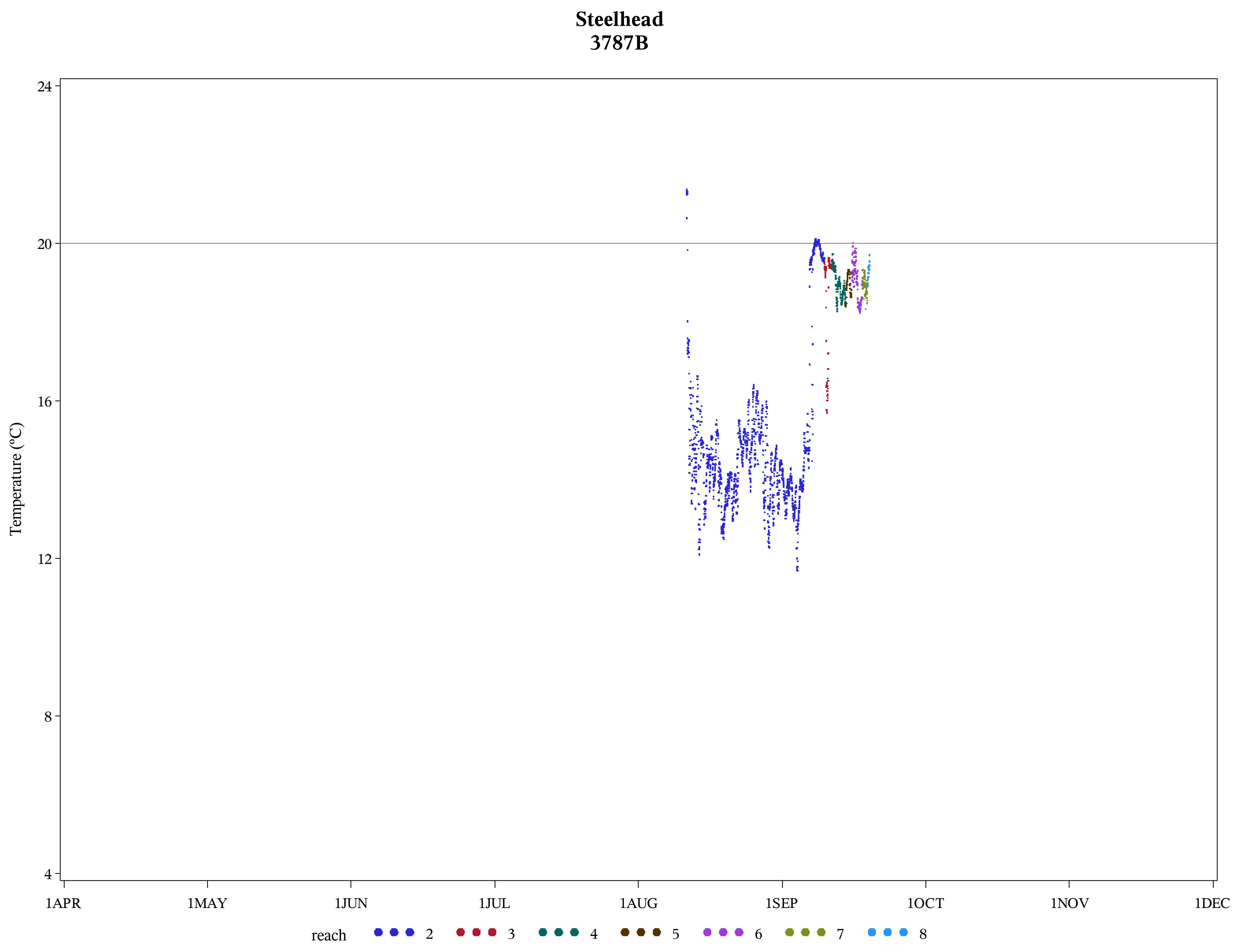

Steelhead
3787B
Temperature (°C)
24
20
16
12
8
4
1APR
1MAY
1JUN
1JUL
1AUG
1SEP
1OCT
1NOV
1DEC
reach
2
3
4
5
6
7
8

Steelhead
3790A

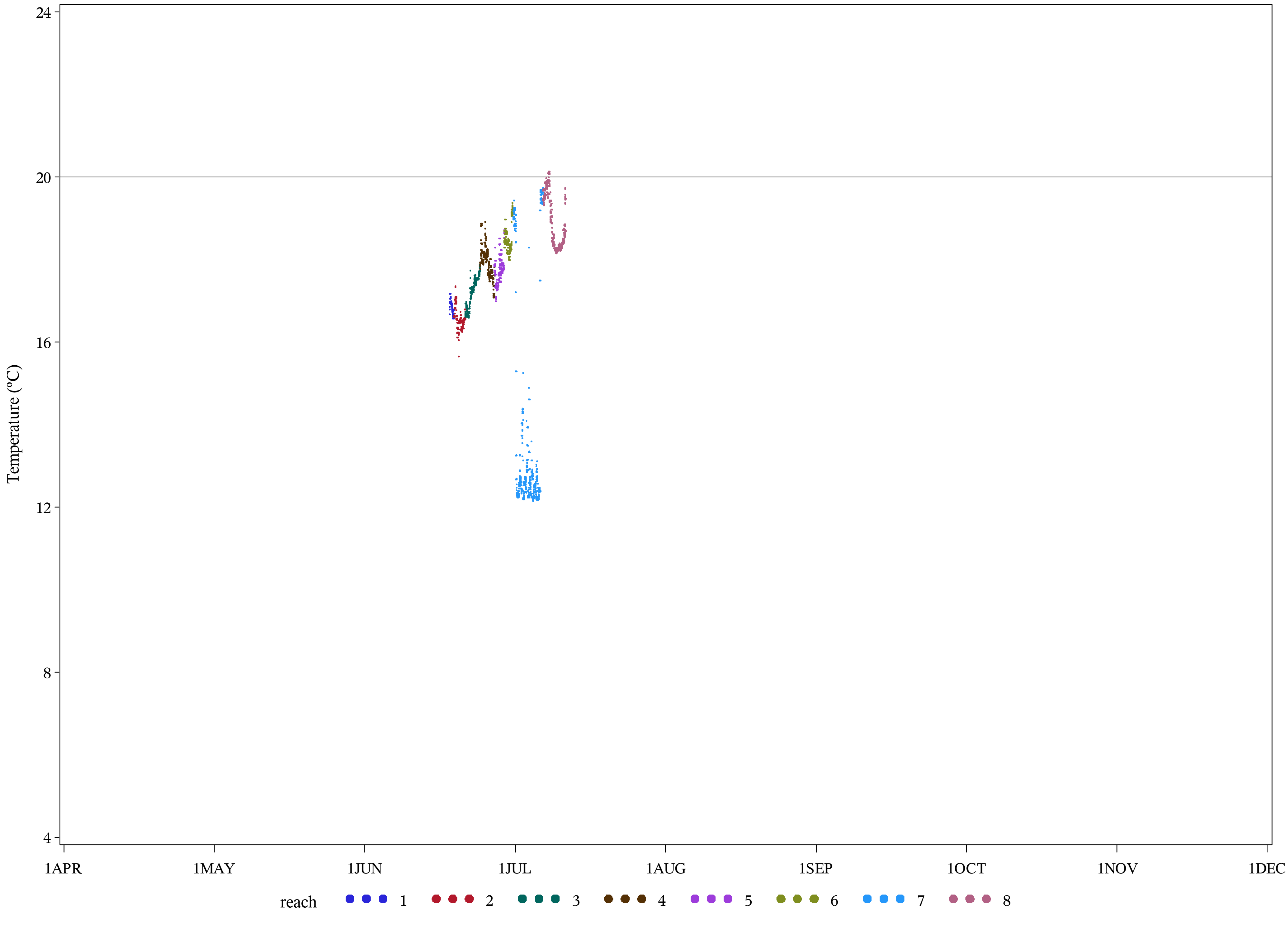

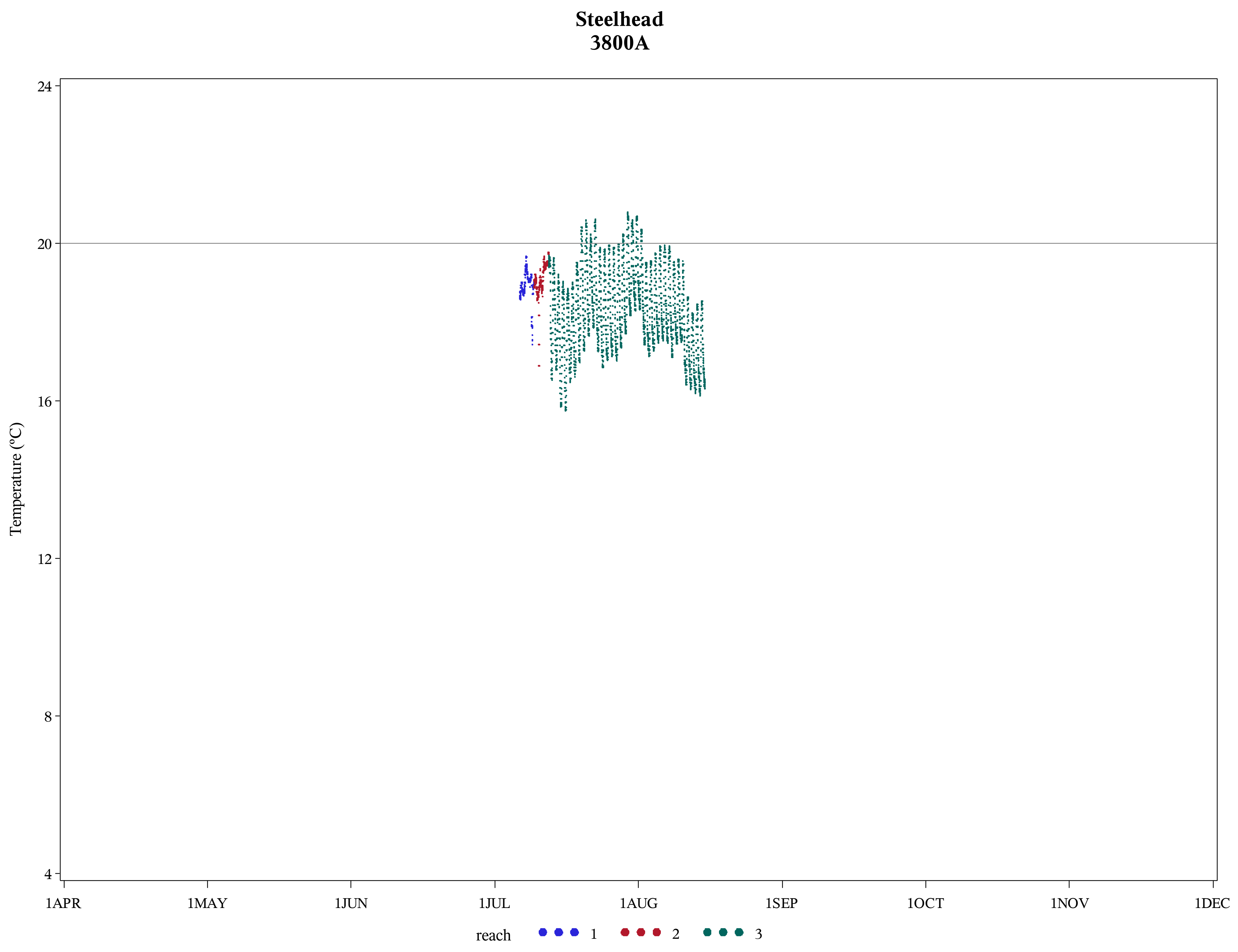
Steelhead
3800A
Temperature (°C)
24
20
16
12
8
4
1APR
1MAY
1JUN
1JUL
1AUG
1SEP
1OCT
1NOV
1DEC
reach
1
2
3

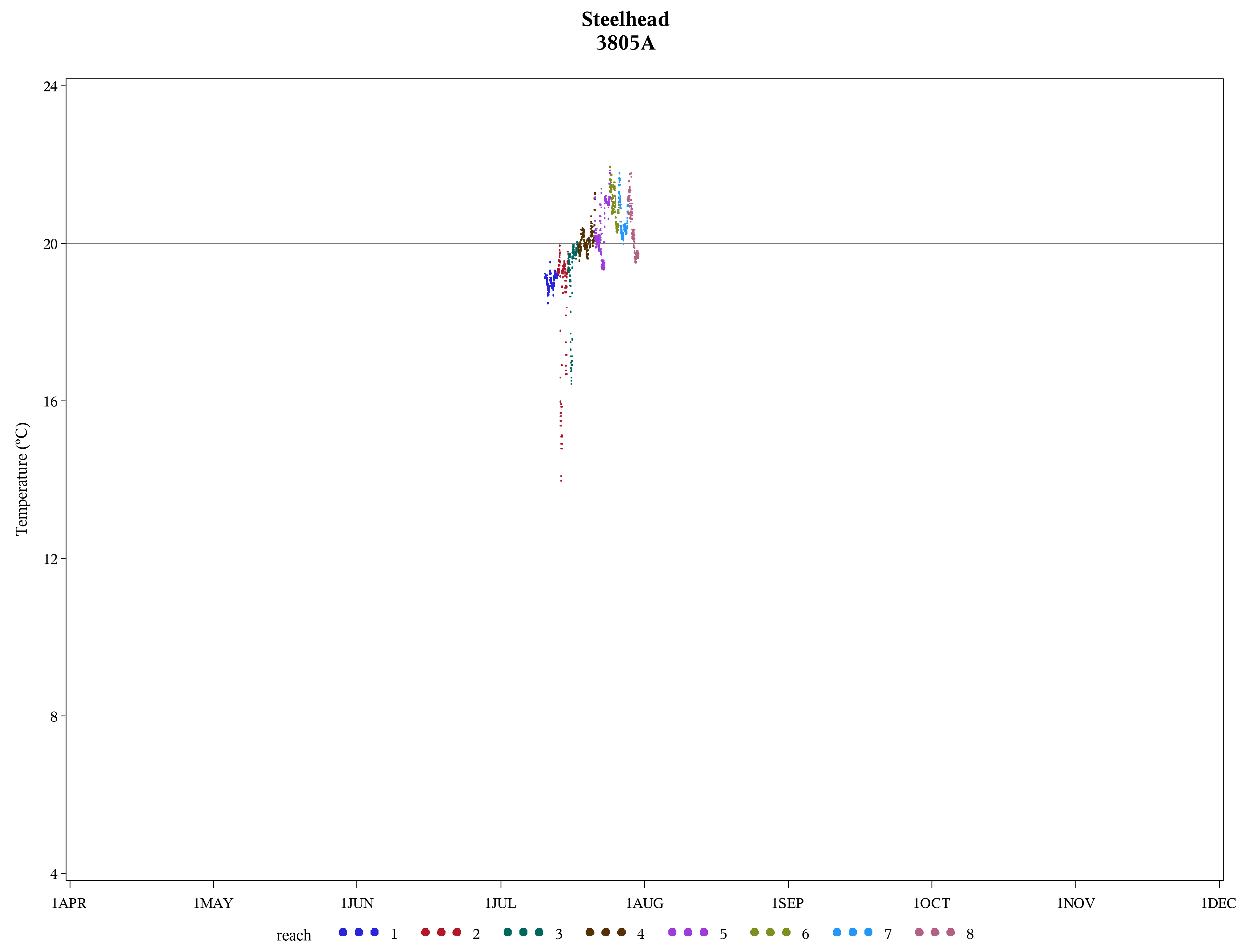

Steelhead
3805A
Temperature (°C)
24
20
16
12
8
4
1APR
1MAY
1JUN
1JUL
1AUG
1SEP
1OCT
1NOV
1DEC
reach
1
2
3
4
5
6
7
8

Steelhead
3814A

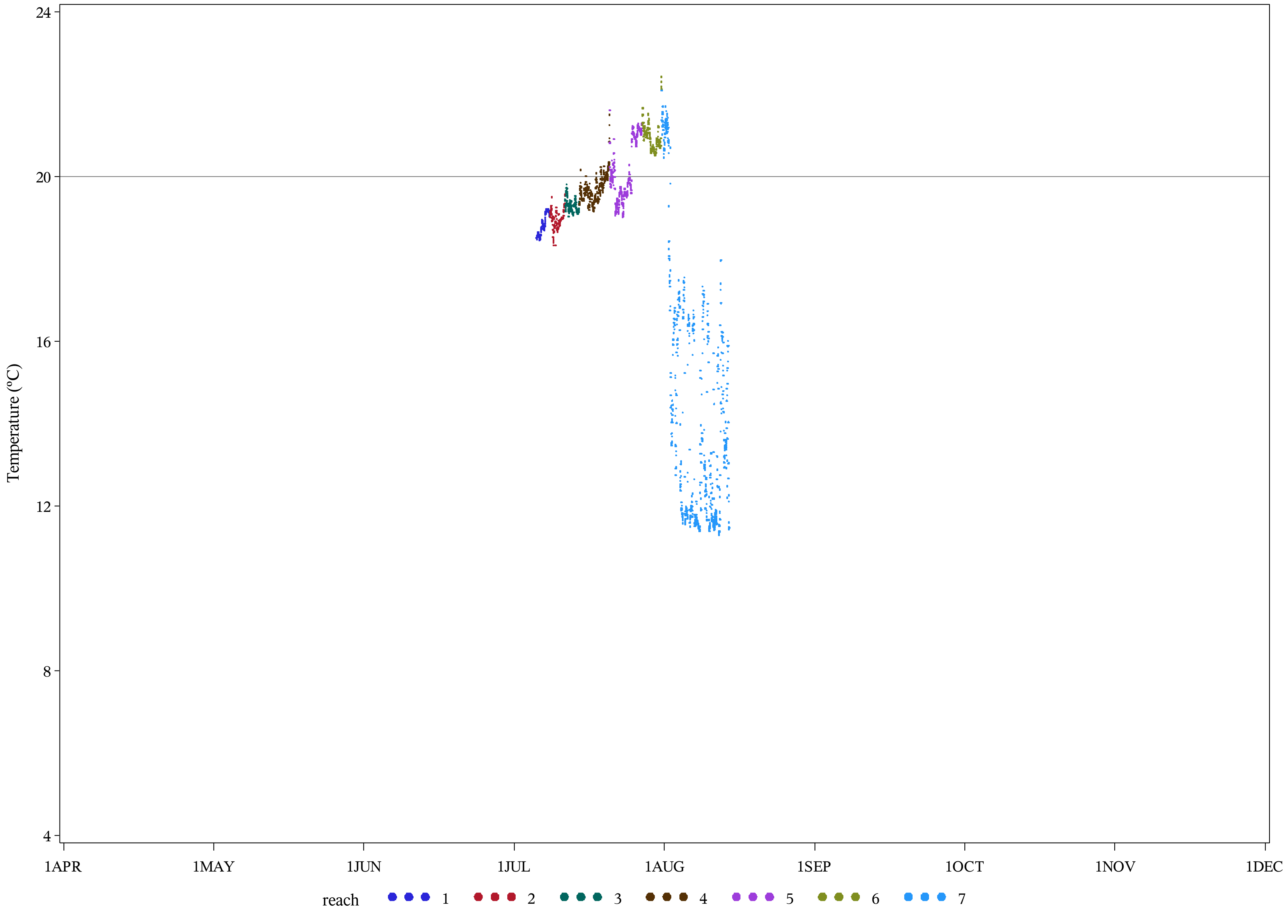

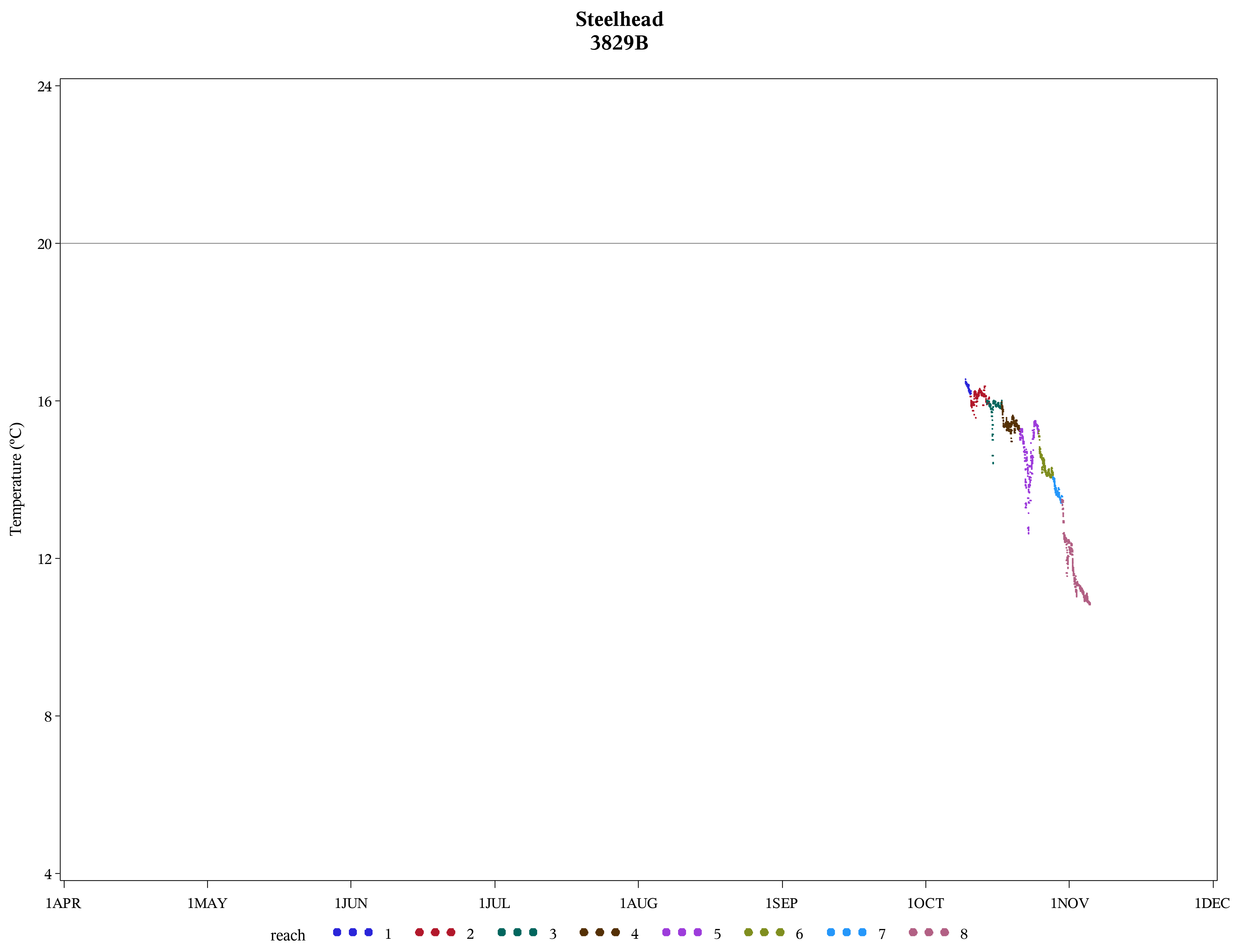

Steelhead
3829B
Temperature (°C)
24
20
16
12
8
4
1APR
1MAY
1JUN
1JUL
1AUG
1SEP
1OCT
1NOV
1DEC
reach
1
2
3
4
5
6
7
8

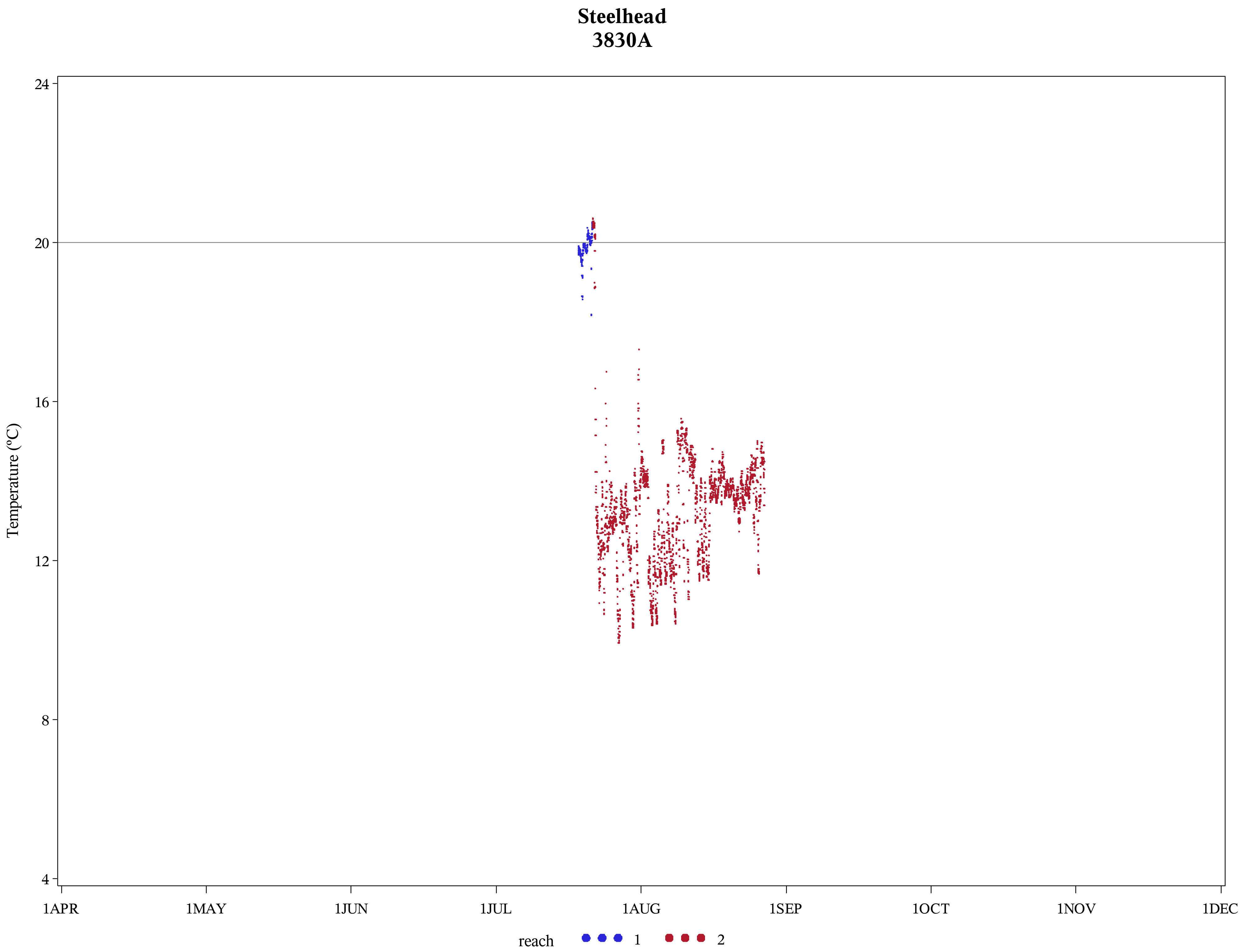

Steelhead
3830A
24
20
16
12
8
4
Temperature (°C)
1APR
1MAY
1JUN
1JUL
1AUG
1SEP
1OCT
1NOV
1DEC
reach
1
2

Steelhead
3831A

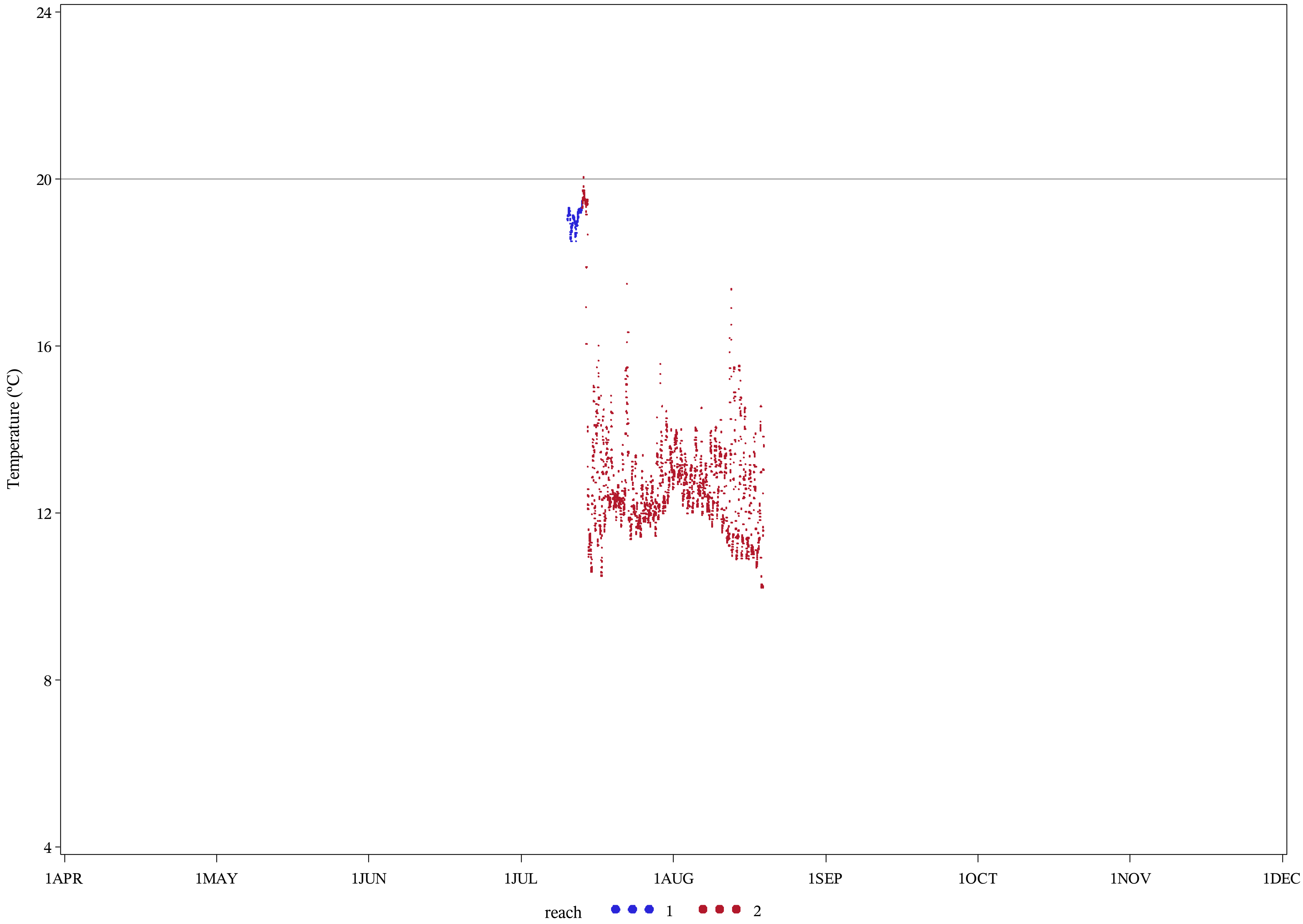

Steelhead
3845A

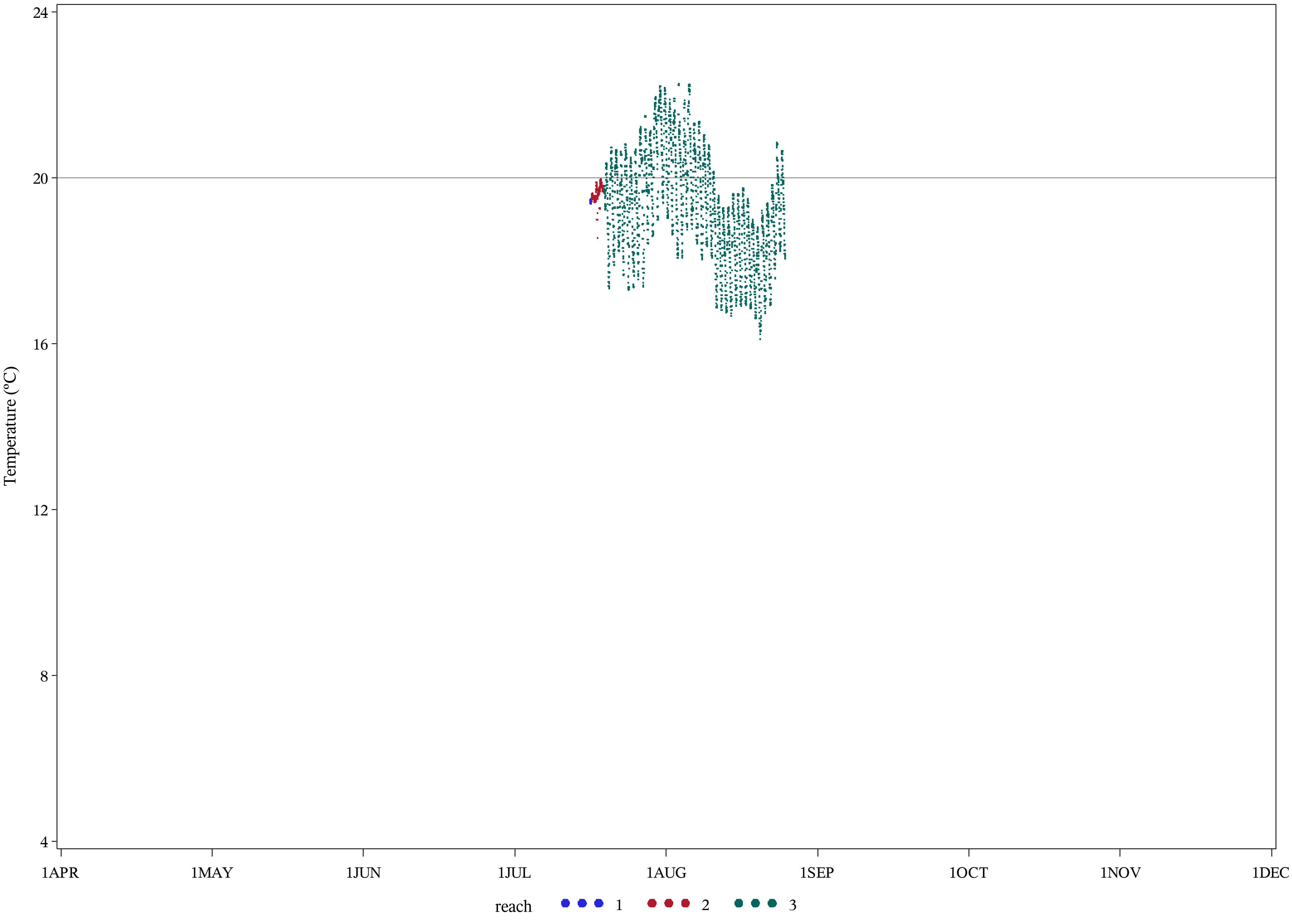

Steelhead
3860A

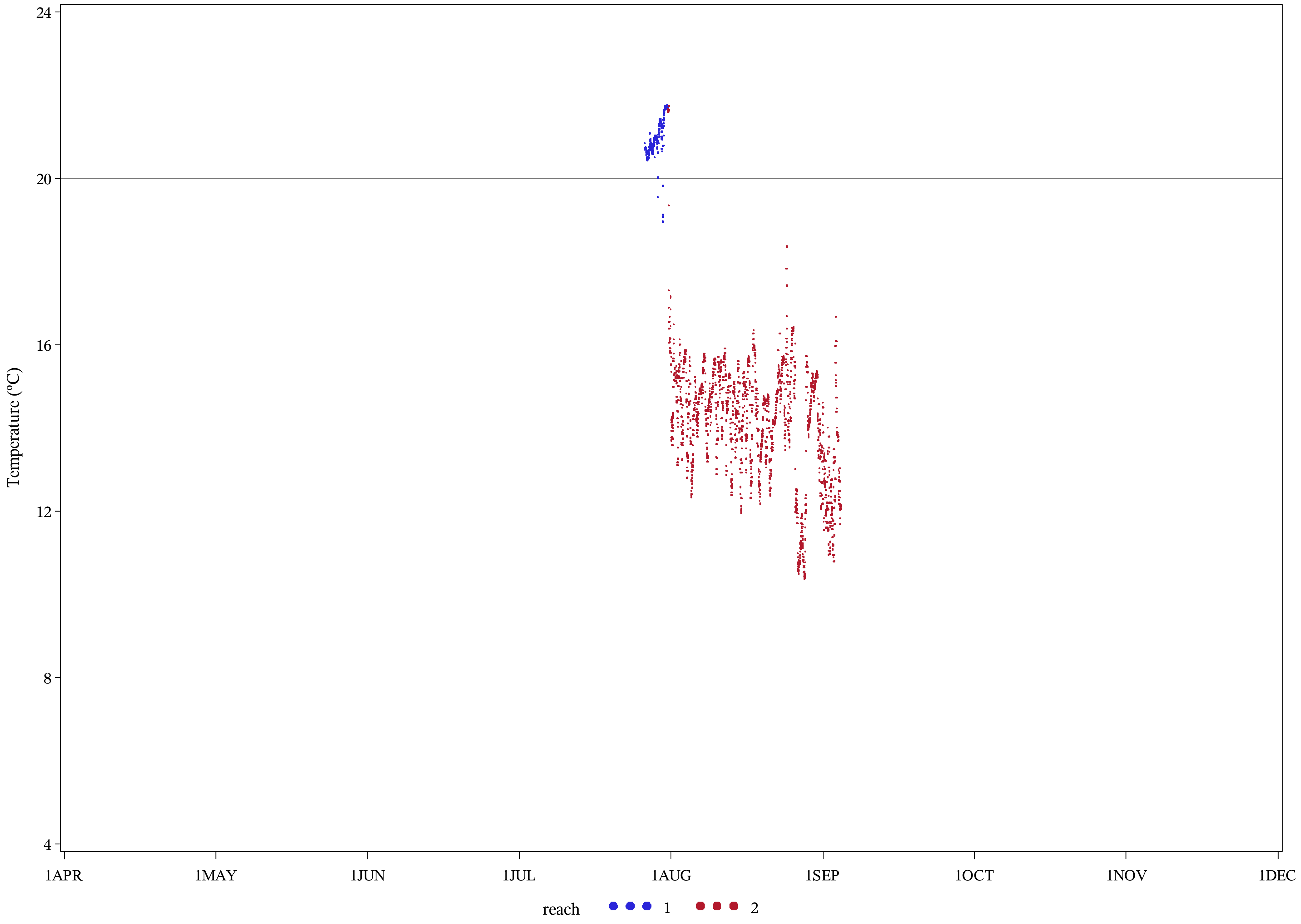

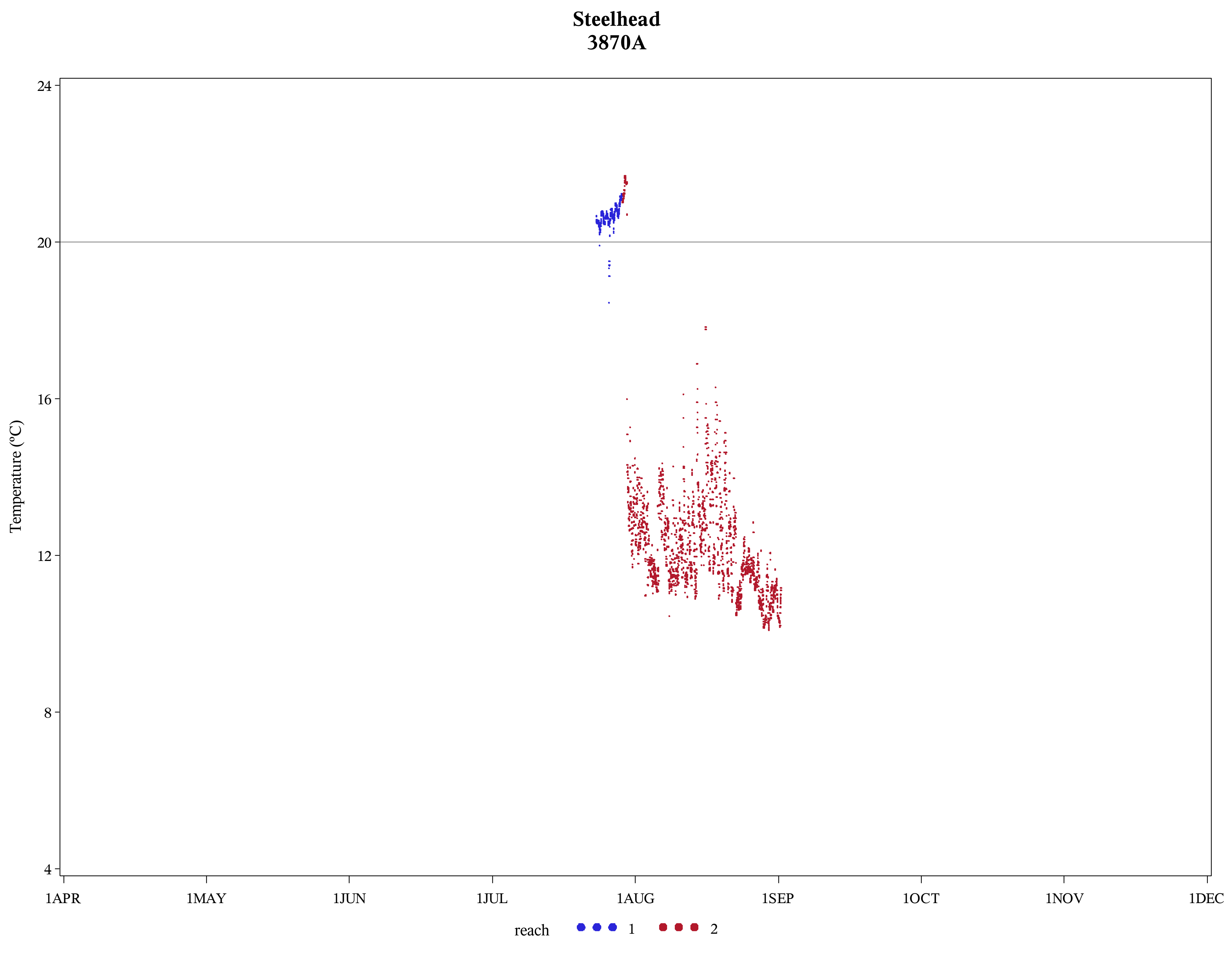

Steelhead
3870A
Temperature (°C)
24
20
16
12
8
4
1APR
1MAY
1JUN
1JUL
1AUG
1SEP
1OCT
1NOV
1DEC
reach
1
2

Summer Chinook
2985A

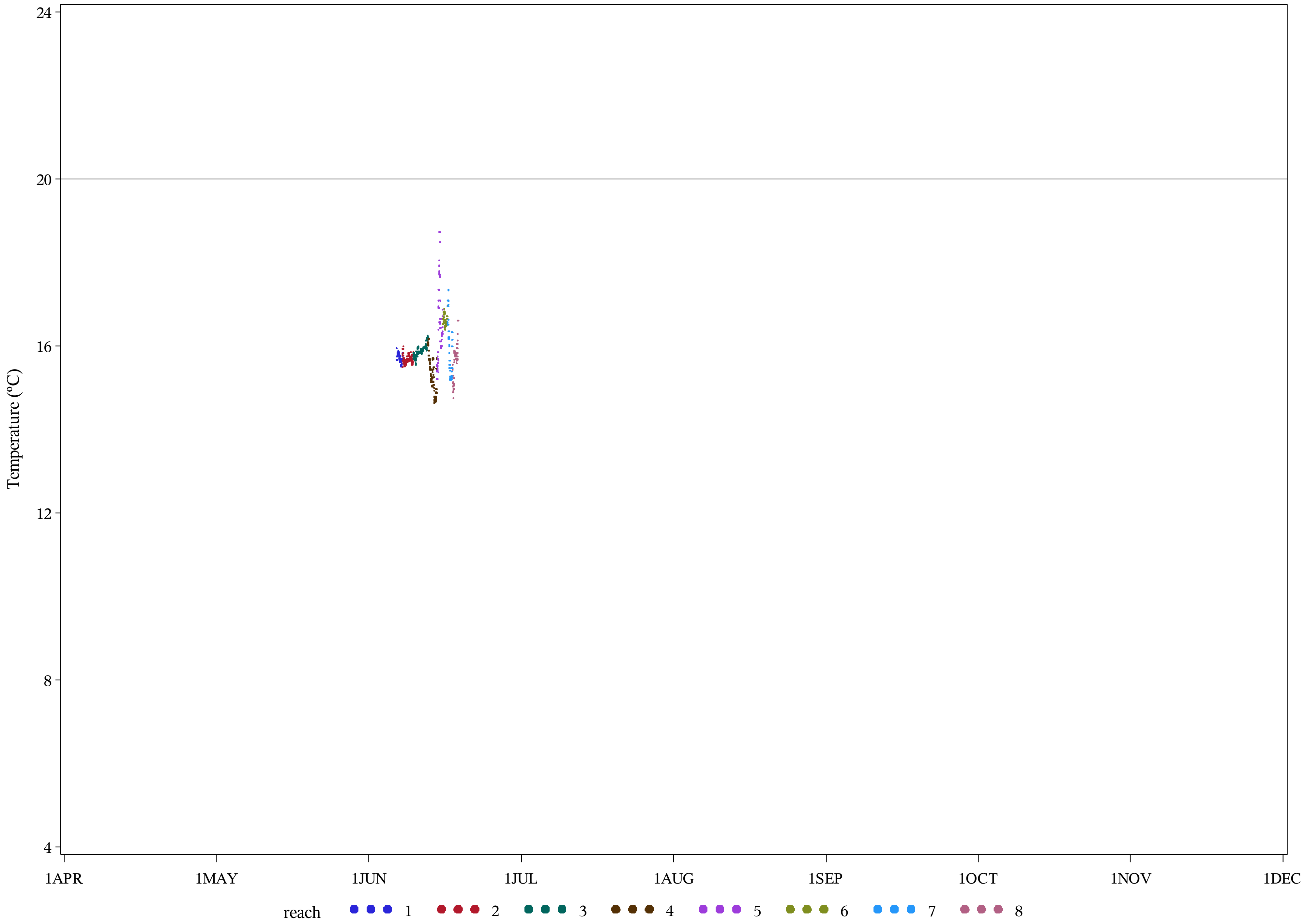

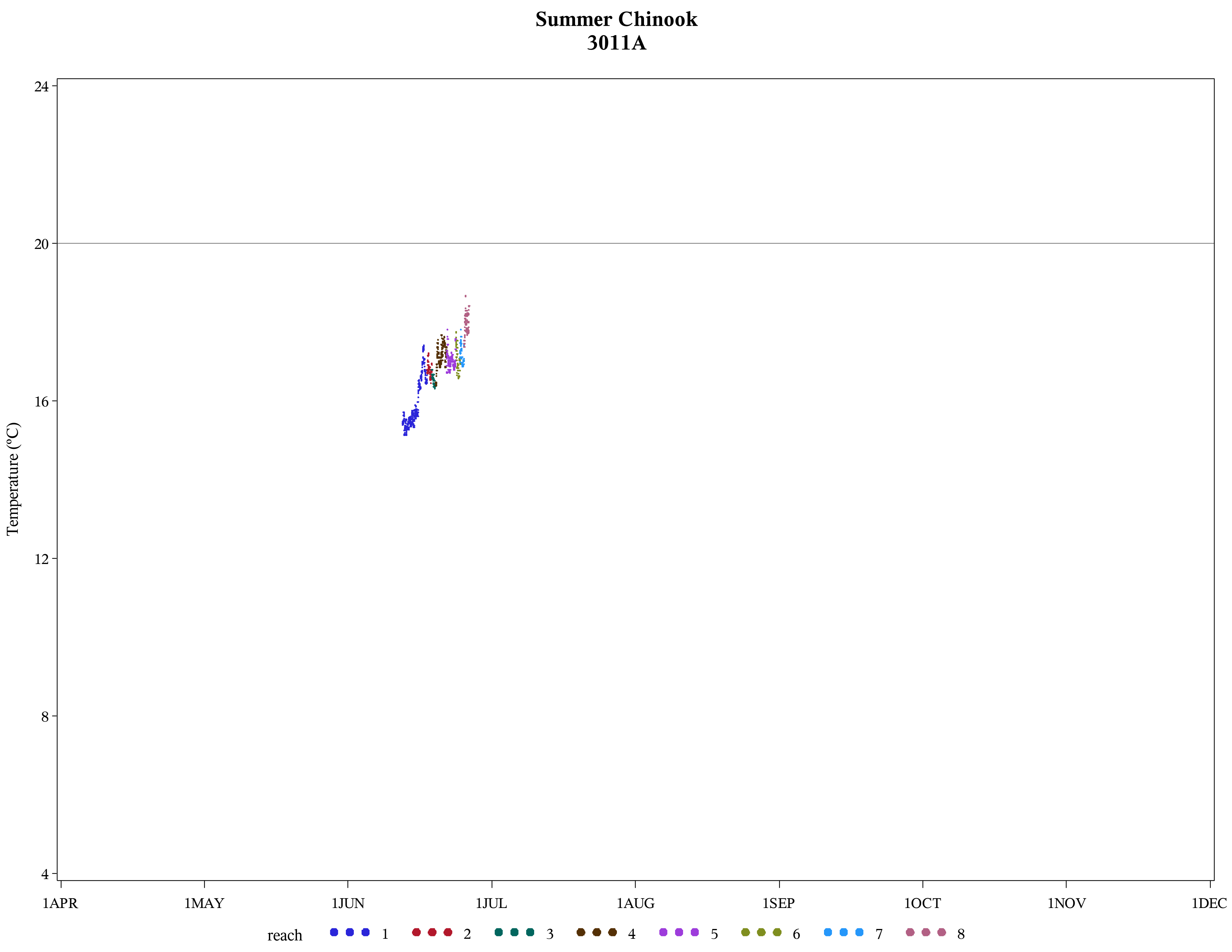

Summary Chinook
3011A
Temperature (°C)
24
20
16
12
8
4
1APR
1MAY
1JUN
1JUL
1AUG
1SEP
1OCT
1NOV
1DEC
reach
1
2
3
4
5
6
7
8

Summer Chinook
3091A

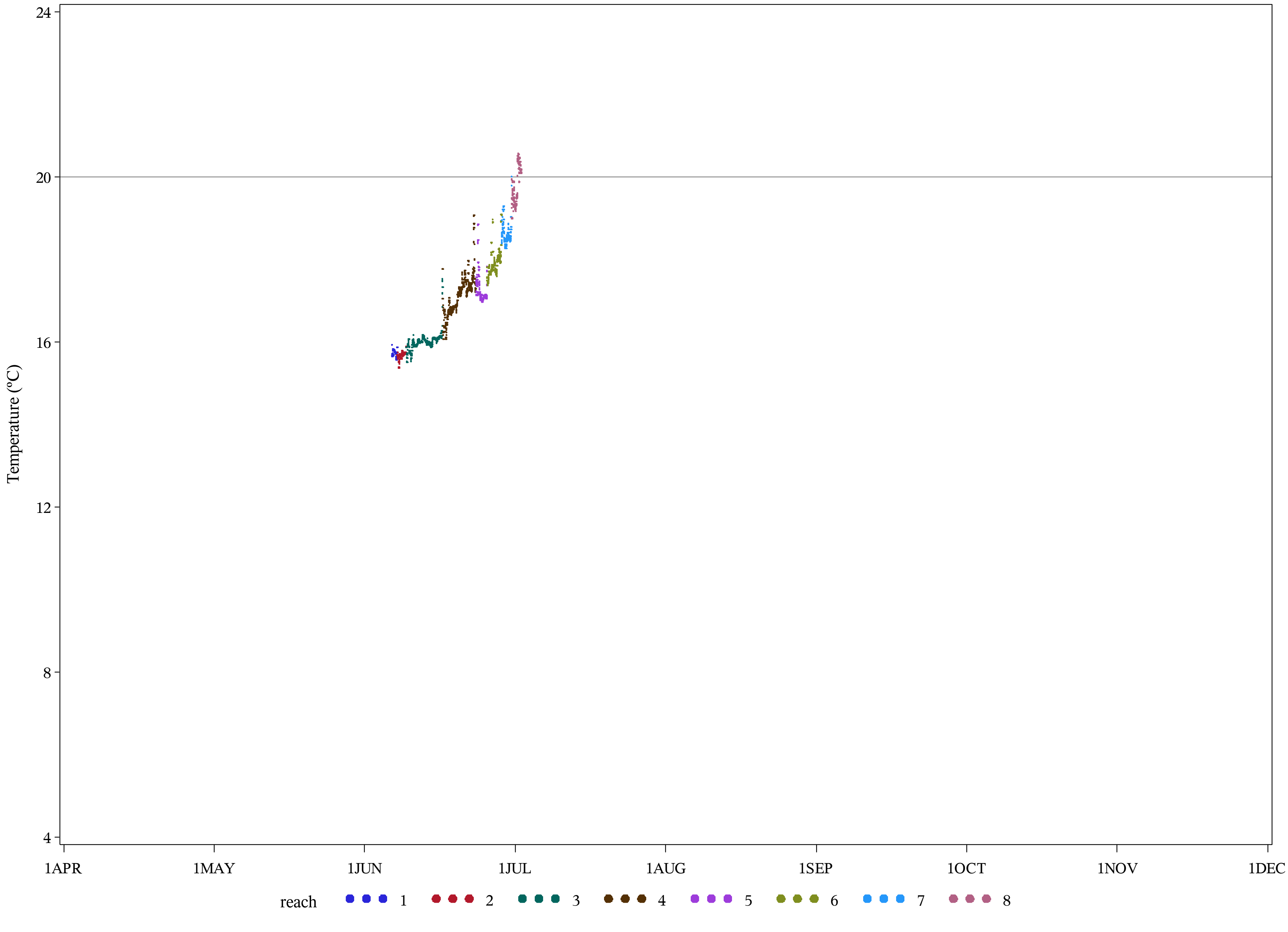

Summer Chinook
3113A

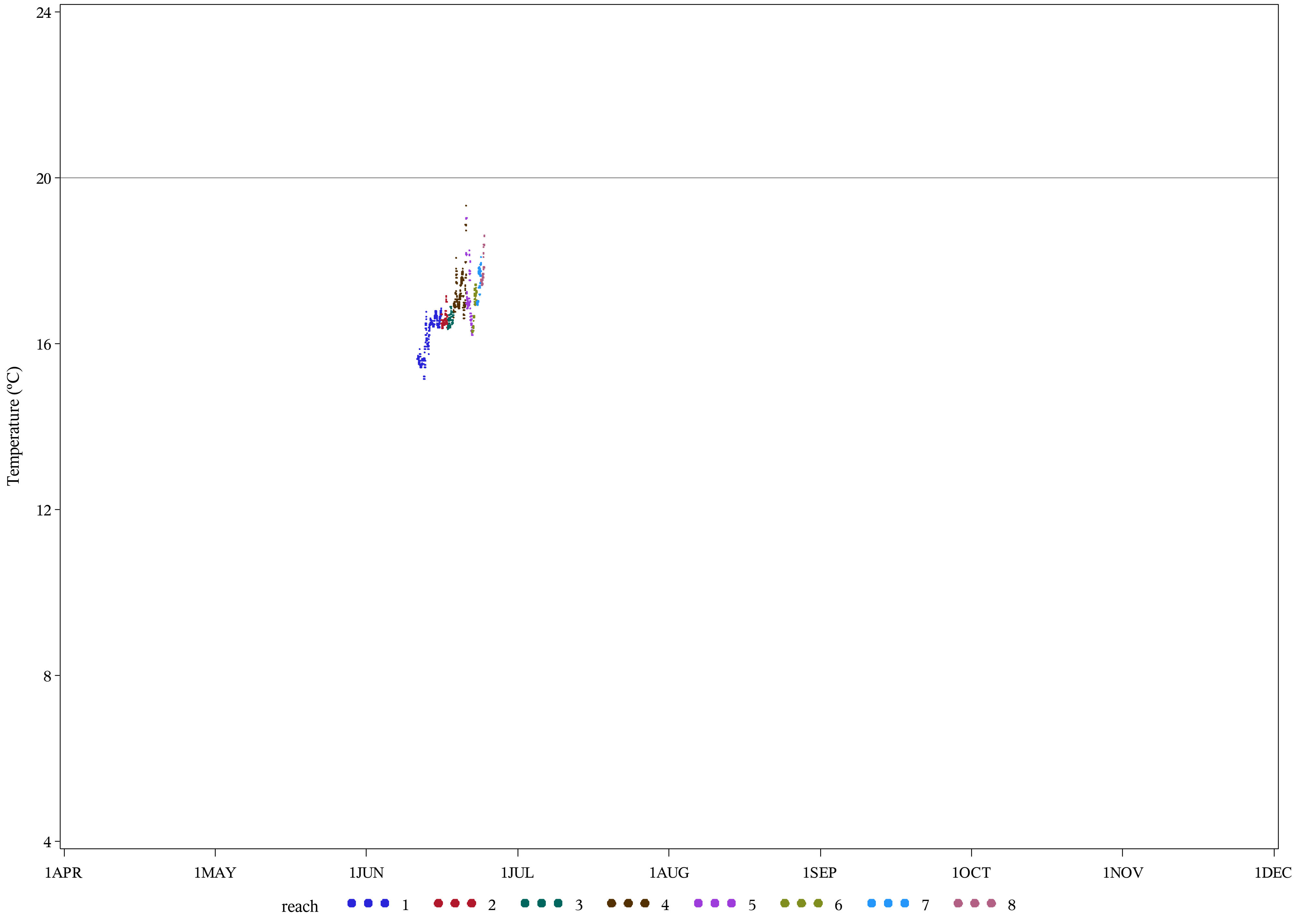

Summer Chinook
3596A

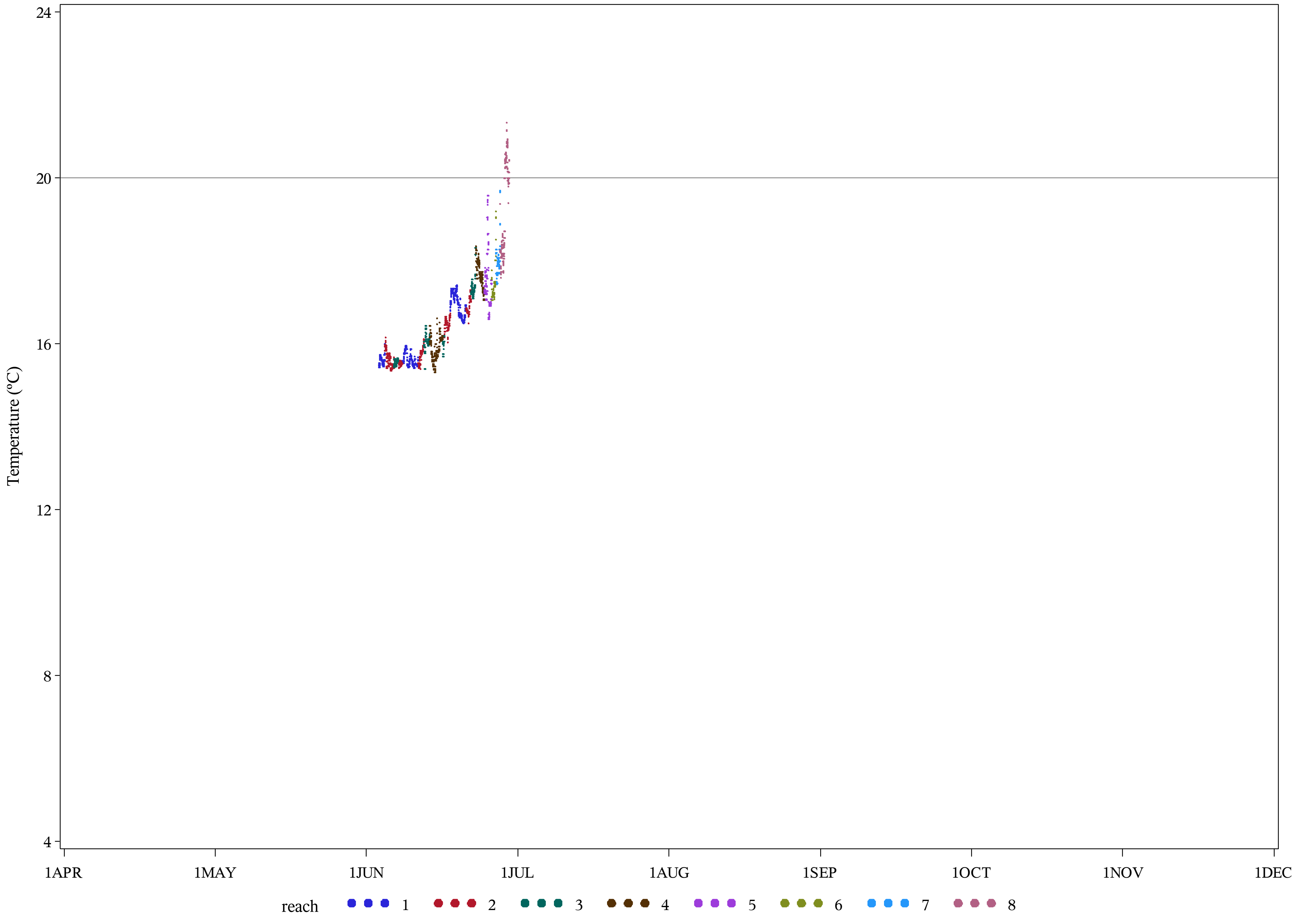

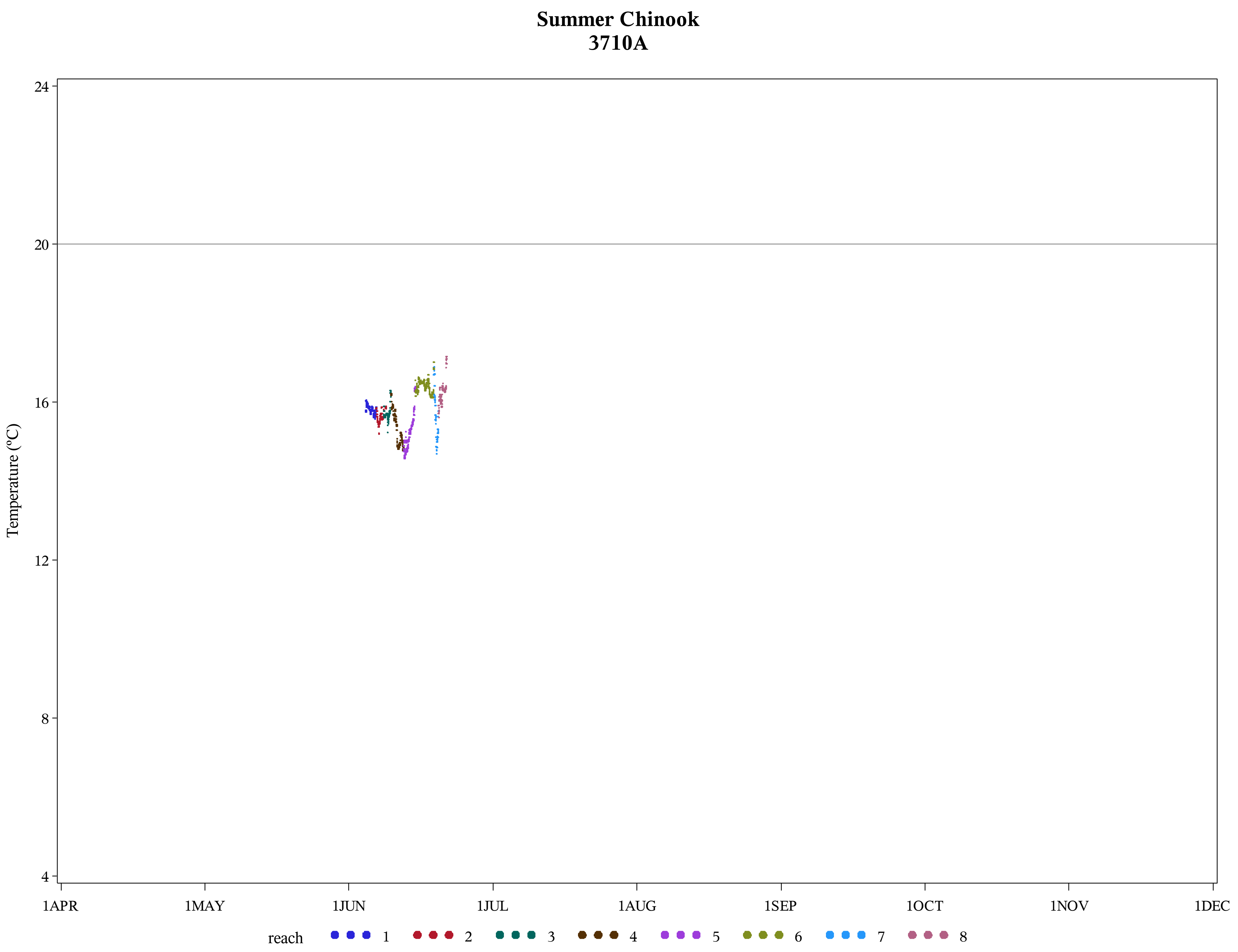

Summer Chinook
3710A
Temperature (°C)
24
20
16
12
8
4
1APR
1MAY
1JUN
1JUL
1AUG
1SEP
1OCT
1NOV
1DEC
reach
1
2
3
4
5
6
7
8

Summer Chinook
3714A

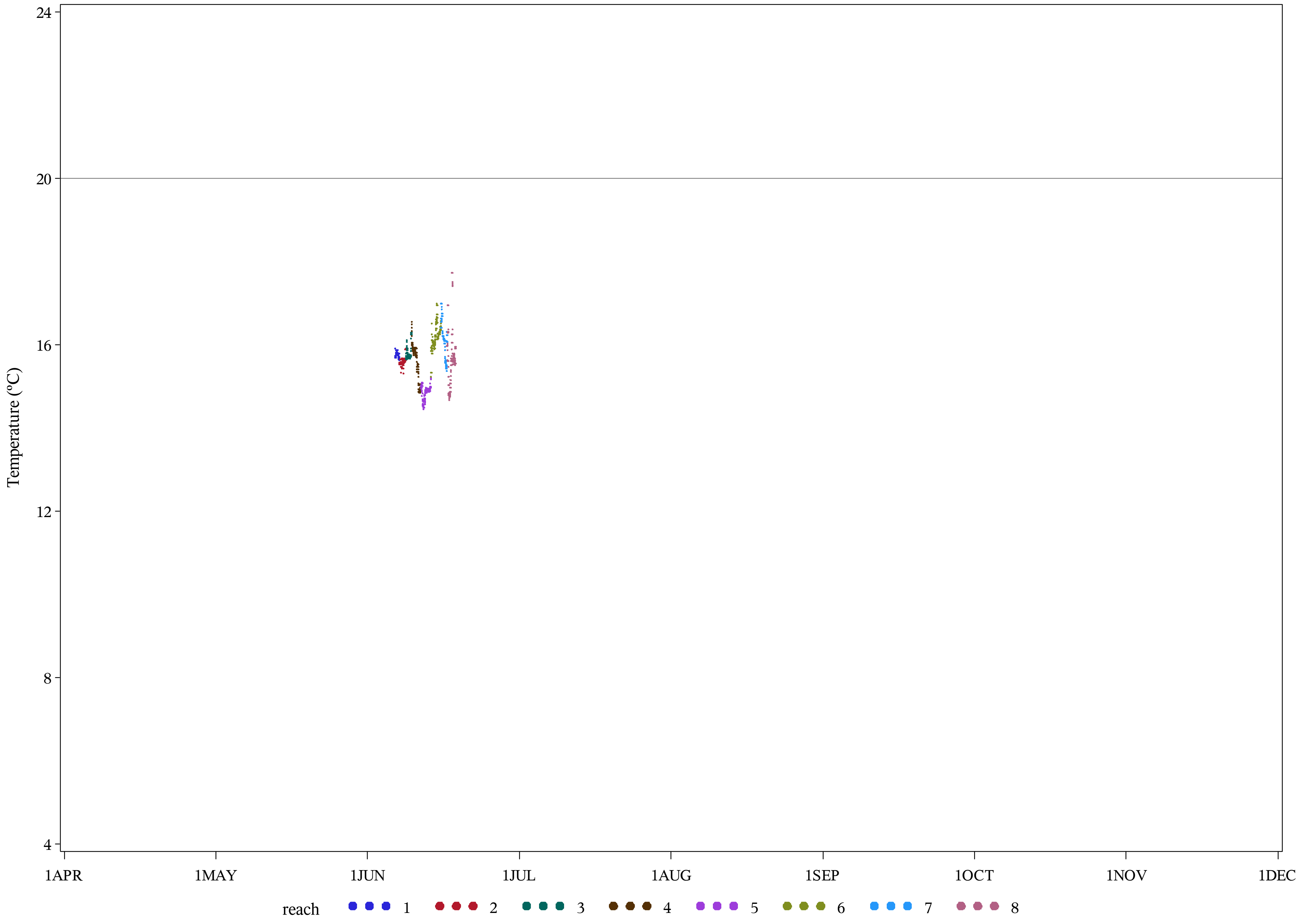

Summer Chinook
3724A

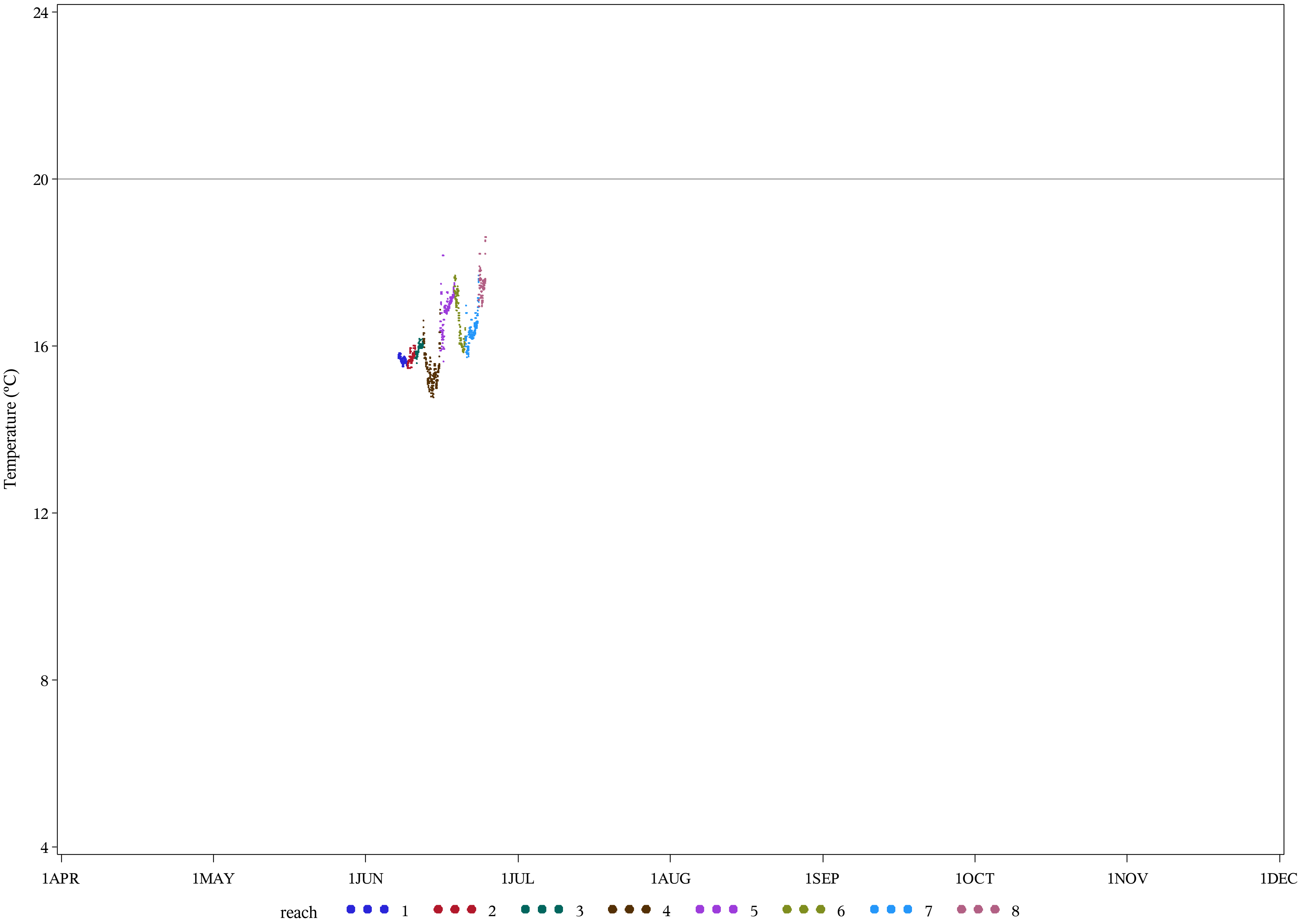

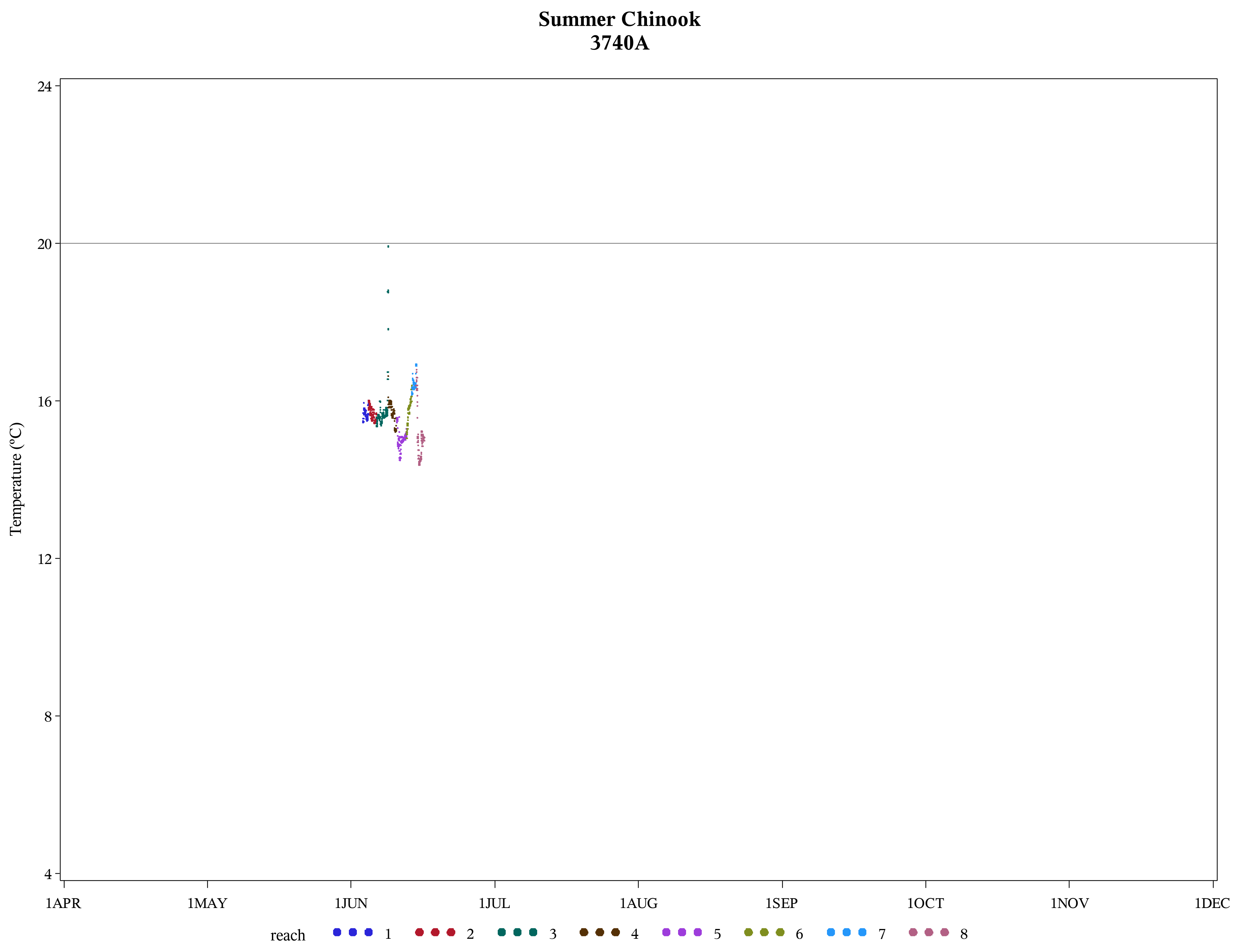
Summer Chinook
3740A
Temperature (°C)
24
20
16
12
8
4
1APR
1MAY
1JUN
1JUL
1AUG
1SEP
1OCT
1NOV
1DEC
reach
1
2
3
4
5
6
7
8

Summer Chinook
3742A

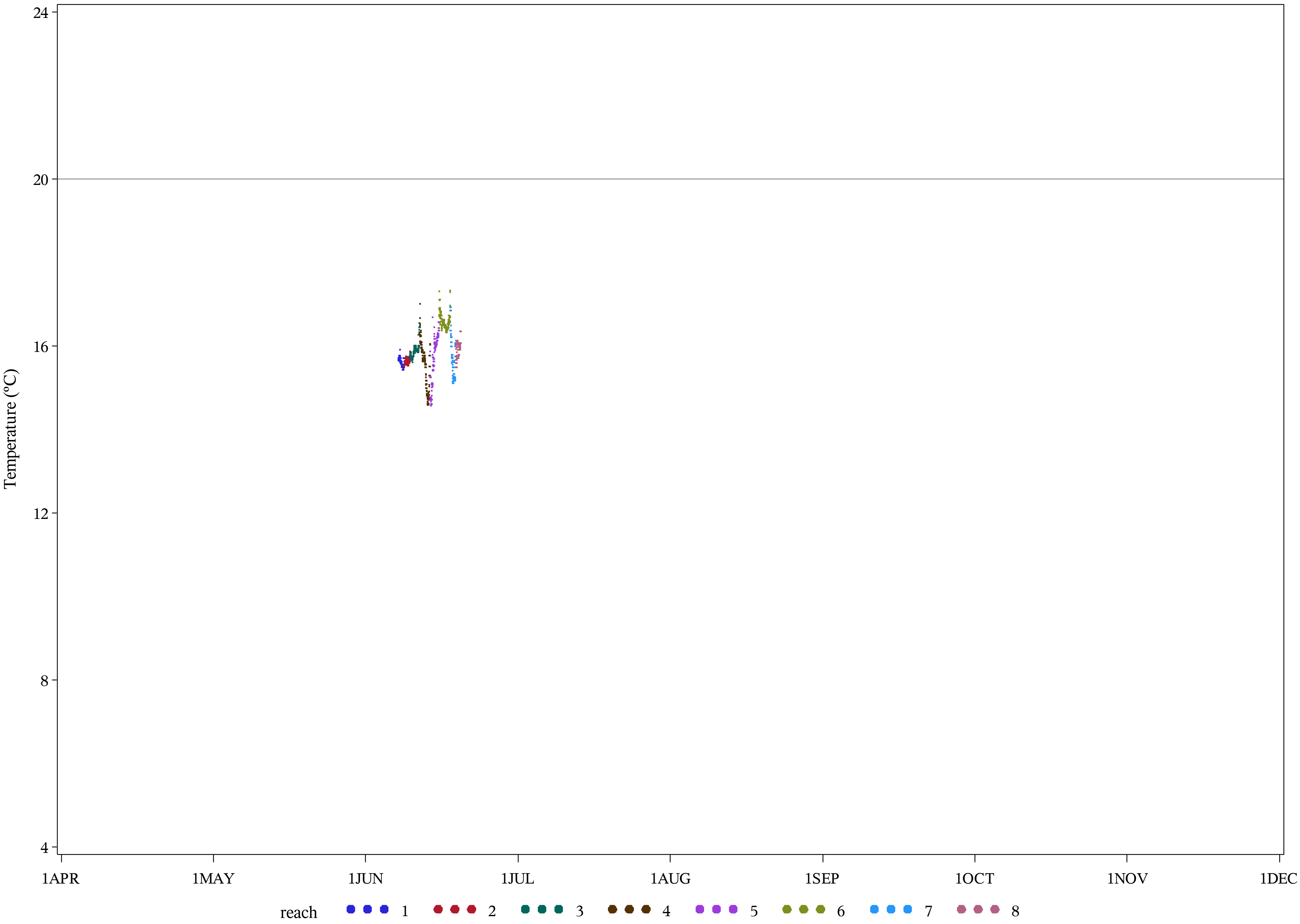

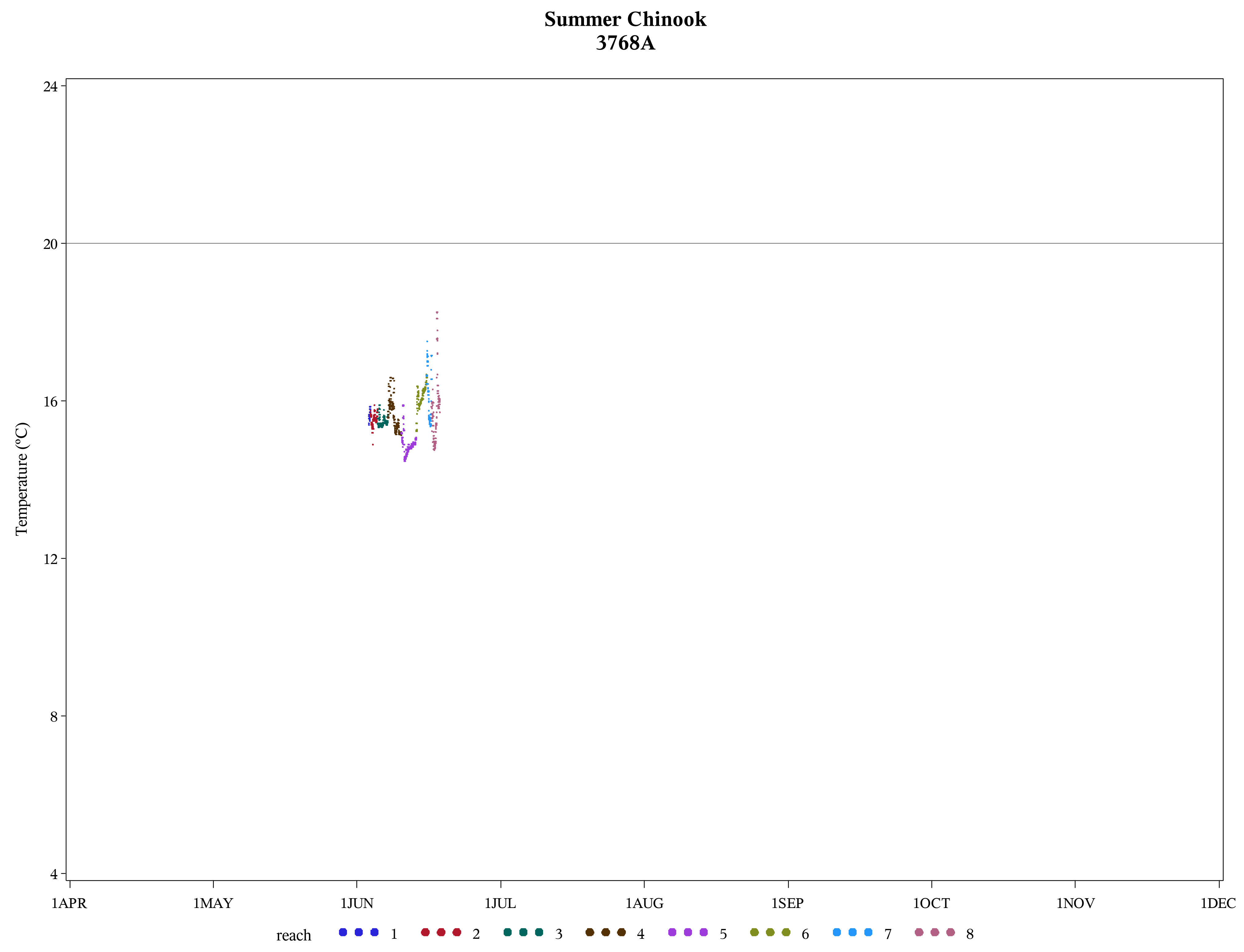
Summer Chinook
3768A
Temperature (°C)
24
20
16
12
8
4
1APR
1MAY
1JUN
1JUL
1AUG
1SEP
1OCT
1NOV
1DEC
reach
1
2
3
4
5
6
7
8

Summer Chinook
3773A

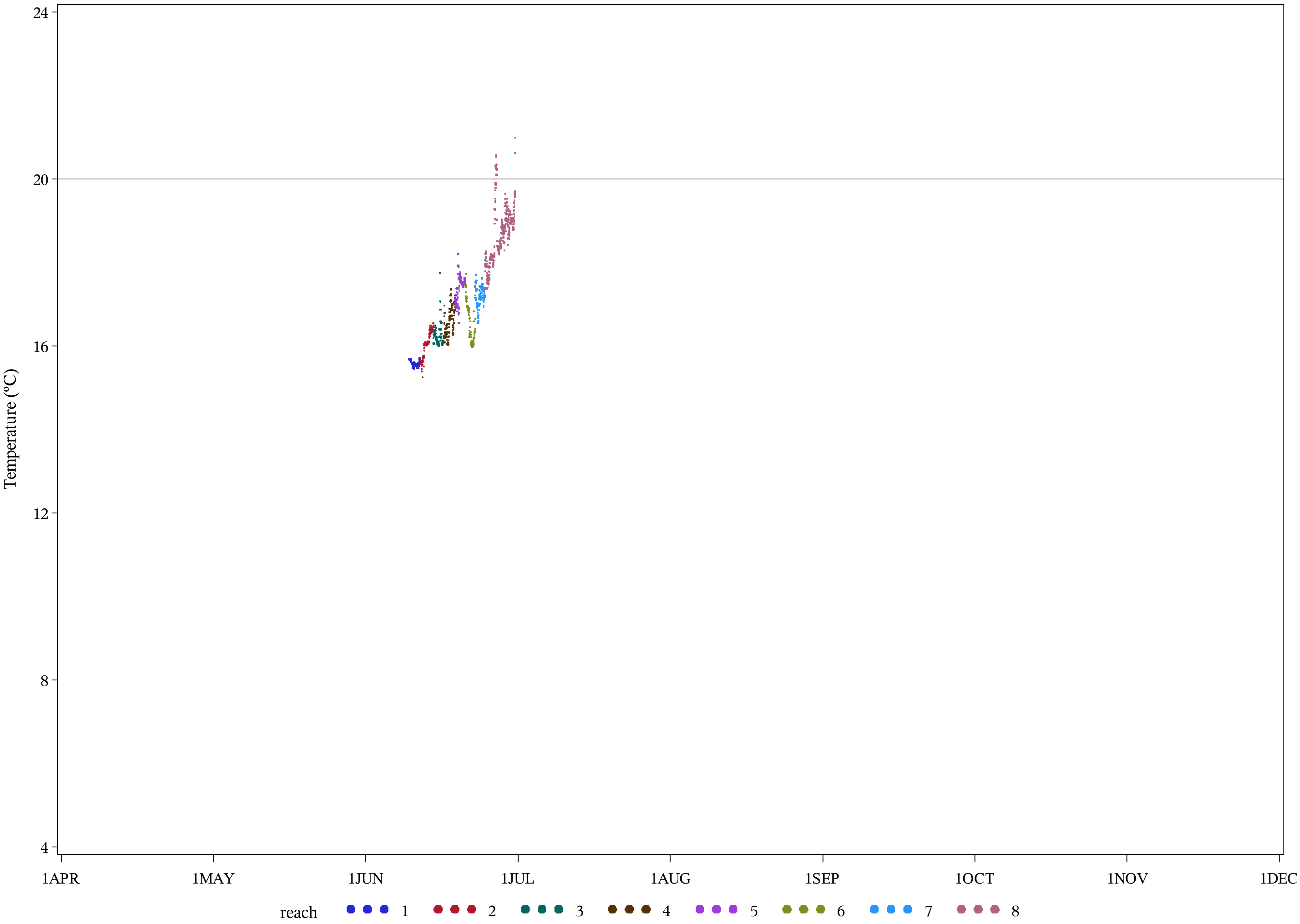

Summer Chinook
3829A

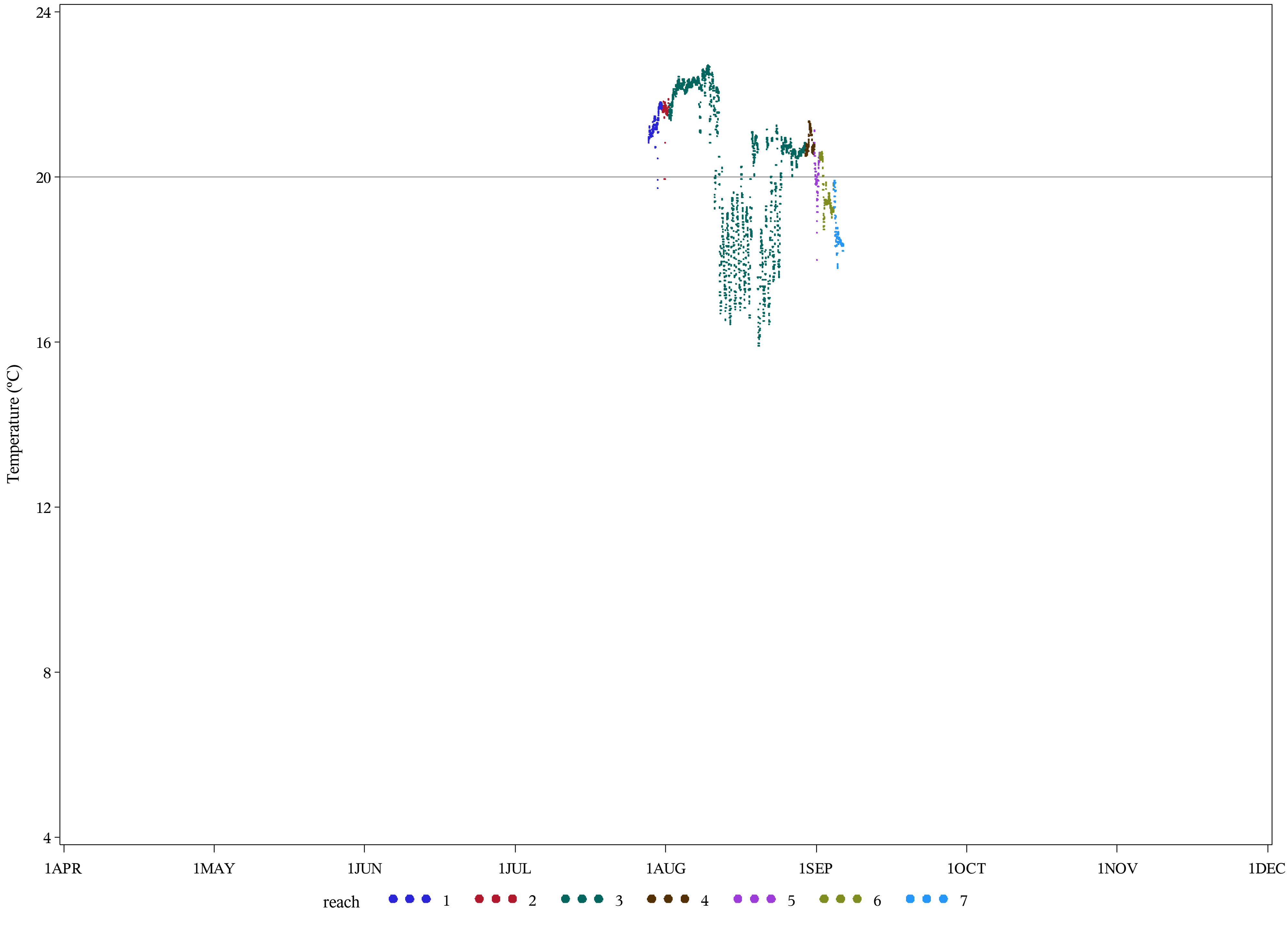

Supplement: S1 Appendix — Graphs show individual body-temperature histories, color coded by river reach. The run (spring Chinook salmon, summer Chinook salmon, fall Chinook salmon, steelhead) and RDST number are shown for each fish. (PDF) [file pone.0204274.s001.pdf]
