# Supplementary material for: Thermal exposure of adult Chinook salmon and steelhead: Diverse behavioral strategies in a large and warming river system
Source: PLoS One. 2018 Sep 21;13(9):e0204274. doi: 10.1371/journal.pone.0204274 (PMC6150539; doi:10.1371/journal.pone.0204274)

Fall Chinook
2660B

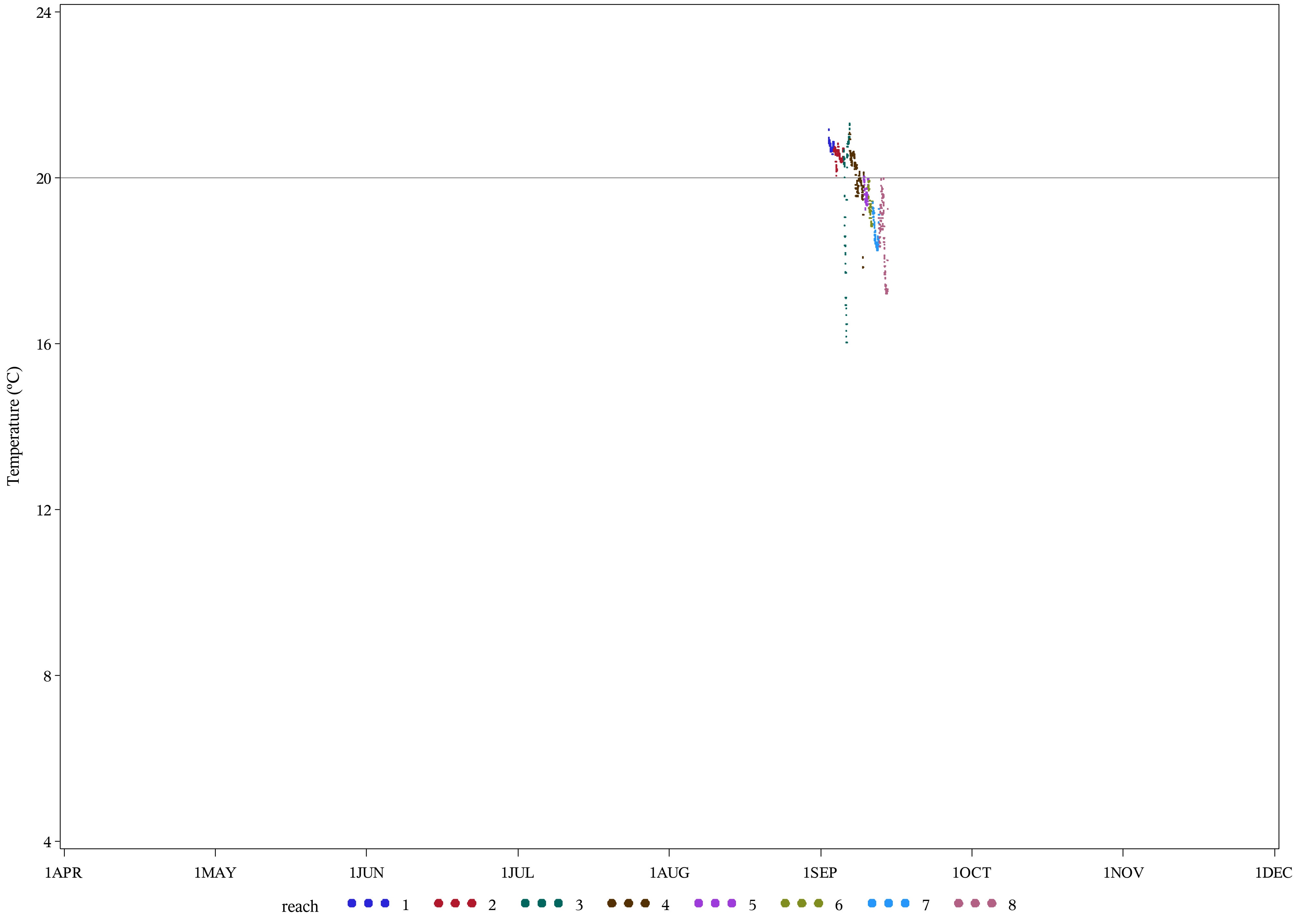

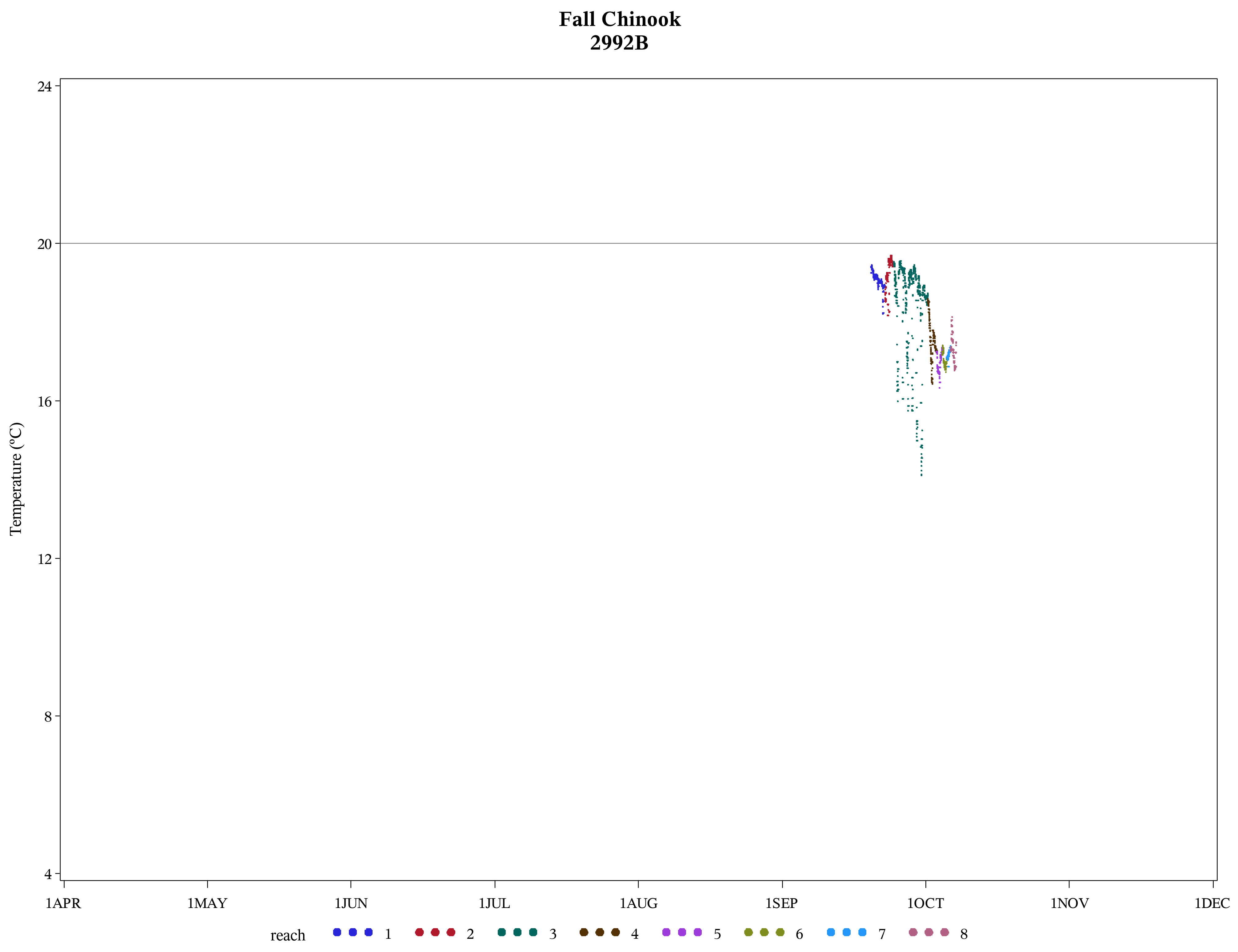
Fall Chinook
2992B
Temperature (°C)
24
20
16
12
8
4
1APR
1MAY
1JUN
1JUL
1AUG
1SEP
1OCT
1NOV
1DEC
reach
1
2
3
4
5
6
7
8

Fall Chinook
3078B

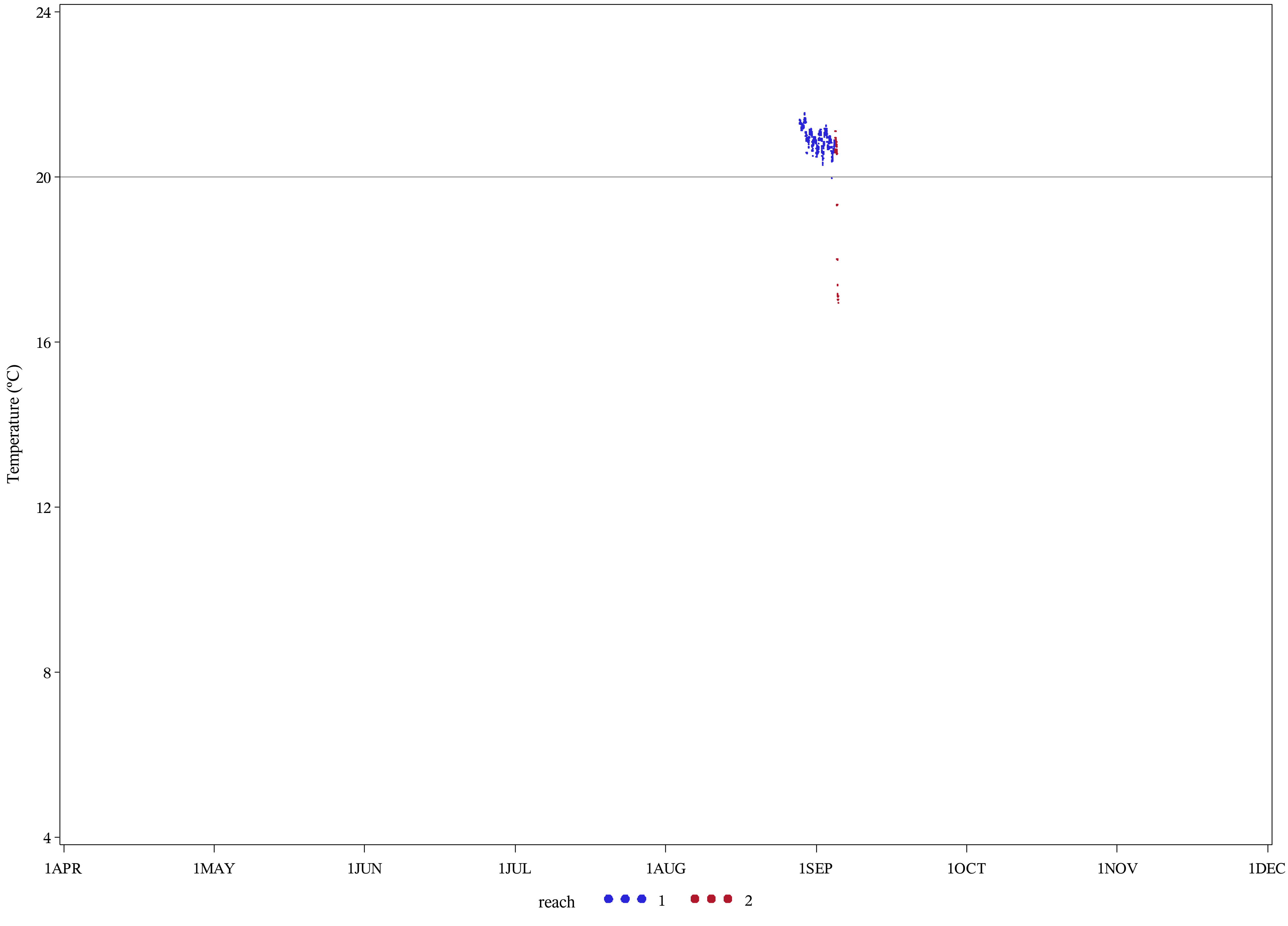

Fall Chinook
3109B

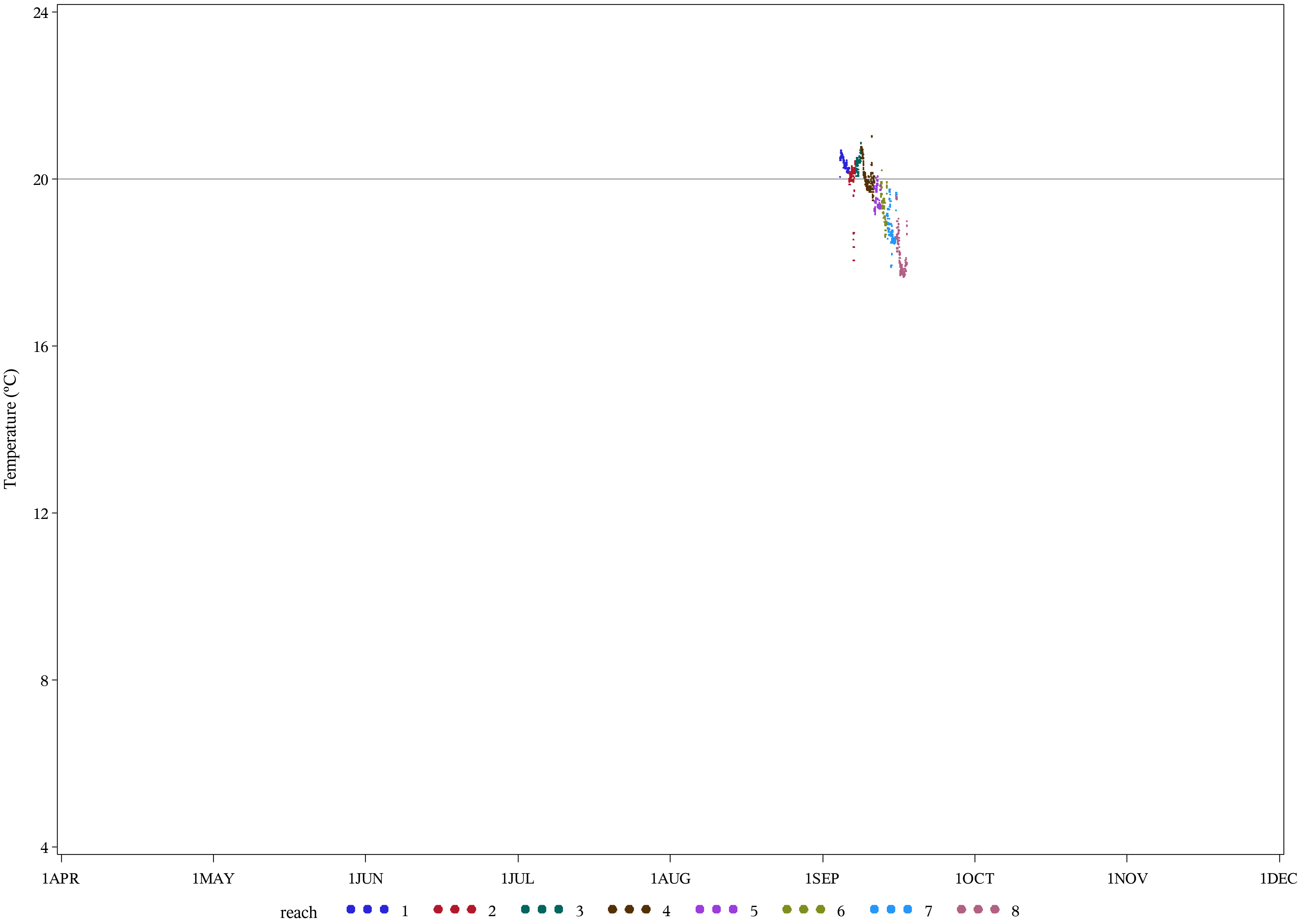

Fall Chinook
3761B

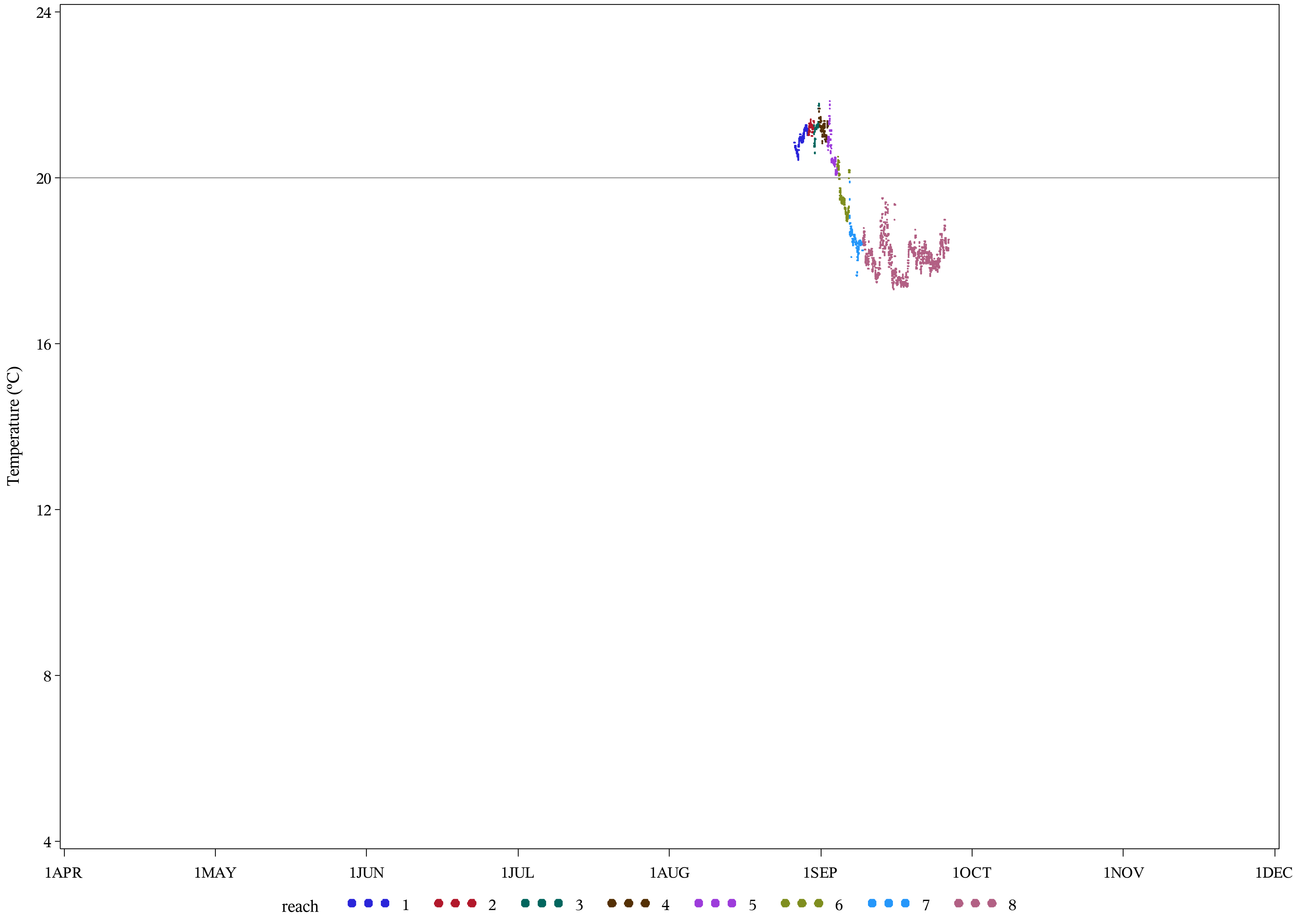

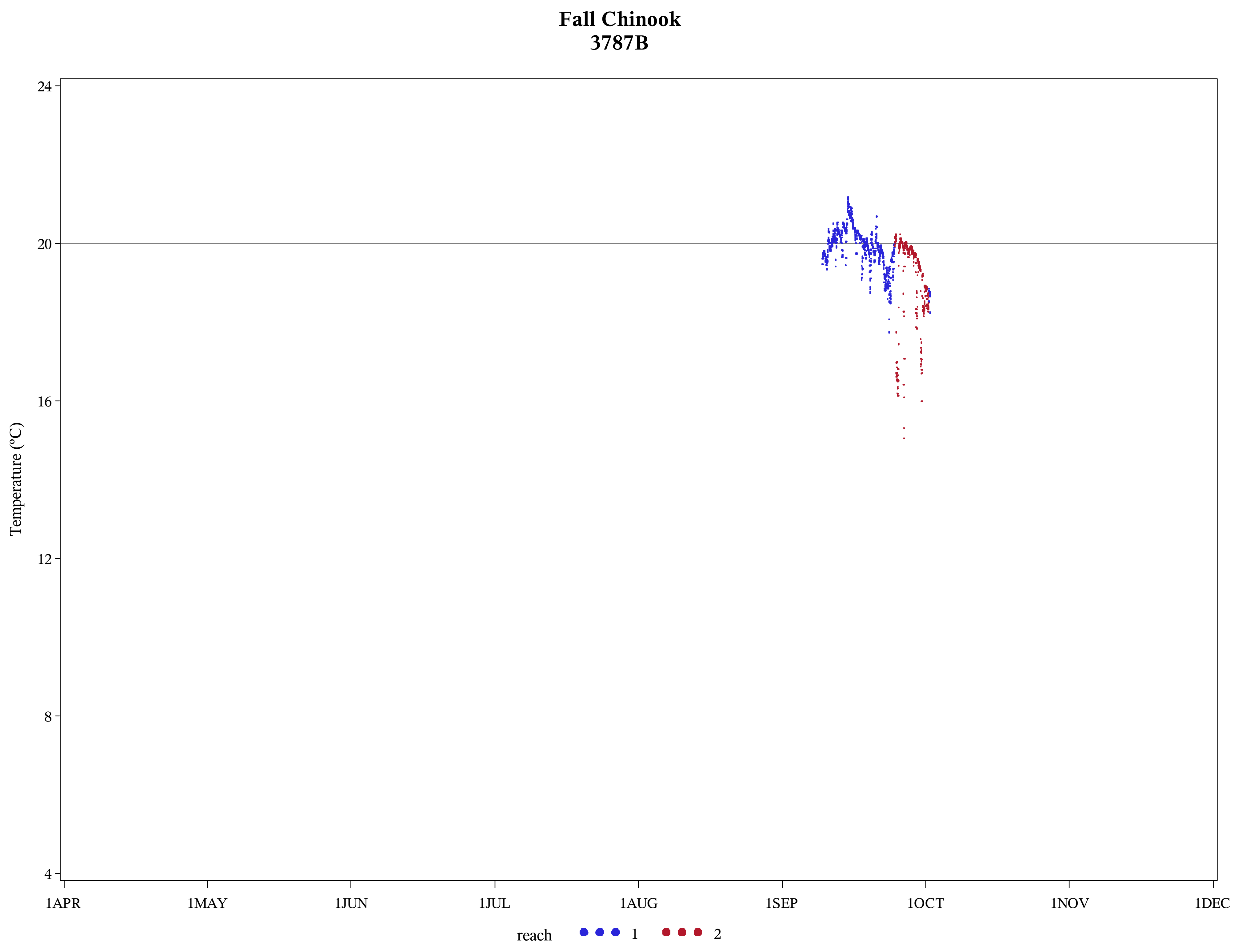

Fall Chinook
3787B
Temperature (°C)
24
20
16
12
8
4
1APR
1MAY
1JUN
1JUL
1AUG
1SEP
1OCT
1NOV
1DEC
reach
1
2

Fall Chinook
3805B

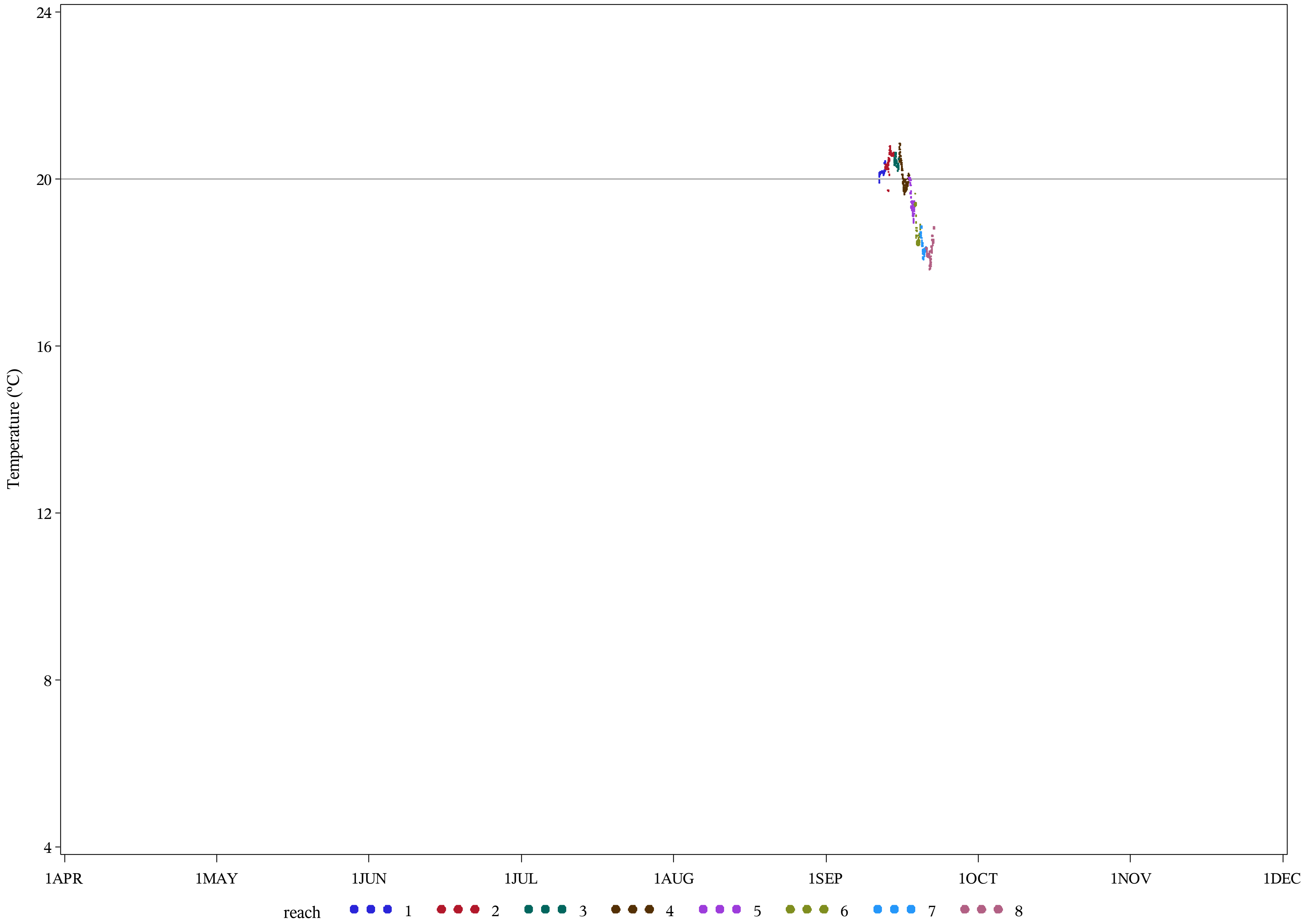

Fall Chinook
4692A

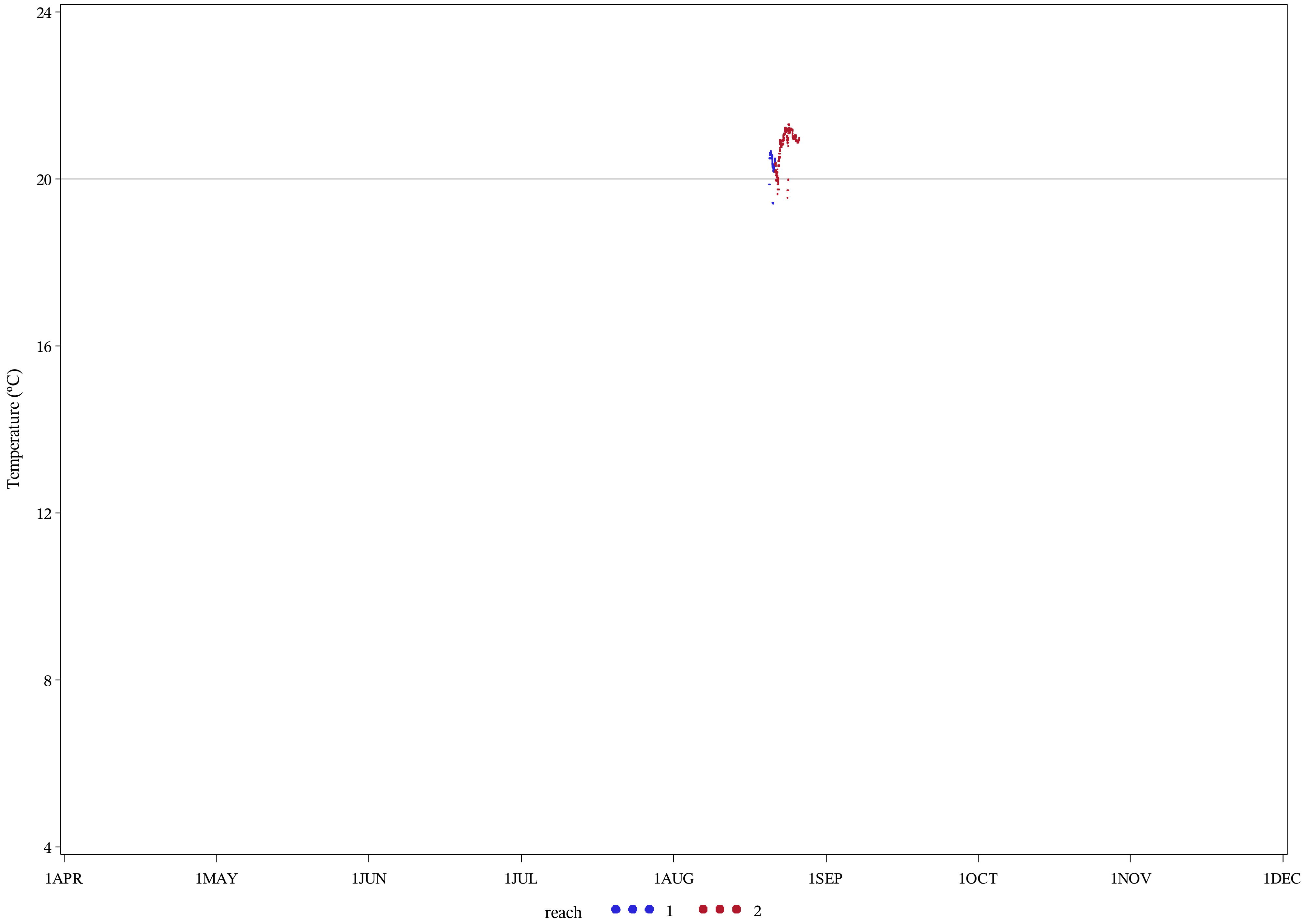

Fall Chinook
4754B

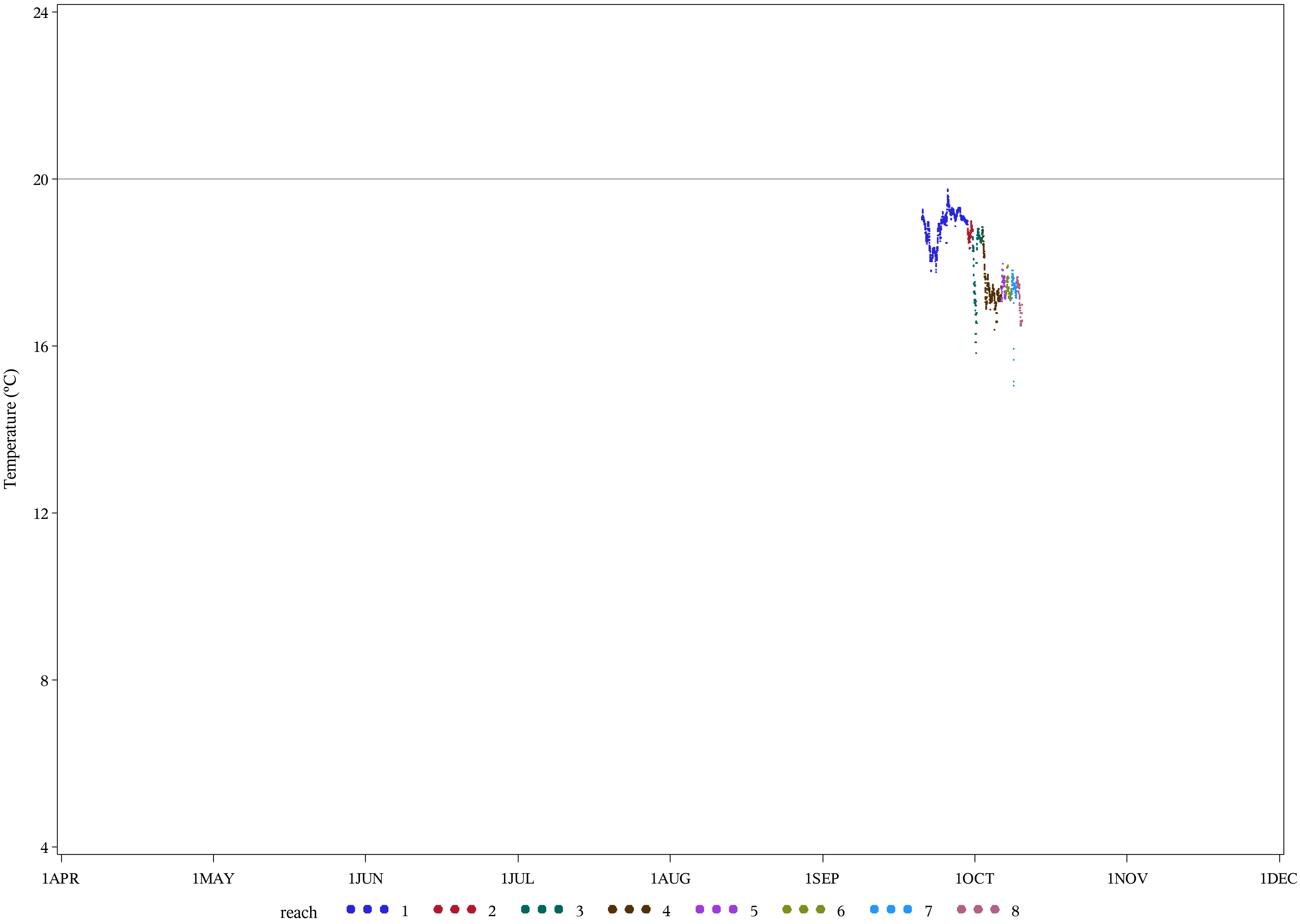

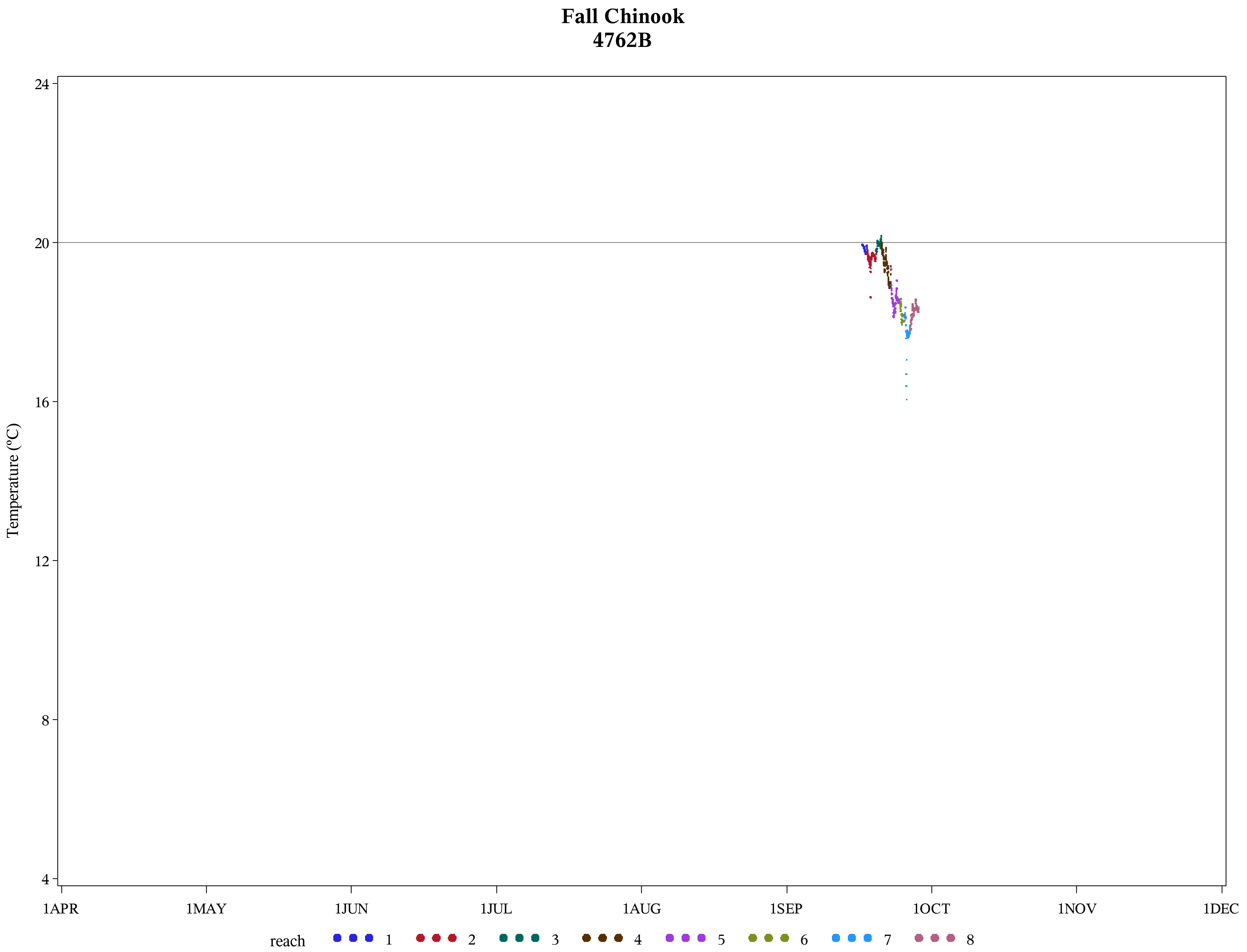

Fall Chinook
4762B
Temperature (°C)
24
20
16
12
8
4
1APR
1MAY
1JUN
1JUL
1AUG
1SEP
1OCT
1NOV
1DEC
reach
1
2
3
4
5
6
7
8

Fall Chinook
4819B

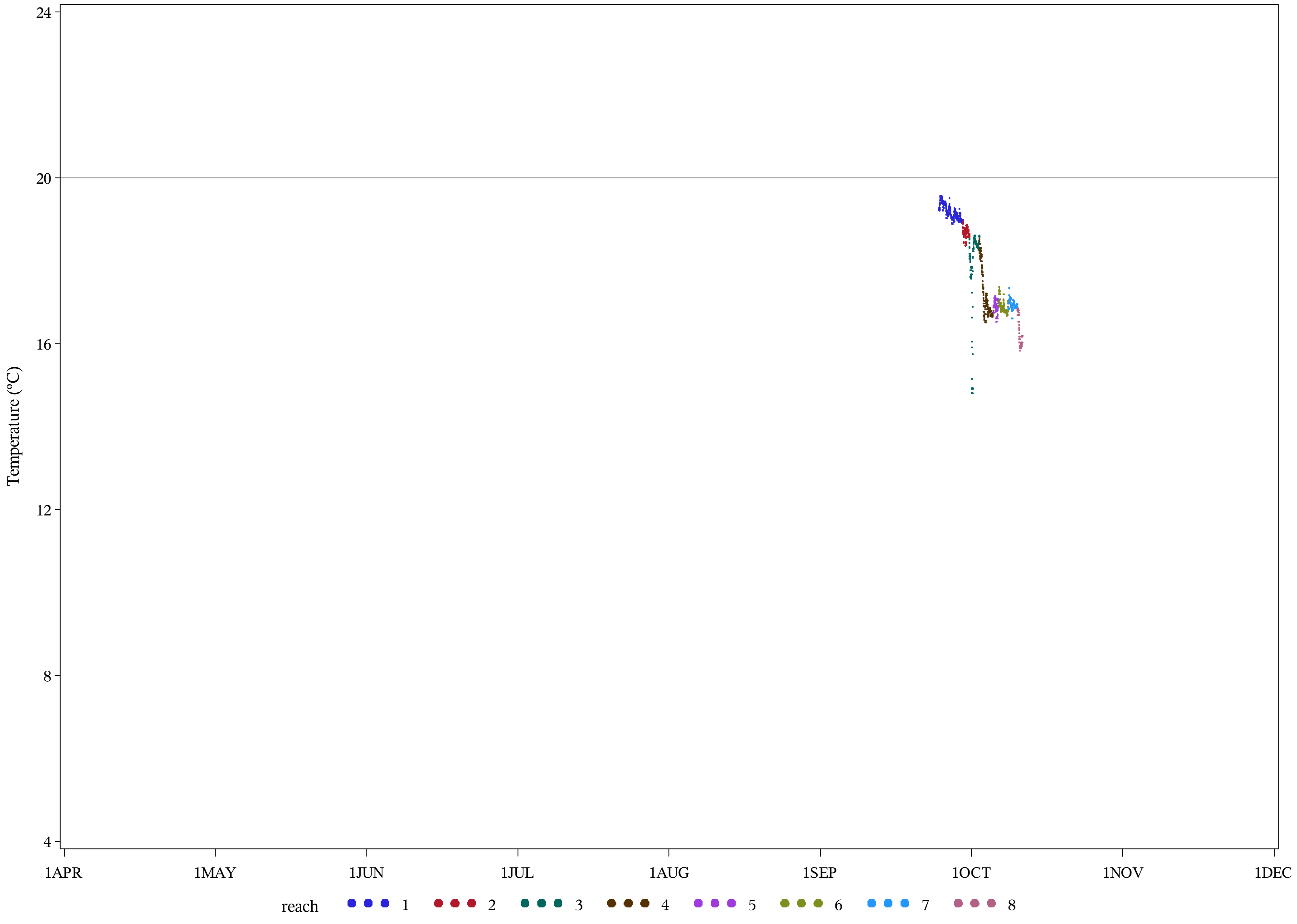

Fall Chinook
4829B

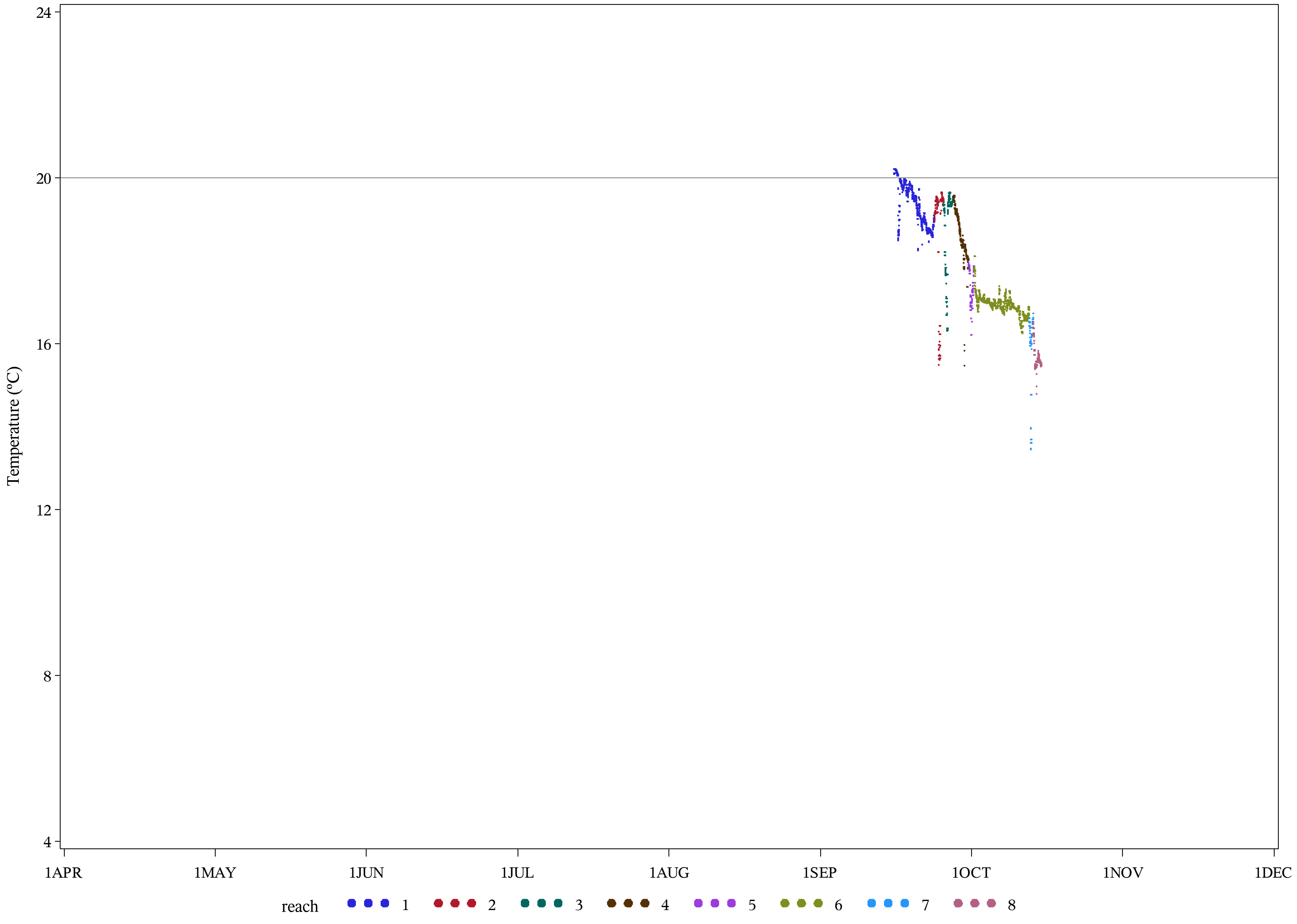

Fall Chinook
4869B

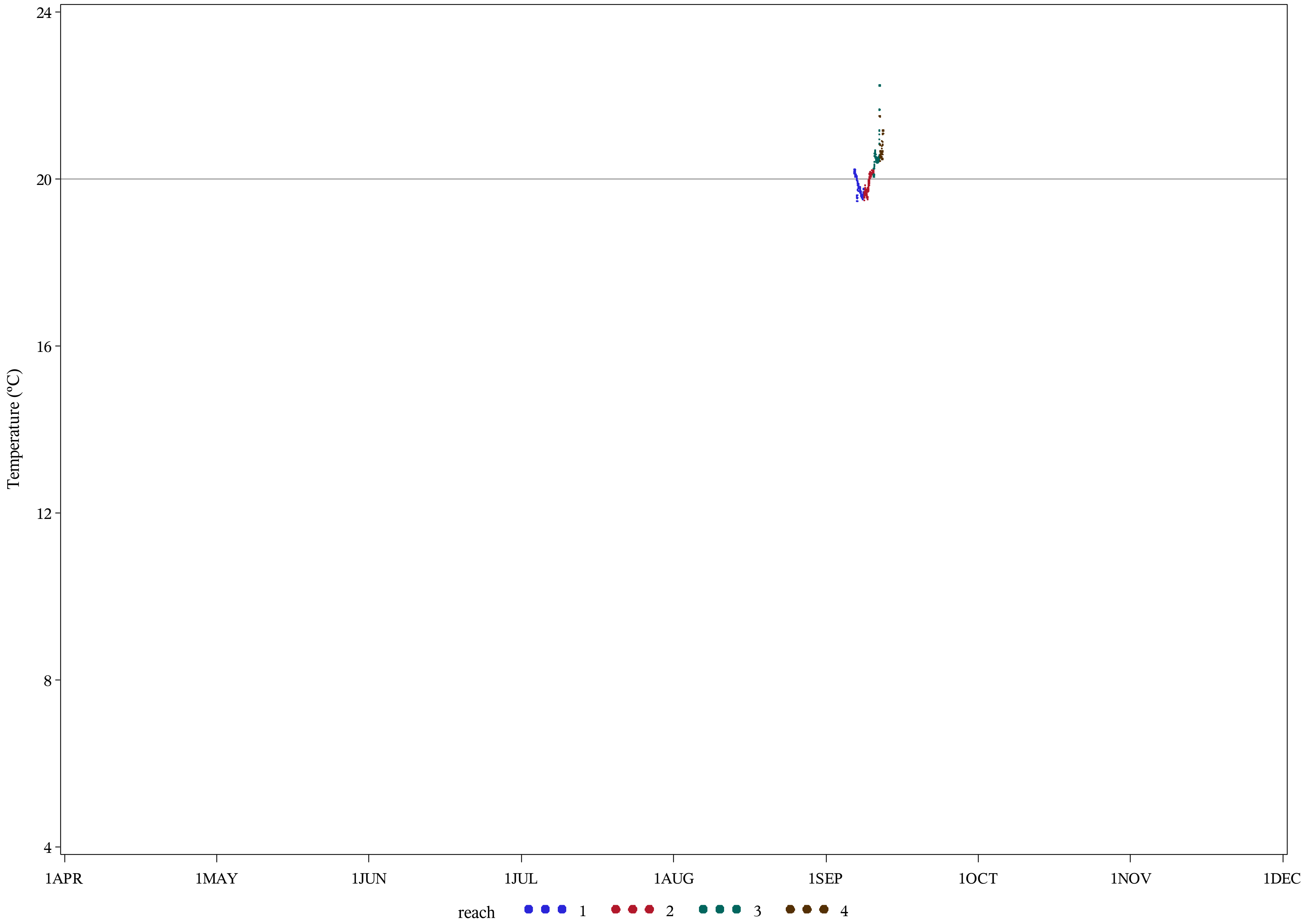

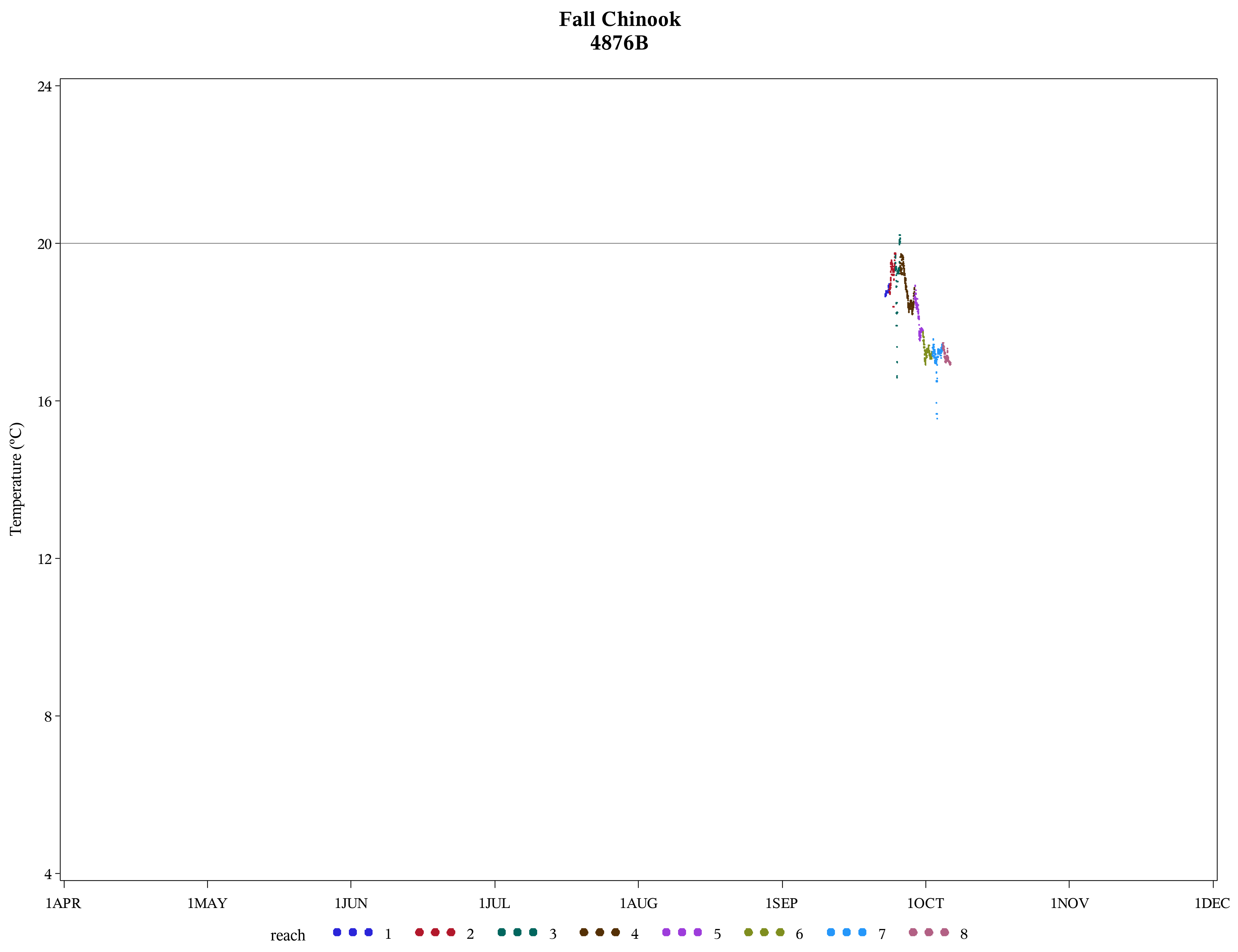
Fall Chinook
4876B
Temperature (°C)
24
20
16
12
8
4
1APR
1MAY
1JUN
1JUL
1AUG
1SEP
1OCT
1NOV
1DEC
reach
1
2
3
4
5
6
7
8

Fall Chinook
4877B

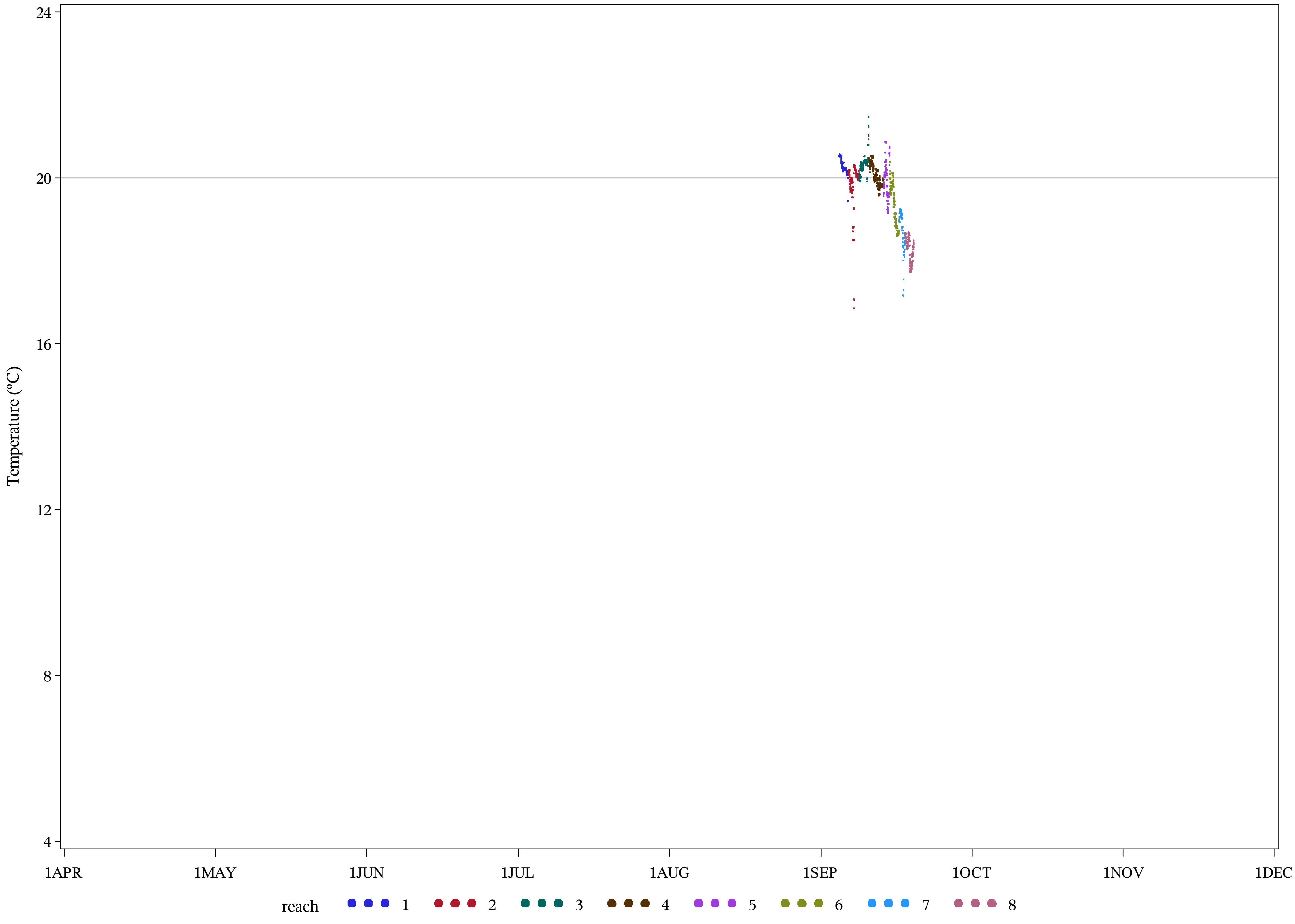

Fall Chinook
4878B

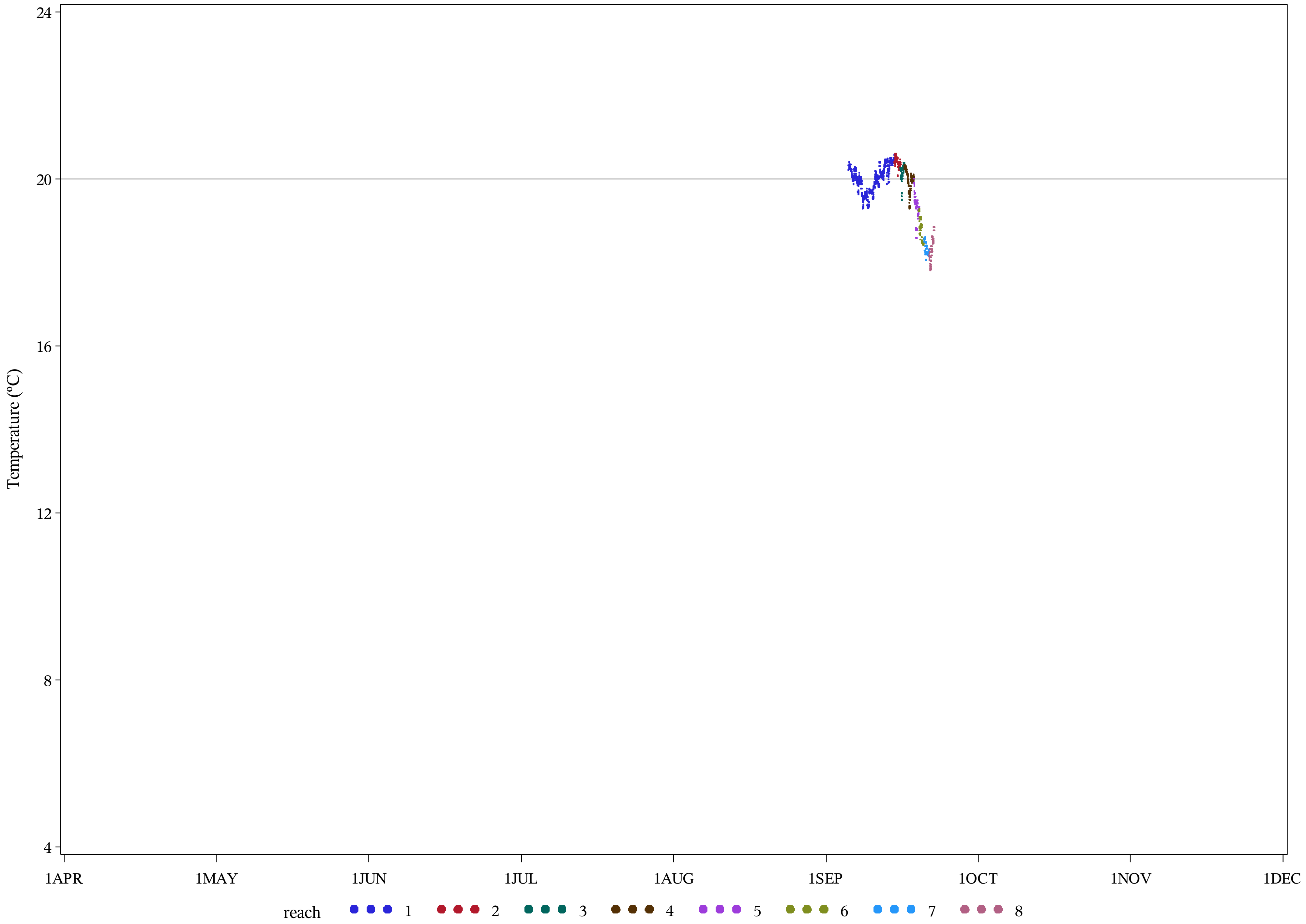

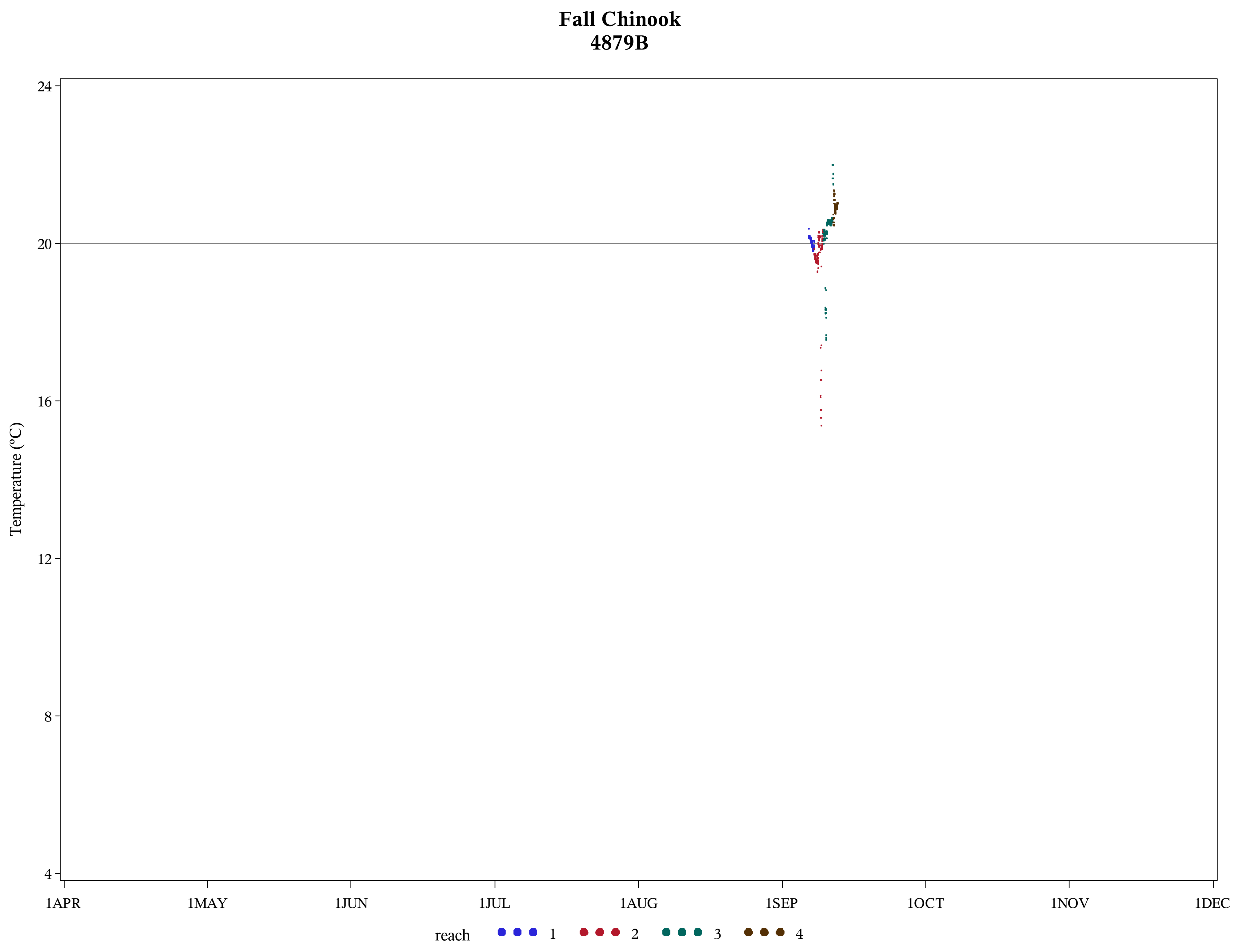

Fall Chinook
4879B
Temperature (°C)
24
20
16
12
8
4
1APR
1MAY
1JUN
1JUL
1AUG
1SEP
1OCT
1NOV
1DEC
reach
1
2
3
4

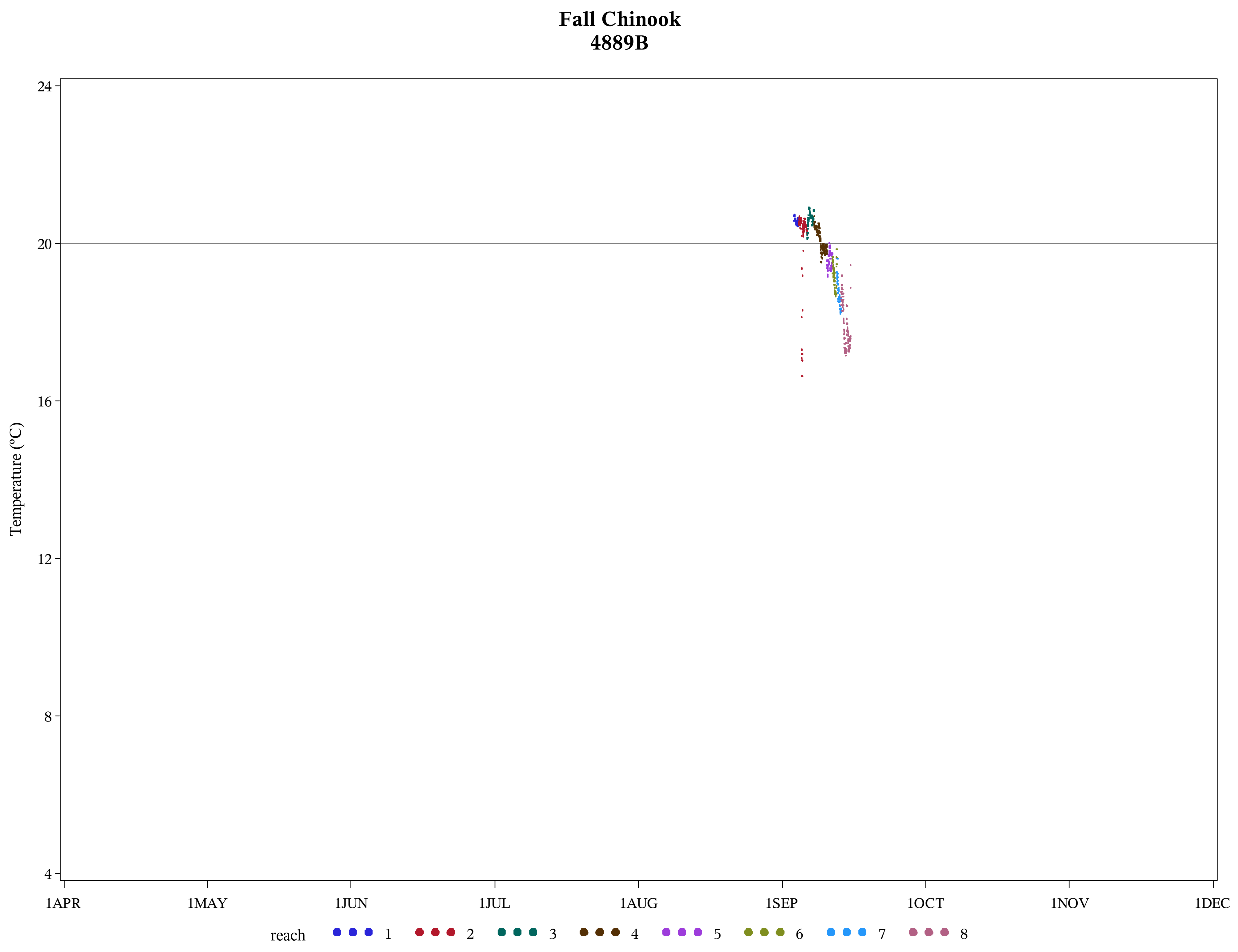

Fall Chinook
4889B
Temperature (°C)
24
20
16
12
8
4
1APR
1MAY
1JUN
1JUL
1AUG
1SEP
1OCT
1NOV
1DEC
reach
1
2
3
4
5
6
7
8

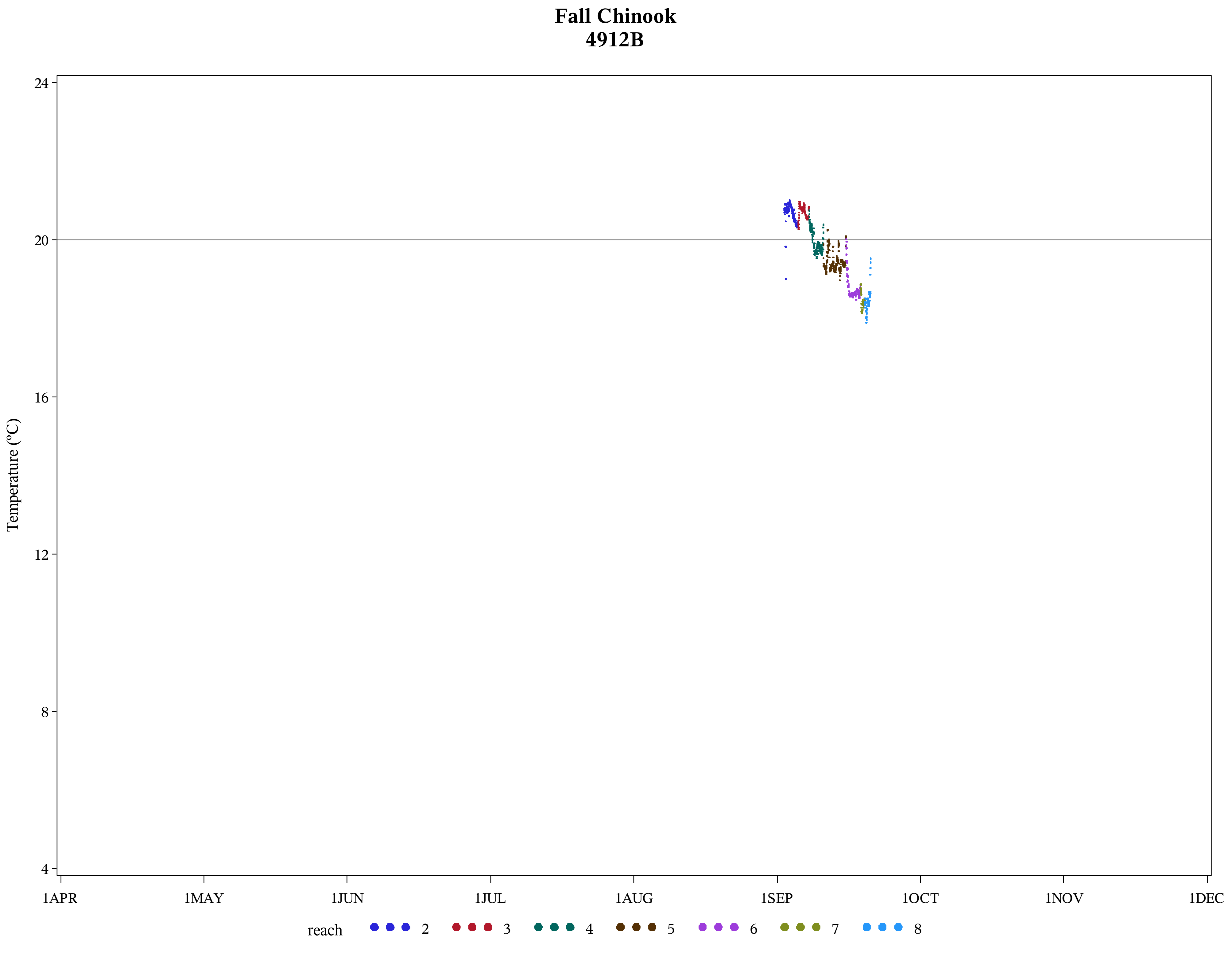
Fall Chinook
4912B
Temperature (°C)
24
20
16
12
8
4
1APR
1MAY
1JUN
1JUL
1AUG
1SEP
1OCT
1NOV
1DEC
reach
2
3
4
5
6
7
8

Fall Chinook
4972B

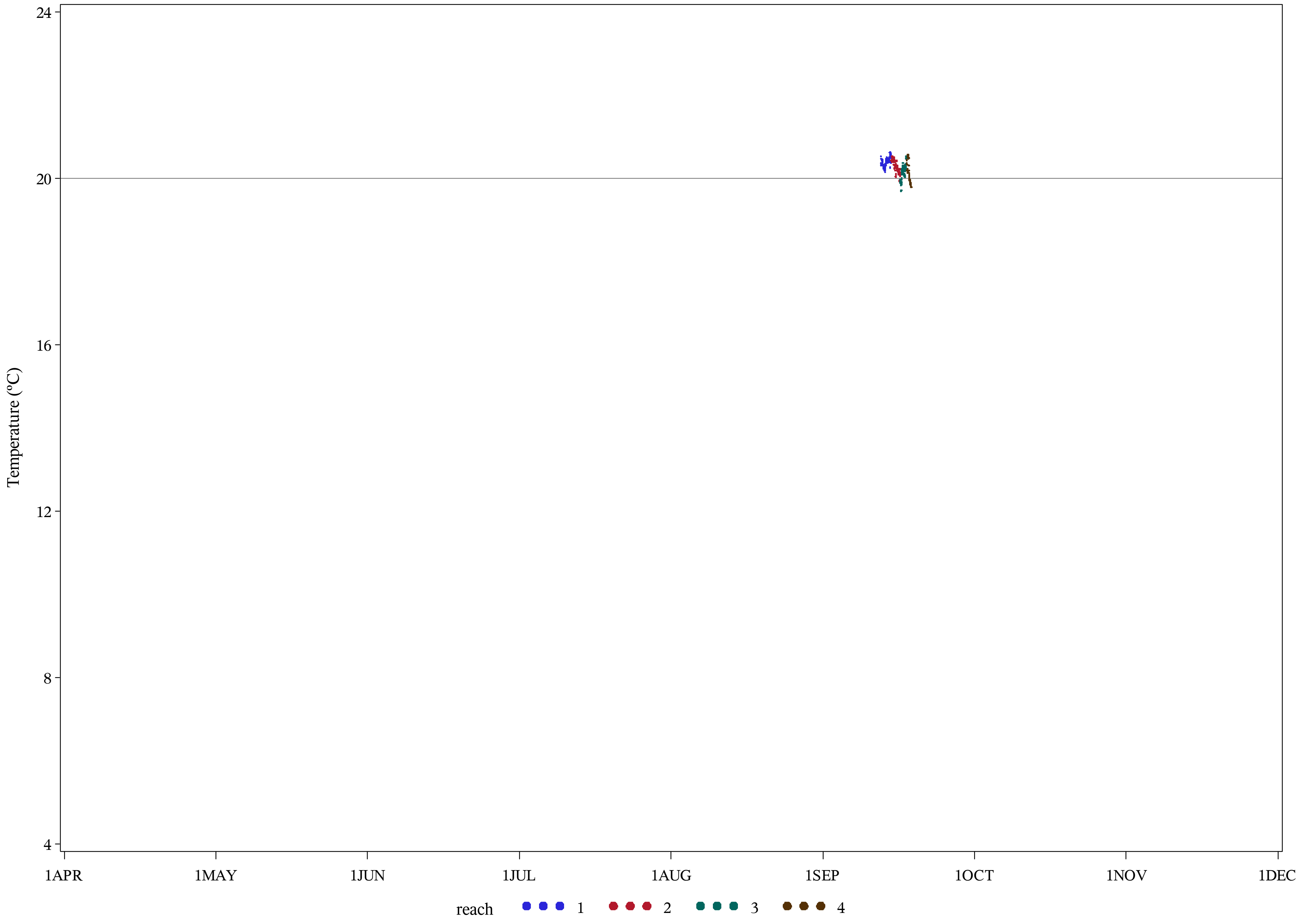

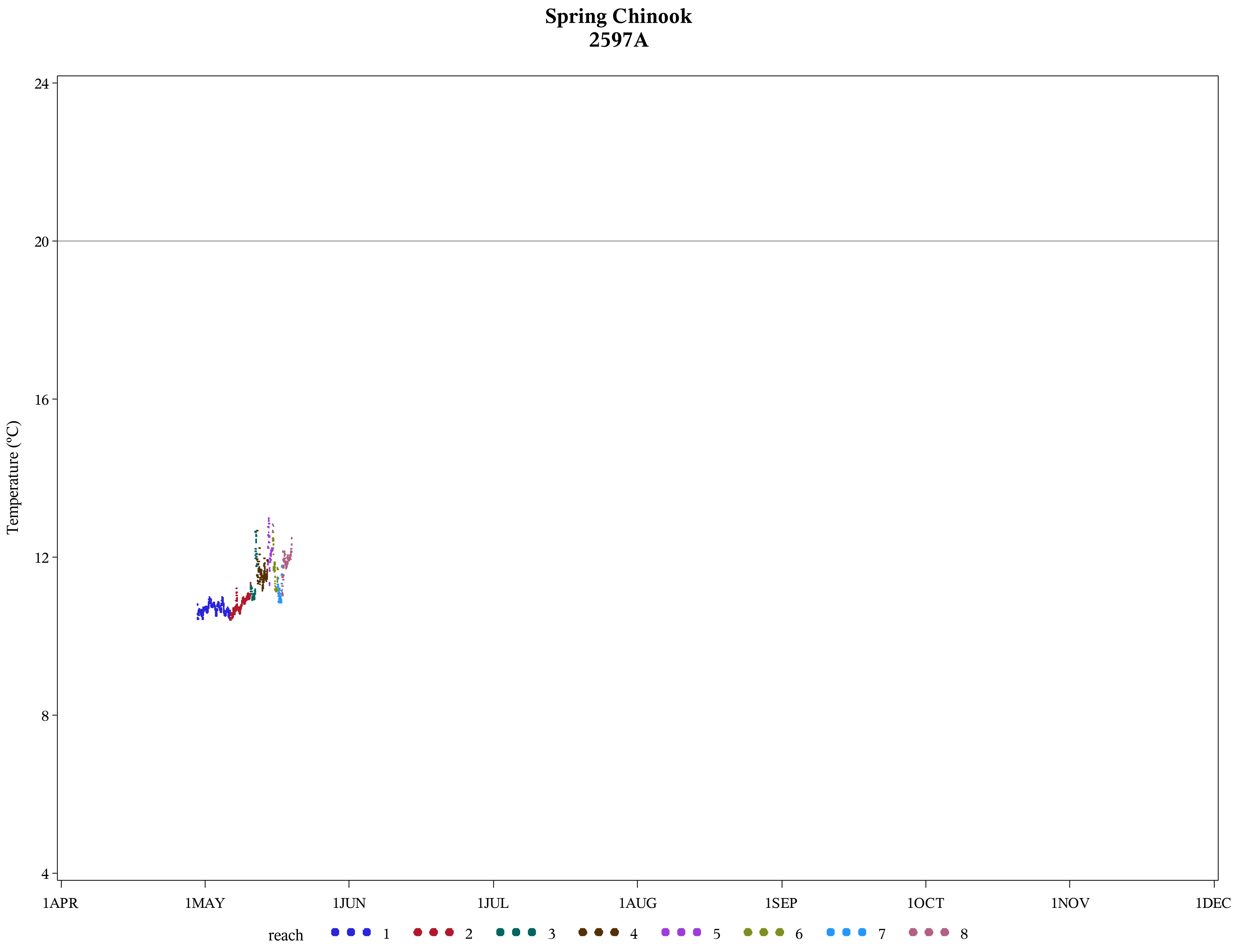

Spring Chinook
2597A
Temperature (°C)
24
20
16
12
8
4
1APR
1MAY
1JUN
1JUL
1AUG
1SEP
1OCT
1NOV
1DEC
reach
1
2
3
4
5
6
7
8

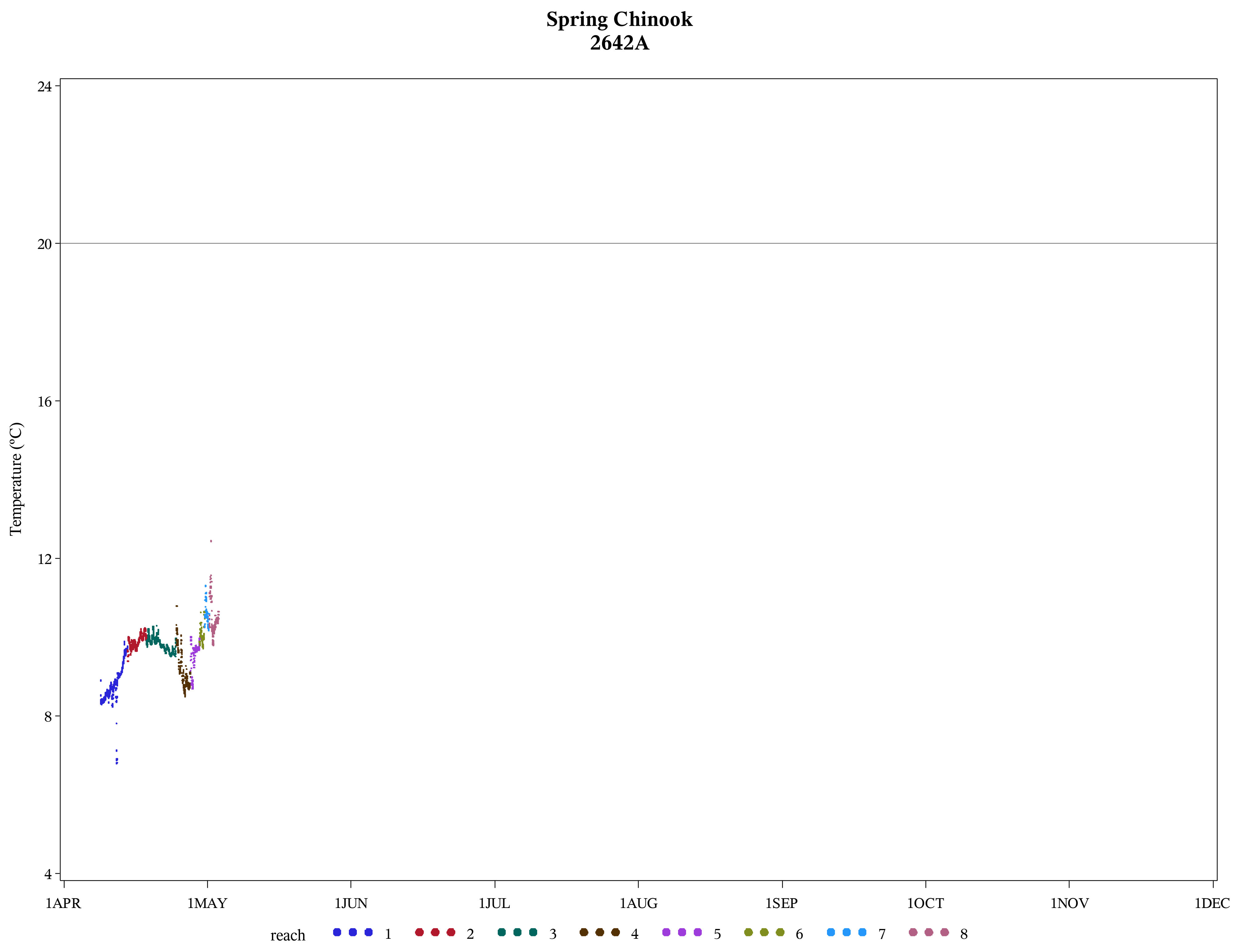

Spring Chinook
2642A
Temperature (°C)
24
20
16
12
8
4
1APR
1MAY
1JUN
1JUL
1AUG
1SEP
1OCT
1NOV
1DEC
reach
1
2
3
4
5
6
7
8

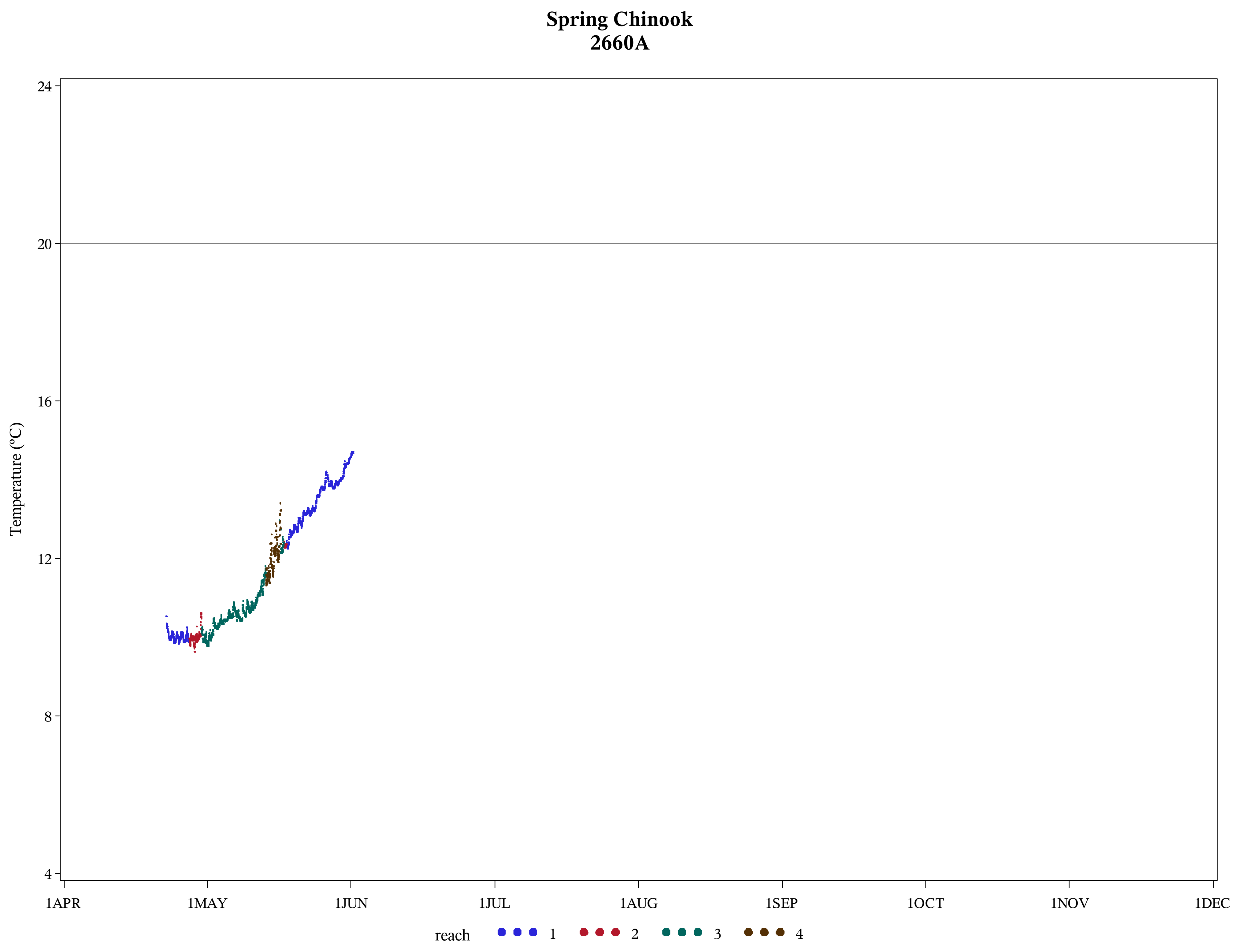
Spring Chinook
2660A
Temperature (°C)
24
20
16
12
8
4
1APR
1MAY
1JUN
1JUL
1AUG
1SEP
1OCT
1NOV
1DEC
reach
1
2
3
4

Spring Chinook
2762A

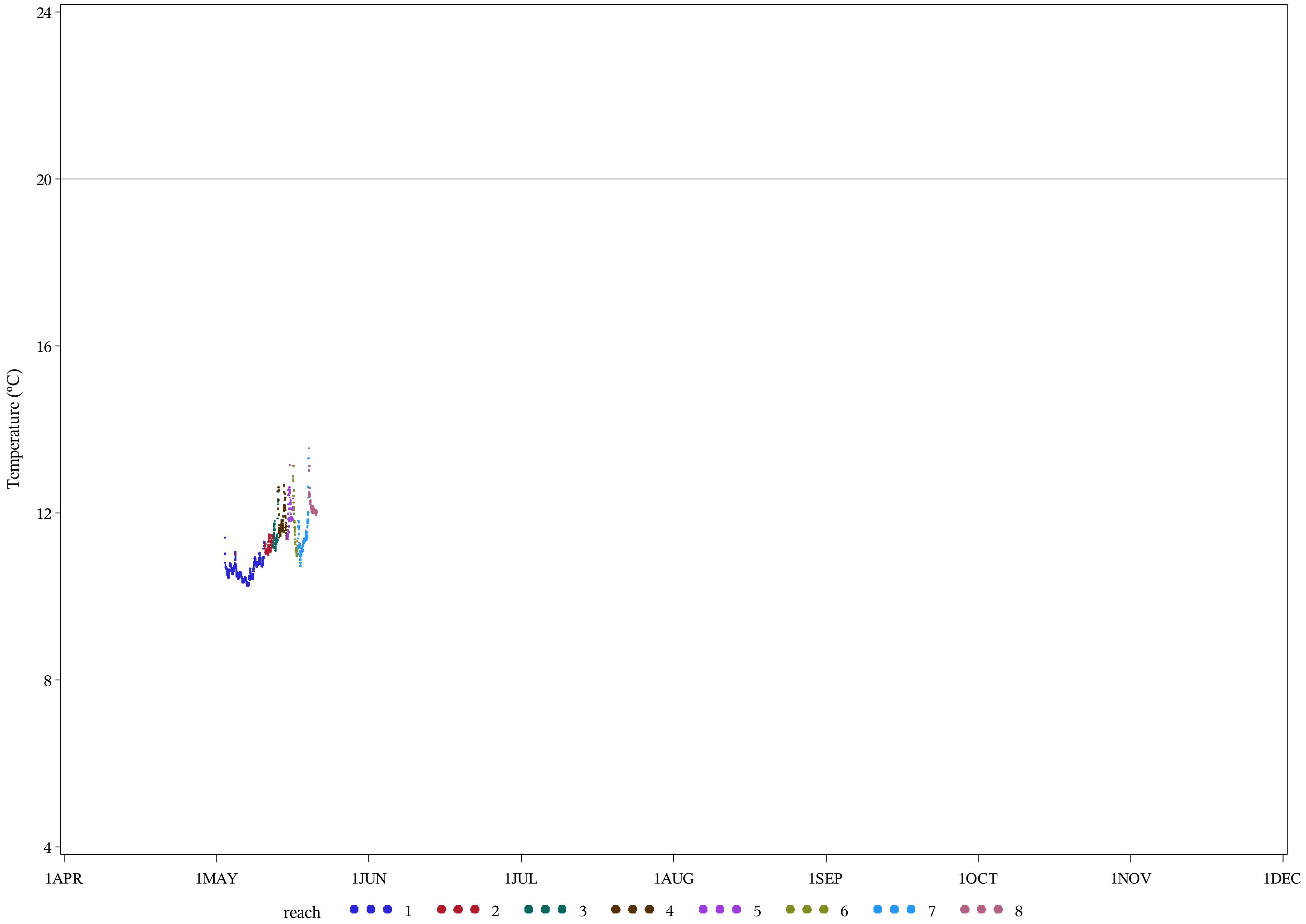

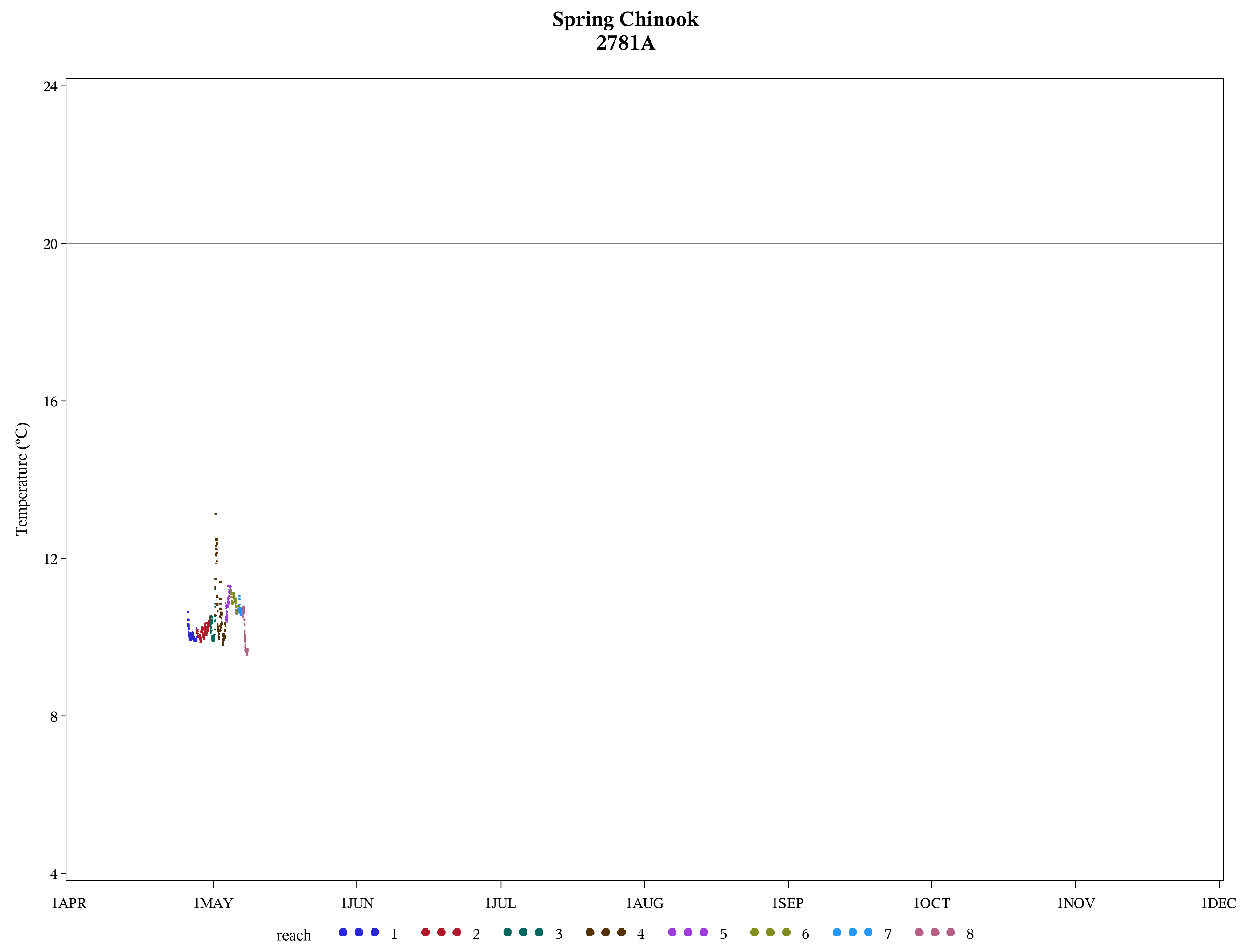

Spring Chinook
2781A
Temperature (°C)
24
20
16
12
8
4
1APR
1MAY
1JUN
1JUL
1AUG
1SEP
1OCT
1NOV
1DEC
reach
1
2
3
4
5
6
7
8

Spring Chinook
2787A

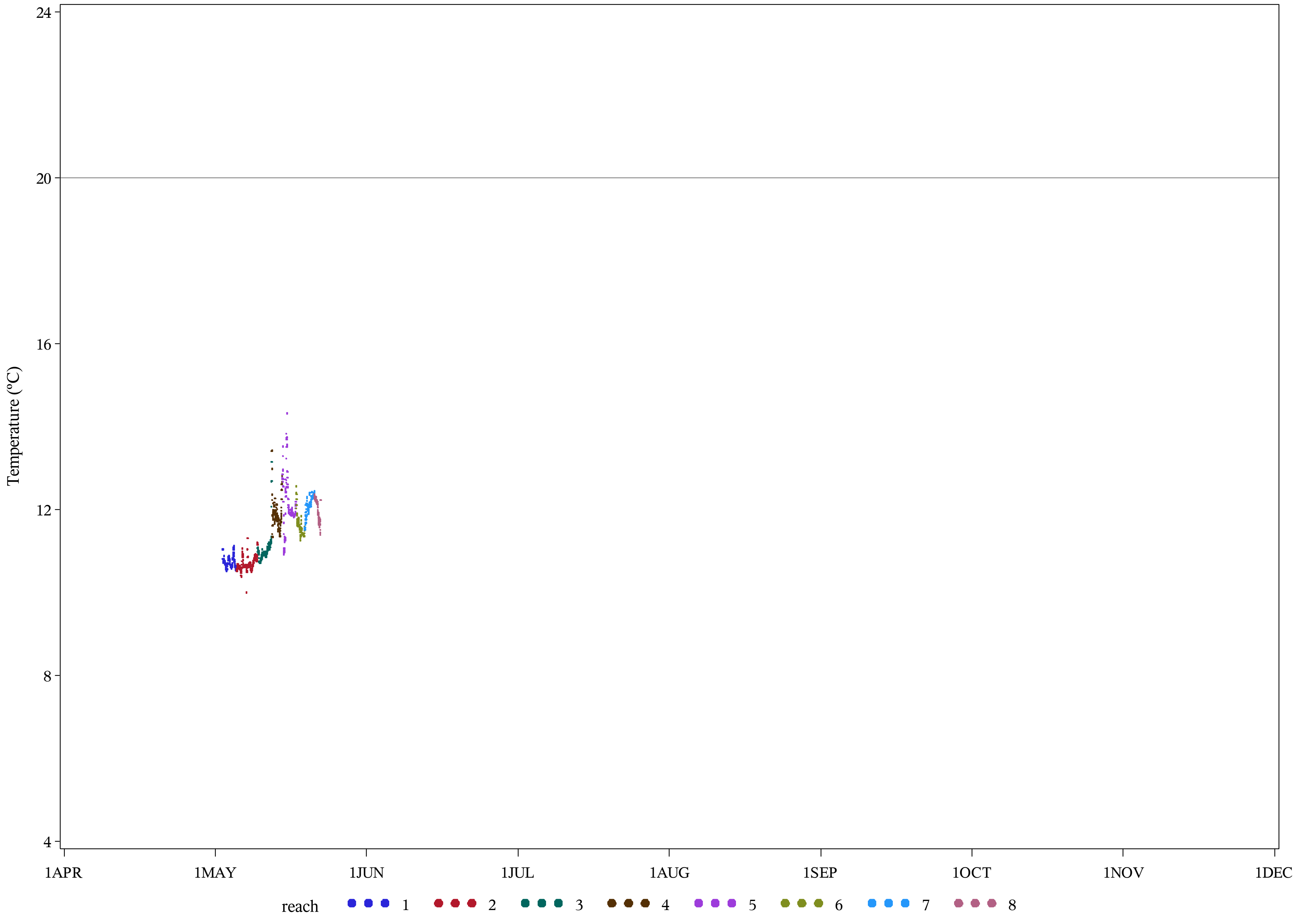

Spring Chinook
2806A

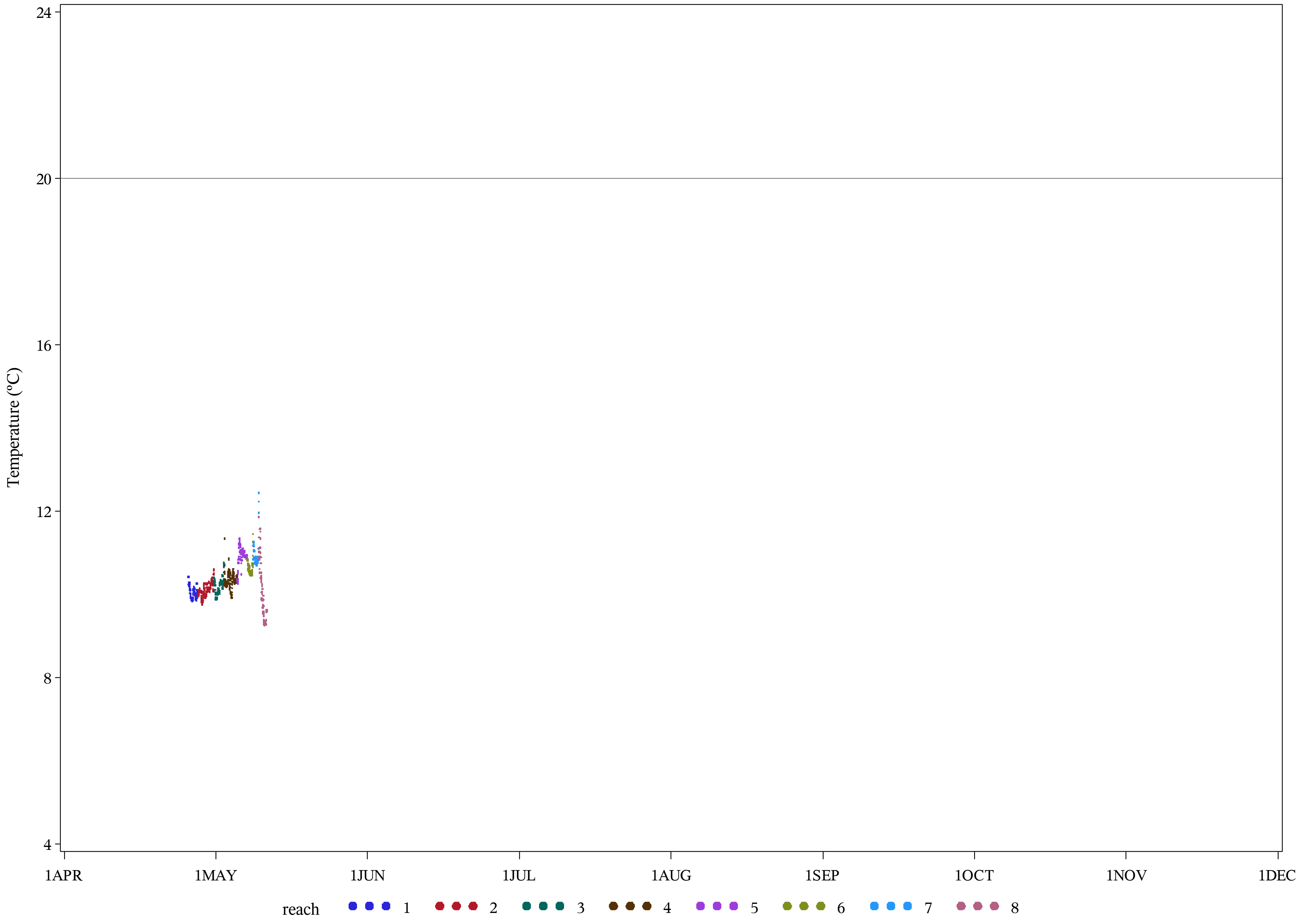

Spring Chinook
2974A

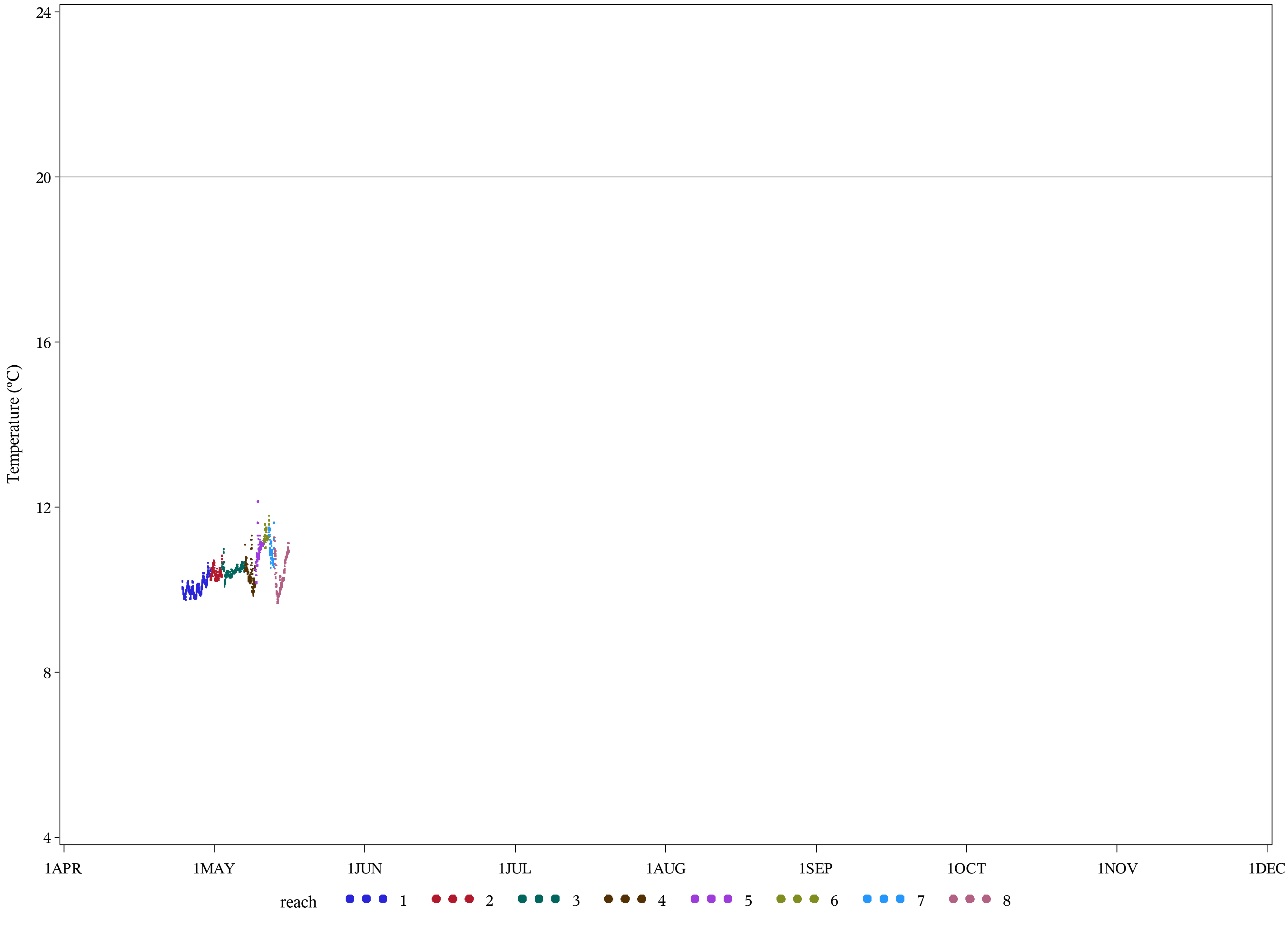

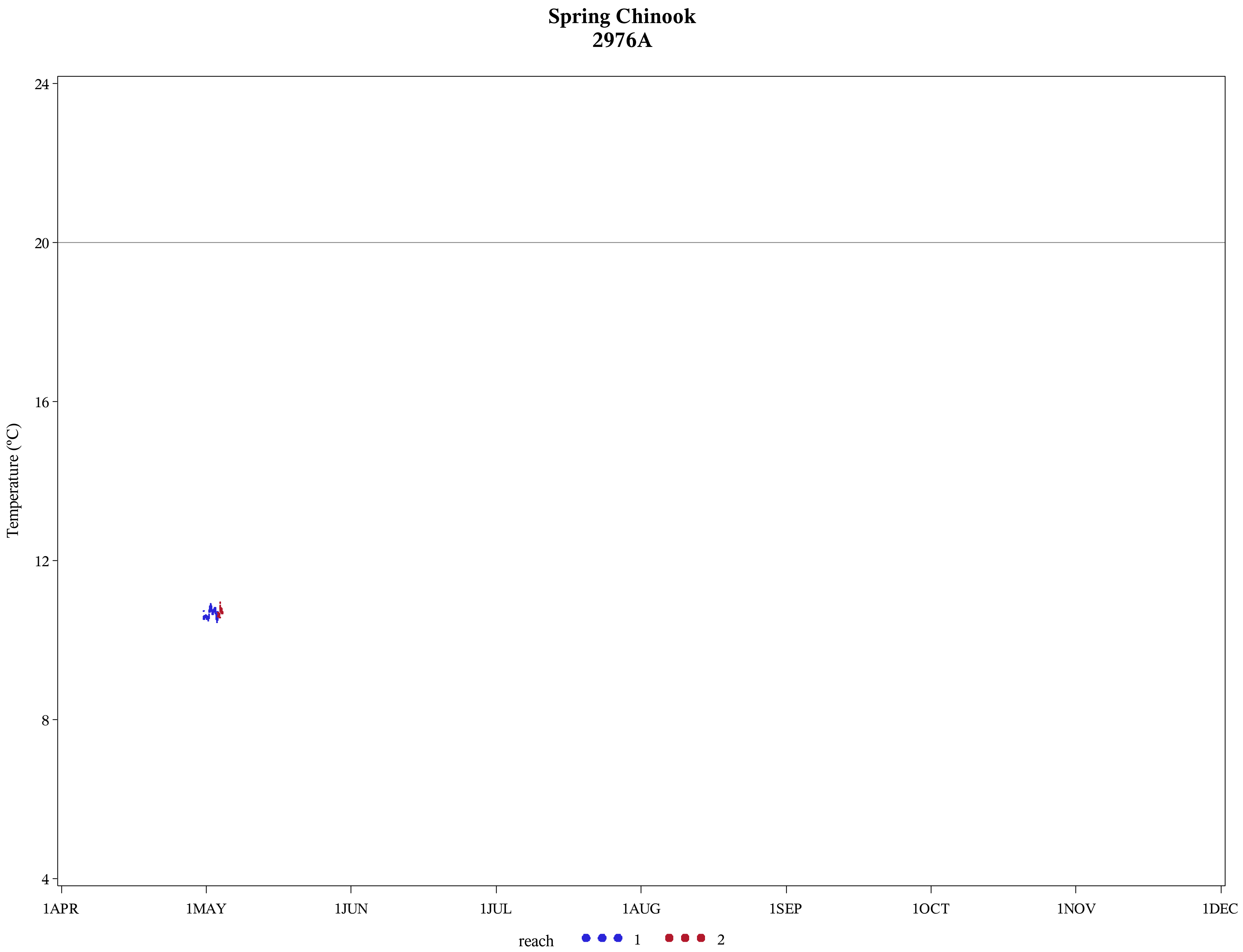
Spring Chinook
2976A
Temperature (°C)
24
20
16
12
8
4
1APR
1MAY
1JUN
1JUL
1AUG
1SEP
1OCT
1NOV
1DEC
reach
1
2

Spring Chinook
2987A

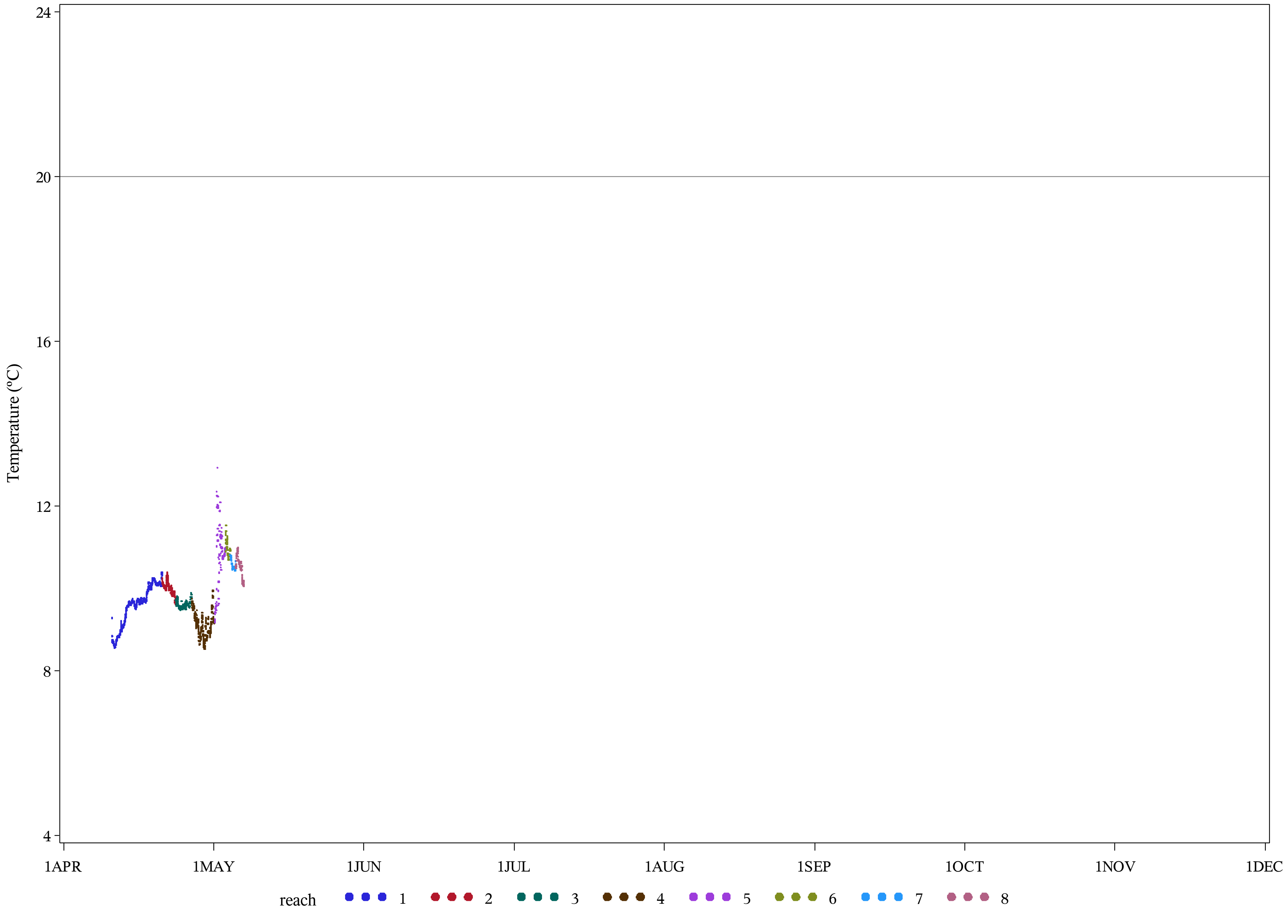

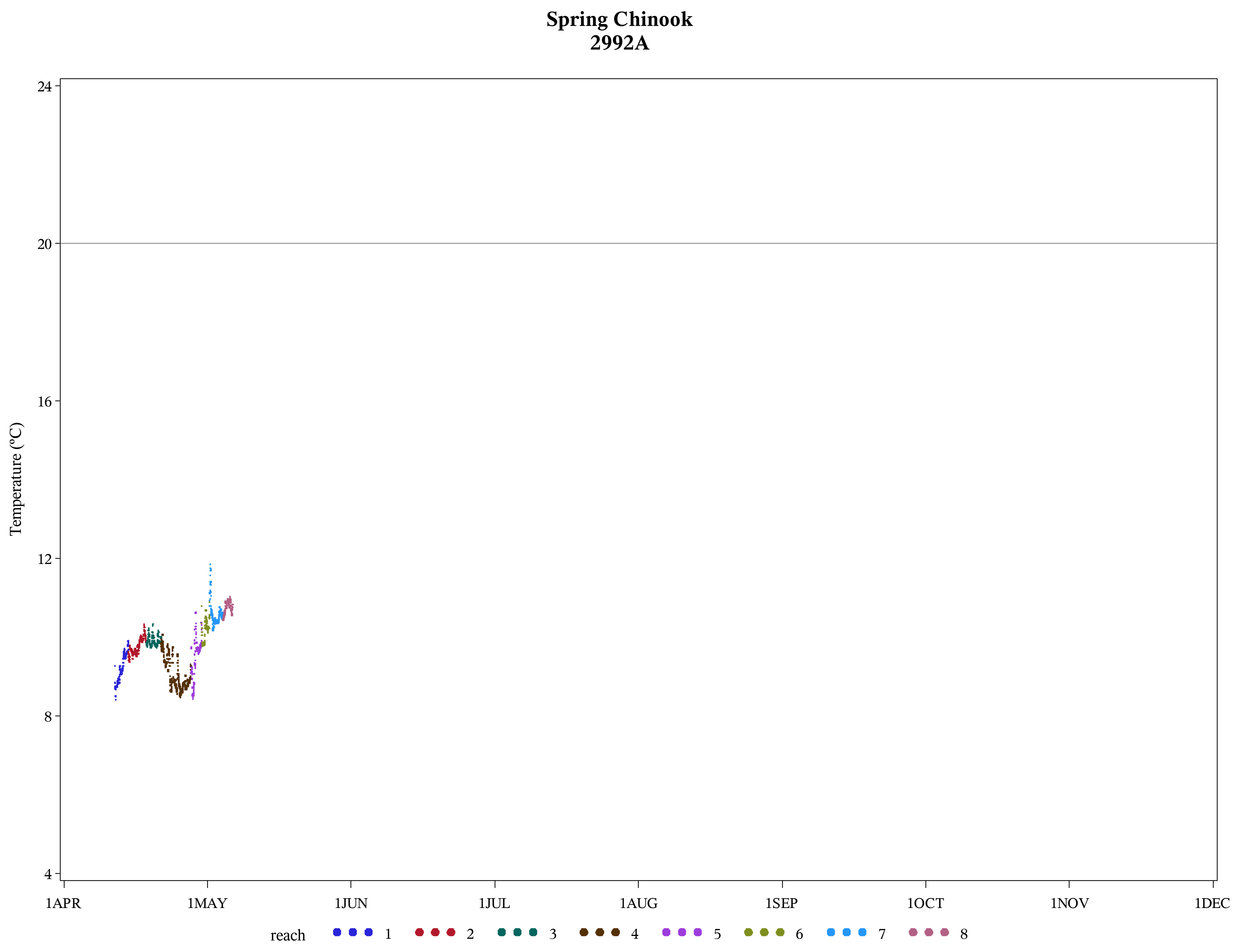

Spring Chinook
2992A
Temperature (°C)
24
20
16
12
8
4
1APR
1MAY
1JUN
1JUL
1AUG
1SEP
1OCT
1NOV
1DEC
reach
1
2
3
4
5
6
7
8

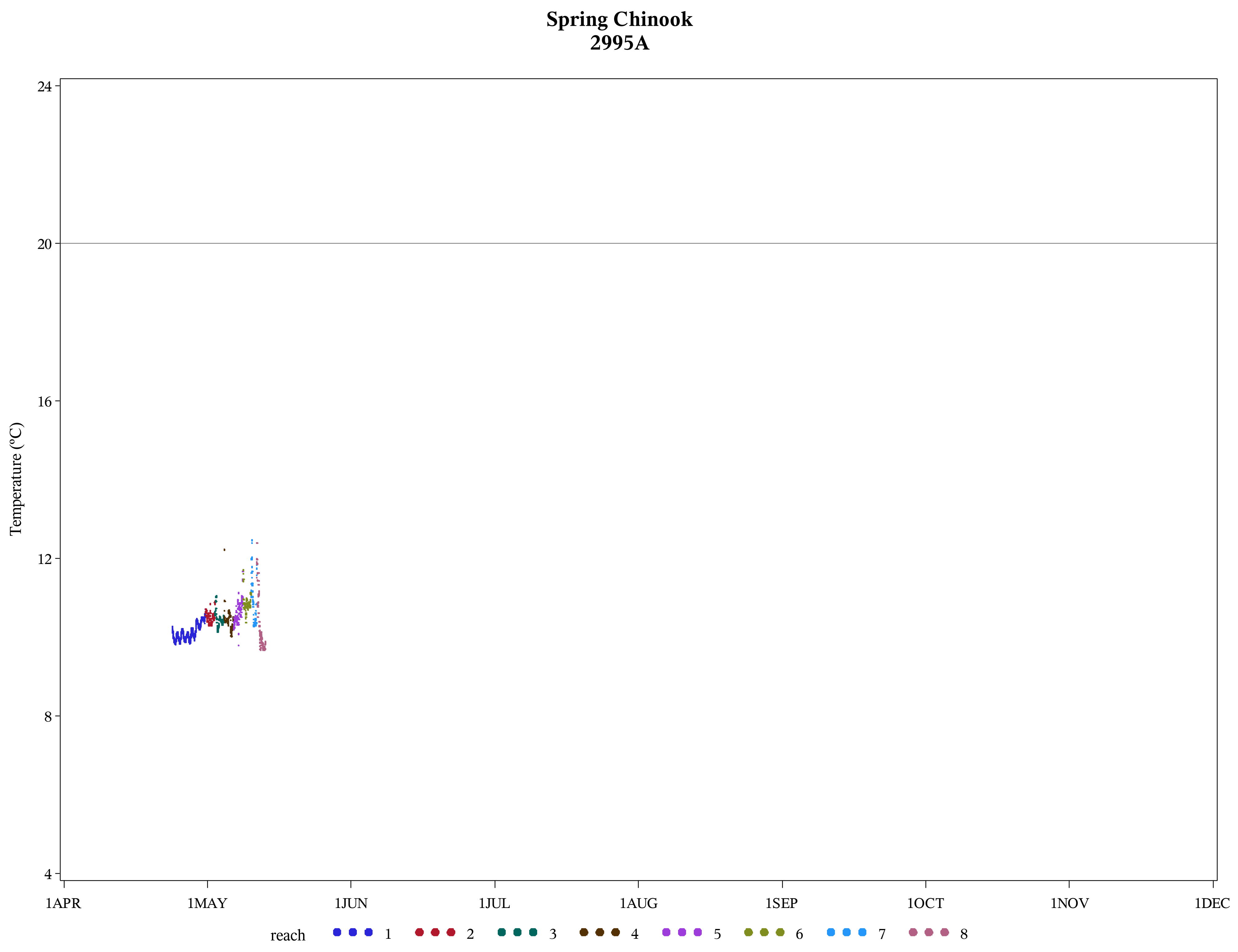

Spring Chinook
2995A
Temperature (°C)
24
20
16
12
8
4
1APR
1MAY
1JUN
1JUL
1AUG
1SEP
1OCT
1NOV
1DEC
reach
1
2
3
4
5
6
7
8

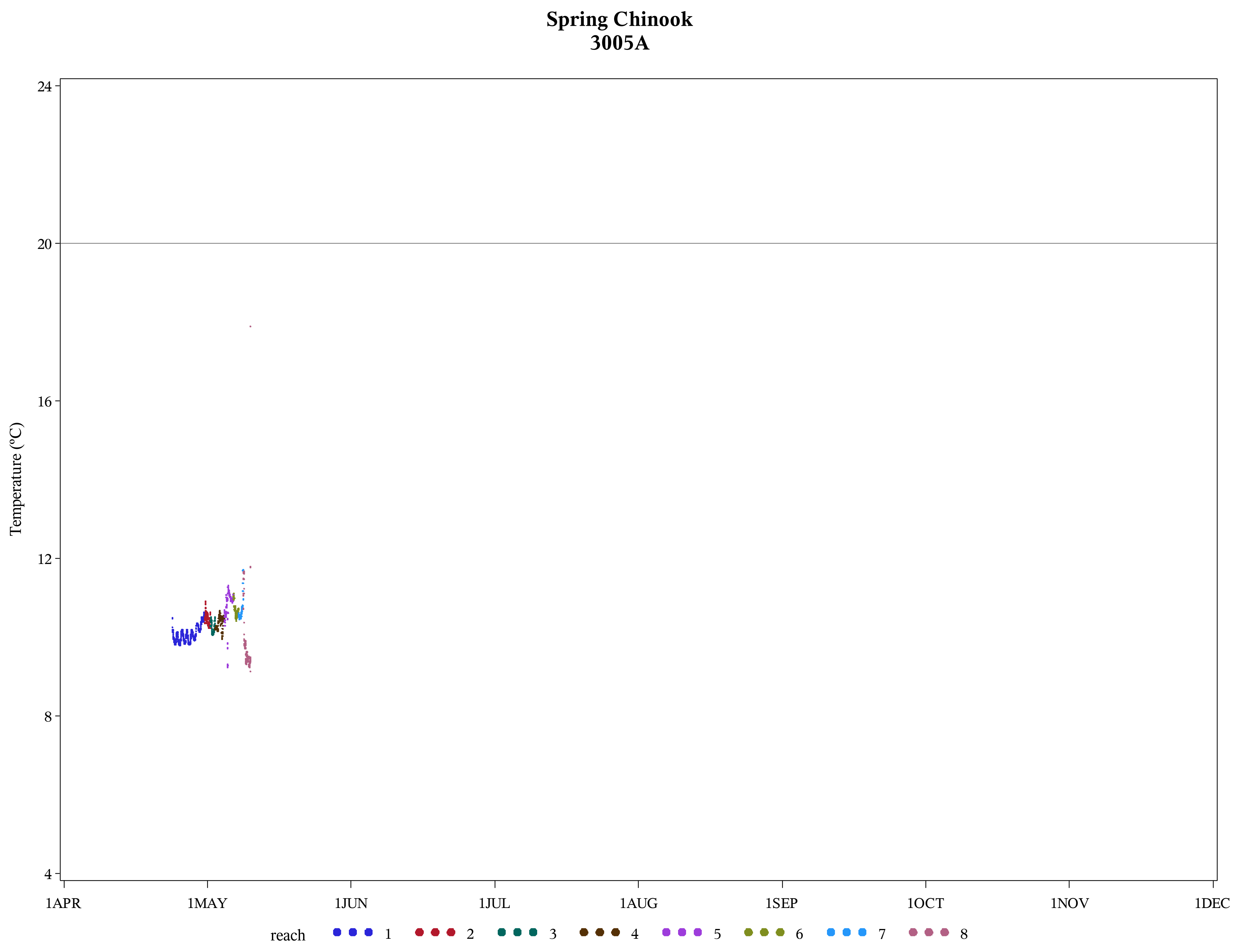
Spring Chinook
3005A
Temperature (°C)
24
20
16
12
8
4
1APR
1MAY
1JUN
1JUL
1AUG
1SEP
1OCT
1NOV
1DEC
reach
1
2
3
4
5
6
7
8

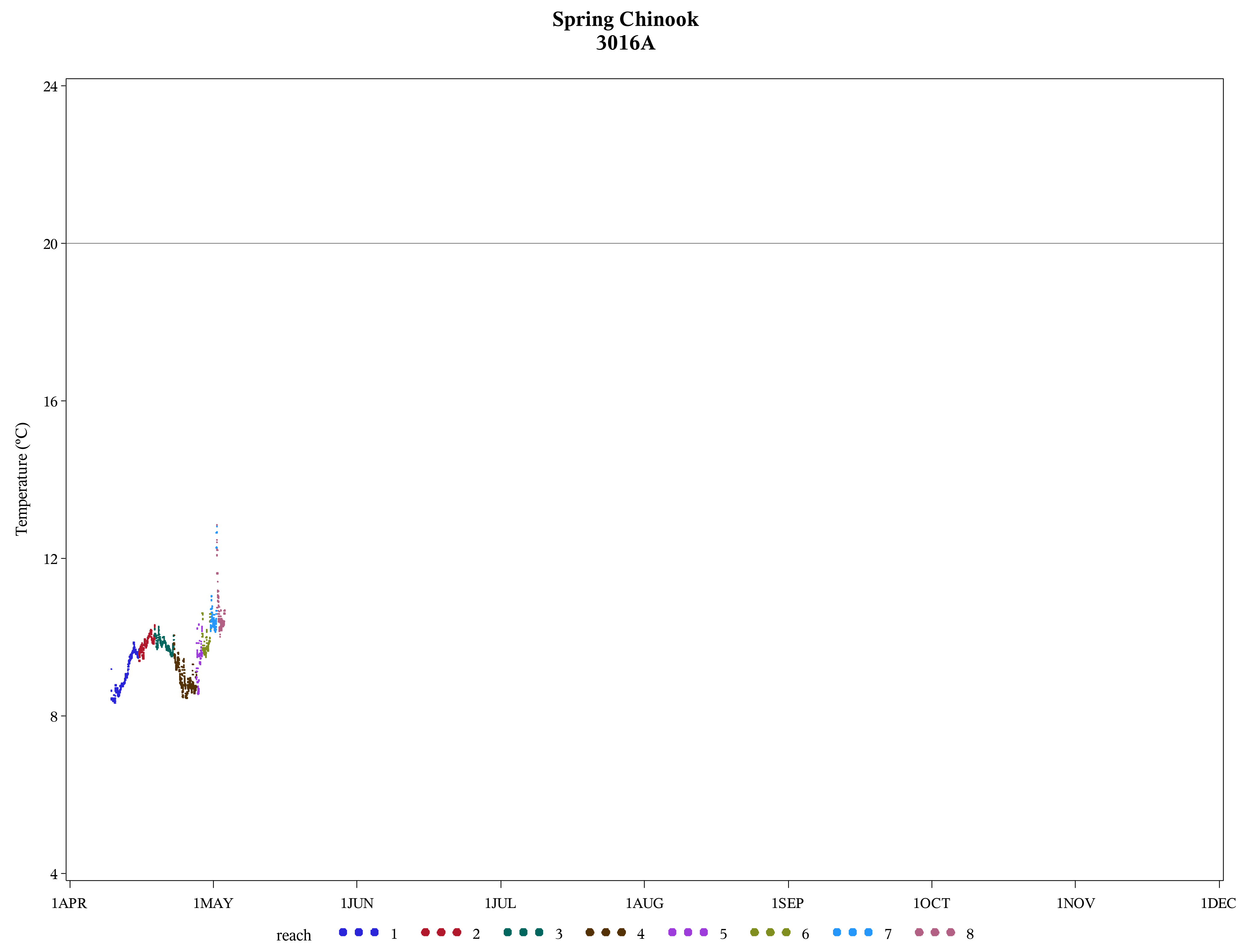

Spring Chinook
3016A
Temperature (°C)
24
20
16
12
8
4
1APR
1MAY
1JUN
1JUL
1AUG
1SEP
1OCT
1NOV
1DEC
reach
1
2
3
4
5
6
7
8

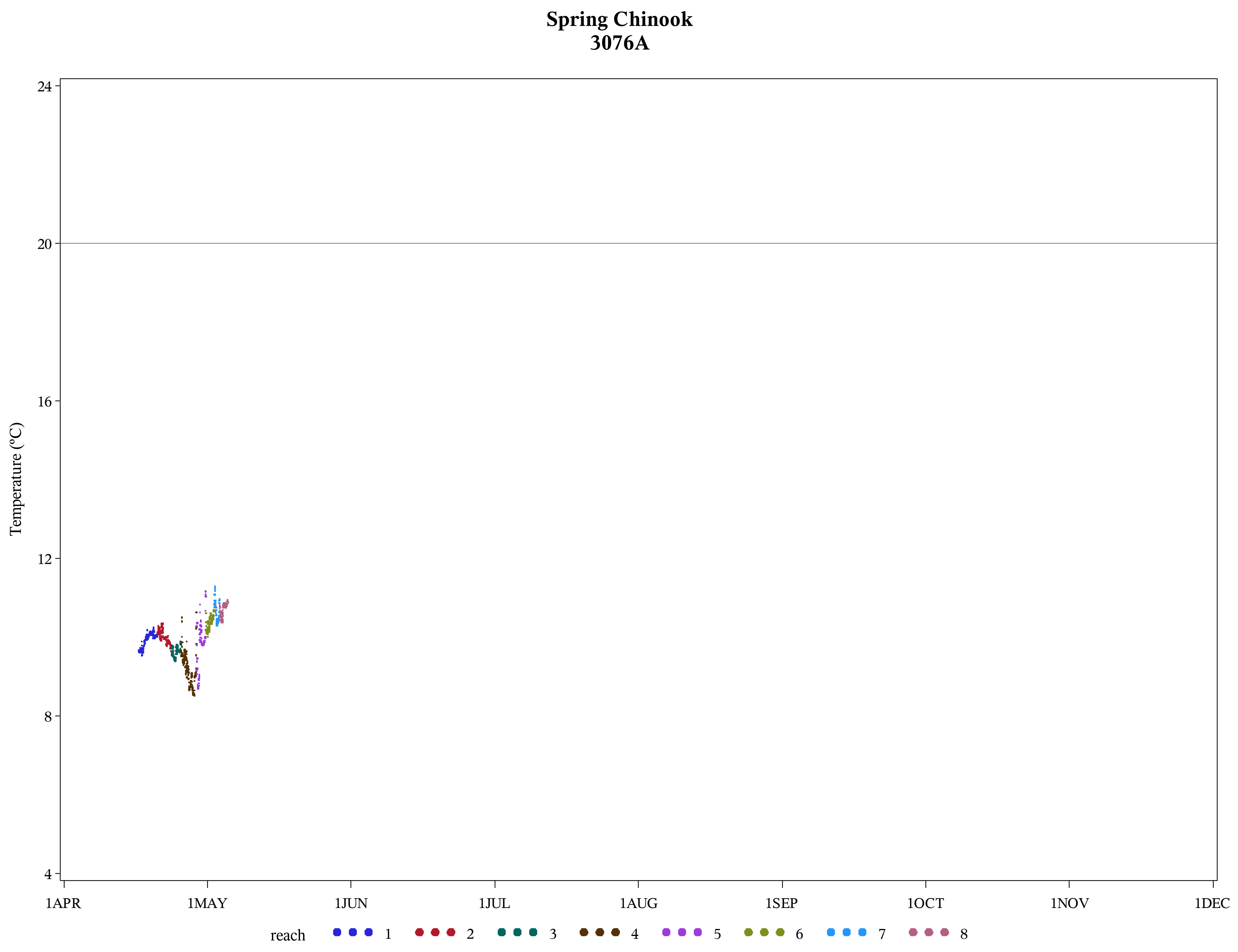

Spring Chinook
3076A
Temperature (°C)
24
20
16
12
8
4
1APR
1MAY
1JUN
1JUL
1AUG
1SEP
1OCT
1NOV
1DEC
reach
1
2
3
4
5
6
7
8

Spring Chinook
3078A

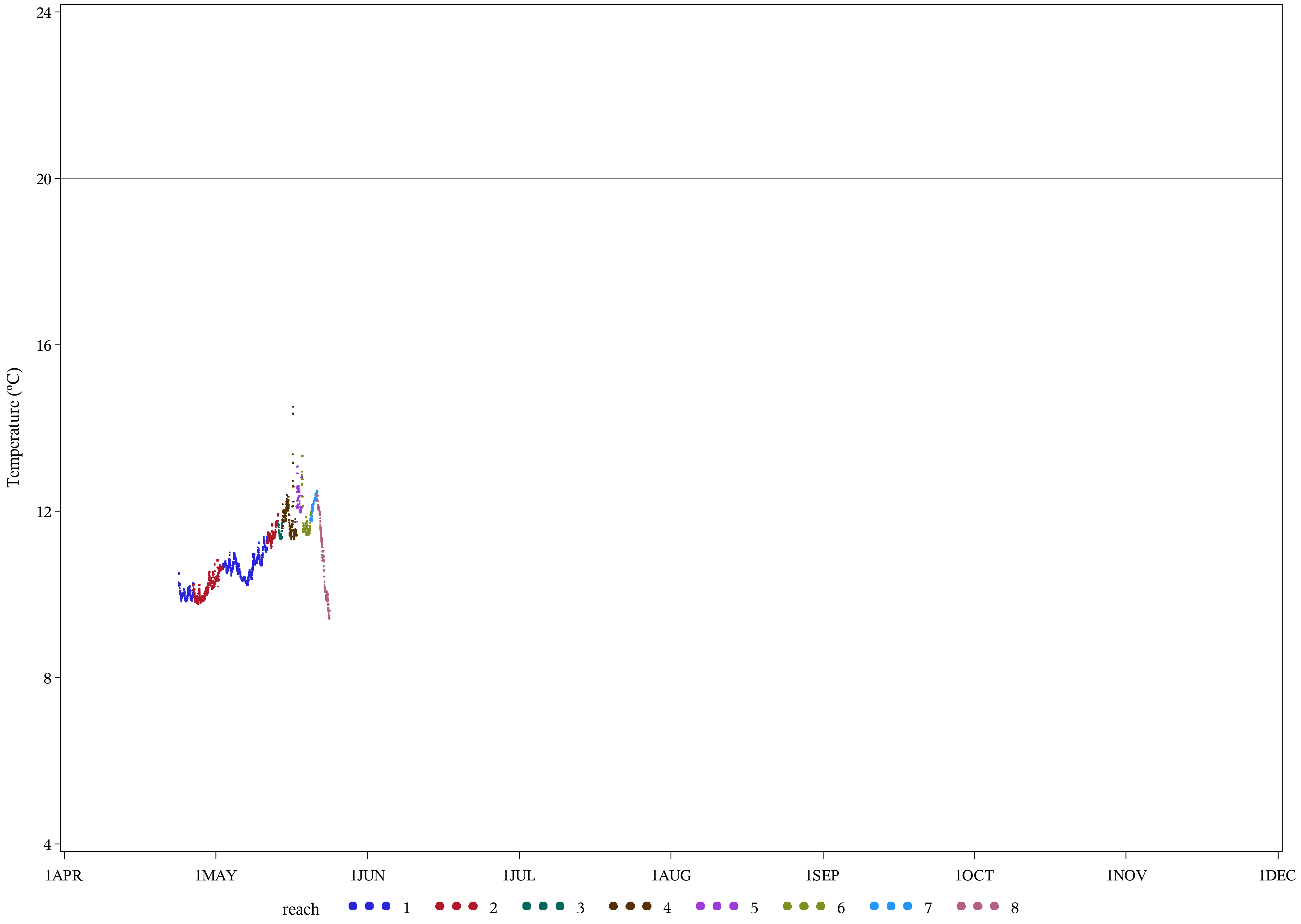

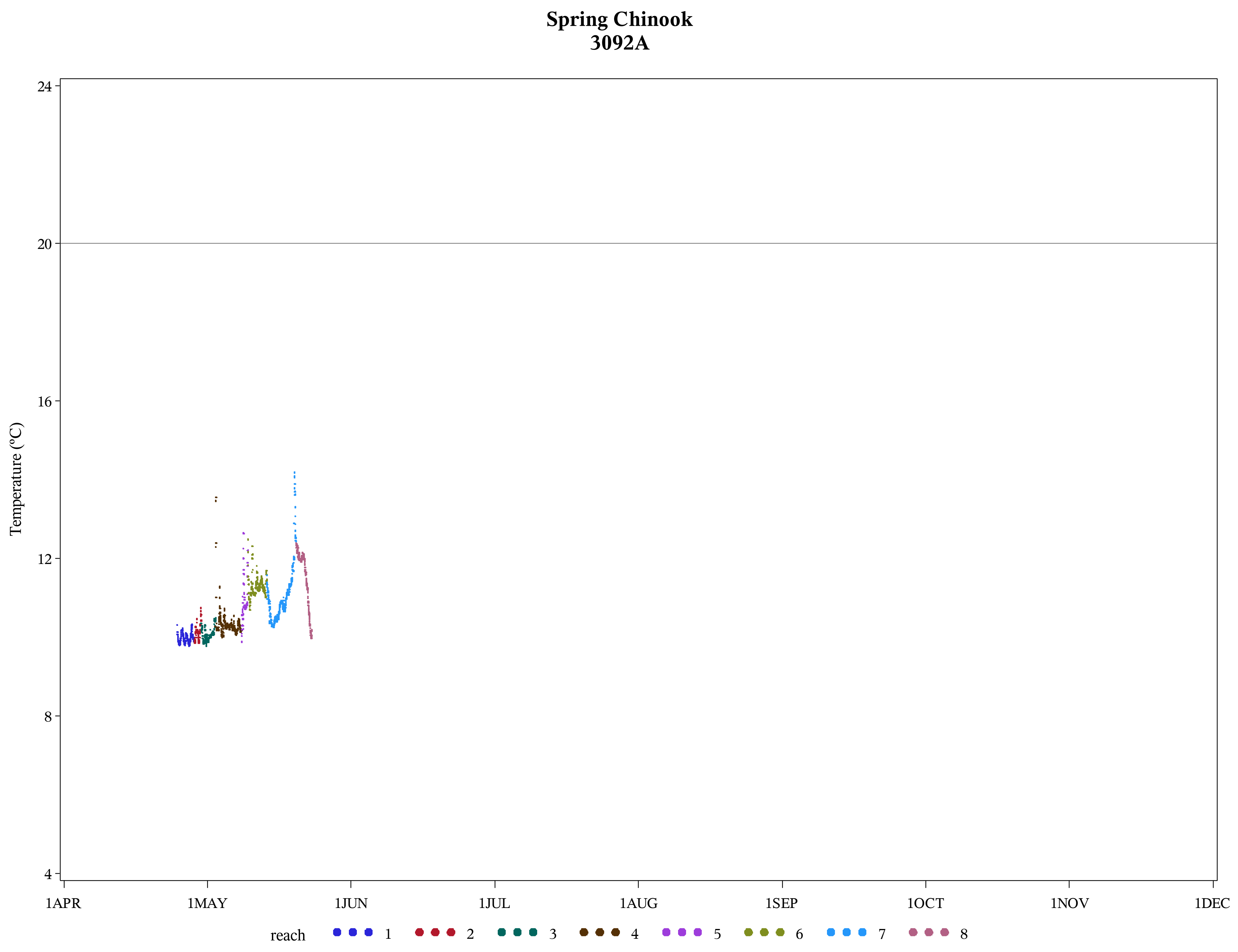
Spring Chinook
3092A
Temperature (°C)
24
20
16
12
8
4
1APR
1MAY
1JUN
1JUL
1AUG
1SEP
1OCT
1NOV
1DEC
reach
1
2
3
4
5
6
7
8

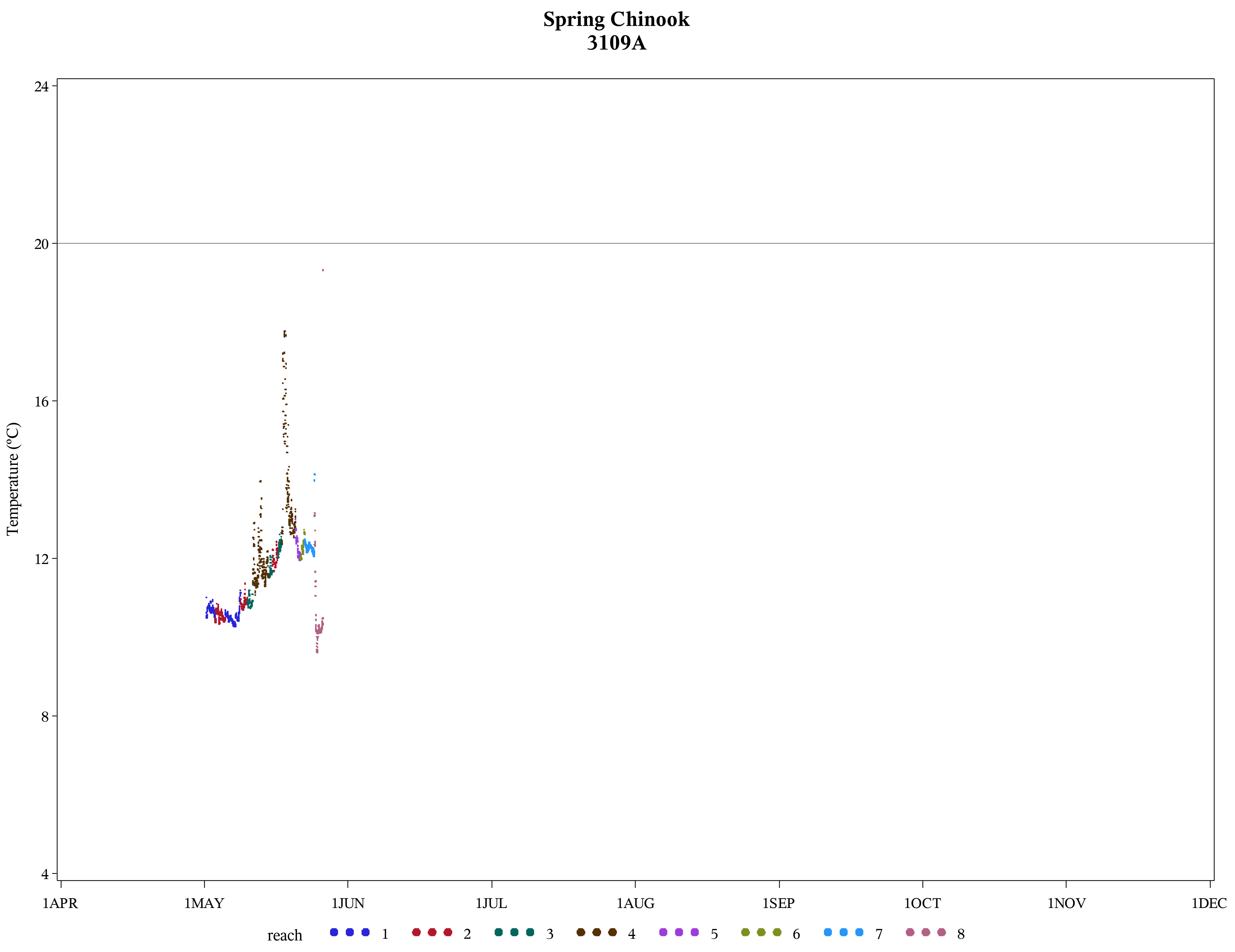

Spring Chinook
3109A
Temperature (°C)
24
20
16
12
8
4
1APR
1MAY
1JUN
1JUL
1AUG
1SEP
1OCT
1NOV
1DEC
reach
1
2
3
4
5
6
7
8

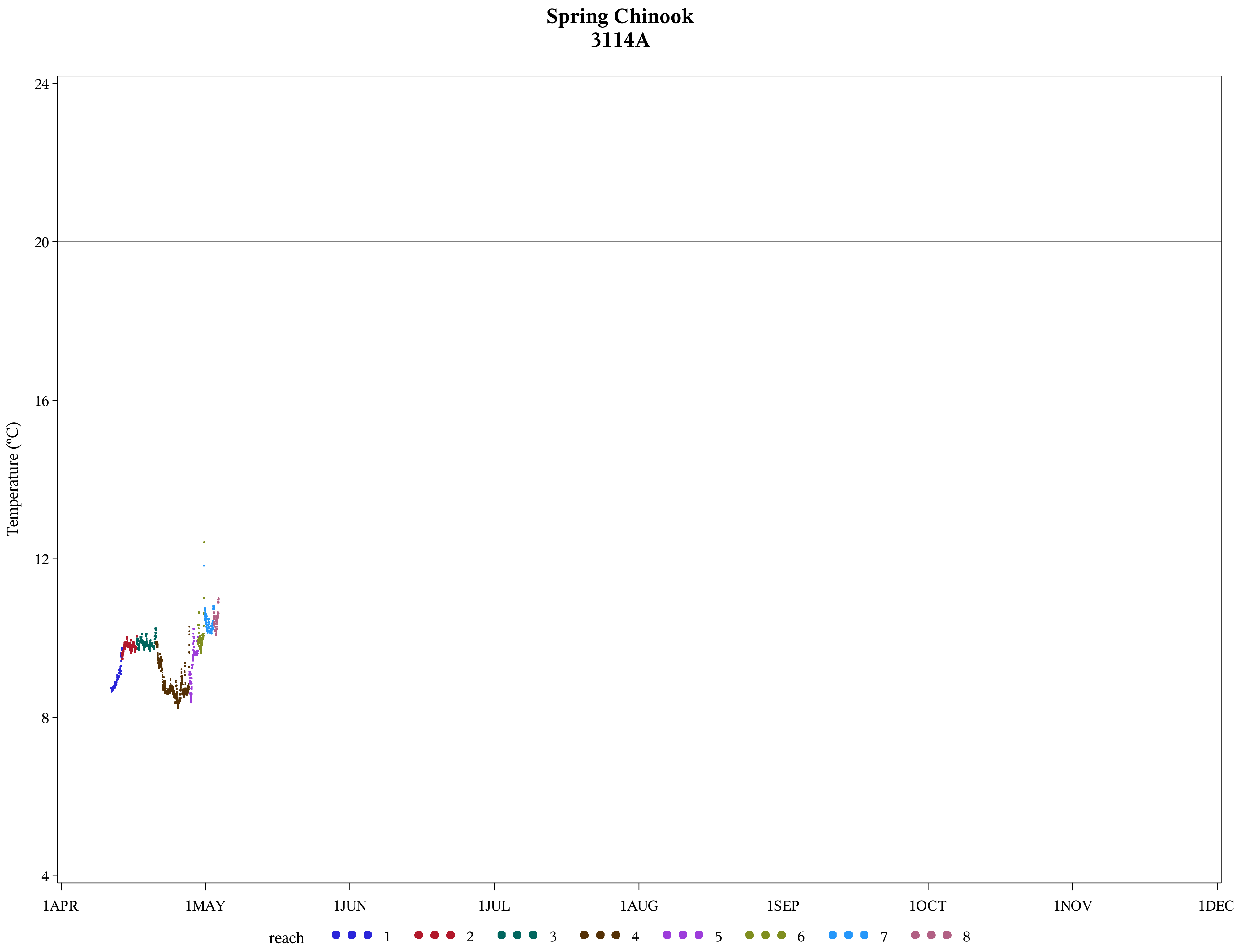

Spring Chinook
3114A
Temperature (°C)
24
20
16
12
8
4
1APR
1MAY
1JUN
1JUL
1AUG
1SEP
1OCT
1NOV
1DEC
reach
1
2
3
4
5
6
7
8

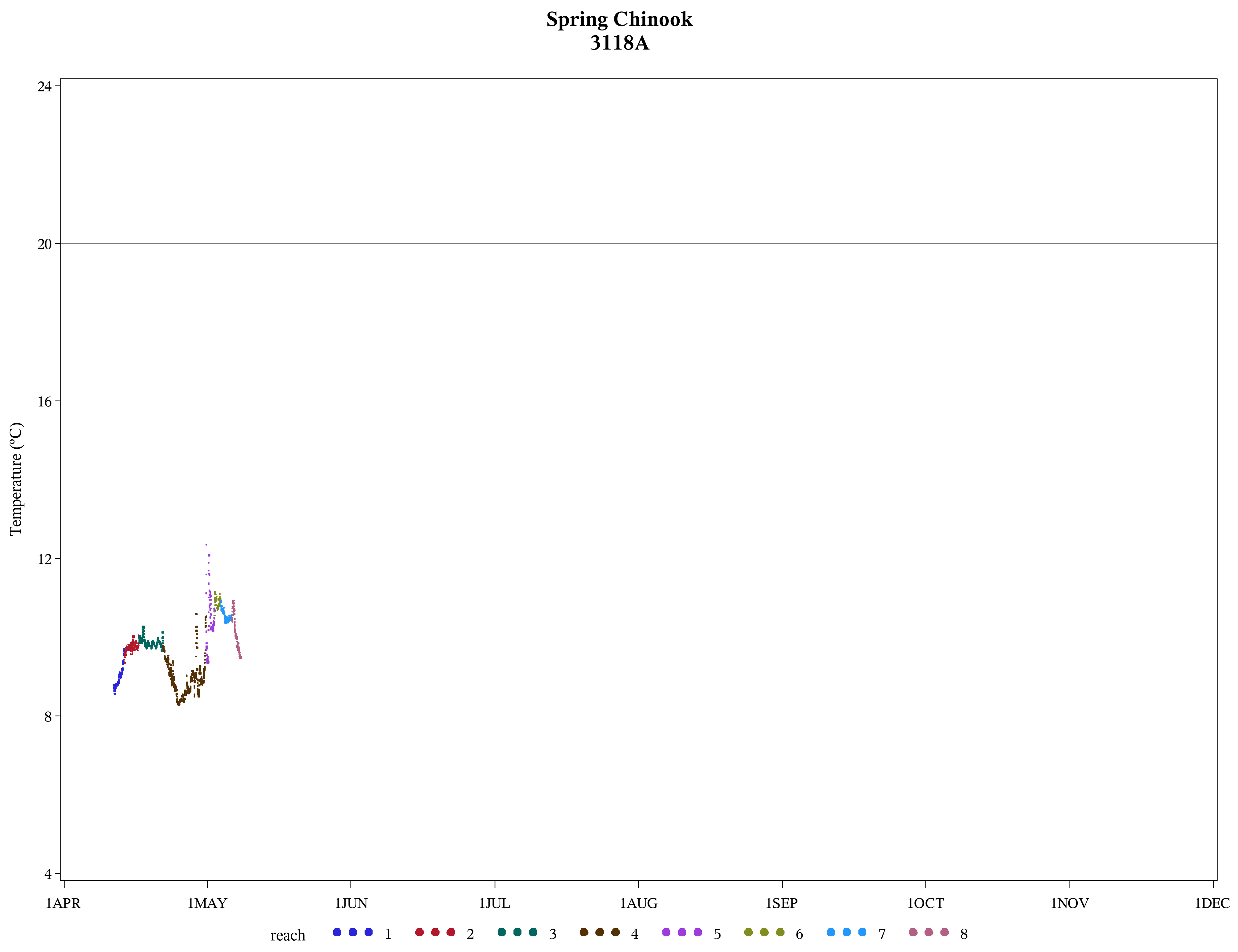
Spring Chinook
3118A
Temperature (ºC)
24
20
16
12
8
4
1APR
1MAY
1JUN
1JUL
1AUG
1SEP
1OCT
1NOV
1DEC
reach
1
2
3
4
5
6
7
8

Spring Chinook
3271A

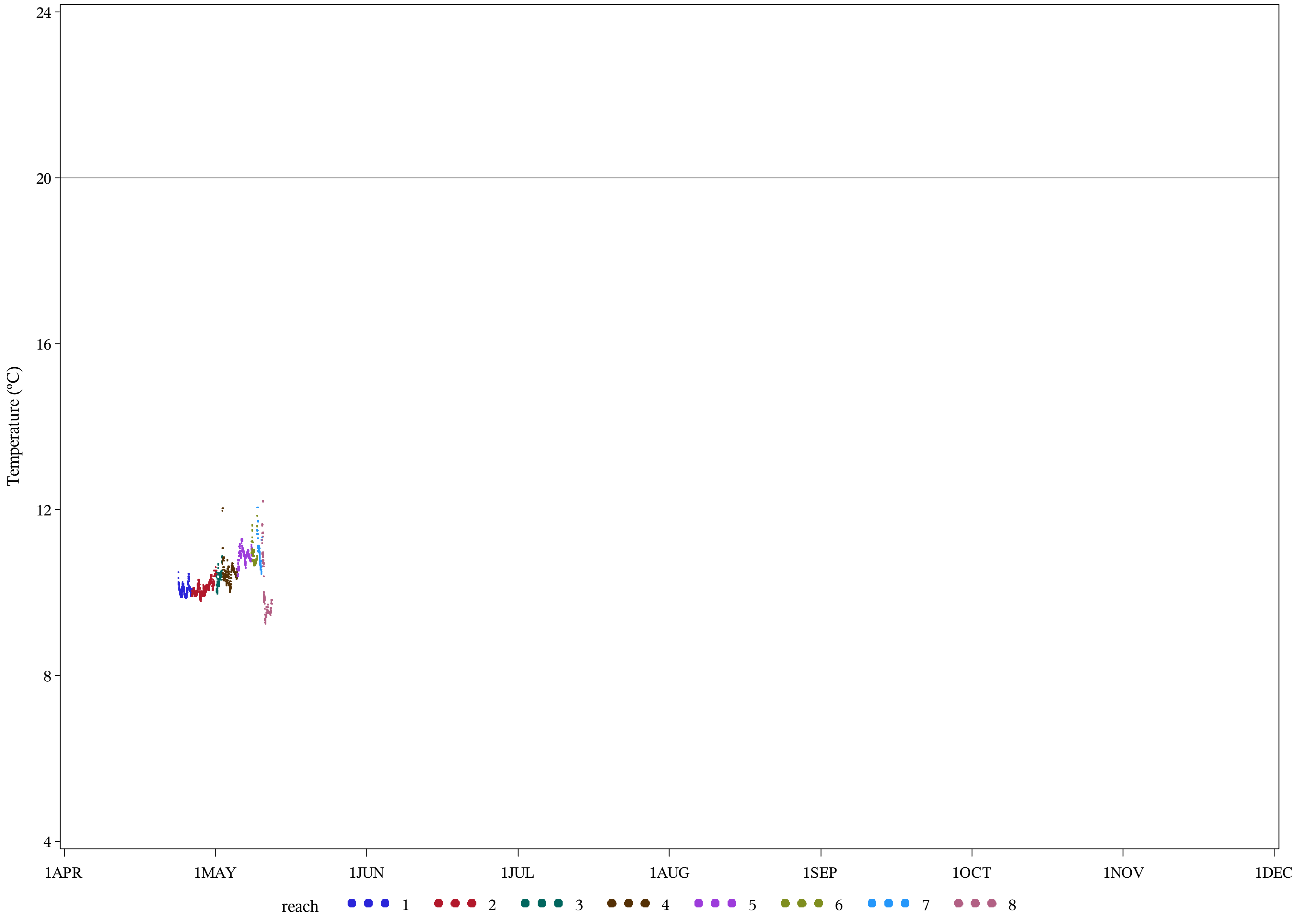

Spring Chinook
3284A

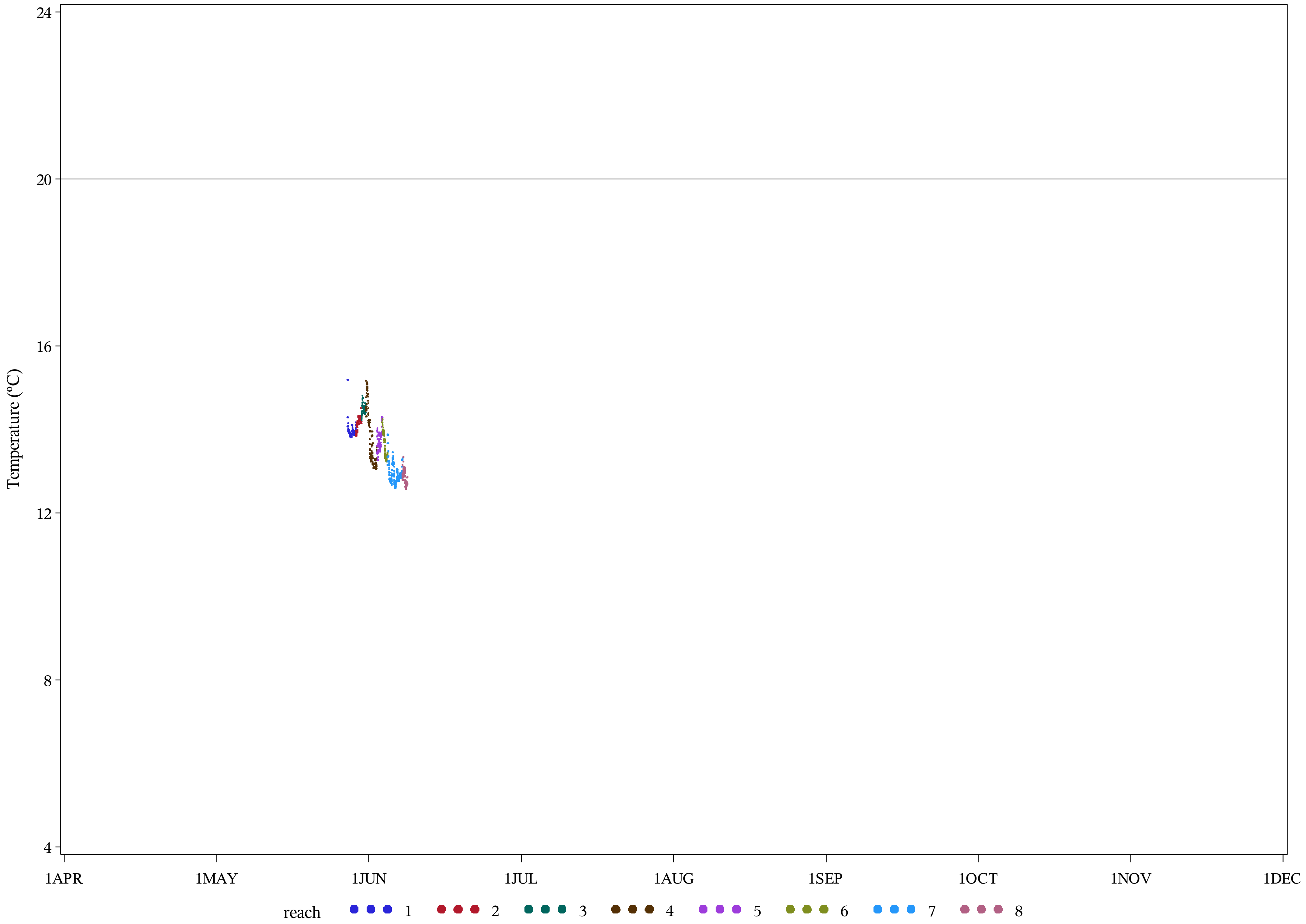

Spring Chinook
3535A

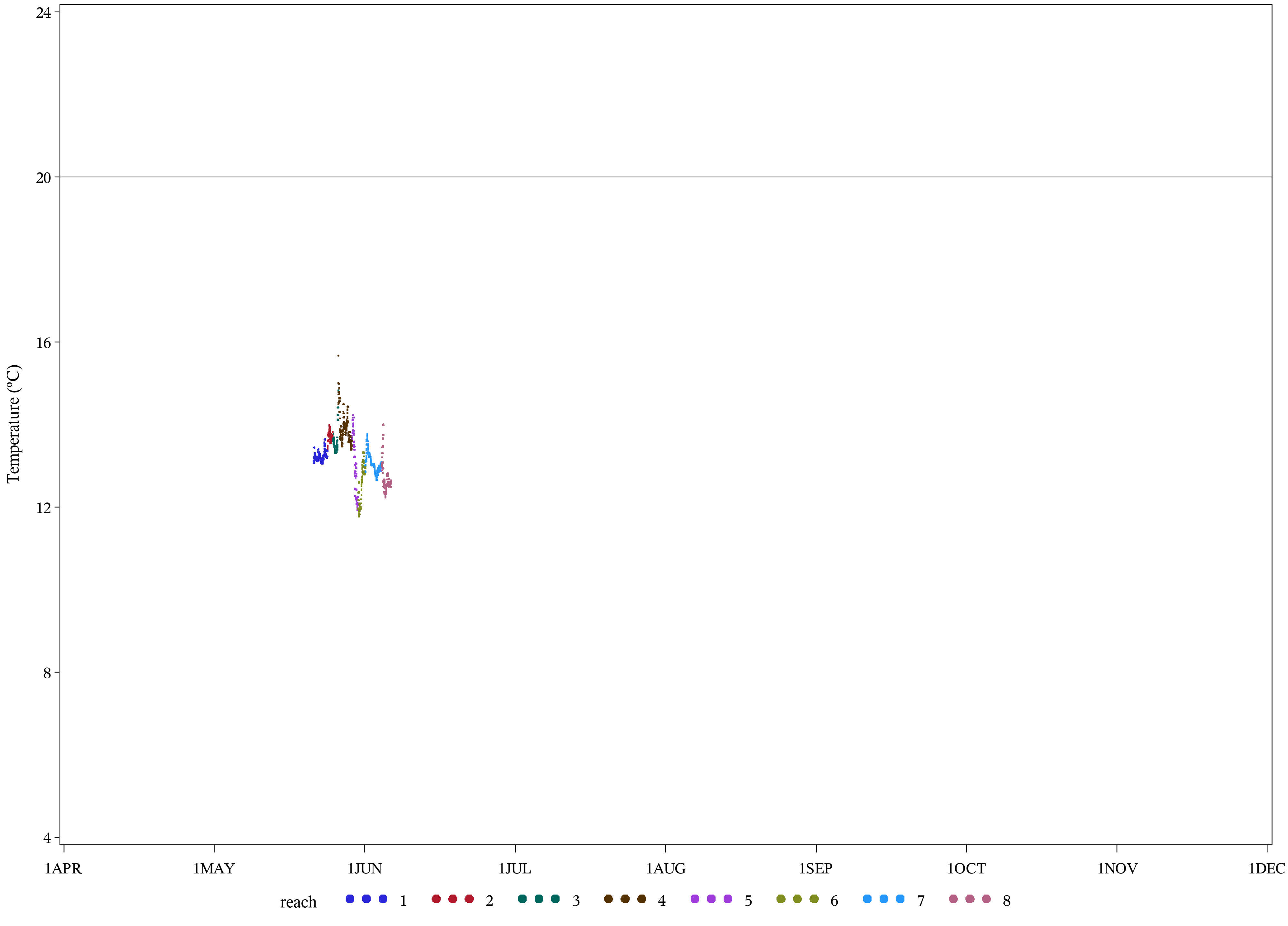

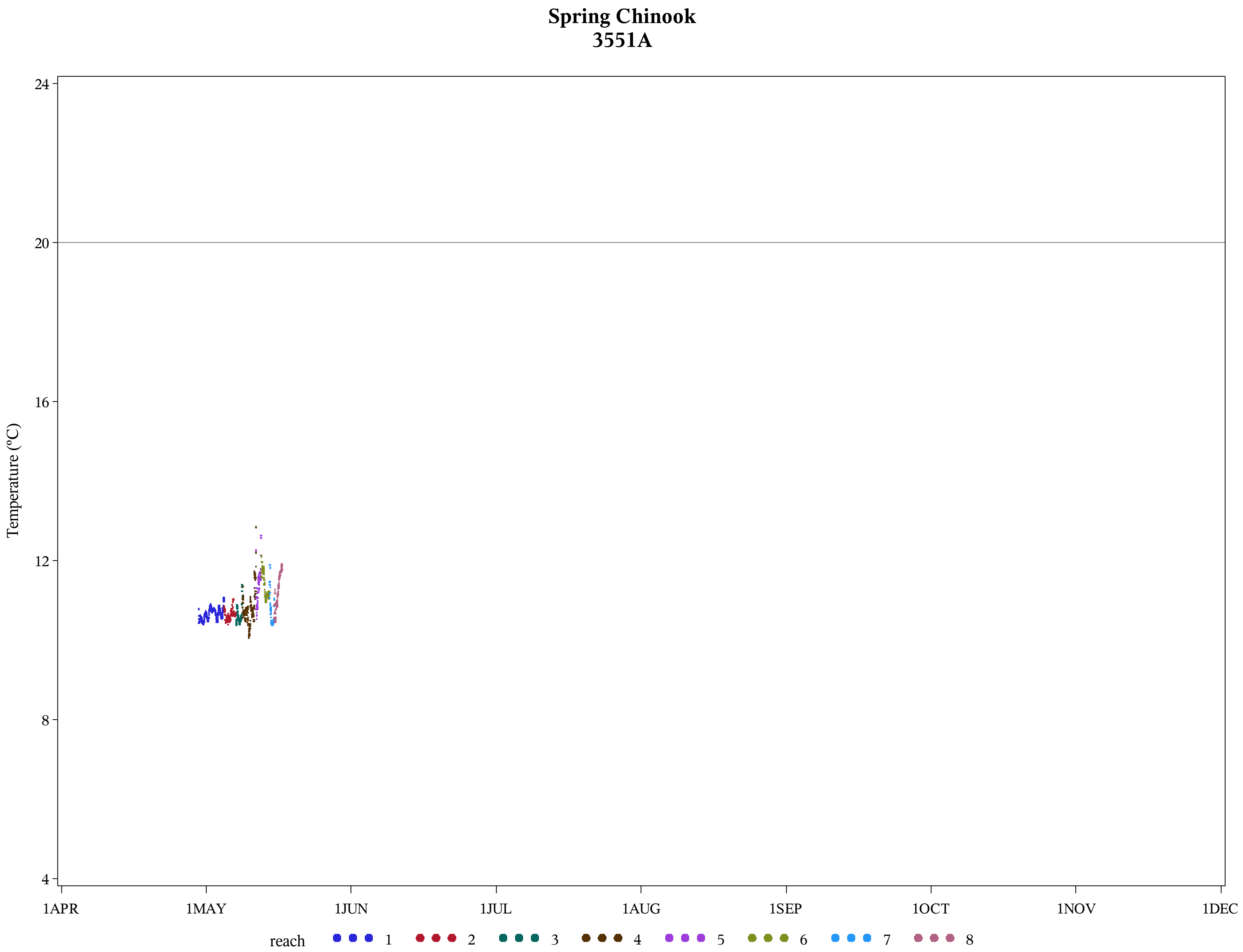

Spring Chinook
3551A
Temperature (°C)
24
20
16
12
8
4
1APR
1MAY
1JUN
1JUL
1AUG
1SEP
1OCT
1NOV
1DEC
reach
1
2
3
4
5
6
7
8

Spring Chinook
3563A

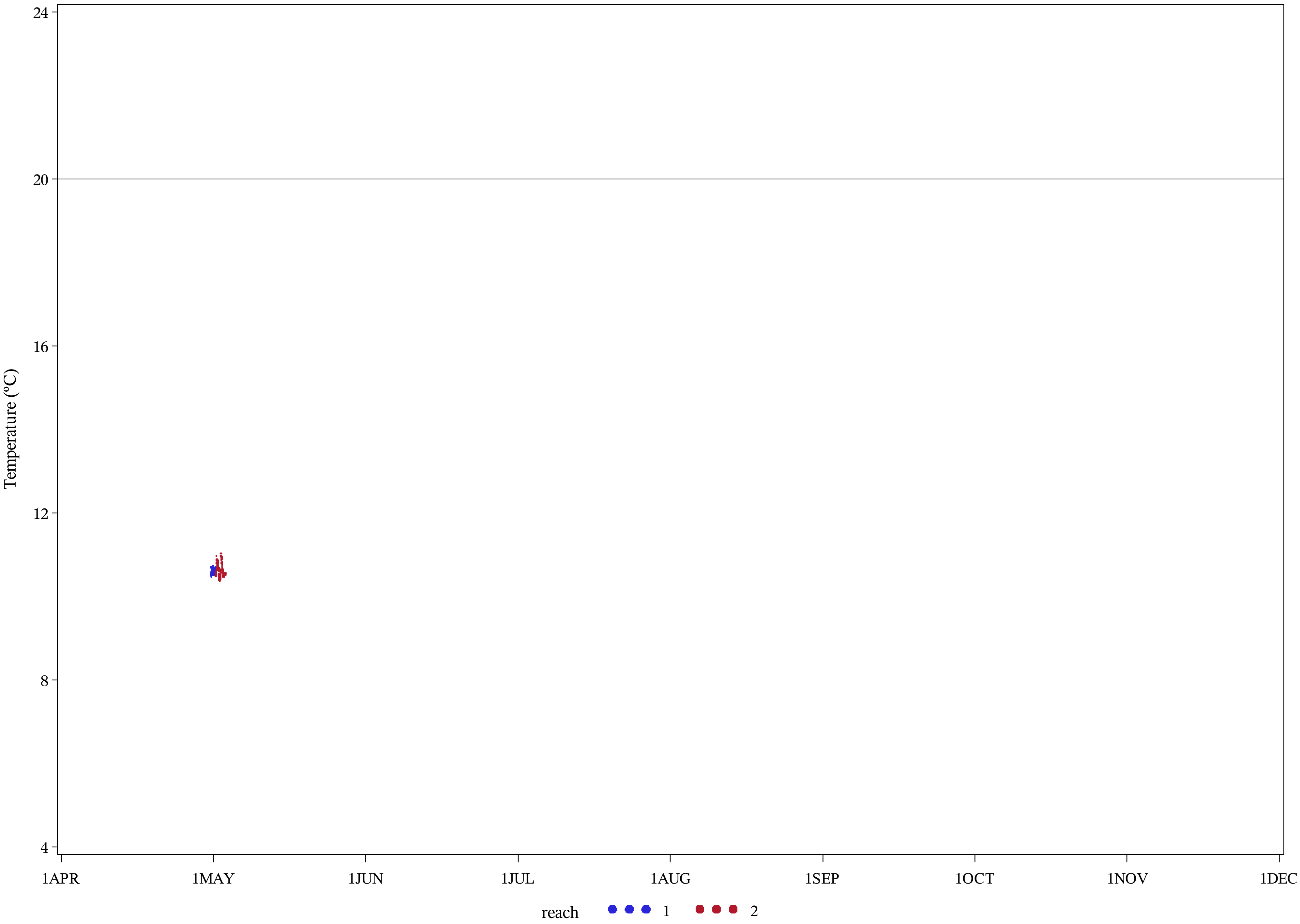

Spring Chinook
3577A

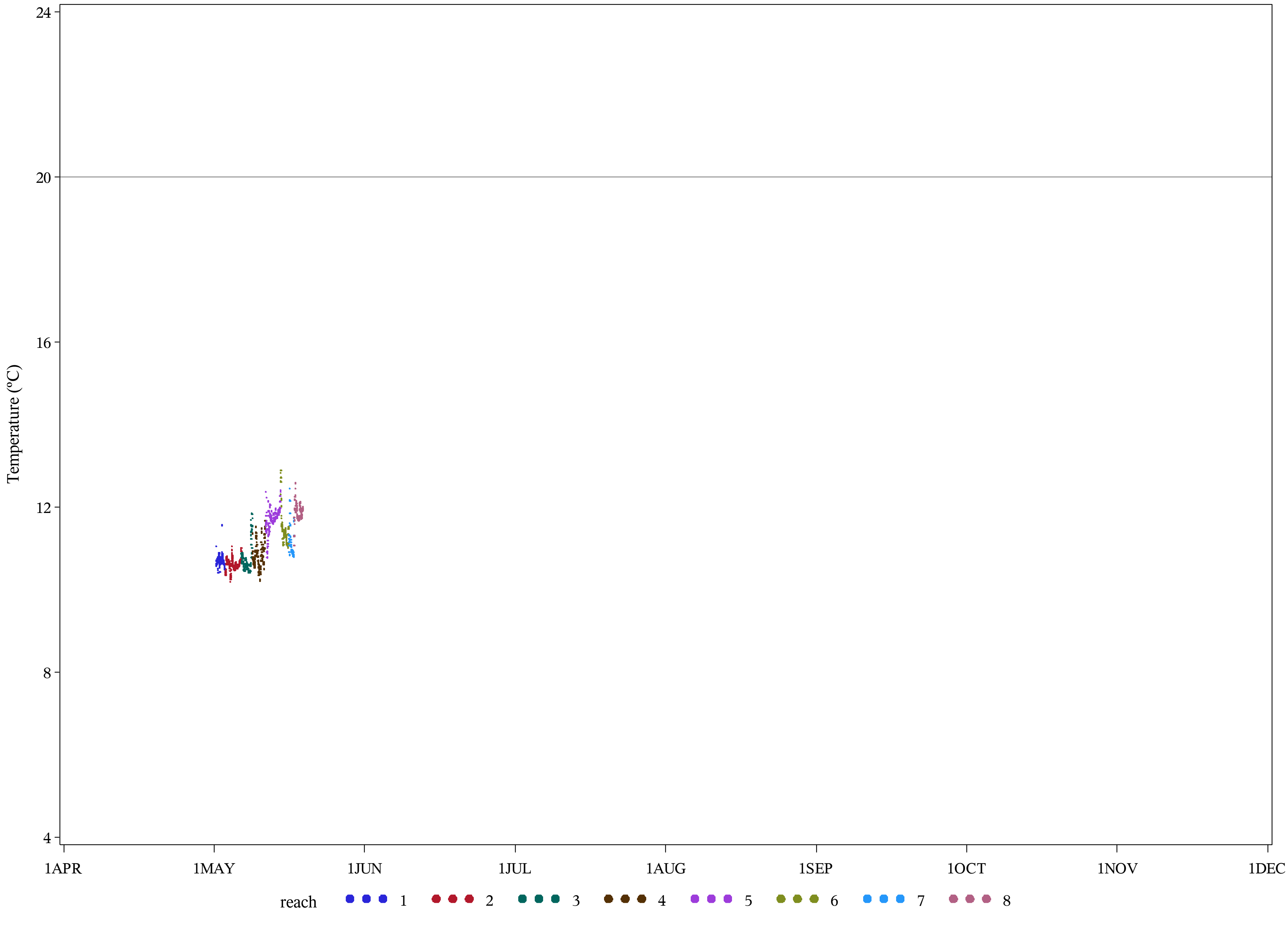

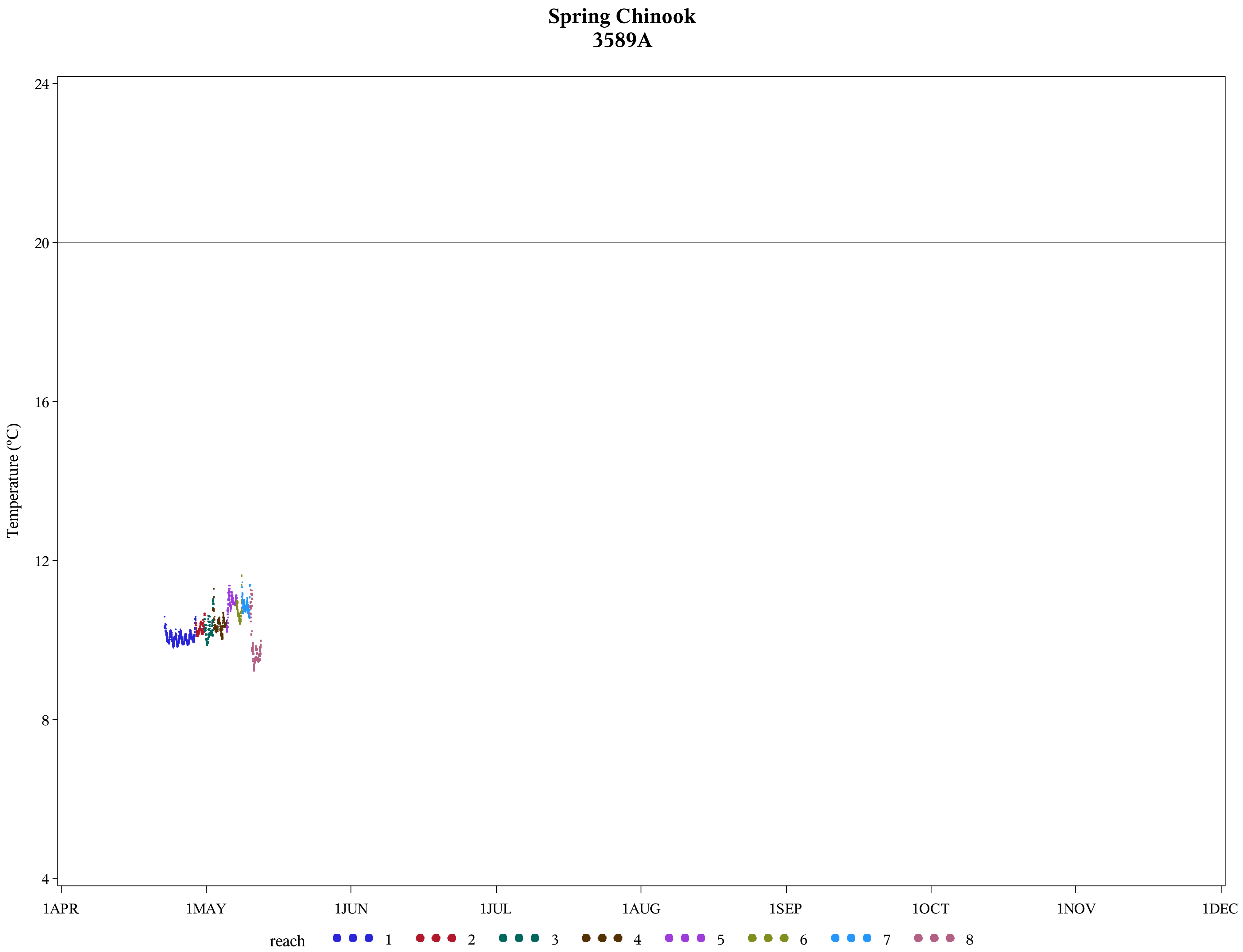

Spring Chinook
3589A
Temperature (°C)
24
20
16
12
8
4
1APR
1MAY
1JUN
1JUL
1AUG
1SEP
1OCT
1NOV
1DEC
reach
1
2
3
4
5
6
7
8

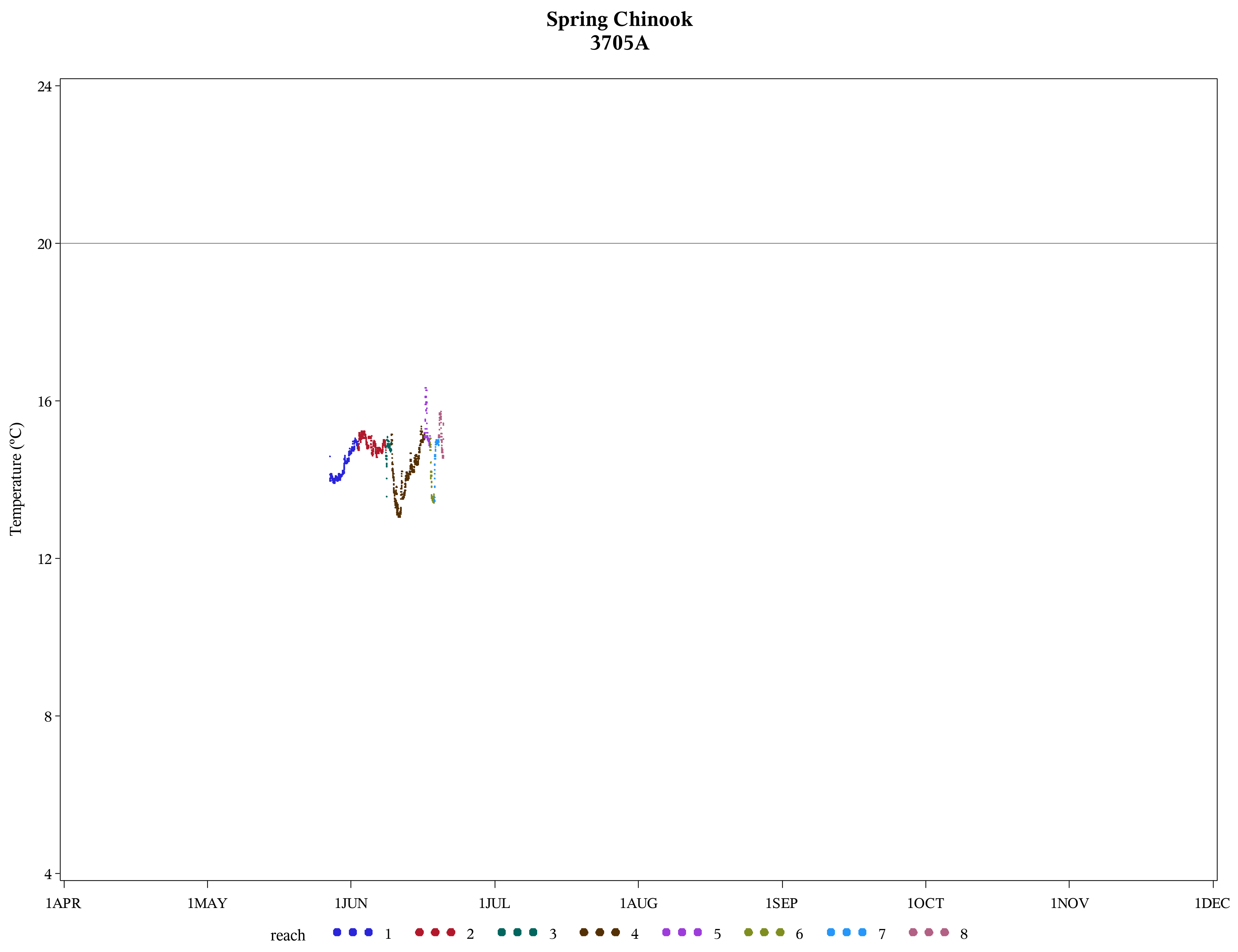
Spring Chinook
3705A
Temperature (°C)
24
20
16
12
8
4
1APR
1MAY
1JUN
1JUL
1AUG
1SEP
1OCT
1NOV
1DEC
reach
1
2
3
4
5
6
7
8

Spring Chinook
3710A

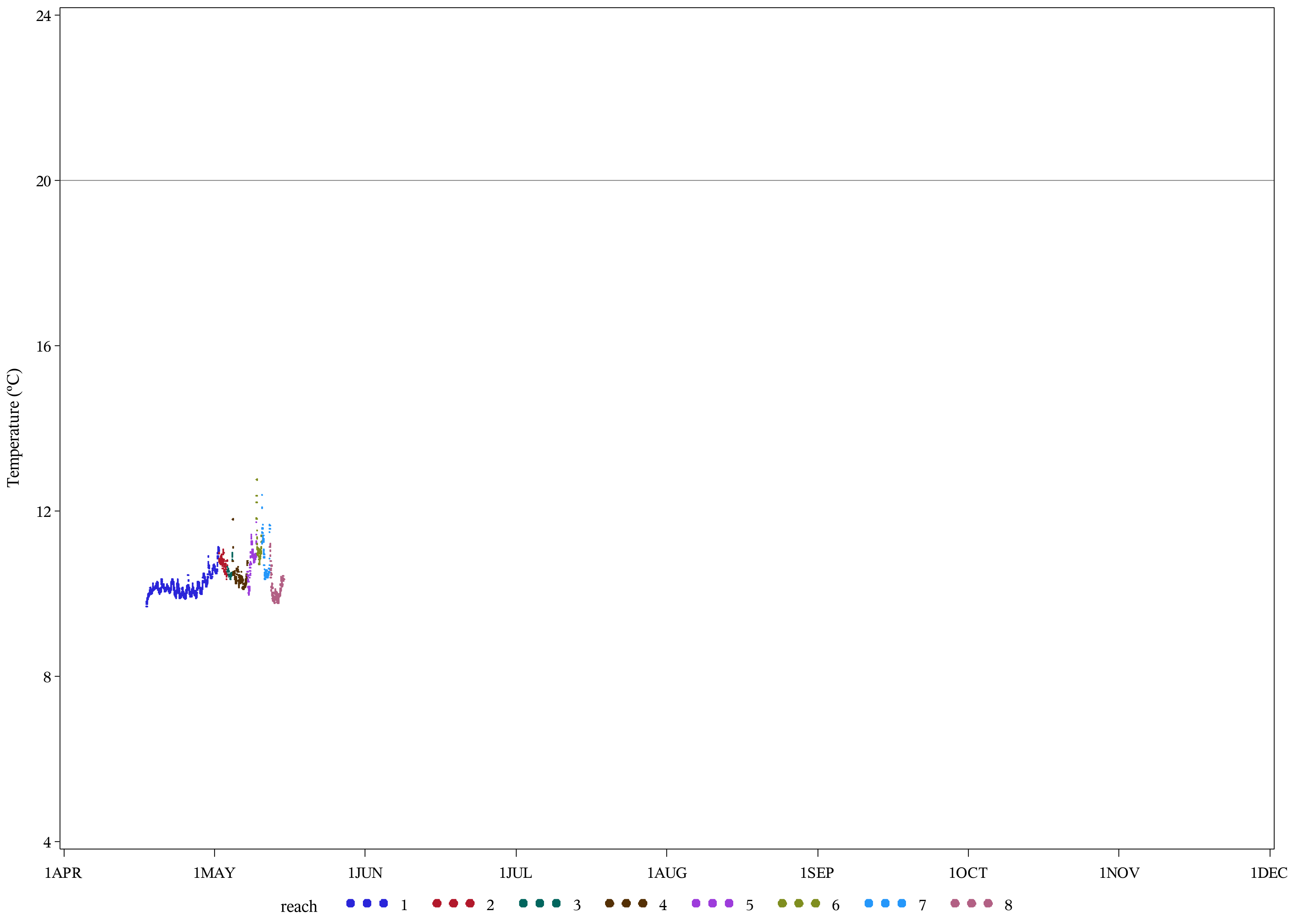

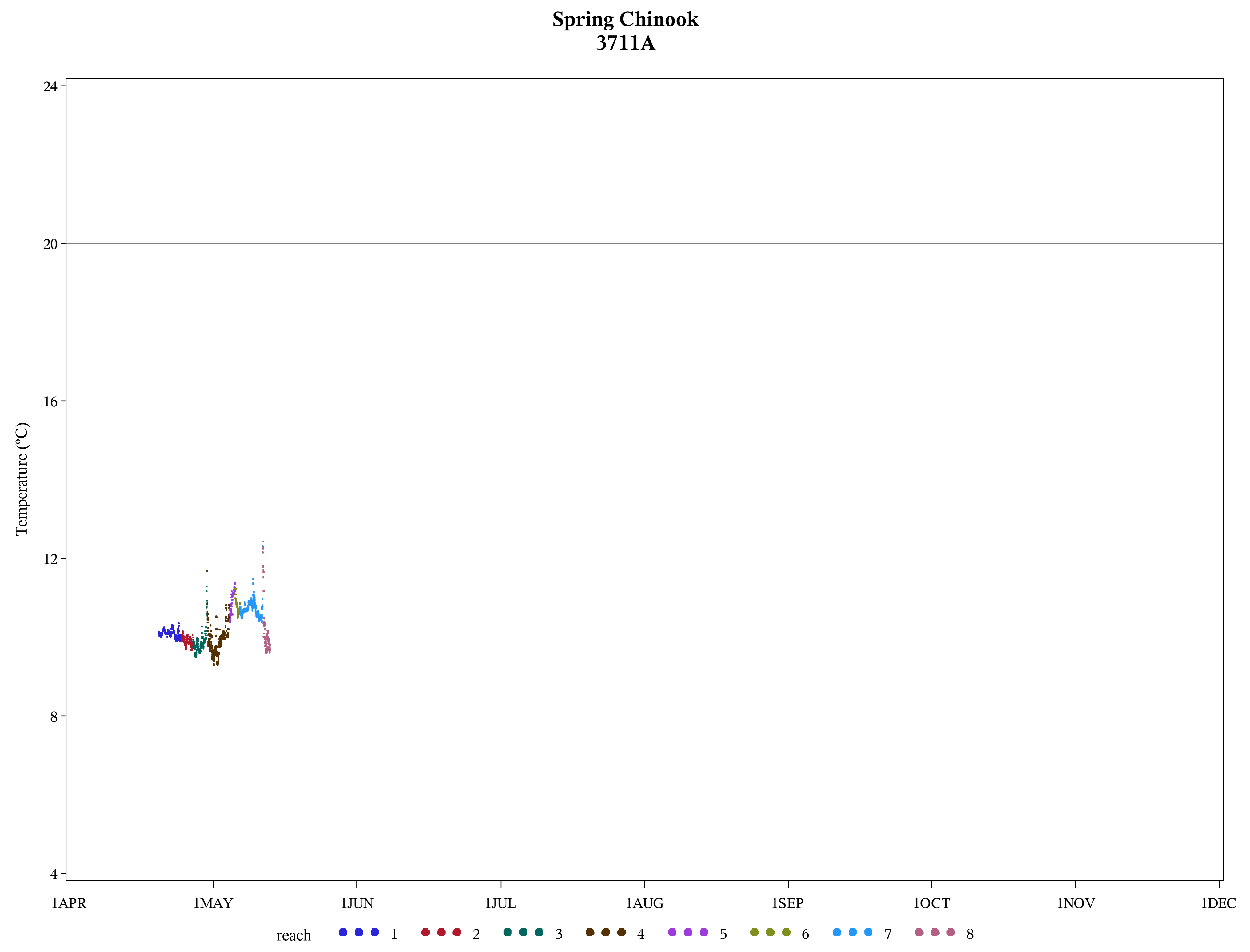

Spring Chinook
3711A
Temperature (°C)
24
20
16
12
8
4
1APR
1MAY
1JUN
1JUL
1AUG
1SEP
1OCT
1NOV
1DEC
reach
1
2
3
4
5
6
7
8

Spring Chinook
3718A

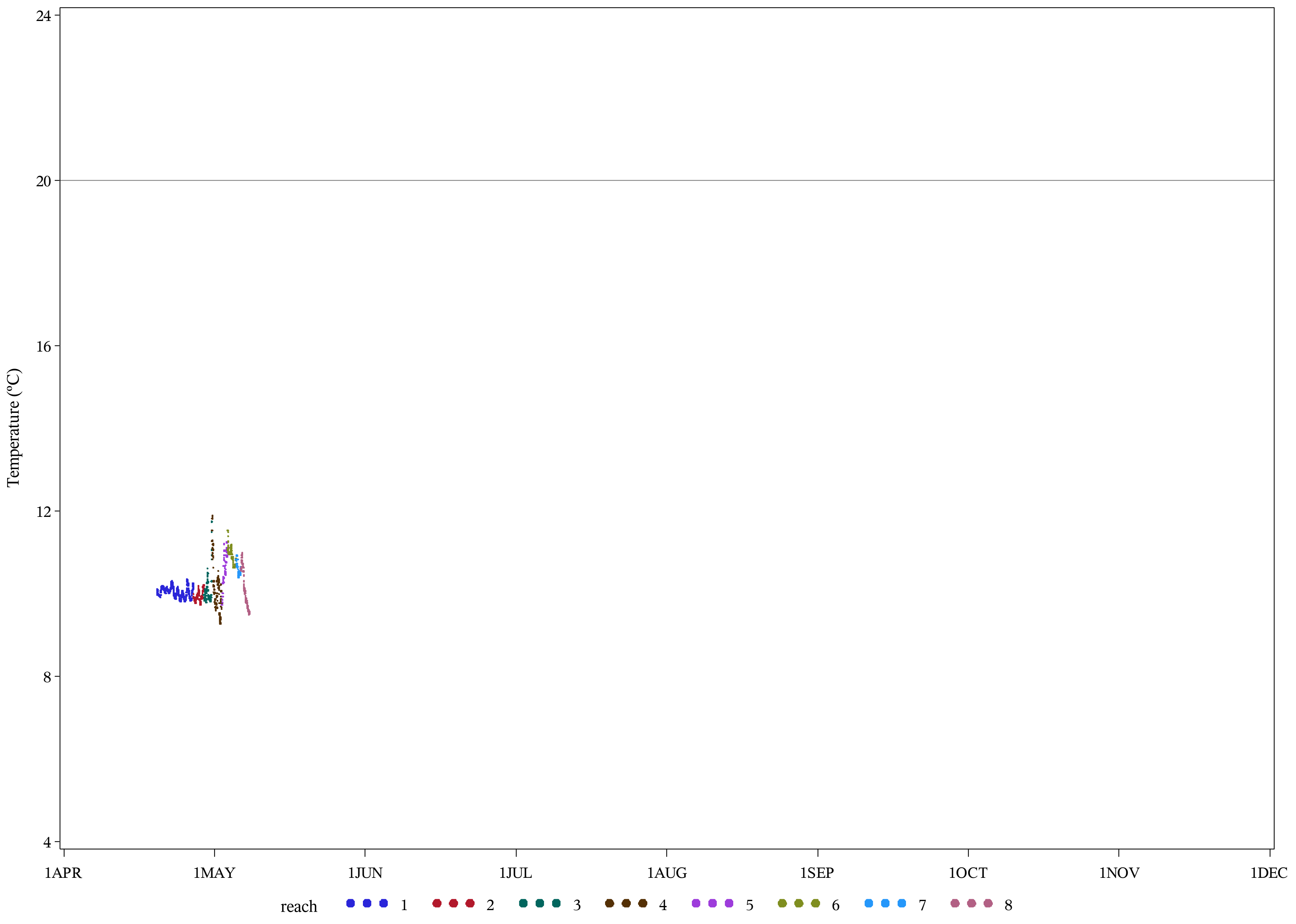

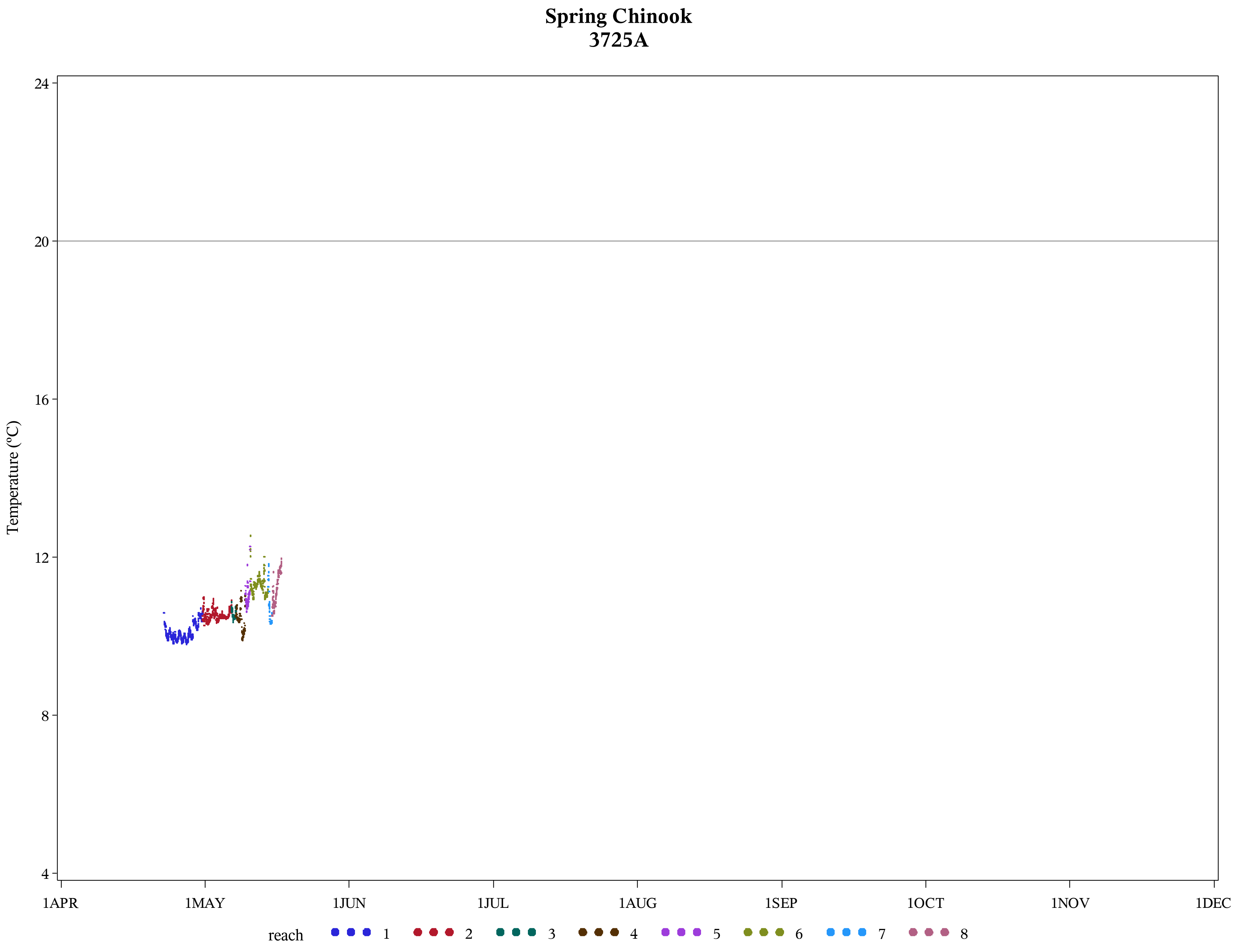
Spring Chinook
3725A
Temperature (°C)
24
20
16
12
8
4
1APR
1MAY
1JUN
1JUL
1AUG
1SEP
1OCT
1NOV
1DEC
reach
1
2
3
4
5
6
7
8

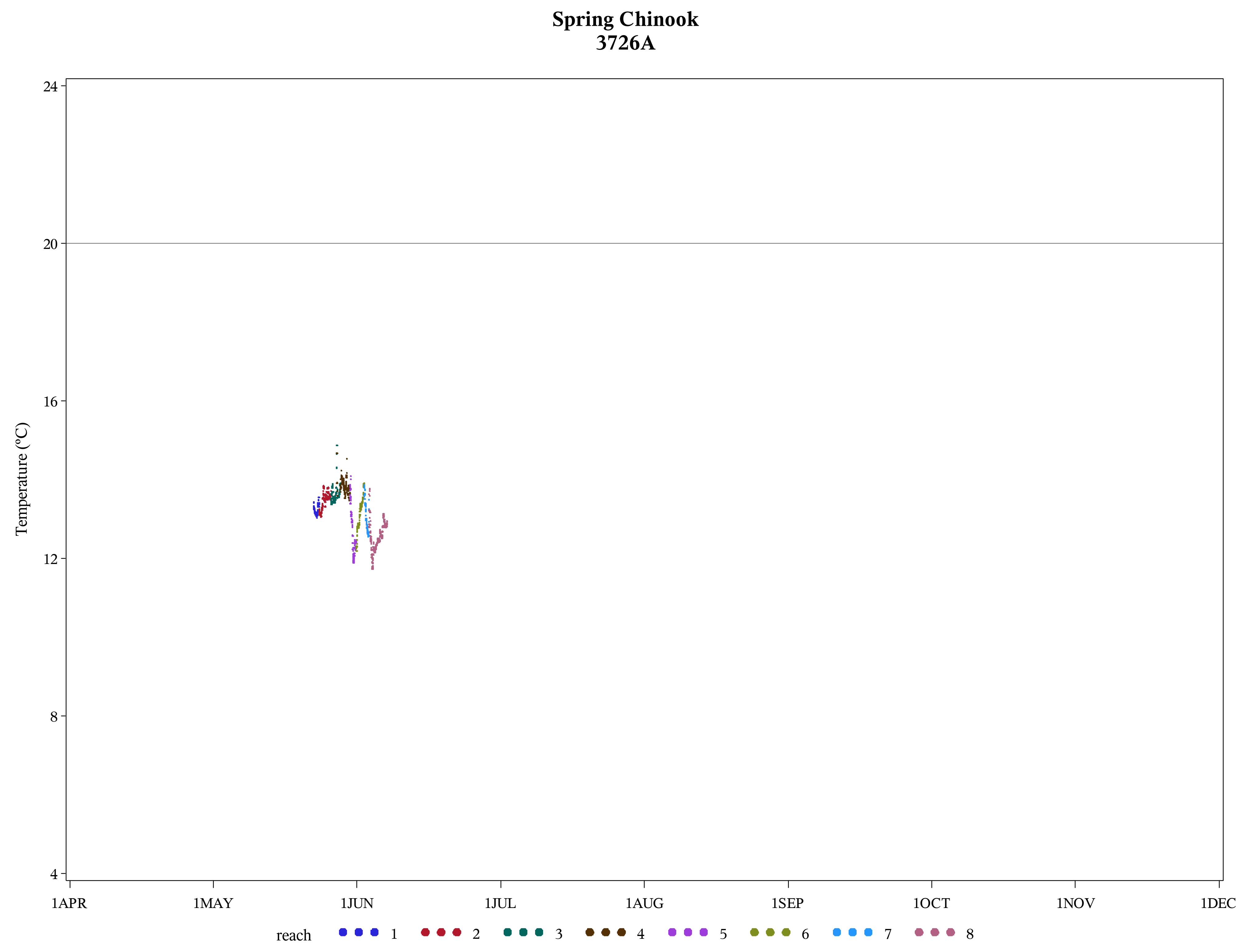
Spring Chinook
3726A
Temperature (°C)
24
20
16
12
8
4
1APR
1MAY
1JUN
1JUL
1AUG
1SEP
1OCT
1NOV
1DEC
reach
1
2
3
4
5
6
7
8

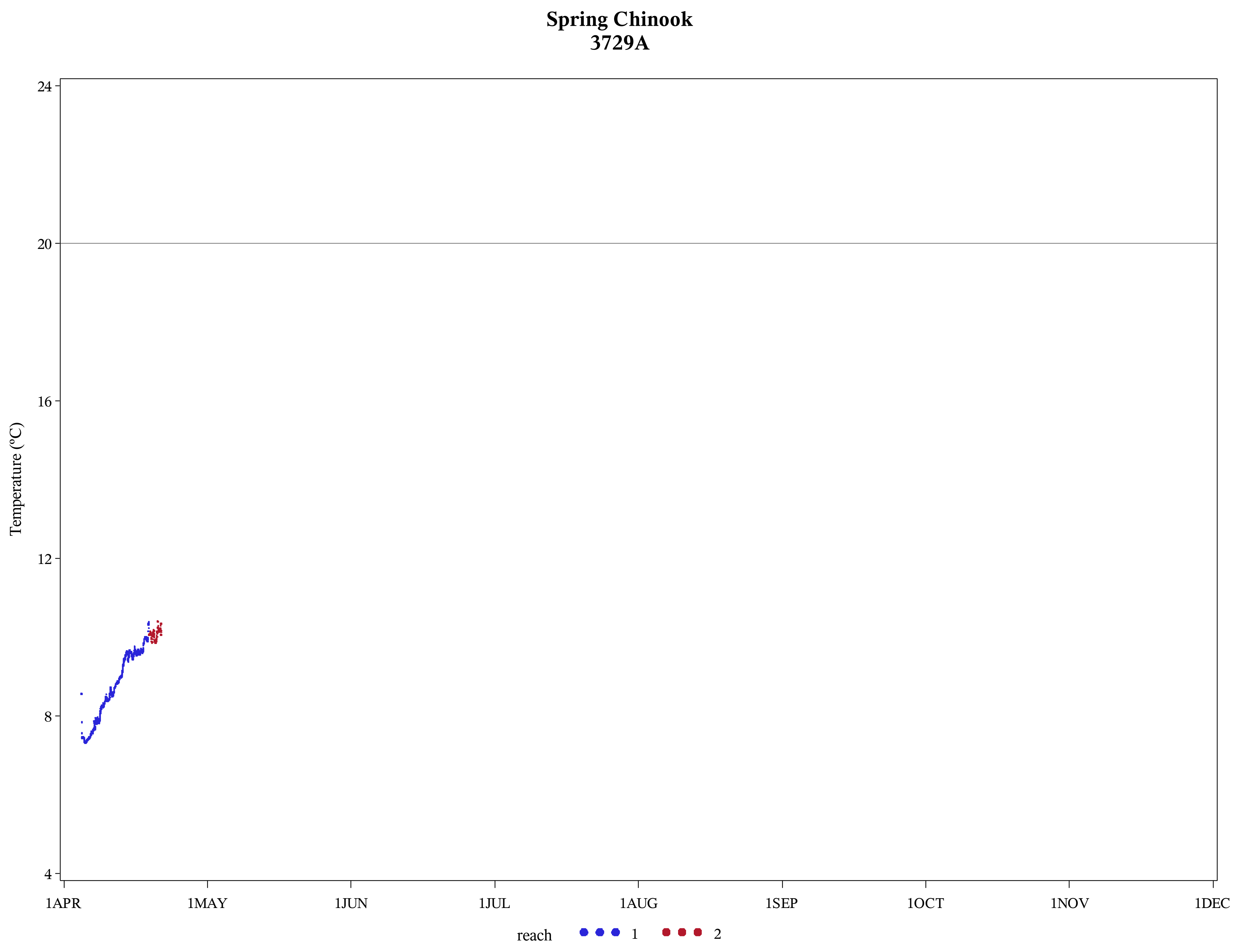

Spring Chinook
3729A
Temperature (°C)
24
20
16
12
8
4
1APR
1MAY
1JUN
1JUL
1AUG
1SEP
1OCT
1NOV
1DEC
reach
1
2

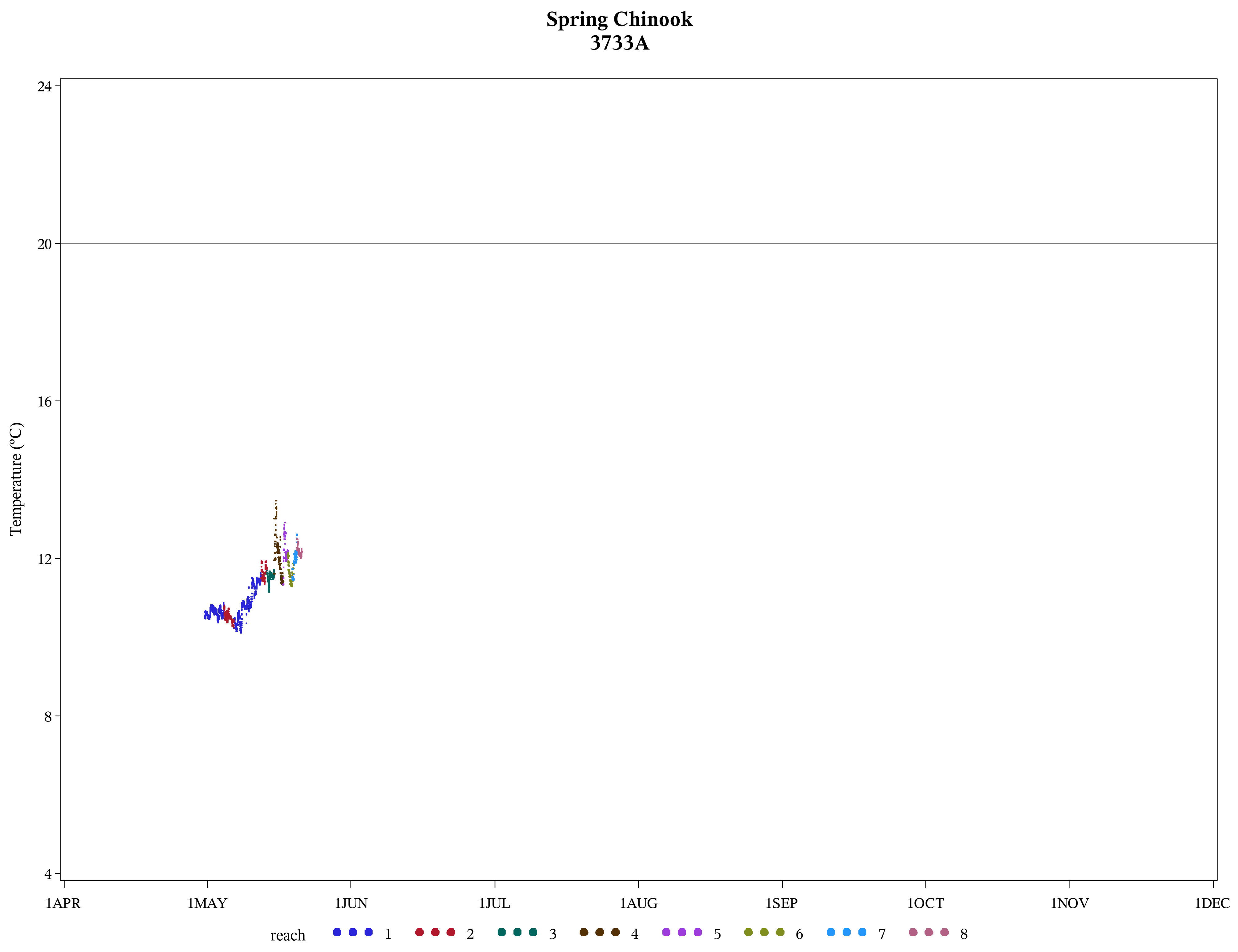

Spring Chinook
3733A
Temperature (°C)
24
20
16
12
8
4
1APR
1MAY
1JUN
1JUL
1AUG
1SEP
1OCT
1NOV
1DEC
reach
1
2
3
4
5
6
7
8

Spring Chinook
3761A

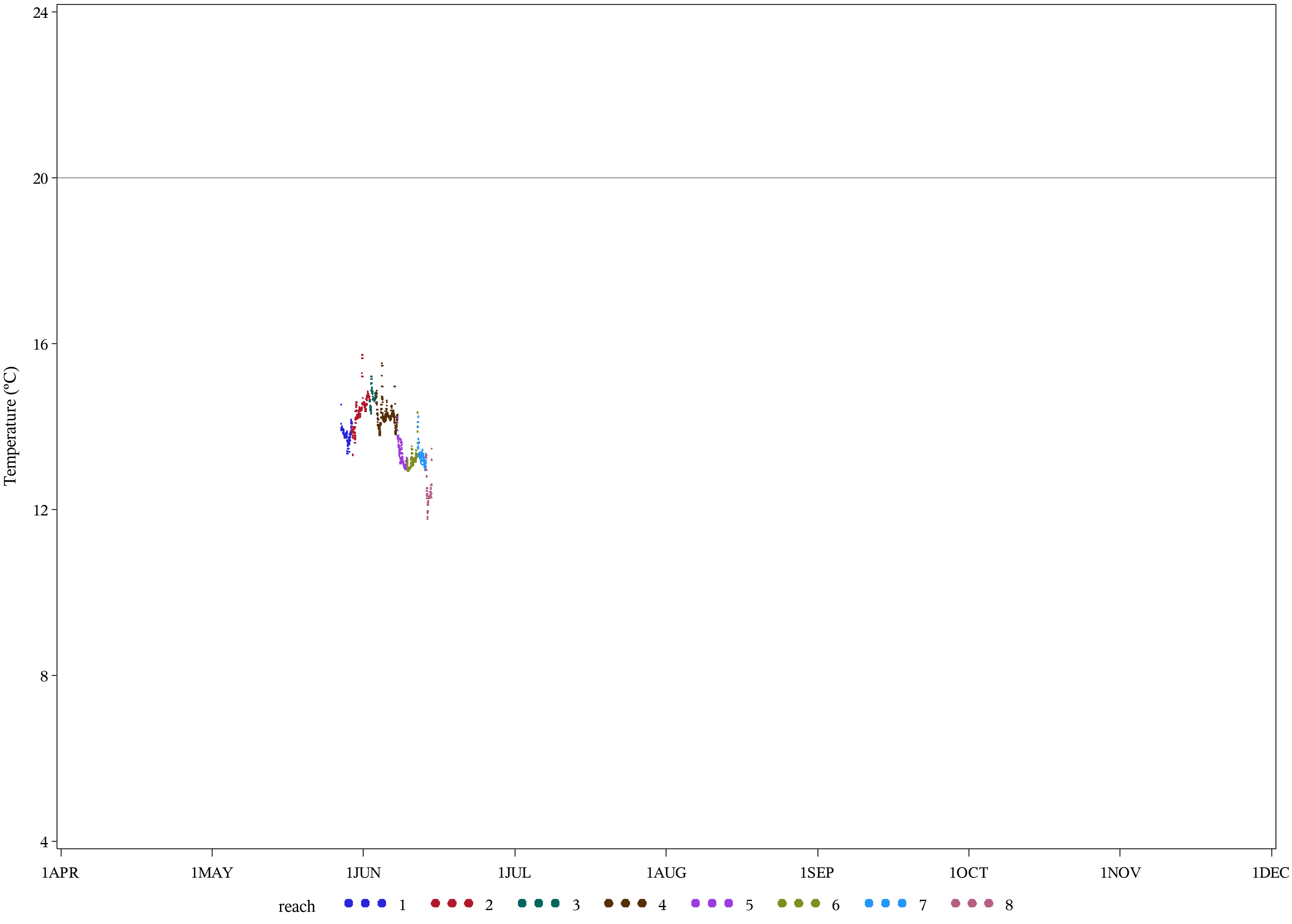

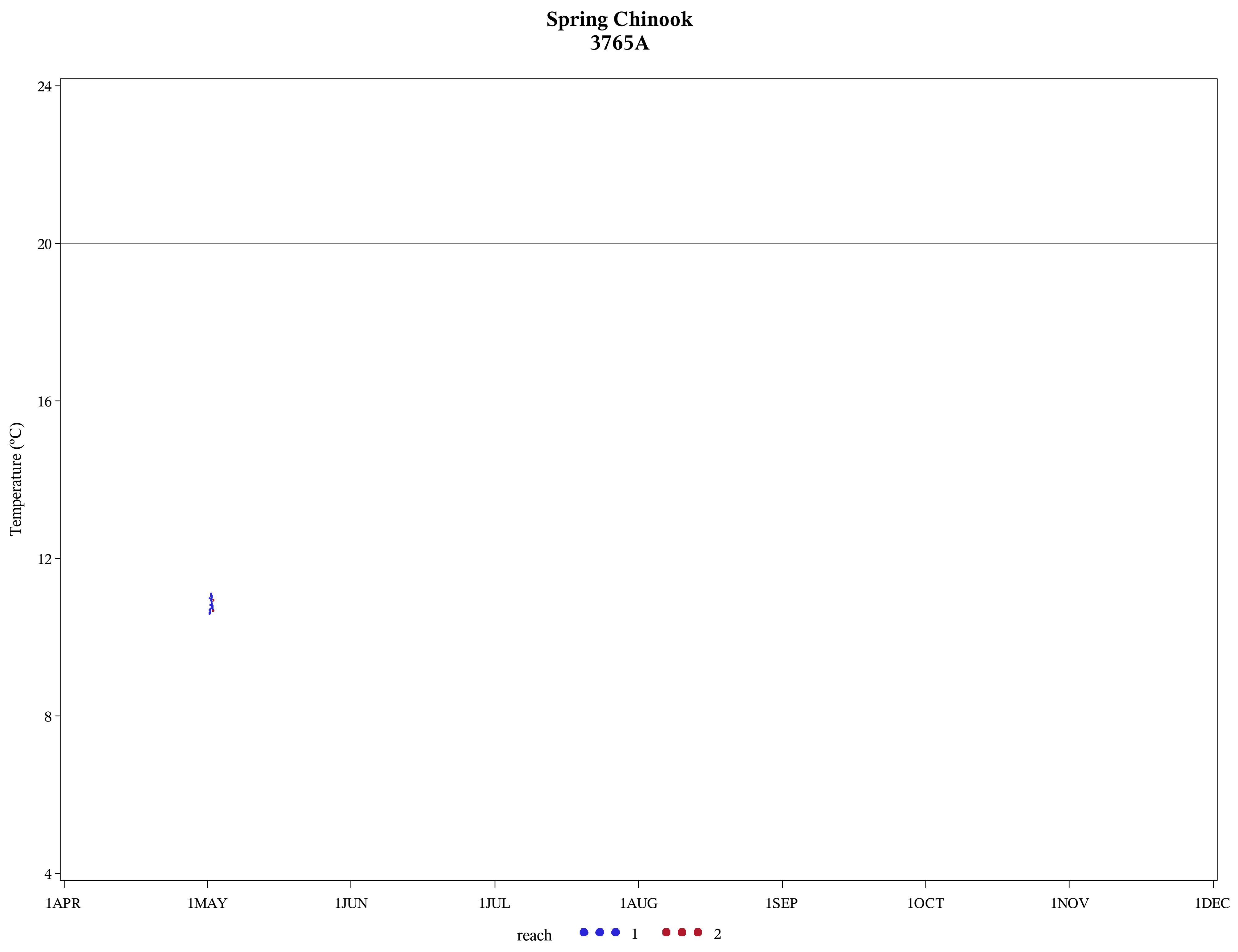

Spring Chinook
3765A
Temperature (ºC)
24
20
16
12
8
4
1APR
1MAY
1JUN
1JUL
1AUG
1SEP
1OCT
1NOV
1DEC
reach
1
2

Spring Chinook
3768A

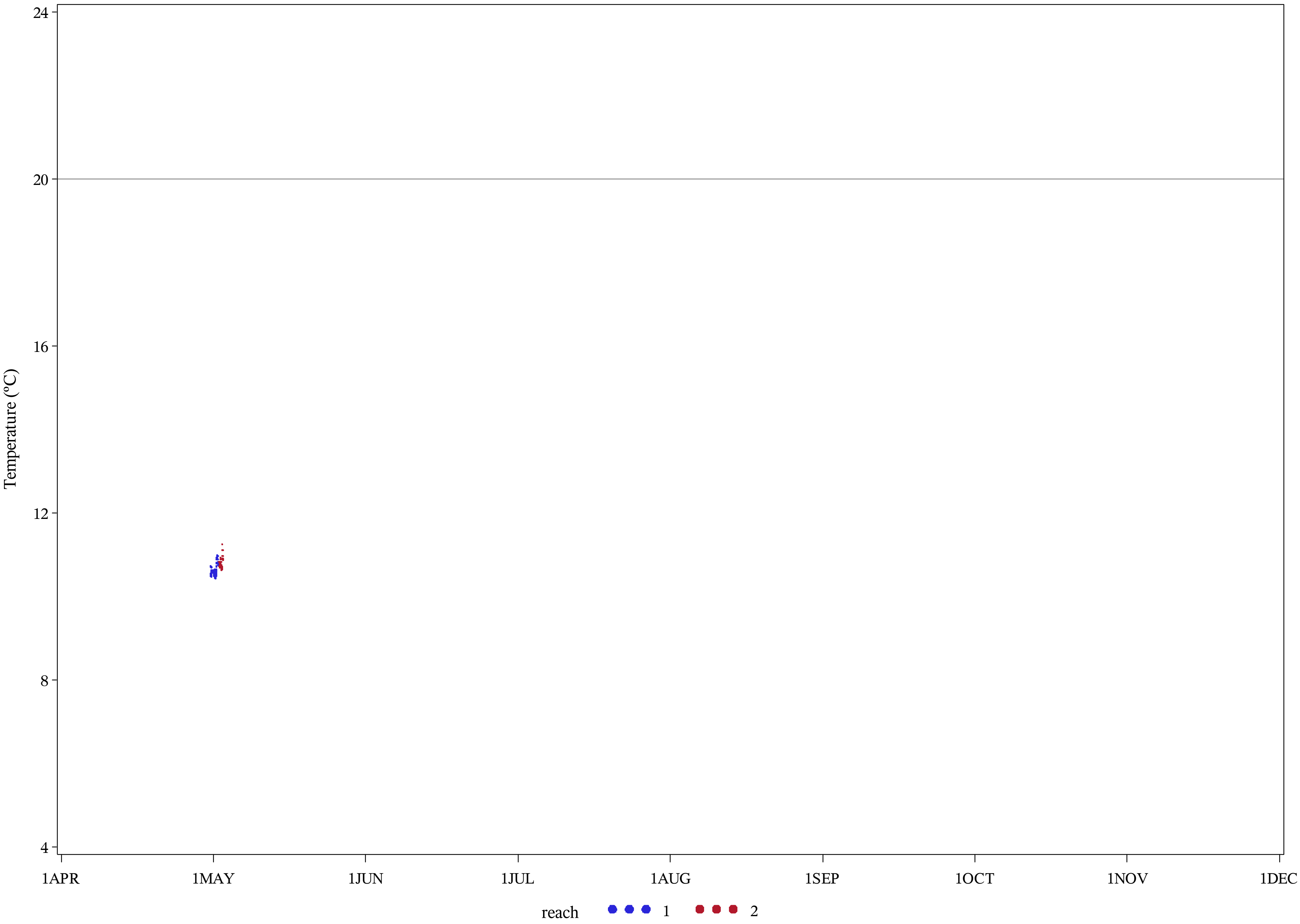

Spring Chinook
3769A

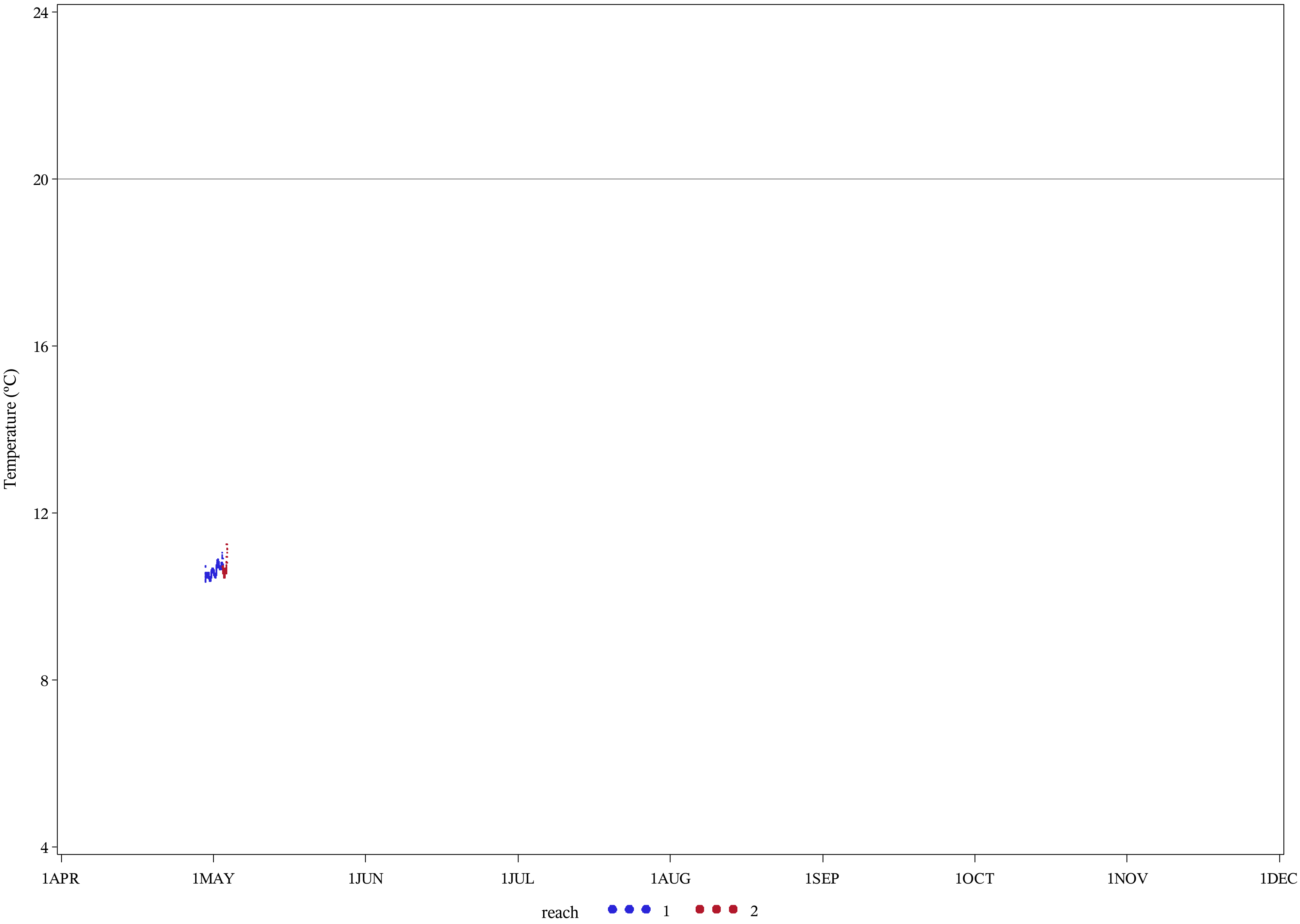

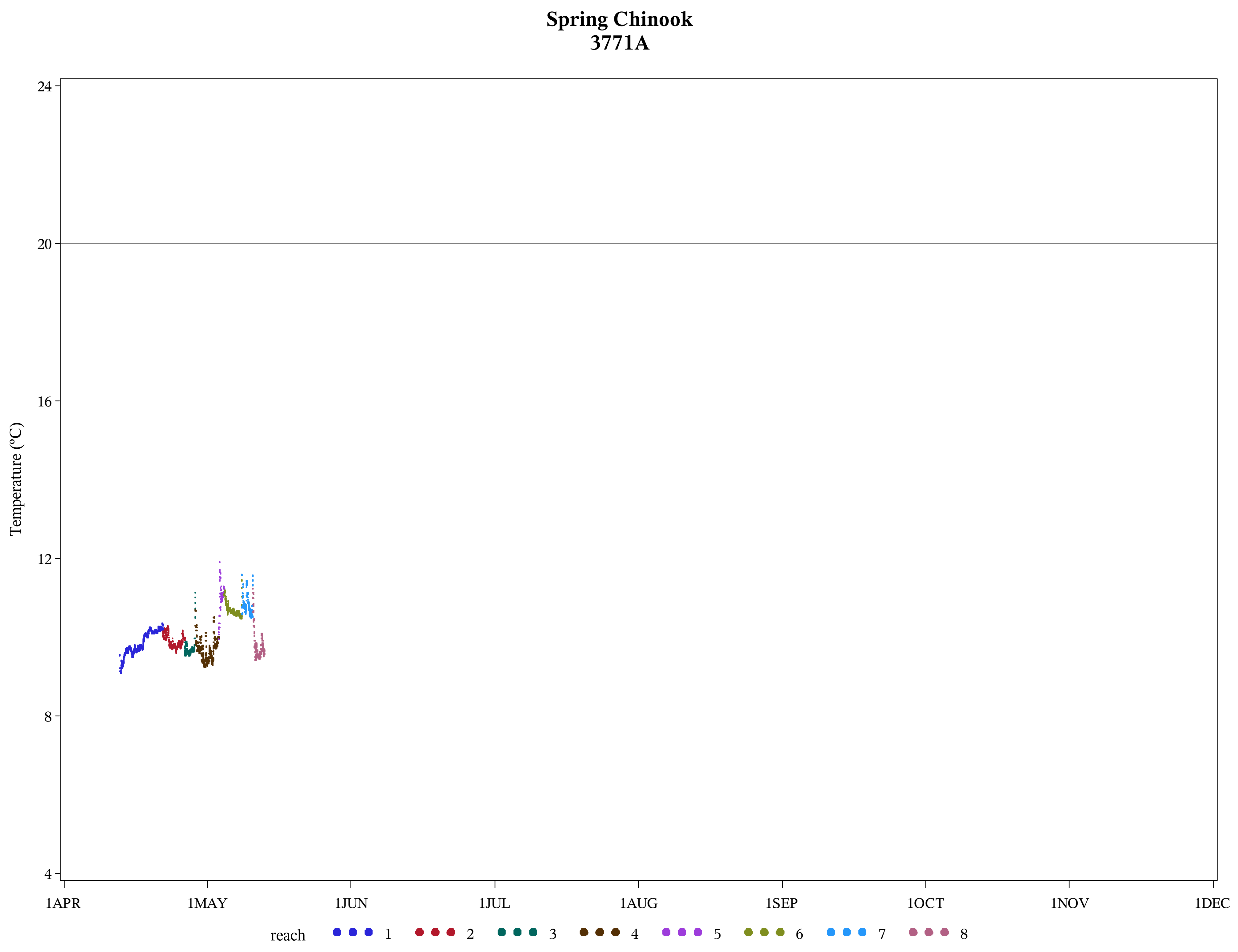

Spring Chinook
3771A
Temperature (ºC)
24
20
16
12
8
4
1APR
1MAY
1JUN
1JUL
1AUG
1SEP
1OCT
1NOV
1DEC
reach
1
2
3
4
5
6
7
8

Spring Chinook
3773A

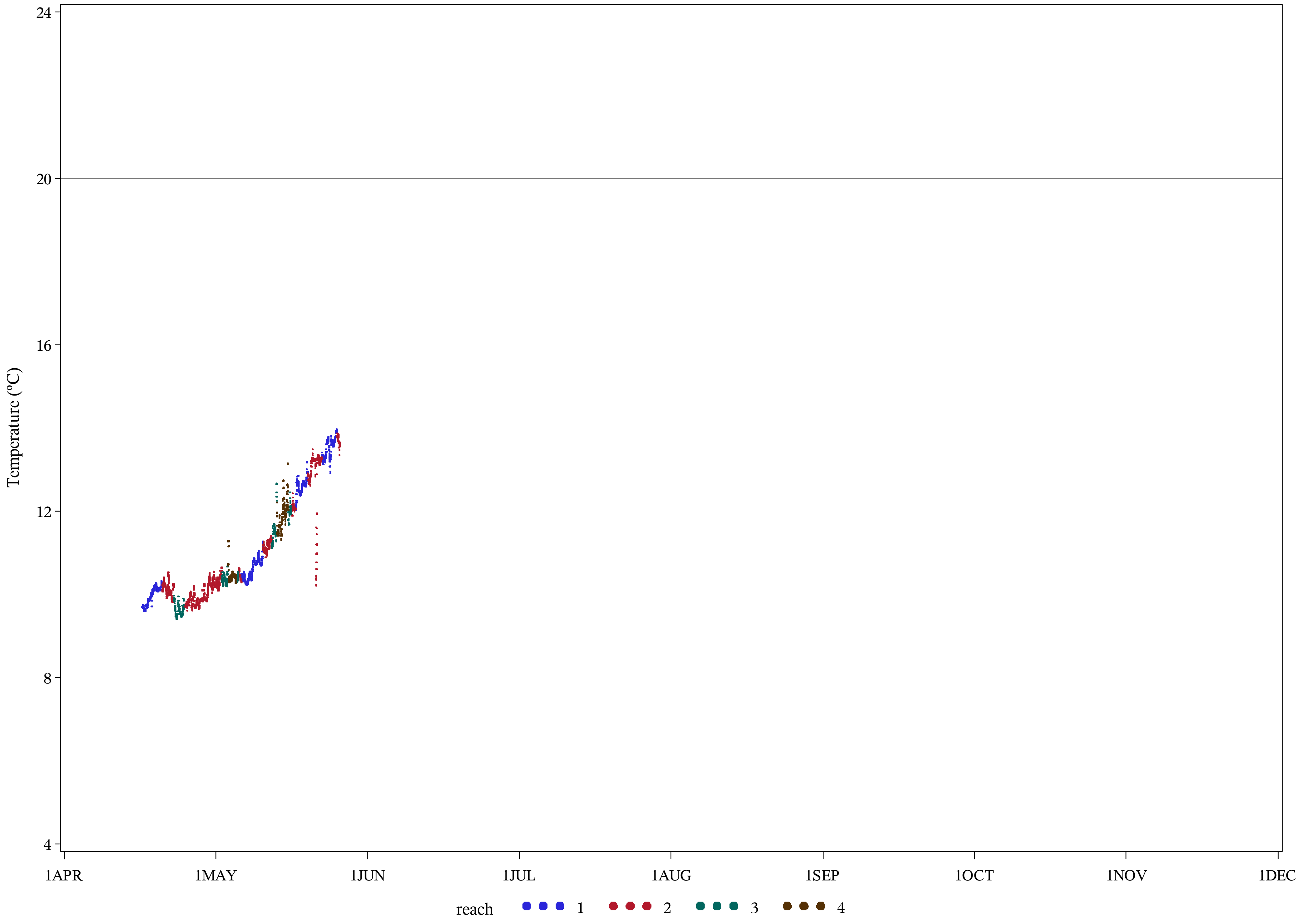

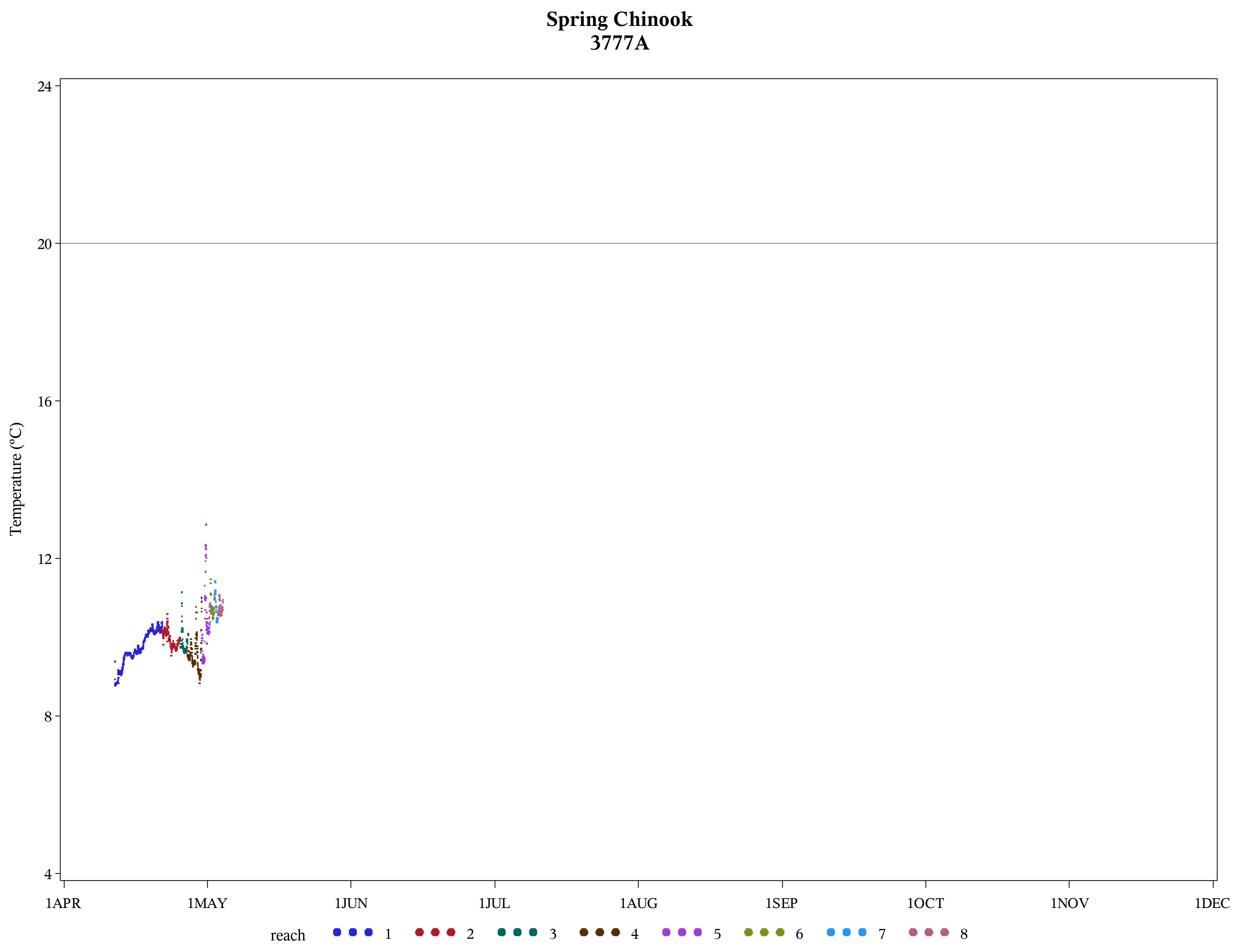
Spring Chinook
3777A
Temperature (°C)
24
20
16
12
8
4
1APR
1MAY
1JUN
1JUL
1AUG
1SEP
1OCT
1NOV
1DEC
reach
1
2
3
4
5
6
7
8

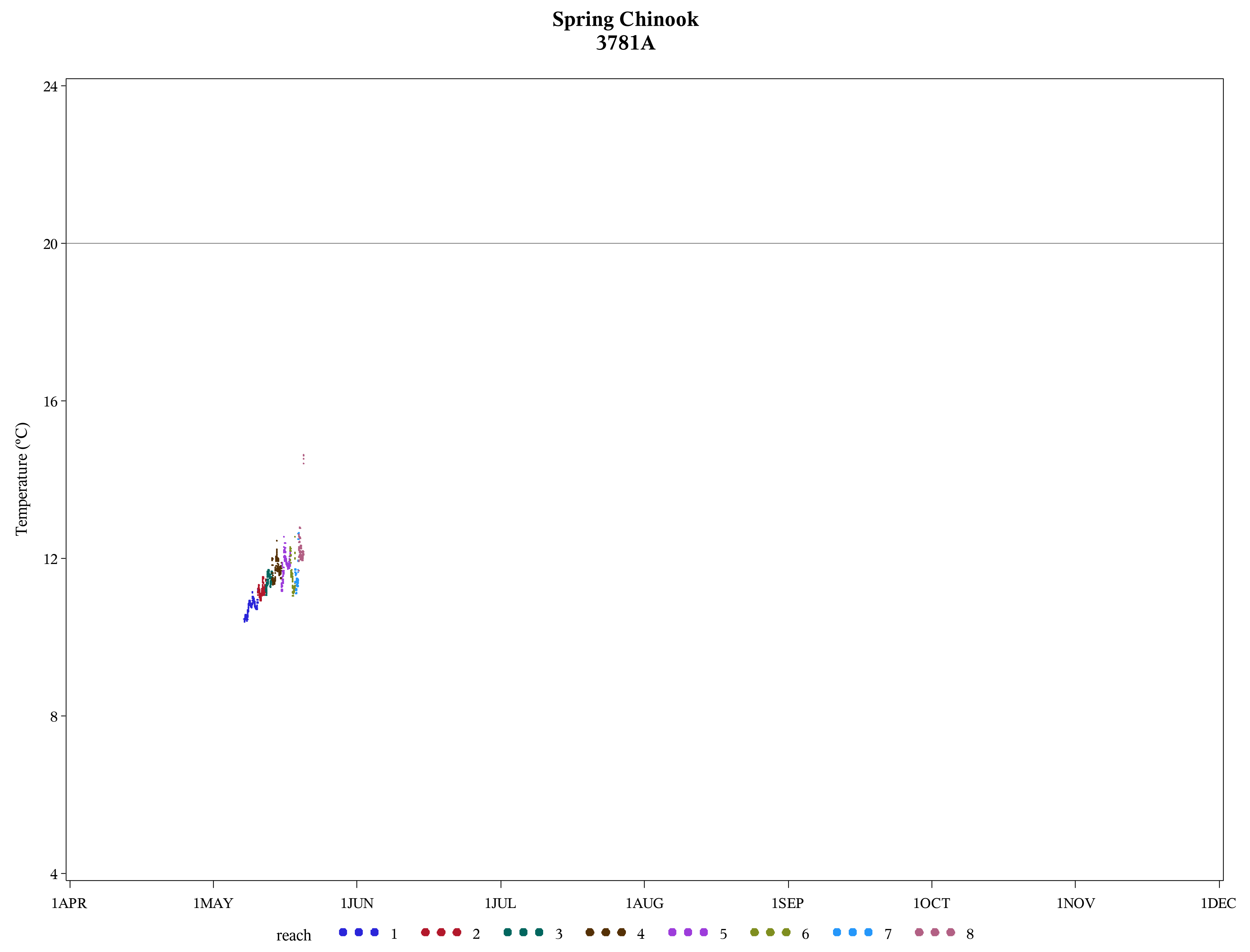
Spring Chinook
3781A
Temperature (°C)
24
20
16
12
8
4
1APR
1MAY
1JUN
1JUL
1AUG
1SEP
1OCT
1NOV
1DEC
reach
1
2
3
4
5
6
7
8

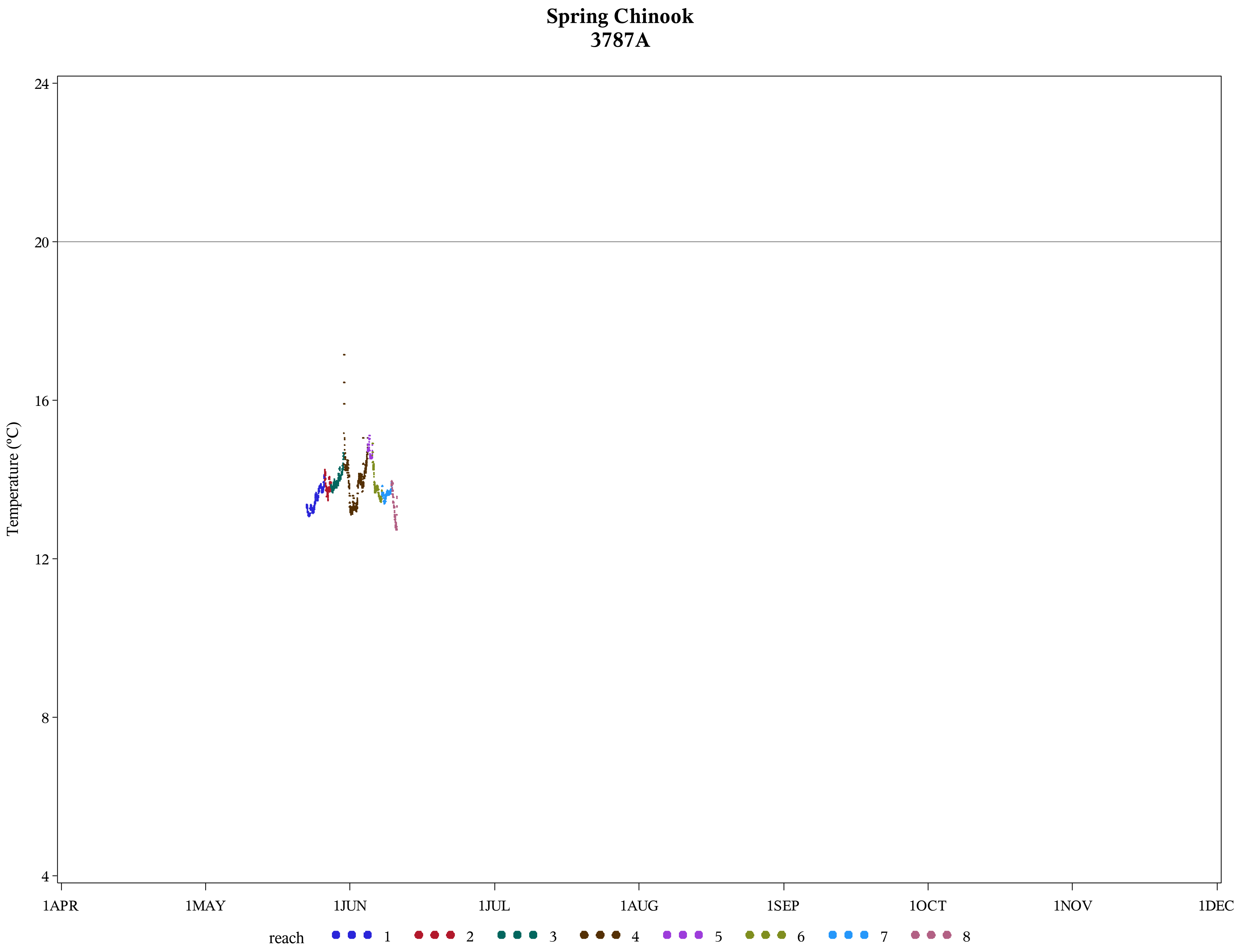

Spring Chinook
3787A
Temperature (°C)
24
20
16
12
8
4
1APR
1MAY
1JUN
1JUL
1AUG
1SEP
1OCT
1NOV
1DEC
reach
1
2
3
4
5
6
7
8

Spring Chinook
3789A

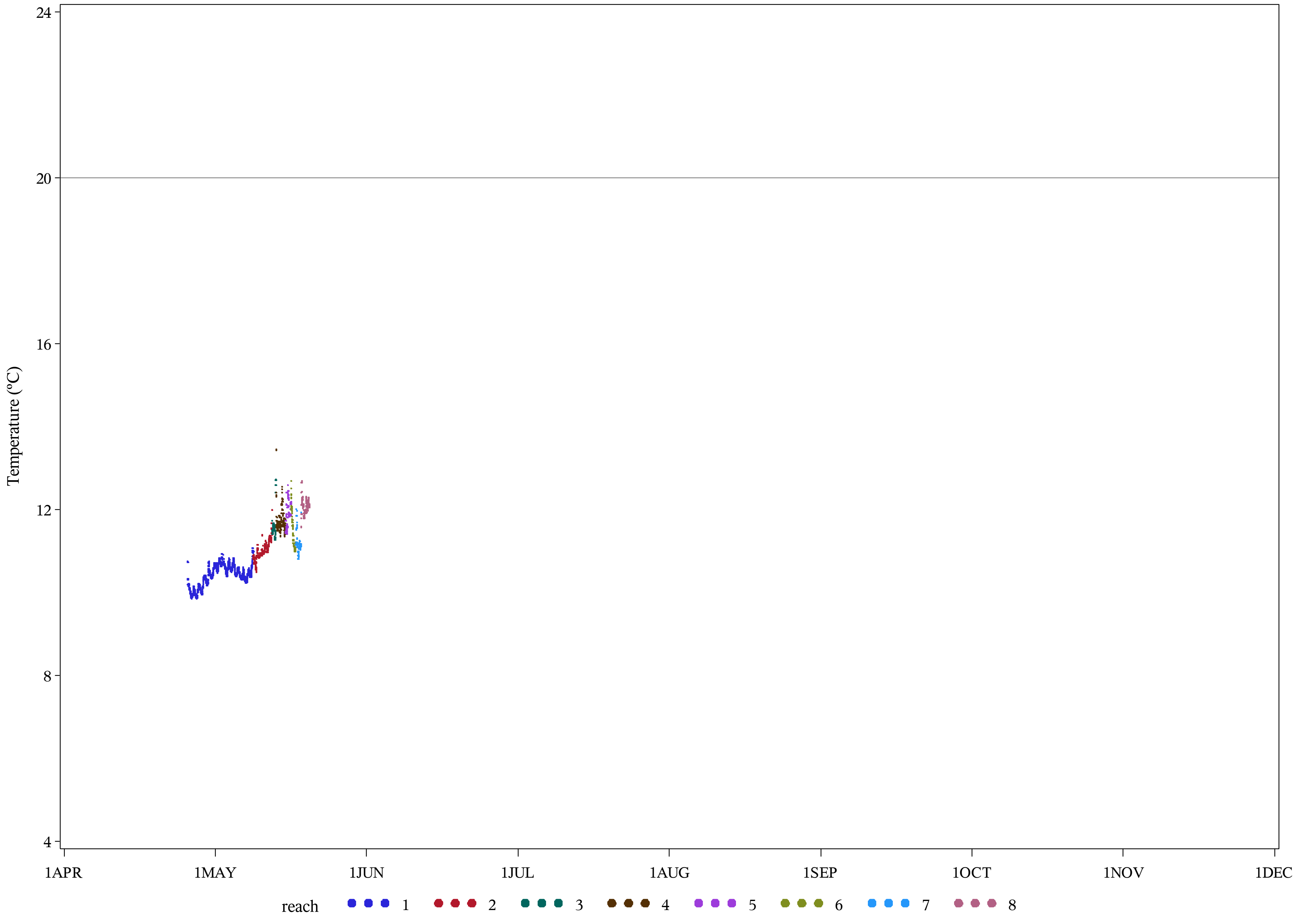

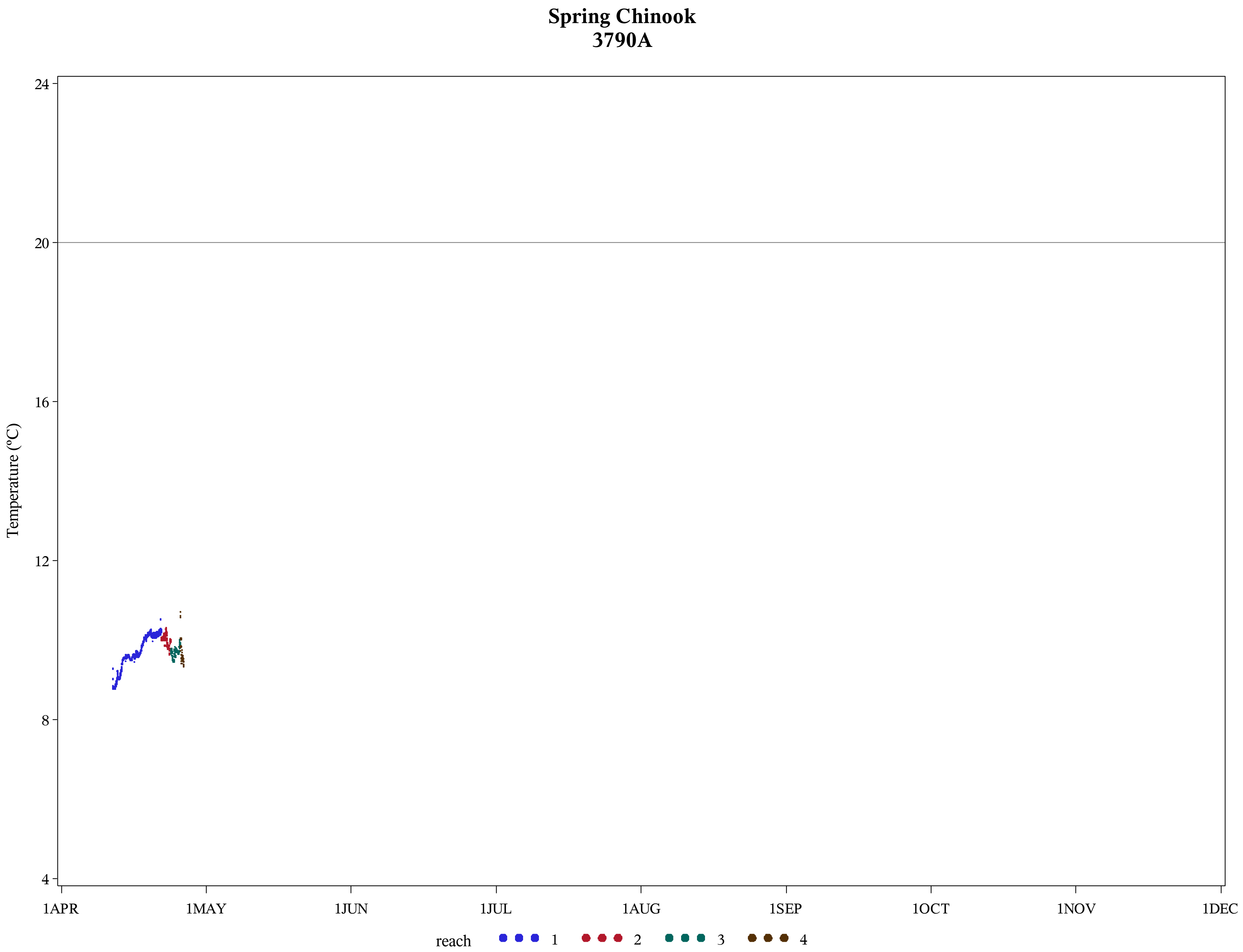

Spring Chinook
3790A
24
20
16
12
8
4
Temperature (°C)
1APR
1MAY
1JUN
1JUL
1AUG
1SEP
1OCT
1NOV
1DEC
reach
1
2
3
4

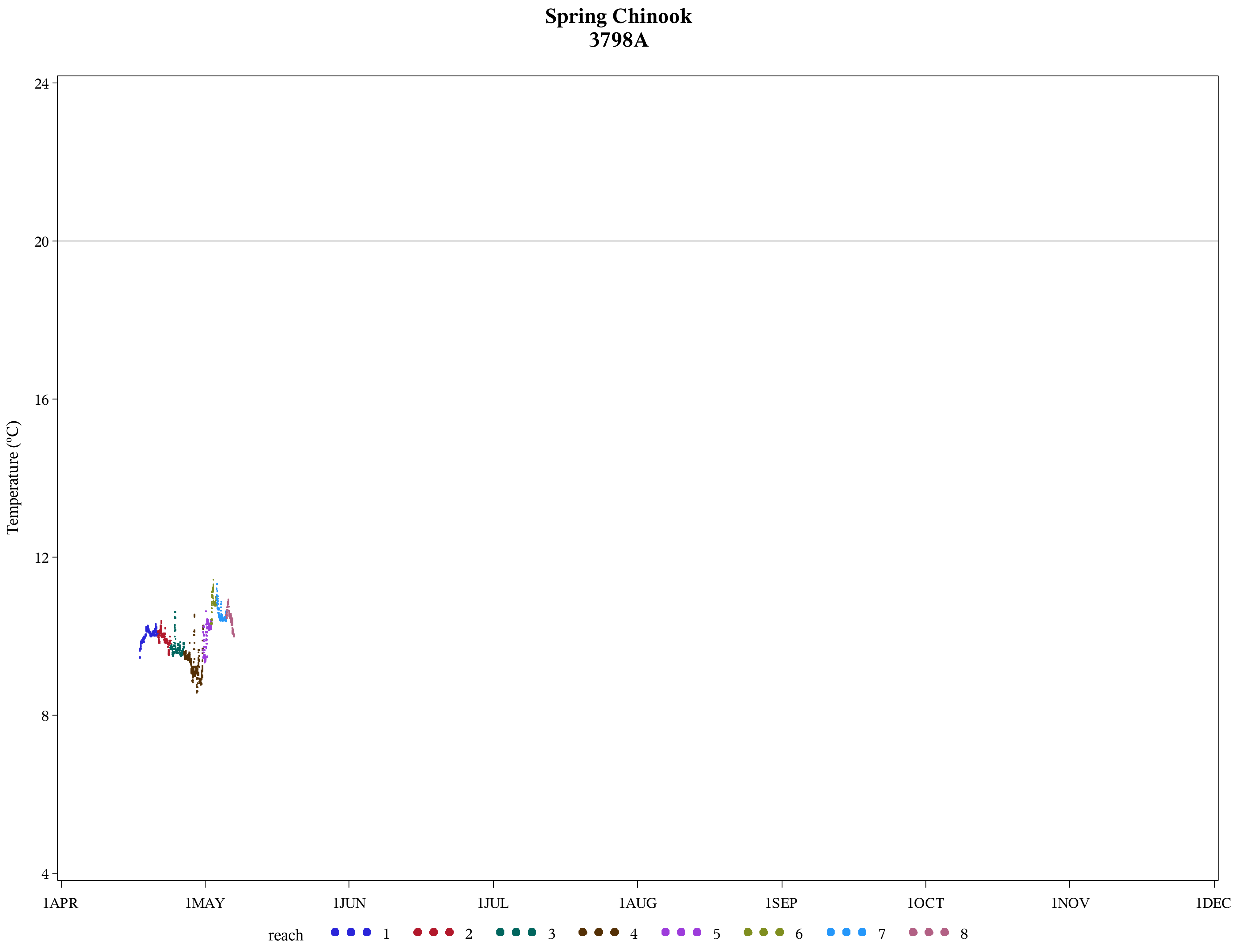

Spring Chinook
3798A
Temperature (°C)
24
20
16
12
8
4
1APR
1MAY
1JUN
1JUL
1AUG
1SEP
1OCT
1NOV
1DEC
reach
1
2
3
4
5
6
7
8

Spring Chinook
3805A

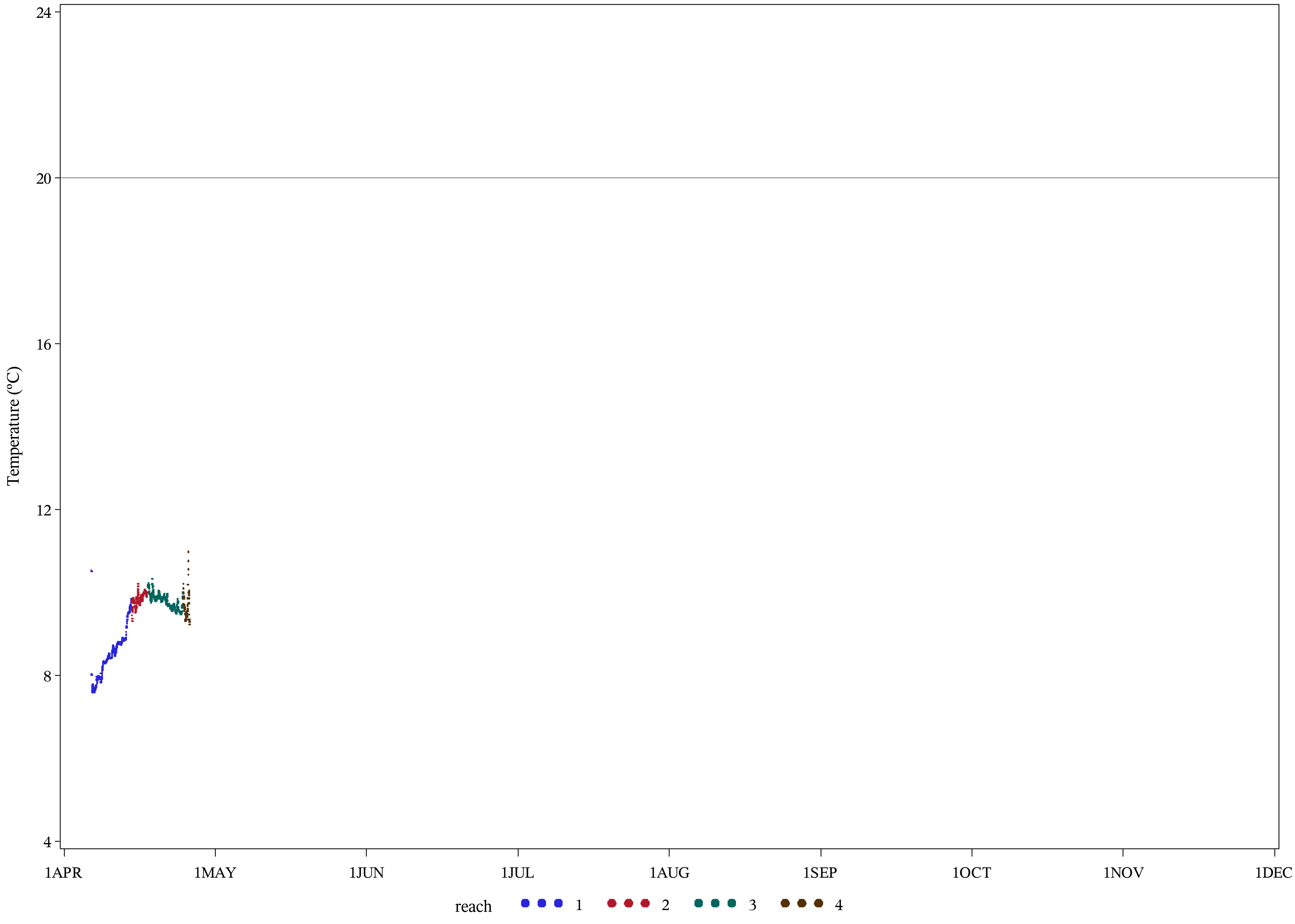

Spring Chinook
3816A

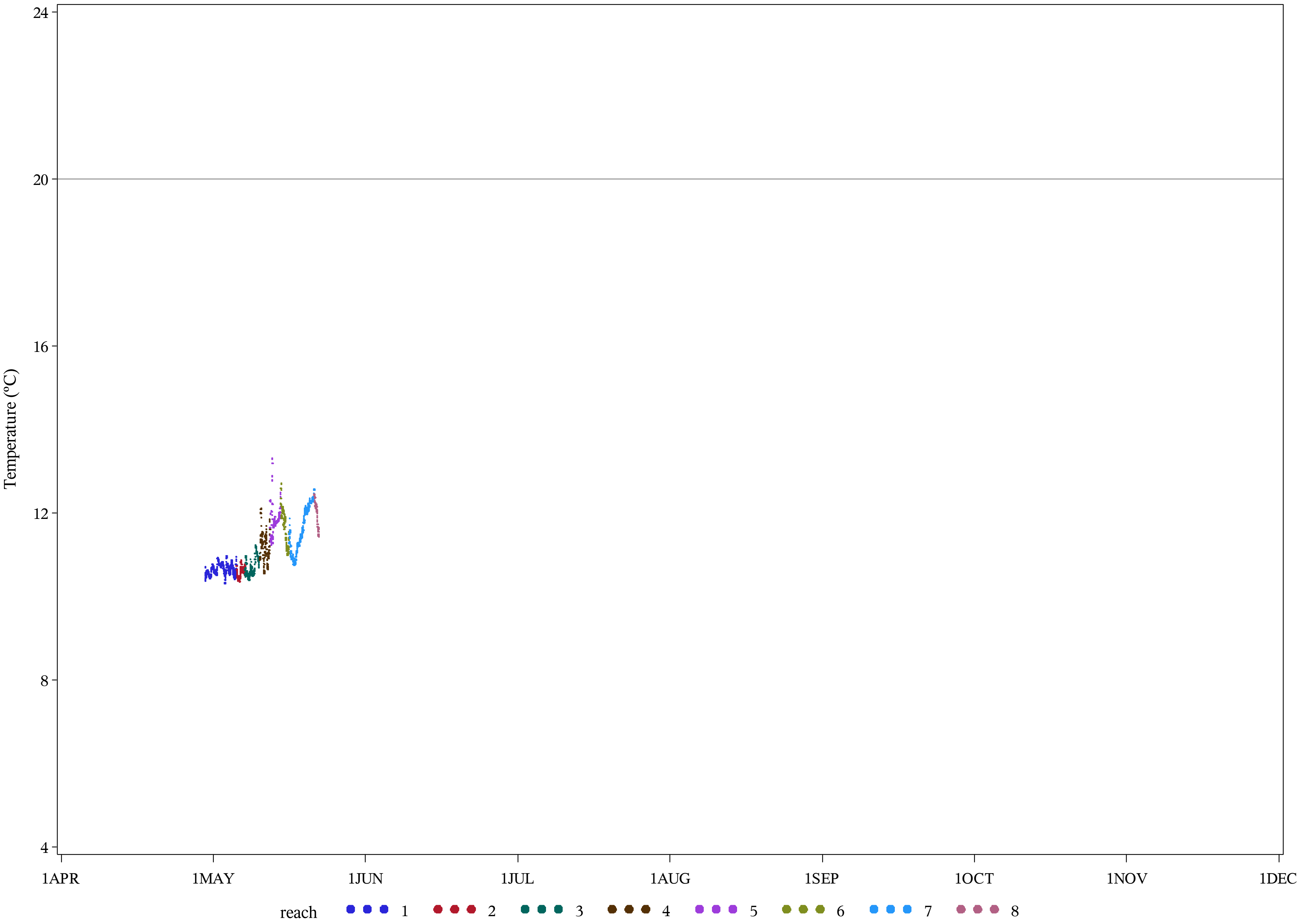

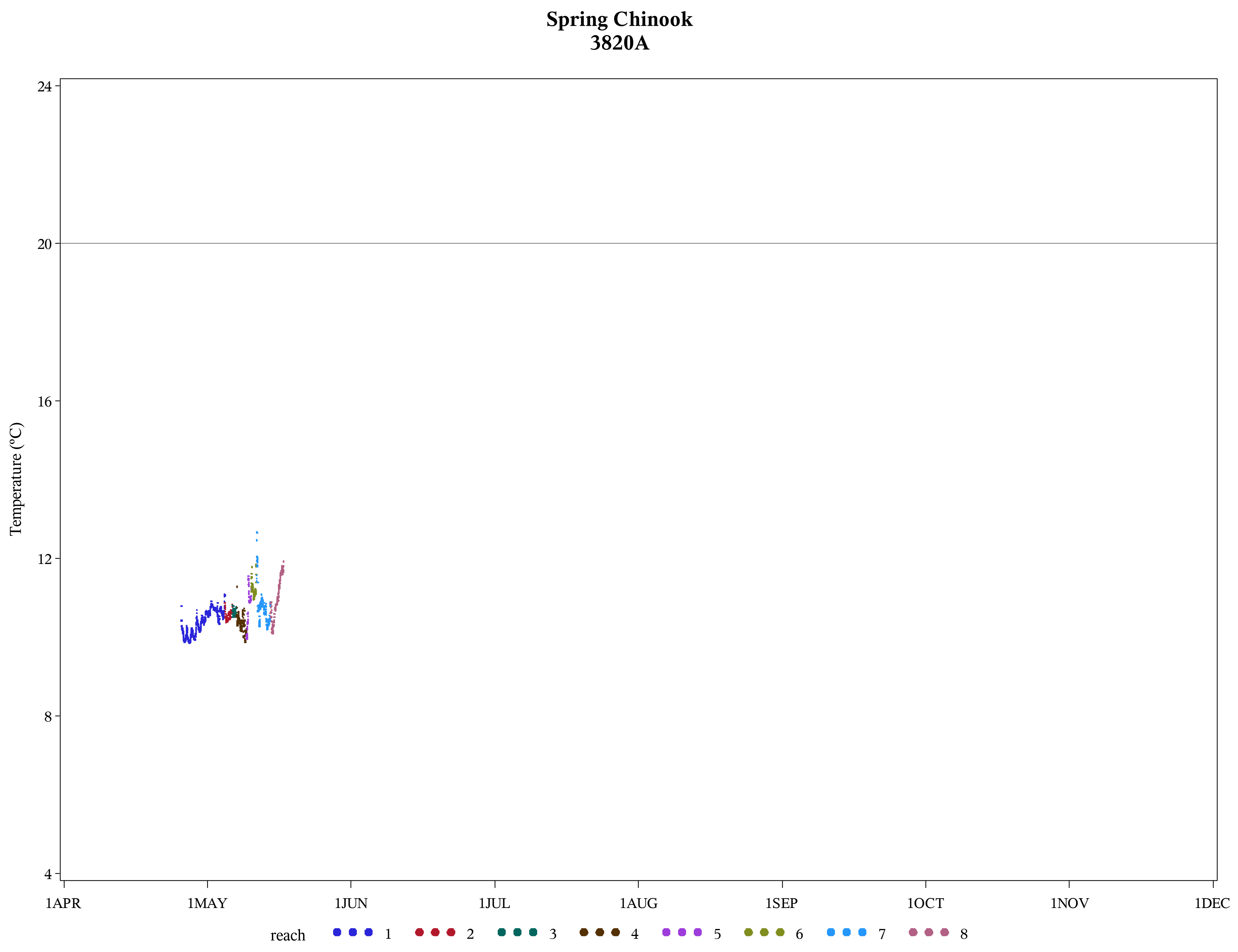

Spring Chinook
3820A
Temperature (°C)
24
20
16
12
8
4
1APR
1MAY
1JUN
1JUL
1AUG
1SEP
1OCT
1NOV
1DEC
reach
1
2
3
4
5
6
7
8

Spring Chinook
3828A

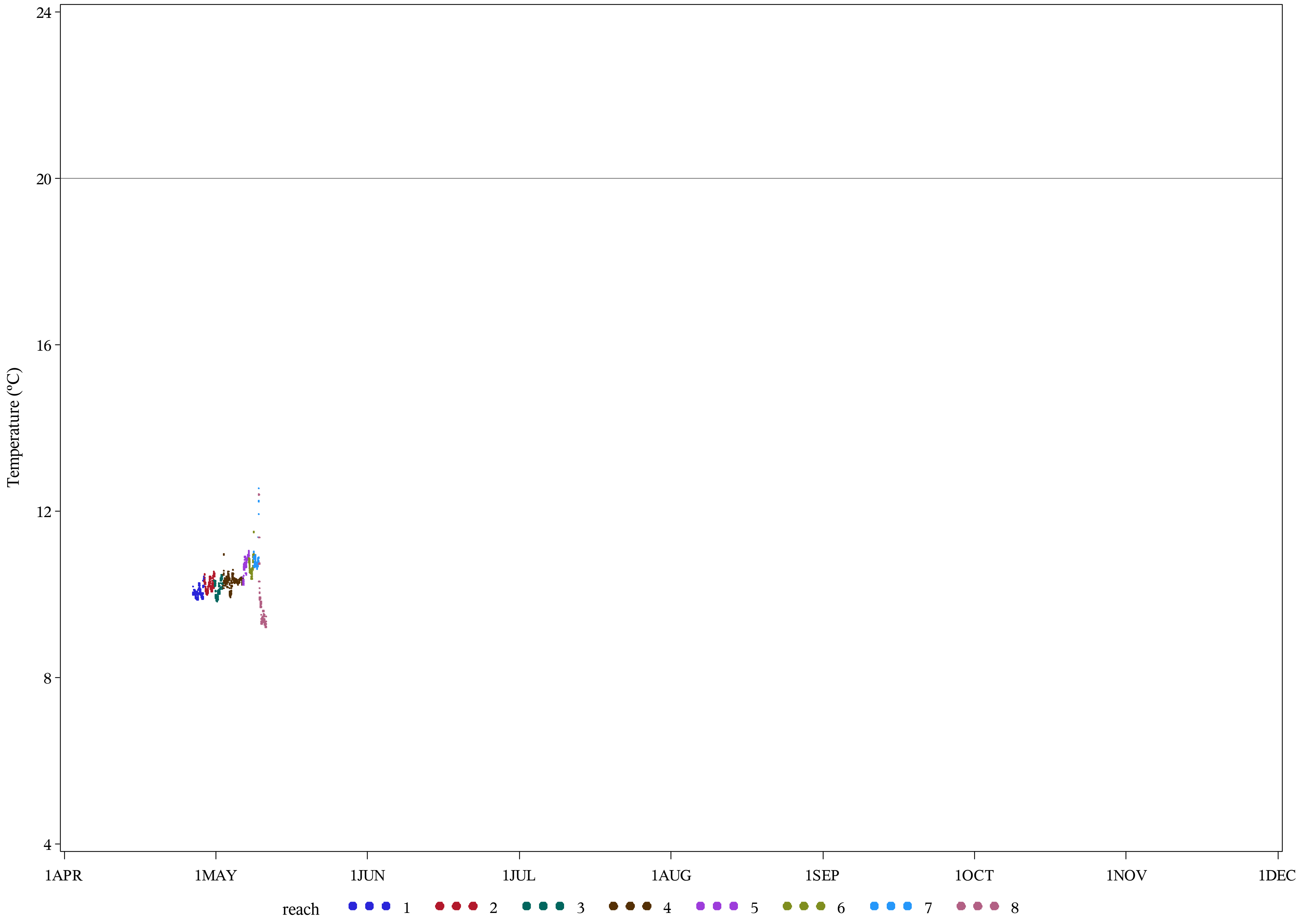

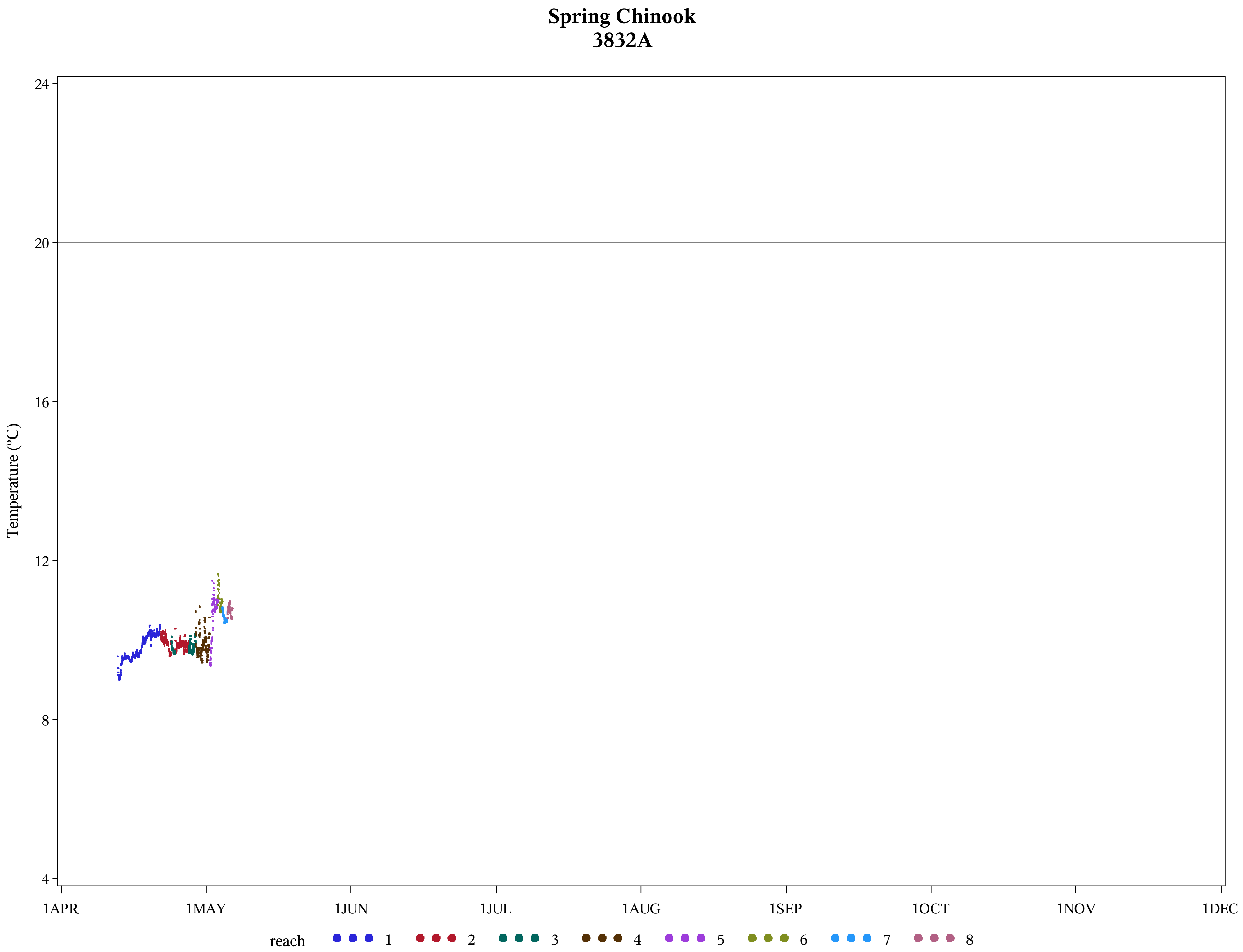

Spring Chinook
3832A
Temperature (°C)
24
20
16
12
8
4
1APR
1MAY
1JUN
1JUL
1AUG
1SEP
1OCT
1NOV
1DEC
reach
1
2
3
4
5
6
7
8

Spring Chinook
3836A

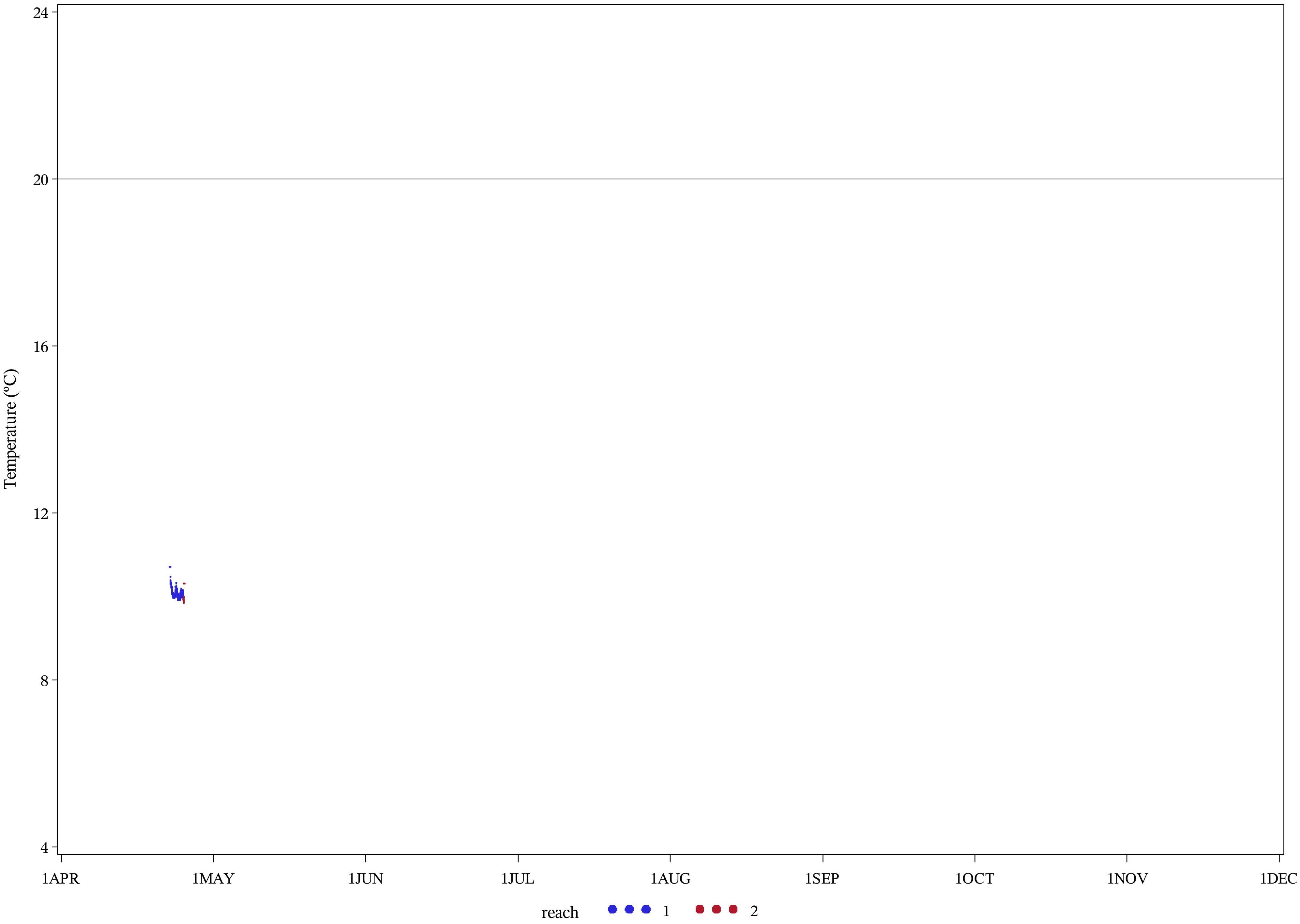

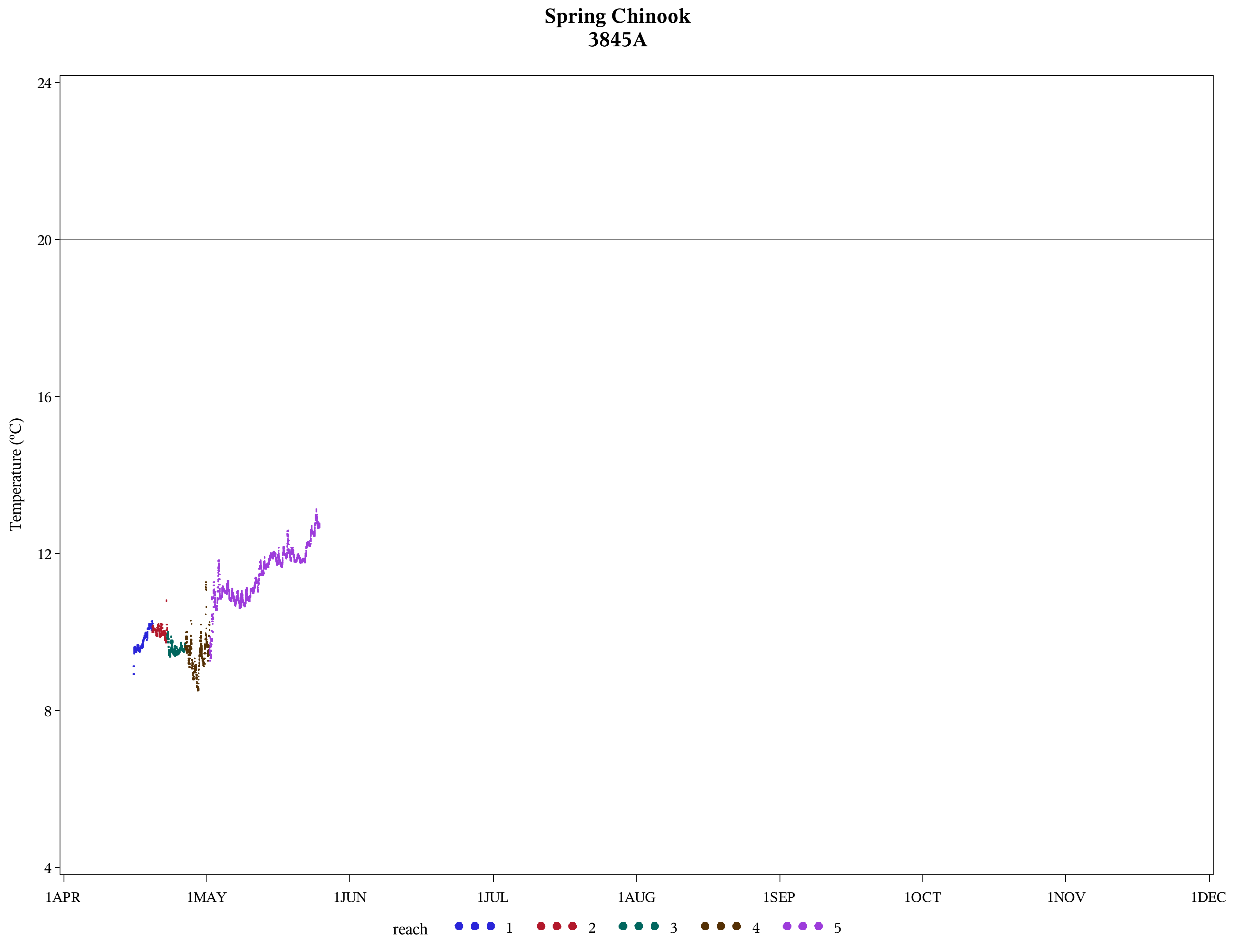

Spring Chinook
3845A
Temperature (°C)
24
20
16
12
8
4
1APR
1MAY
1JUN
1JUL
1AUG
1SEP
1OCT
1NOV
1DEC
reach
1
2
3
4
5

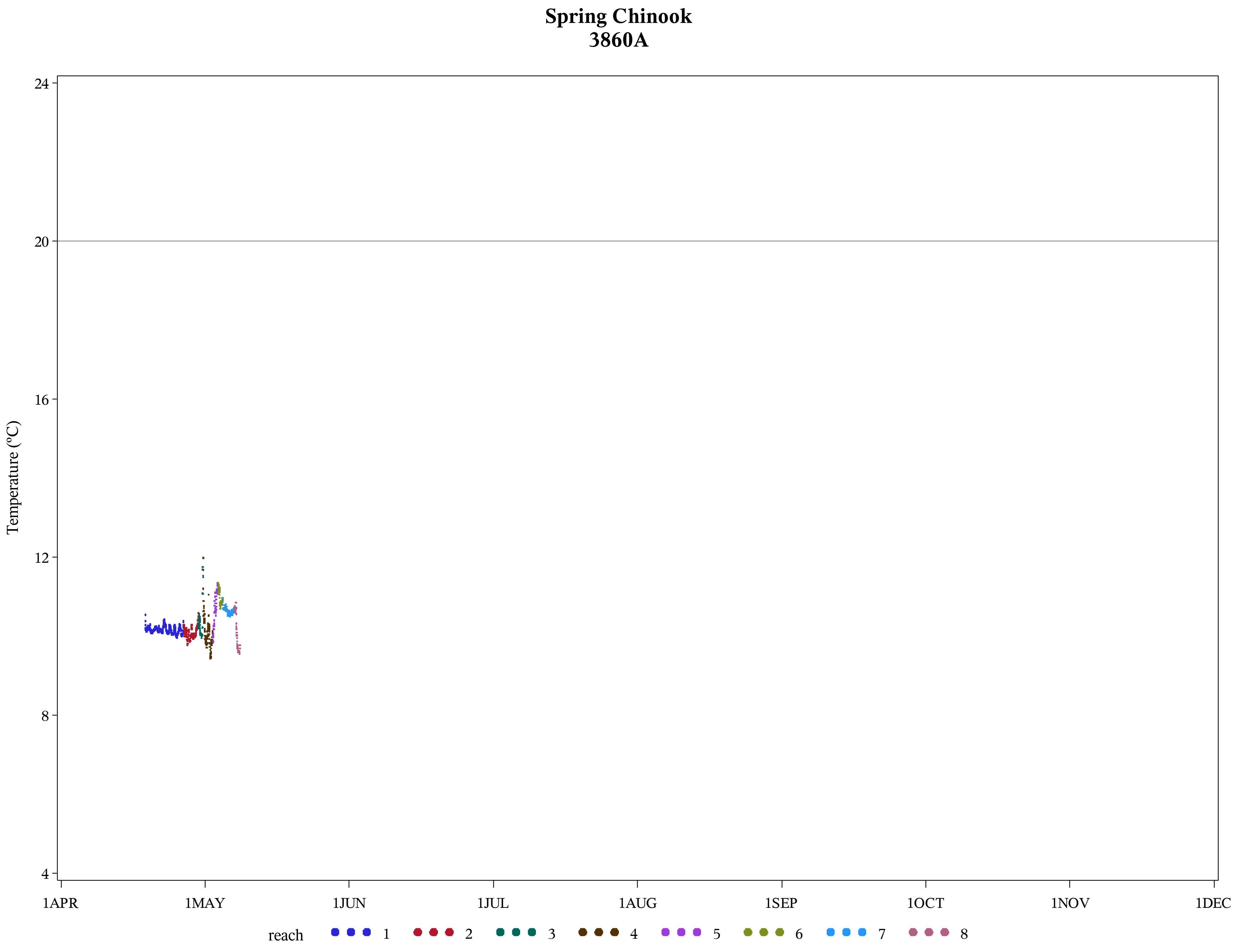
Spring Chinook
3860A
Temperature (°C)
24
20
16
12
8
4
1APR
1MAY
1JUN
1JUL
1AUG
1SEP
1OCT
1NOV
1DEC
reach
1
2
3
4
5
6
7
8

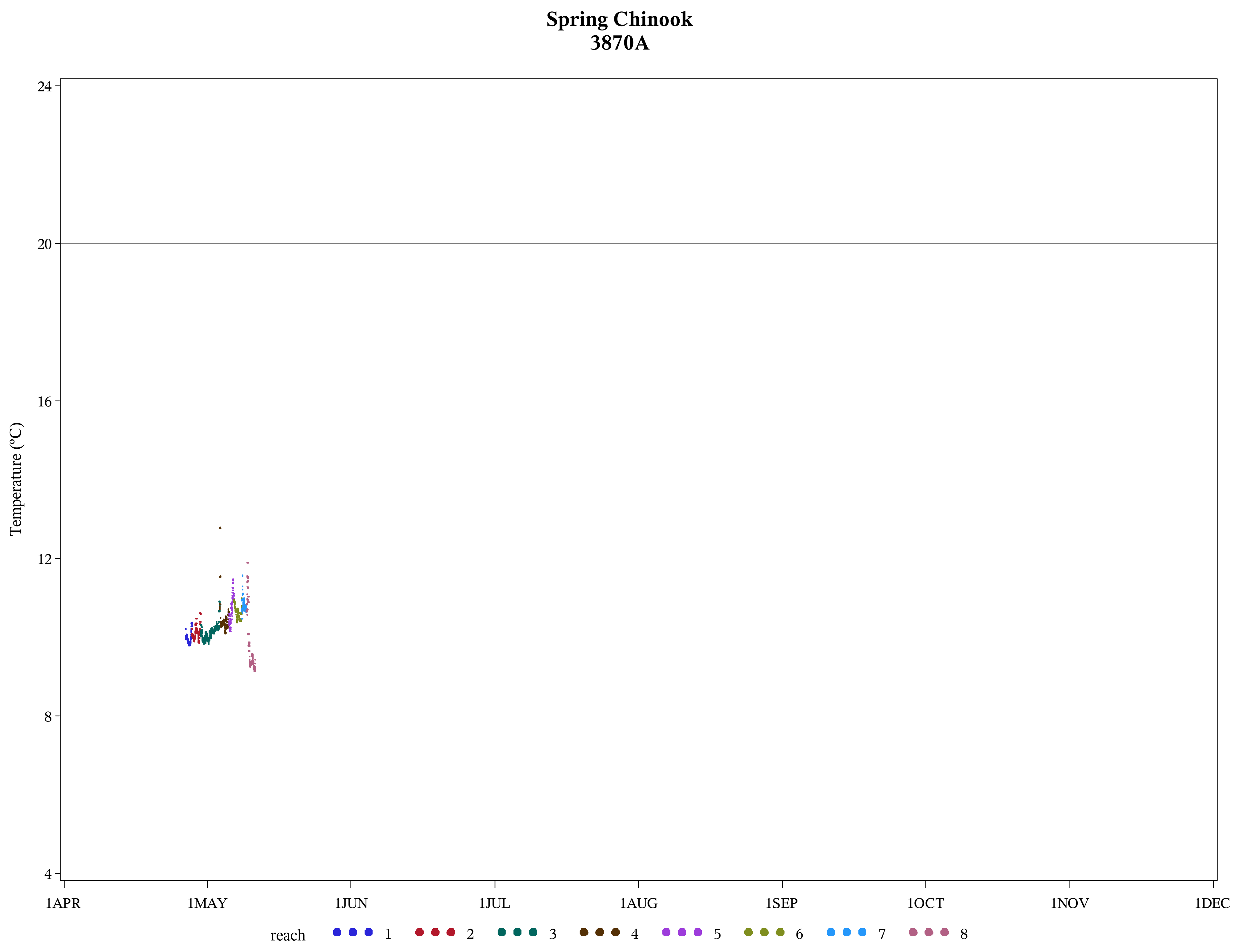
Spring Chinook
3870A
Temperature (°C)
24
20
16
12
8
4
1APR
1MAY
1JUN
1JUL
1AUG
1SEP
1OCT
1NOV
1DEC
reach
1
2
3
4
5
6
7
8

Spring Chinook
4734A

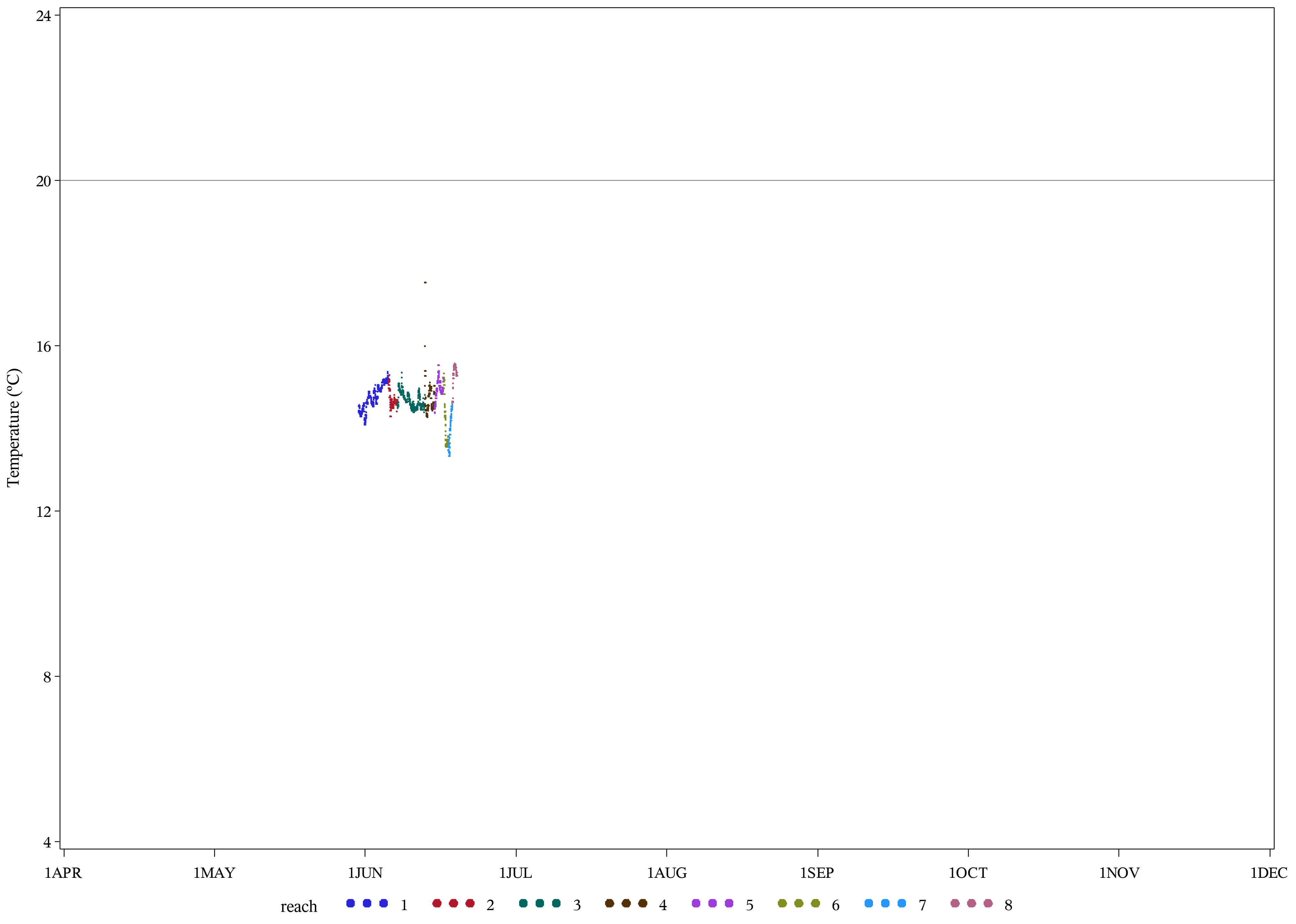

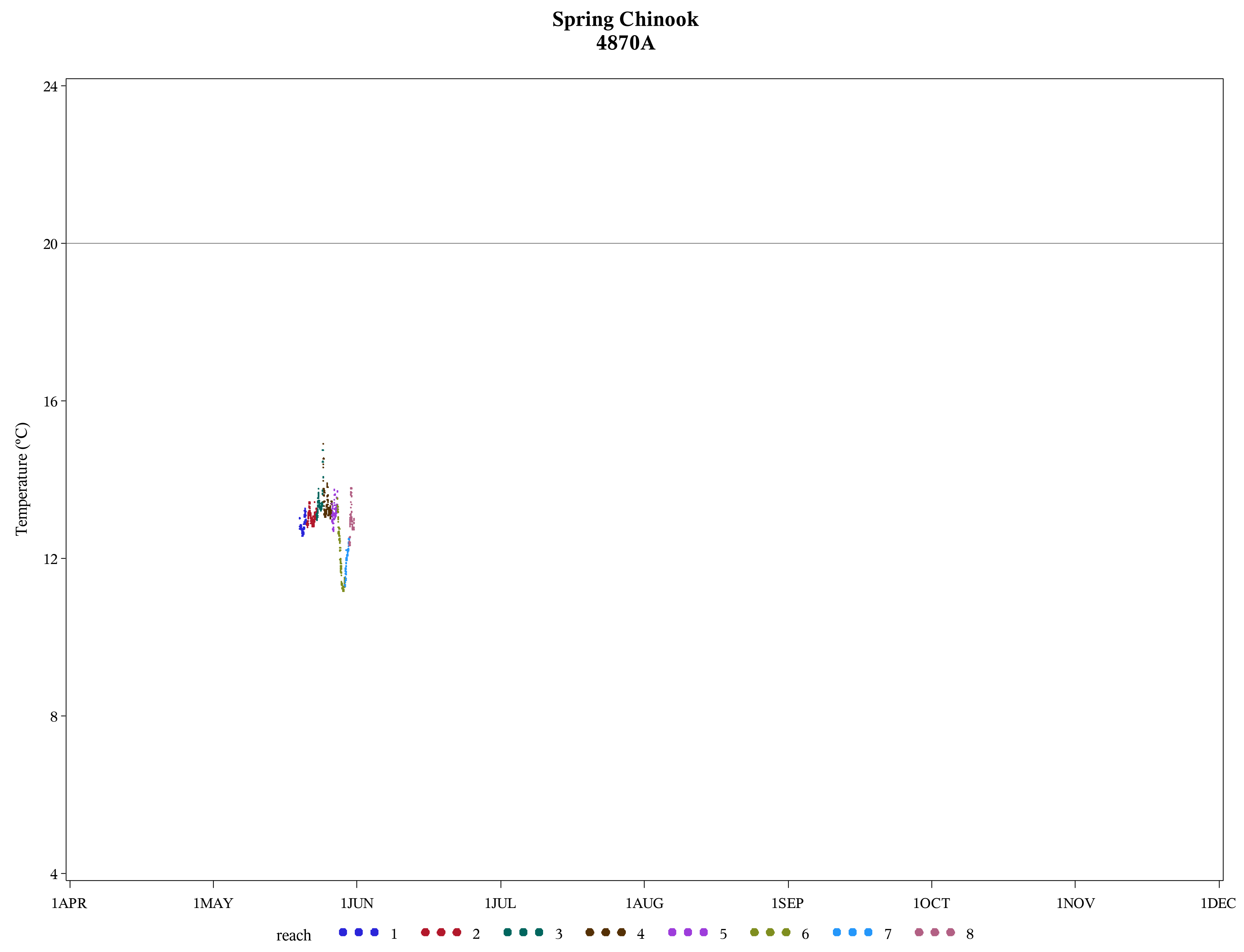
Spring Chinook
4870A
Temperature (°C)
24
20
16
12
8
4
1APR
1MAY
1JUN
1JUL
1AUG
1SEP
1OCT
1NOV
1DEC
reach
1
2
3
4
5
6
7
8

Spring Chinook
4877A

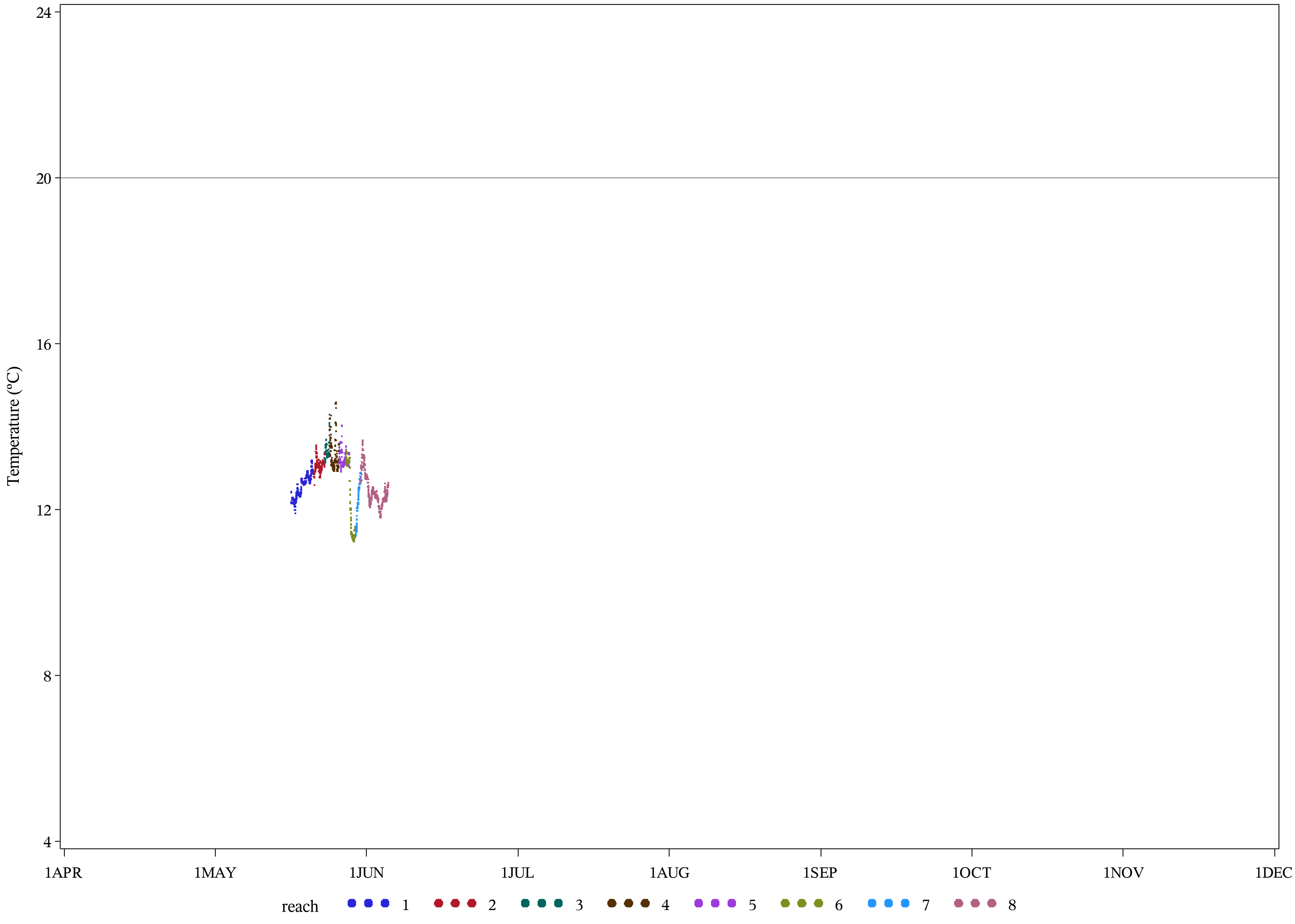

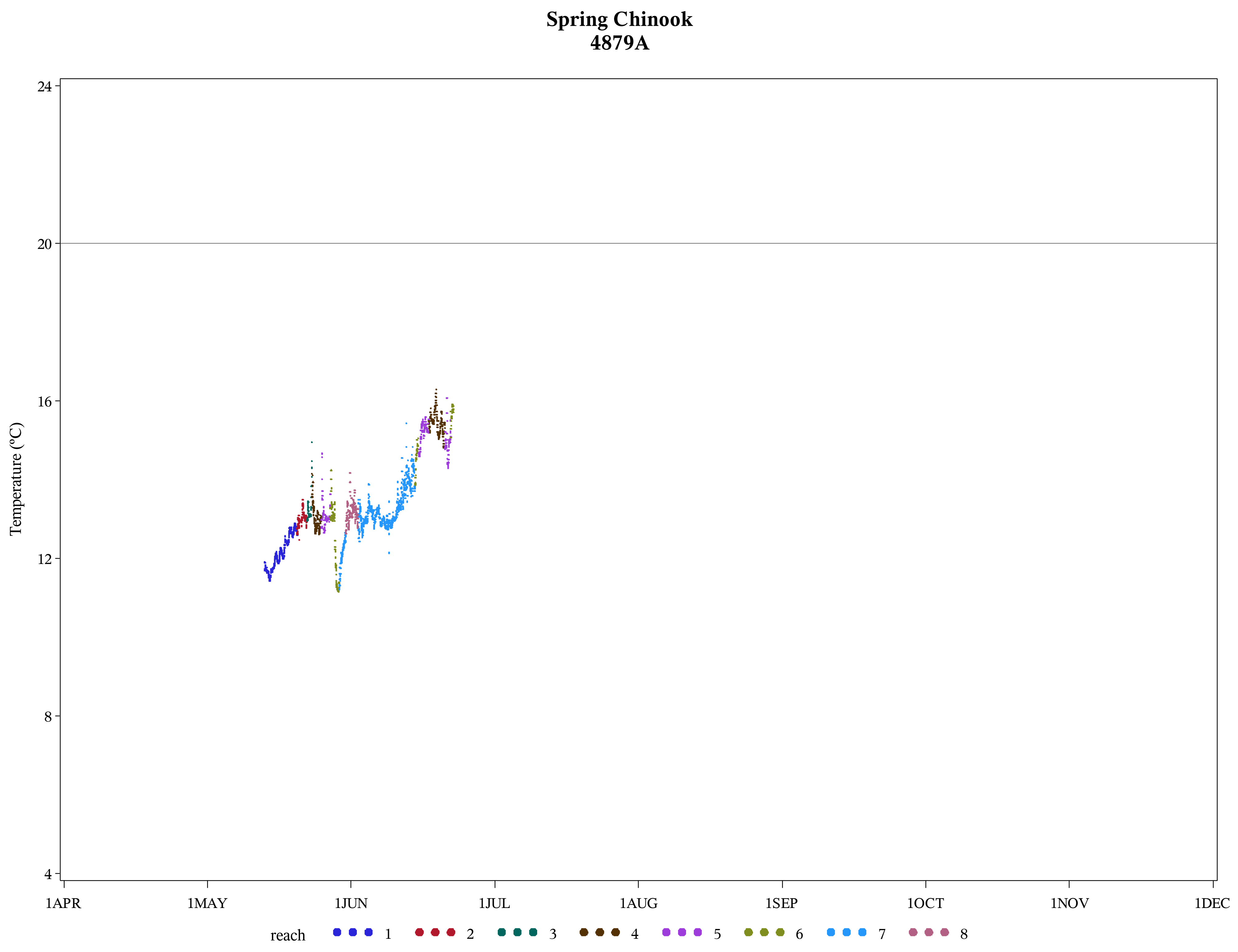

Spring Chinook
4879A
Temperature (°C)
24
20
16
12
8
4
1APR
1MAY
1JUN
1JUL
1AUG
1SEP
1OCT
1NOV
1DEC
reach
1
2
3
4
5
6
7
8

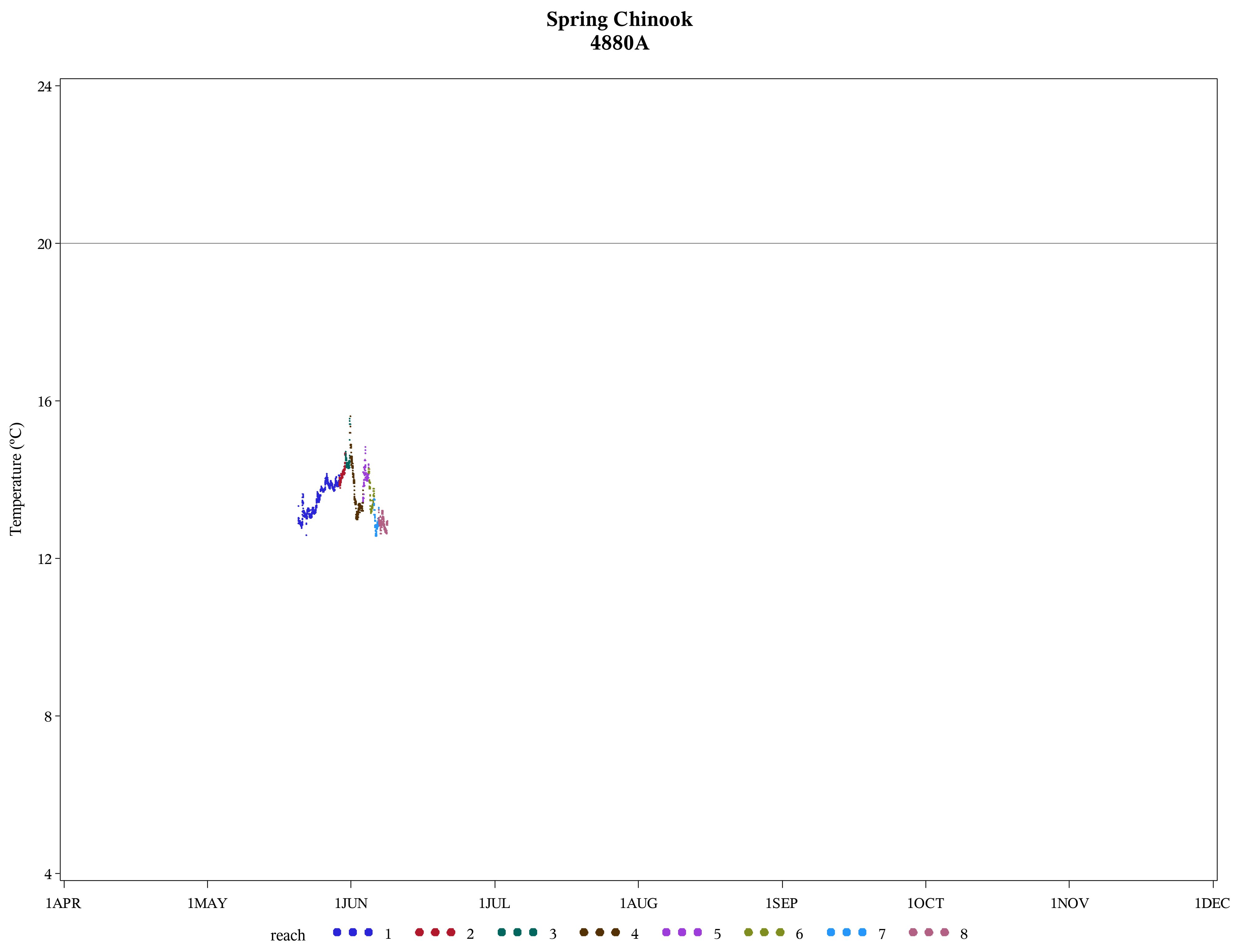

Spring Chinook
4880A
Temperature (°C)
24
20
16
12
8
4
1APR
1MAY
1JUN
1JUL
1AUG
1SEP
1OCT
1NOV
1DEC
reach
1
2
3
4
5
6
7
8

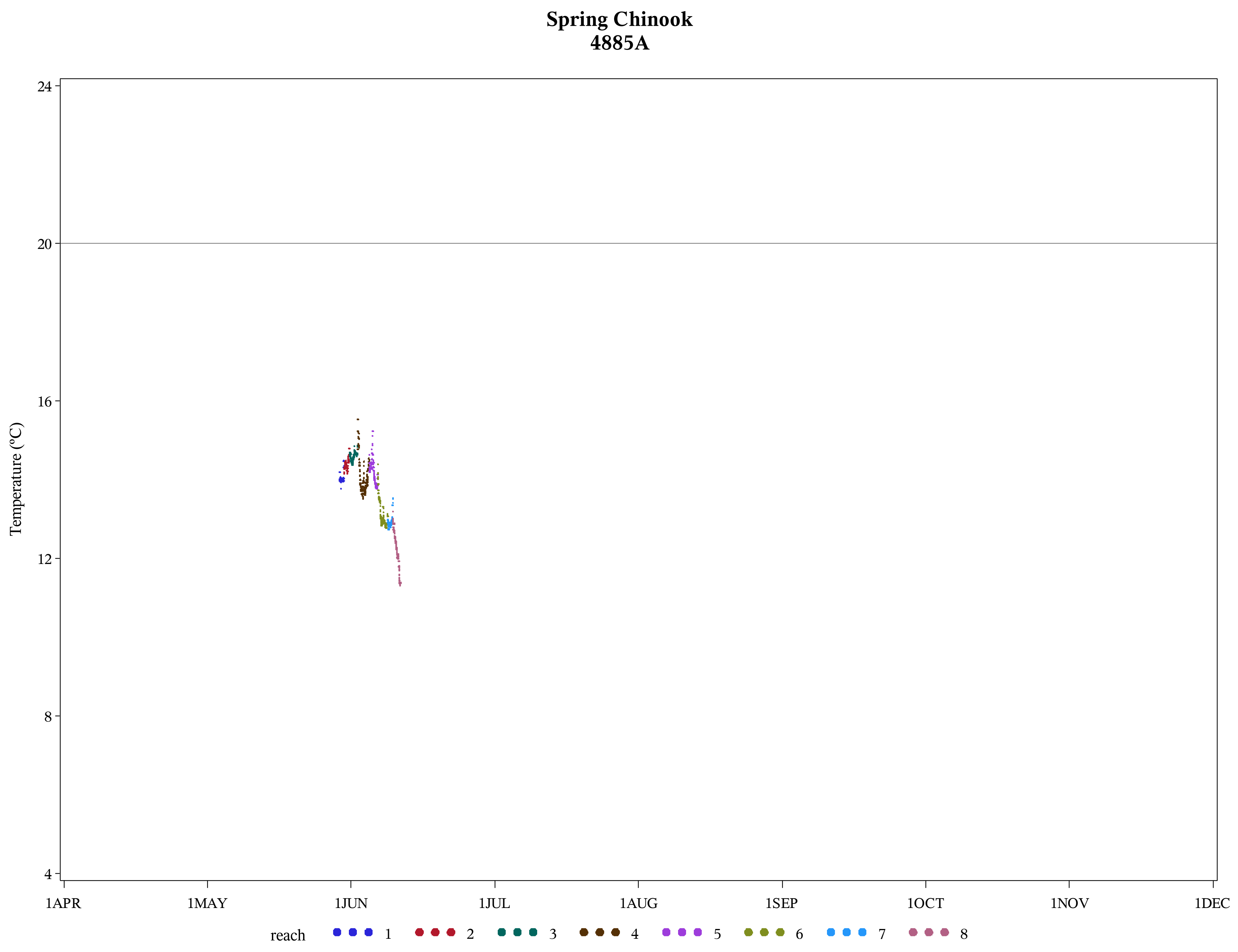
Spring Chinook
4885A
Temperature (°C)
24
20
16
12
8
4
1APR
1MAY
1JUN
1JUL
1AUG
1SEP
1OCT
1NOV
1DEC
reach
1
2
3
4
5
6
7
8

Spring Chinook
4886A

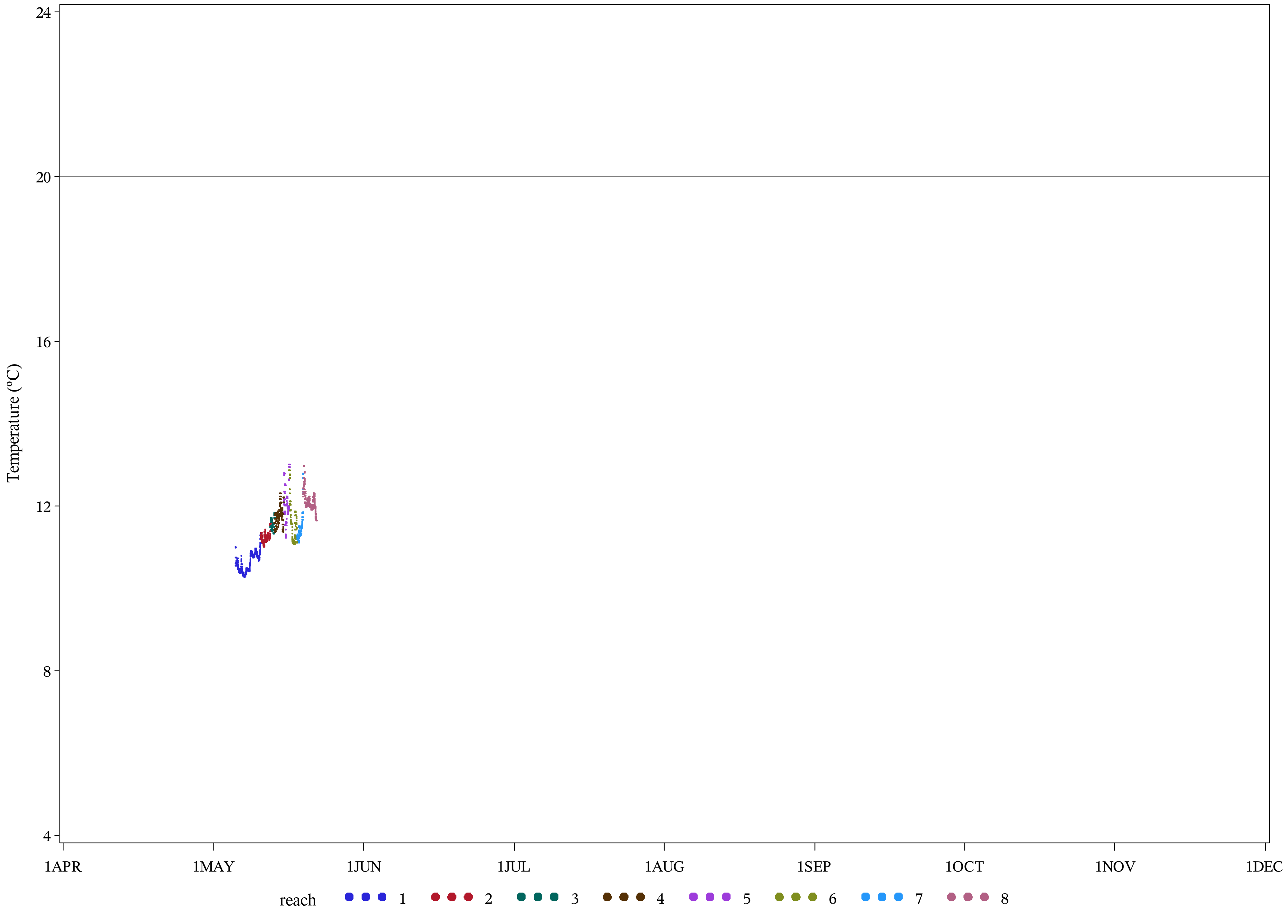

Spring Chinook
4887A

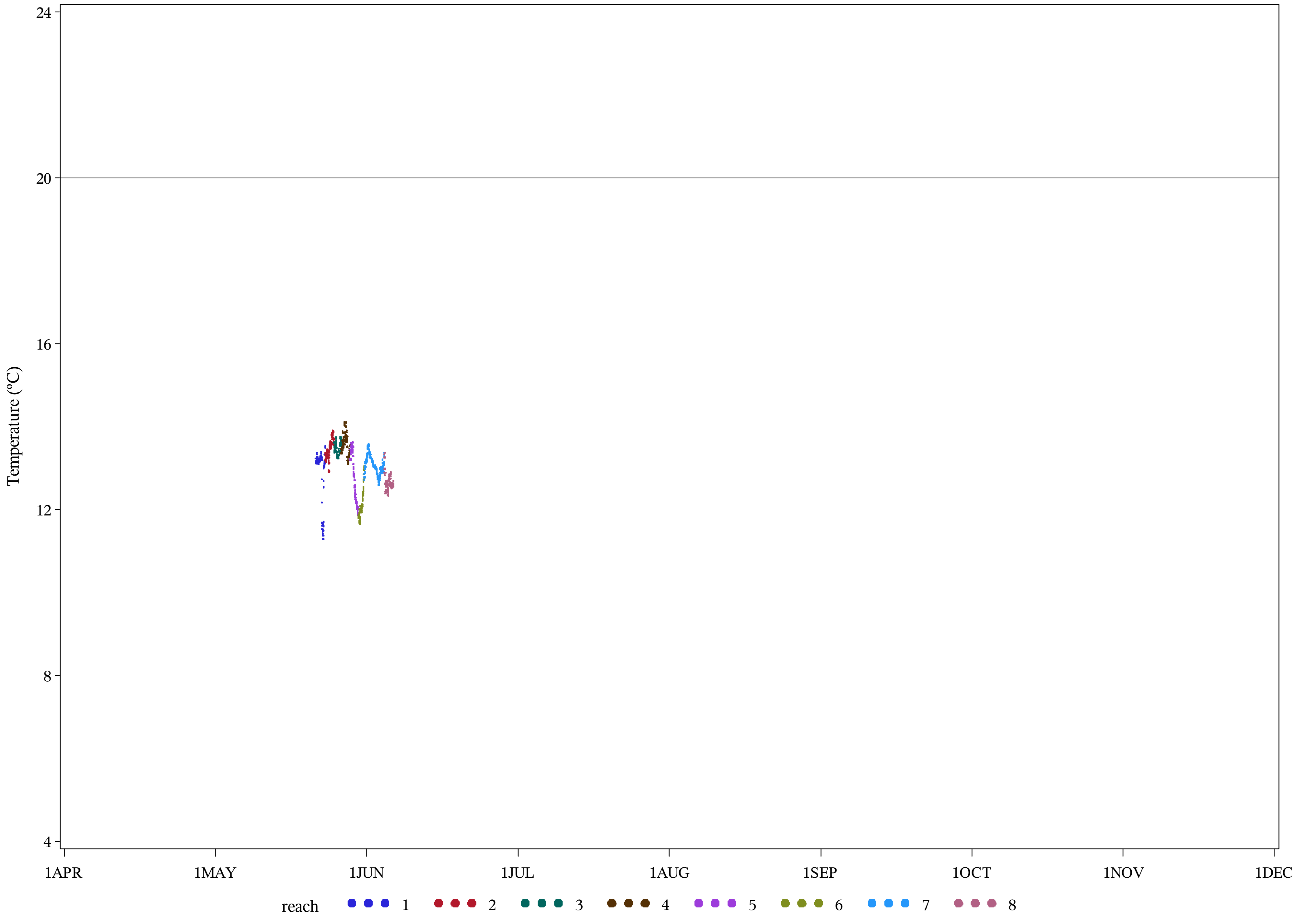

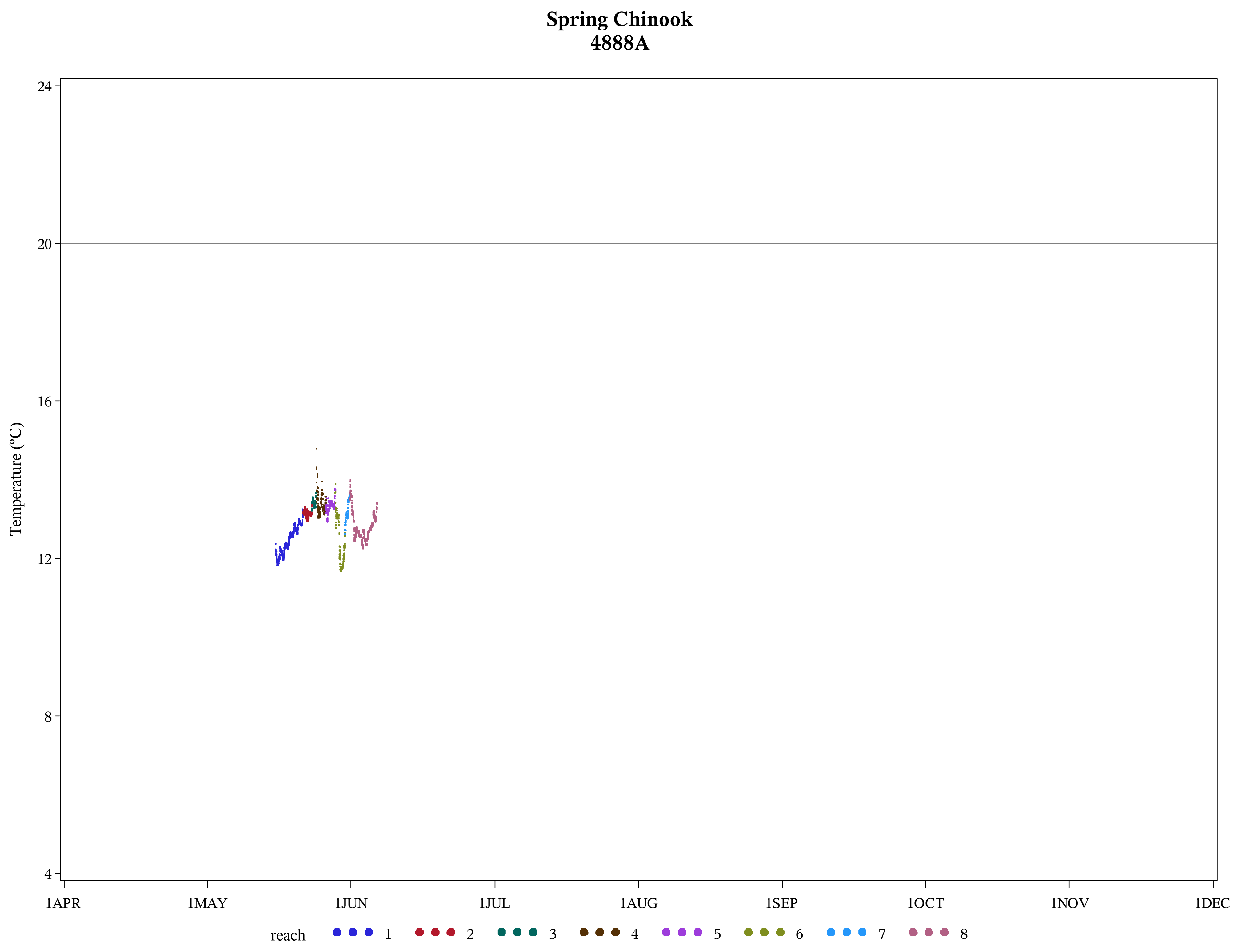

Spring Chinook
4888A
Temperature (°C)
24
20
16
12
8
4
1APR
1MAY
1JUN
1JUL
1AUG
1SEP
1OCT
1NOV
1DEC
reach
1
2
3
4
5
6
7
8

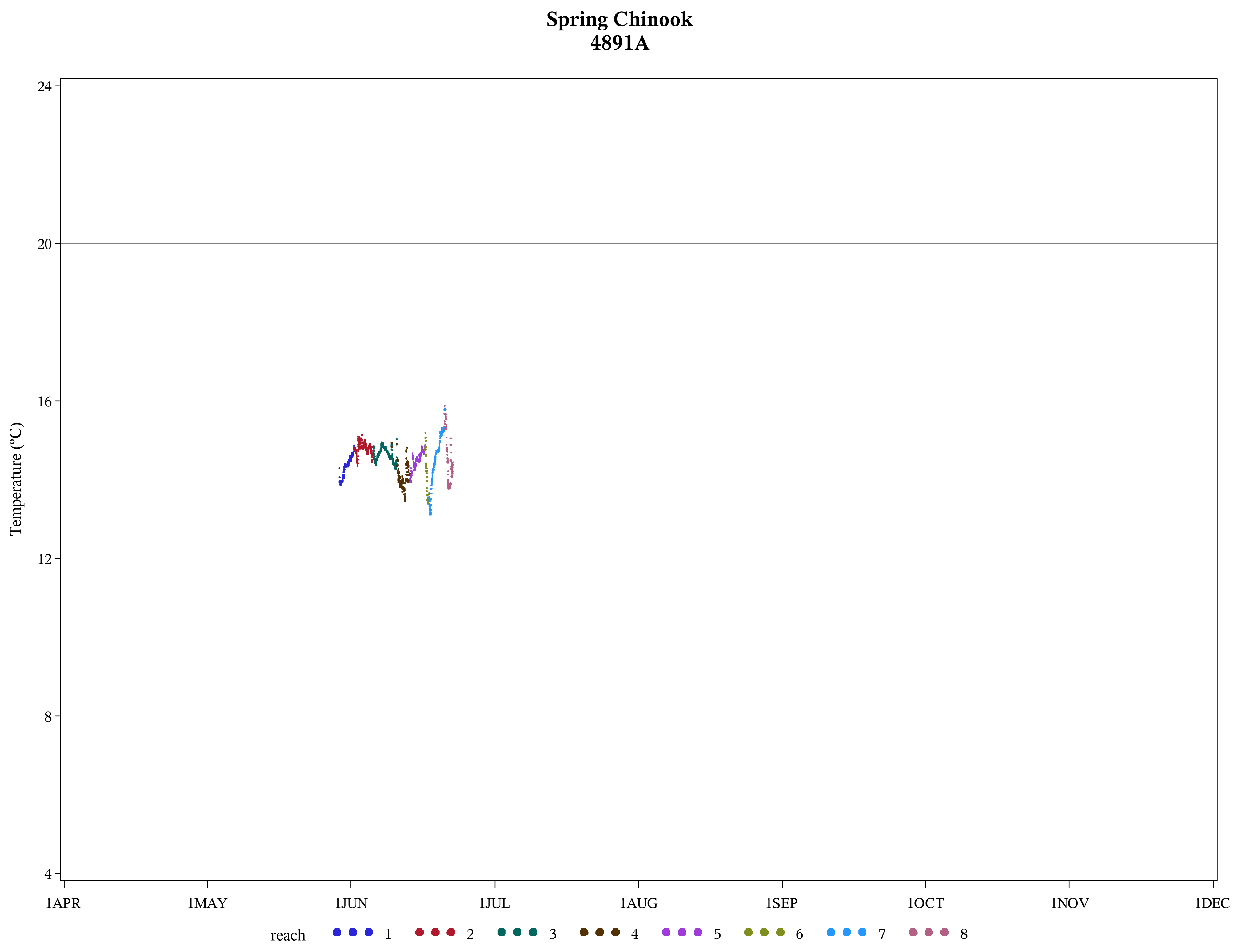
Spring Chinook
4891A
Temperature (°C)
24
20
16
12
8
4
1APR
1MAY
1JUN
1JUL
1AUG
1SEP
1OCT
1NOV
1DEC
reach
1
2
3
4
5
6
7
8

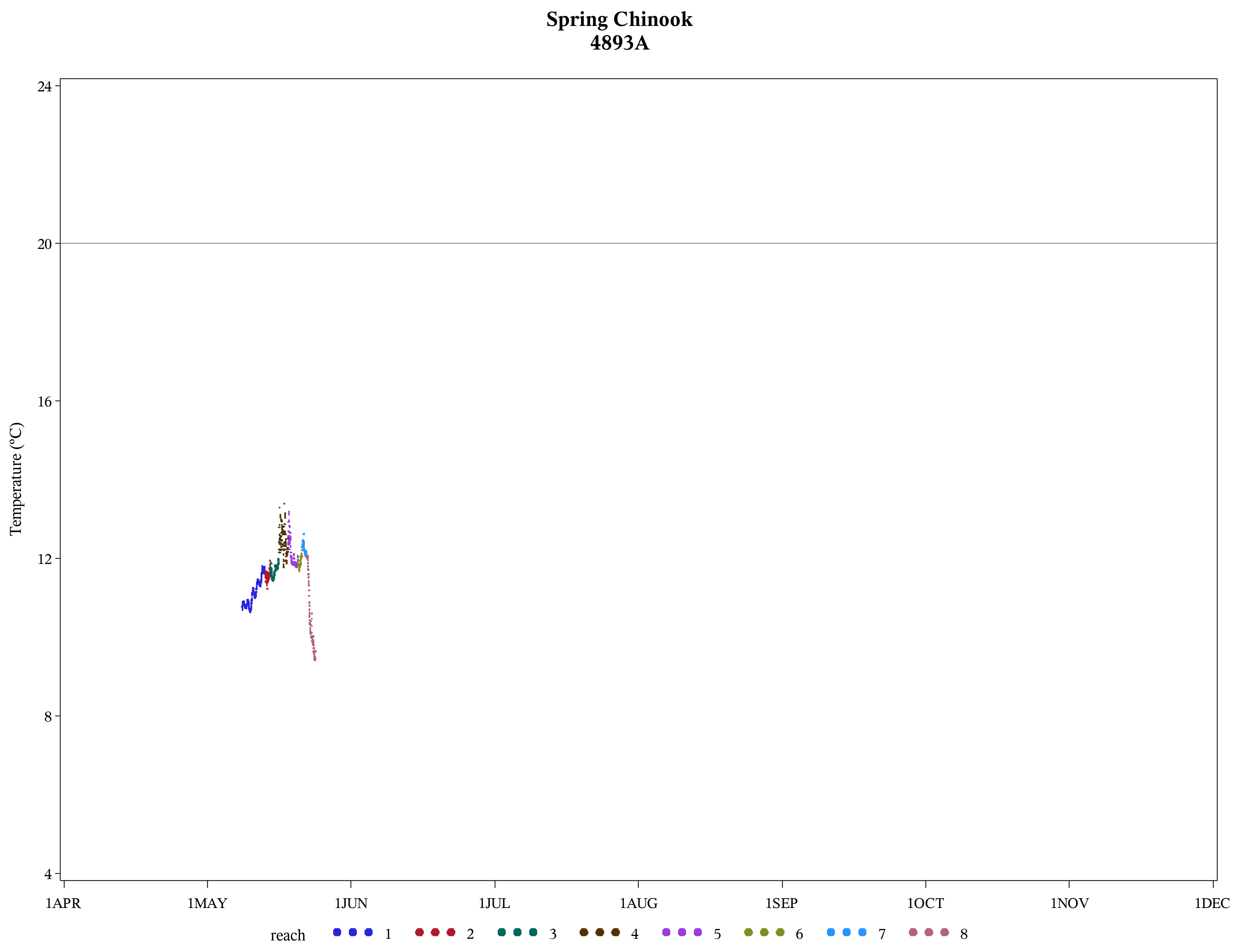

Spring Chinook
4893A
Temperature (°C)
24
20
16
12
8
4
1APR
1MAY
1JUN
1JUL
1AUG
1SEP
1OCT
1NOV
1DEC
reach
1
2
3
4
5
6
7
8

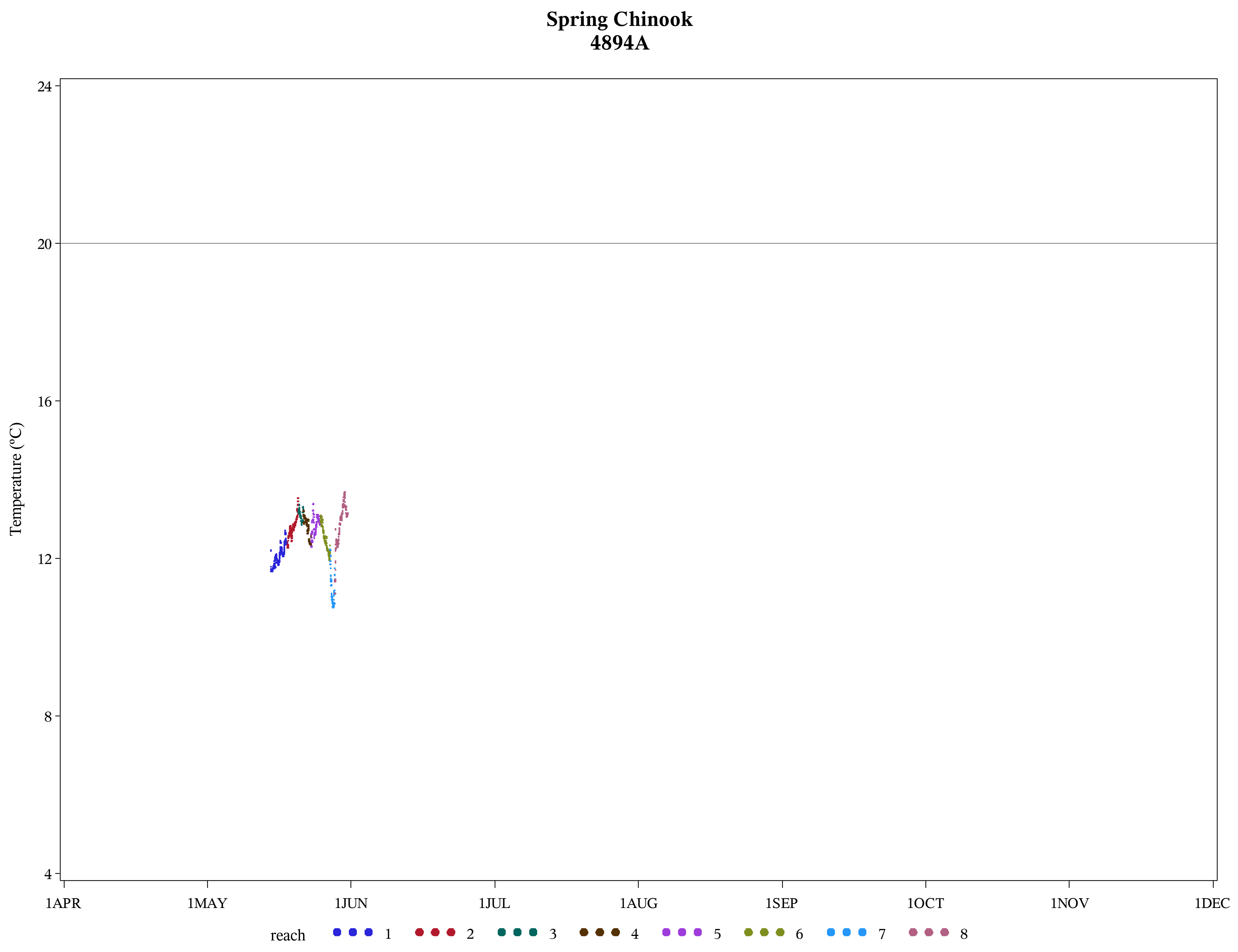
Spring Chinook
4894A
Temperature (°C)
24
20
16
12
8
4
1APR
1MAY
1JUN
1JUL
1AUG
1SEP
1OCT
1NOV
1DEC
reach
1
2
3
4
5
6
7
8

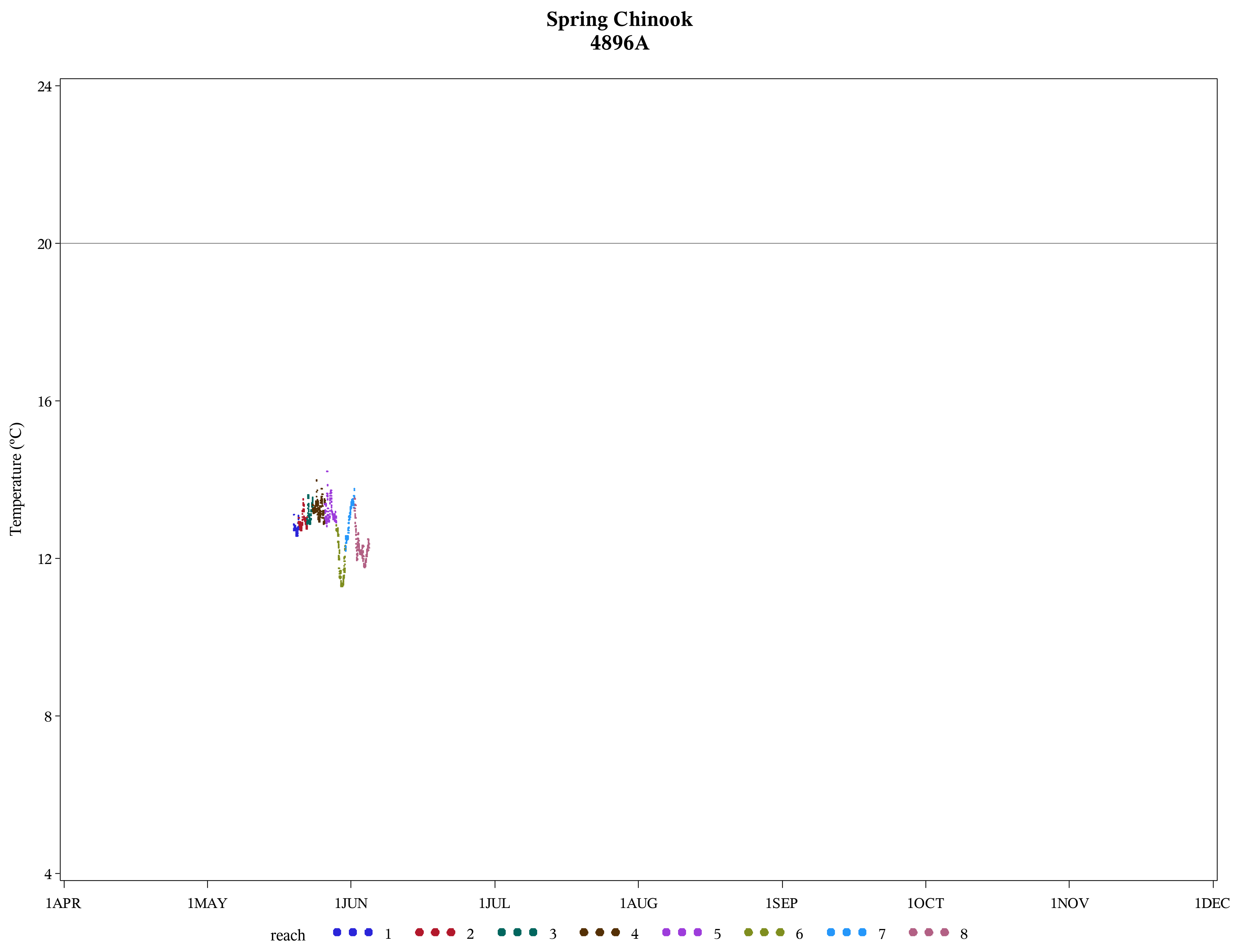
Spring Chinook
4896A
Temperature (°C)
24
20
16
12
8
4
1APR
1MAY
1JUN
1JUL
1AUG
1SEP
1OCT
1NOV
1DEC
reach
1
2
3
4
5
6
7
8

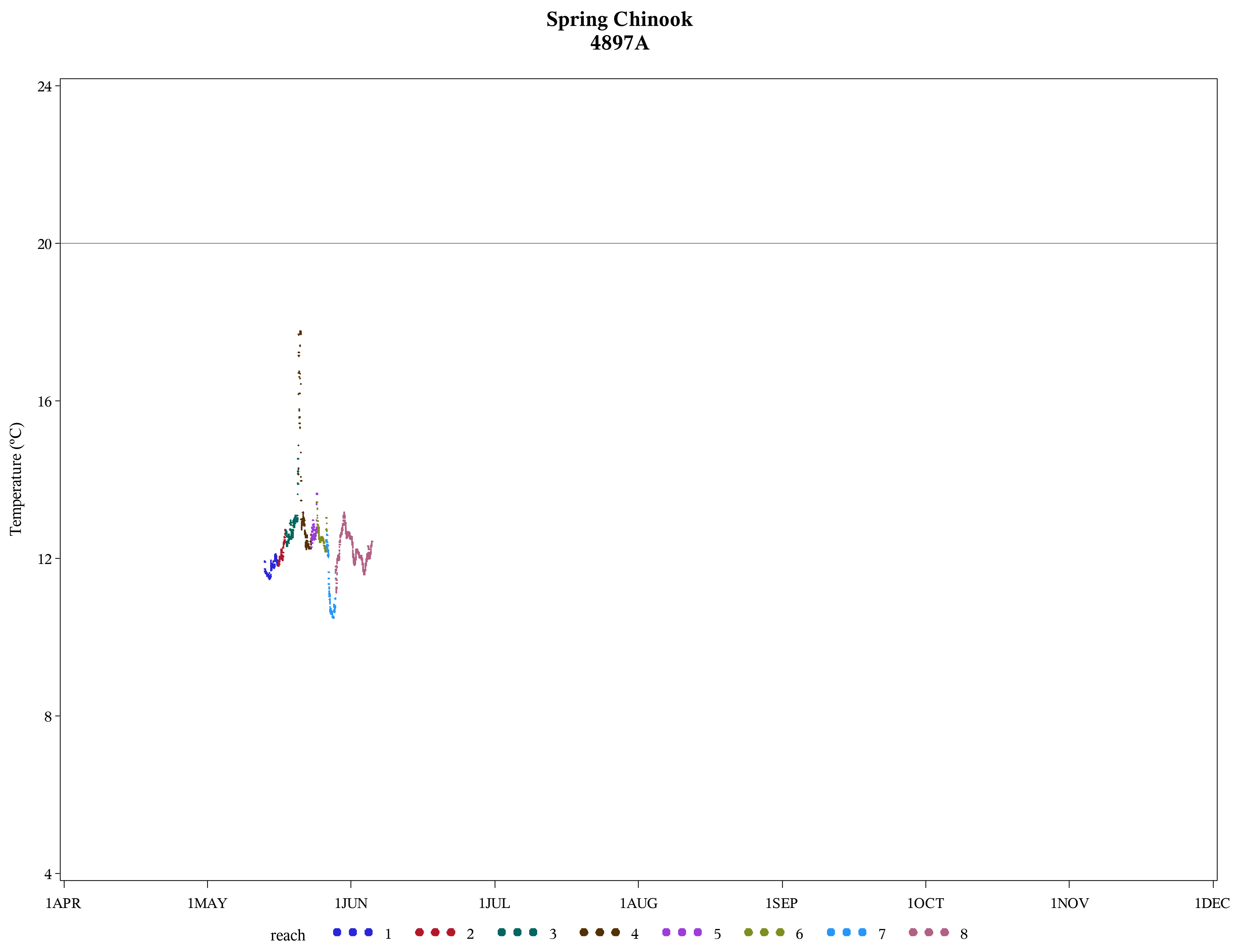

Spring Chinook
4897A
Temperature (°C)
24
20
16
12
8
4
1APR
1MAY
1JUN
1JUL
1AUG
1SEP
1OCT
1NOV
1DEC
reach
1
2
3
4
5
6
7
8

Spring Chinook
4900A

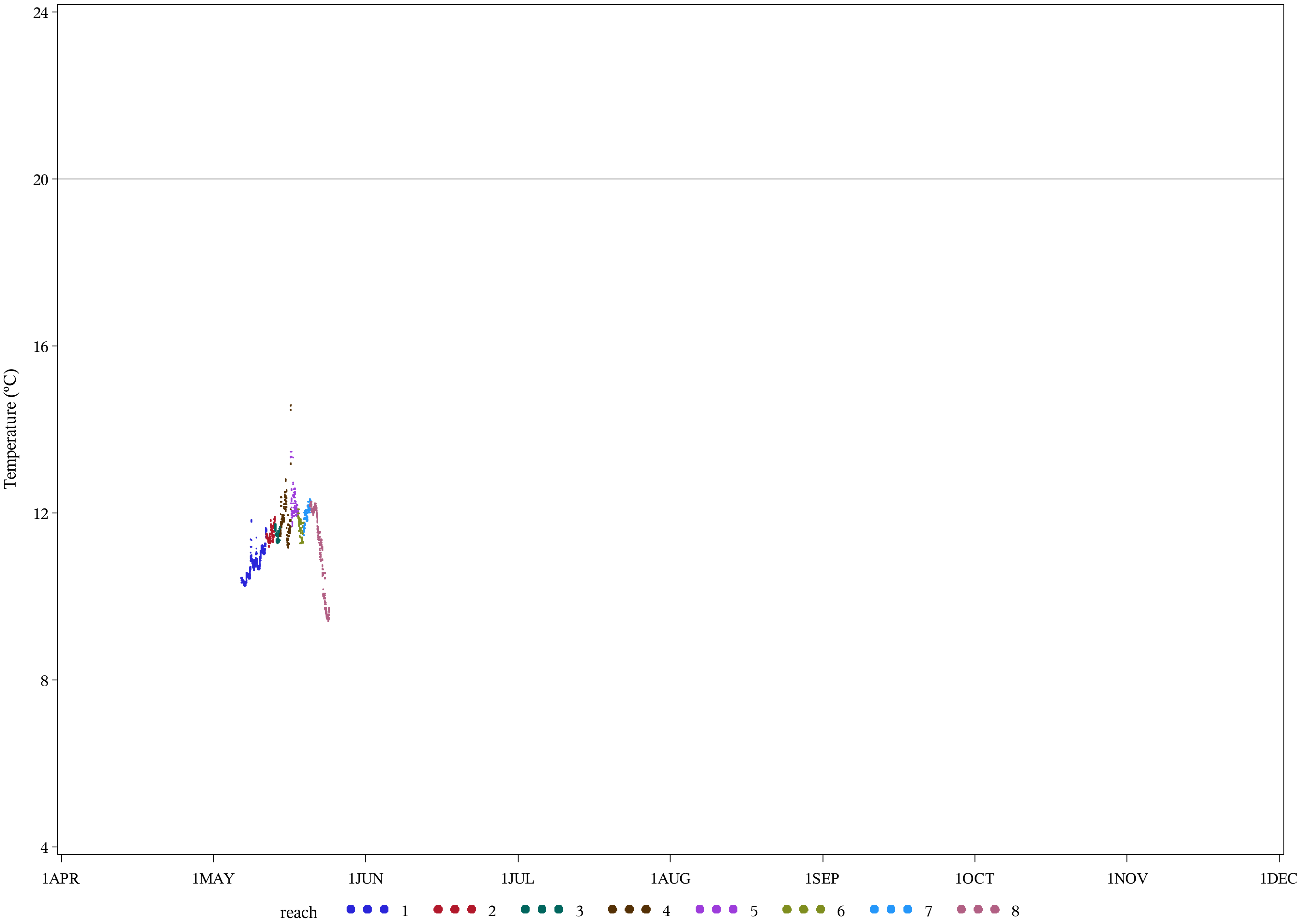

Spring Chinook
4903A

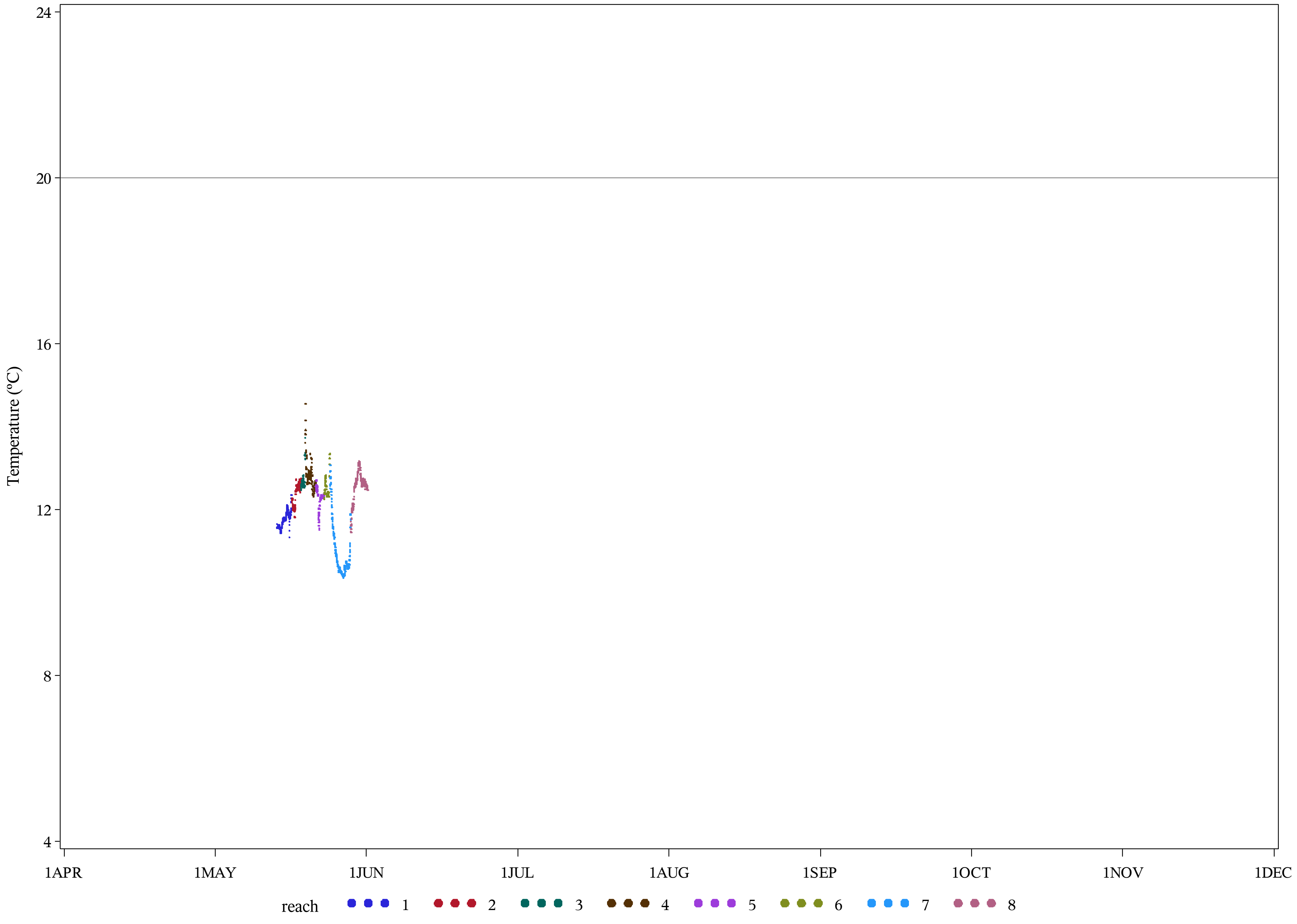

Spring Chinook
4906A

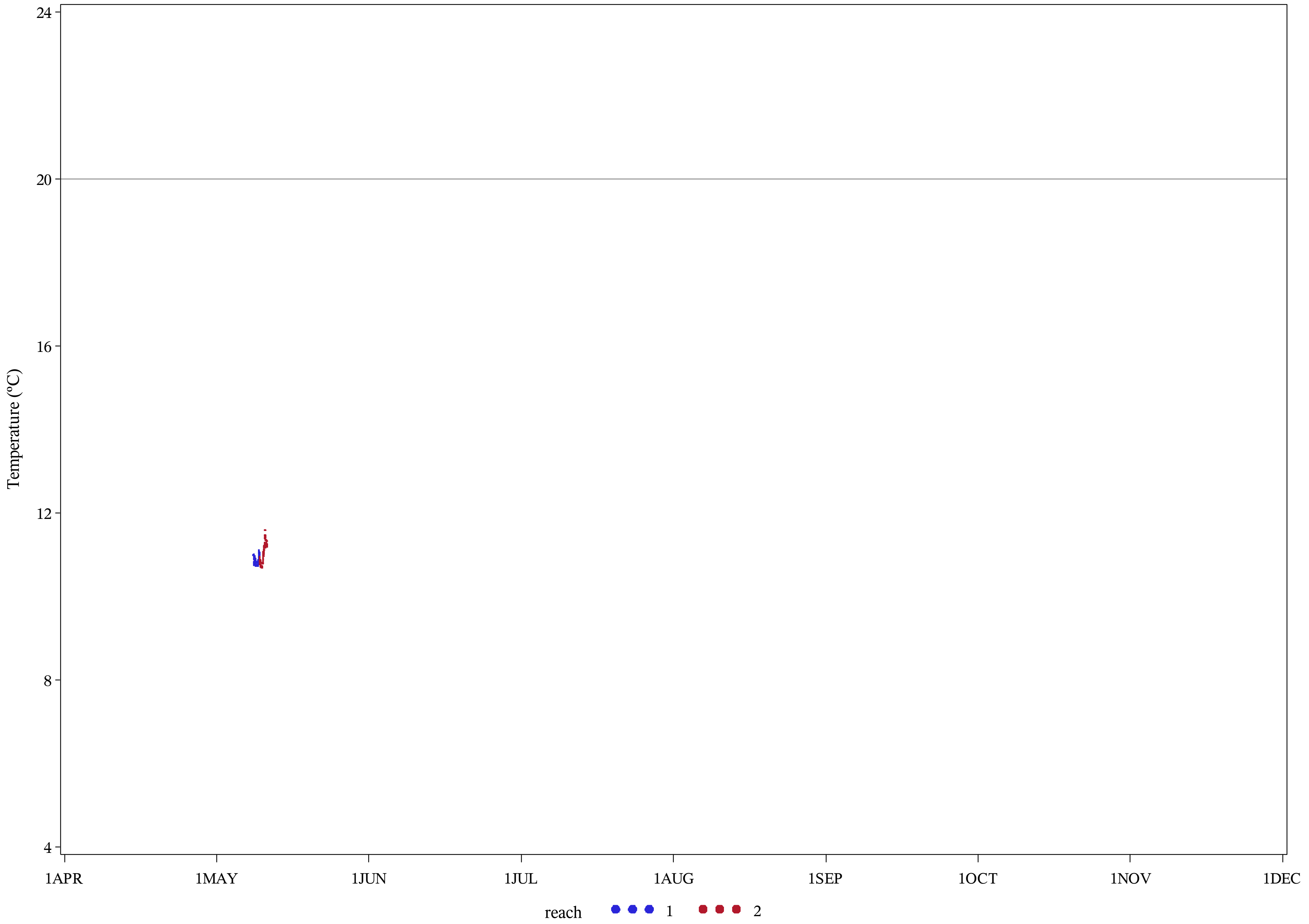

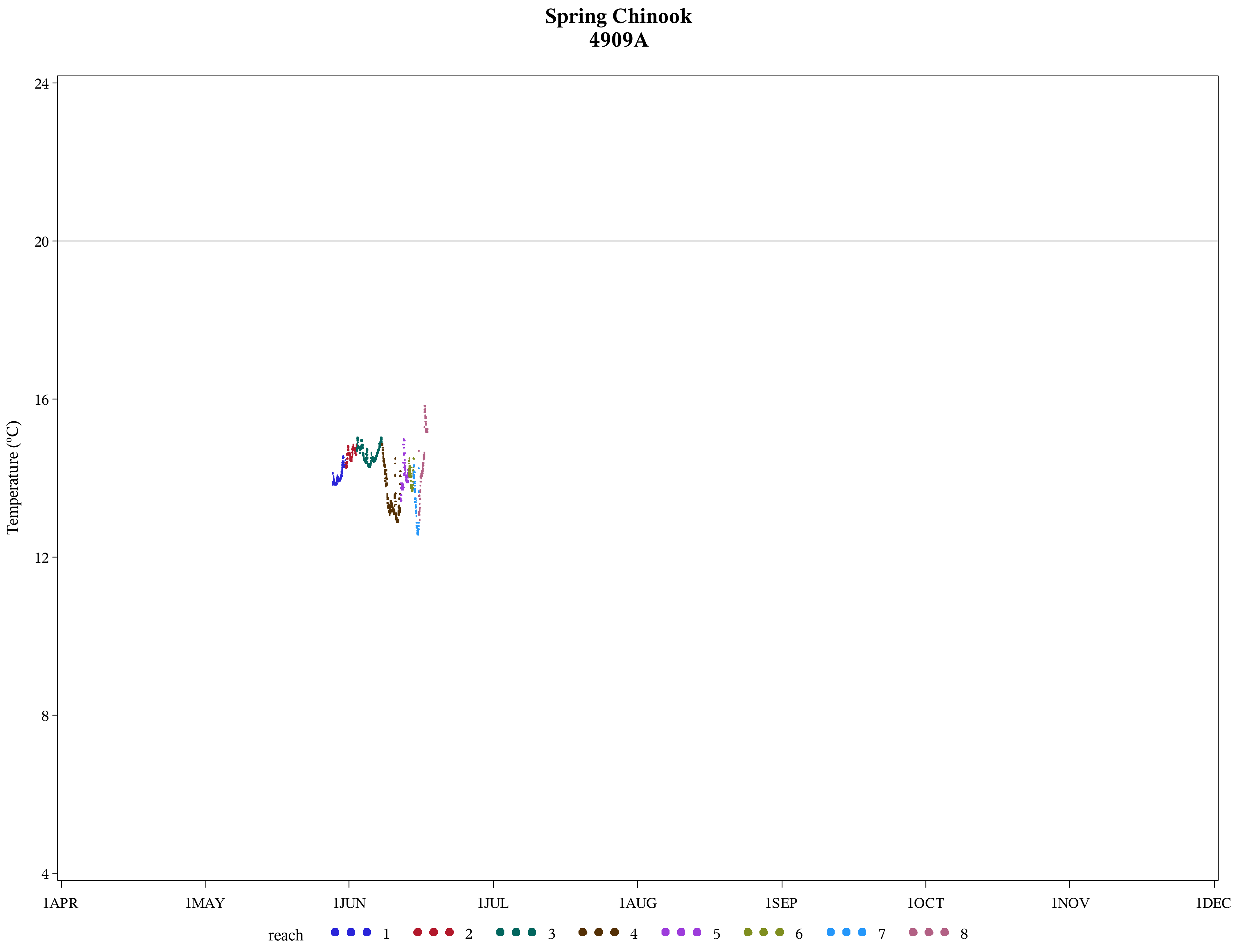

Spring Chinook
4909A
Temperature (°C)
24
20
16
12
8
4
1APR
1MAY
1JUN
1JUL
1AUG
1SEP
1OCT
1NOV
1DEC
reach
1
2
3
4
5
6
7
8

Spring Chinook
4912A

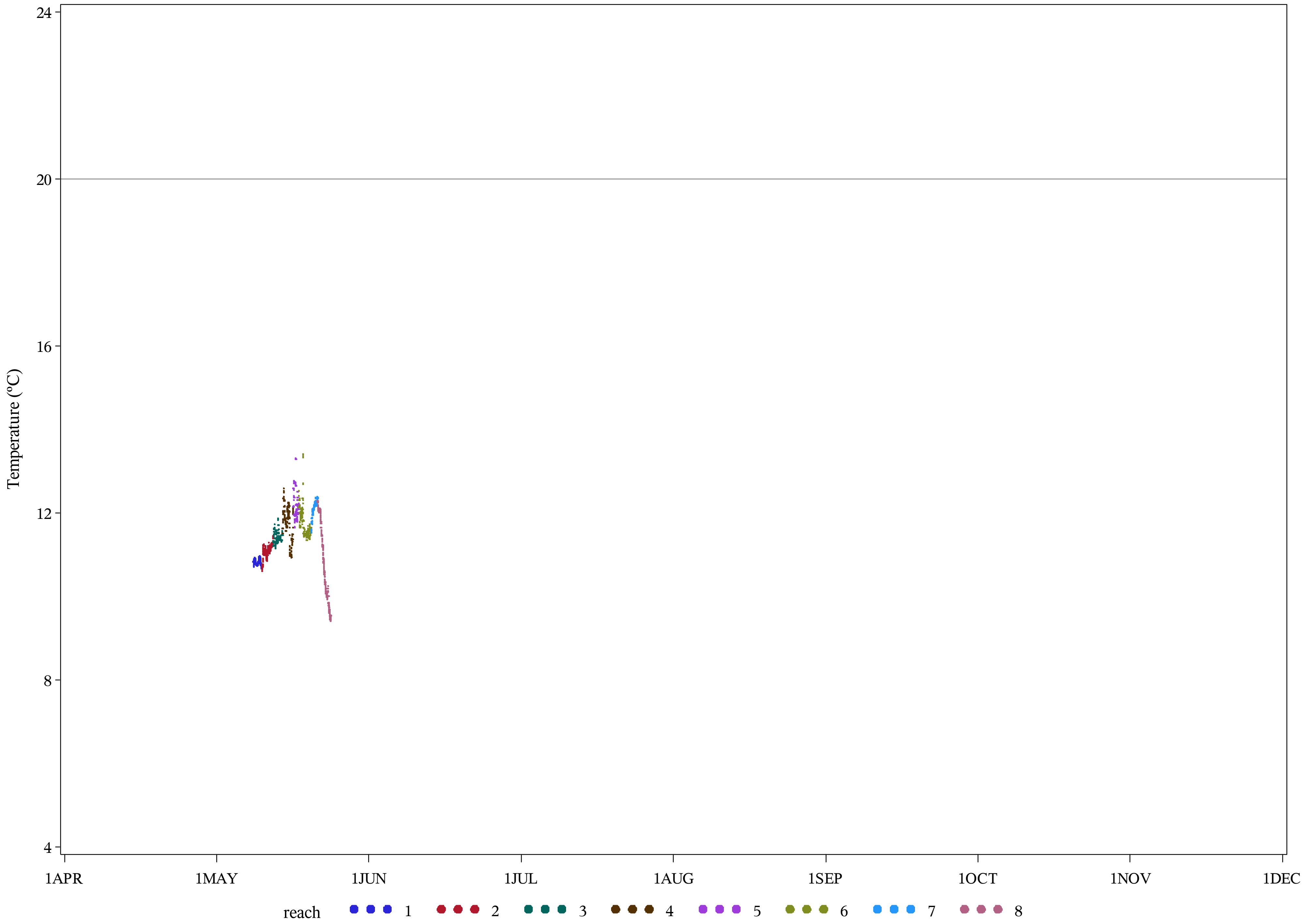

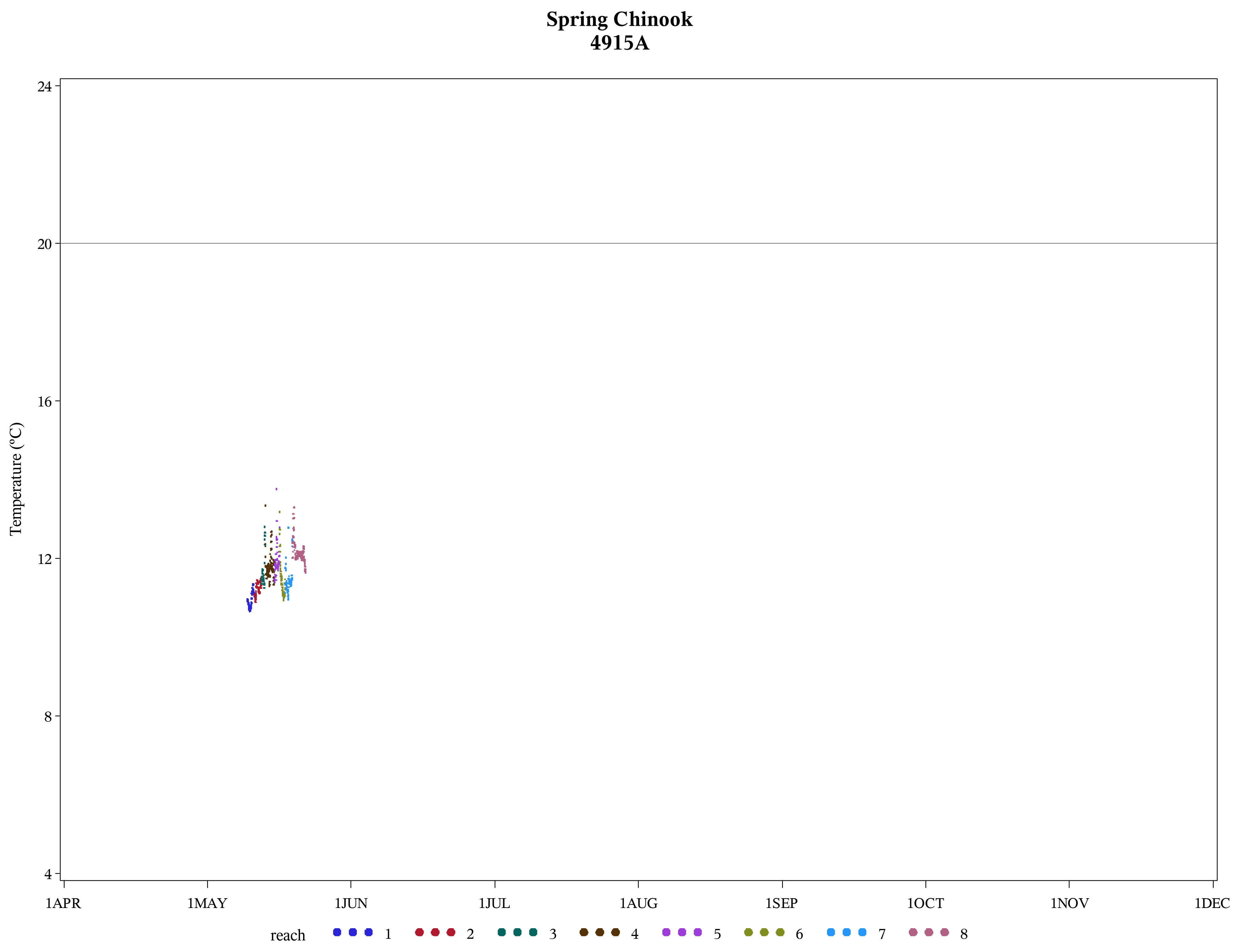

Spring Chinook
4915A
Temperature (°C)
24
20
16
12
8
4
1APR
1MAY
1JUN
1JUL
1AUG
1SEP
1OCT
1NOV
1DEC
reach
1
2
3
4
5
6
7
8

Spring Chinook
4916A

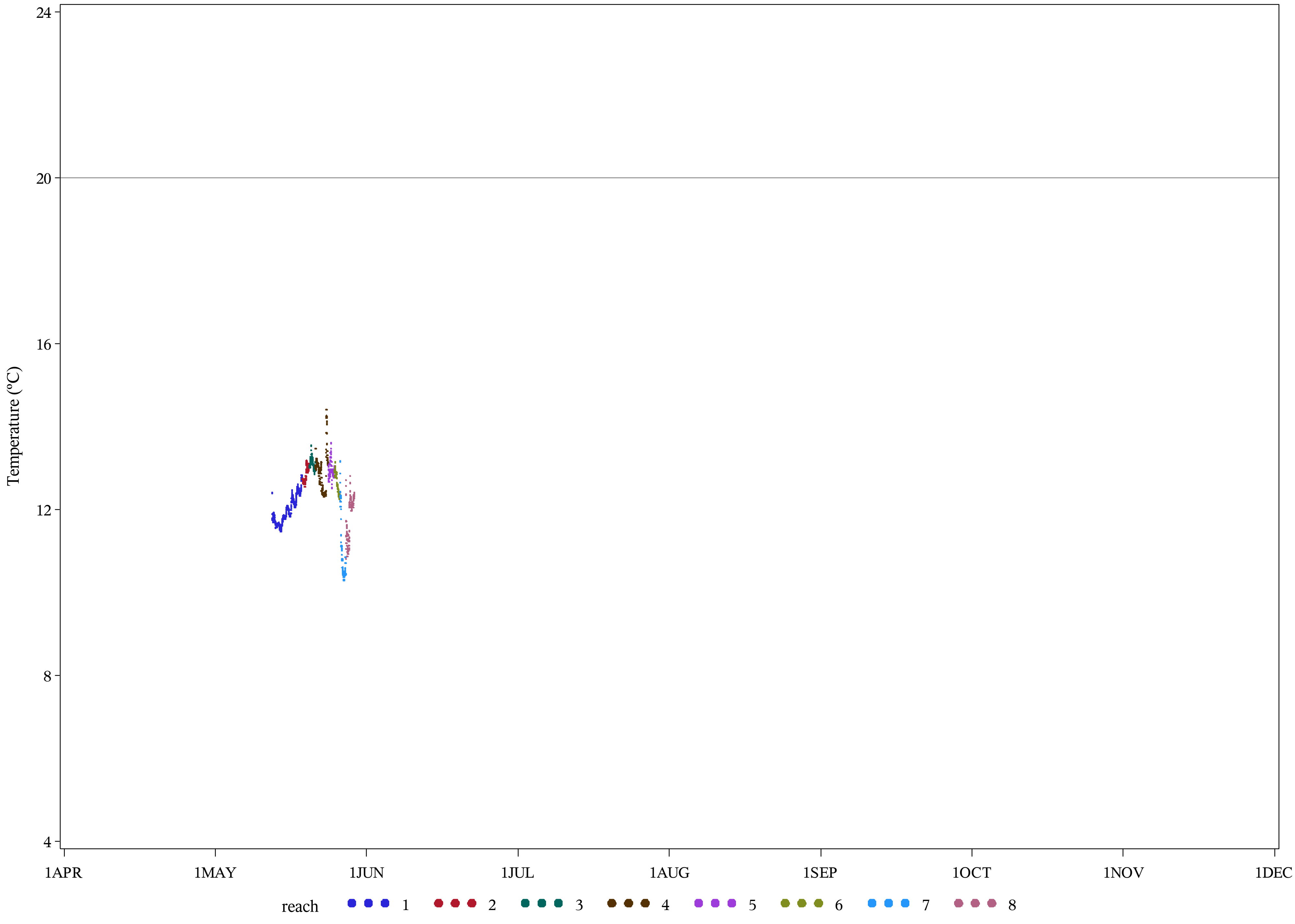

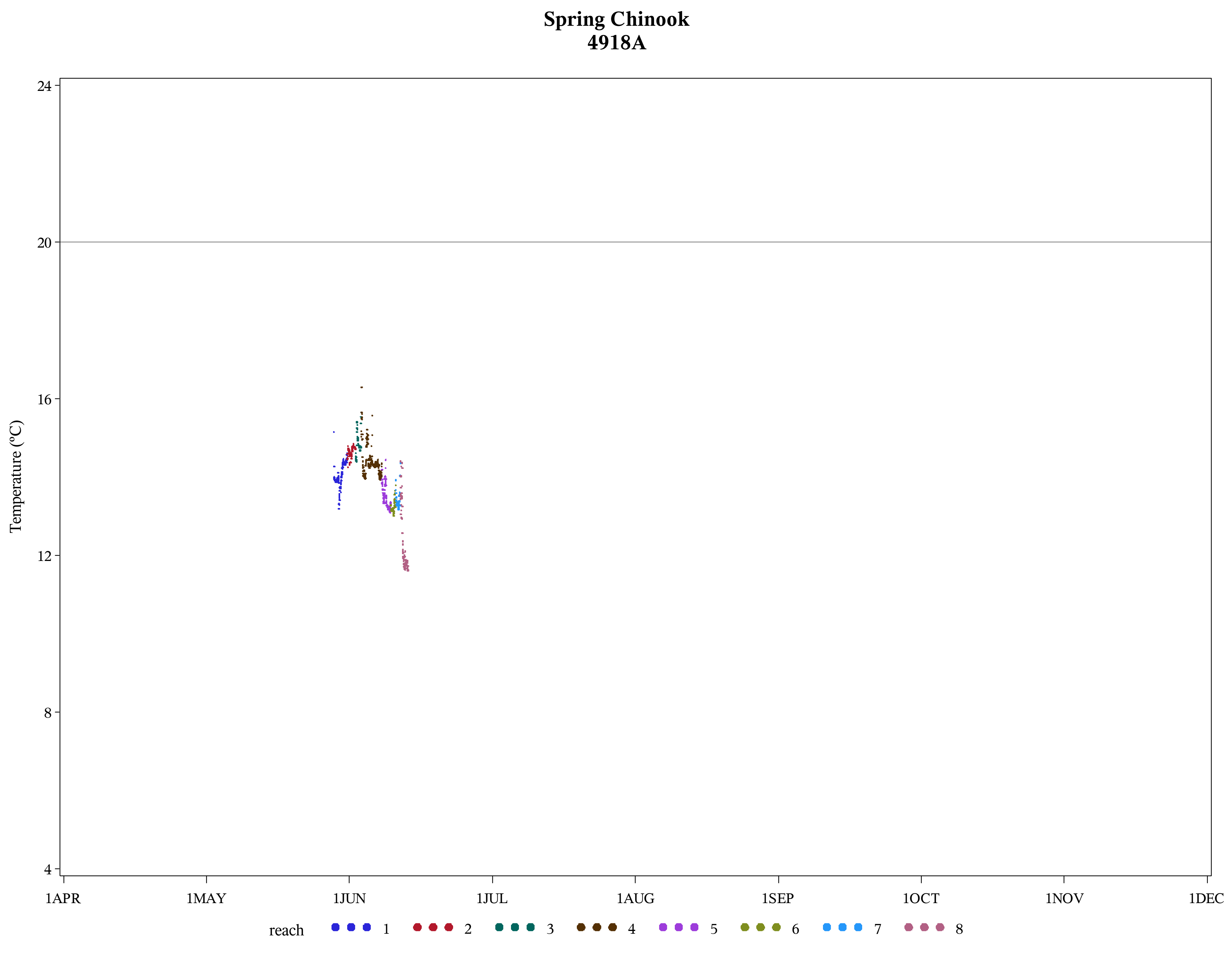
Spring Chinook
4918A
Temperature (°C)
24
20
16
12
8
4
1APR
1MAY
1JUN
1JUL
1AUG
1SEP
1OCT
1NOV
1DEC
reach
1
2
3
4
5
6
7
8

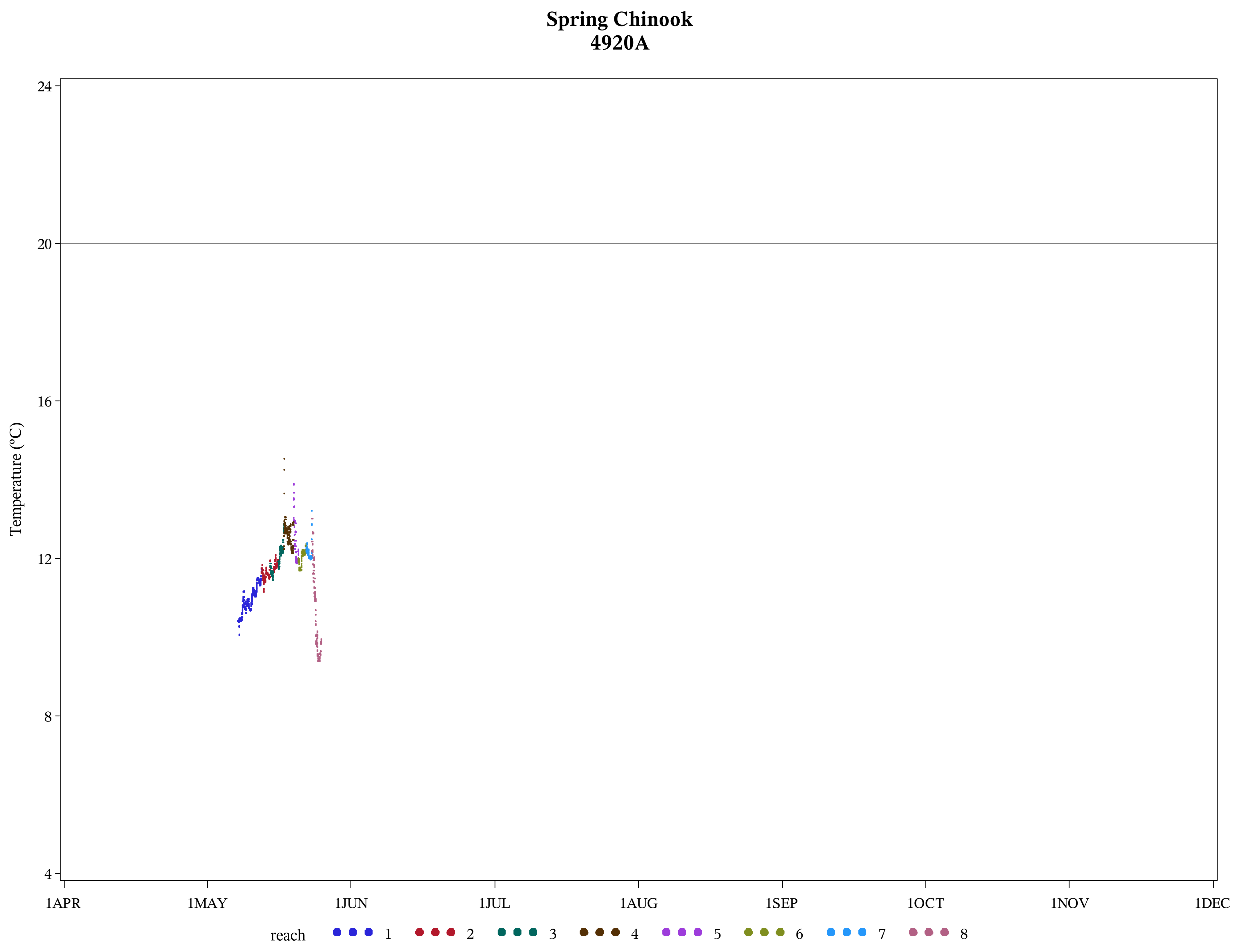

Spring Chinook
4920A
Temperature (°C)
24
20
16
12
8
4
1APR
1MAY
1JUN
1JUL
1AUG
1SEP
1OCT
1NOV
1DEC
reach
1
2
3
4
5
6
7
8

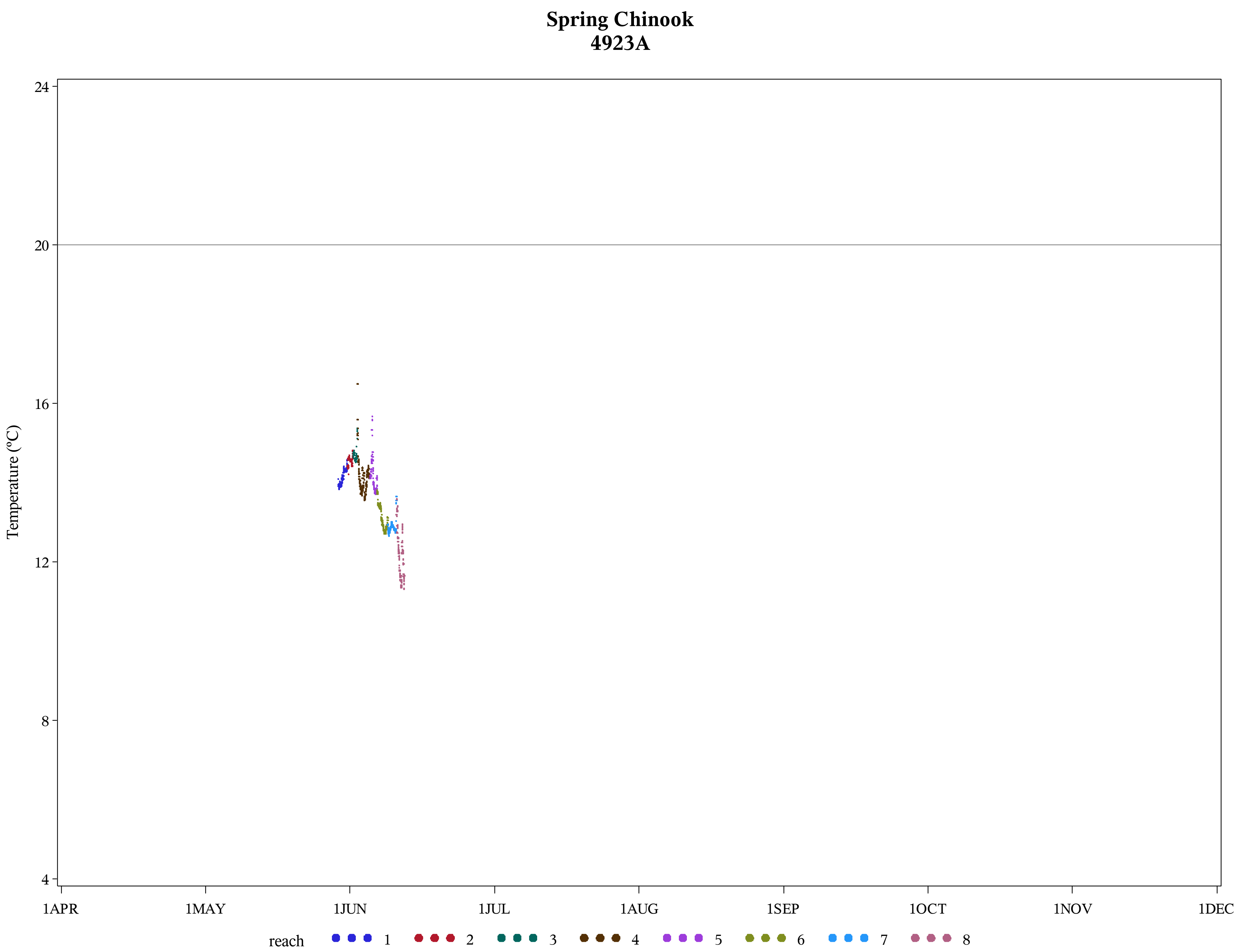
Spring Chinook
4923A
Temperature (°C)
24
20
16
12
8
4
1APR
1MAY
1JUN
1JUL
1AUG
1SEP
1OCT
1NOV
1DEC
reach
1
2
3
4
5
6
7
8

Spring Chinook
4924A

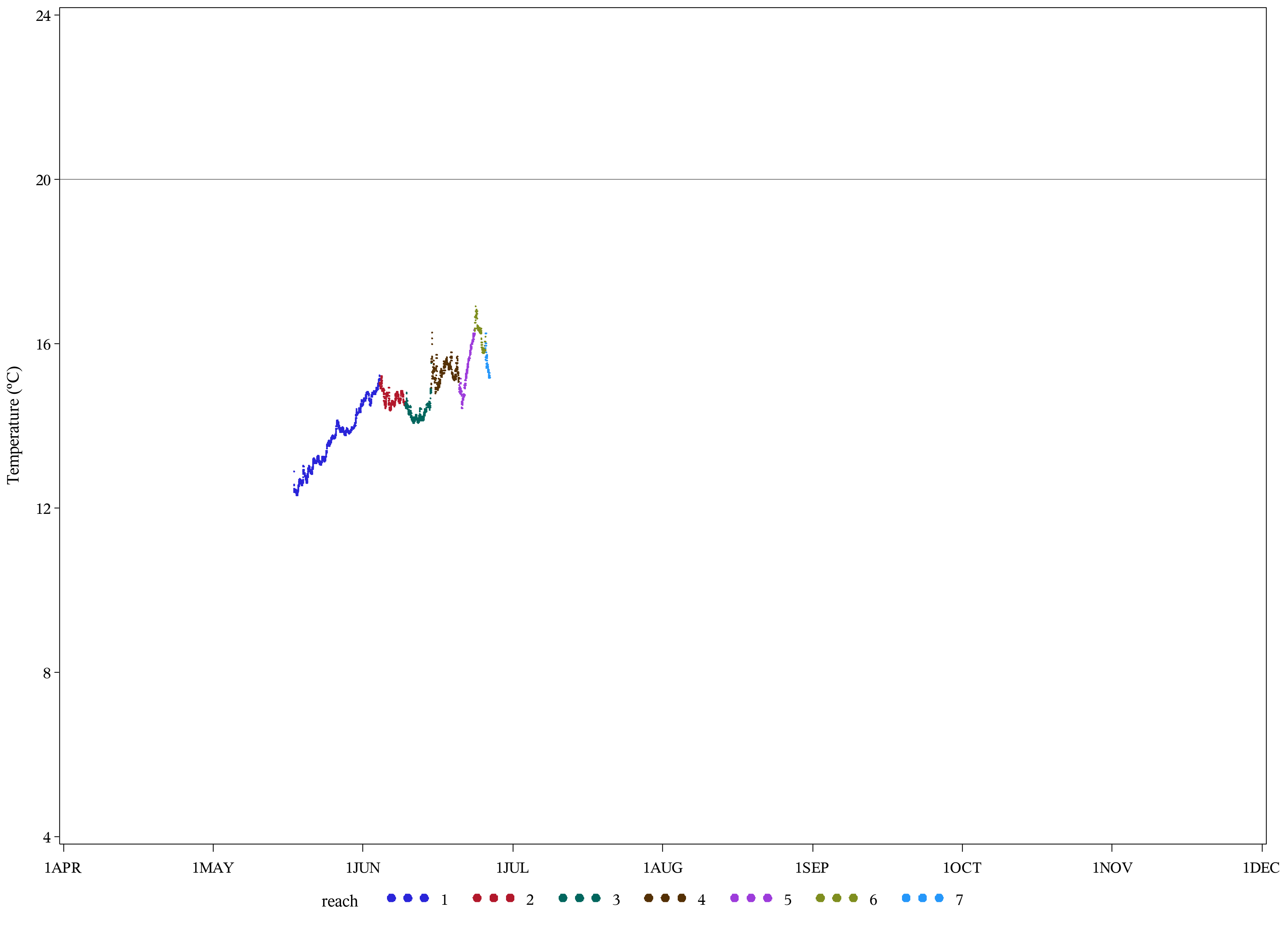

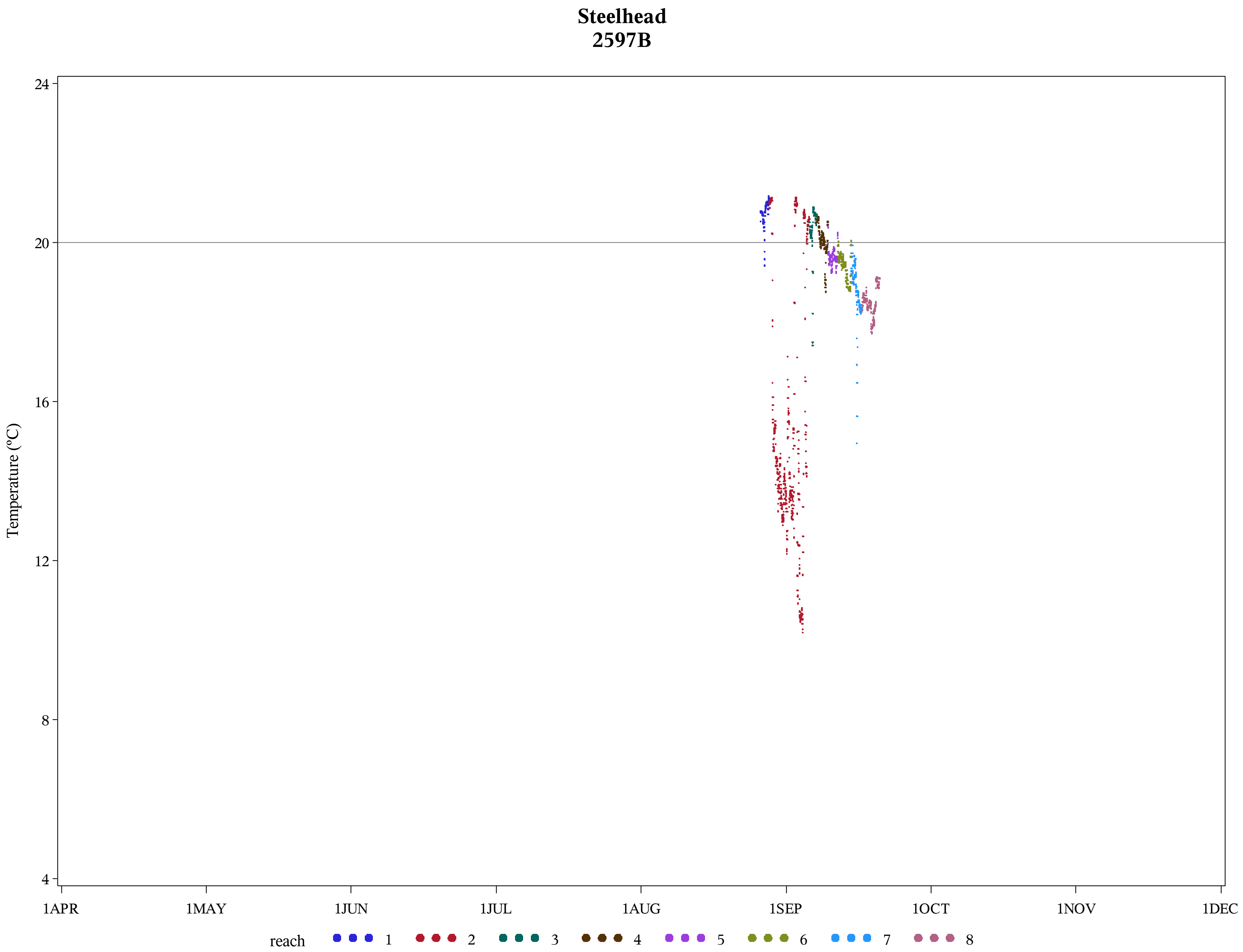

Steelhead
2597B
Temperature (°C)
24
20
16
12
8
4
1APR
1MAY
1JUN
1JUL
1AUG
1SEP
1OCT
1NOV
1DEC
reach
1
2
3
4
5
6
7
8

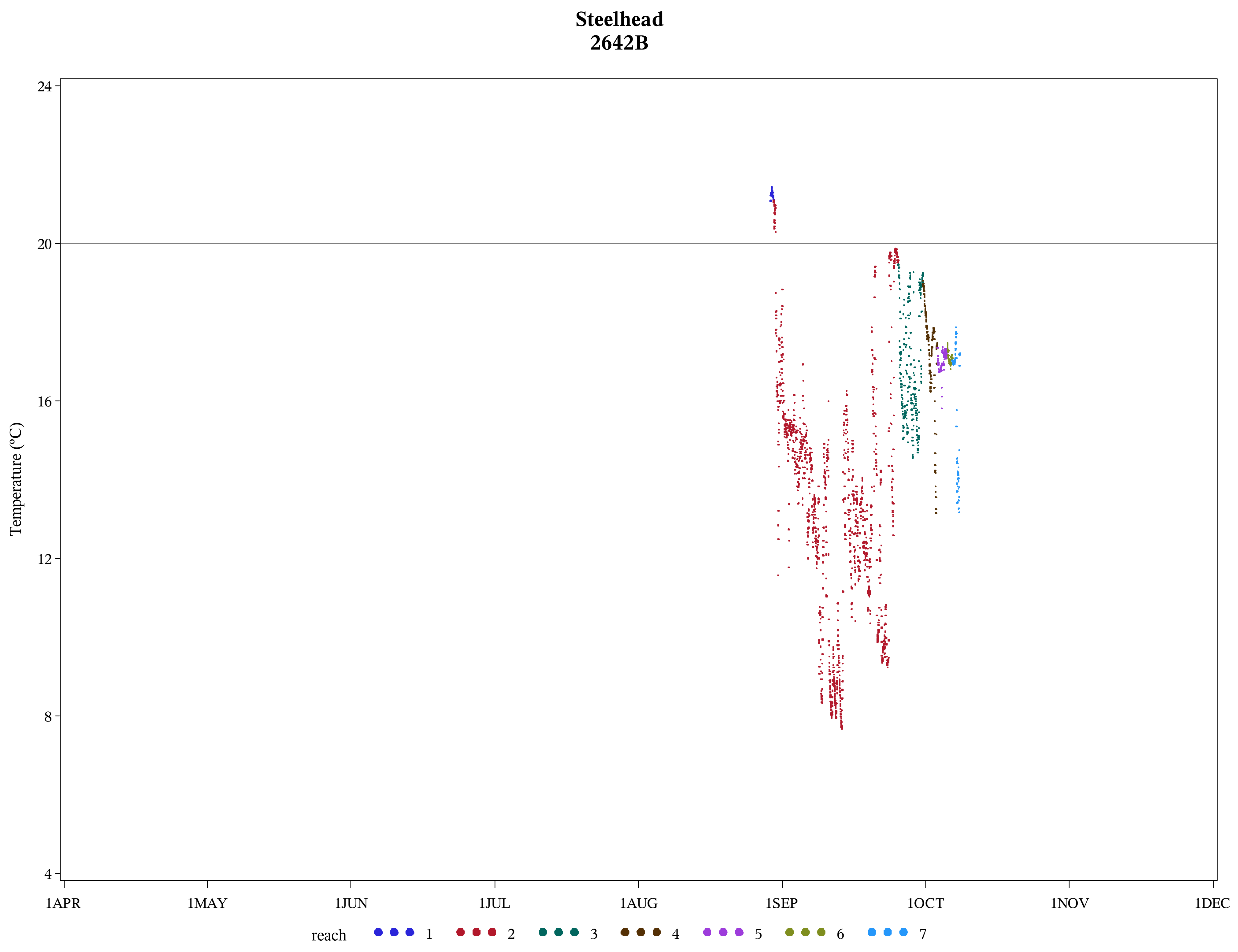

Steelhead
2642B
Temperature (°C)
24
20
16
12
8
4
1APR
1MAY
1JUN
1JUL
1AUG
1SEP
1OCT
1NOV
1DEC
reach
1
2
3
4
5
6
7

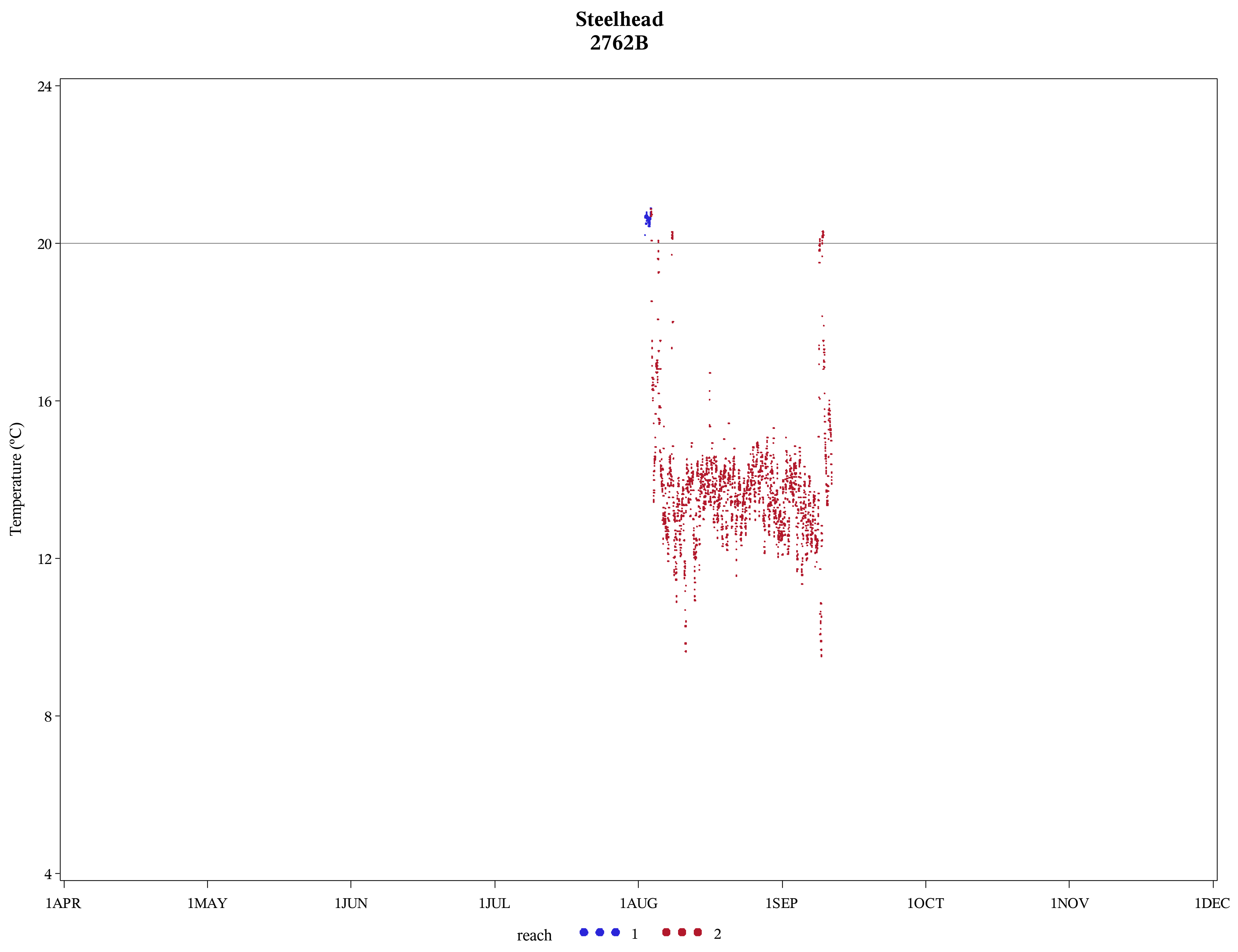

Steelhead
2762B
Temperature (°C)
24
20
16
12
8
4
1APR
1MAY
1JUN
1JUL
1AUG
1SEP
1OCT
1NOV
1DEC
reach
1
2

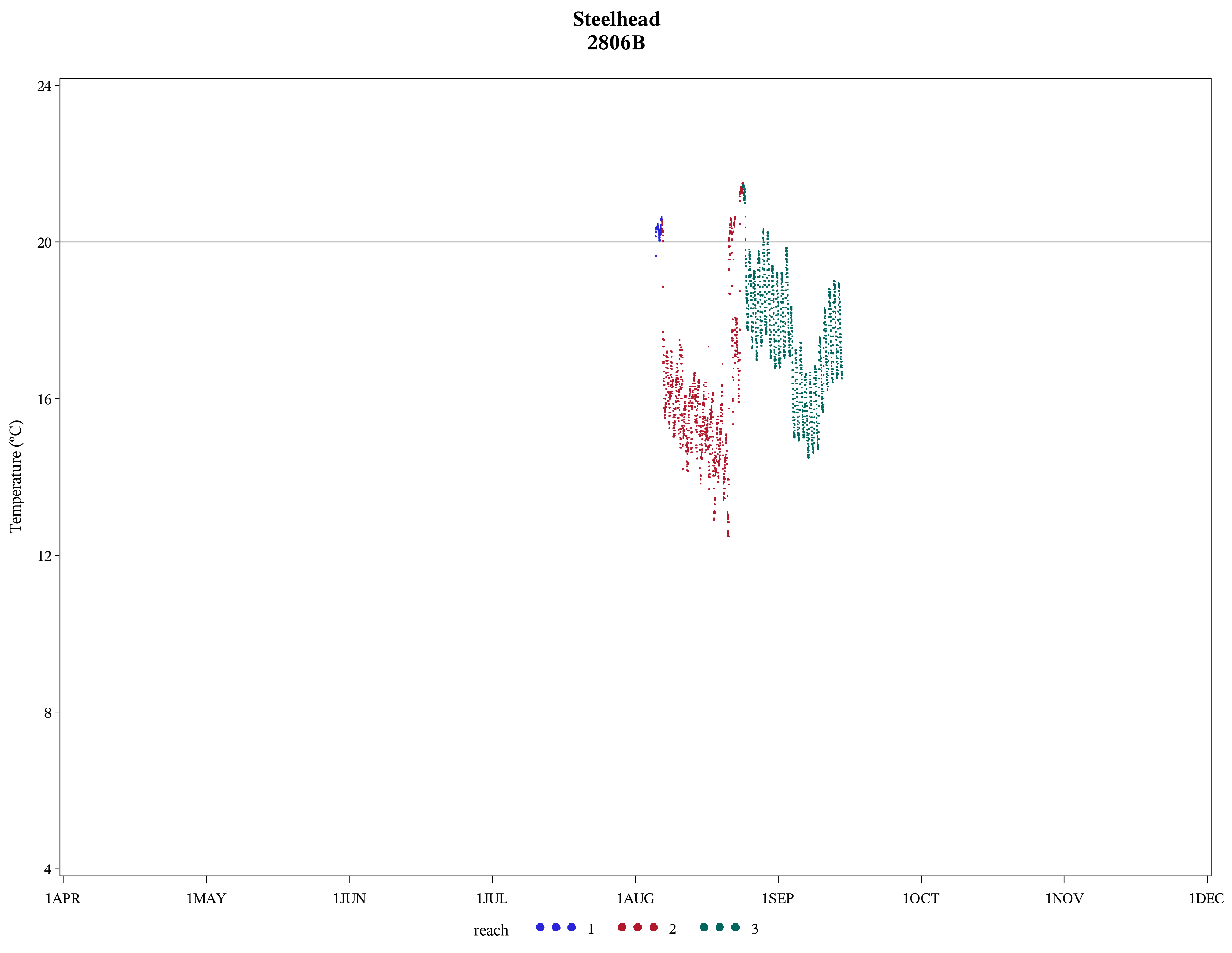

Steelhead
2806B
Temperature (°C)
24
20
16
12
8
4
1APR
1MAY
1JUN
1JUL
1AUG
1SEP
1OCT
1NOV
1DEC
reach
1
2
3

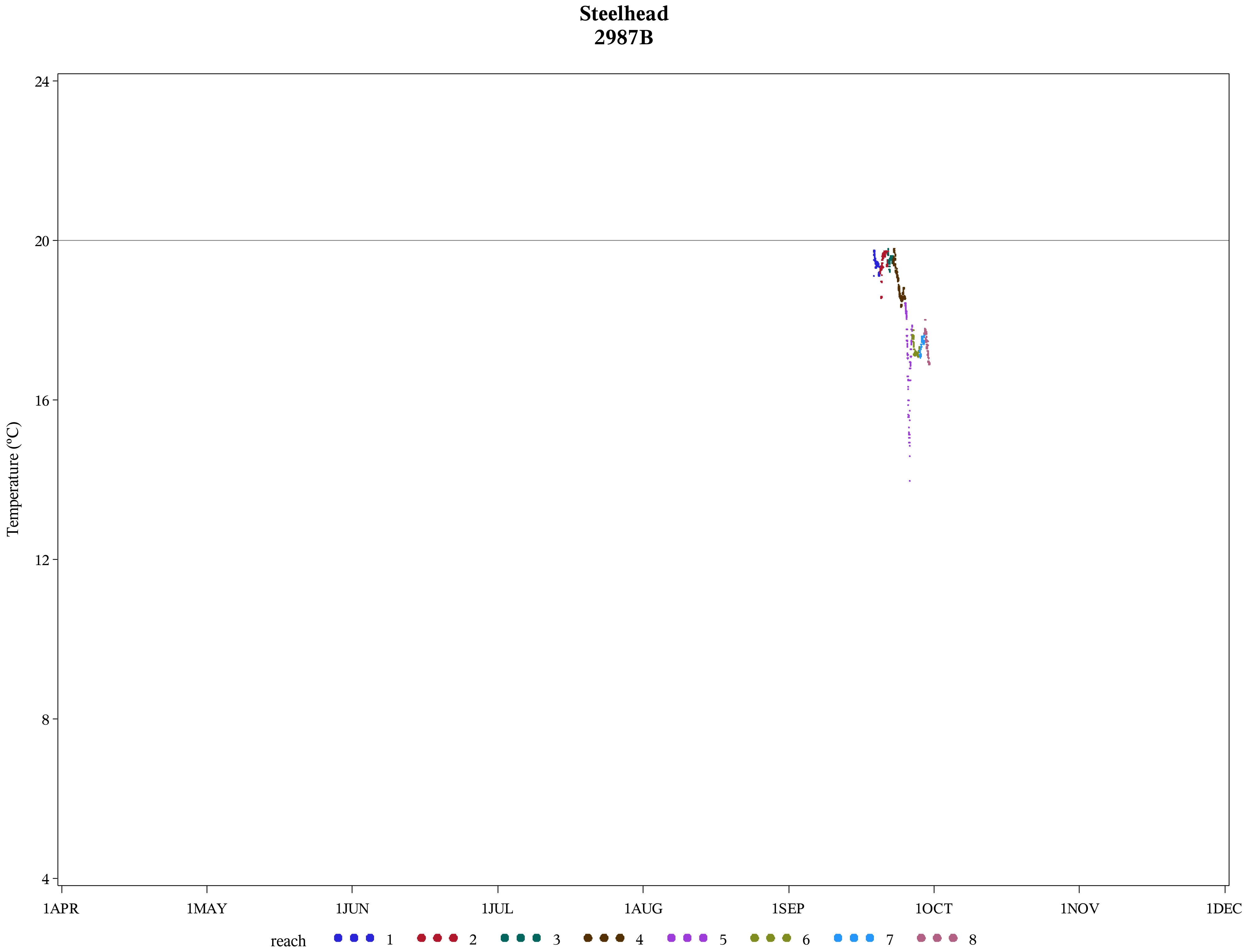
Steelhead
2987B
Temperature (°C)
24
20
16
12
8
4
1APR
1MAY
1JUN
1JUL
1AUG
1SEP
1OCT
1NOV
1DEC
reach
1
2
3
4
5
6
7
8

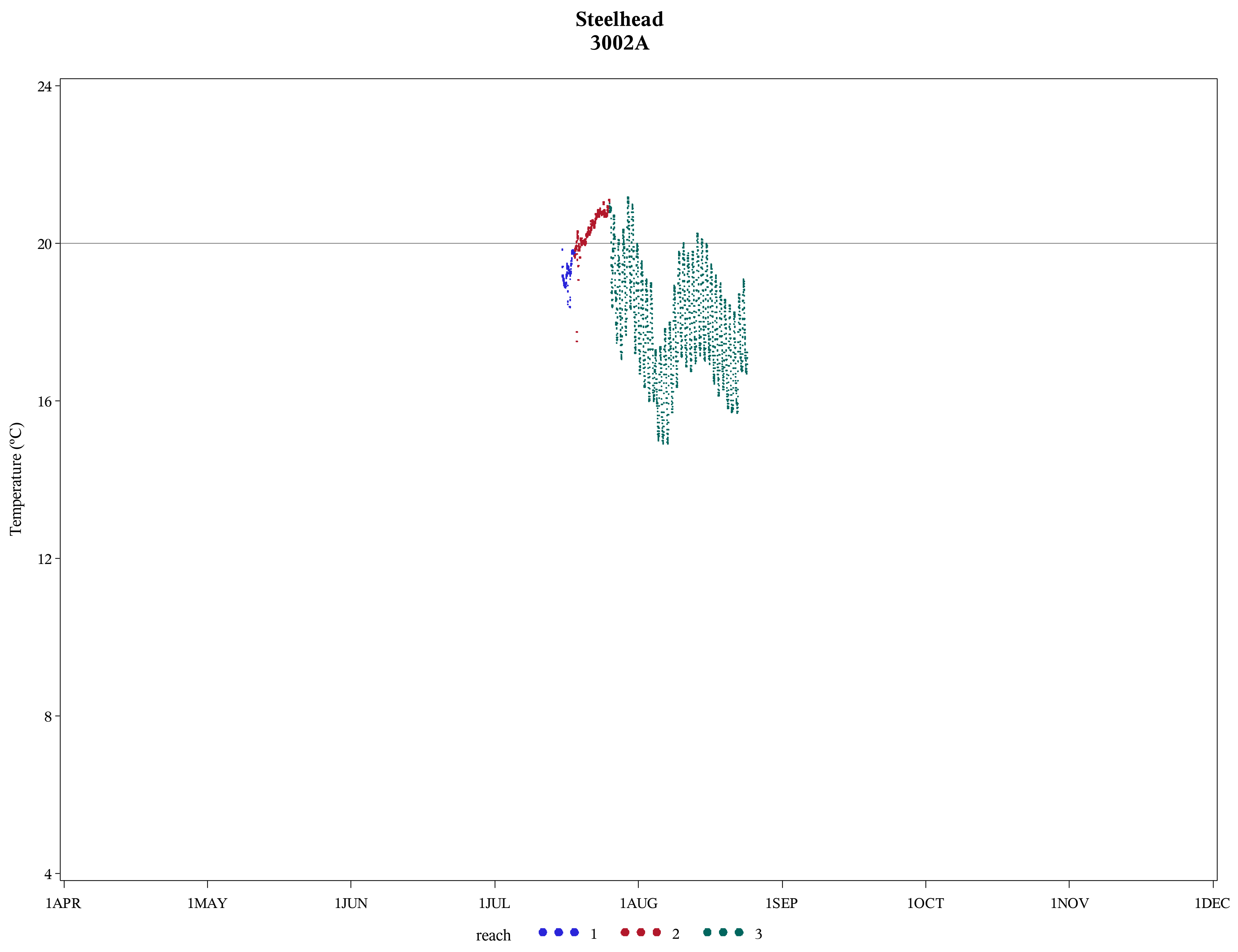

Steelhead
3002A
Temperature (°C)
24
20
16
12
8
4
1APR
1MAY
1JUN
1JUL
1AUG
1SEP
1OCT
1NOV
1DEC
reach
1
2
3

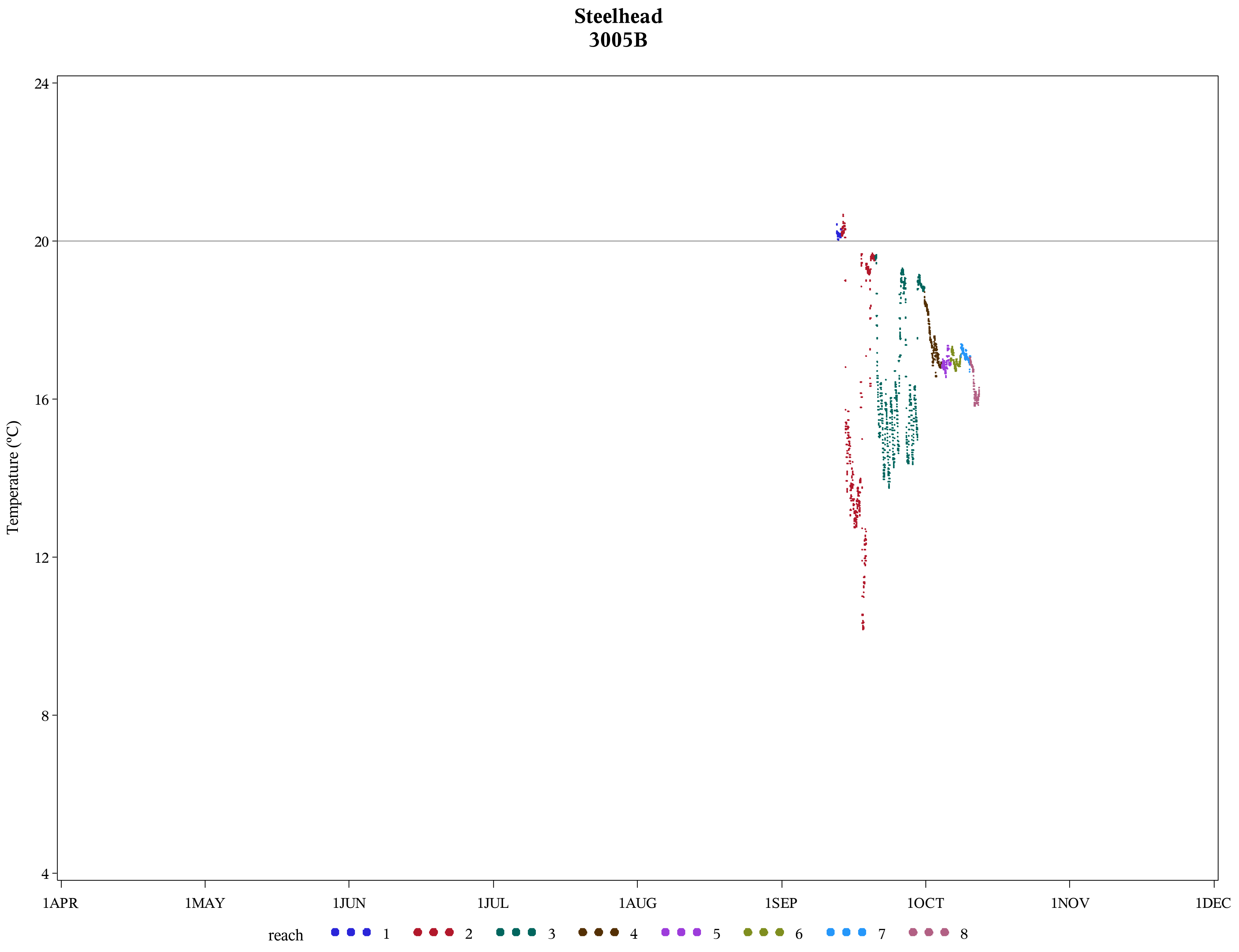

Steelhead
3005B
Temperature (°C)
24
20
16
12
8
4
1APR
1MAY
1JUN
1JUL
1AUG
1SEP
1OCT
1NOV
1DEC
reach
1
2
3
4
5
6
7
8

Steelhead
3076B

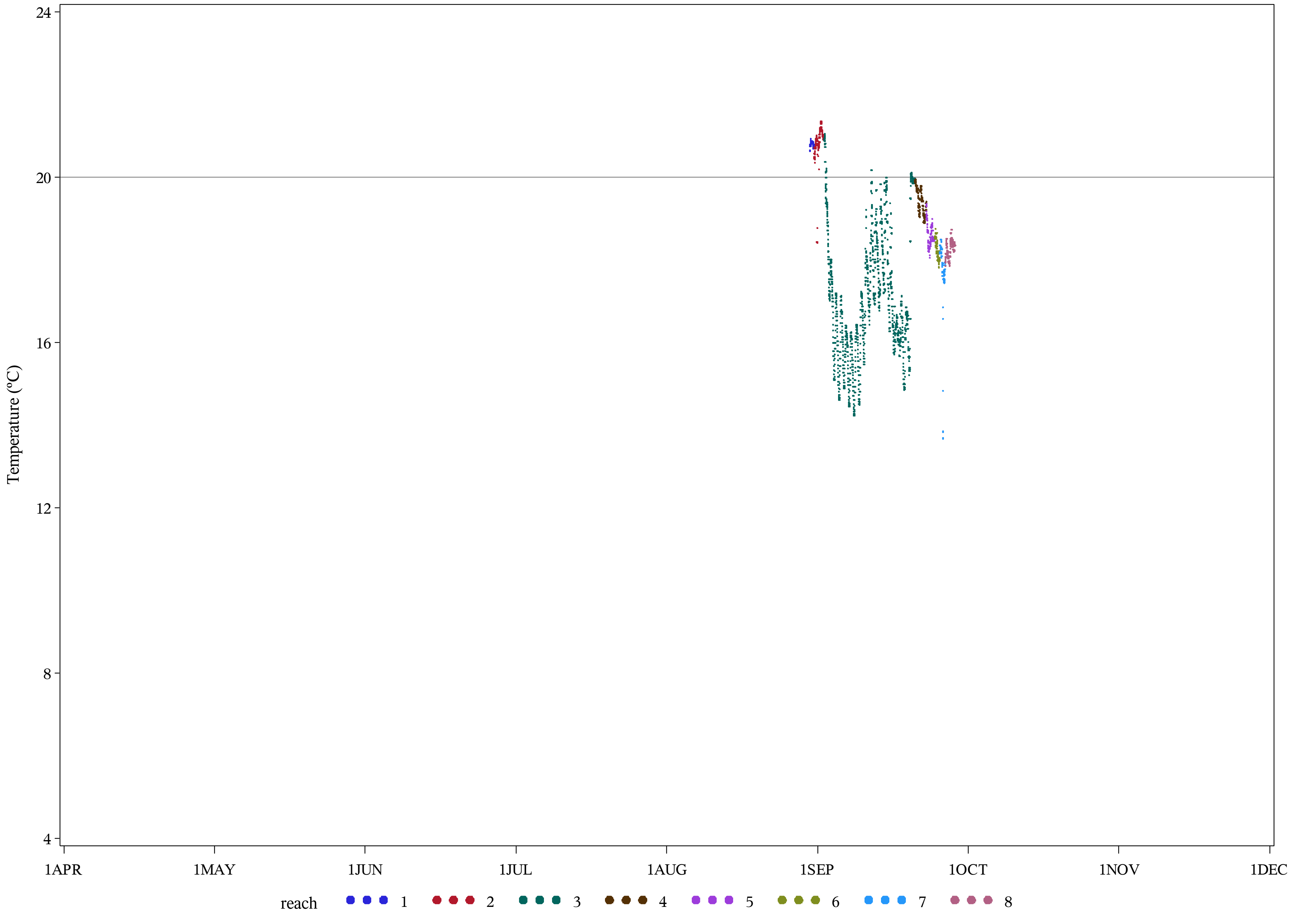

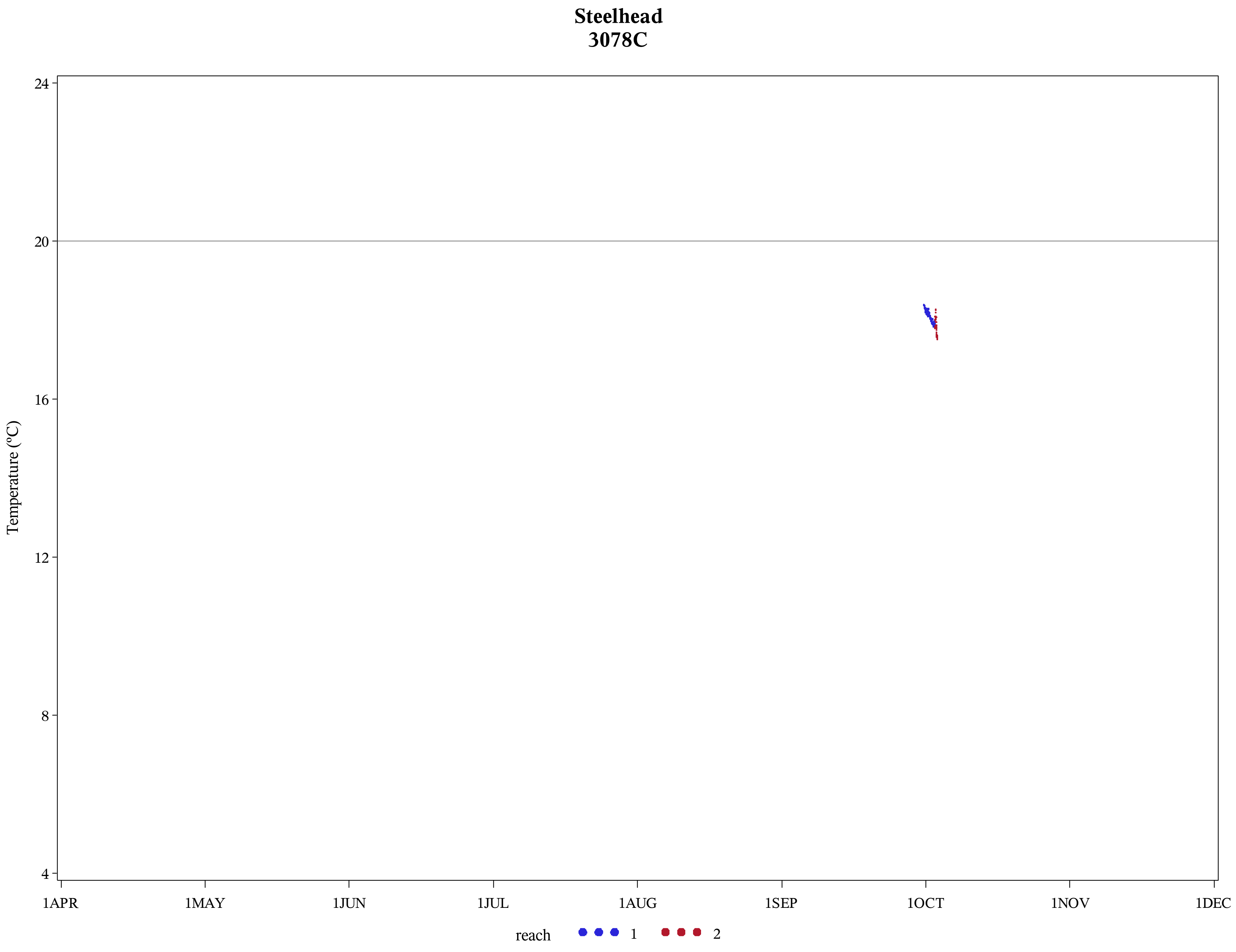
Steelhead
3078C
Temperature (°C)
24
20
16
12
8
4
1APR
1MAY
1JUN
1JUL
1AUG
1SEP
1OCT
1NOV
1DEC
reach
1
2

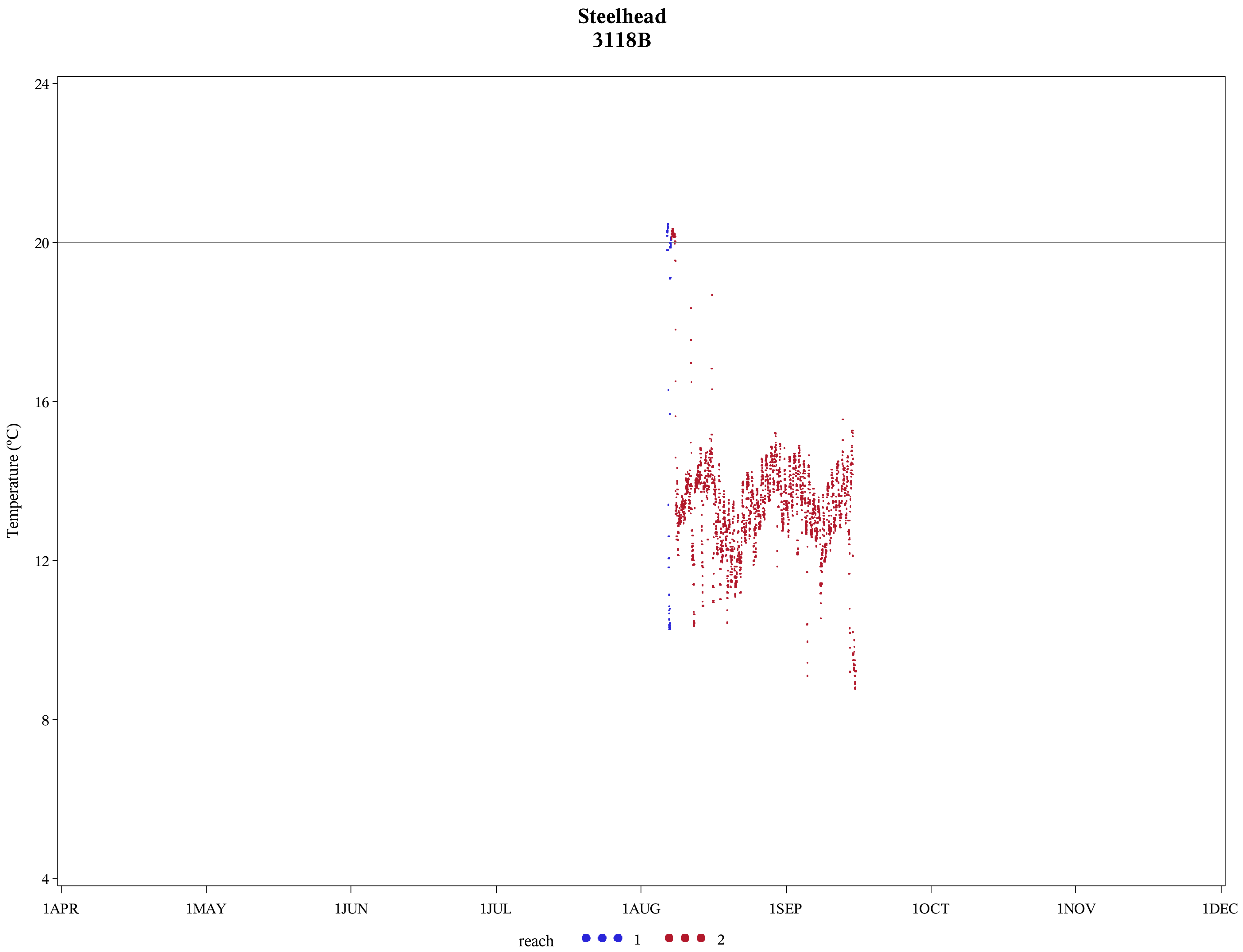

Steelhead
3118B
Temperature (°C)
24
20
16
12
8
4
1APR
1MAY
1JUN
1JUL
1AUG
1SEP
1OCT
1NOV
1DEC
reach
1
2

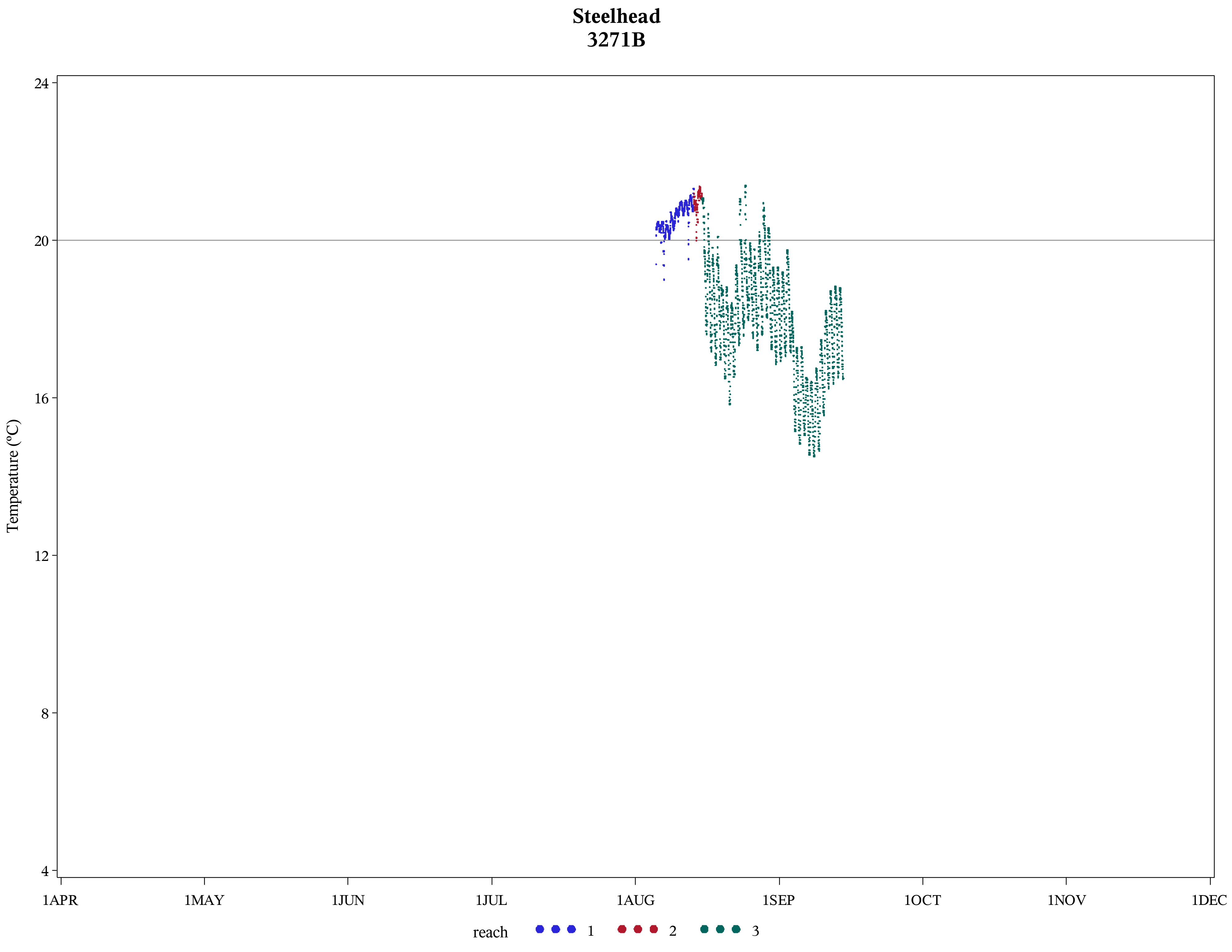

Steelhead
3271B
Temperature (°C)
24
20
16
12
8
4
1APR
1MAY
1JUN
1JUL
1AUG
1SEP
1OCT
1NOV
1DEC
reach
1
2
3

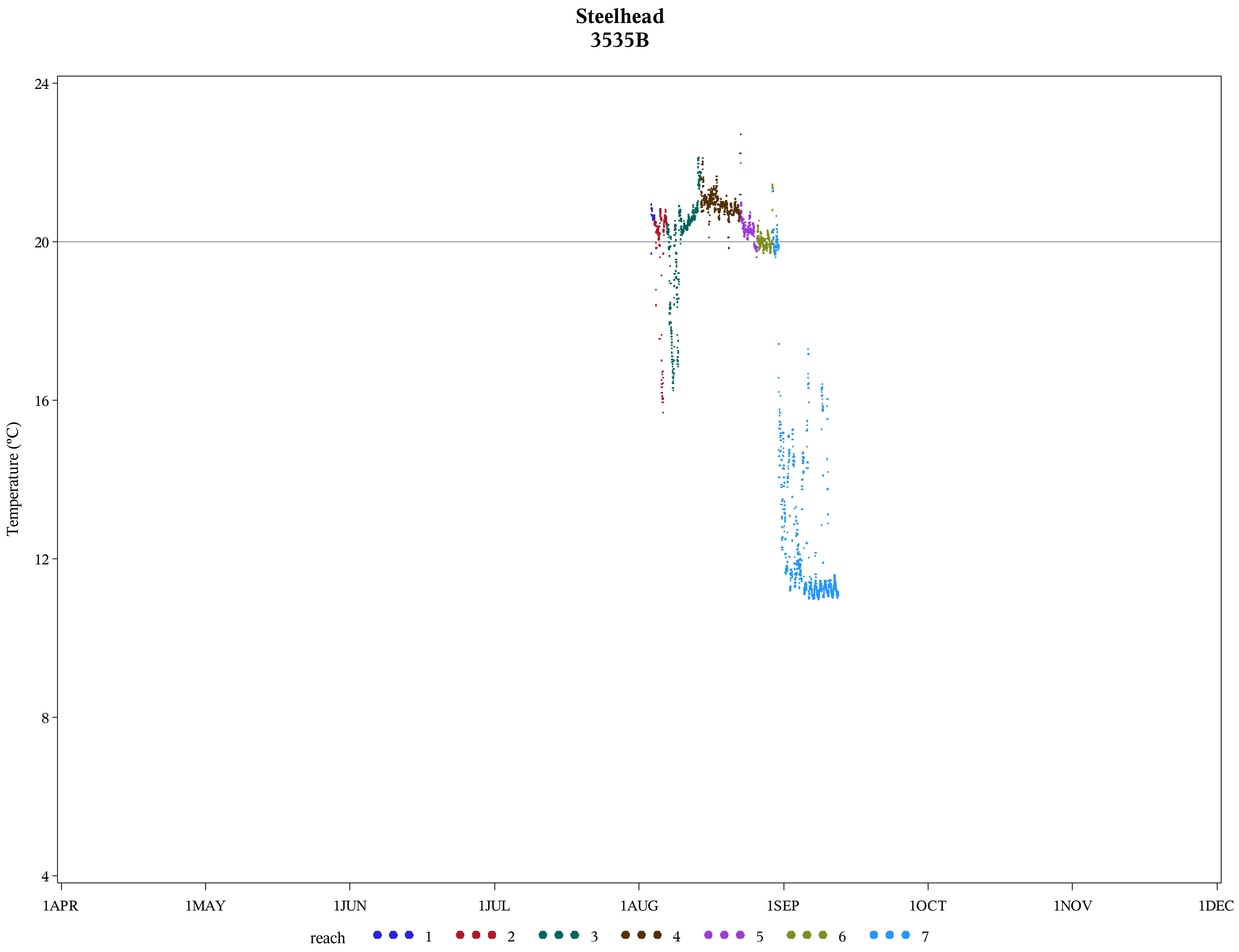
Steelhead
3535B
Temperature (°C)
24
20
16
12
8
4
1APR
1MAY
1JUN
1JUL
1AUG
1SEP
1OCT
1NOV
1DEC
reach
1
2
3
4
5
6
7

Steelhead
3551B

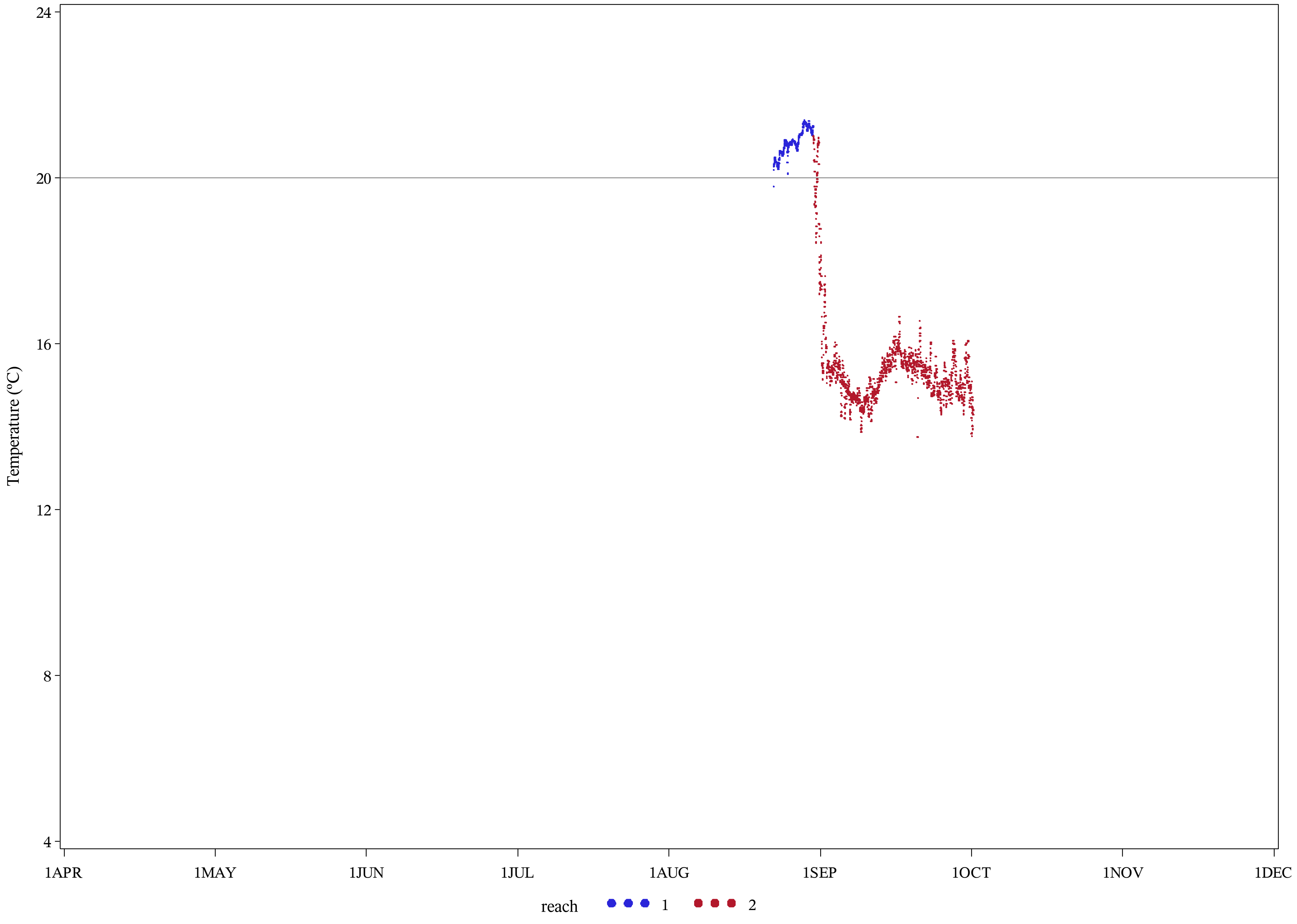

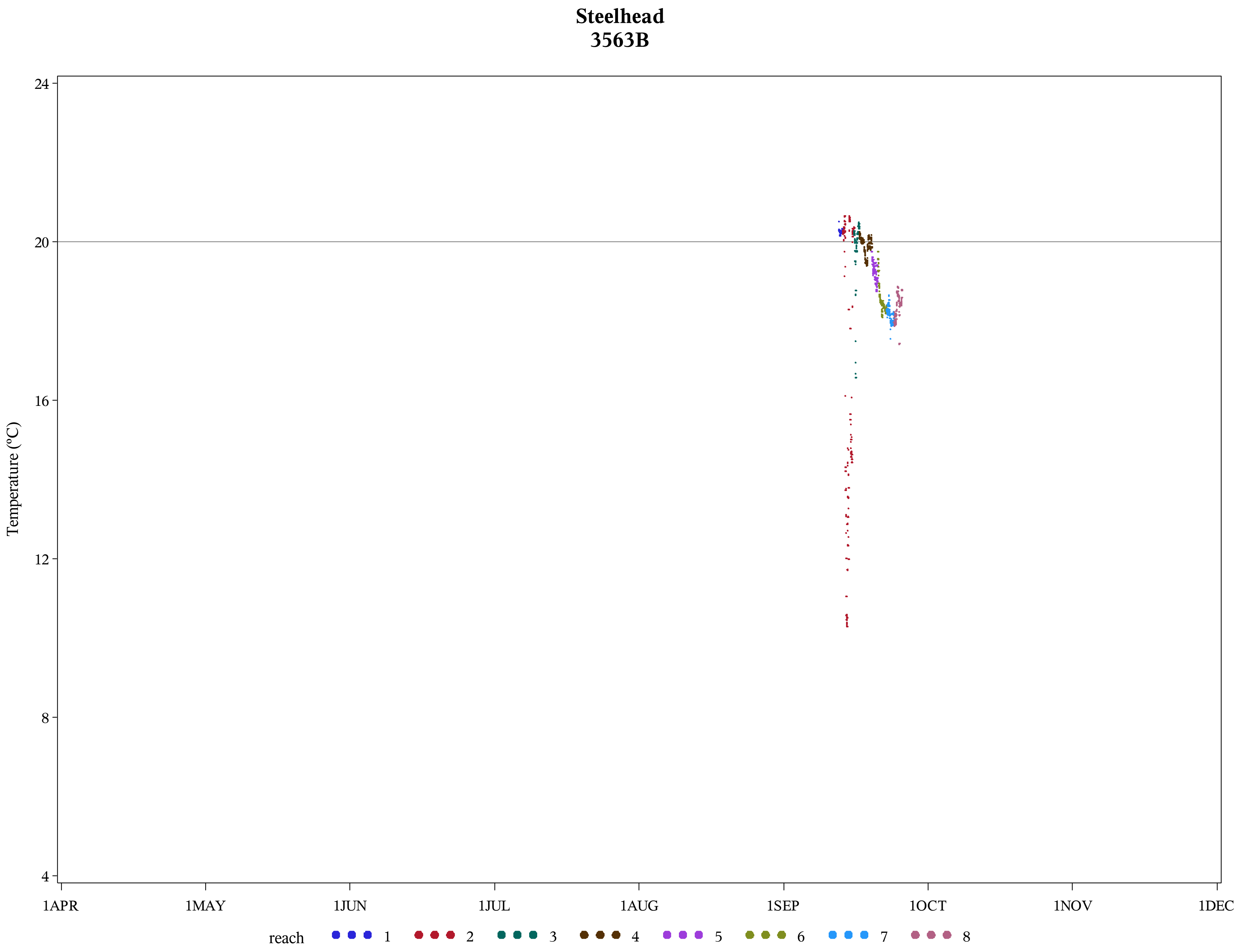

Steelhead
3563B
Temperature (°C)
24
20
16
12
8
4
1APR
1MAY
1JUN
1JUL
1AUG
1SEP
1OCT
1NOV
1DEC
reach
1
2
3
4
5
6
7
8

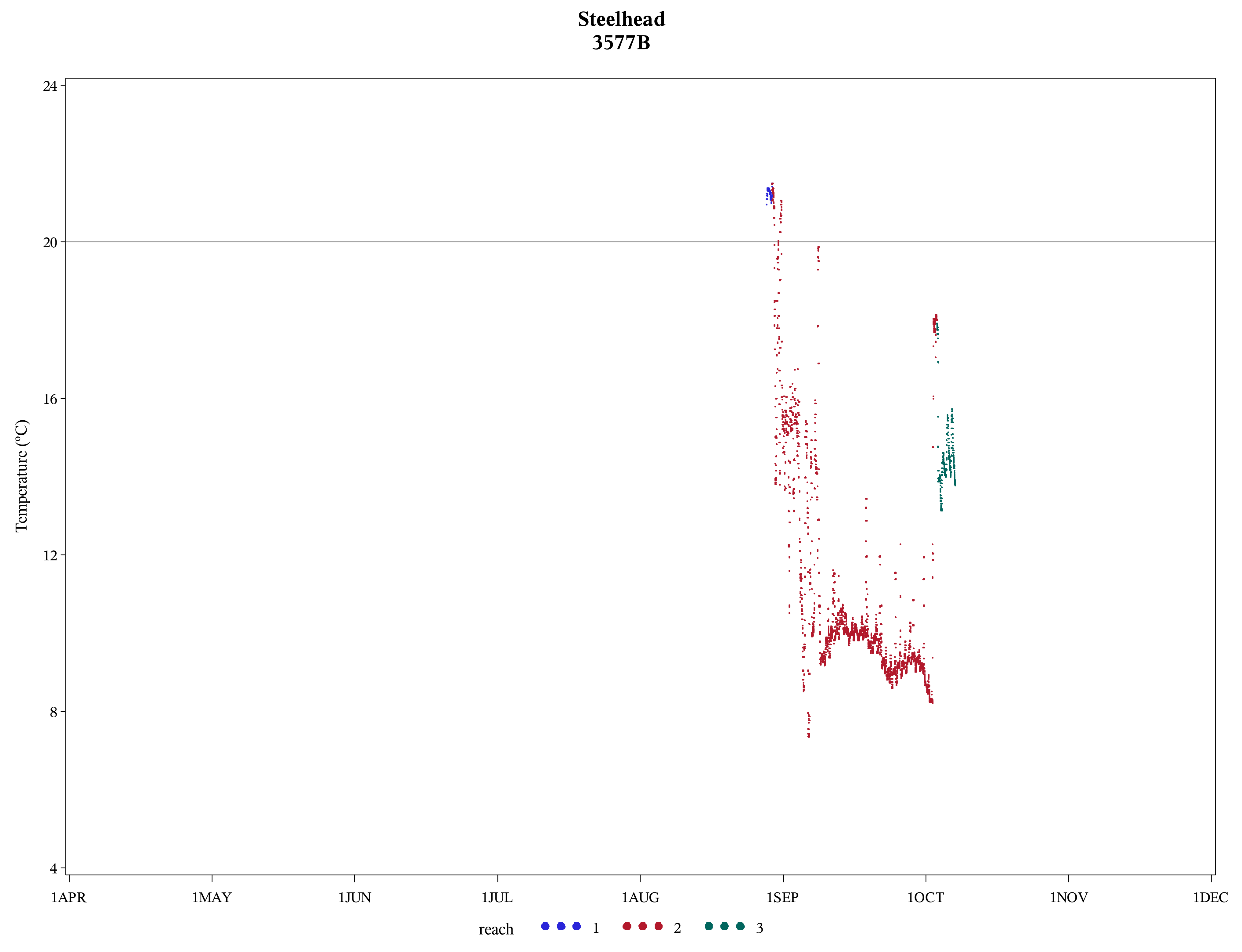
Steelhead
3577B
Temperature (°C)
24
20
16
12
8
4
1APR
1MAY
1JUN
1JUL
1AUG
1SEP
1OCT
1NOV
1DEC
reach
1
2
3

Steelhead
3705B

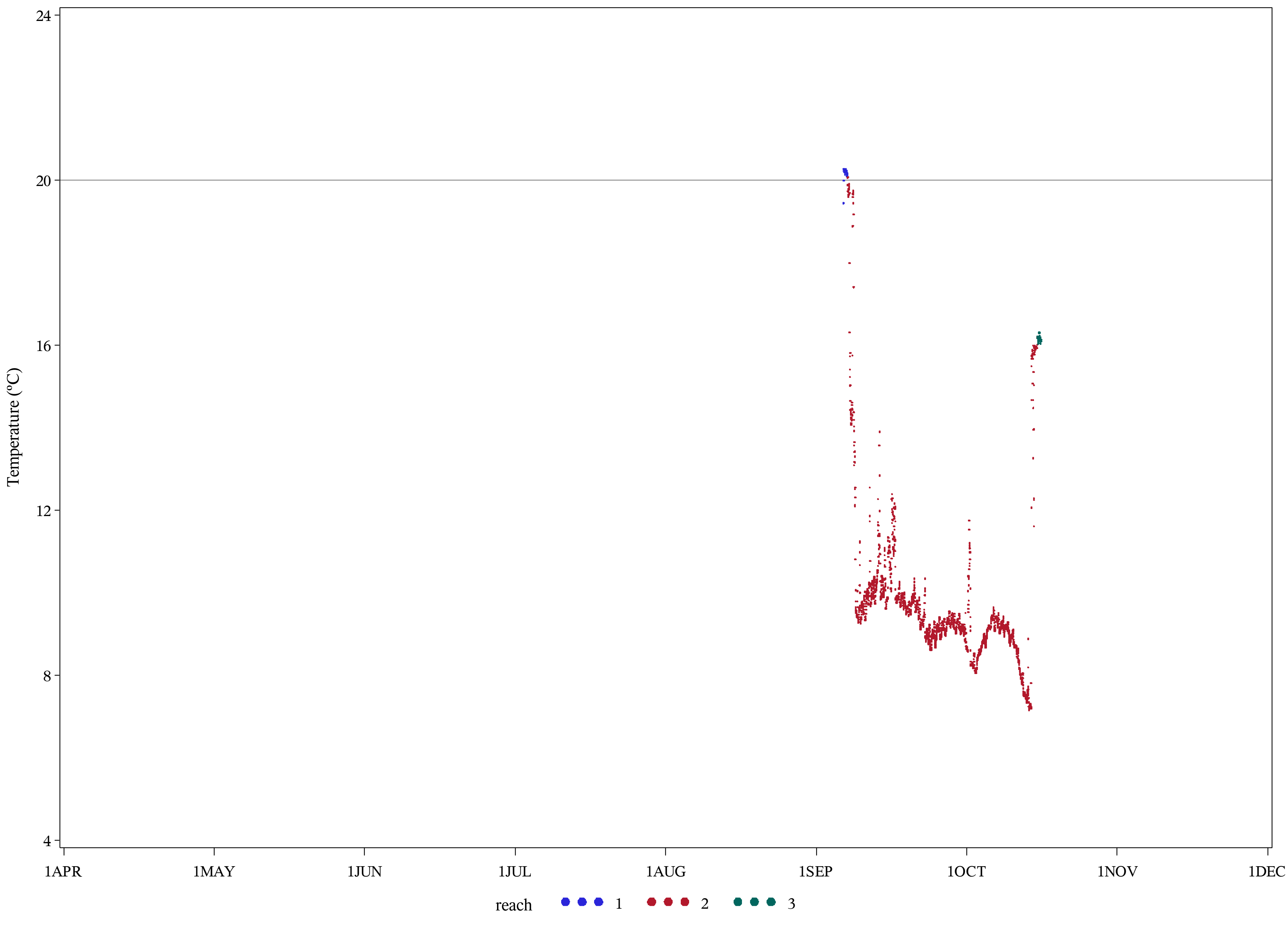

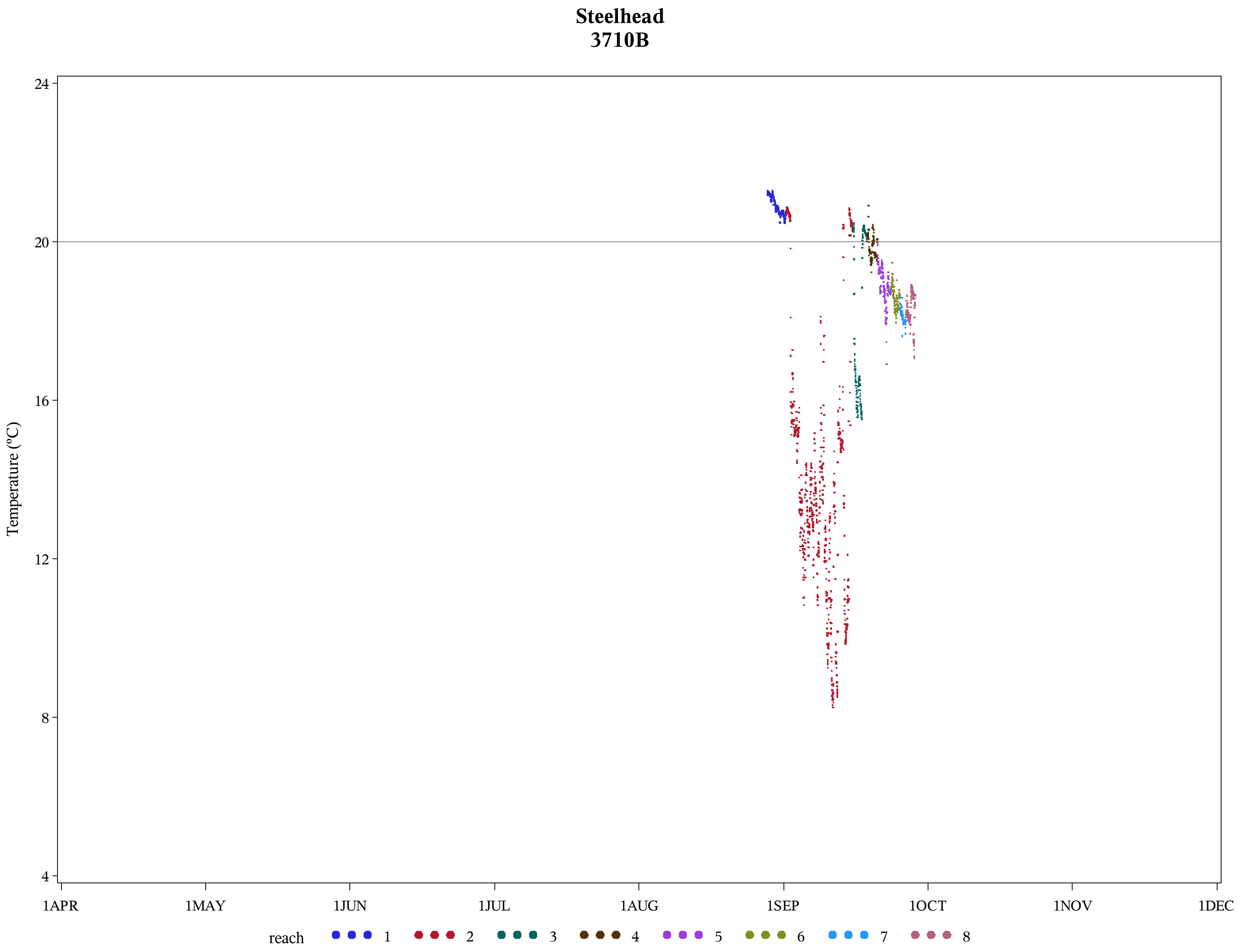

Steelhead
3710B
Temperature (°C)
24
20
16
12
8
4
1APR
1MAY
1JUN
1JUL
1AUG
1SEP
1OCT
1NOV
1DEC
reach
1
2
3
4
5
6
7
8

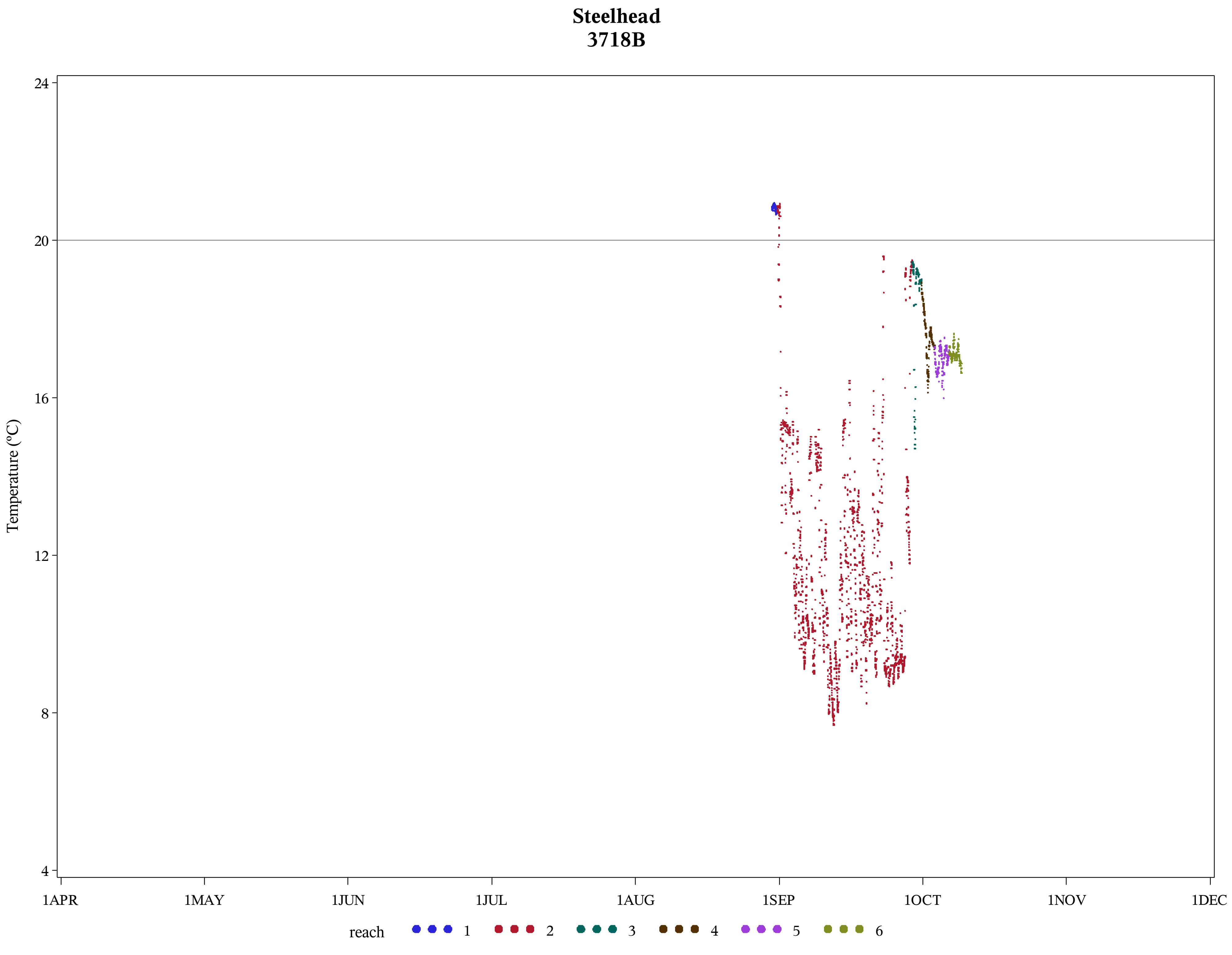
Steelhead
3718B
Temperature (°C)
24
20
16
12
8
4
1APR
1MAY
1JUN
1JUL
1AUG
1SEP
1OCT
1NOV
1DEC
reach
1
2
3
4
5
6

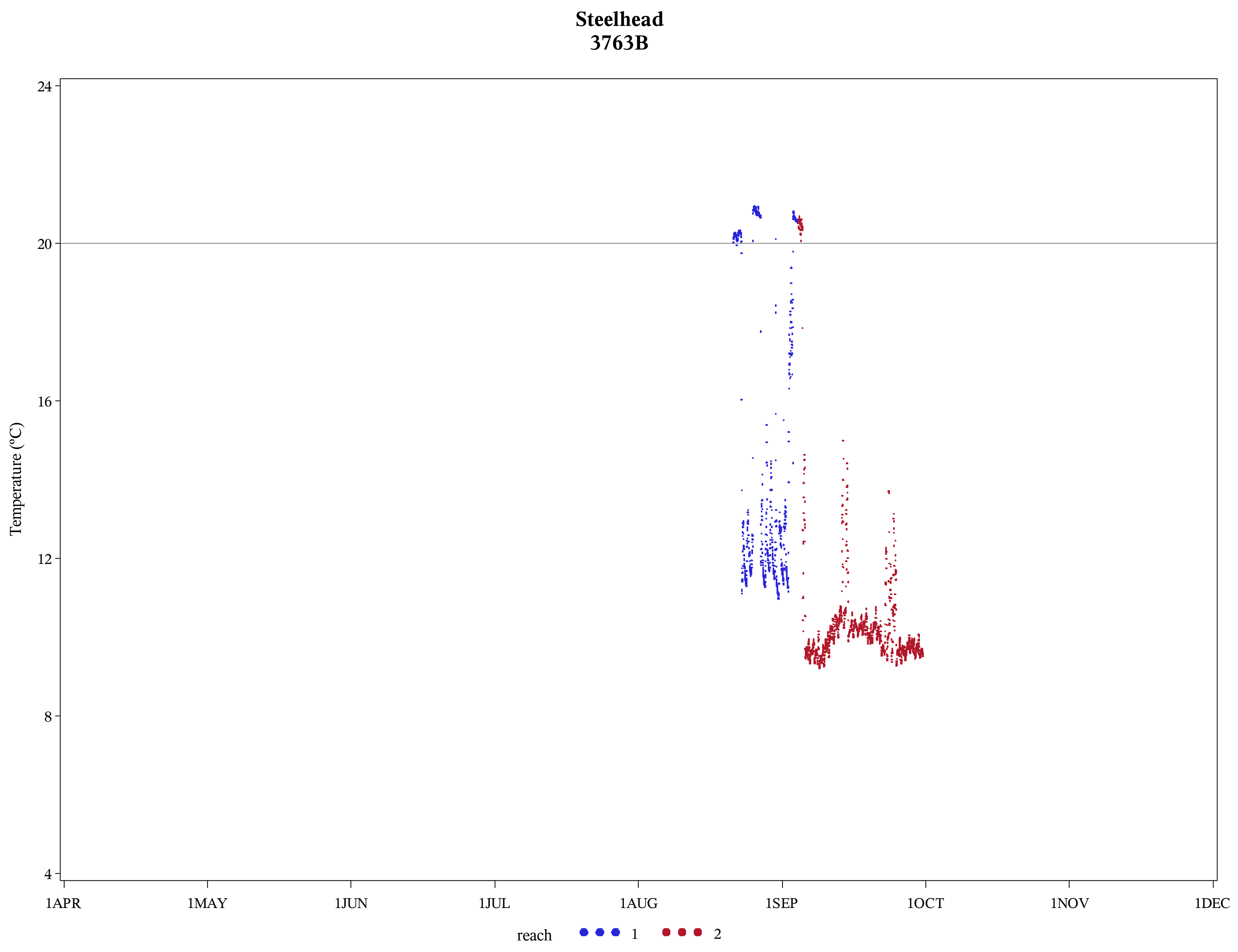

Steelhead
3763B
Temperature (°C)
24
20
16
12
8
4
1APR
1MAY
1JUN
1JUL
1AUG
1SEP
1OCT
1NOV
1DEC
reach
1
2

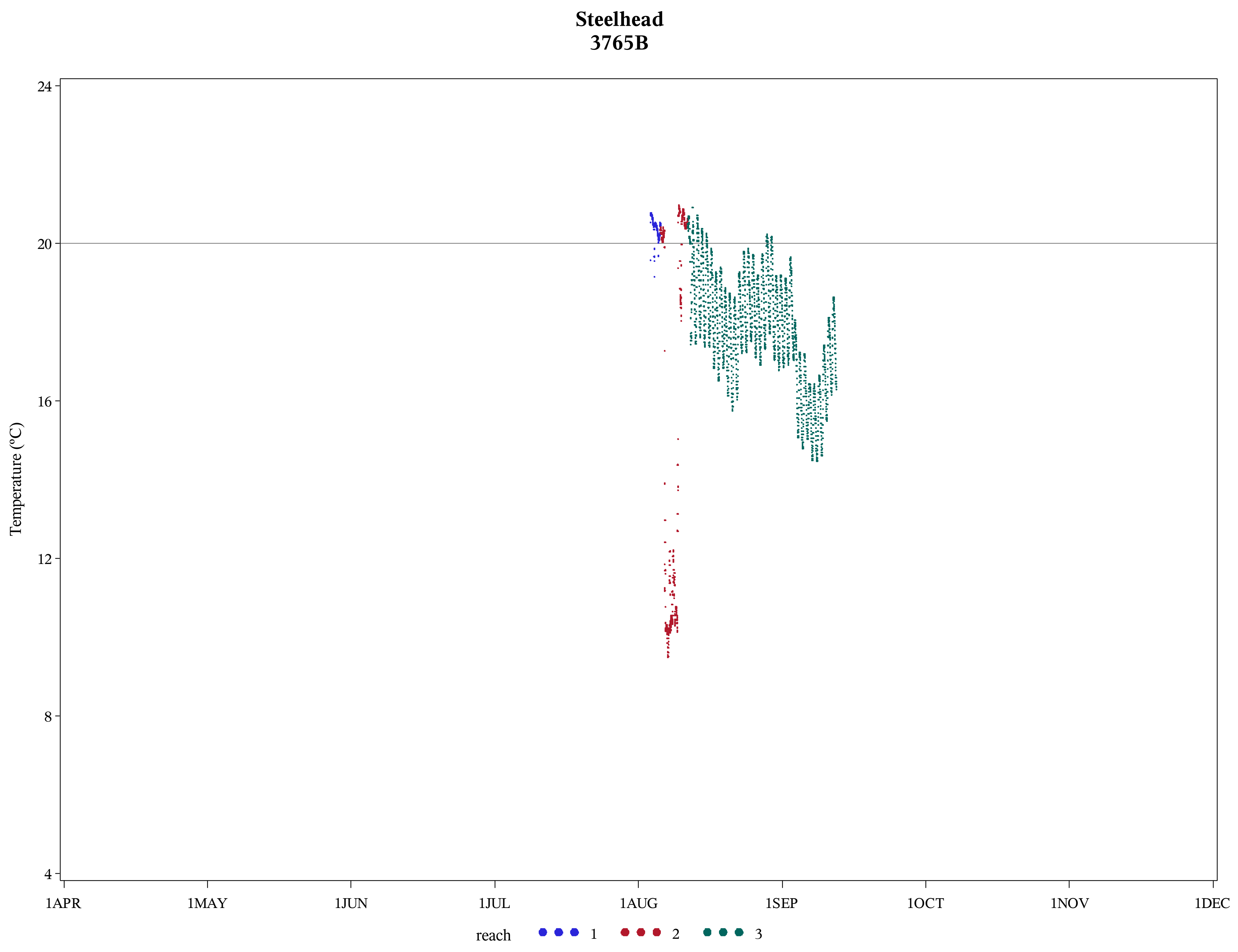
Steelhead
3765B
Temperature (°C)
24
20
16
12
8
4
1APR
1MAY
1JUN
1JUL
1AUG
1SEP
1OCT
1NOV
1DEC
reach
1
2
3

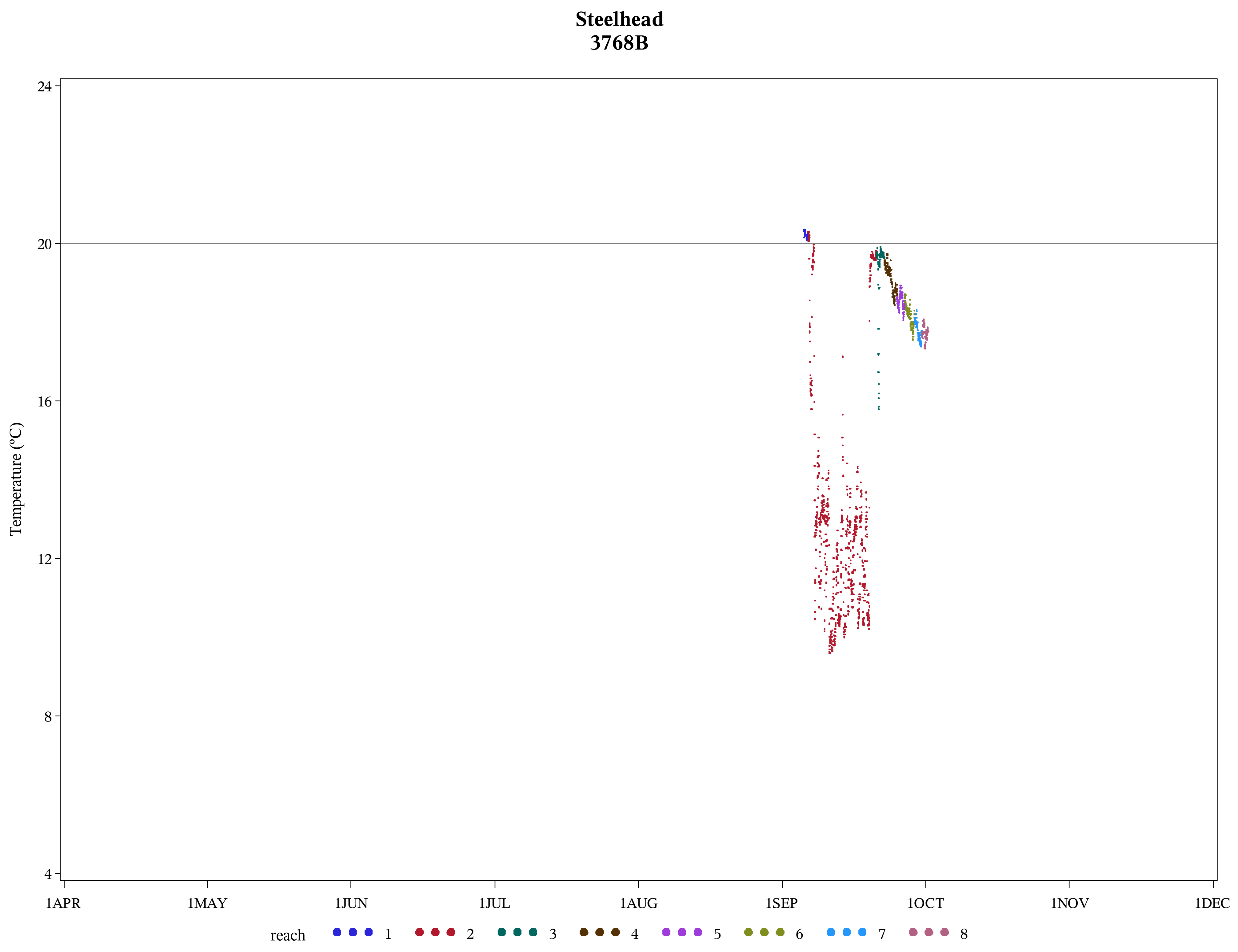
Steelhead
3768B
Temperature (°C)
24
20
16
12
8
4
1APR
1MAY
1JUN
1JUL
1AUG
1SEP
1OCT
1NOV
1DEC
reach
1
2
3
4
5
6
7
8

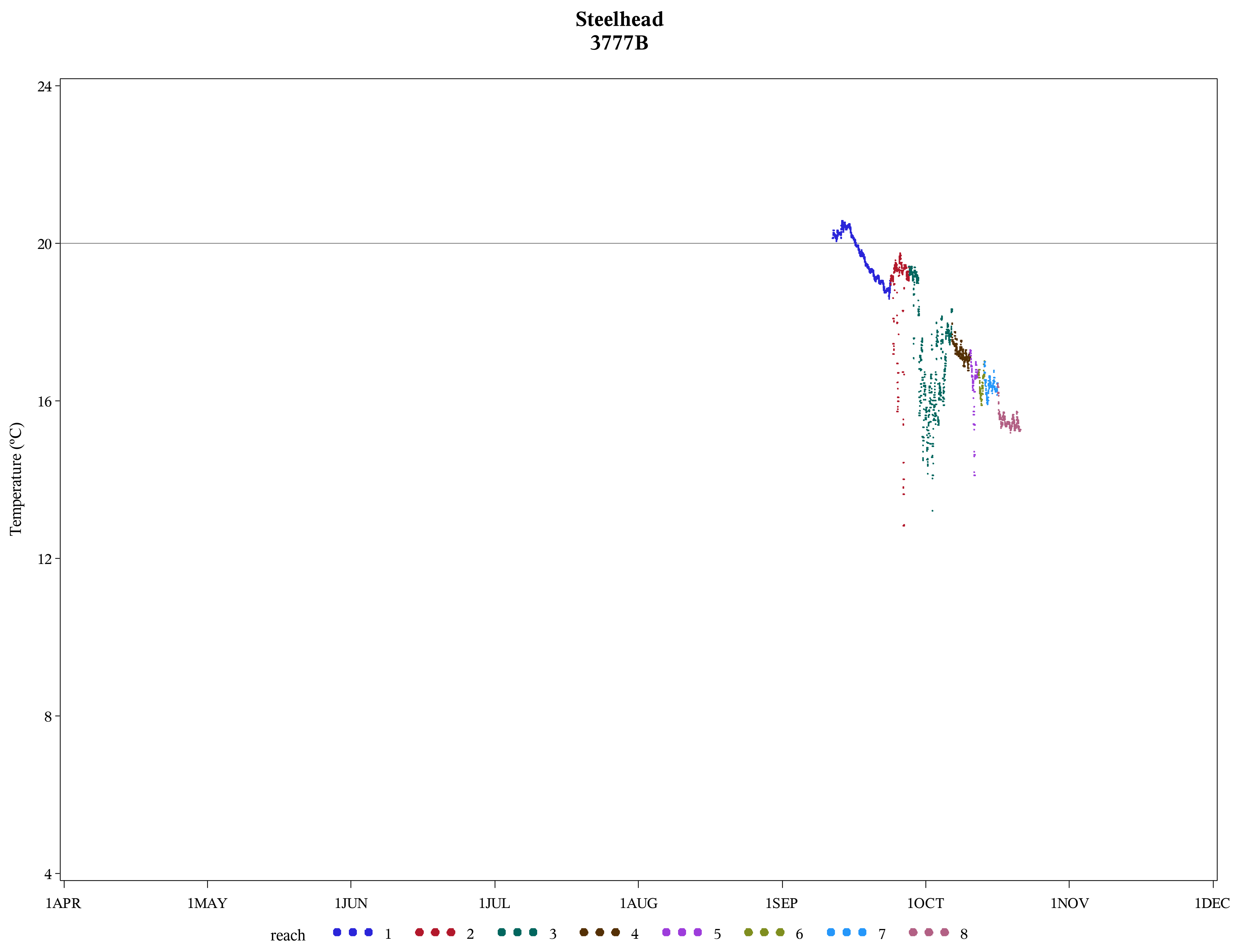

Steelhead
3777B
Temperature (°C)
24
20
16
12
8
4
1APR
1MAY
1JUN
1JUL
1AUG
1SEP
1OCT
1NOV
1DEC
reach
1
2
3
4
5
6
7
8

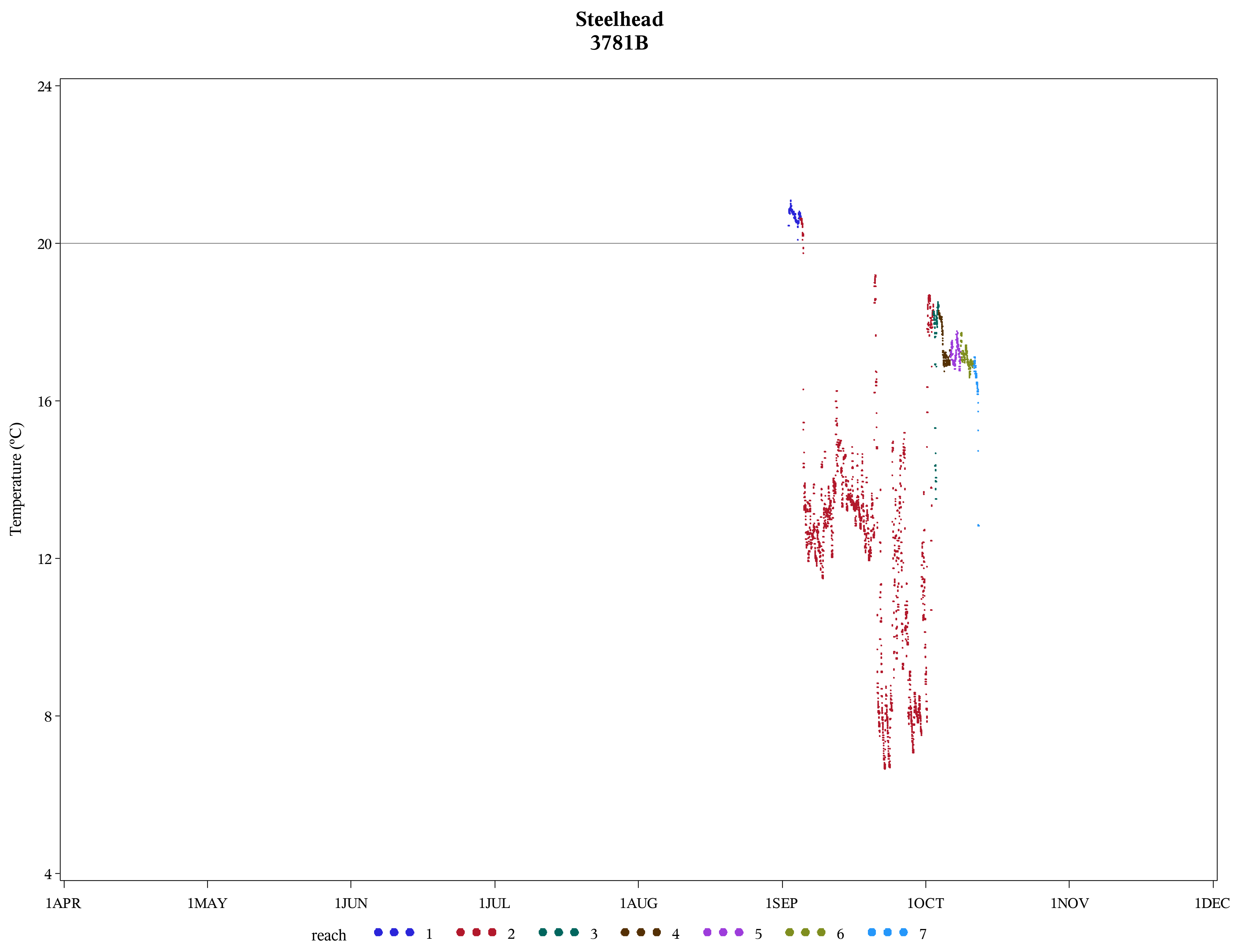

Steelhead
3781B
Temperature (°C)
24
20
16
12
8
4
1APR
1MAY
1JUN
1JUL
1AUG
1SEP
1OCT
1NOV
1DEC
reach
1
2
3
4
5
6
7

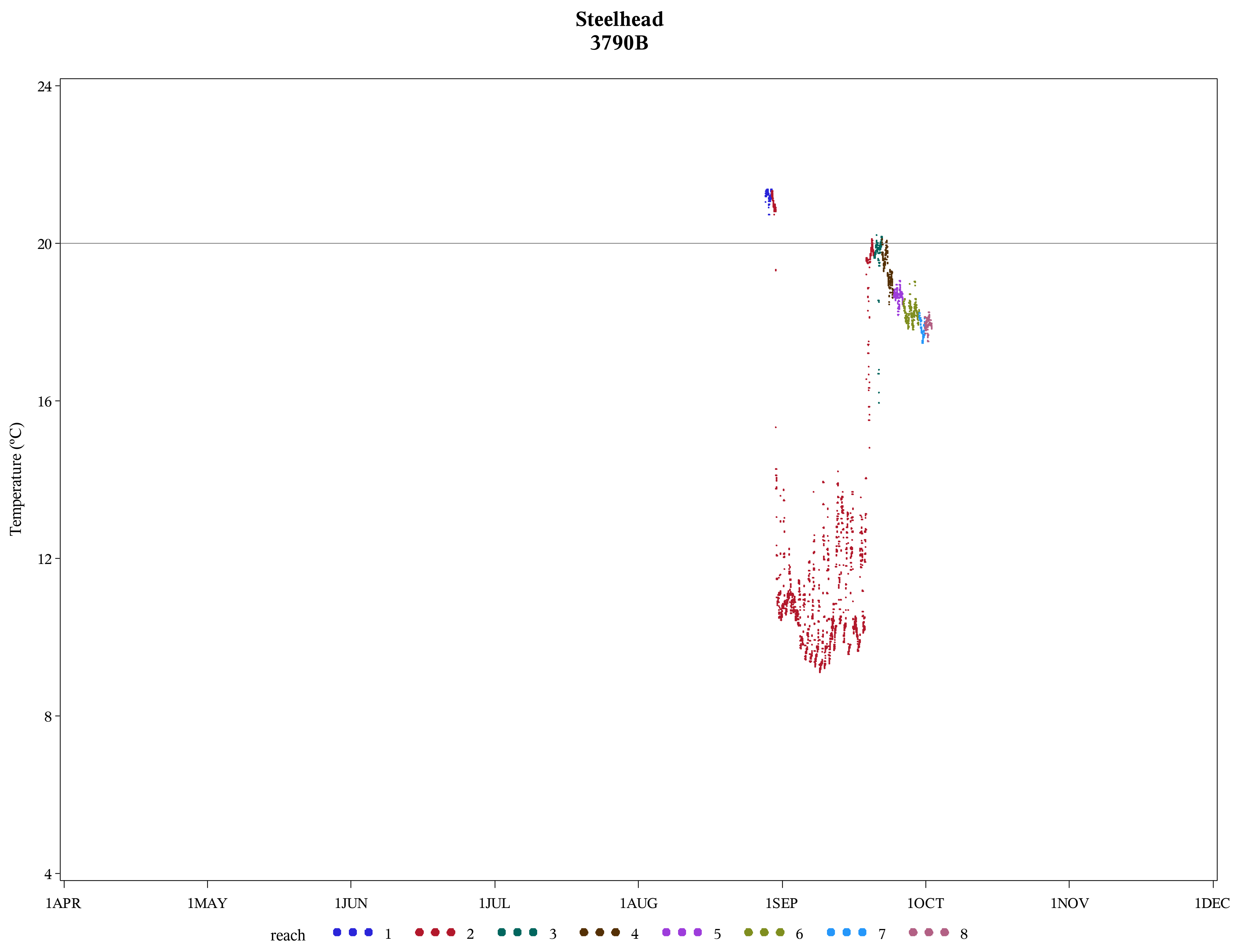

Steelhead
3790B
Temperature (°C)
24
20
16
12
8
4
1APR
1MAY
1JUN
1JUL
1AUG
1SEP
1OCT
1NOV
1DEC
reach
1
2
3
4
5
6
7
8

Steelhead
3816C

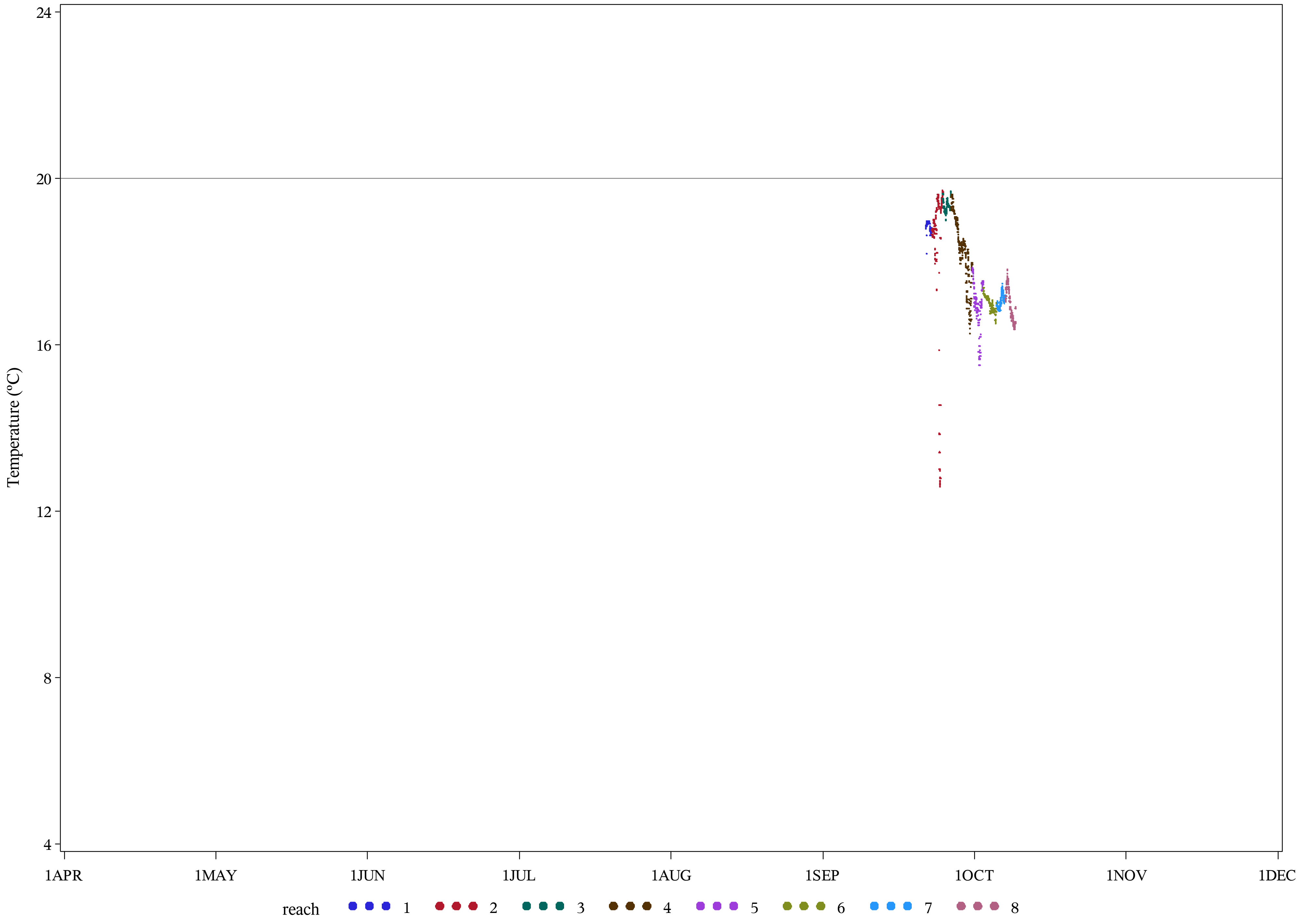

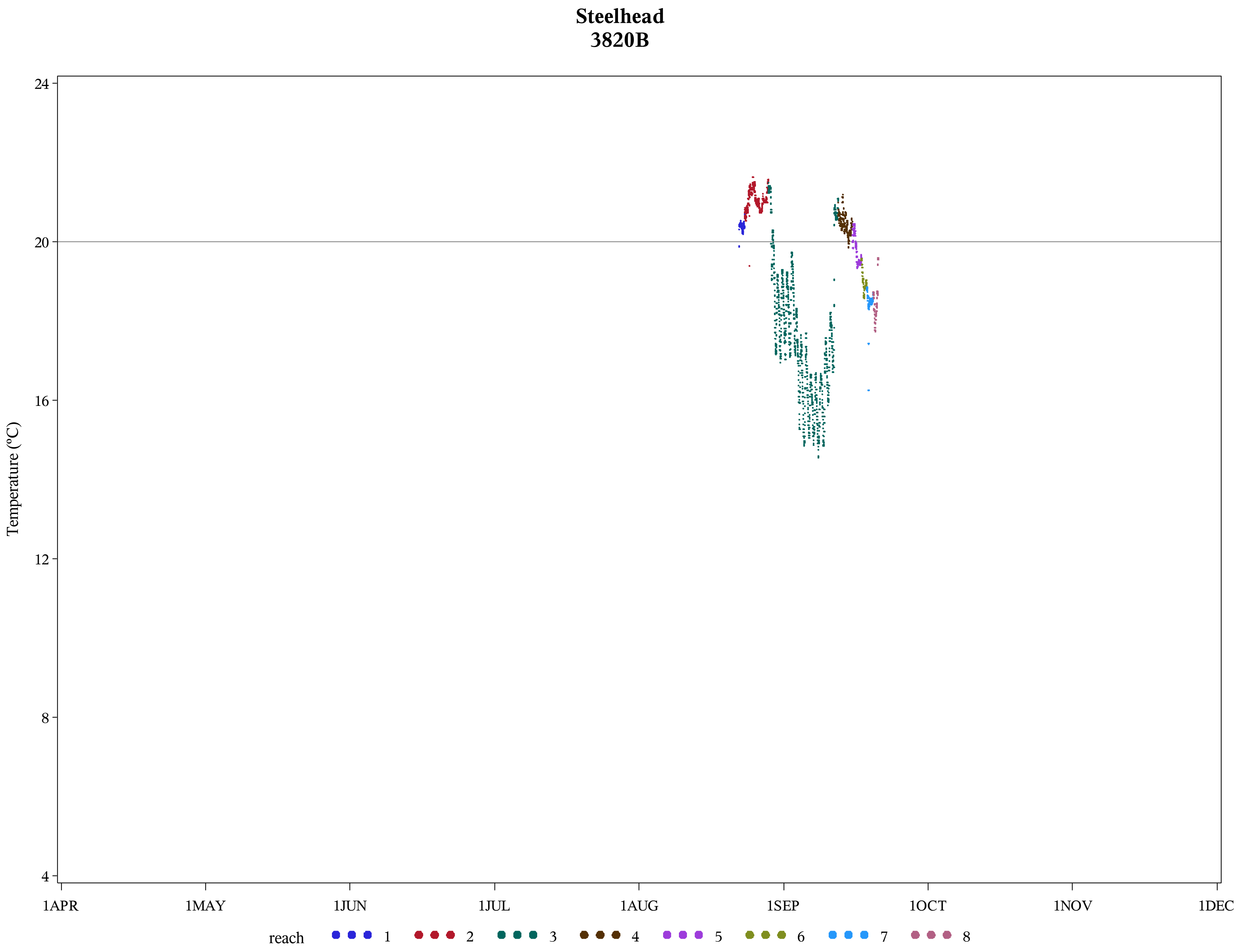

Steelhead
3820B
Temperature (°C)
24
20
16
12
8
4
1APR
1MAY
1JUN
1JUL
1AUG
1SEP
1OCT
1NOV
1DEC
reach
1
2
3
4
5
6
7
8

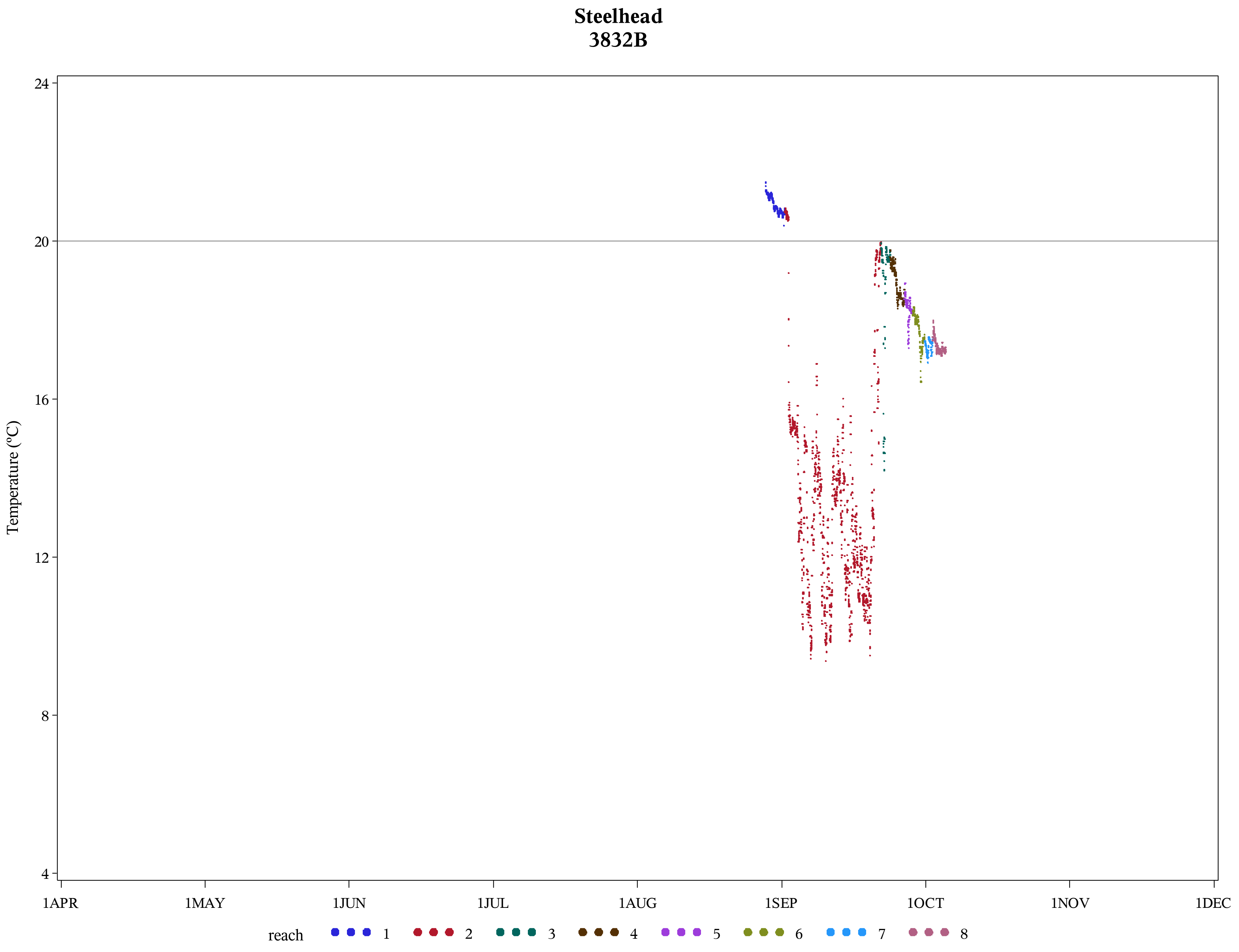

Steelhead
3832B
Temperature (°C)
24
20
16
12
8
4
1APR
1MAY
1JUN
1JUL
1AUG
1SEP
1OCT
1NOV
1DEC
reach
1
2
3
4
5
6
7
8

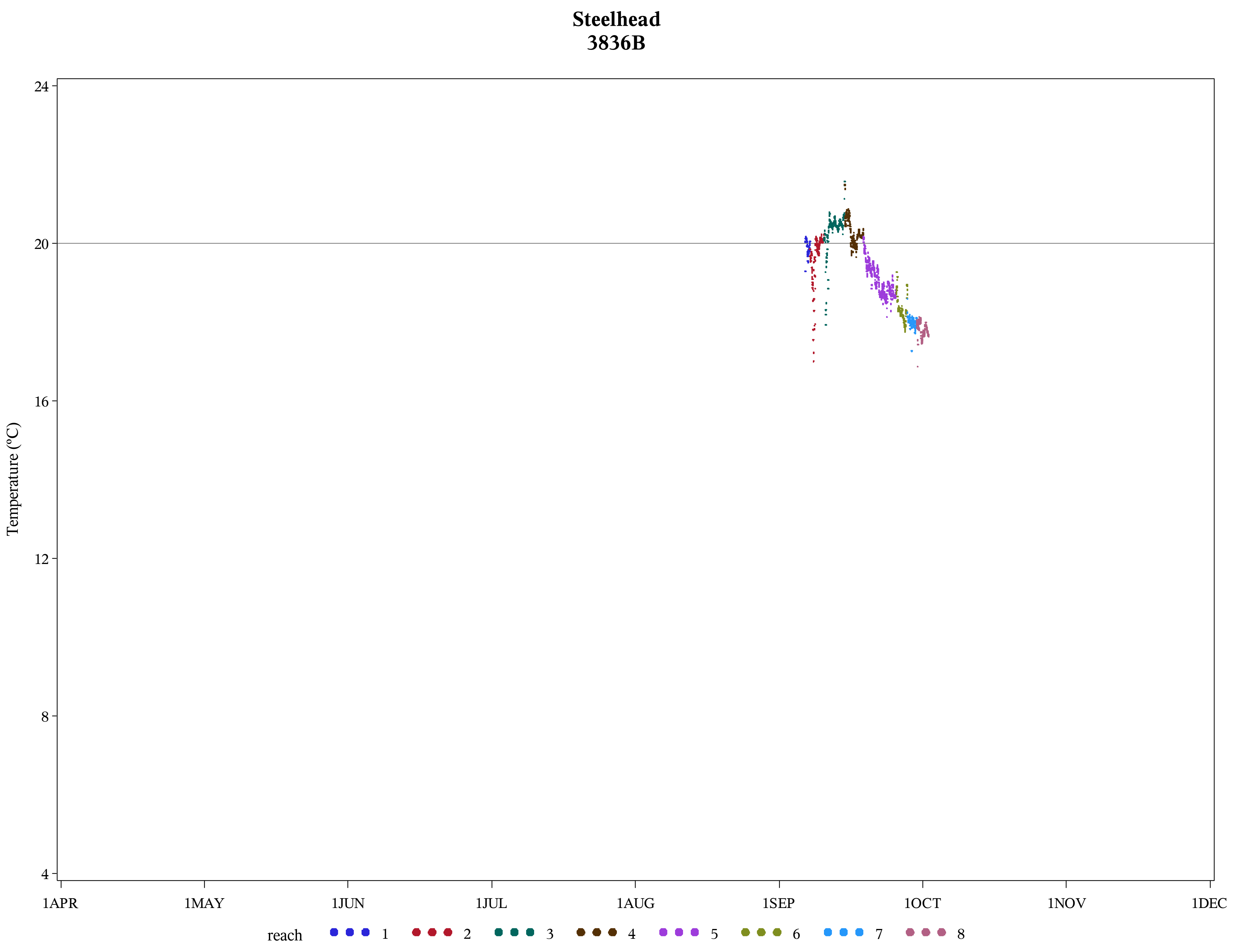

Steelhead
3836B
Temperature (°C)
24
20
16
12
8
4
1APR
1MAY
1JUN
1JUL
1AUG
1SEP
1OCT
1NOV
1DEC
reach
1
2
3
4
5
6
7
8

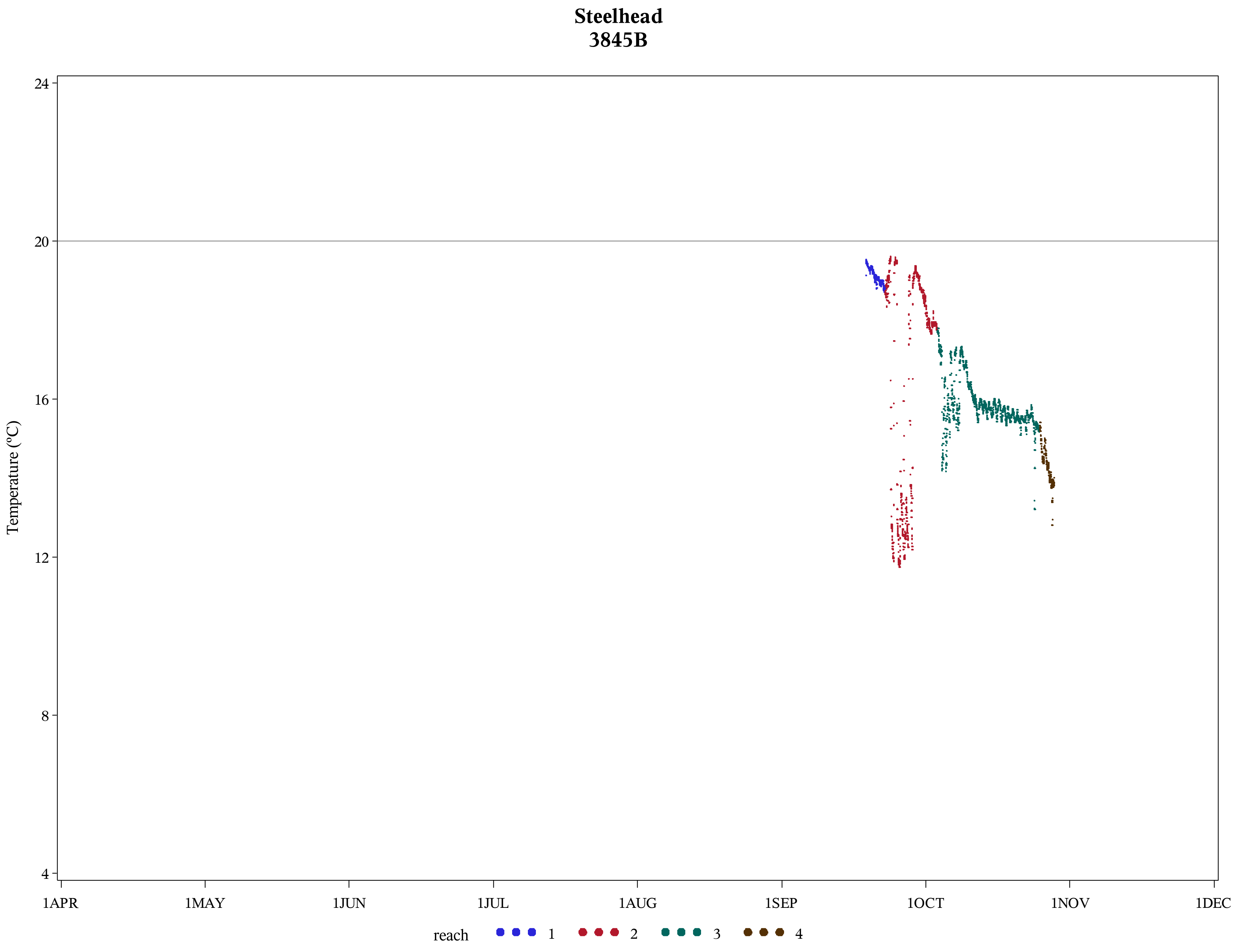
Steelhead
3845B
24
20
16
12
8
4
Temperature (°C)
1APR
1MAY
1JUN
1JUL
1AUG
1SEP
1OCT
1NOV
1DEC
reach
1
2
3
4

Steelhead
3858A

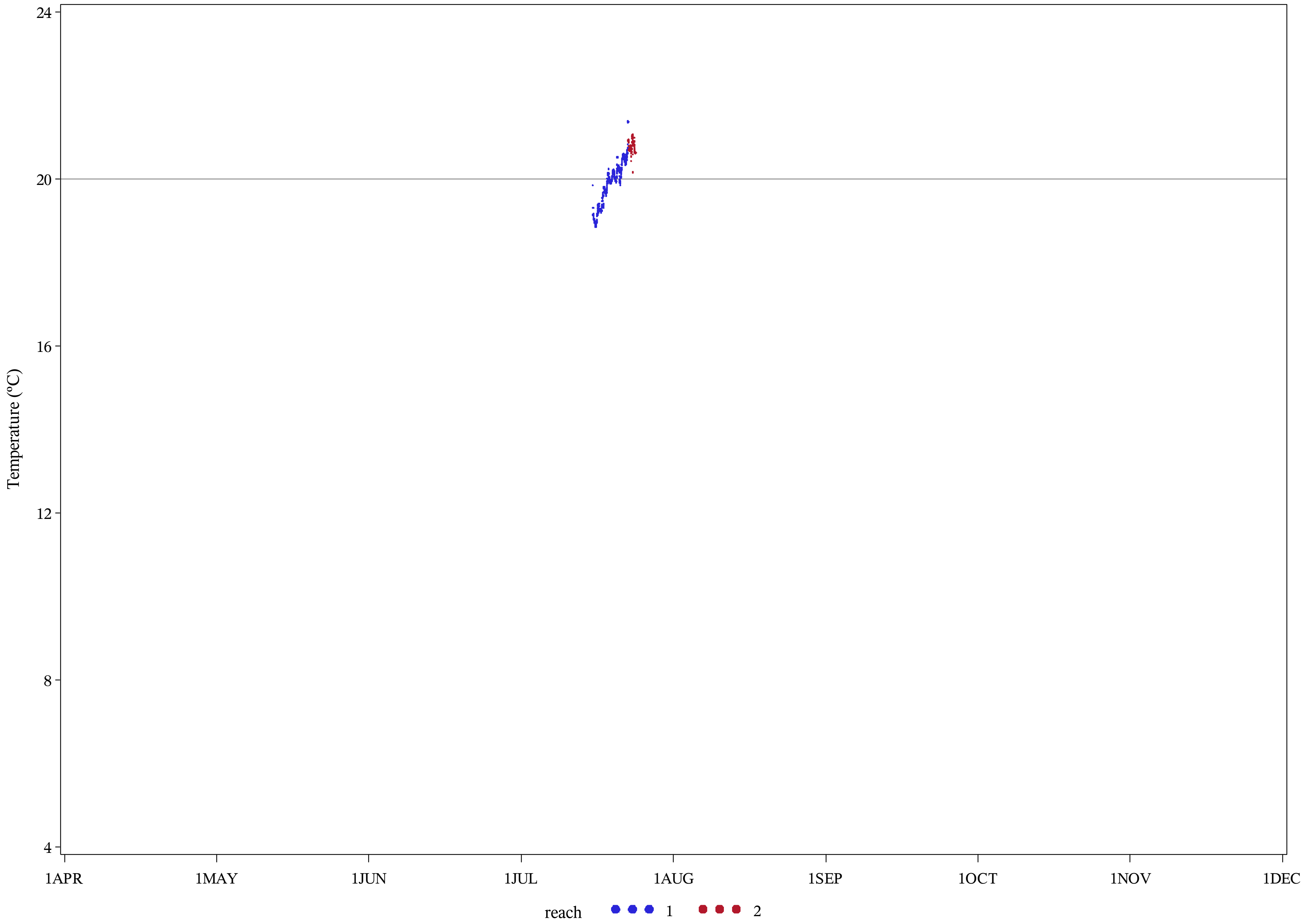

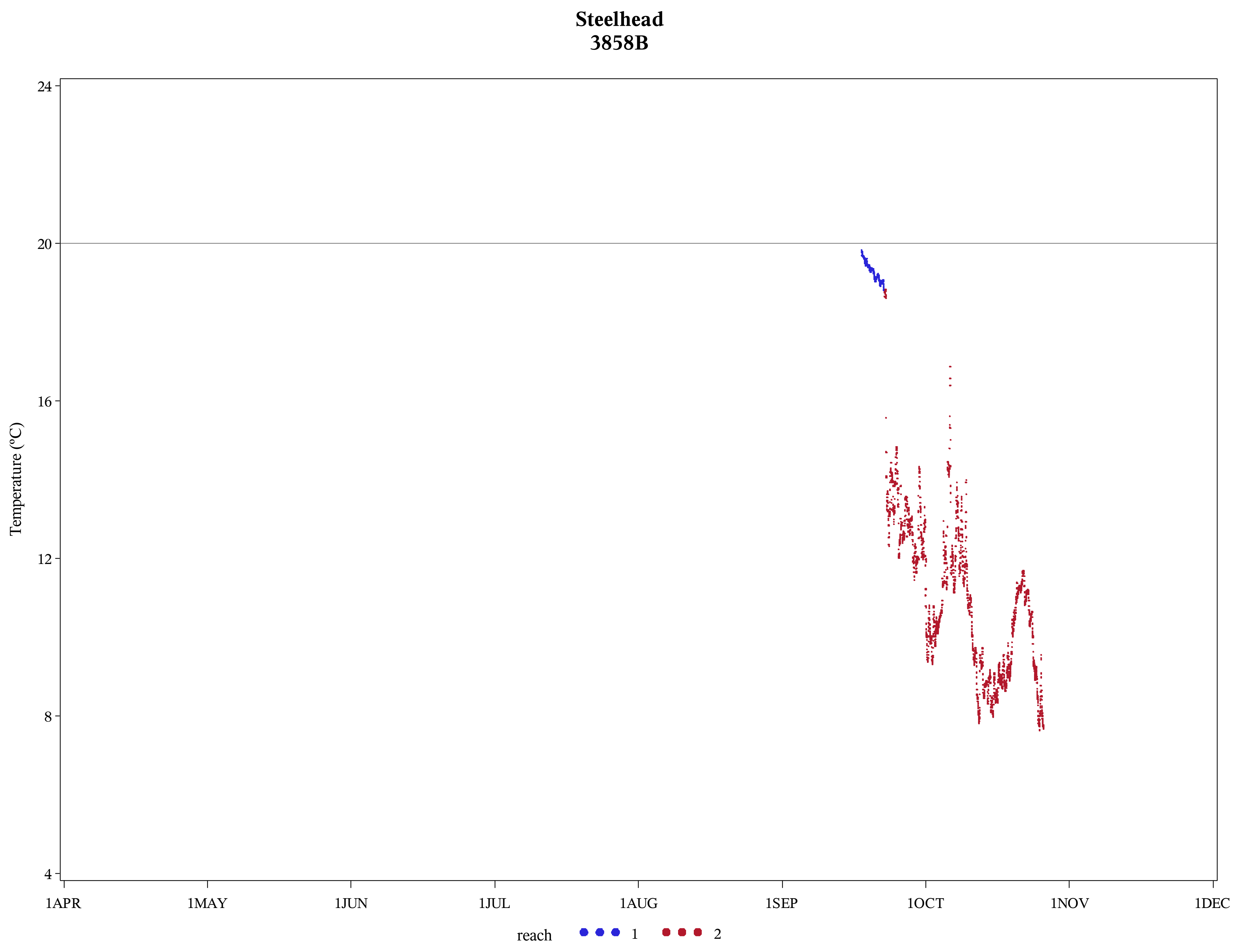

Steelhead
3858B
Temperature (°C)
24
20
16
12
8
4
1APR
1MAY
1JUN
1JUL
1AUG
1SEP
1OCT
1NOV
1DEC
reach
1
2

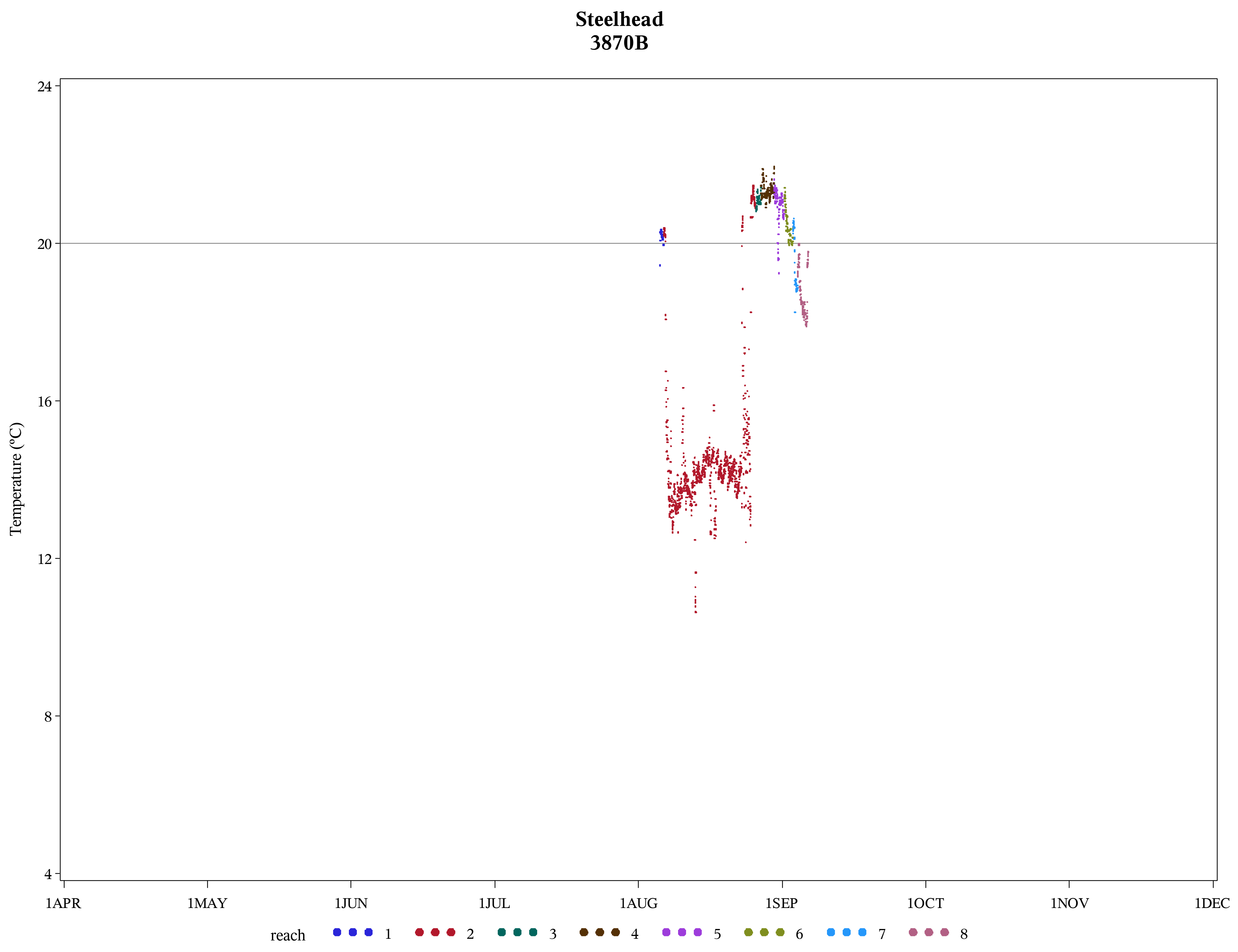

Steelhead
3870B
24
20
16
12
8
4
Temperature (°C)
1APR
1MAY
1JUN
1JUL
1AUG
1SEP
1OCT
1NOV
1DEC
reach
1
2
3
4
5
6
7
8

Steelhead
4579A

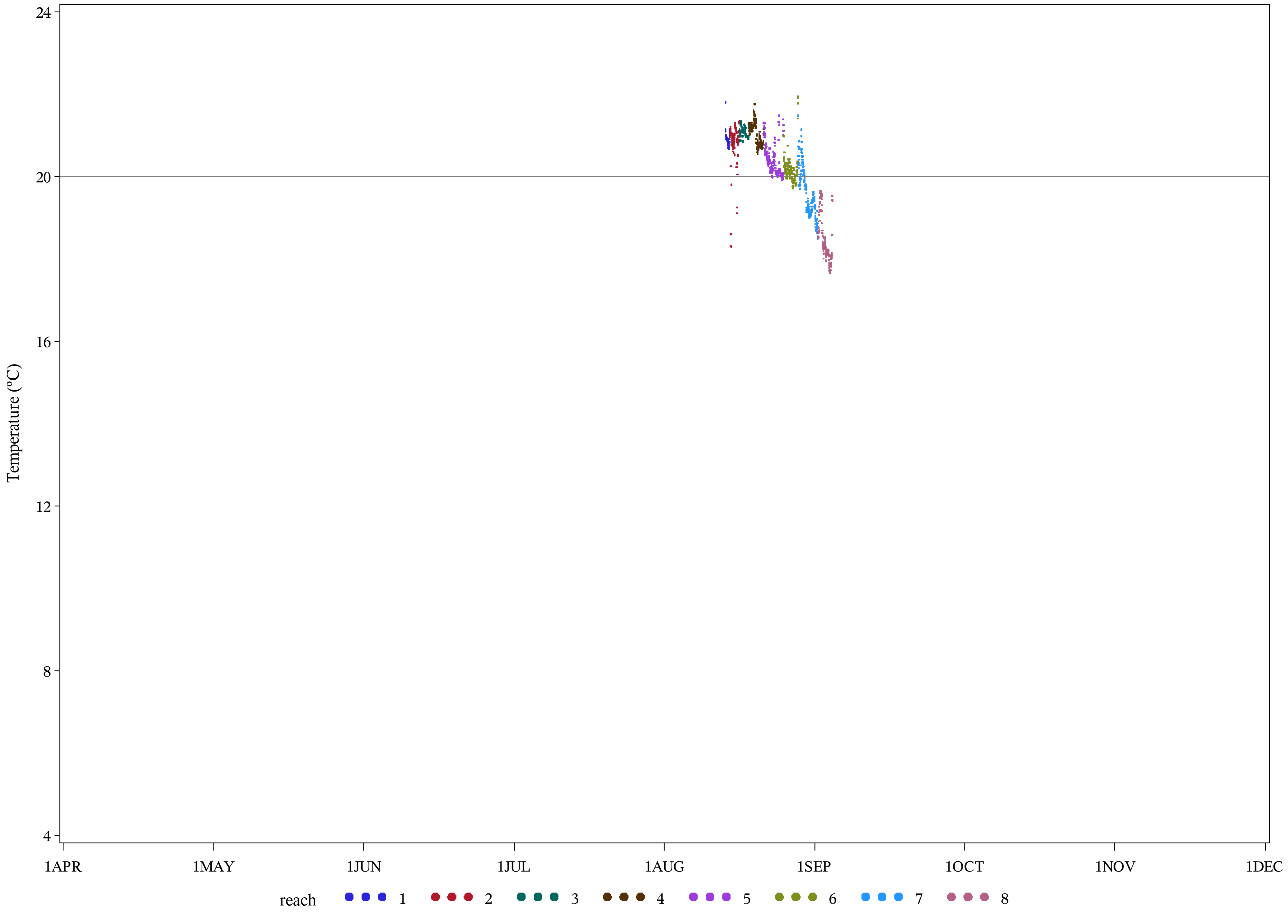

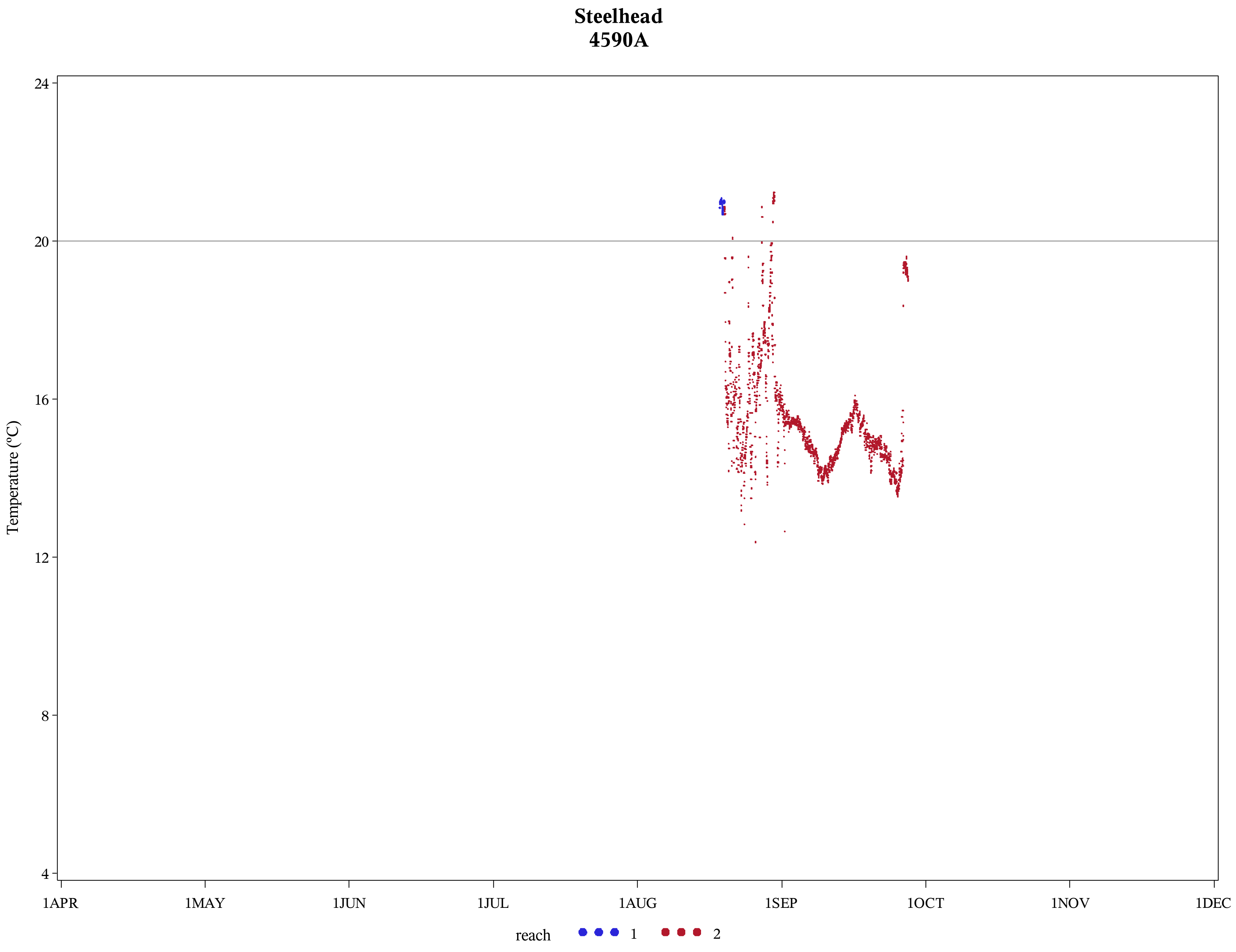

Steelhead
4590A
Temperature (°C)
24
20
16
12
8
4
1APR
1MAY
1JUN
1JUL
1AUG
1SEP
1OCT
1NOV
1DEC
reach
1
2

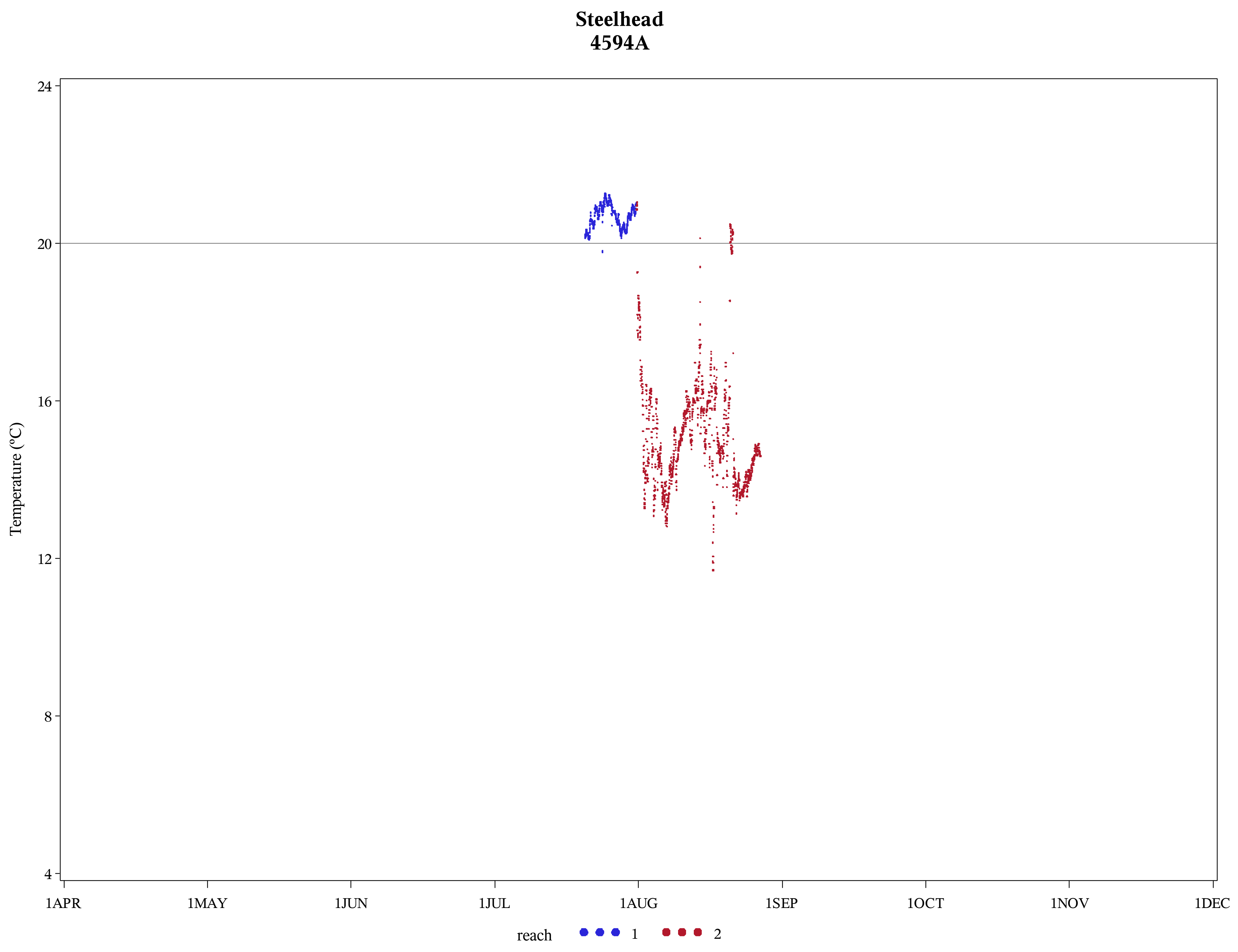
Steelhead
4594A
Temperature (°C)
24
20
16
12
8
4
1APR
1MAY
1JUN
1JUL
1AUG
1SEP
1OCT
1NOV
1DEC
reach
1
2

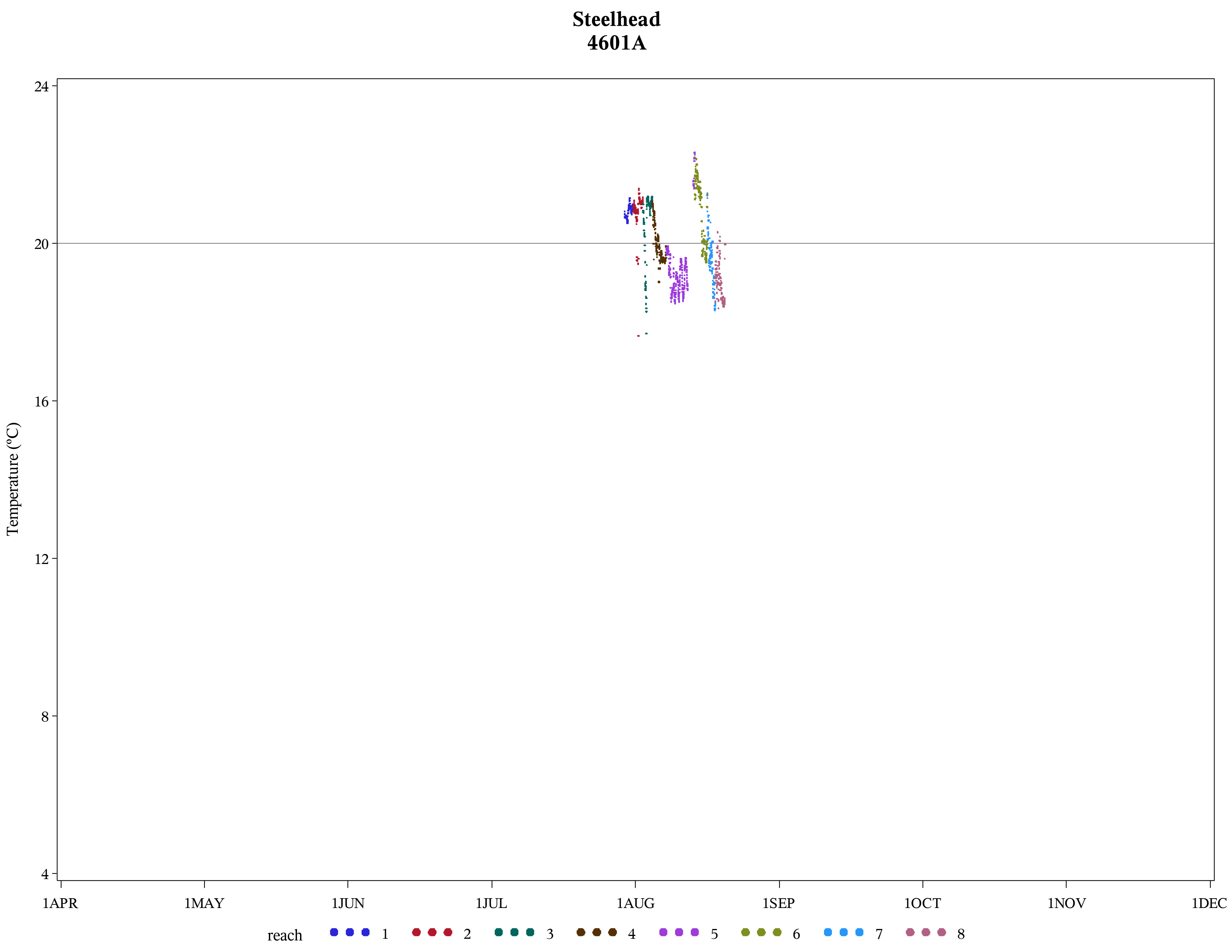
Steelhead
4601A
Temperature (°C)
24
20
16
12
8
4
1APR
1MAY
1JUN
1JUL
1AUG
1SEP
1OCT
1NOV
1DEC
reach
1
2
3
4
5
6
7
8

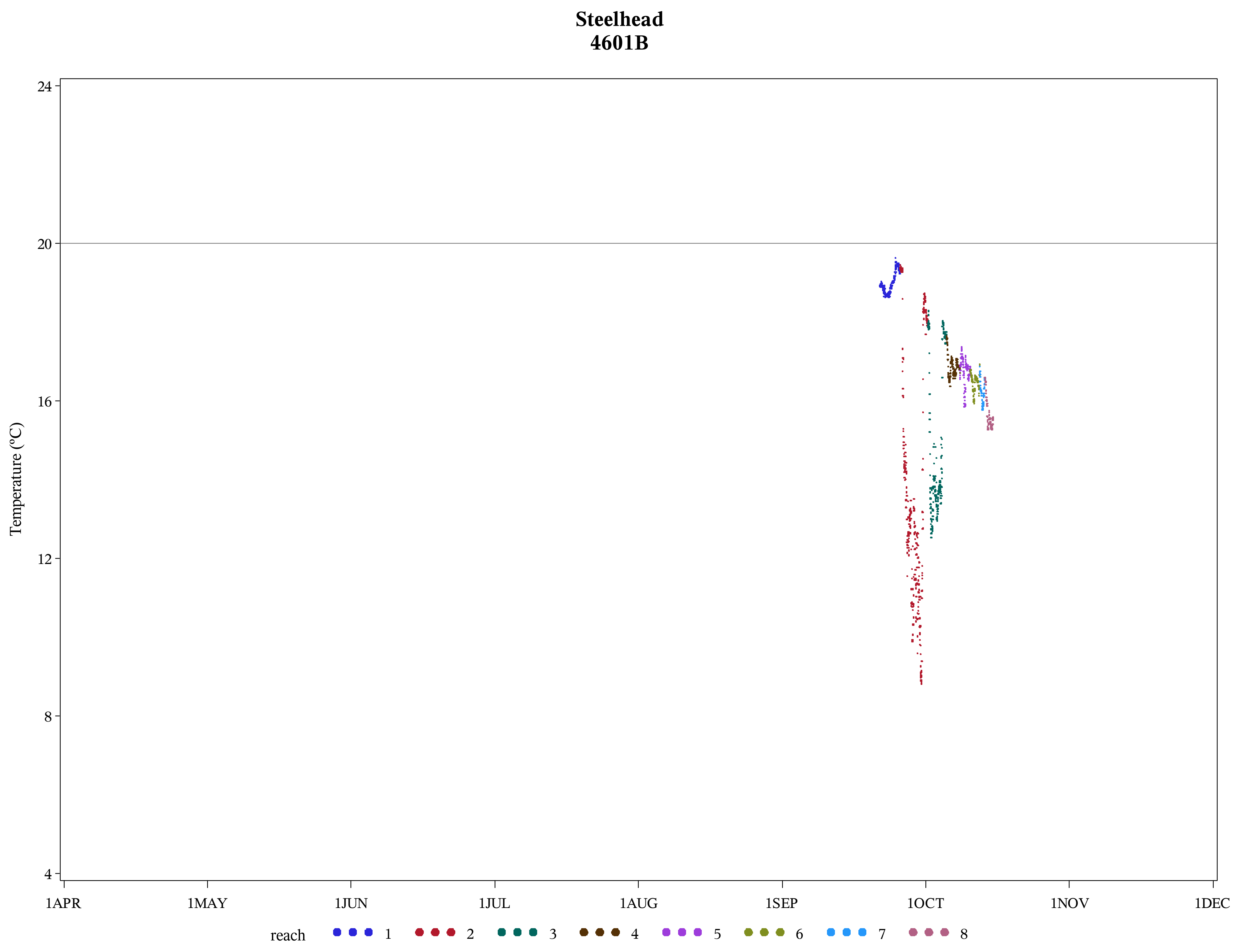
Steelhead
4601B
Temperature (°C)
24
20
16
12
8
4
1APR
1MAY
1JUN
1JUL
1AUG
1SEP
1OCT
1NOV
1DEC
reach
1
2
3
4
5
6
7
8

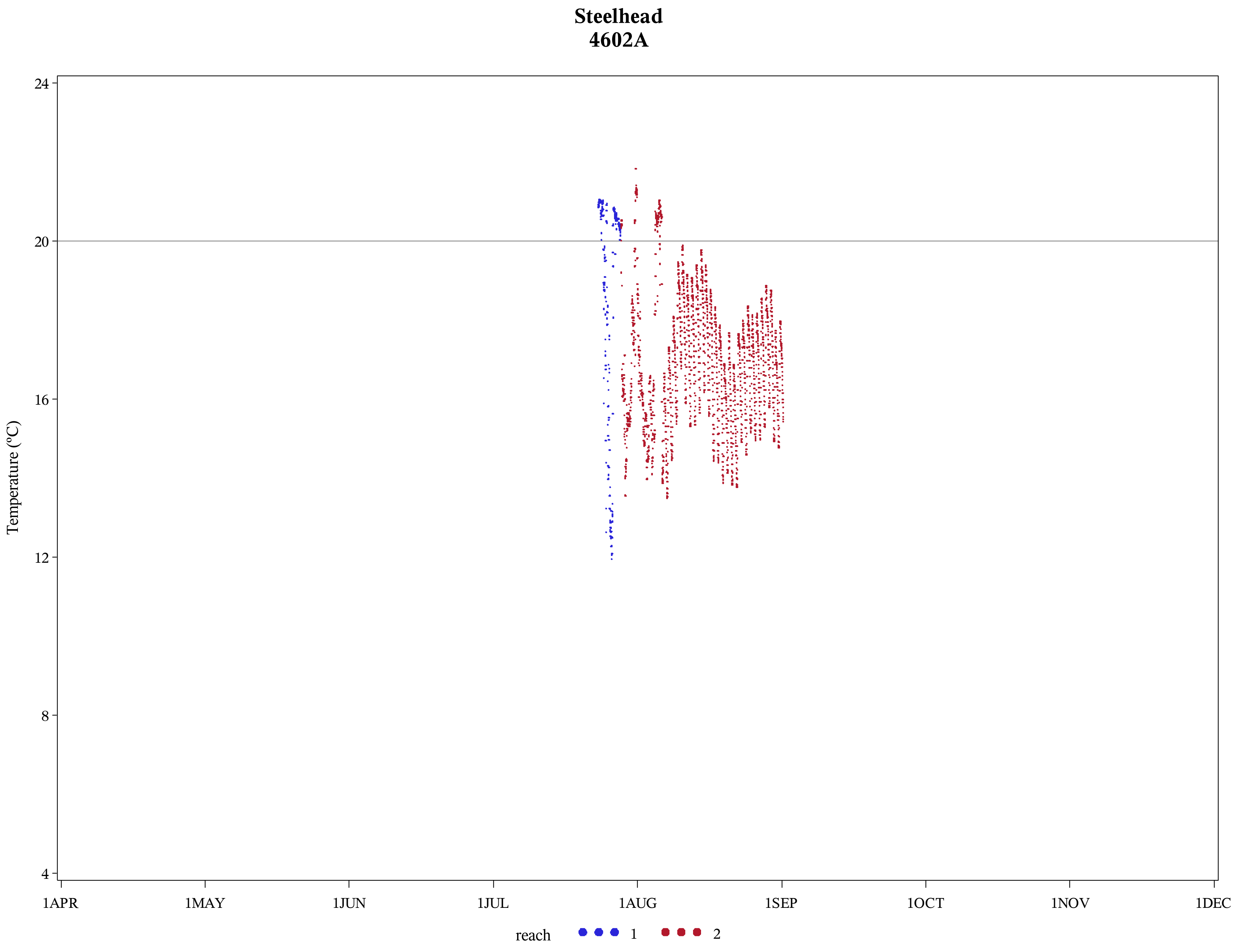

Steelhead
4602A
Temperature (°C)
24
20
16
12
8
4
1APR
1MAY
1JUN
1JUL
1AUG
1SEP
1OCT
1NOV
1DEC
reach
1
2

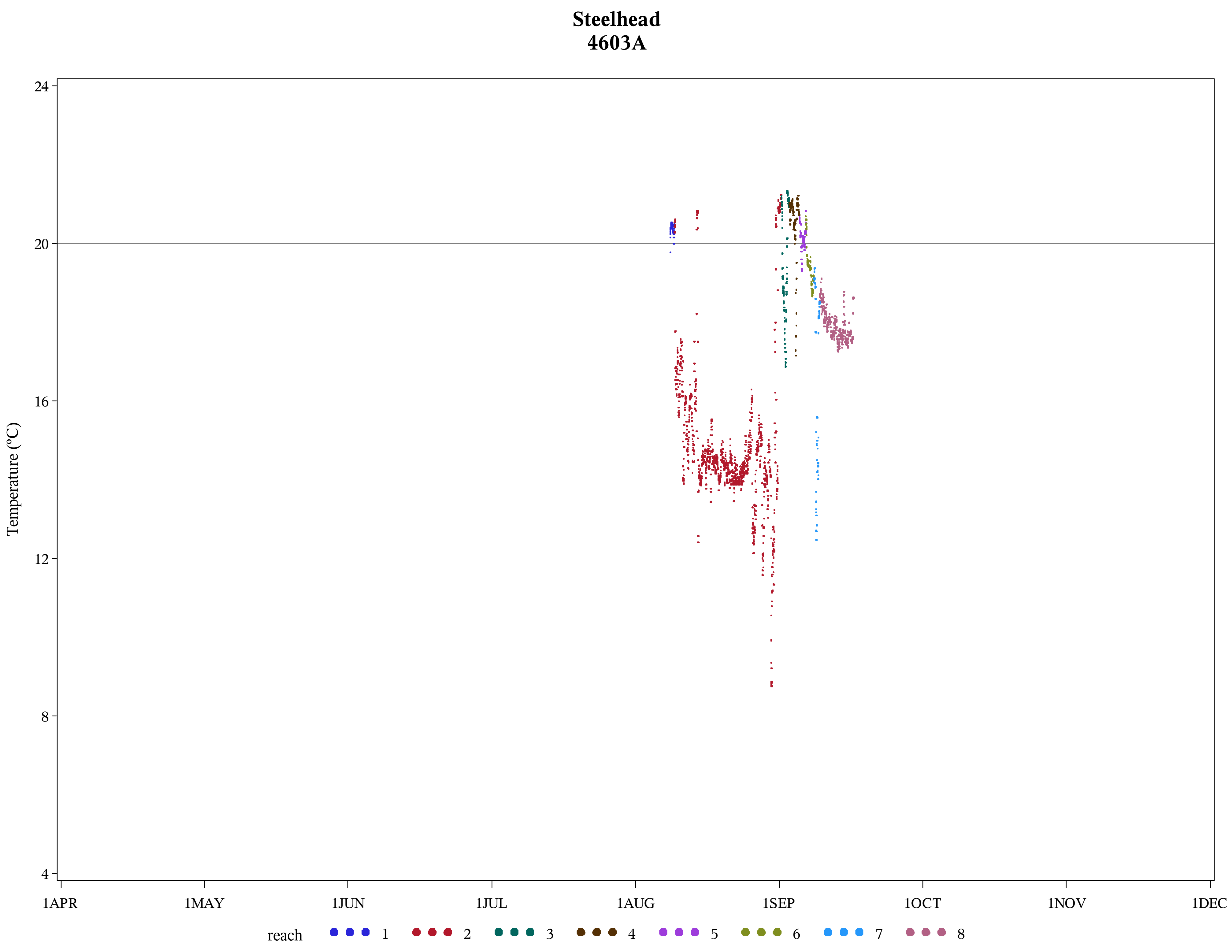

Steelhead
4603A
Temperature (°C)
24
20
16
12
8
4
1APR
1MAY
1JUN
1JUL
1AUG
1SEP
1OCT
1NOV
1DEC
reach
1
2
3
4
5
6
7
8

Steelhead
4607A

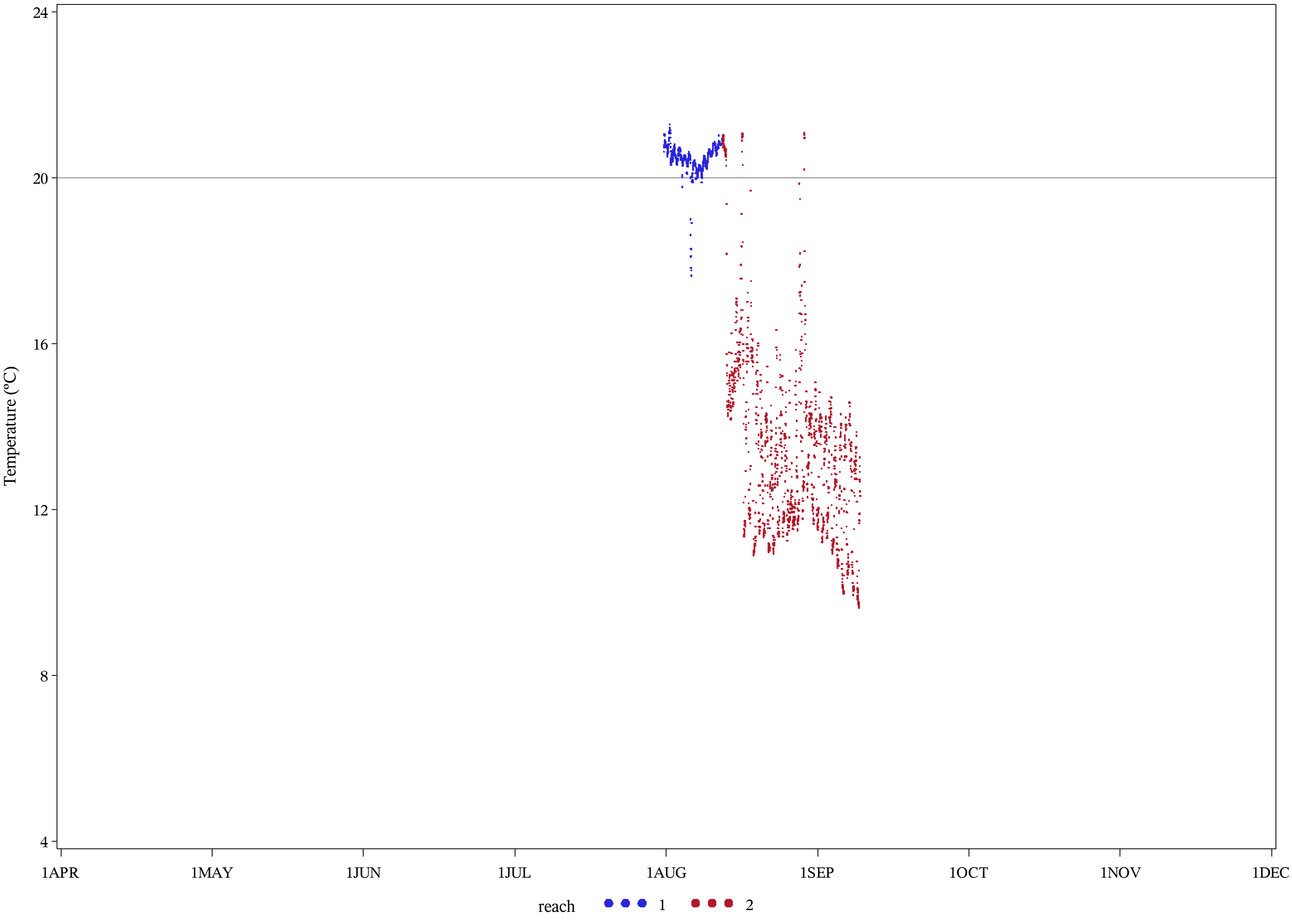

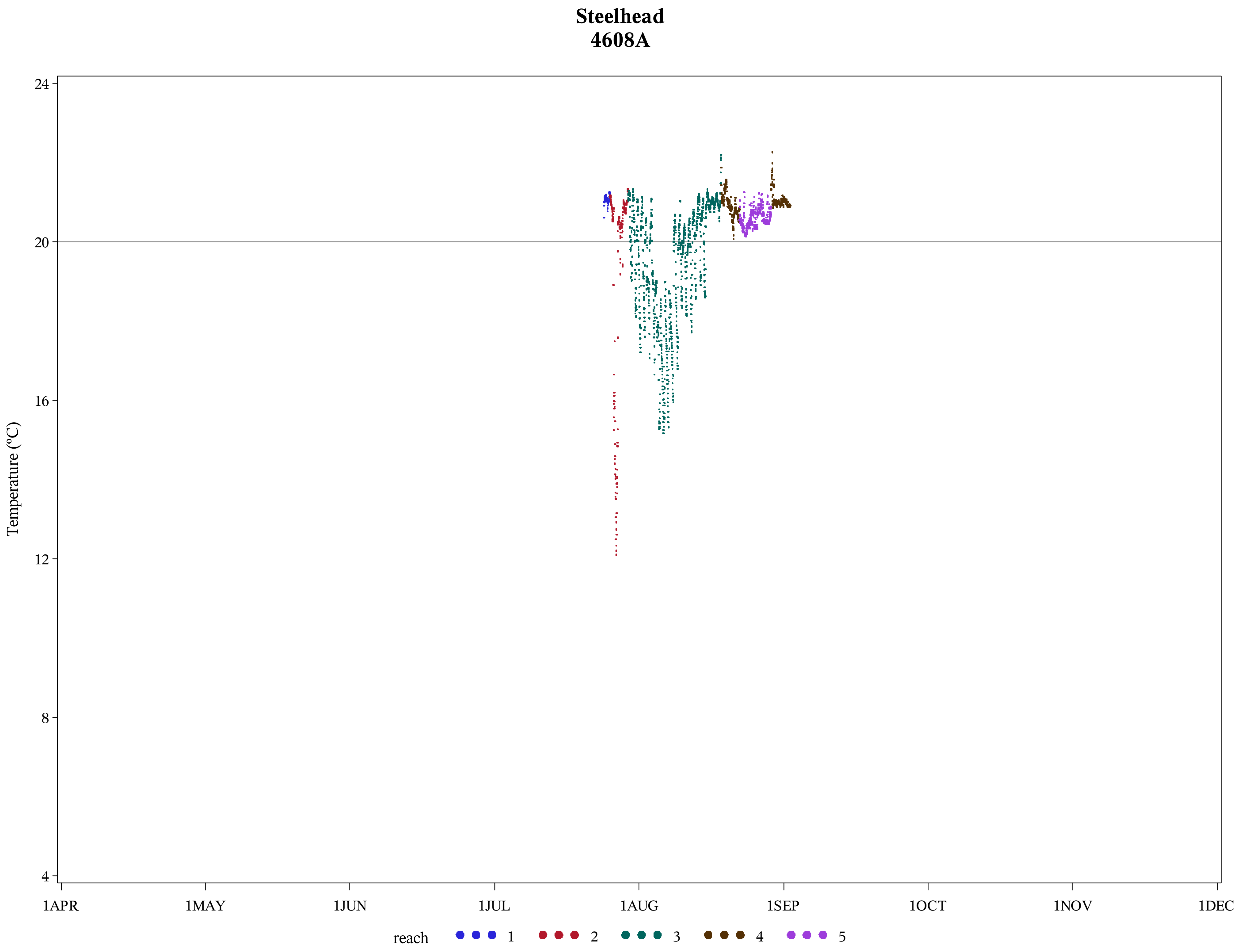

Steelhead
4608A
Temperature (°C)
24
20
16
12
8
4
1APR
1MAY
1JUN
1JUL
1AUG
1SEP
1OCT
1NOV
1DEC
reach
1
2
3
4
5

Steelhead
4610A

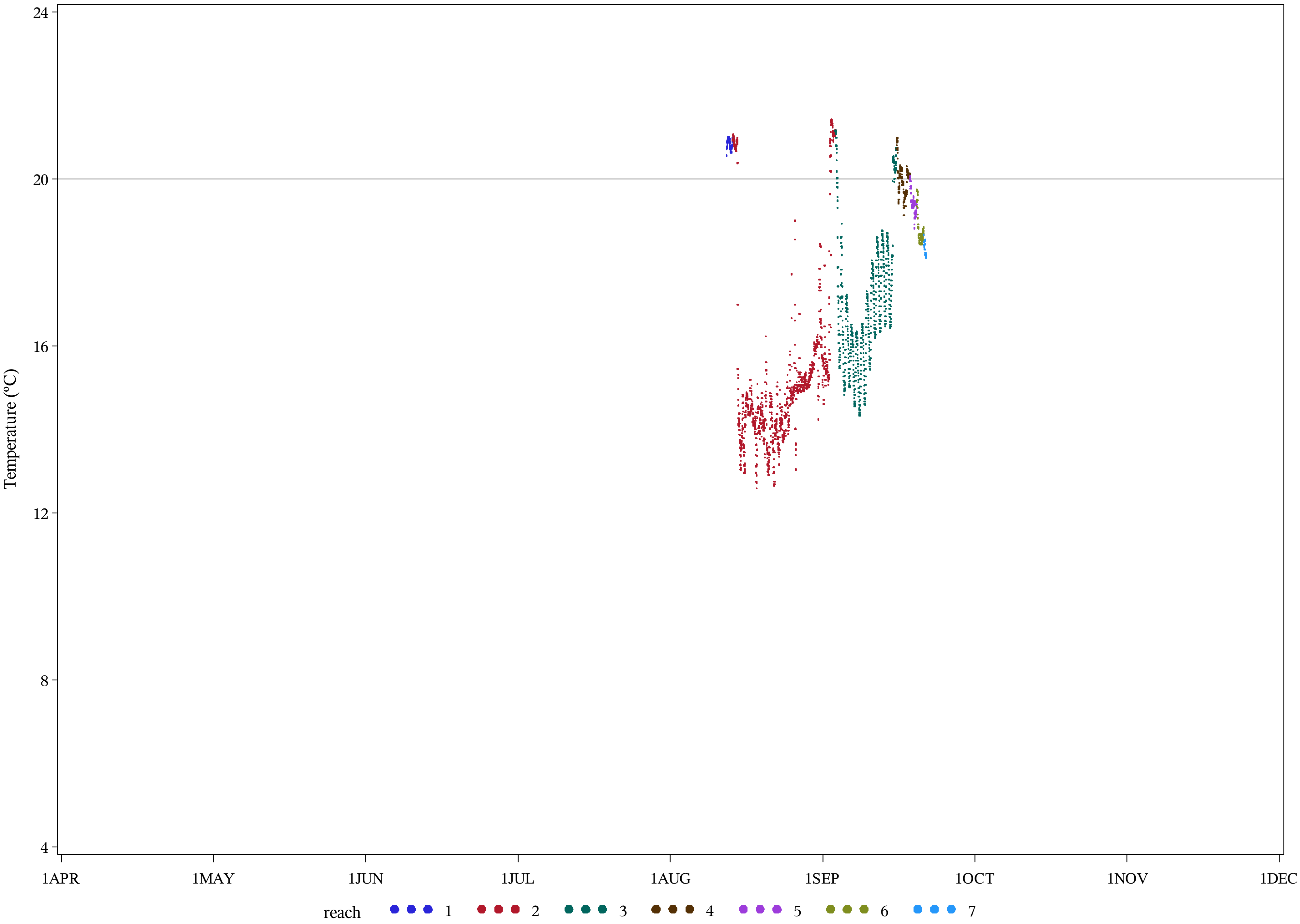

Steelhead
4611A

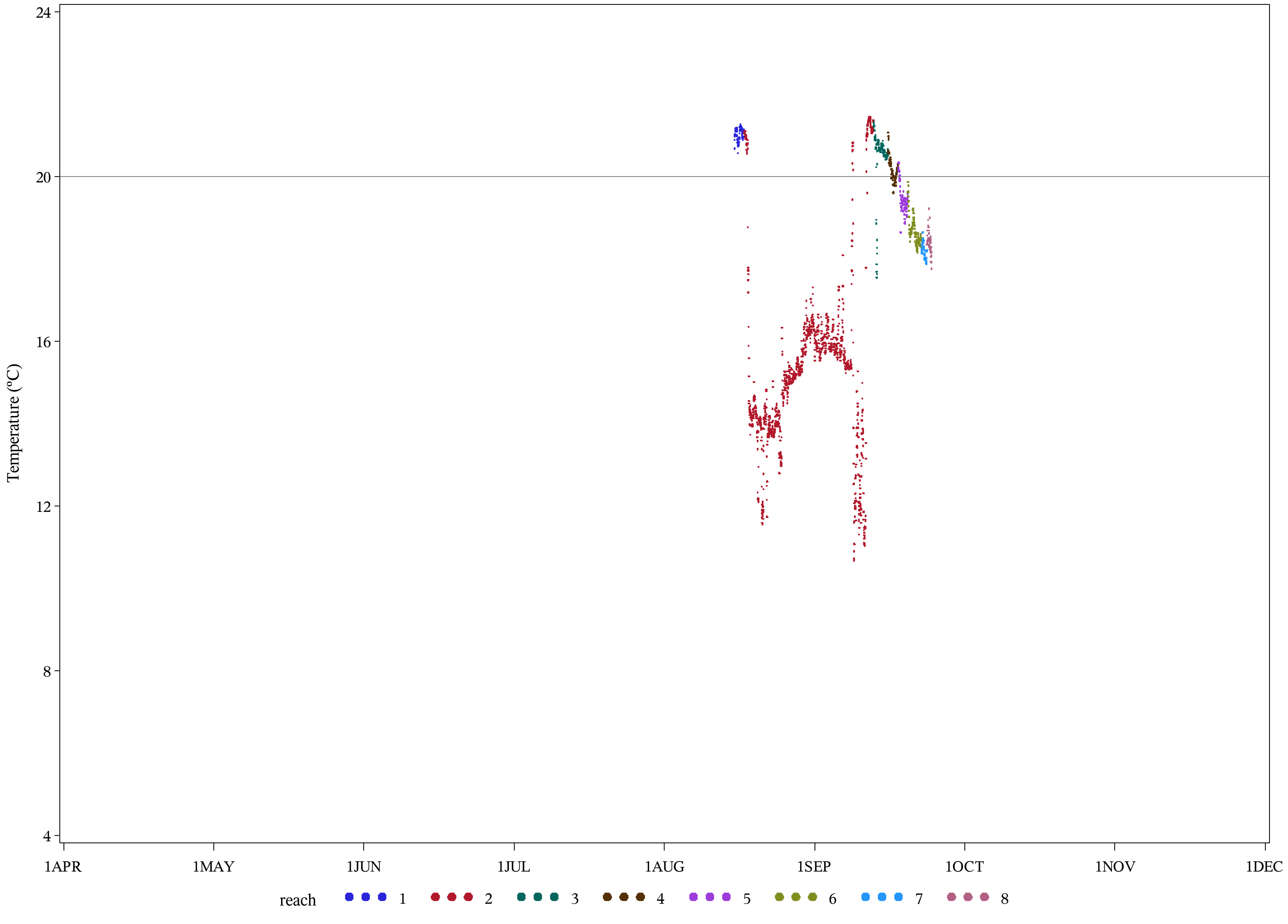

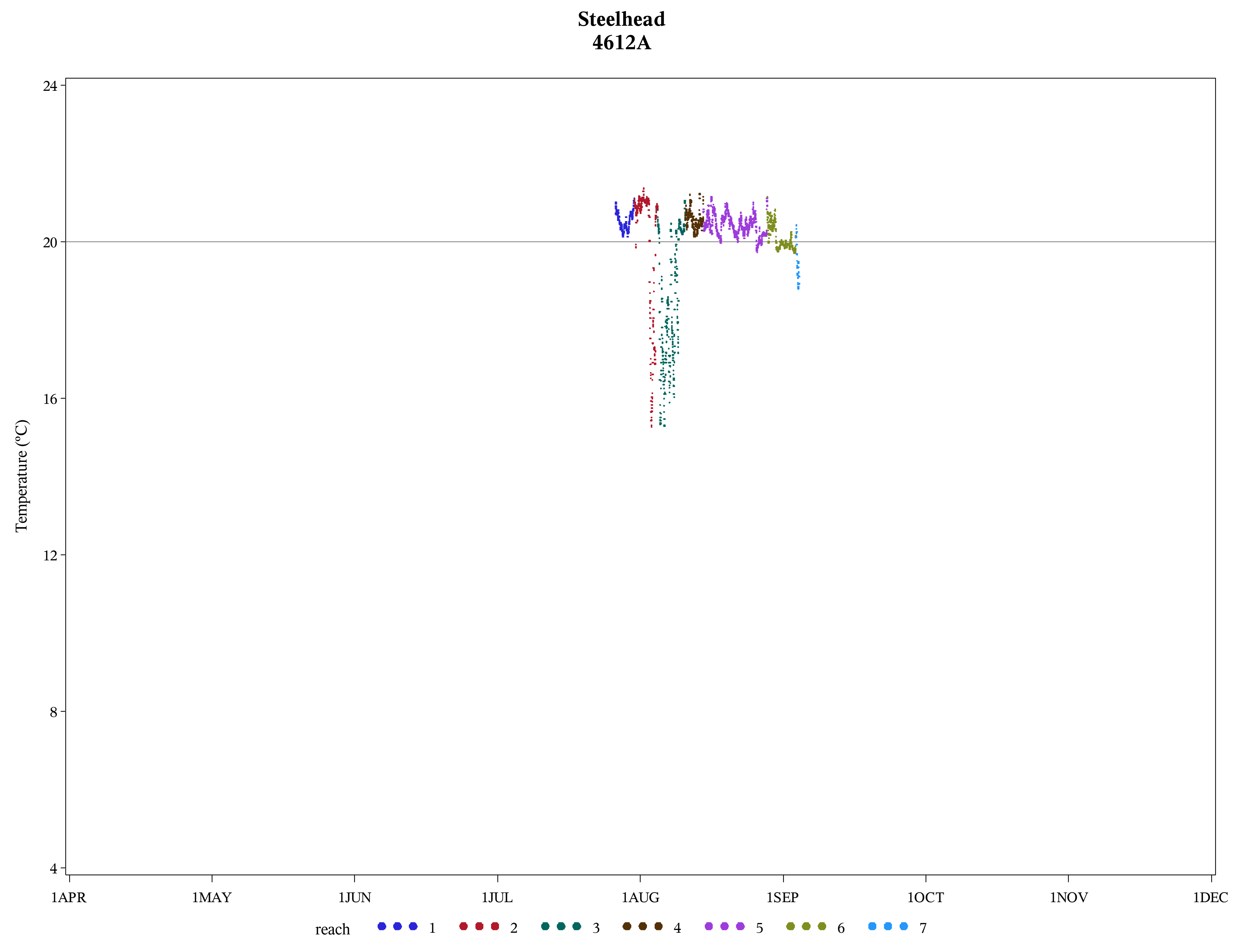
Steelhead
4612A
Temperature (°C)
24
20
16
12
8
4
1APR
1MAY
1JUN
1JUL
1AUG
1SEP
1OCT
1NOV
1DEC
reach
1
2
3
4
5
6
7

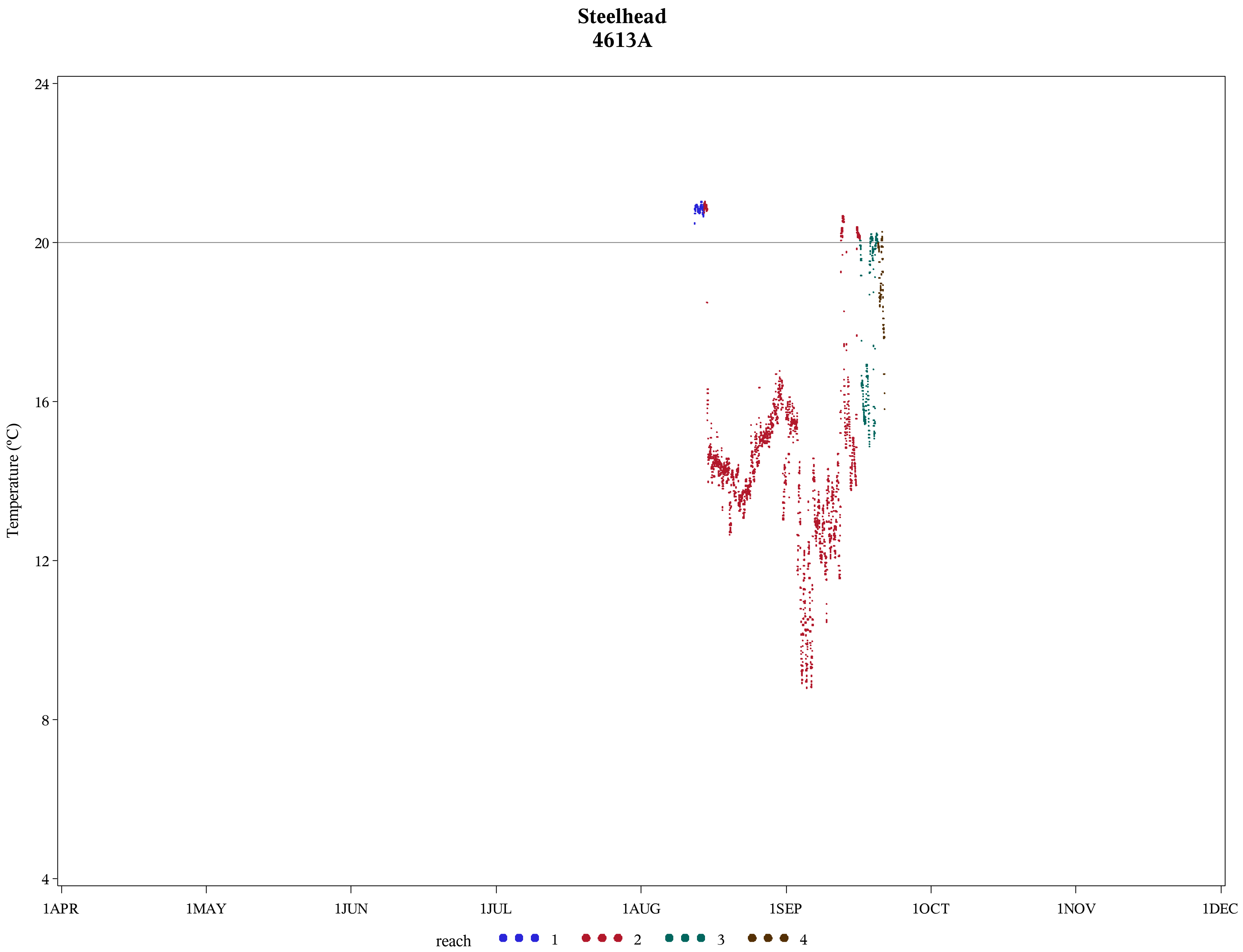
Steelhead
4613A
Temperature (°C)
24
20
16
12
8
4
1APR
1MAY
1JUN
1JUL
1AUG
1SEP
1OCT
1NOV
1DEC
reach
1
2
3
4

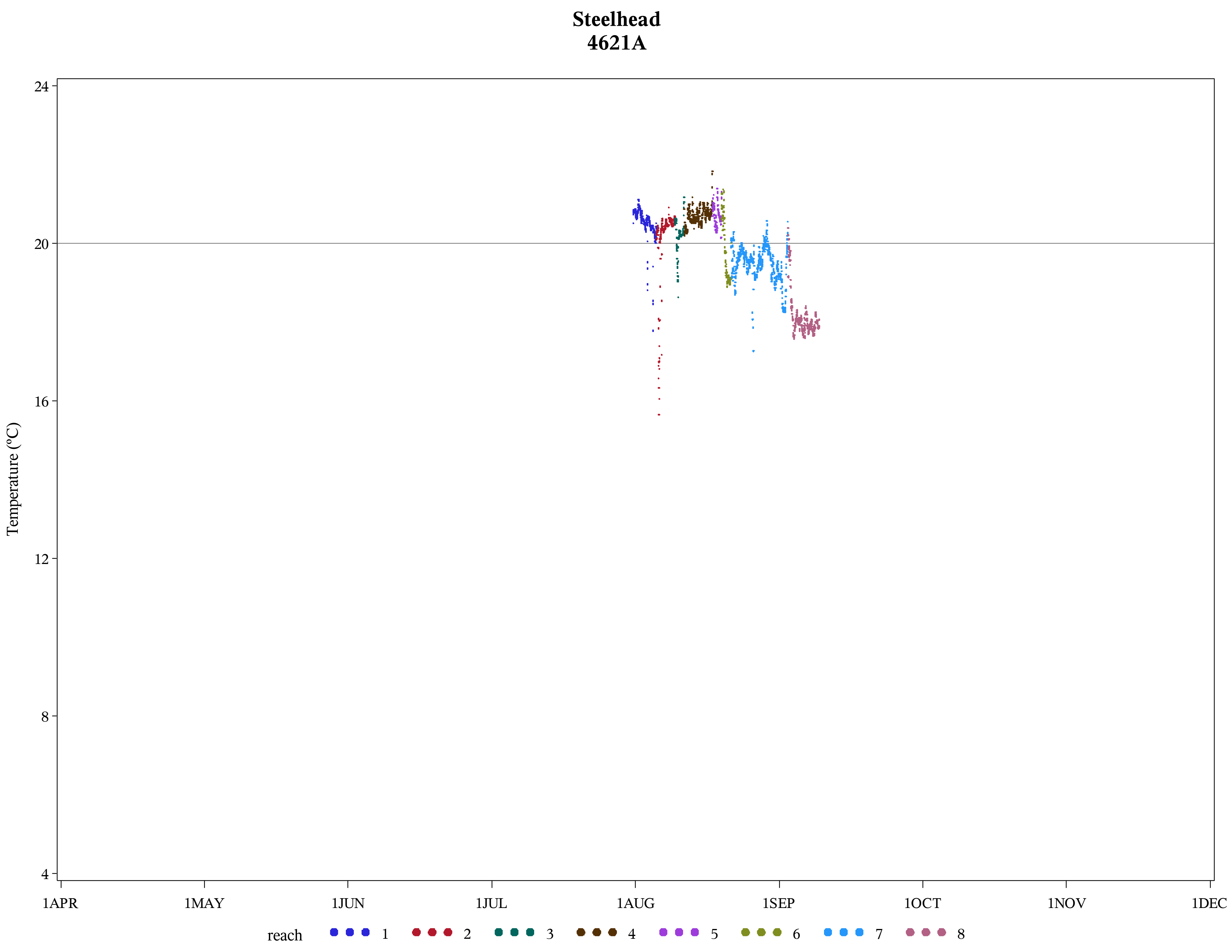

Steelhead
4621A
Temperature (°C)
24
20
16
12
8
4
1APR
1MAY
1JUN
1JUL
1AUG
1SEP
1OCT
1NOV
1DEC
reach
1
2
3
4
5
6
7
8

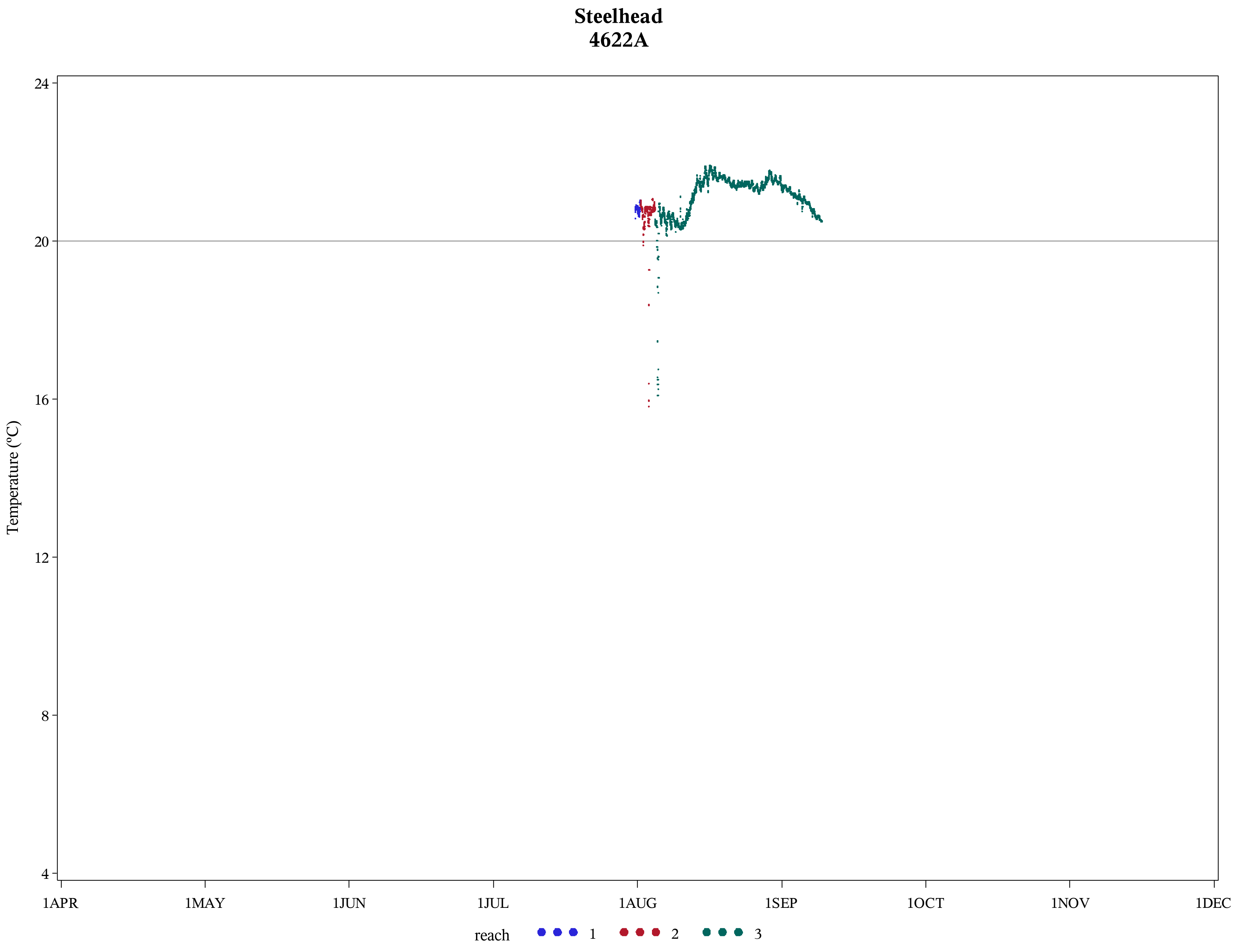
Steelhead
4622A
Temperature (°C)
24
20
16
12
8
4
1APR
1MAY
1JUN
1JUL
1AUG
1SEP
1OCT
1NOV
1DEC
reach
1
2
3

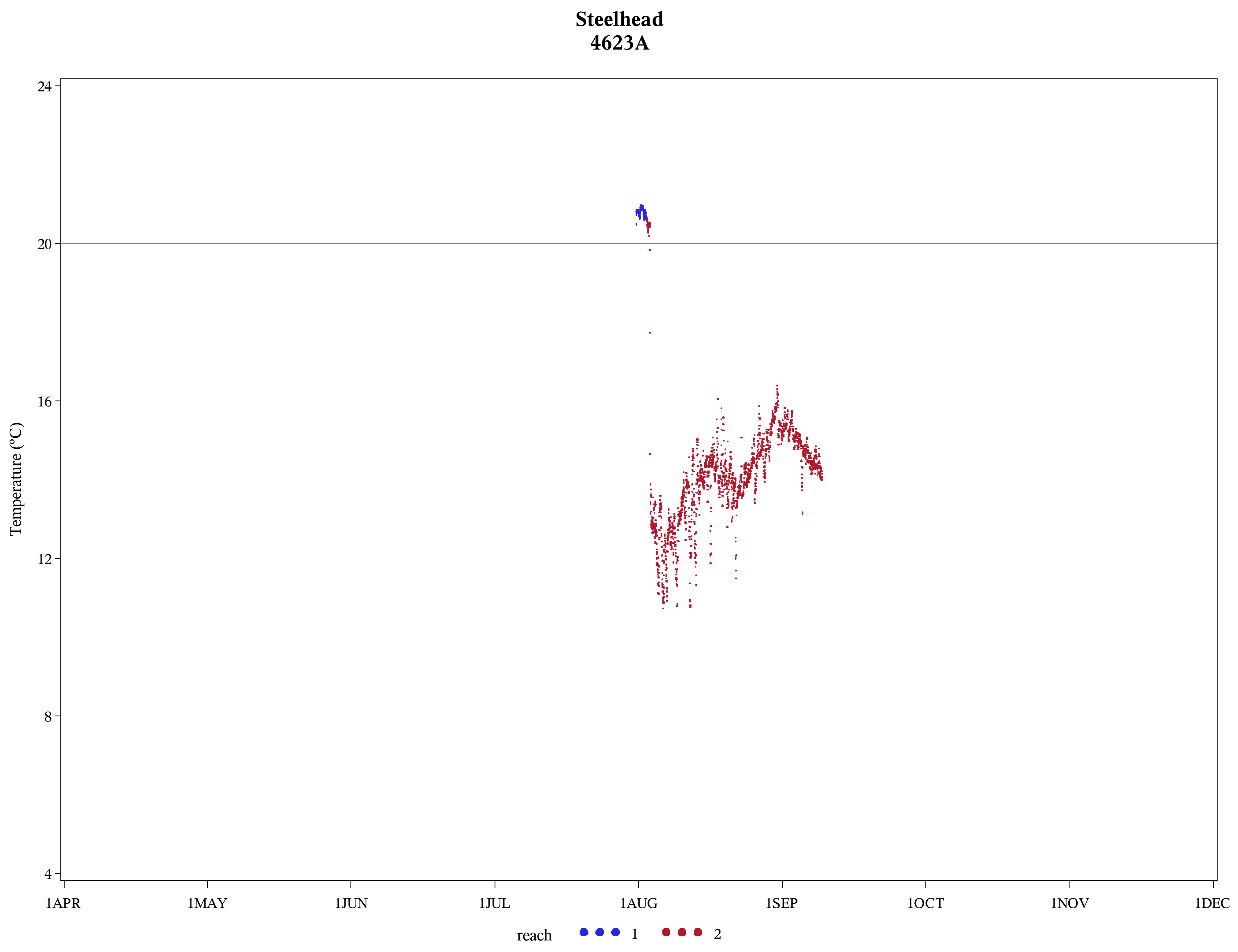
Steelhead
4623A
Temperature (°C)
24
20
16
12
8
4
1APR
1MAY
1JUN
1JUL
1AUG
1SEP
1OCT
1NOV
1DEC
reach
1
2

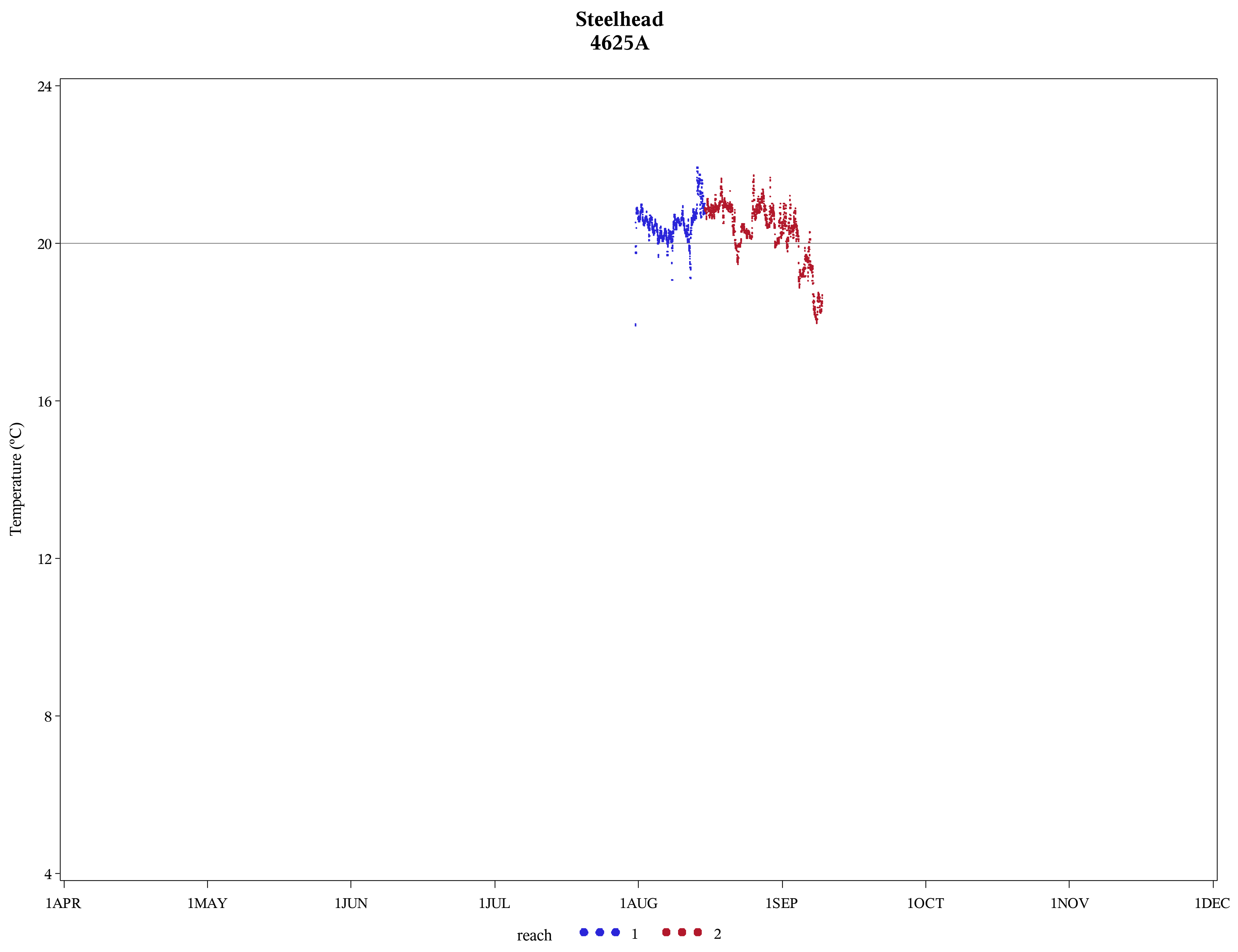
Steelhead
4625A
24
20
16
12
8
4
Temperature (°C)
1APR
1MAY
1JUN
1JUL
1AUG
1SEP
1OCT
1NOV
1DEC
reach
1
2

Steelhead
4626A

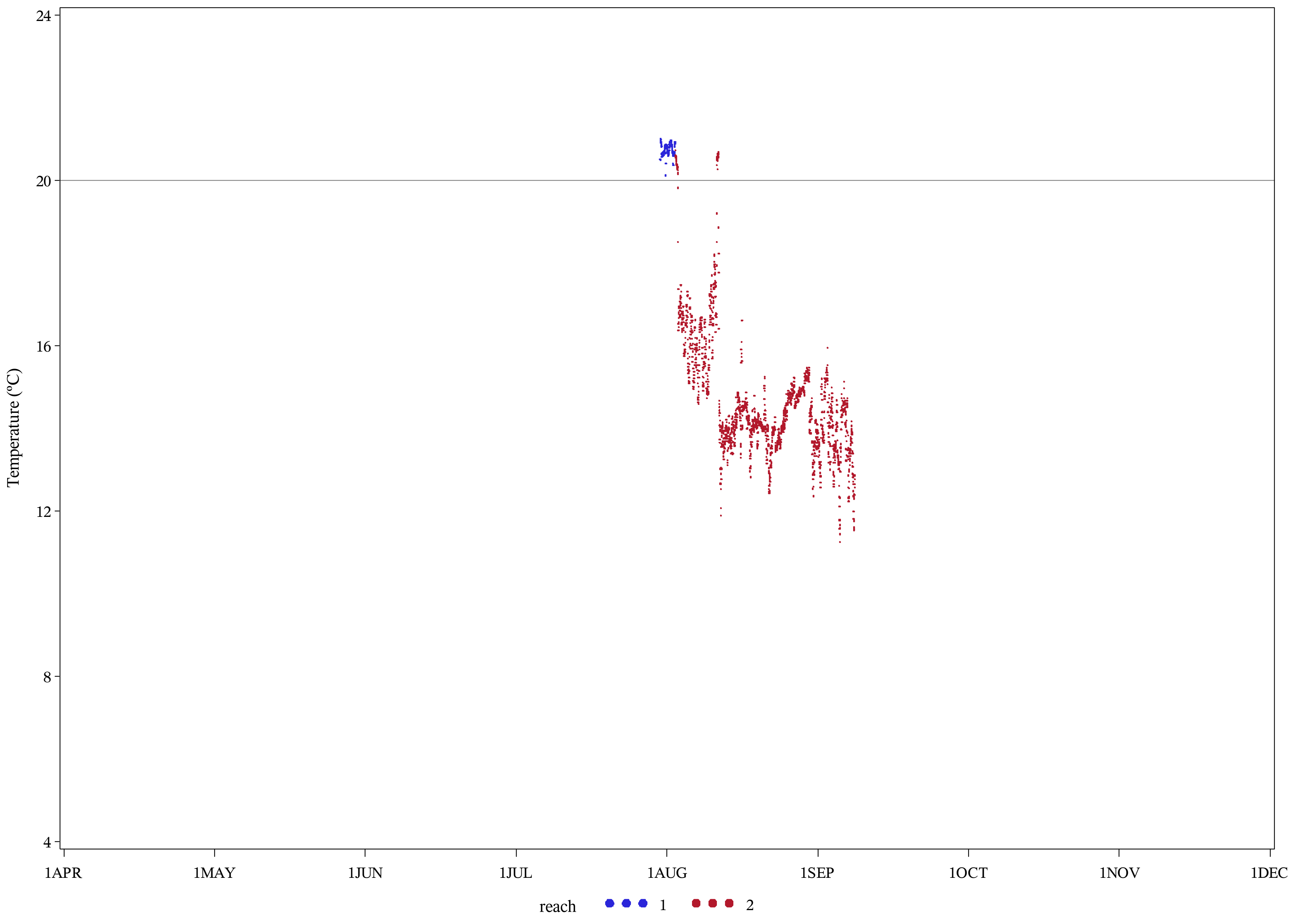

Steelhead
4627A

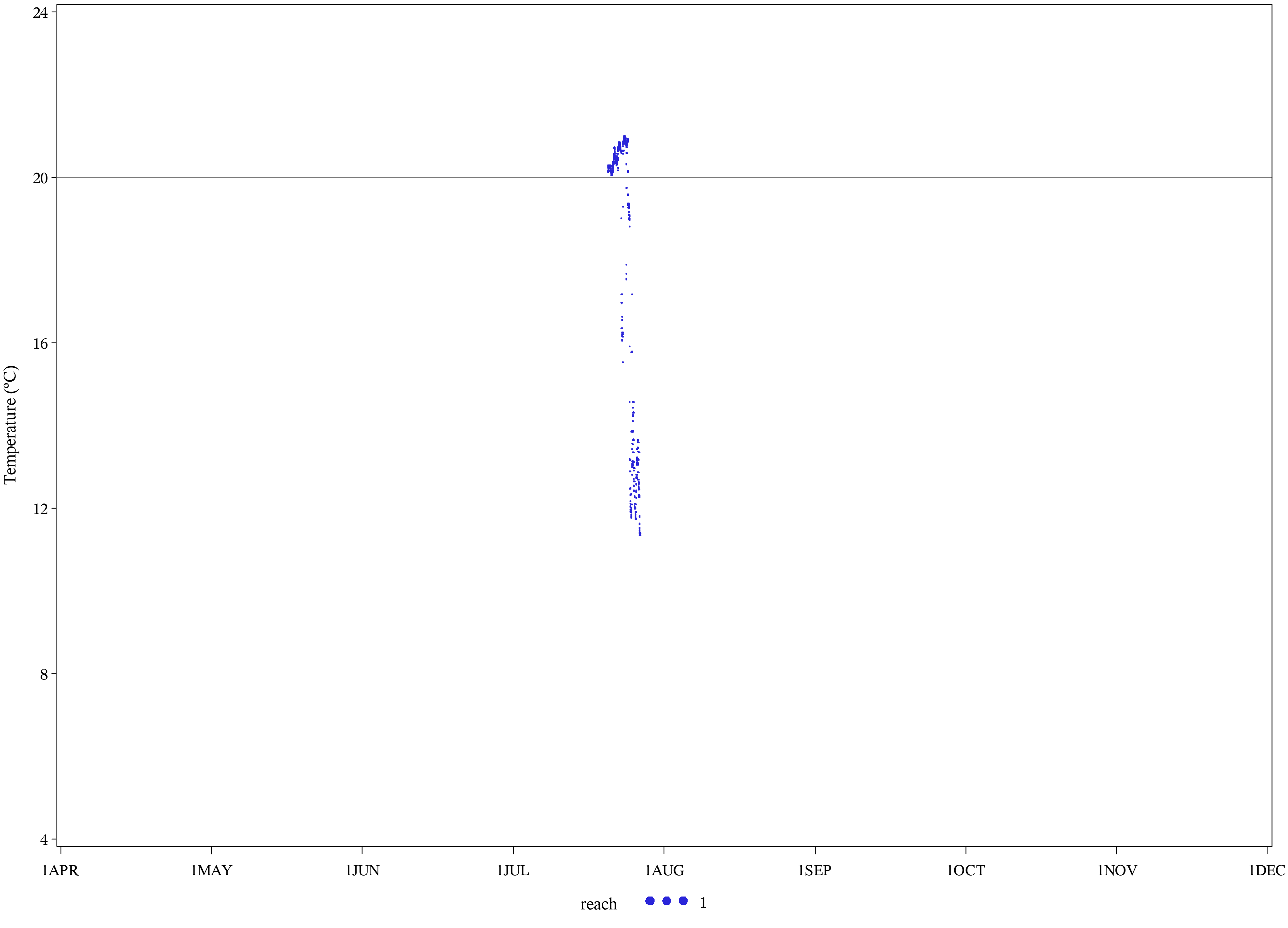

Steelhead
4629A

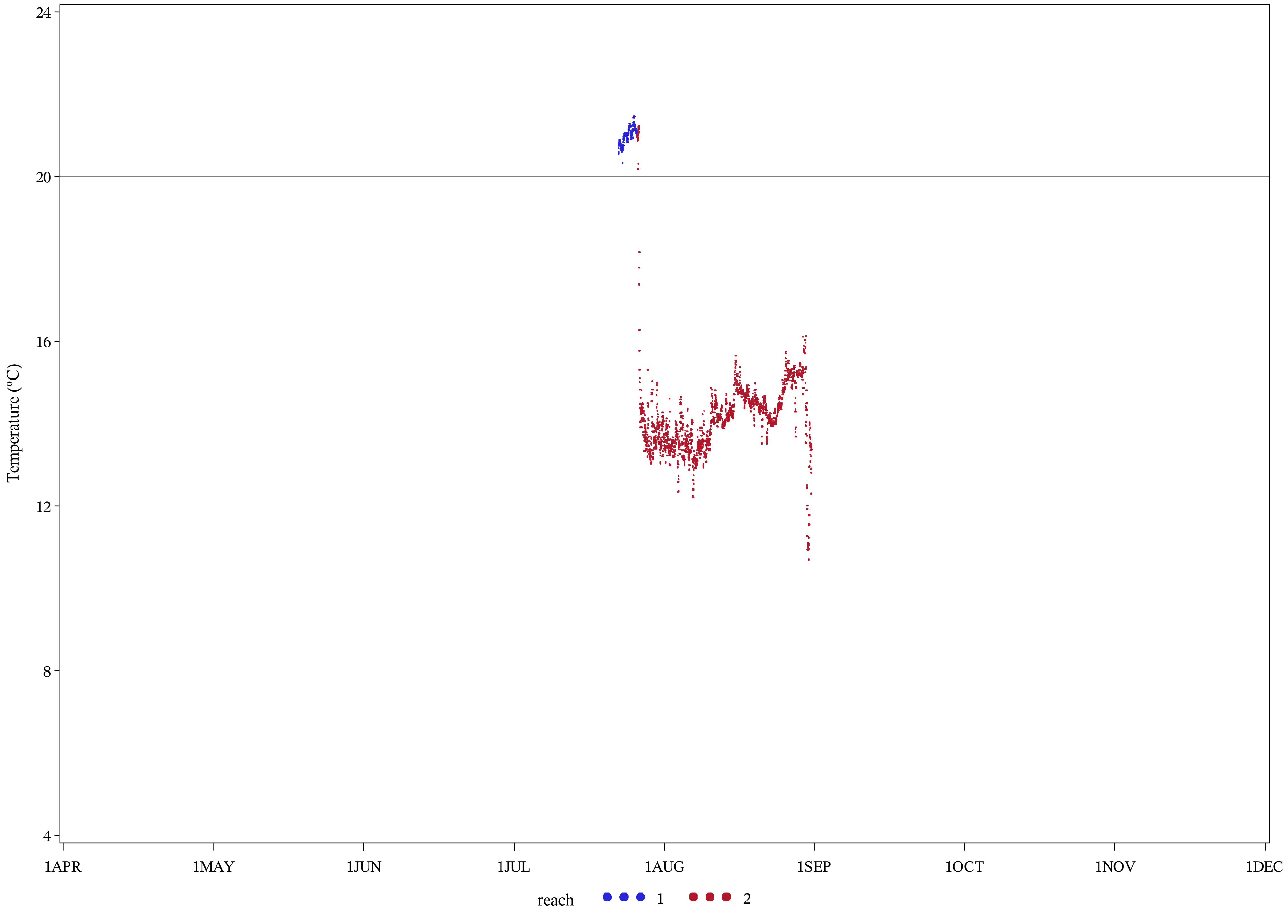

Steelhead
4630A

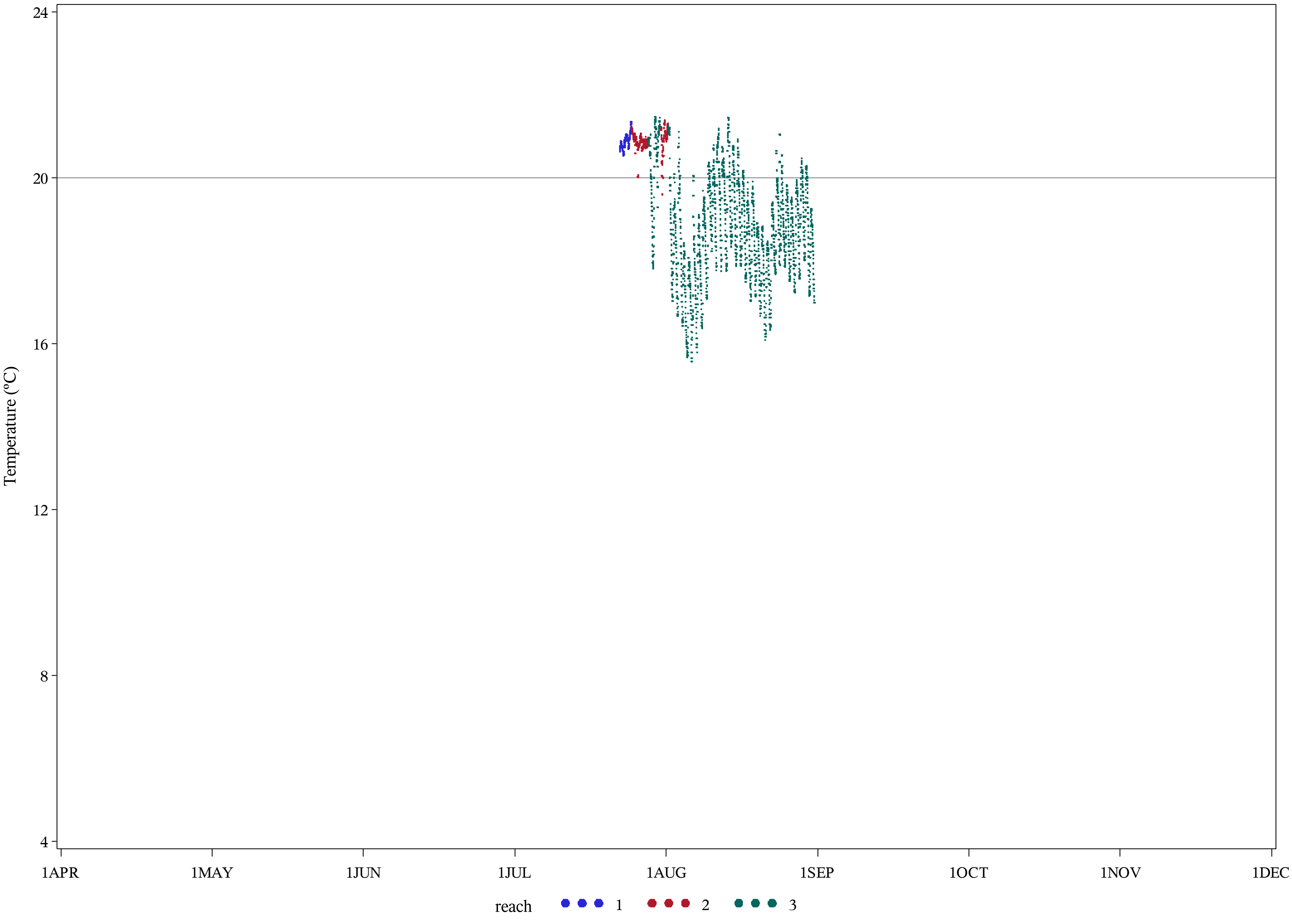

Steelhead
4631A

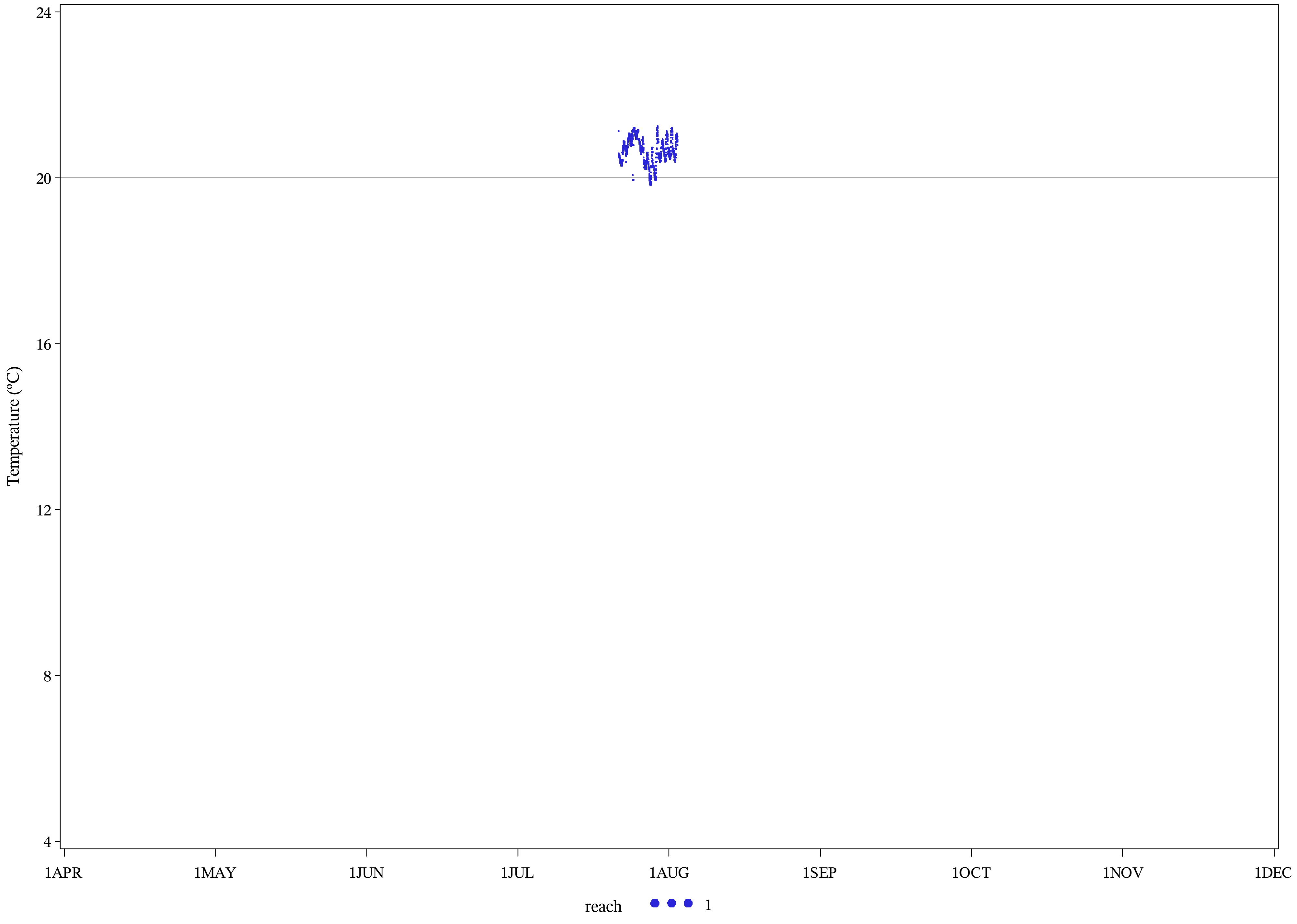

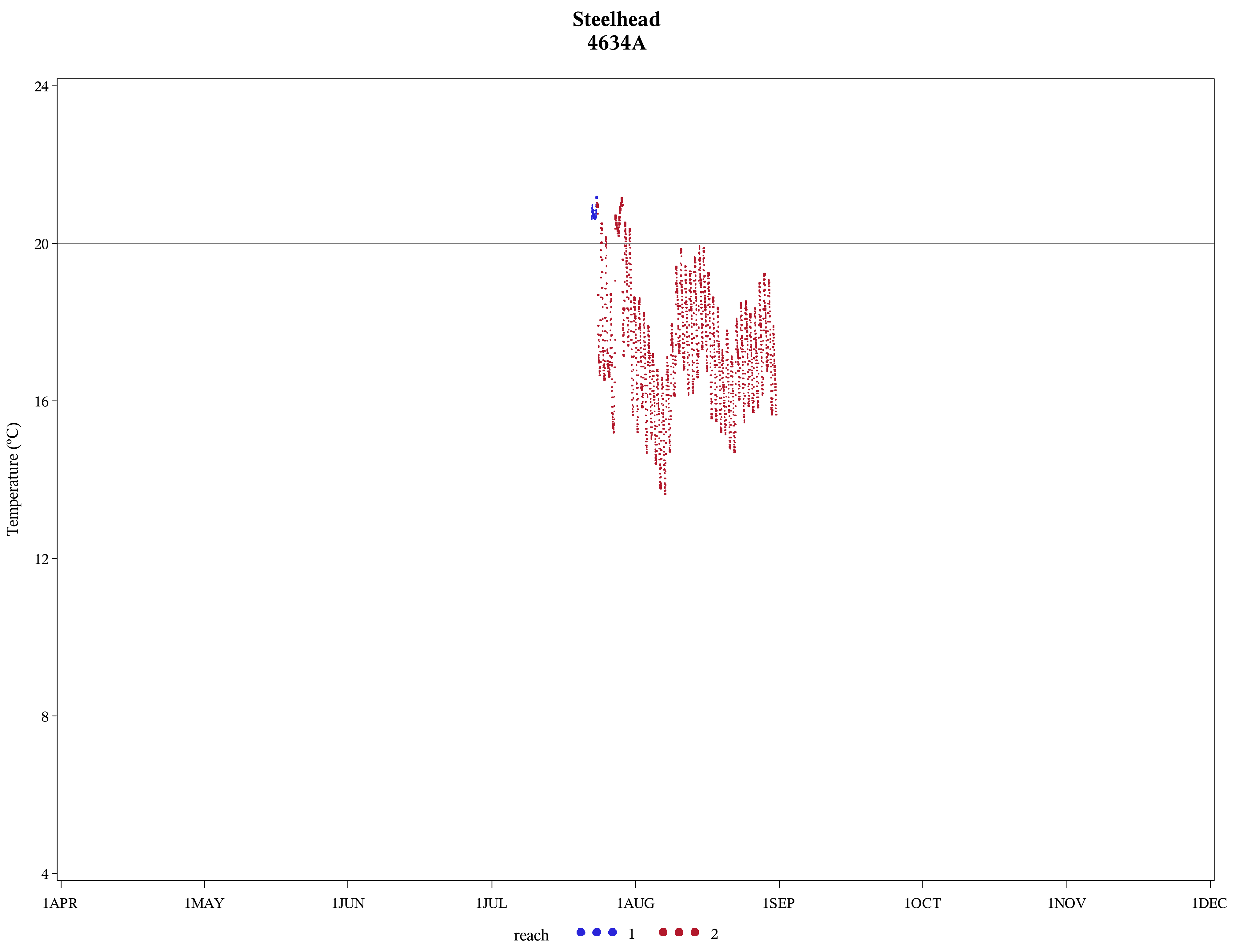

Steelhead
4634A
Temperature (°C)
24
20
16
12
8
4
1APR
1MAY
1JUN
1JUL
1AUG
1SEP
1OCT
1NOV
1DEC
reach
1
2

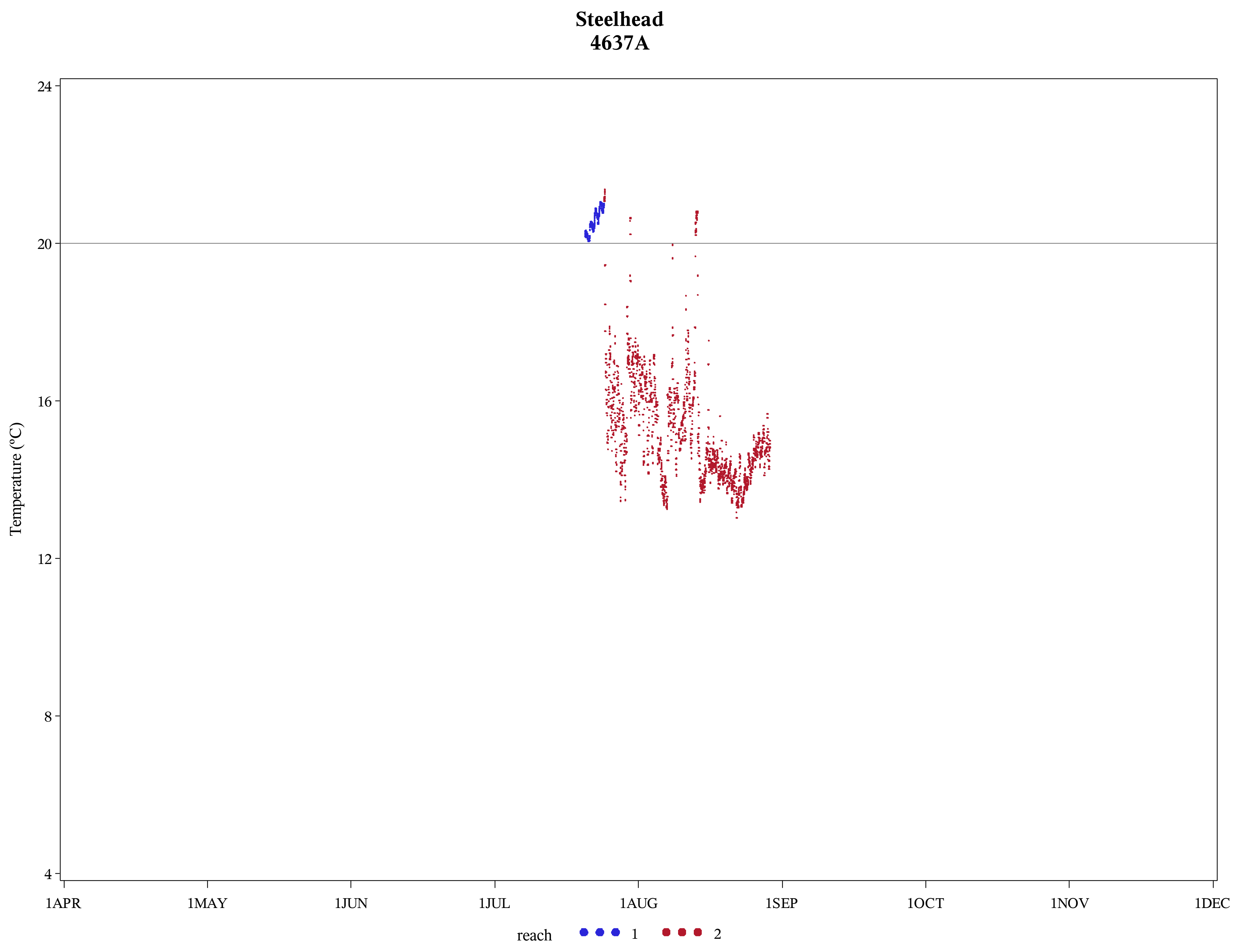

Steelhead
4637A
Temperature (°C)
24
20
16
12
8
4
1APR
1MAY
1JUN
1JUL
1AUG
1SEP
1OCT
1NOV
1DEC
reach
1
2

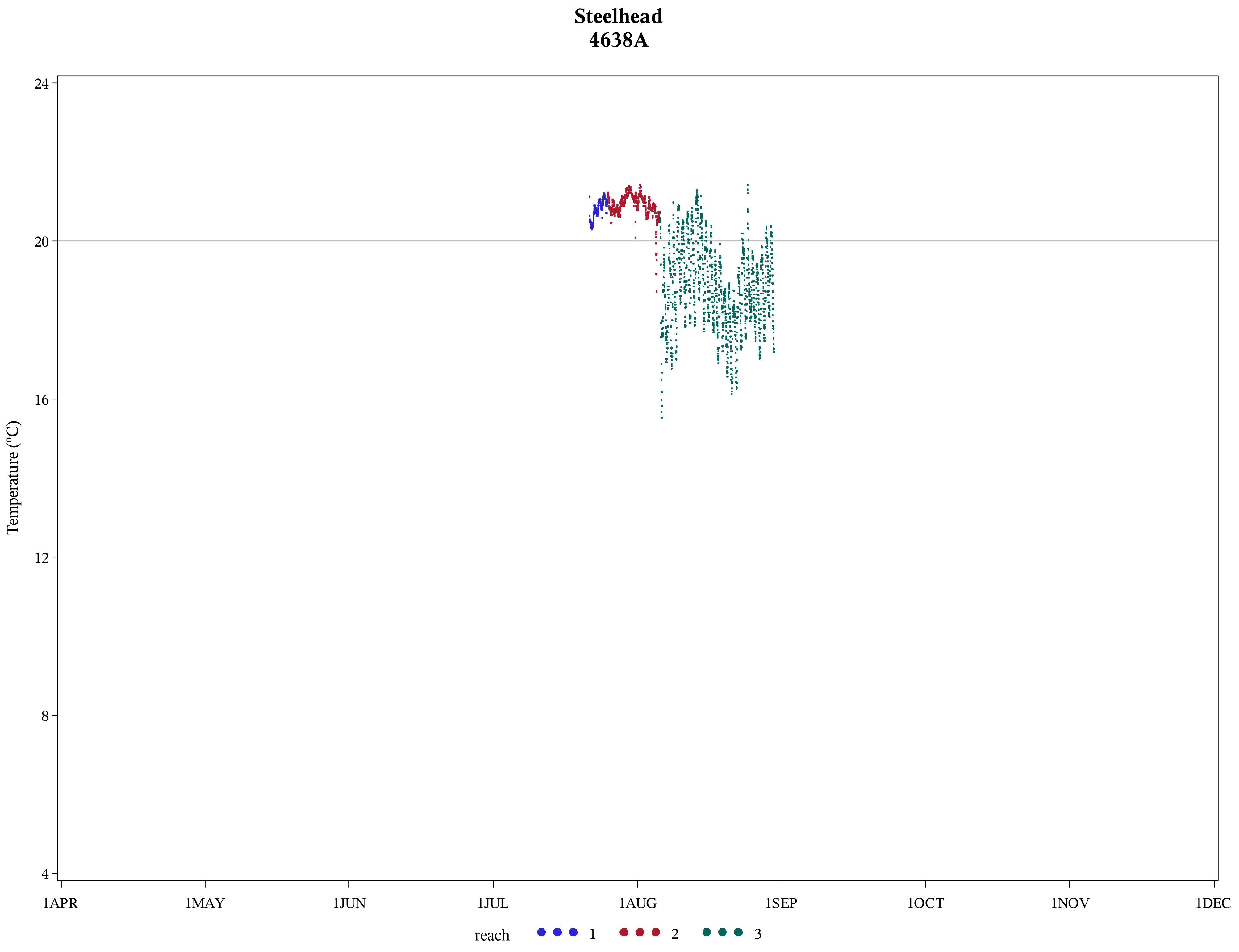

Steelhead
4638A
Temperature (°C)
24
20
16
12
8
4
1APR
1MAY
1JUN
1JUL
1AUG
1SEP
1OCT
1NOV
1DEC
reach
1
2
3

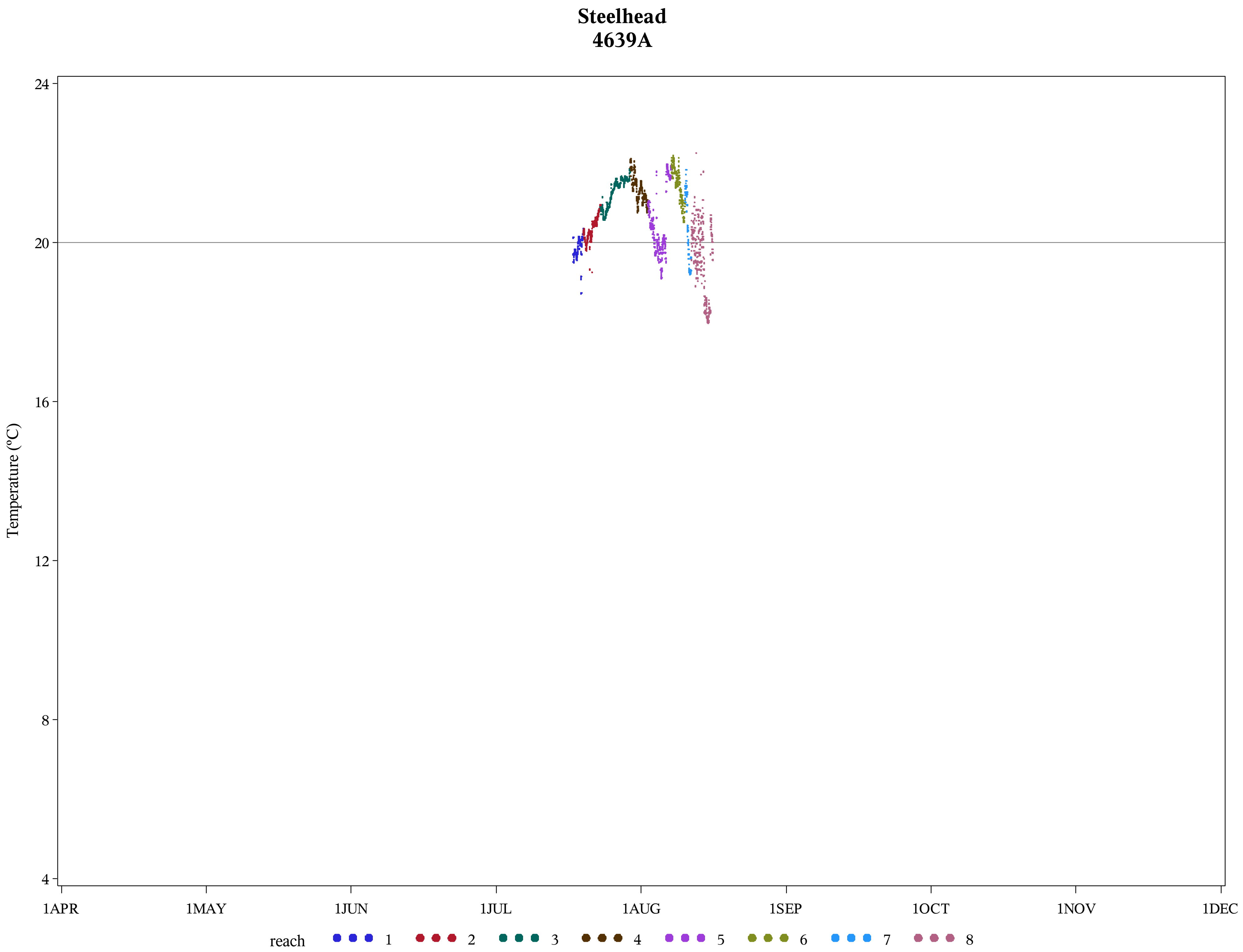

Steelhead
4639A
Temperature (°C)
24
20
16
12
8
4
1APR
1MAY
1JUN
1JUL
1AUG
1SEP
1OCT
1NOV
1DEC
reach
1
2
3
4
5
6
7
8

Steelhead
4639B

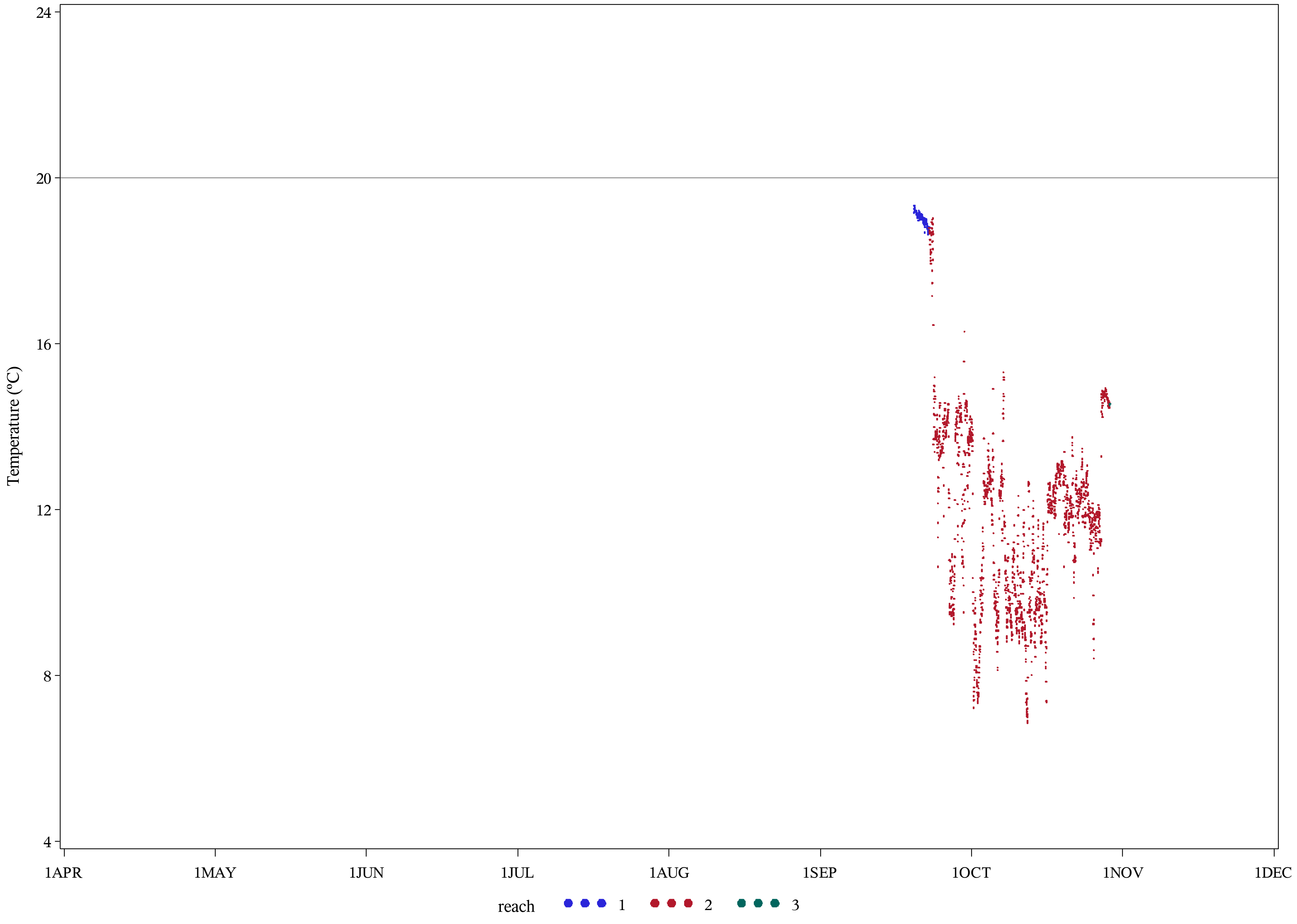

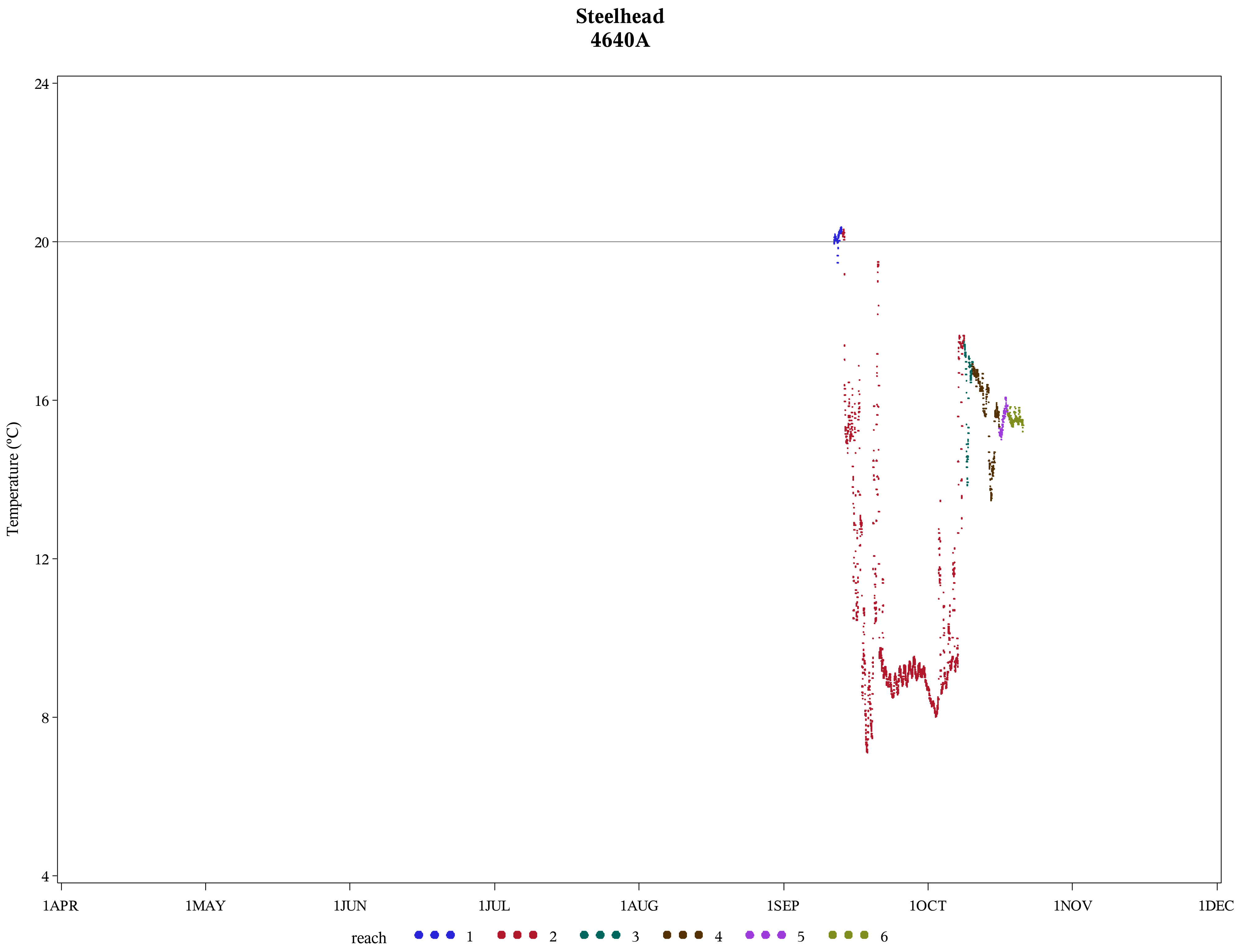

Steelhead
4640A
Temperature (°C)
24
20
16
12
8
4
1APR
1MAY
1JUN
1JUL
1AUG
1SEP
1OCT
1NOV
1DEC
reach
1
2
3
4
5
6

Steelhead
4642A

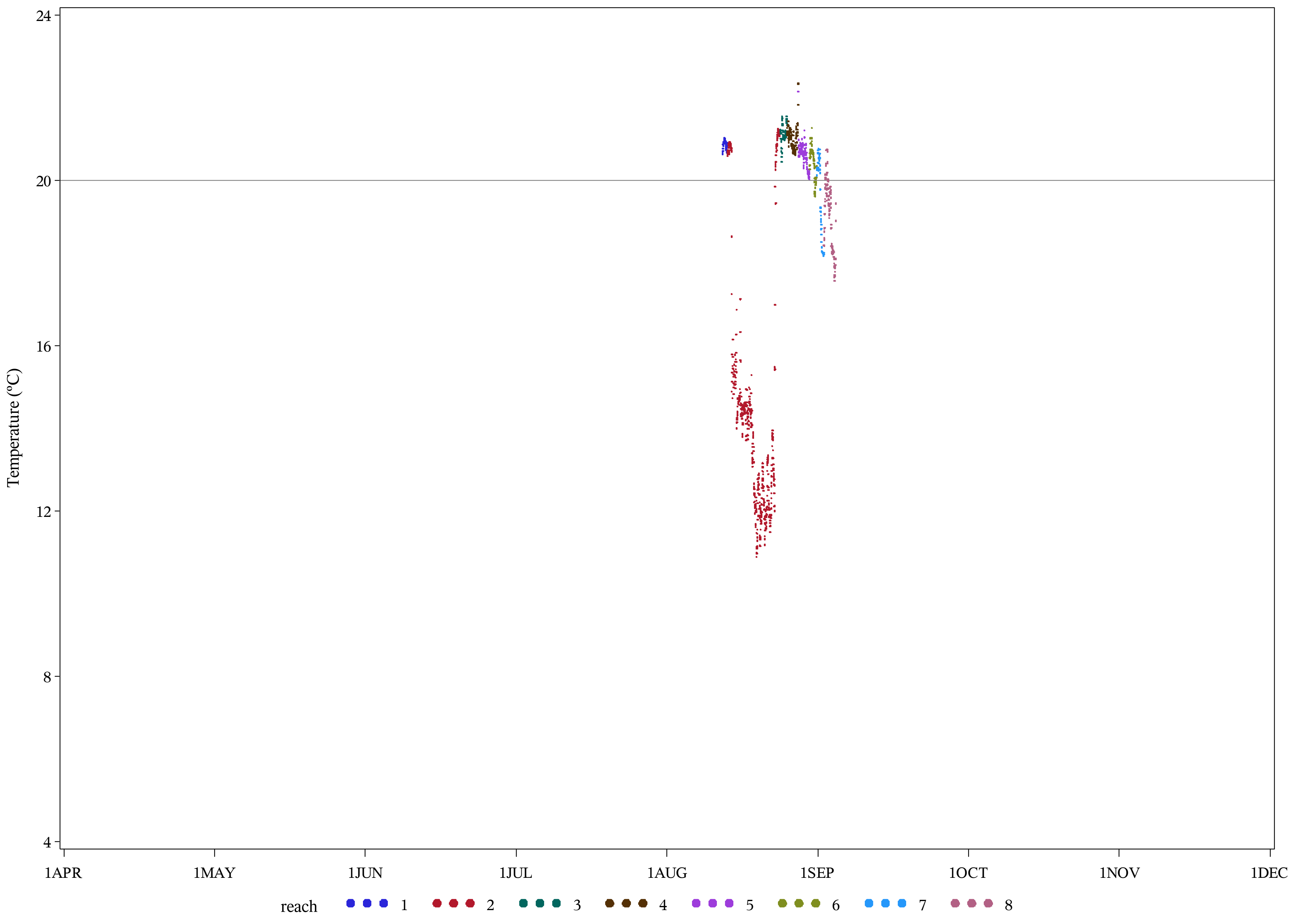

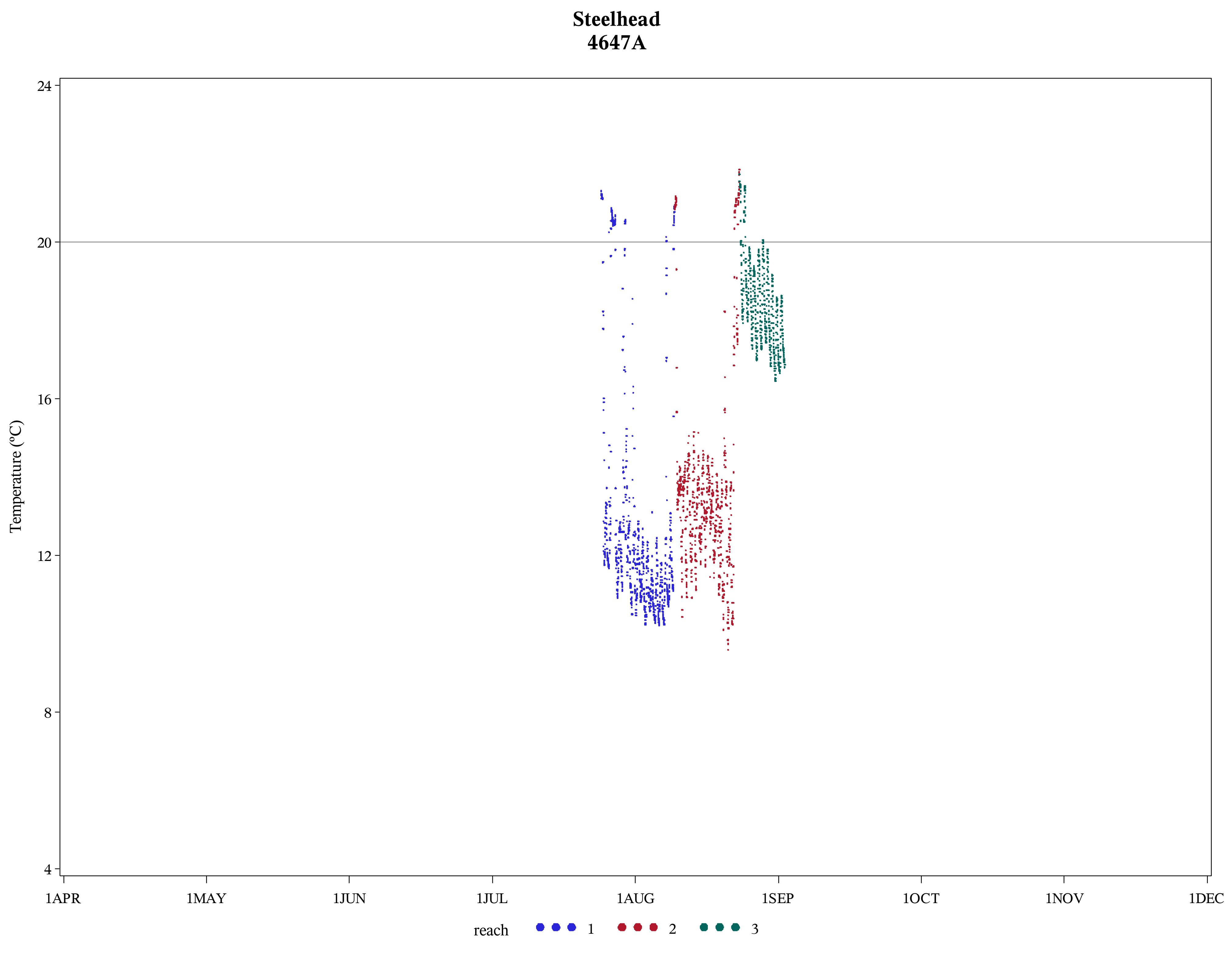

Steelhead
4647A
24
20
16
12
8
4
Temperature (°C)
1APR
1MAY
1JUN
1JUL
1AUG
1SEP
1OCT
1NOV
1DEC
reach
1
2
3

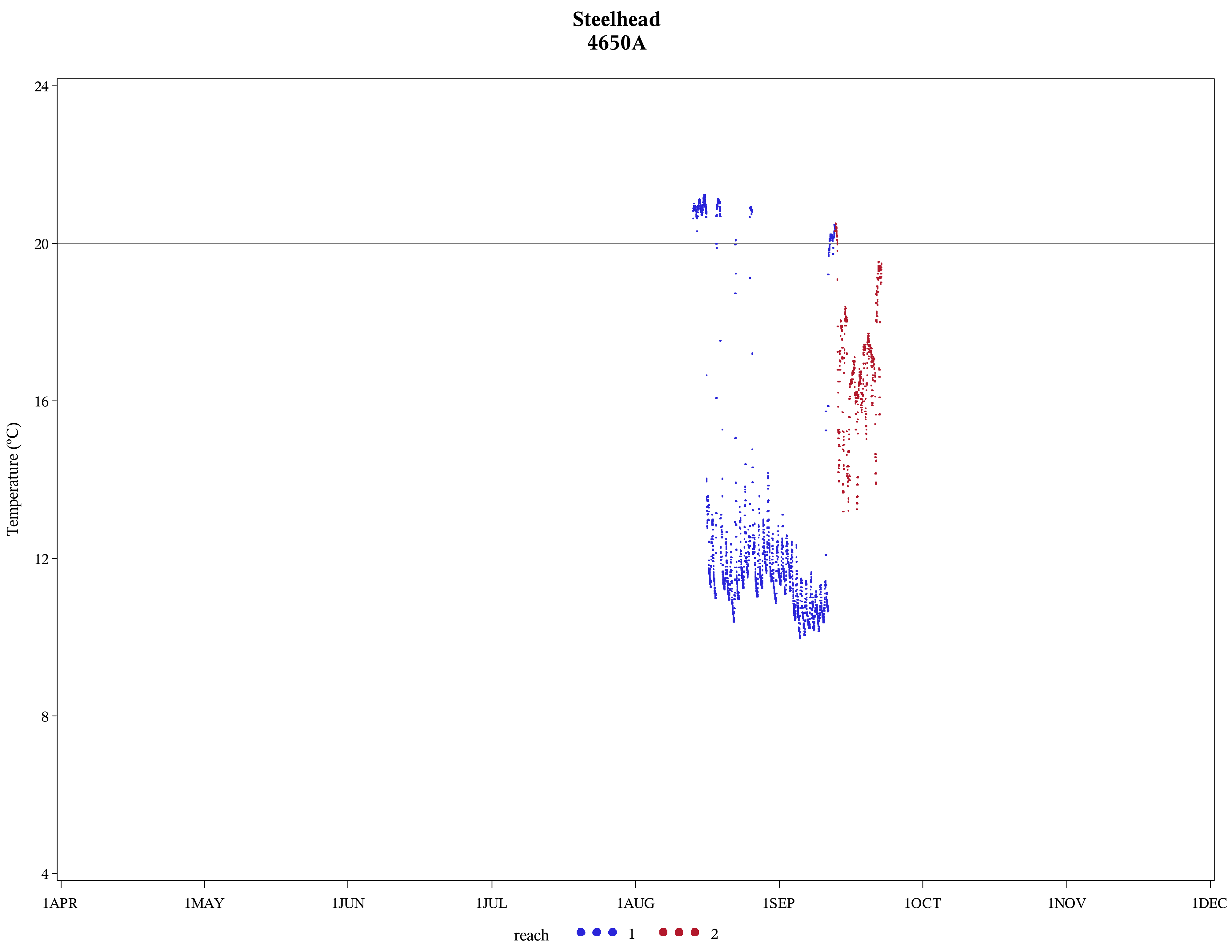

Steelhead
4650A
Temperature (°C)
24
20
16
12
8
4
1APR
1MAY
1JUN
1JUL
1AUG
1SEP
1OCT
1NOV
1DEC
reach
1
2

Steelhead
4651A

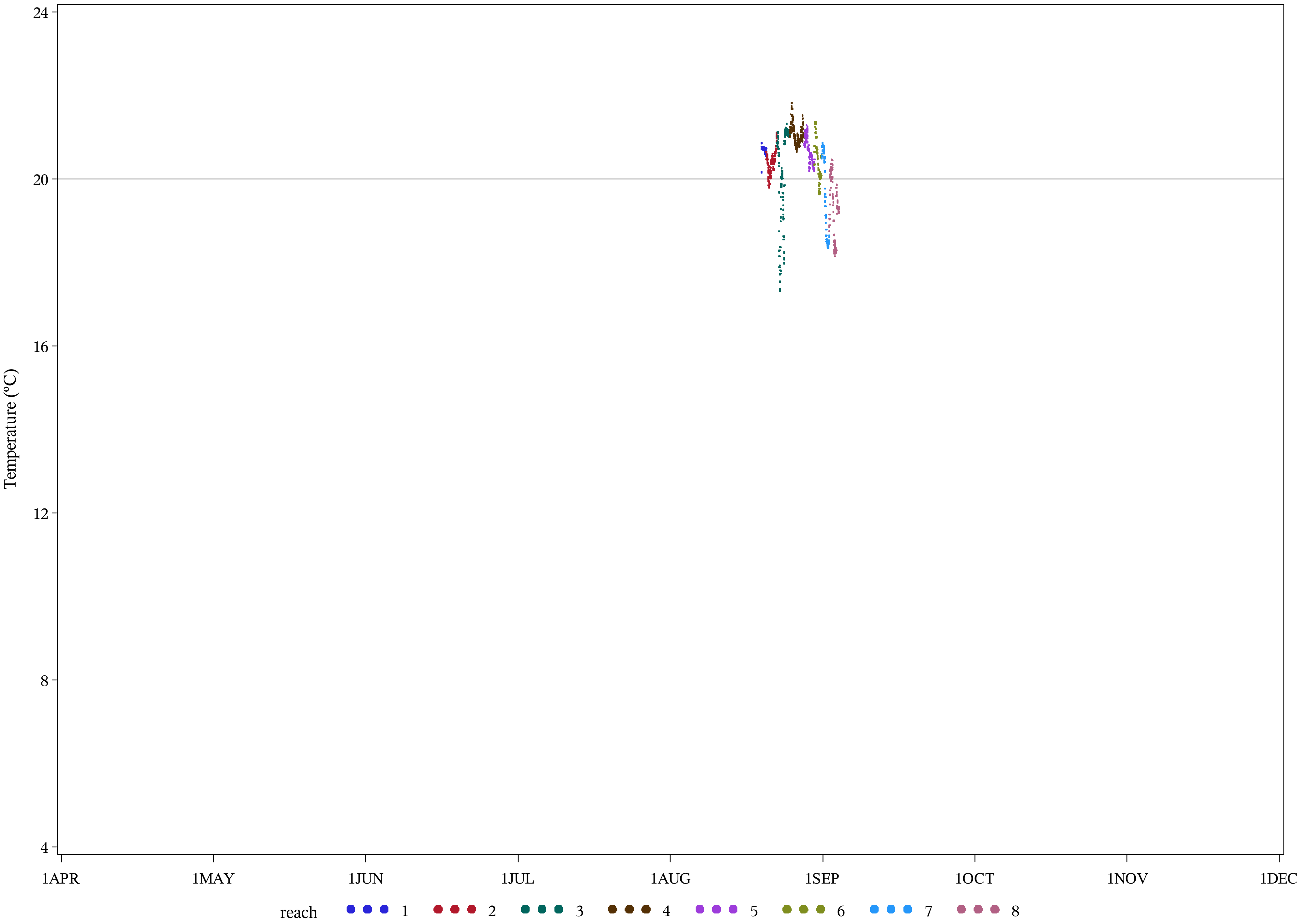

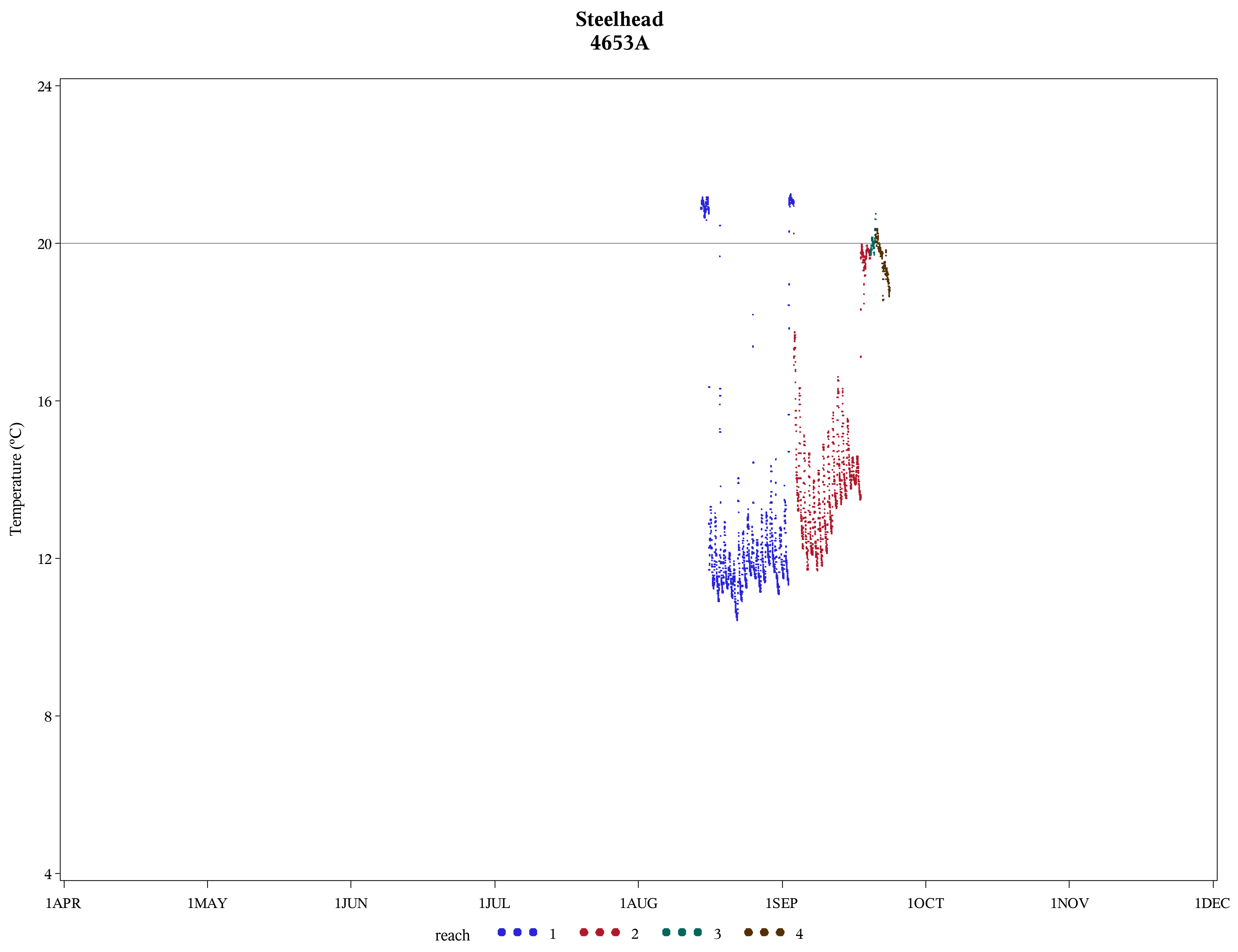
Steelhead
4653A
Temperature (°C)
24
20
16
12
8
4
1APR
1MAY
1JUN
1JUL
1AUG
1SEP
1OCT
1NOV
1DEC
reach
1
2
3
4

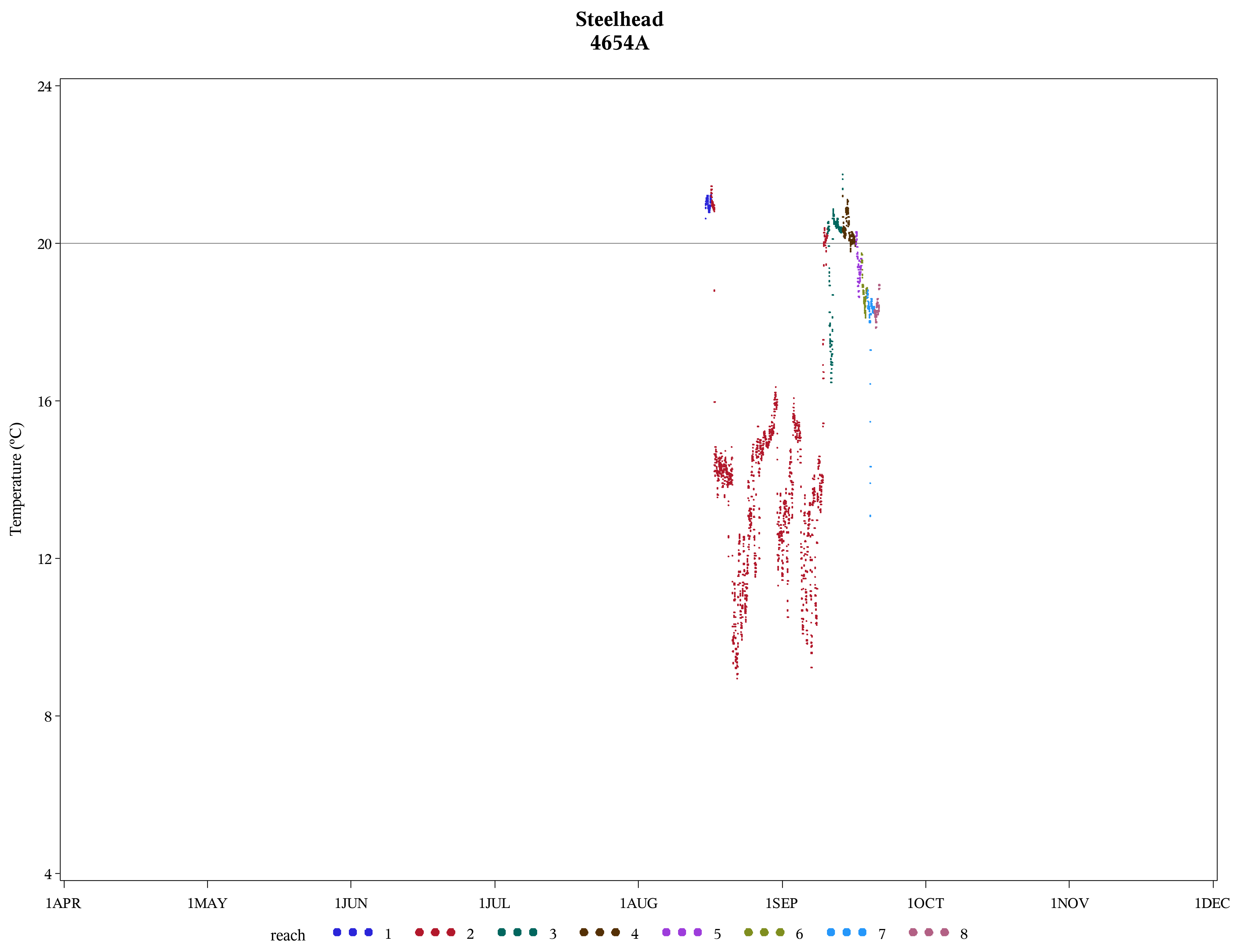

Steelhead
4654A
Temperature (°C)
24
20
16
12
8
4
1APR
1MAY
1JUN
1JUL
1AUG
1SEP
1OCT
1NOV
1DEC
reach
1
2
3
4
5
6
7
8

Steelhead
4656A

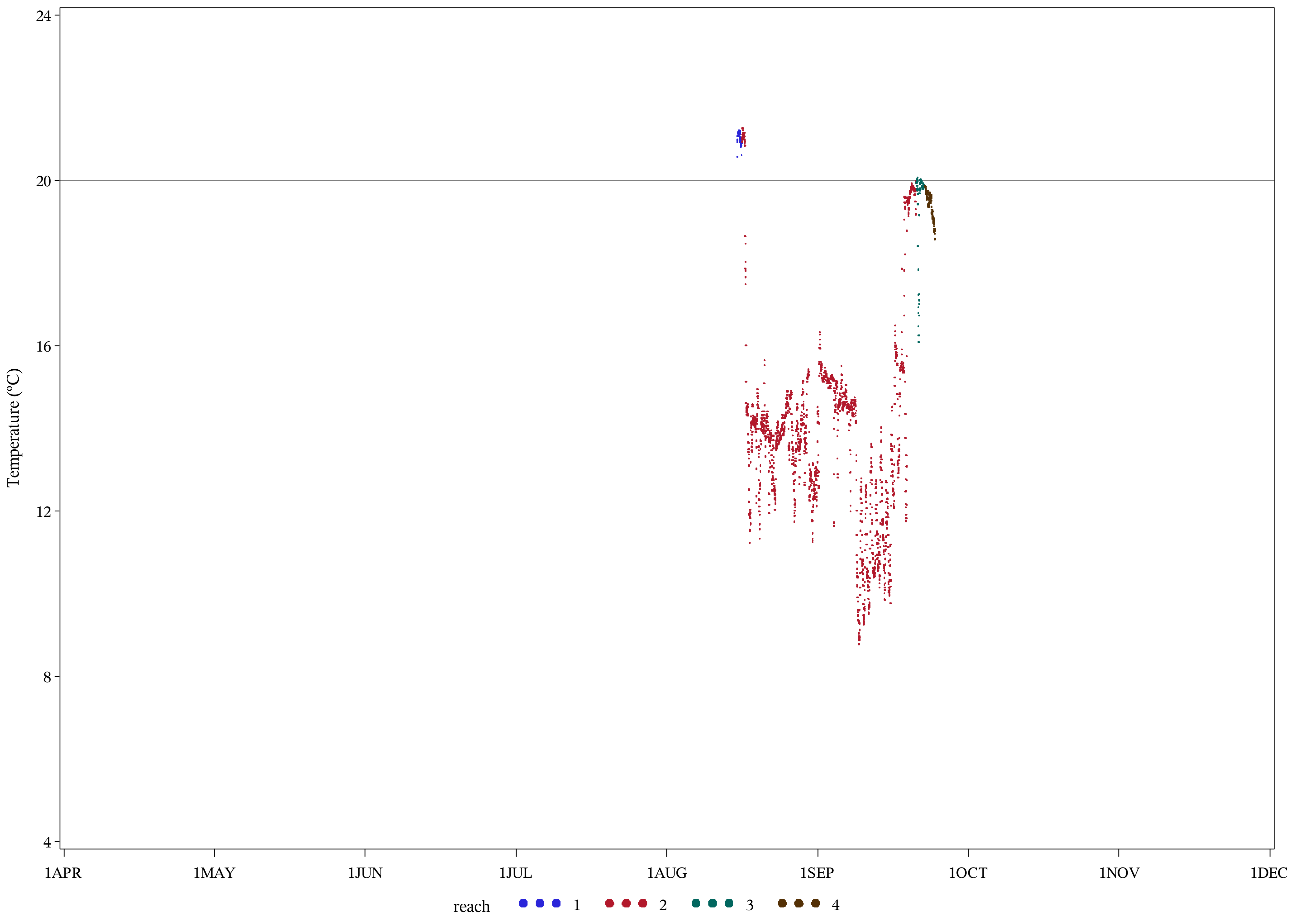

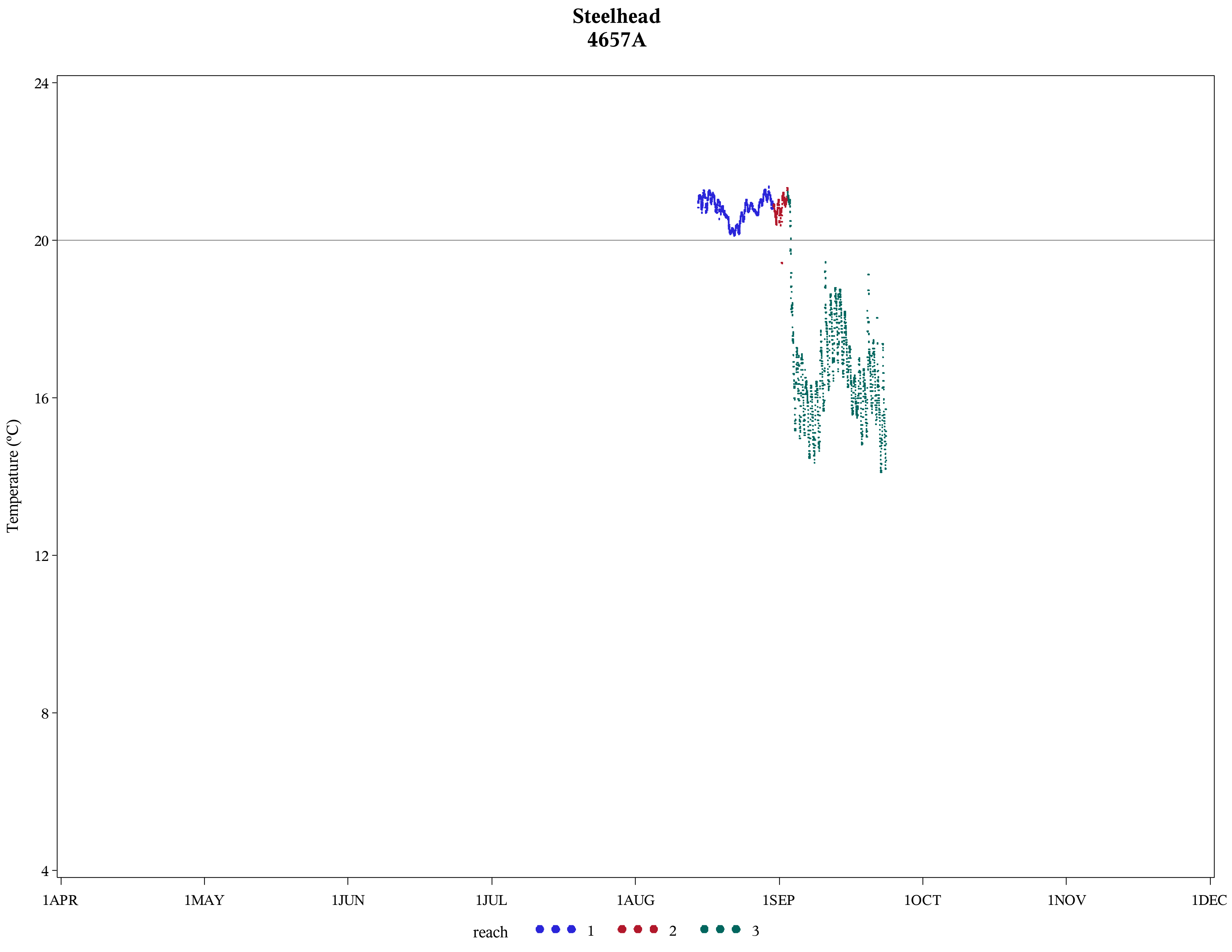

Steelhead
4657A
Temperature (°C)
24
20
16
12
8
4
1APR
1MAY
1JUN
1JUL
1AUG
1SEP
1OCT
1NOV
1DEC
reach
1
2
3

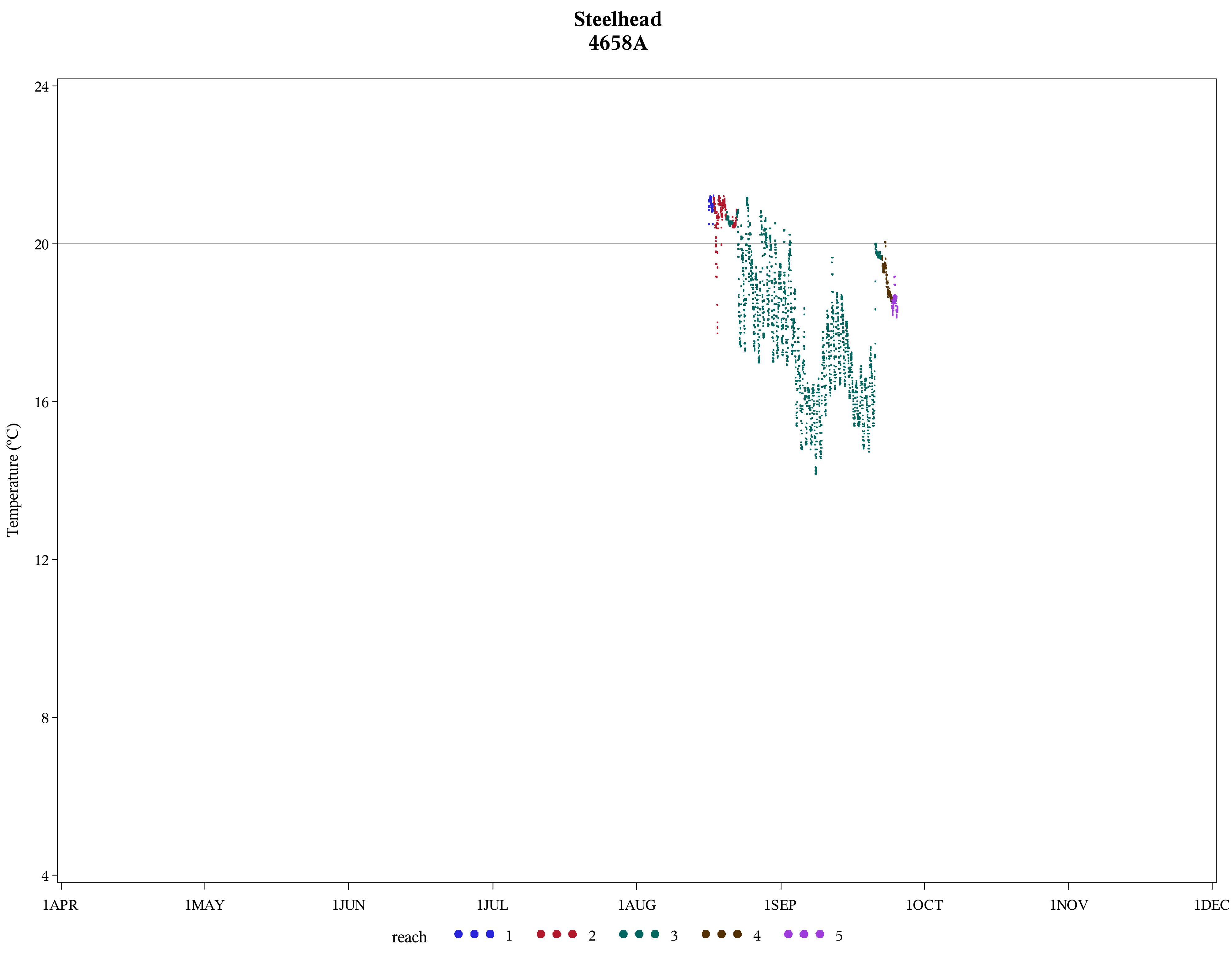

Steelhead
4658A
Temperature (°C)
24
20
16
12
8
4
1APR
1MAY
1JUN
1JUL
1AUG
1SEP
1OCT
1NOV
1DEC
reach
1
2
3
4
5

Steelhead
4659A

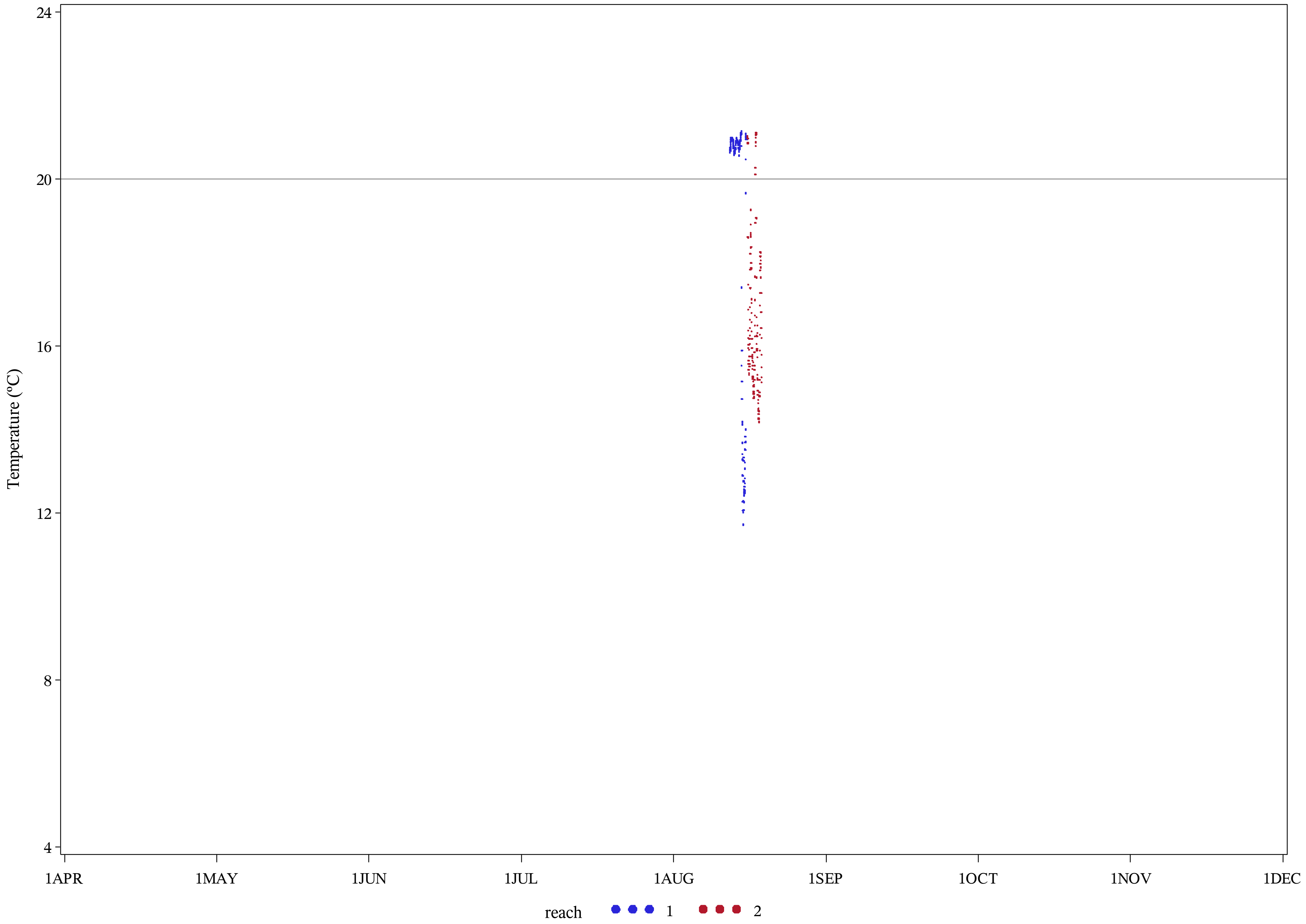

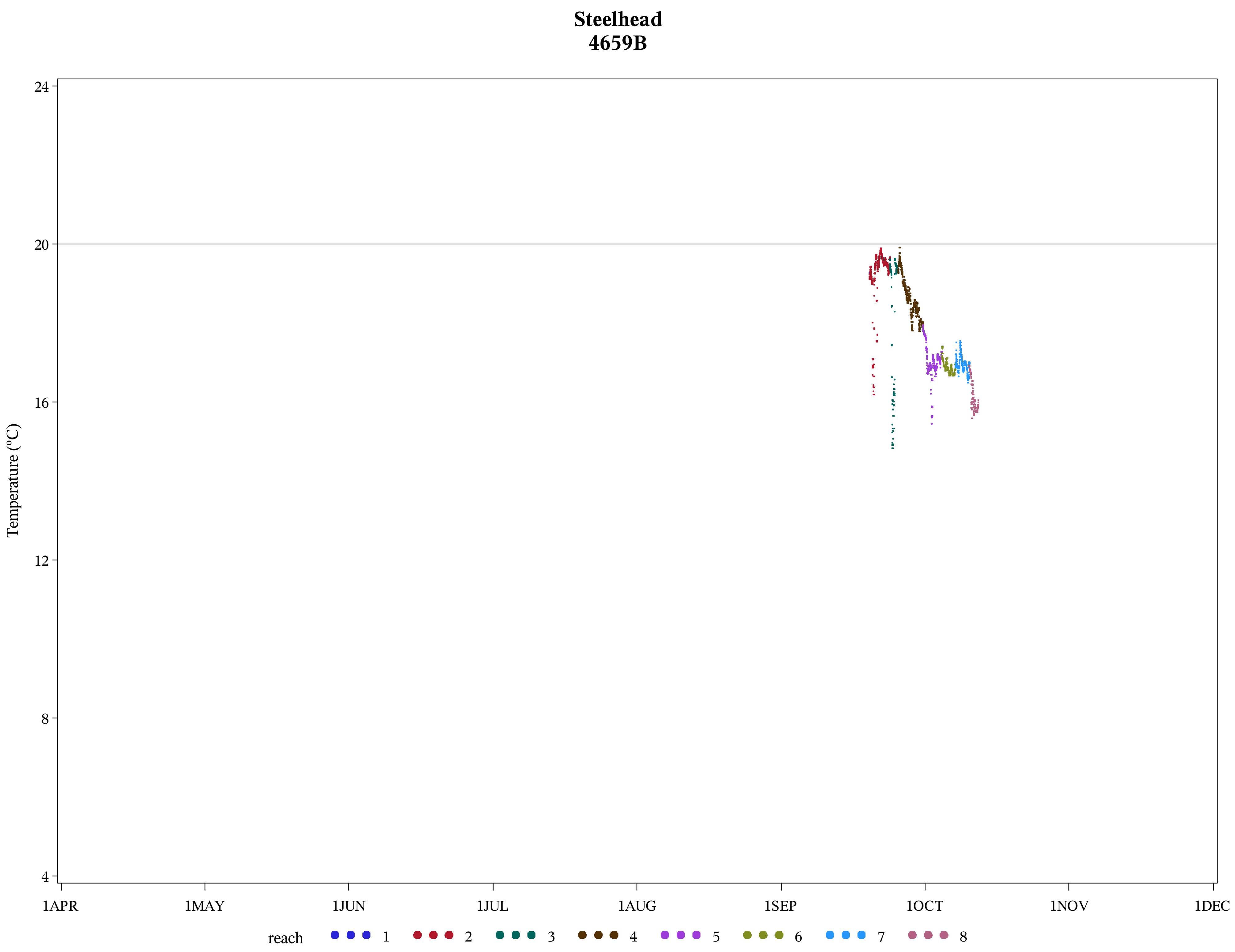

Steelhead
4659B
Temperature (°C)
24
20
16
12
8
4
1APR
1MAY
1JUN
1JUL
1AUG
1SEP
1OCT
1NOV
1DEC
reach
1
2
3
4
5
6
7
8

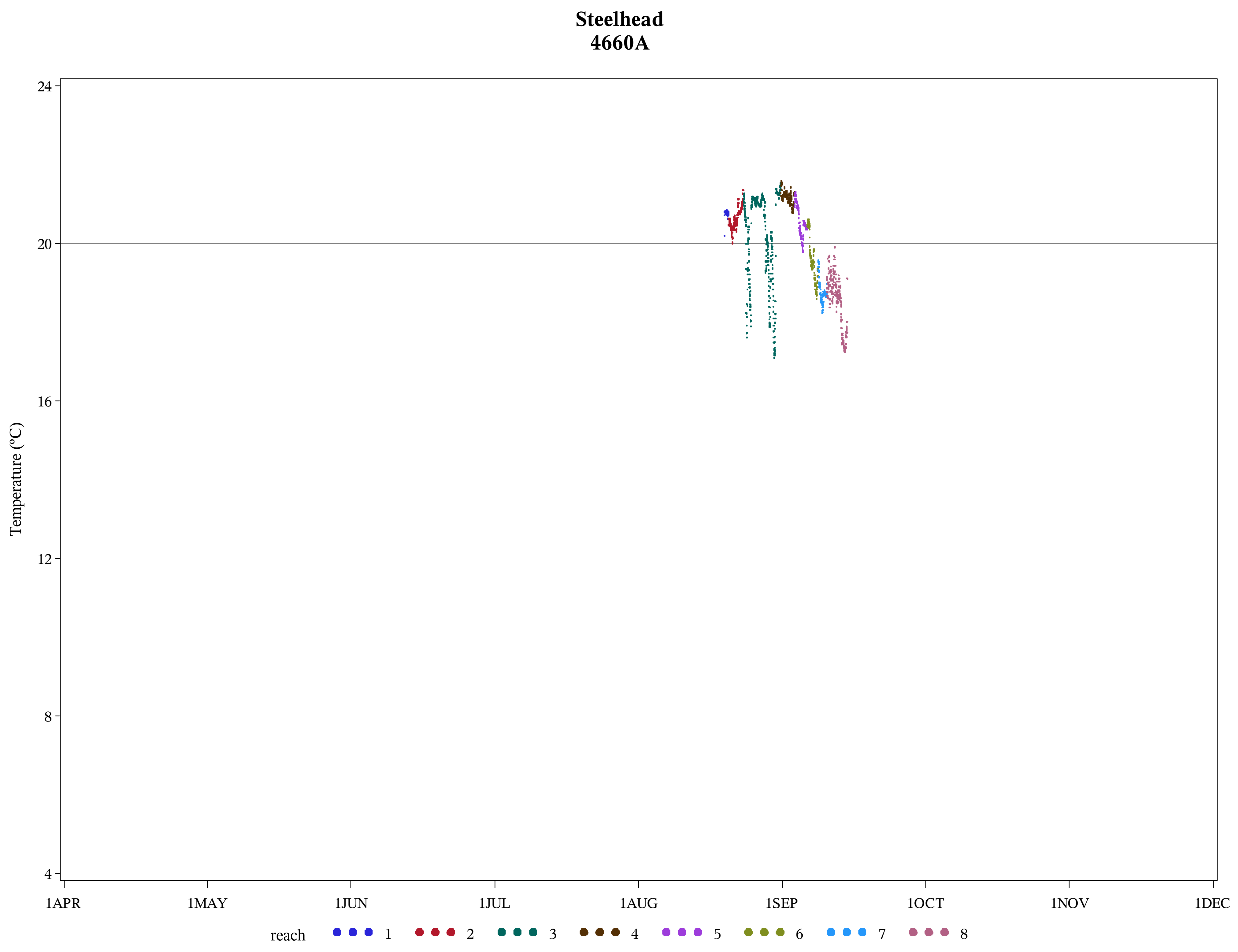
Steelhead
4660A
Temperature (°C)
24
20
16
12
8
4
1APR
1MAY
1JUN
1JUL
1AUG
1SEP
1OCT
1NOV
1DEC
reach
1
2
3
4
5
6
7
8

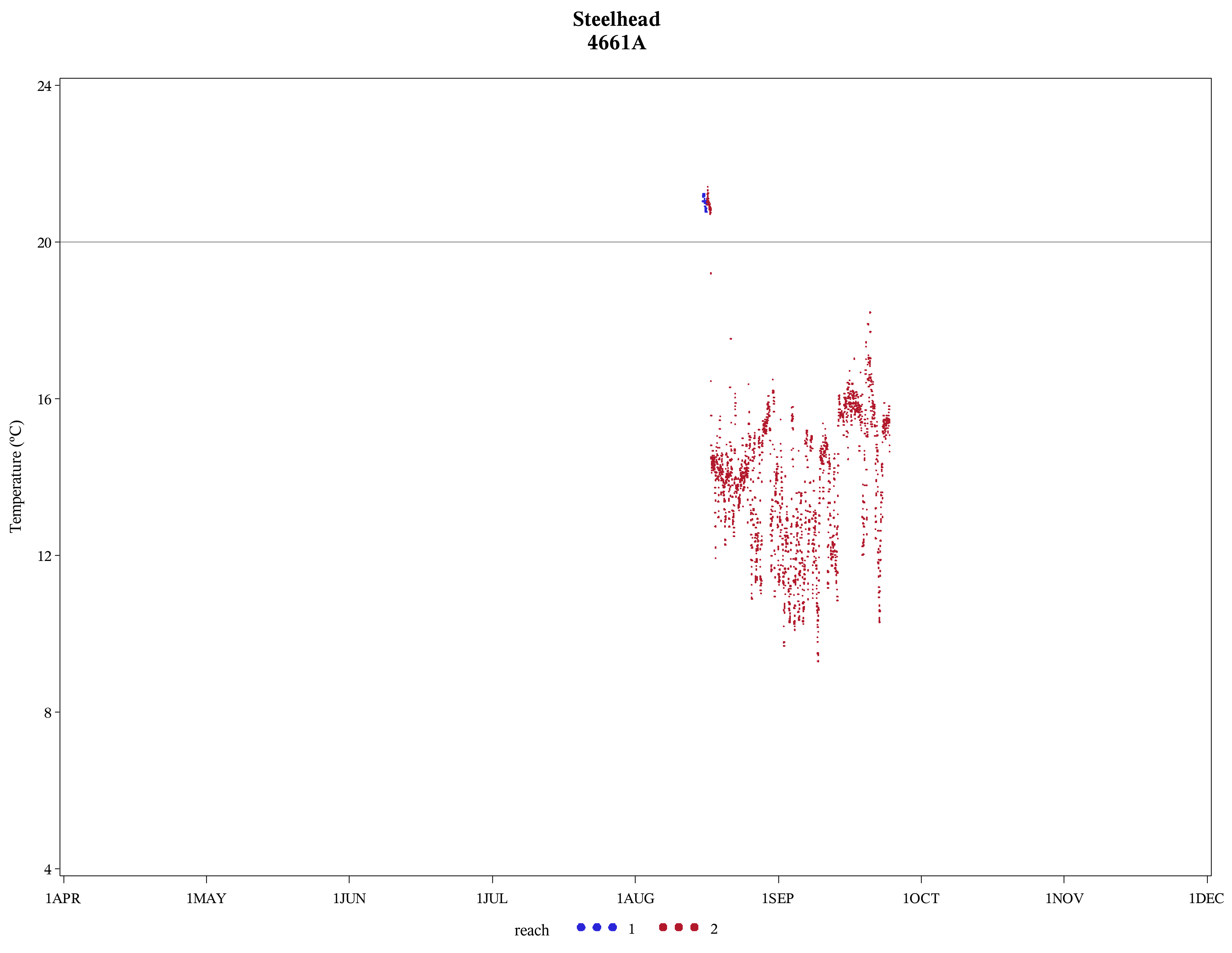
Steelhead
4661A
Temperature (°C)
24
20
16
12
8
4
1APR
1MAY
1JUN
1JUL
1AUG
1SEP
1OCT
1NOV
1DEC
reach
1
2

Steelhead
4663A

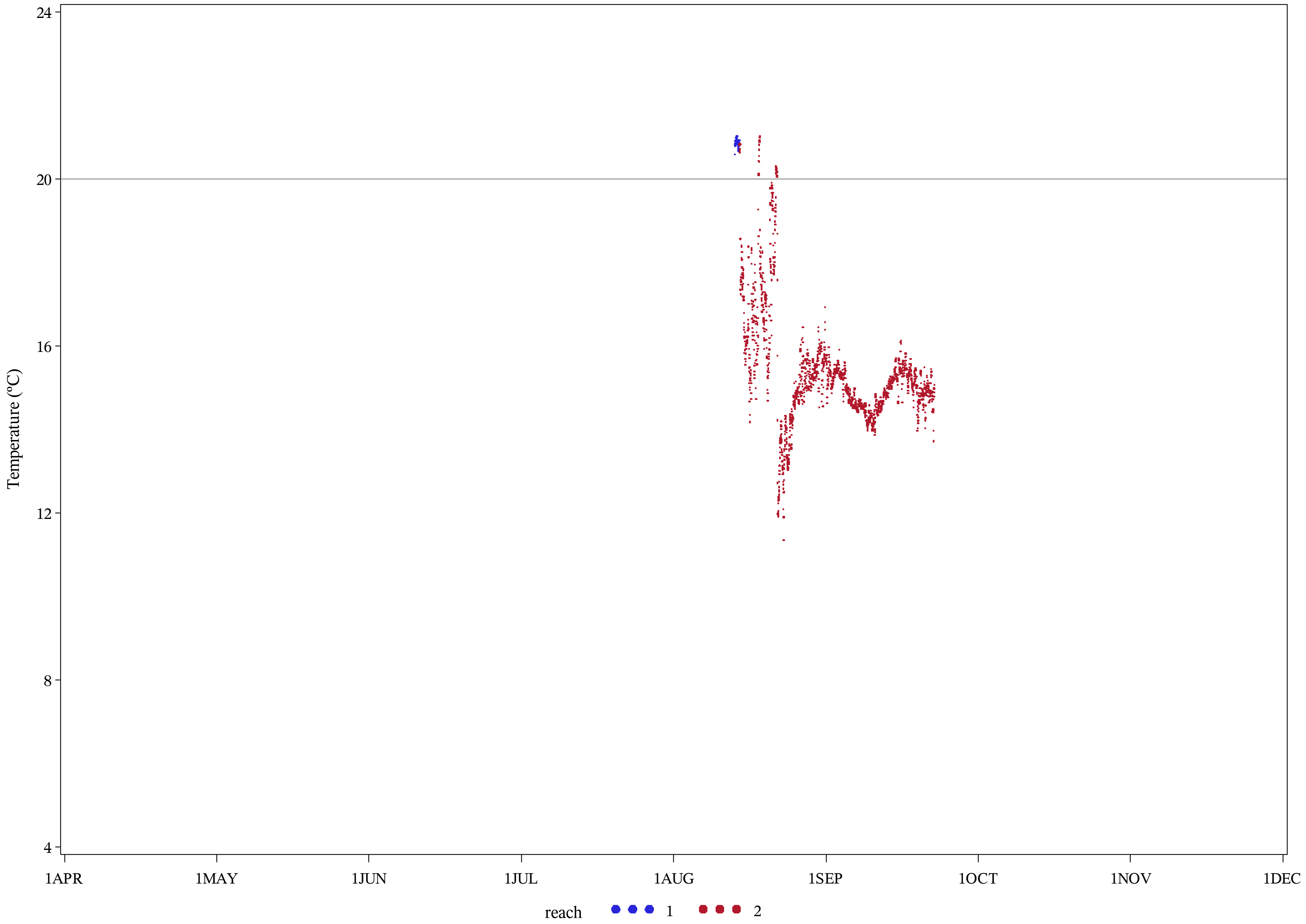

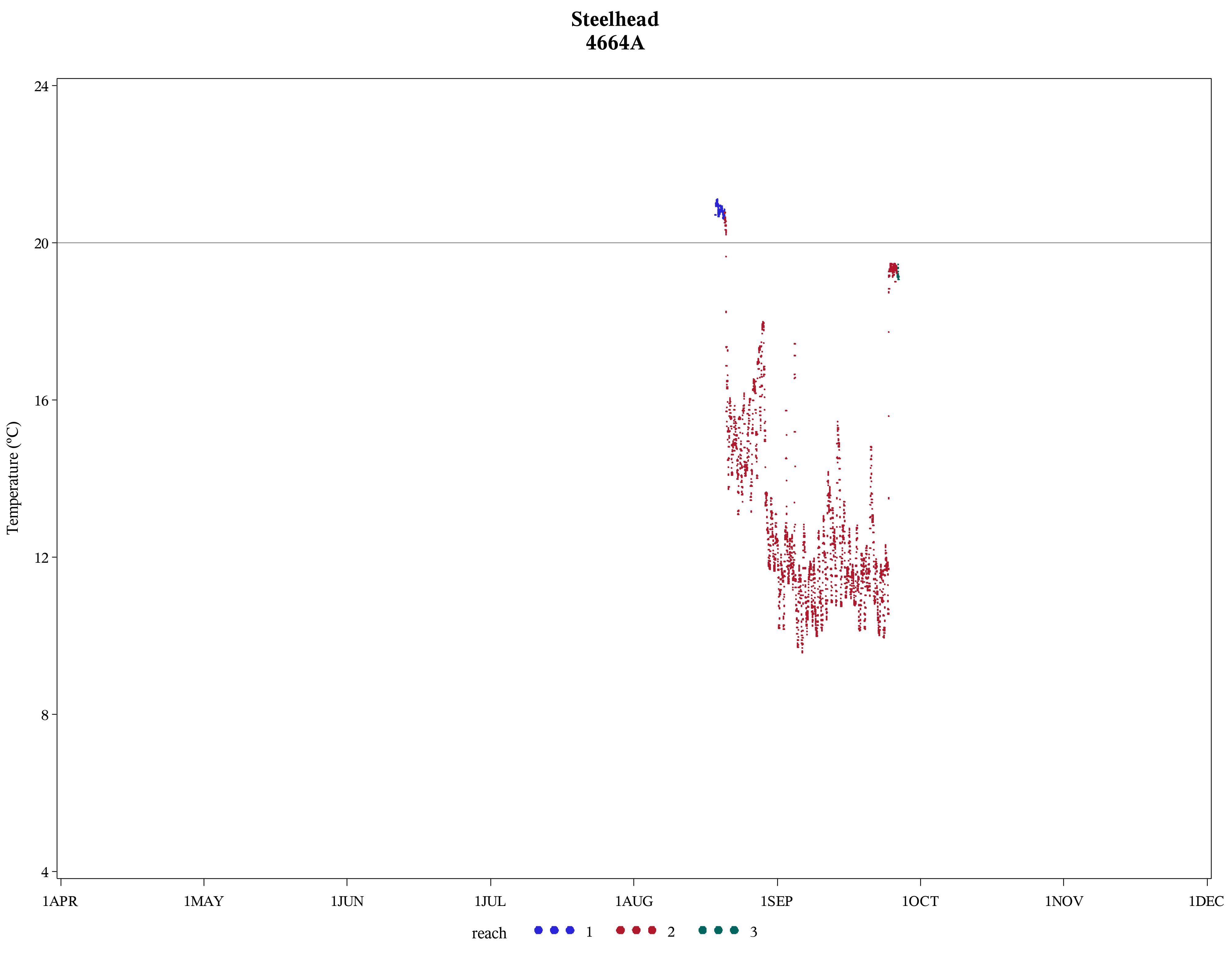
Steelhead
4664A
Temperature (°C)
24
20
16
12
8
4
1APR
1MAY
1JUN
1JUL
1AUG
1SEP
1OCT
1NOV
1DEC
reach
1
2
3

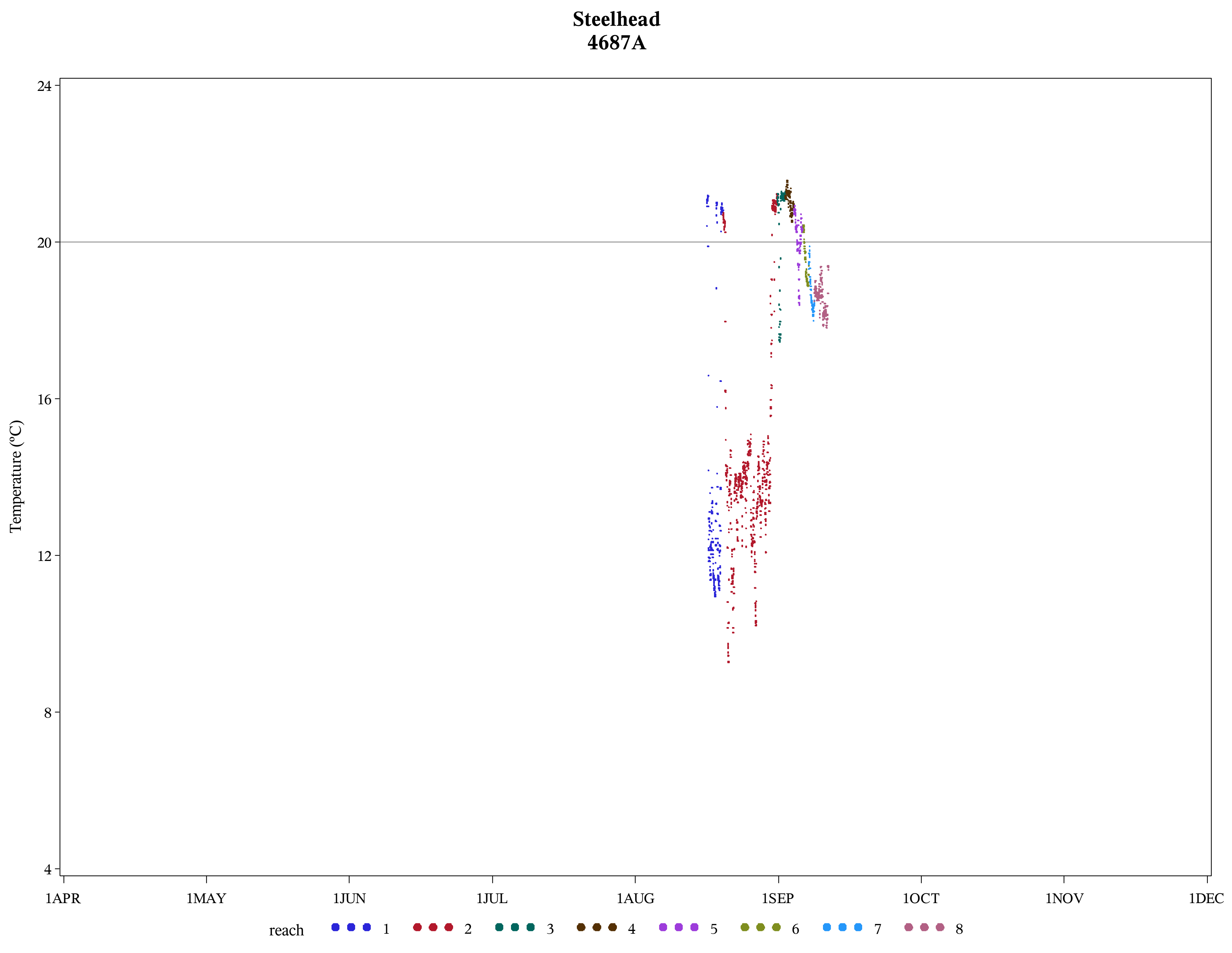
Steelhead
4687A
Temperature (°C)
24
20
16
12
8
4
1APR
1MAY
1JUN
1JUL
1AUG
1SEP
1OCT
1NOV
1DEC
reach
1
2
3
4
5
6
7
8

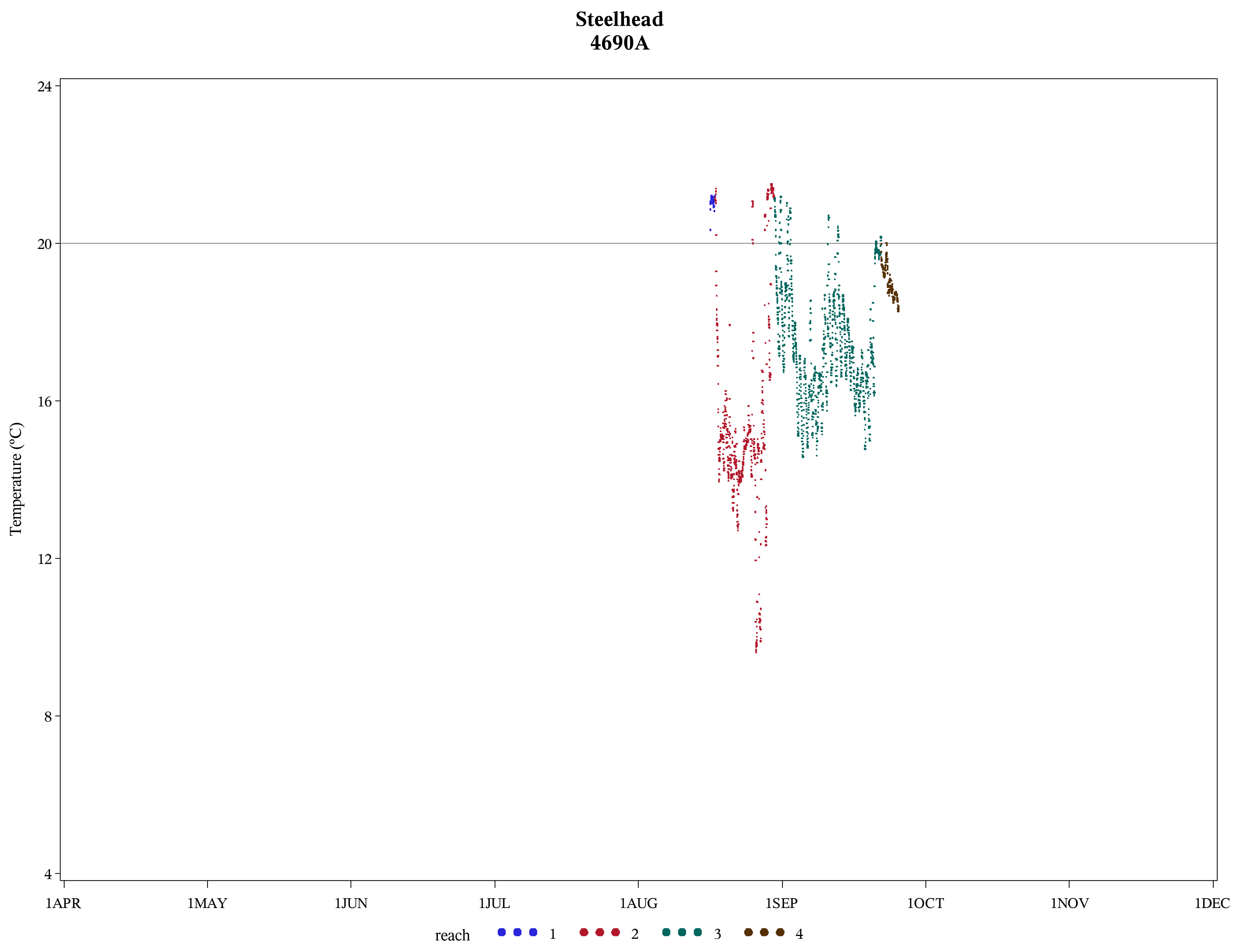

Steelhead
4690A
Temperature (°C)
24
20
16
12
8
4
1APR
1MAY
1JUN
1JUL
1AUG
1SEP
1OCT
1NOV
1DEC
reach
1
2
3
4

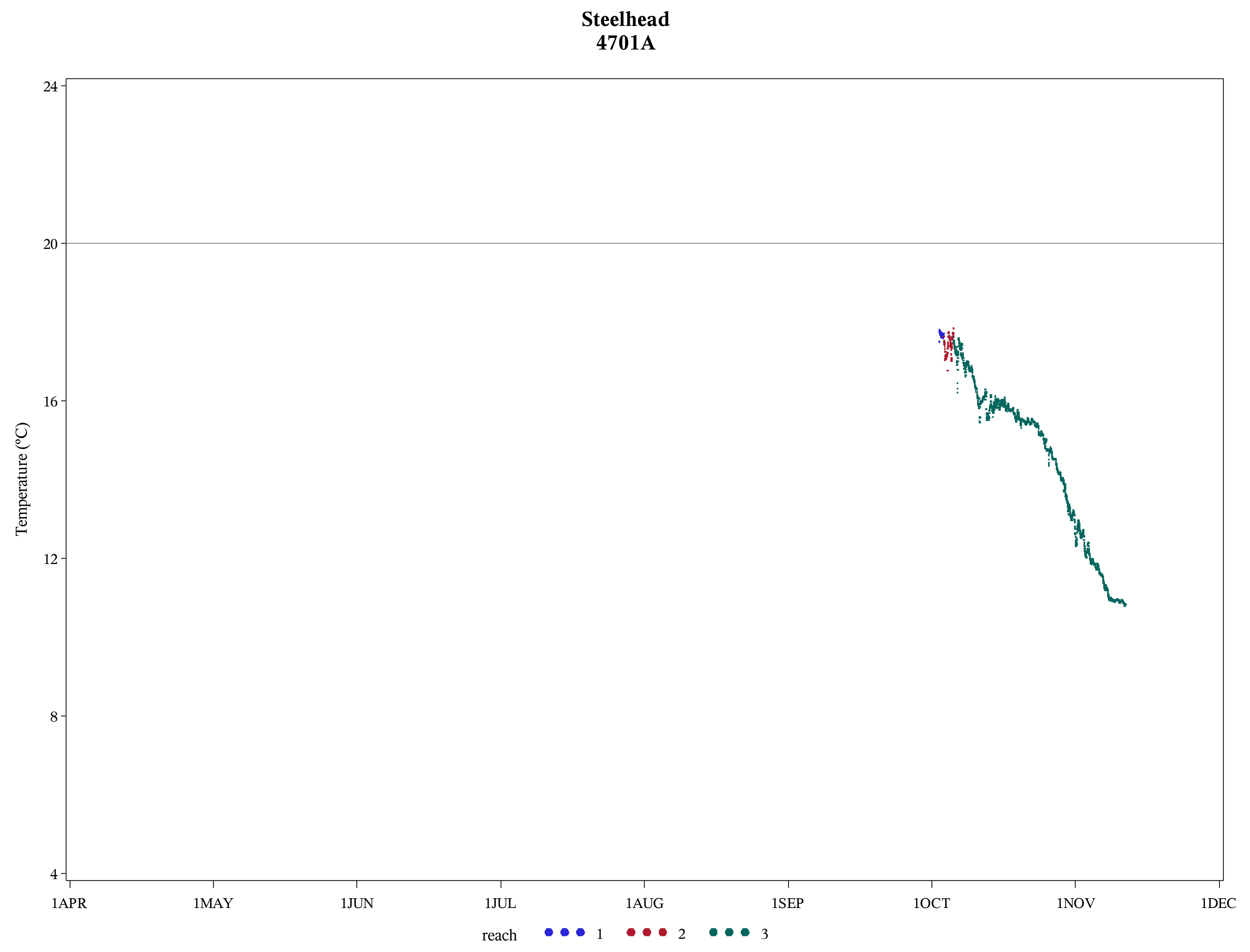

Steelhead
4701A
Temperature (°C)
24
20
16
12
8
4
1APR
1MAY
1JUN
1JUL
1AUG
1SEP
1OCT
1NOV
1DEC
reach
1
2
3

Steelhead
4730B

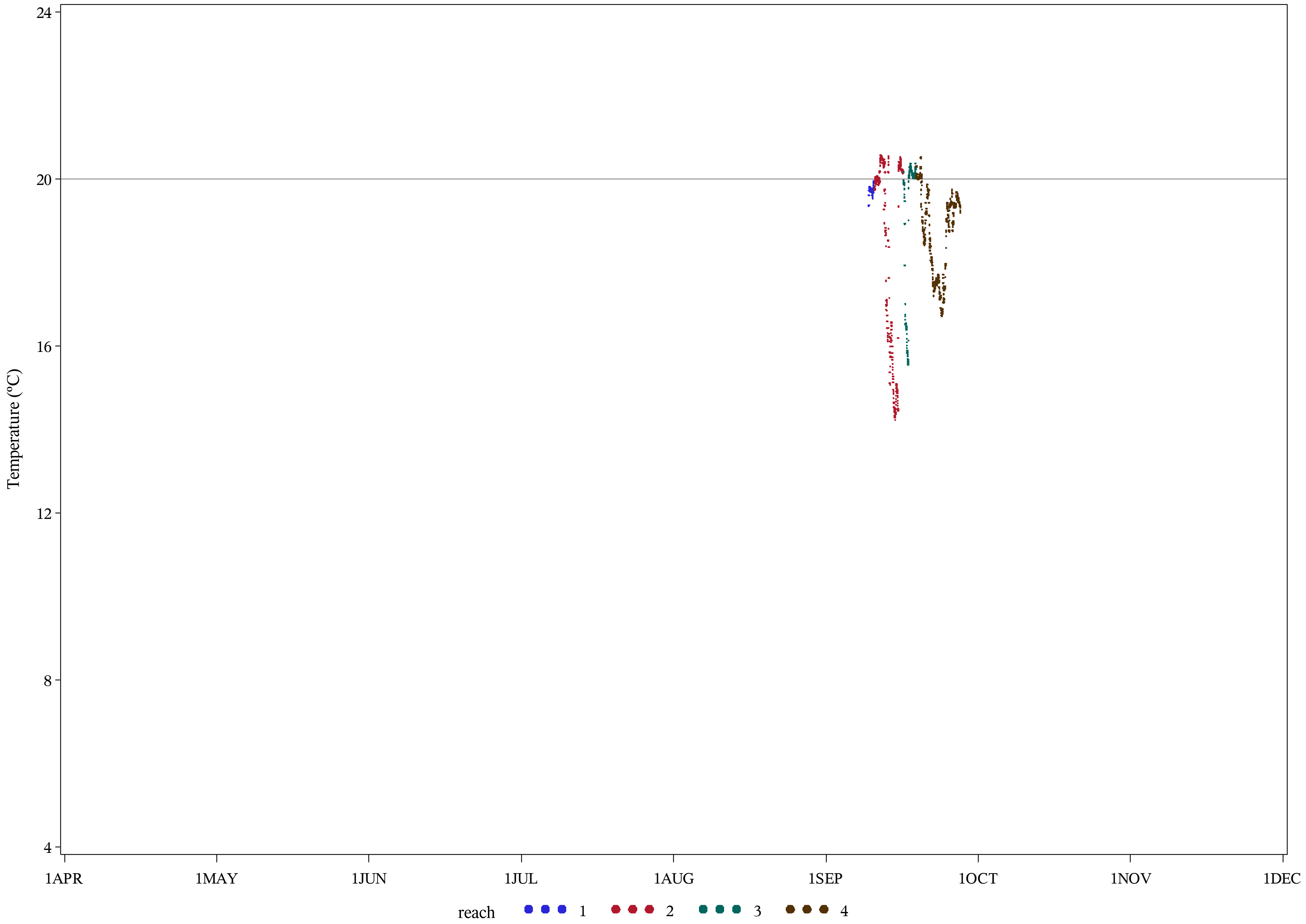

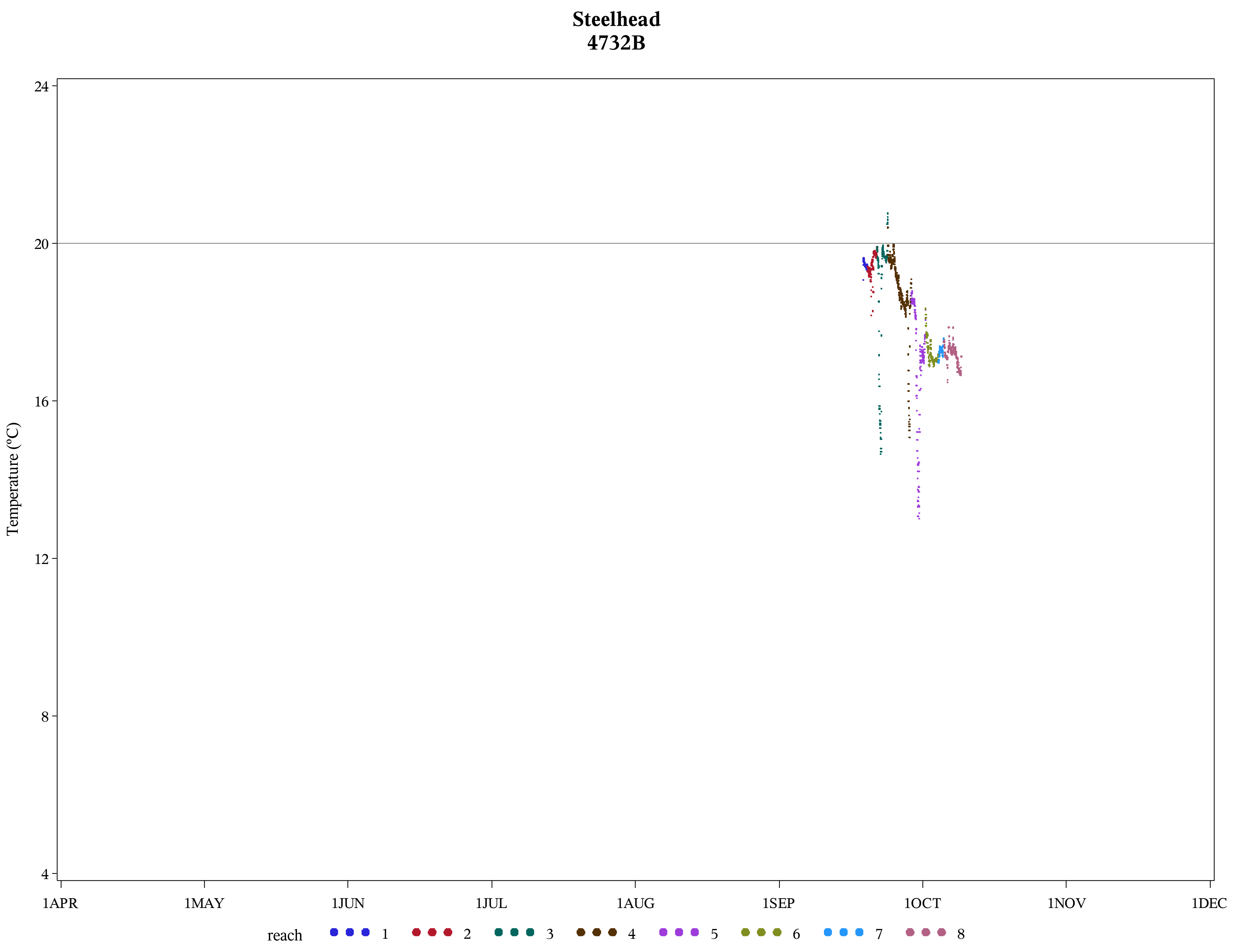

Steelhead
4732B
Temperature (°C)
24
20
16
12
8
4
1APR
1MAY
1JUN
1JUL
1AUG
1SEP
1OCT
1NOV
1DEC
reach
1
2
3
4
5
6
7
8

Steelhead
4734B

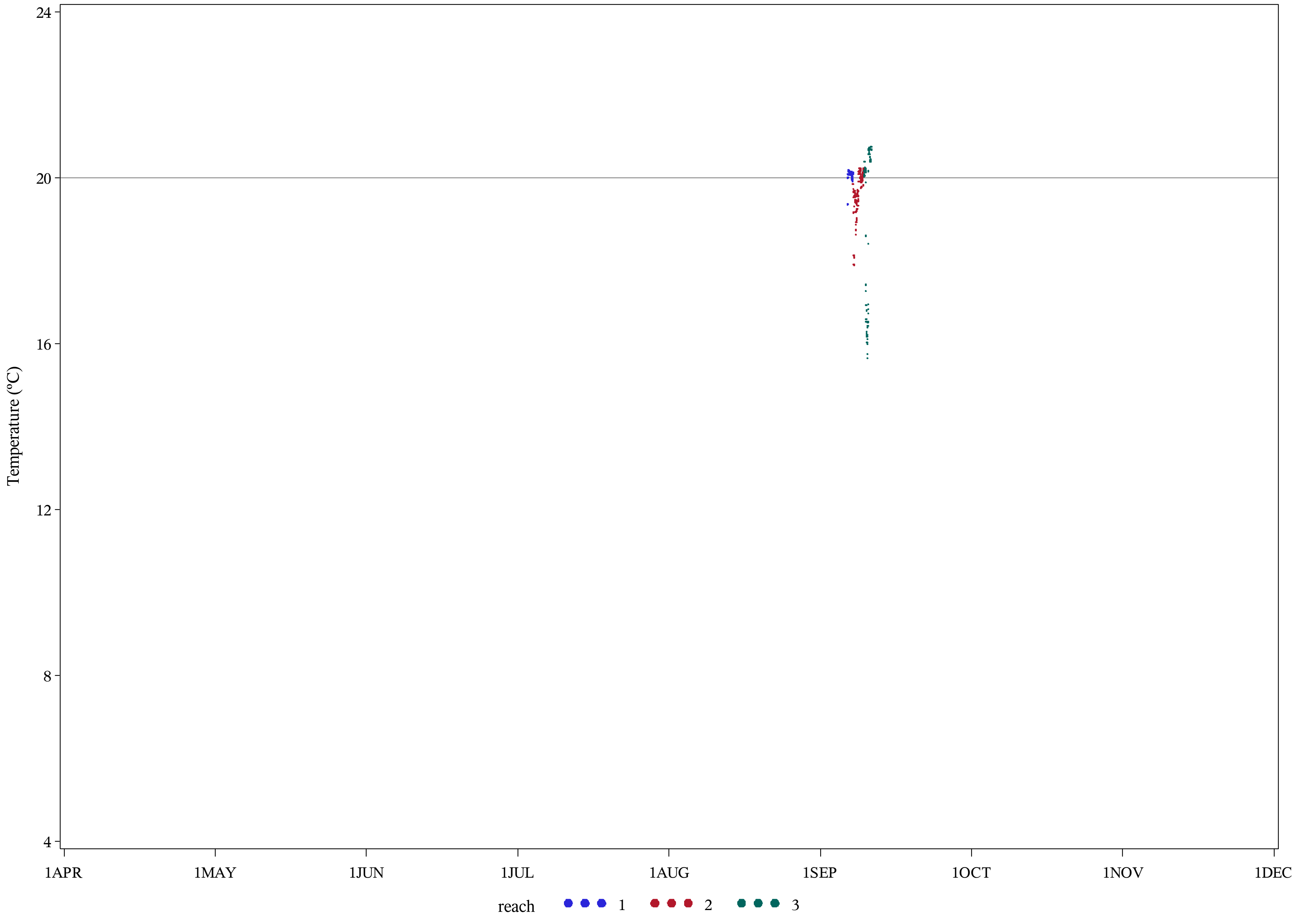

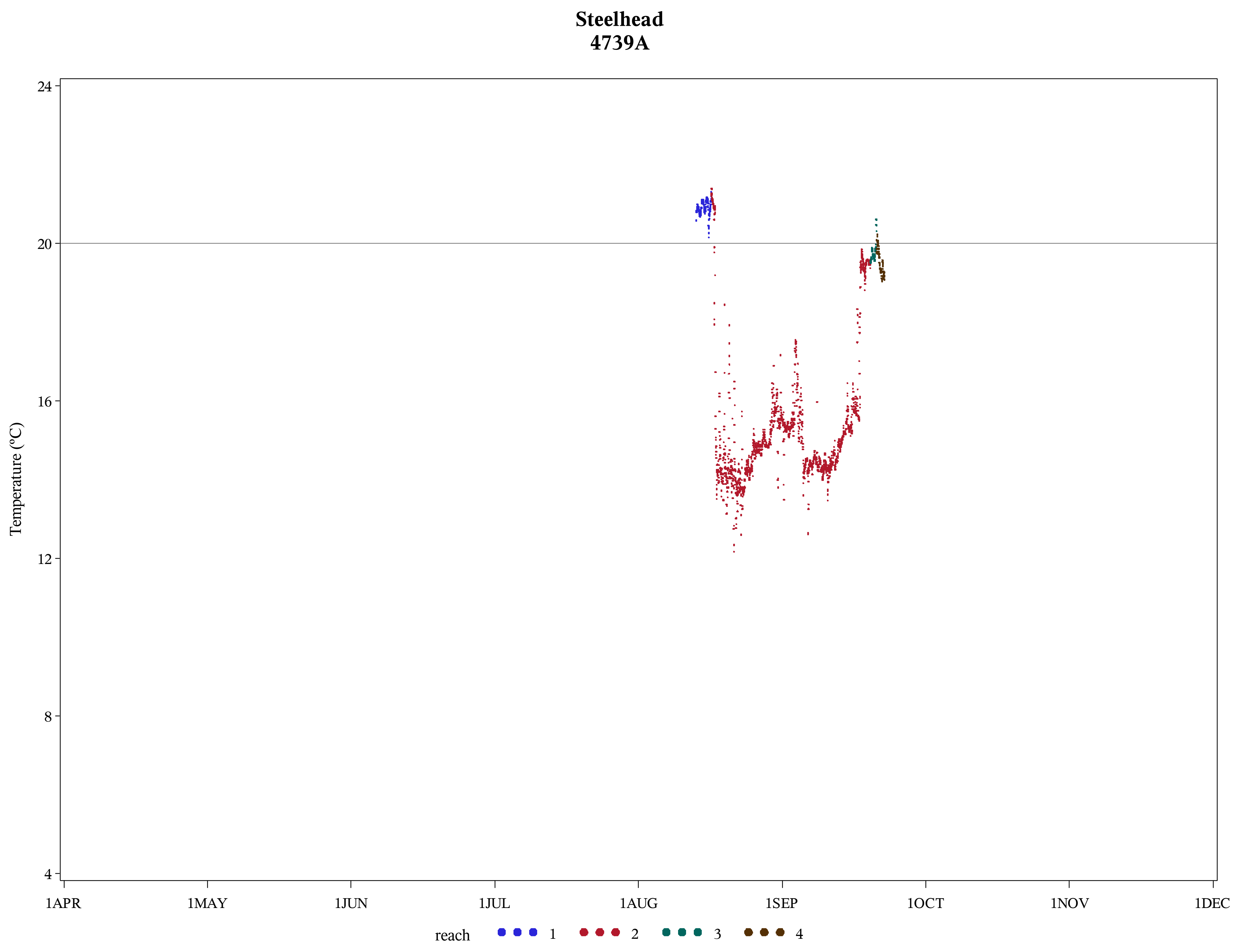

Steelhead
4739A
Temperature (°C)
24
20
16
12
8
4
1APR
1MAY
1JUN
1JUL
1AUG
1SEP
1OCT
1NOV
1DEC
reach
1
2
3
4

Steelhead
4751B

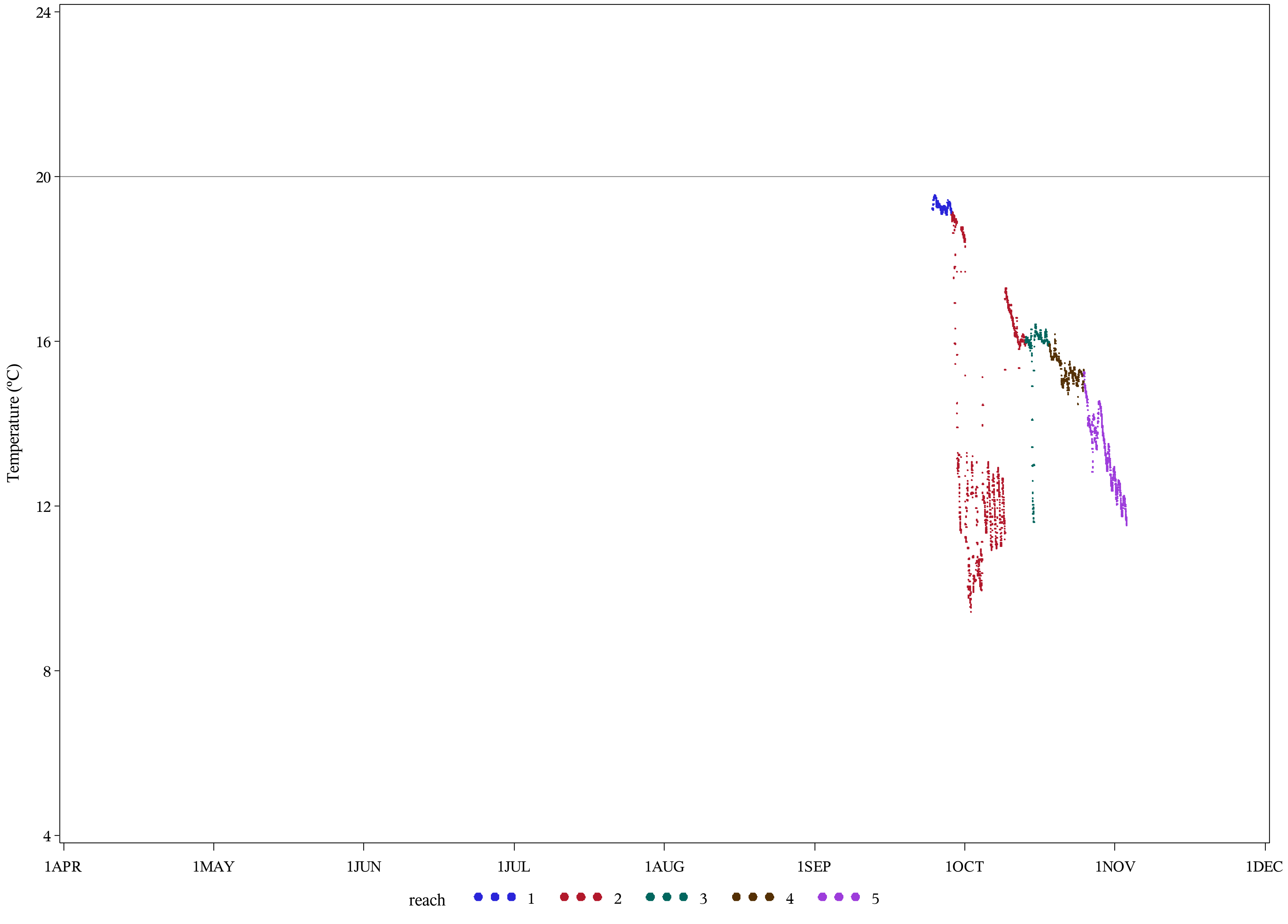

Steelhead
4752B

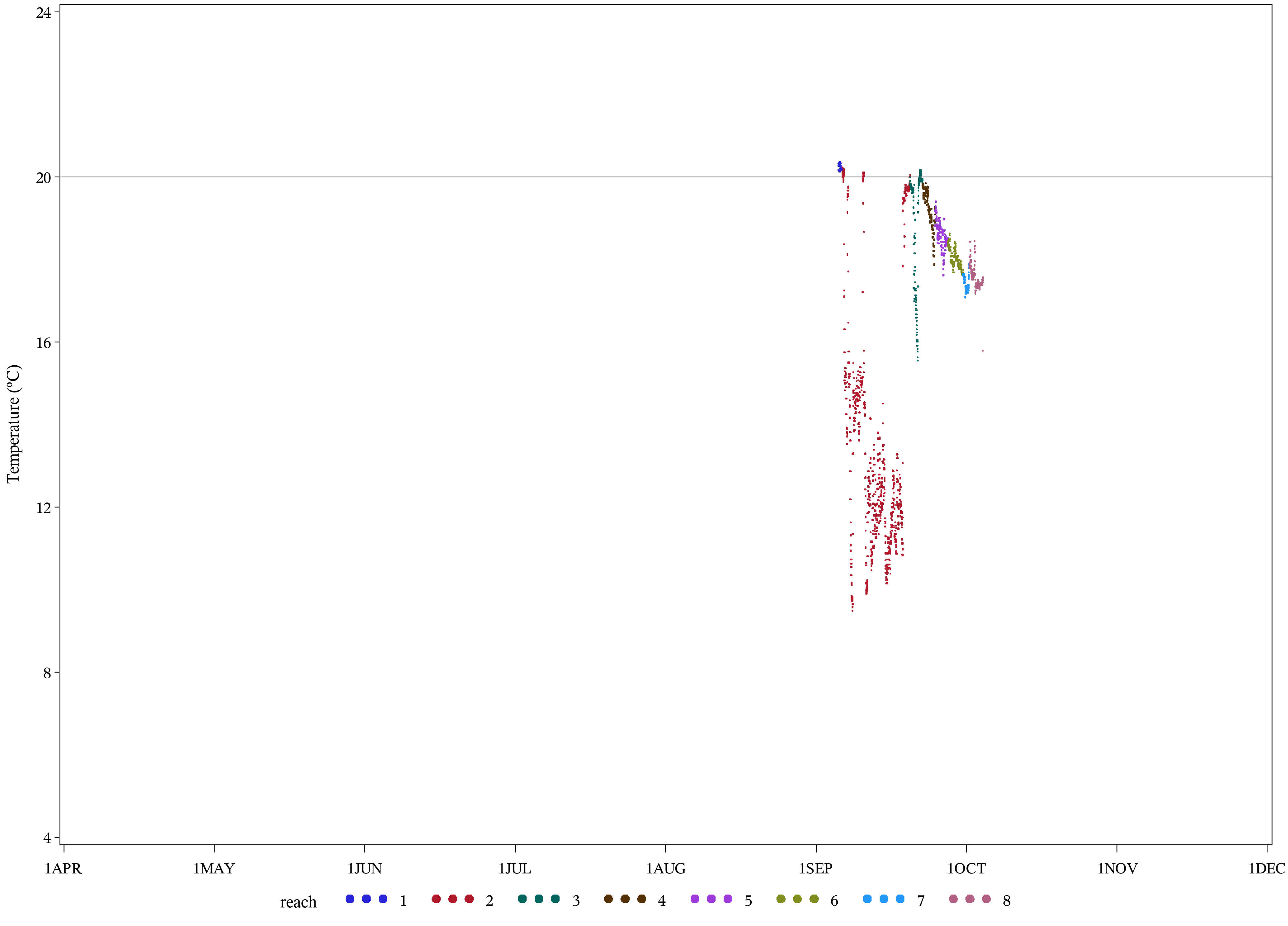

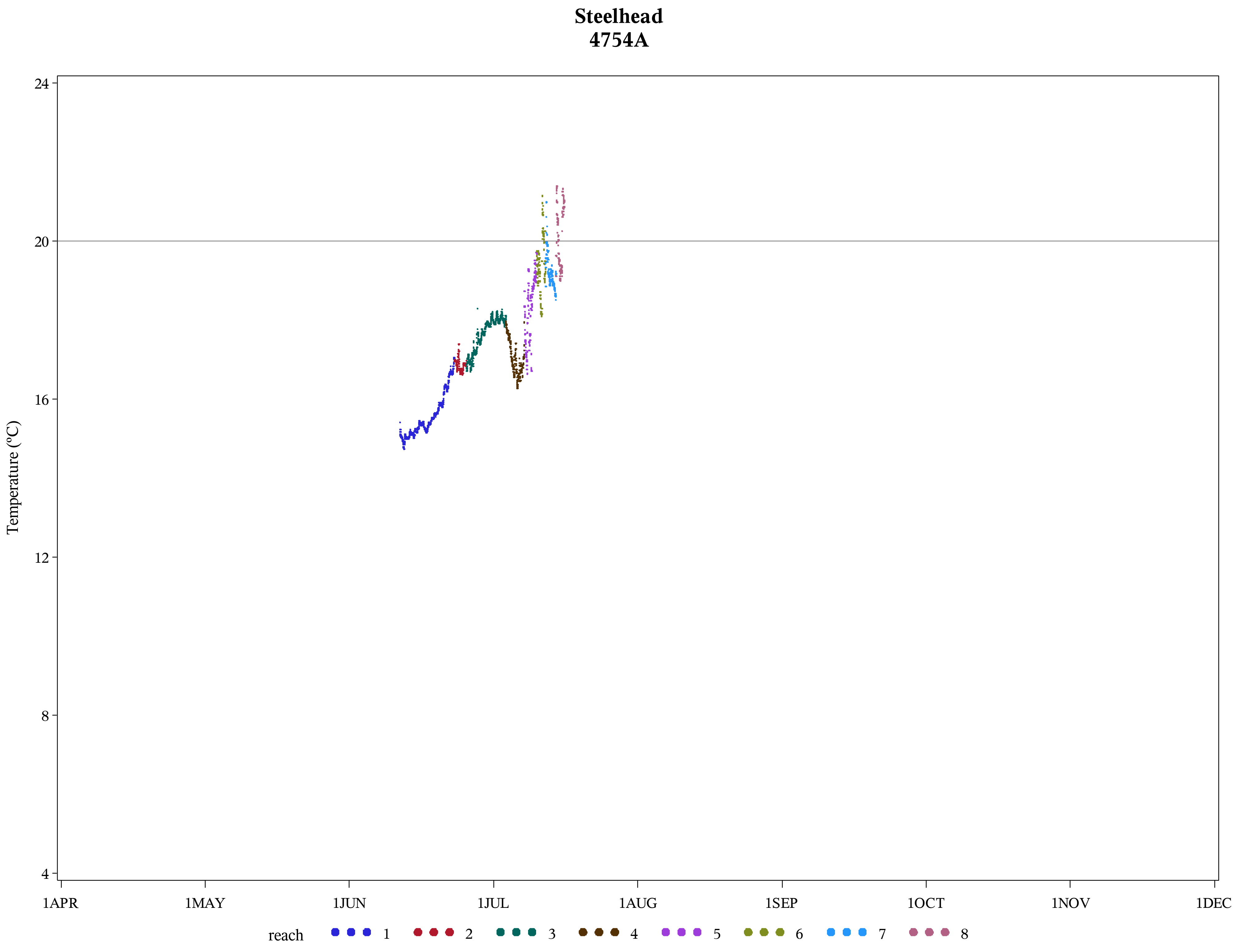
Steelhead
4754A
Temperature (°C)
24
20
16
12
8
4
1APR
1MAY
1JUN
1JUL
1AUG
1SEP
1OCT
1NOV
1DEC
reach
1
2
3
4
5
6
7
8

Steelhead
4759B

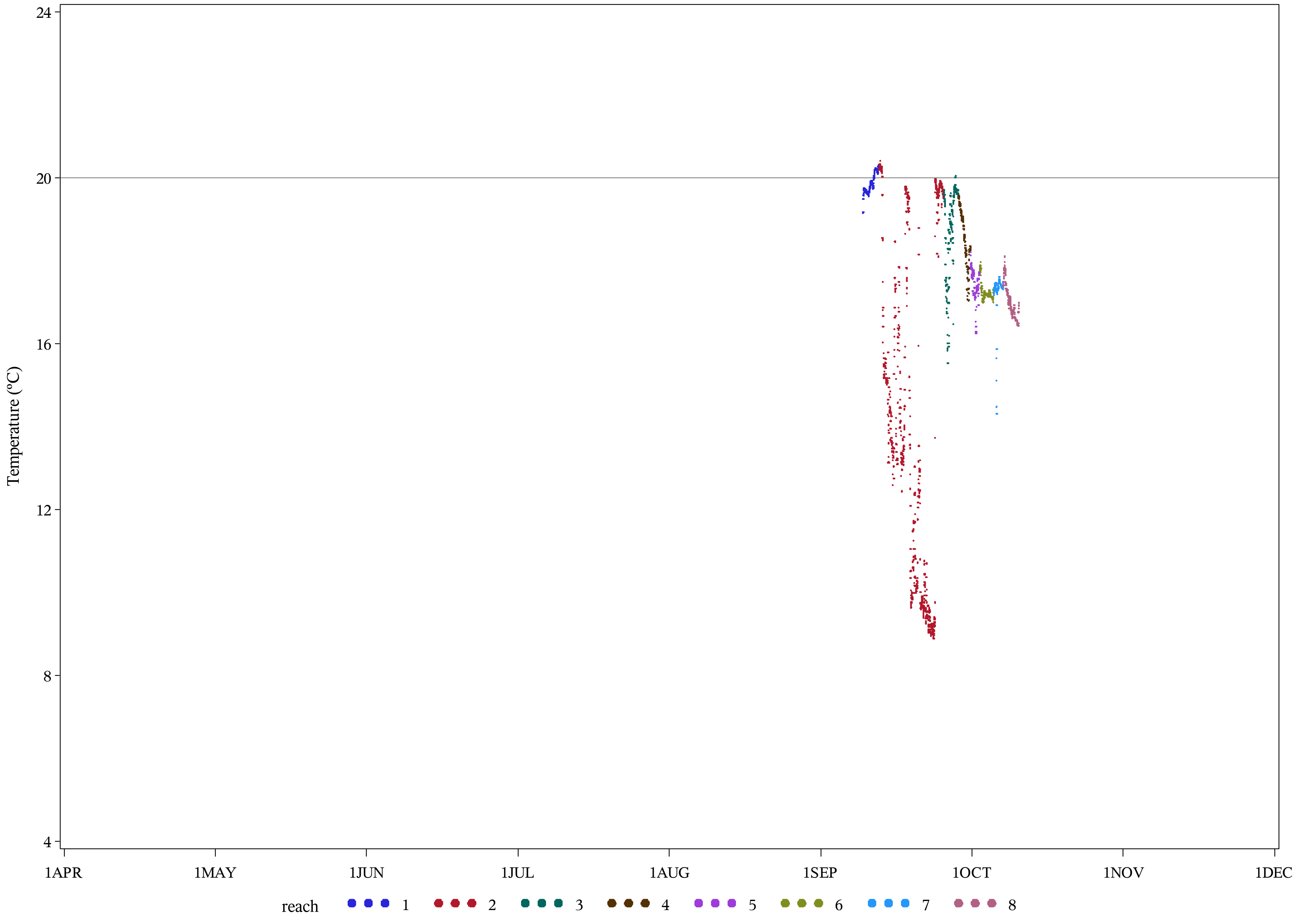

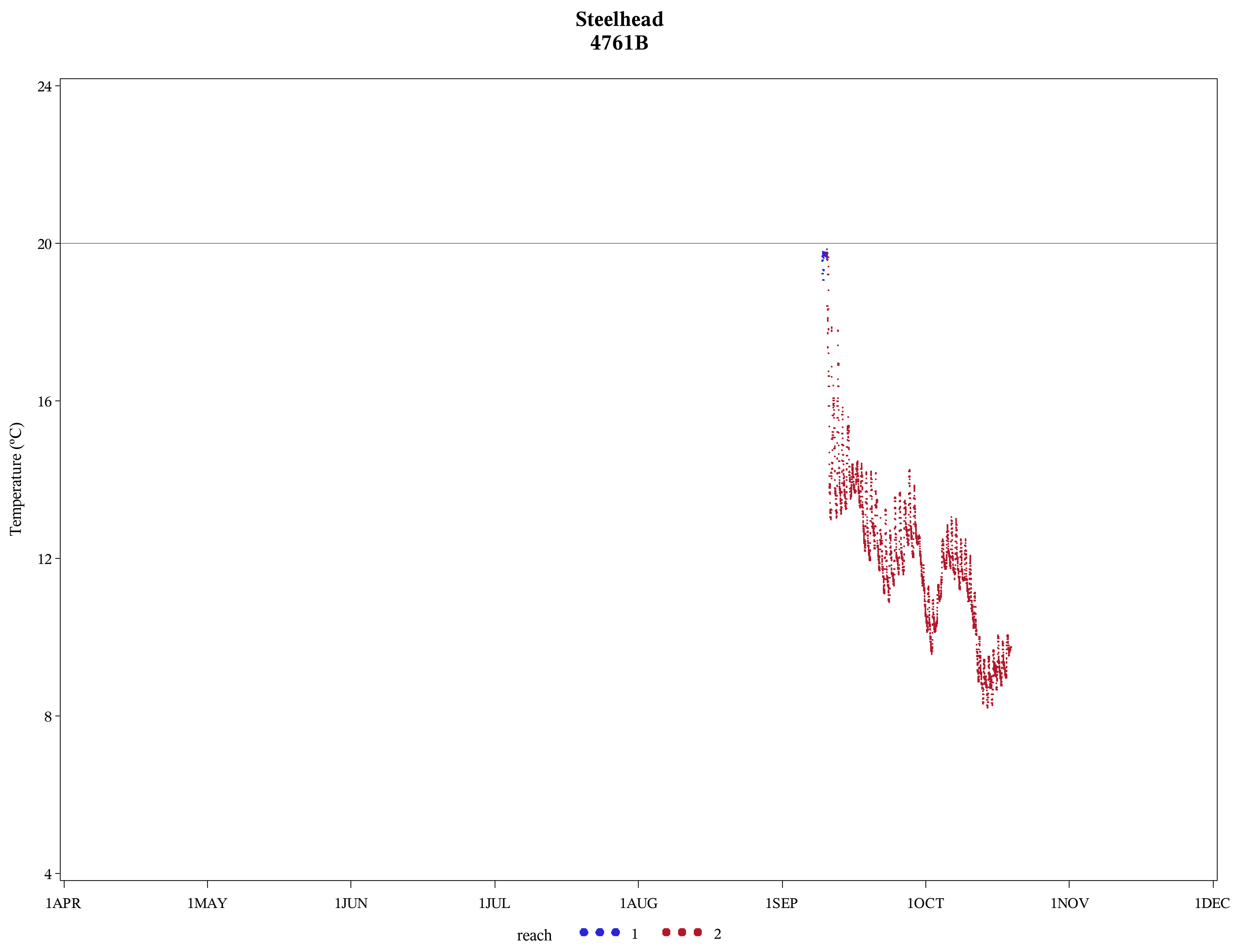

Steelhead
4761B
Temperature (°C)
24
20
16
12
8
4
1APR
1MAY
1JUN
1JUL
1AUG
1SEP
1OCT
1NOV
1DEC
reach
1
2

Steelhead
4763B

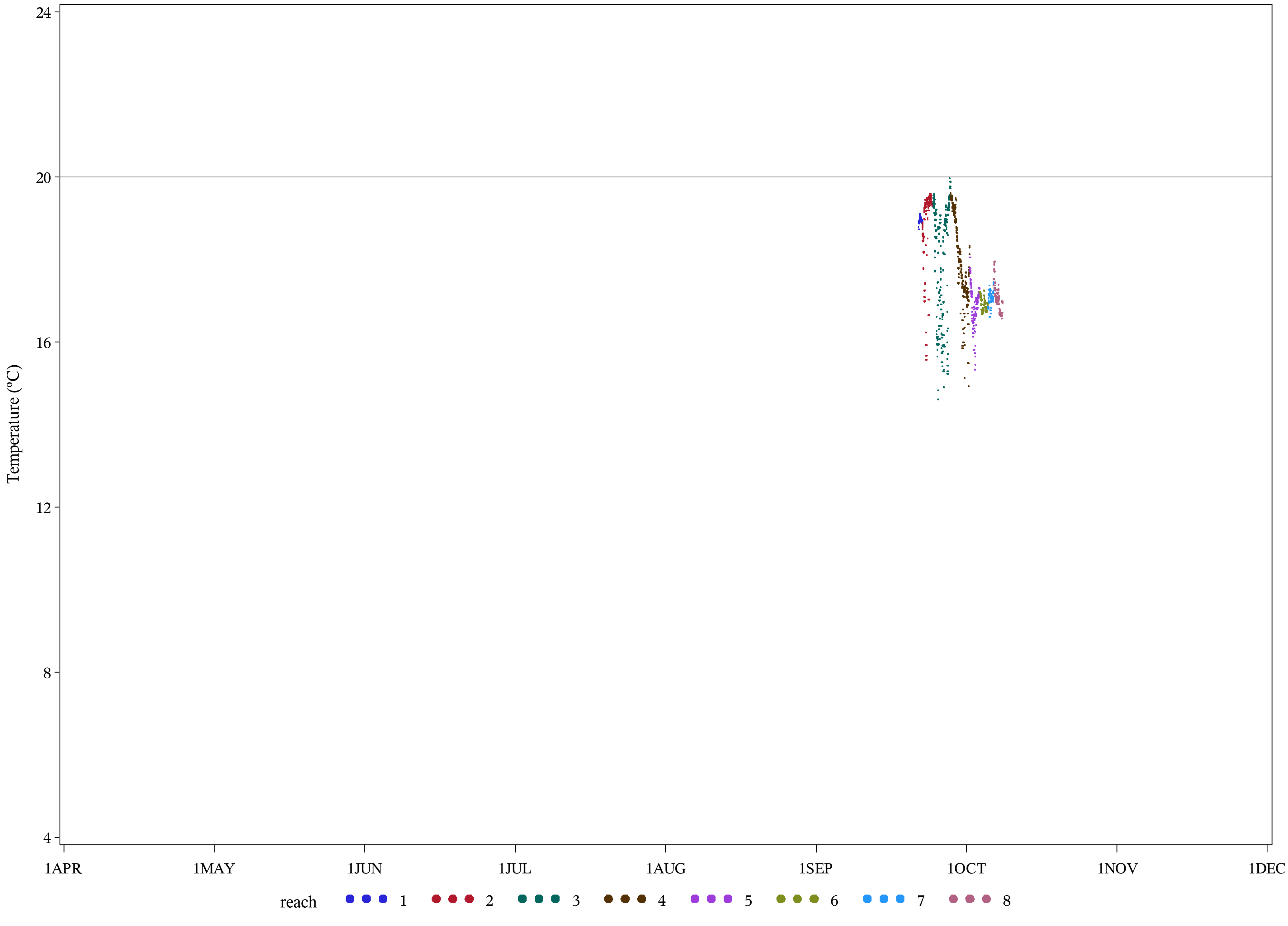

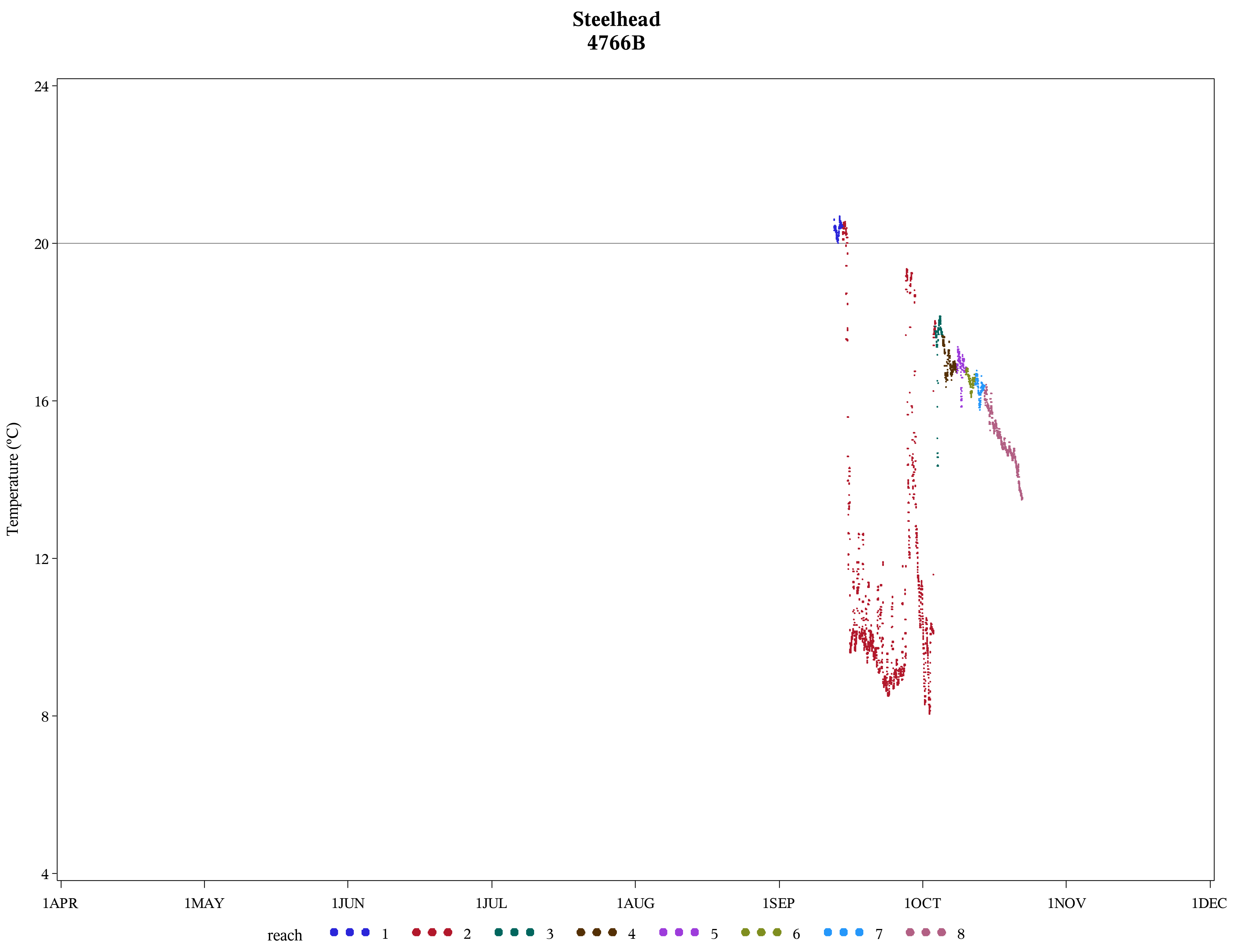
Steelhead
4766B
Temperature (°C)
24
20
16
12
8
4
1APR
1MAY
1JUN
1JUL
1AUG
1SEP
1OCT
1NOV
1DEC
reach
1
2
3
4
5
6
7
8

Steelhead
4768A

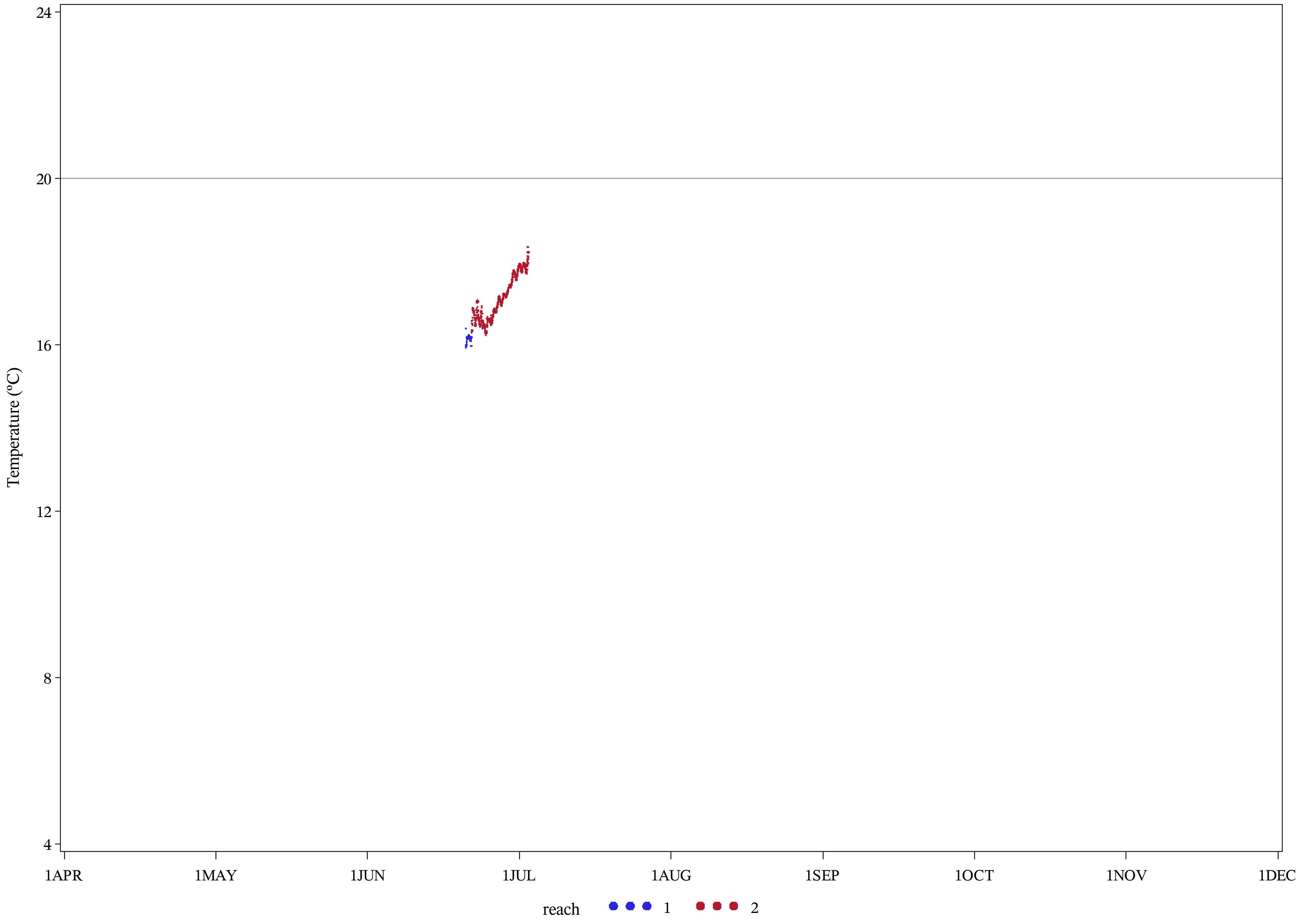

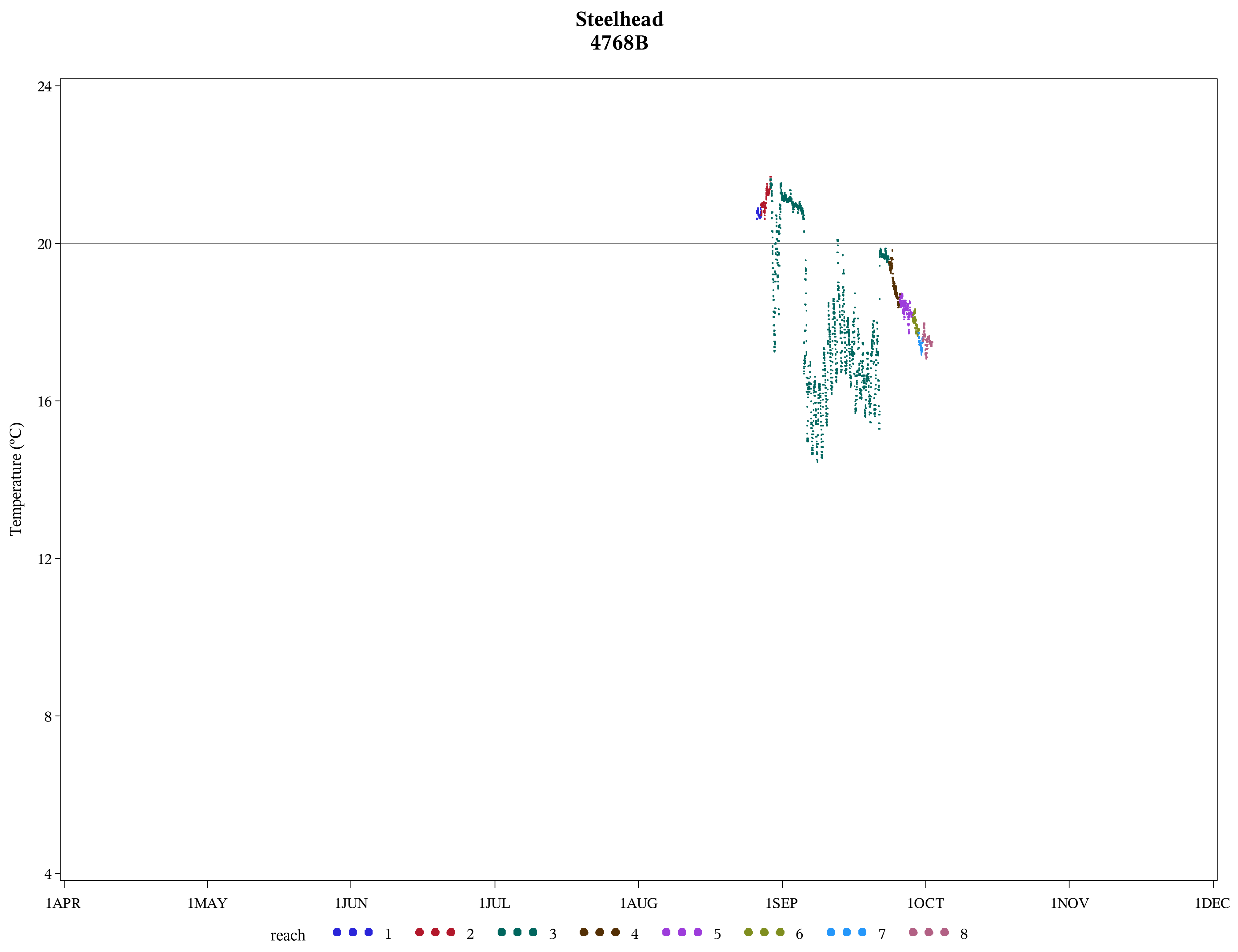

Steelhead
4768B
Temperature (°C)
24
20
16
12
8
4
1APR
1MAY
1JUN
1JUL
1AUG
1SEP
1OCT
1NOV
1DEC
reach
1
2
3
4
5
6
7
8

Steelhead
4771A

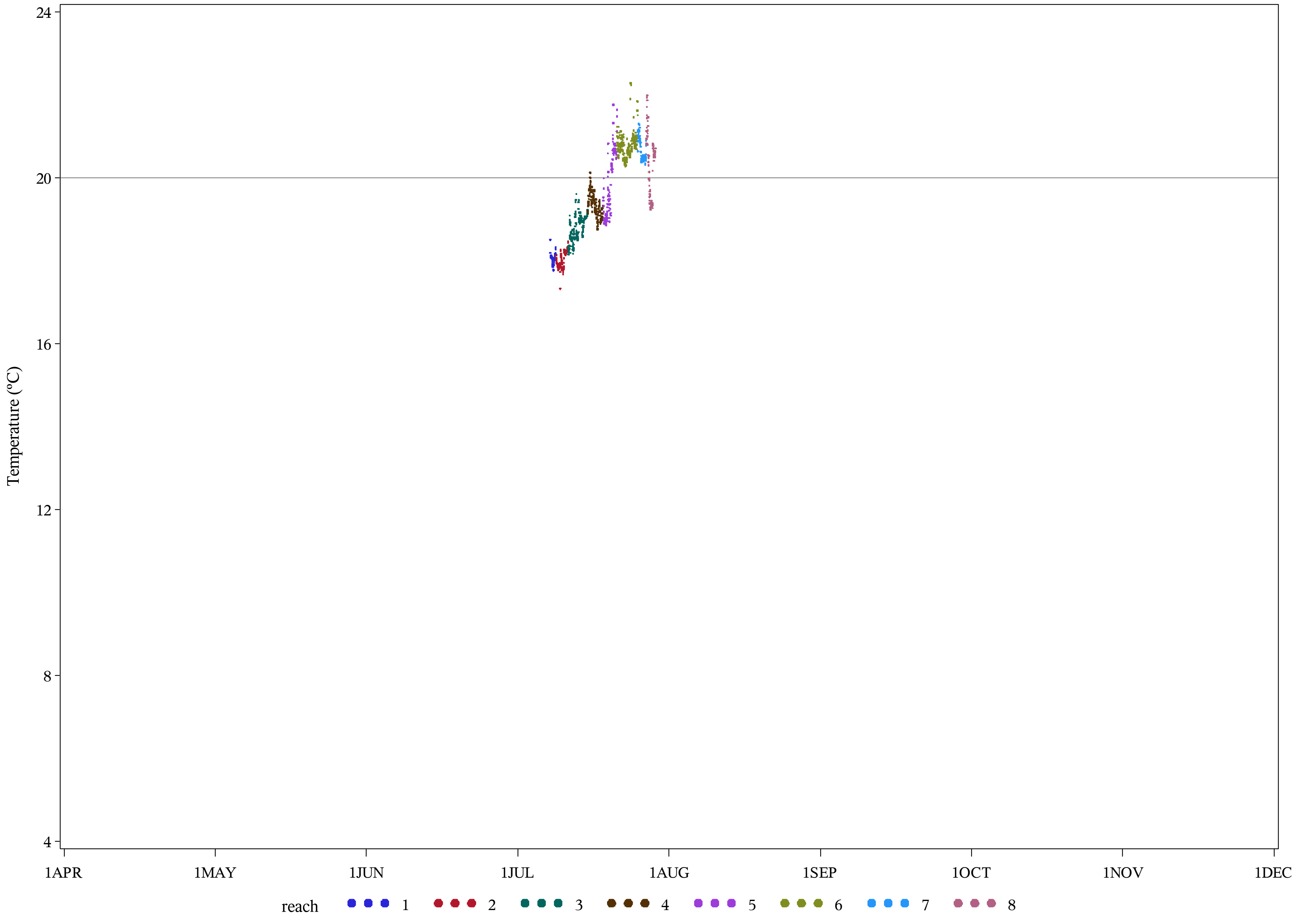

Steelhead
4777A

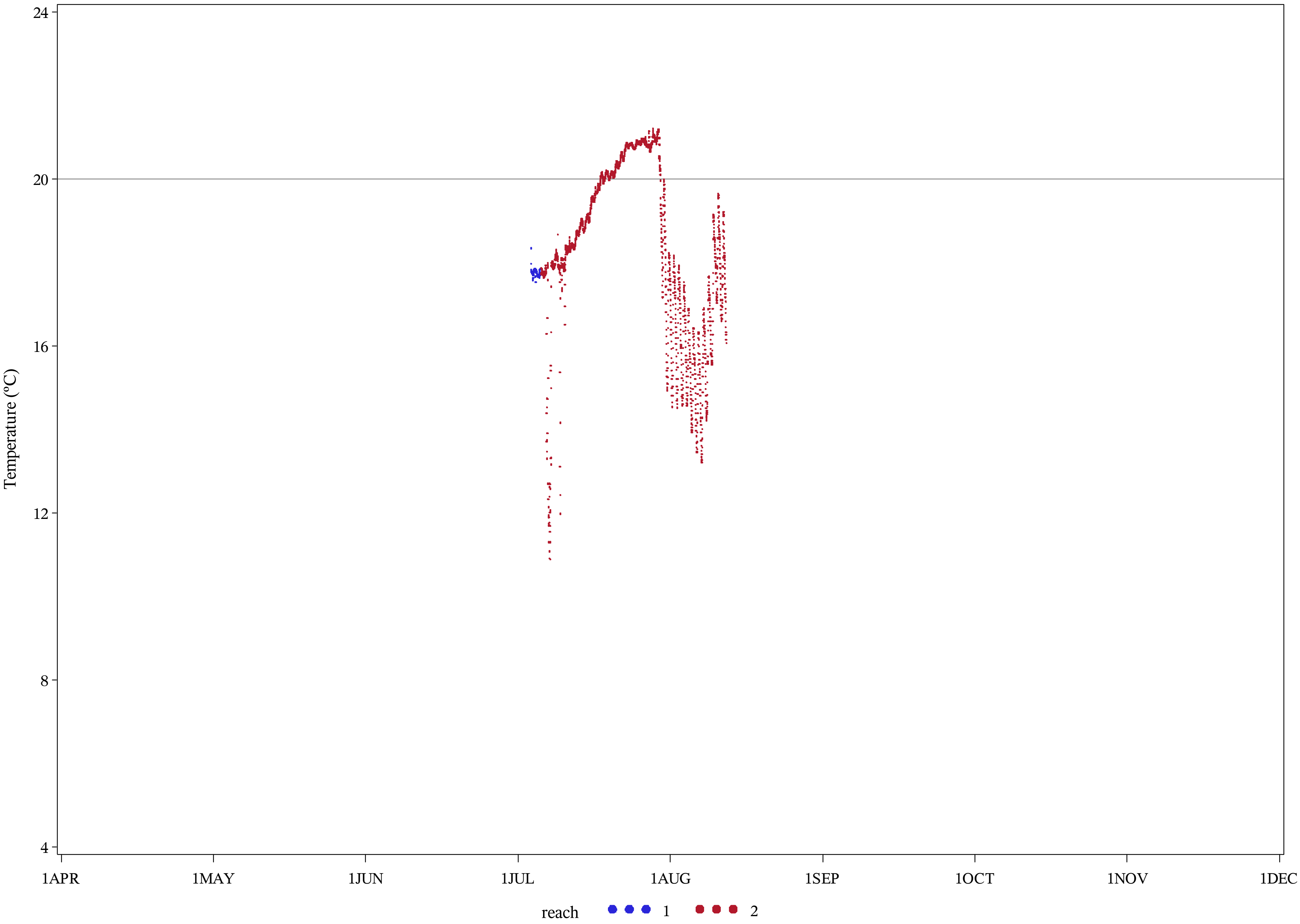

Steelhead
4777B

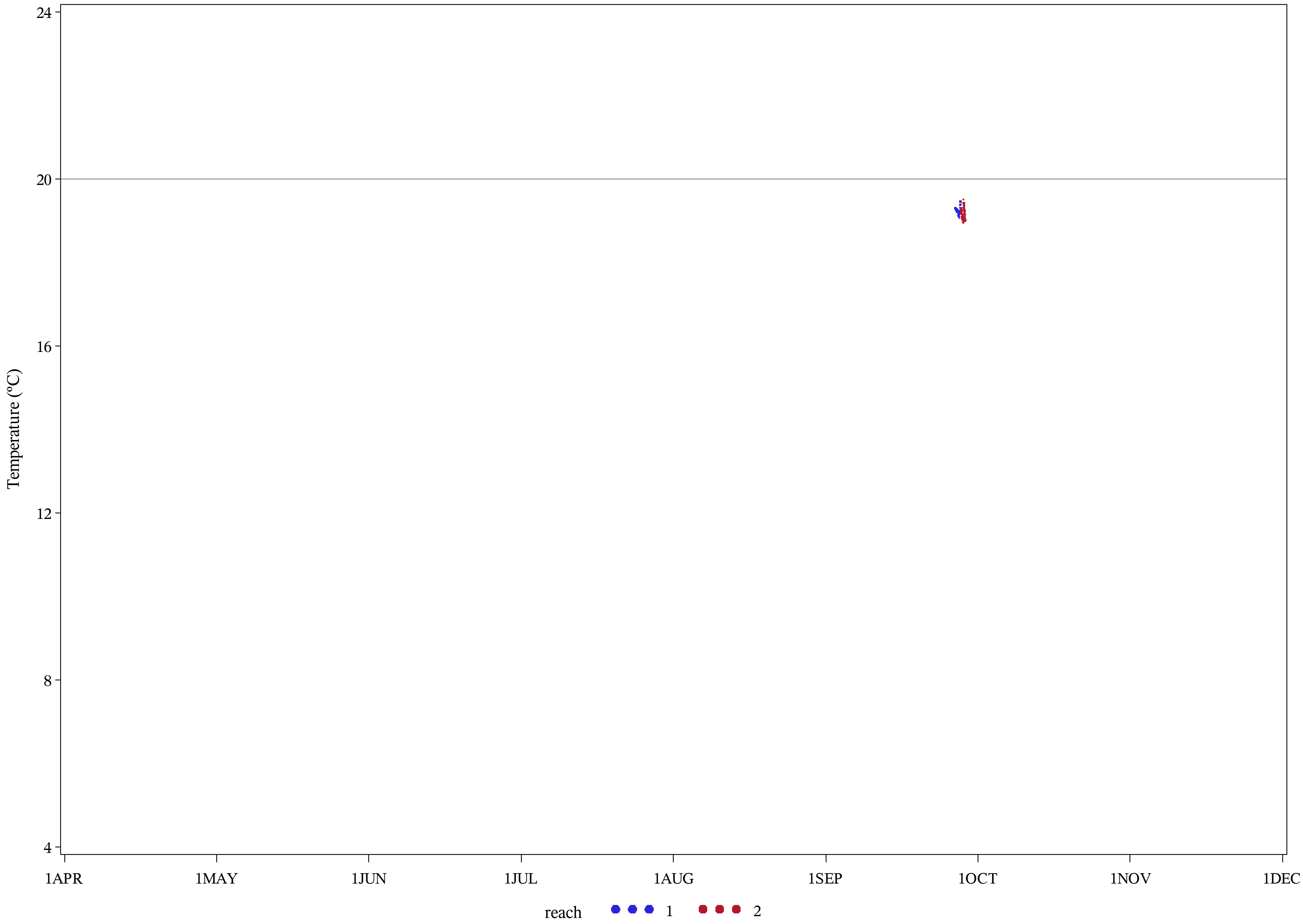

Steelhead
4778A

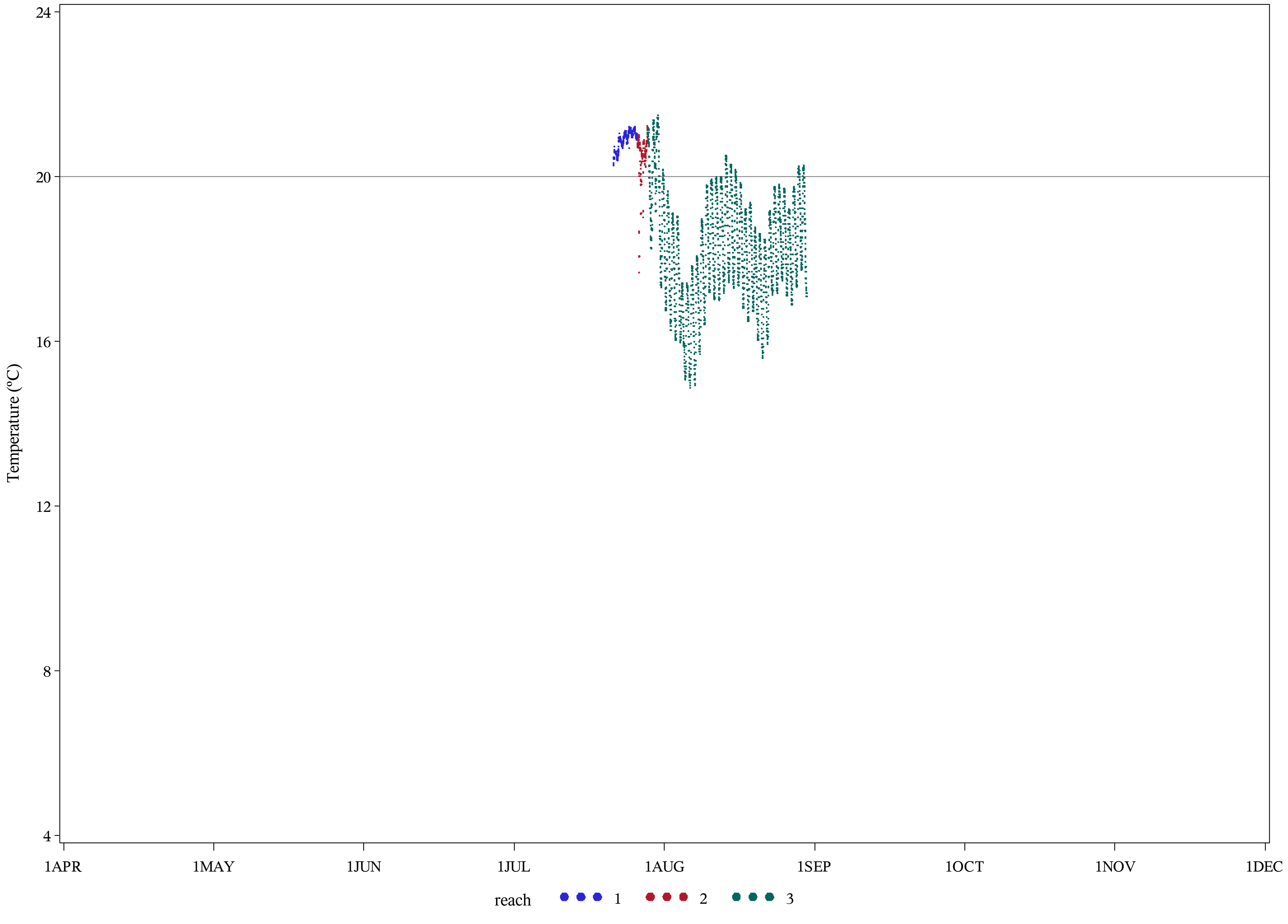

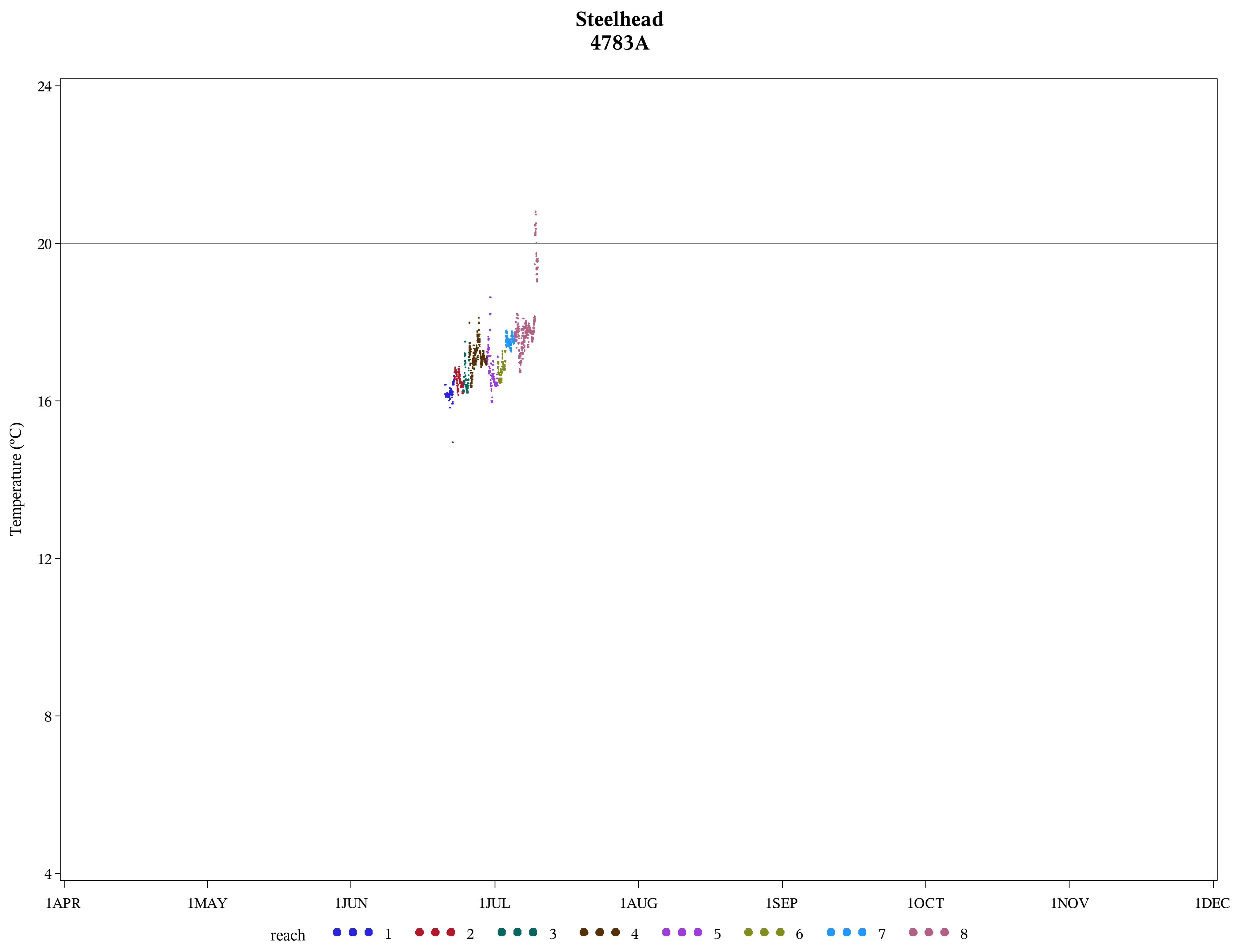

Steelhead
4783A
Temperature (°C)
24
20
16
12
8
4
1APR
1MAY
1JUN
1JUL
1AUG
1SEP
1OCT
1NOV
1DEC
reach
1
2
3
4
5
6
7
8

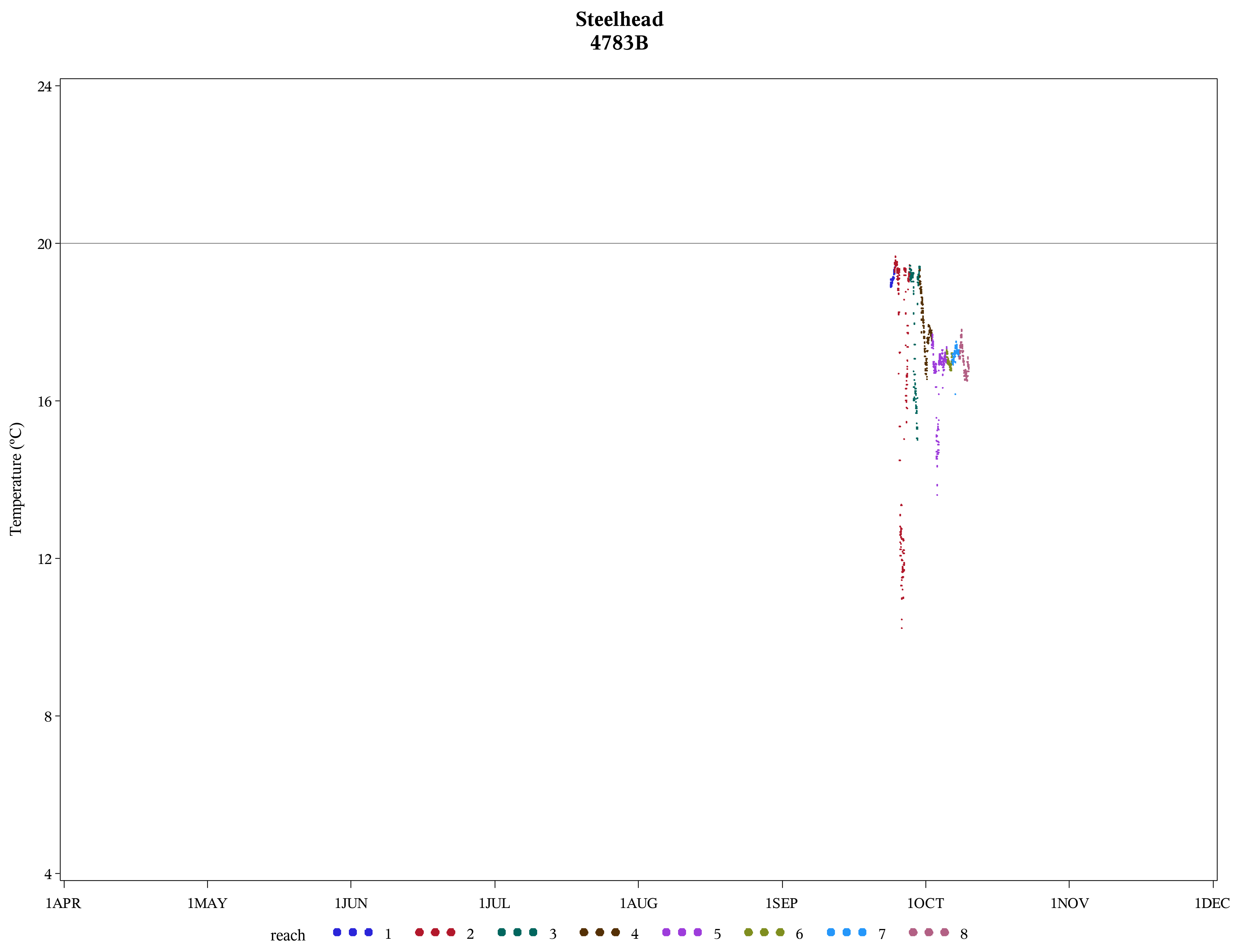

Steelhead
4783B
Temperature (°C)
24
20
16
12
8
4
1APR
1MAY
1JUN
1JUL
1AUG
1SEP
1OCT
1NOV
1DEC
reach
1
2
3
4
5
6
7
8

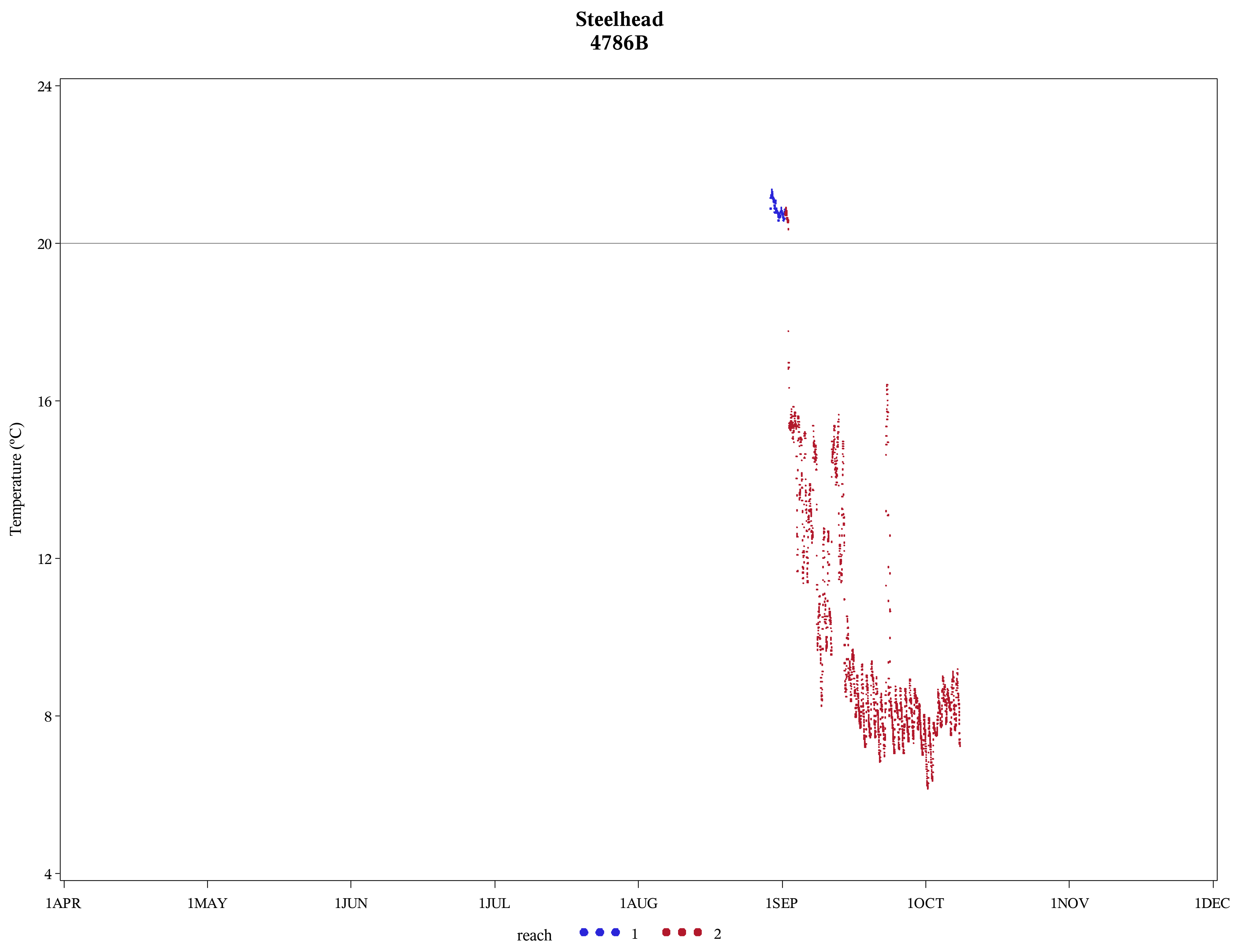
Steelhead
4786B
Temperature (°C)
24
20
16
12
8
4
1APR
1MAY
1JUN
1JUL
1AUG
1SEP
1OCT
1NOV
1DEC
reach
1
2

Steelhead
4787A

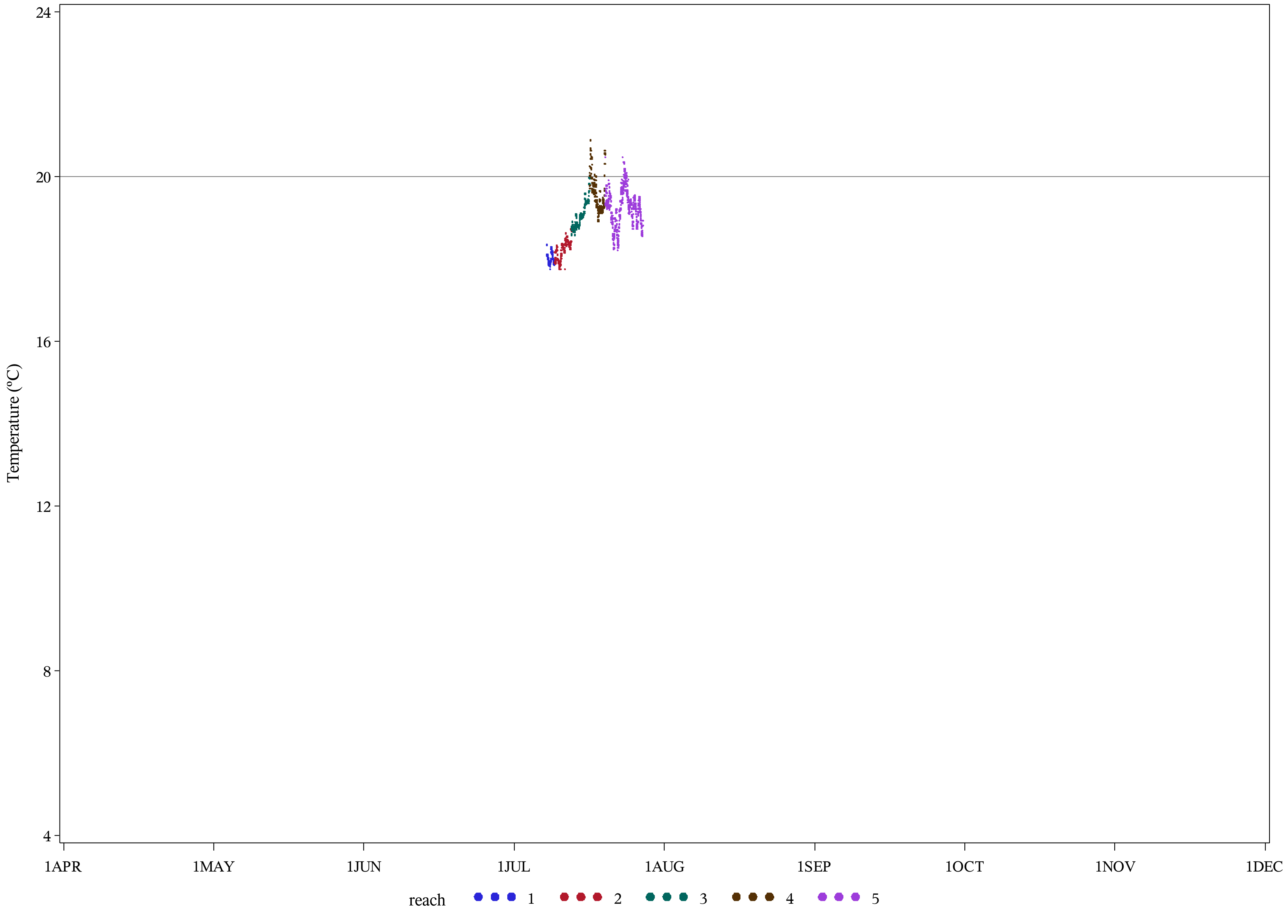

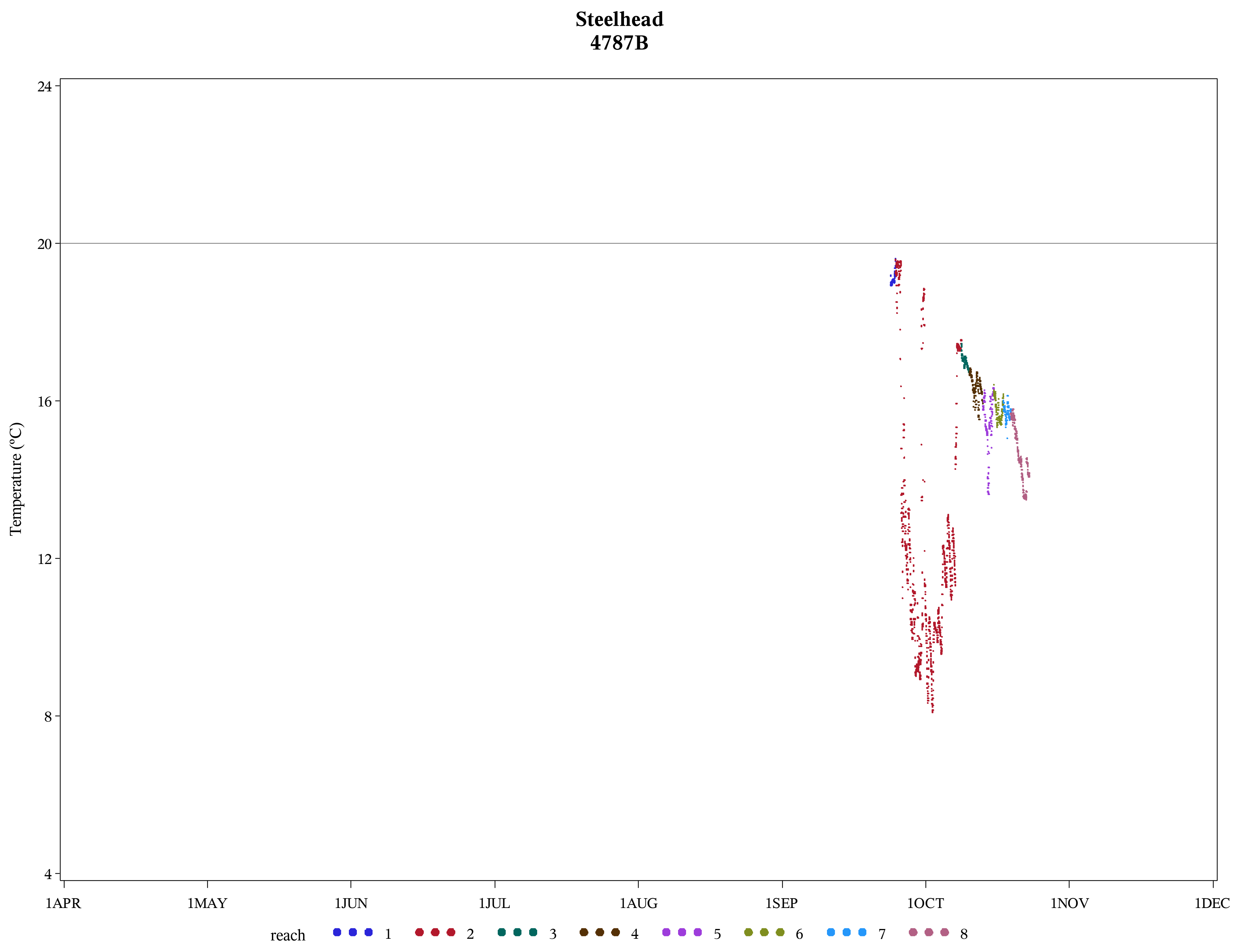

Steelhead
4787B
Temperature (°C)
24
20
16
12
8
4
1APR
1MAY
1JUN
1JUL
1AUG
1SEP
1OCT
1NOV
1DEC
reach
1
2
3
4
5
6
7
8

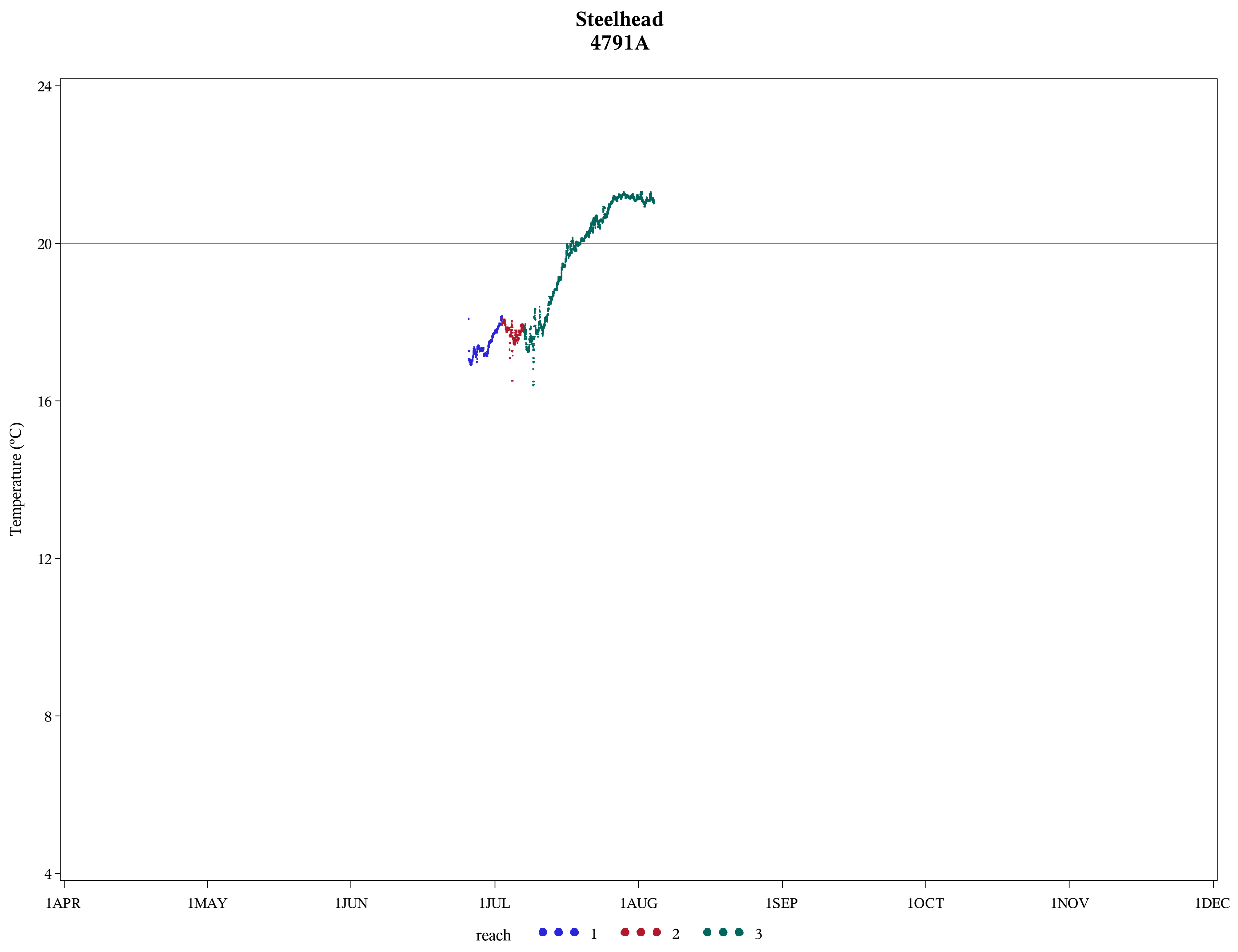
Steelhead
4791A
Temperature (°C)
24
20
16
12
8
4
1APR
1MAY
1JUN
1JUL
1AUG
1SEP
1OCT
1NOV
1DEC
reach
1
2
3

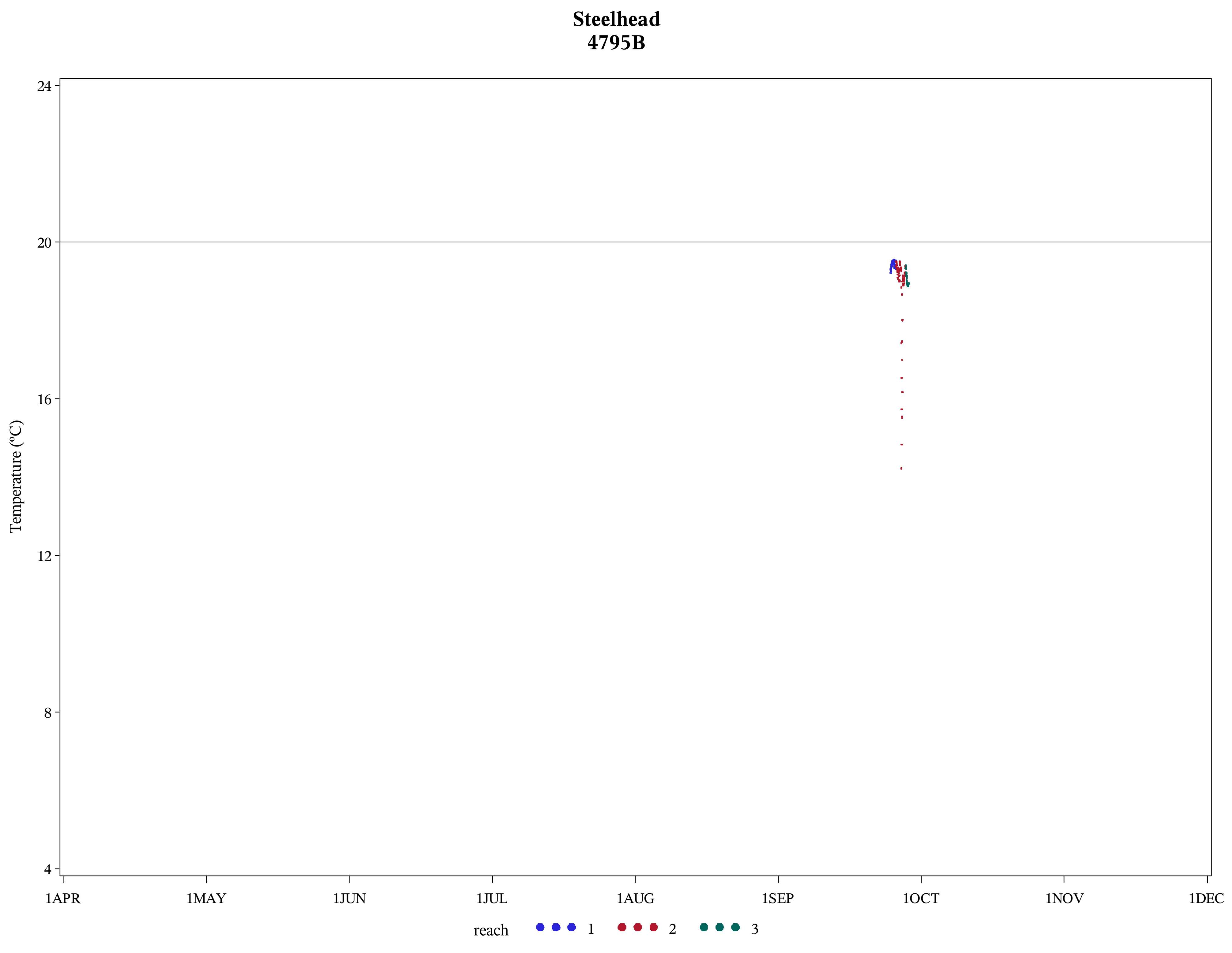

Steelhead
4795B
24
20
16
12
8
4
Temperature (°C)
1APR
1MAY
1JUN
1JUL
1AUG
1SEP
1OCT
1NOV
1DEC
reach
1
2
3

Steelhead
4809B

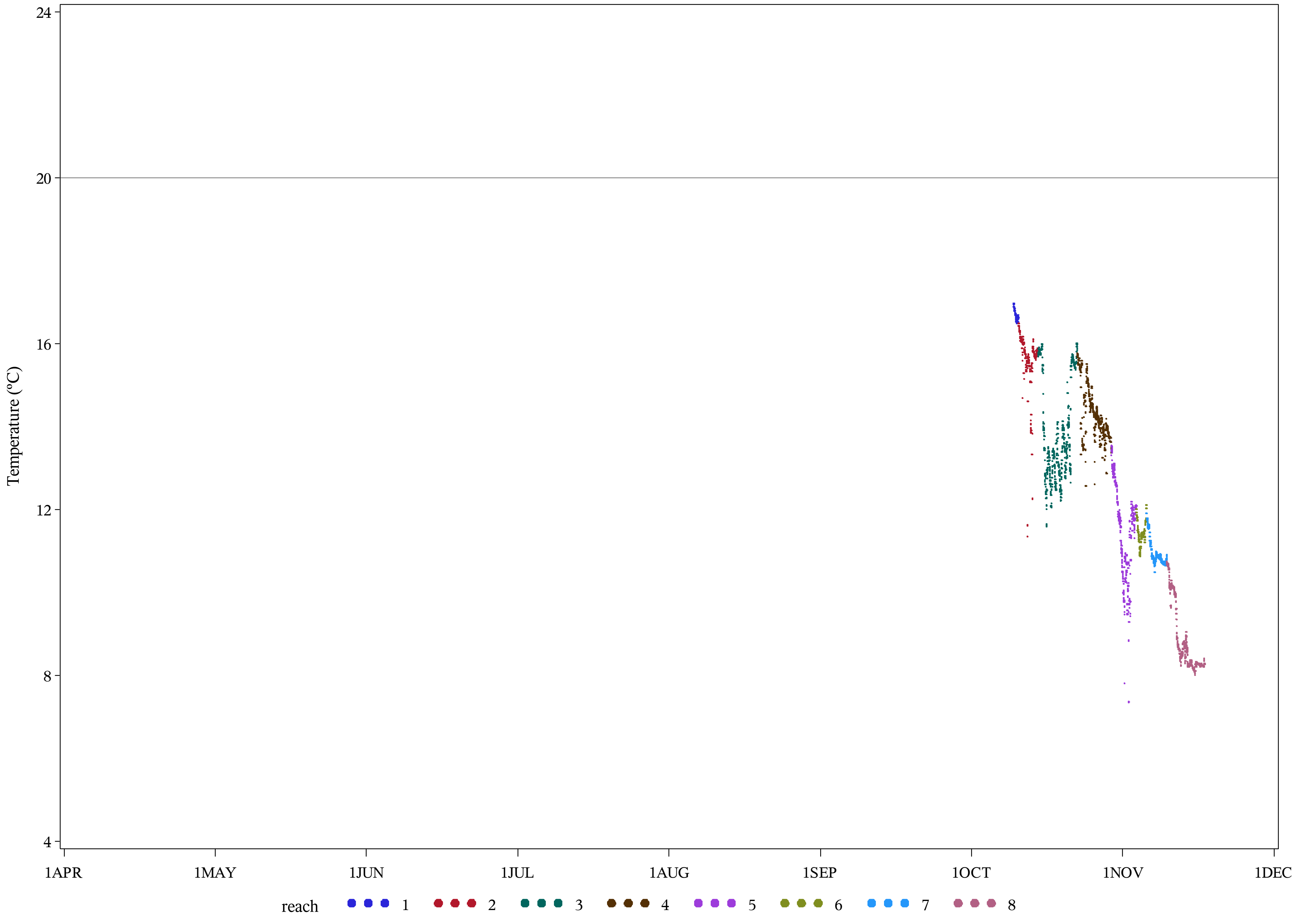

Steelhead
4813B

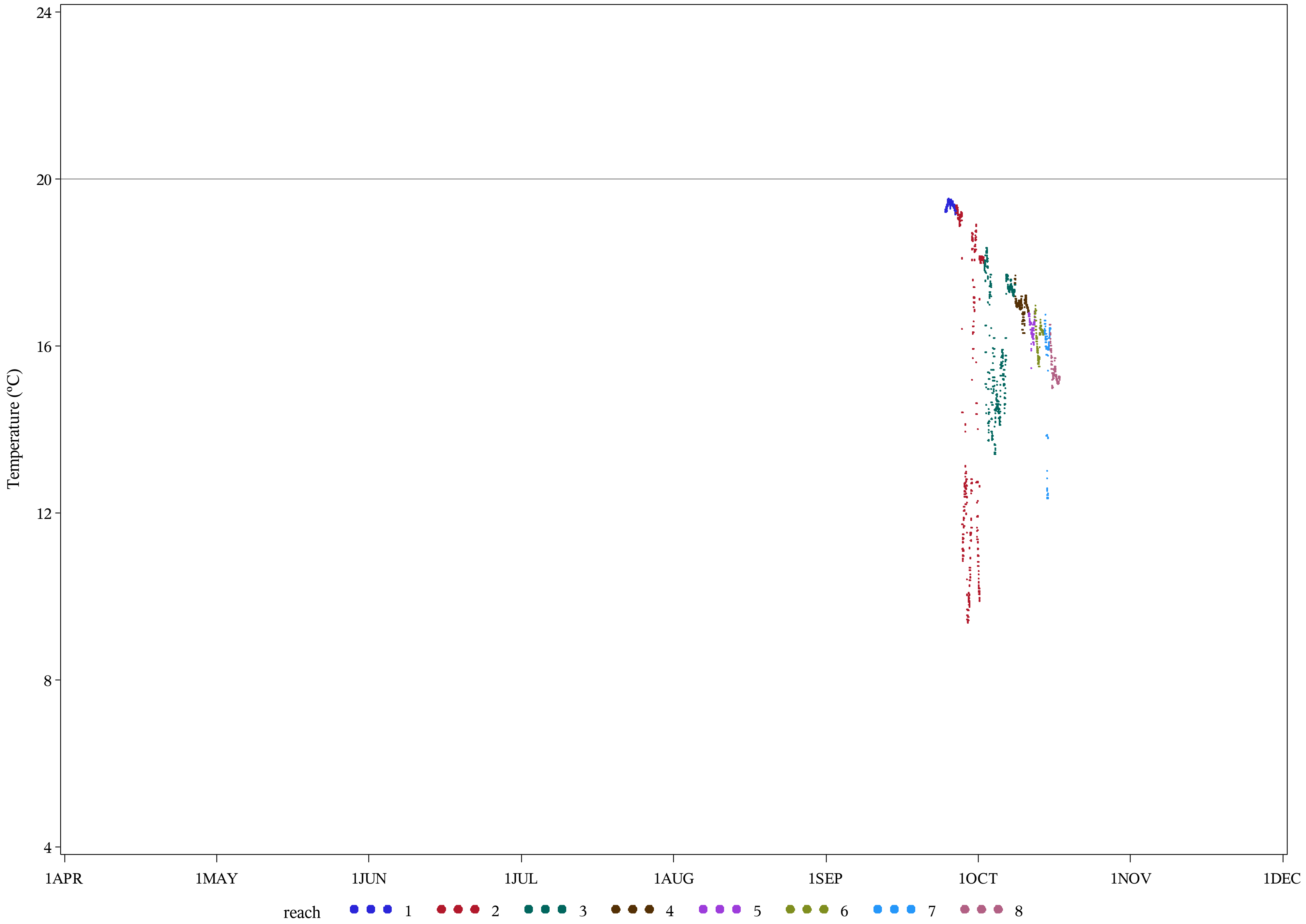

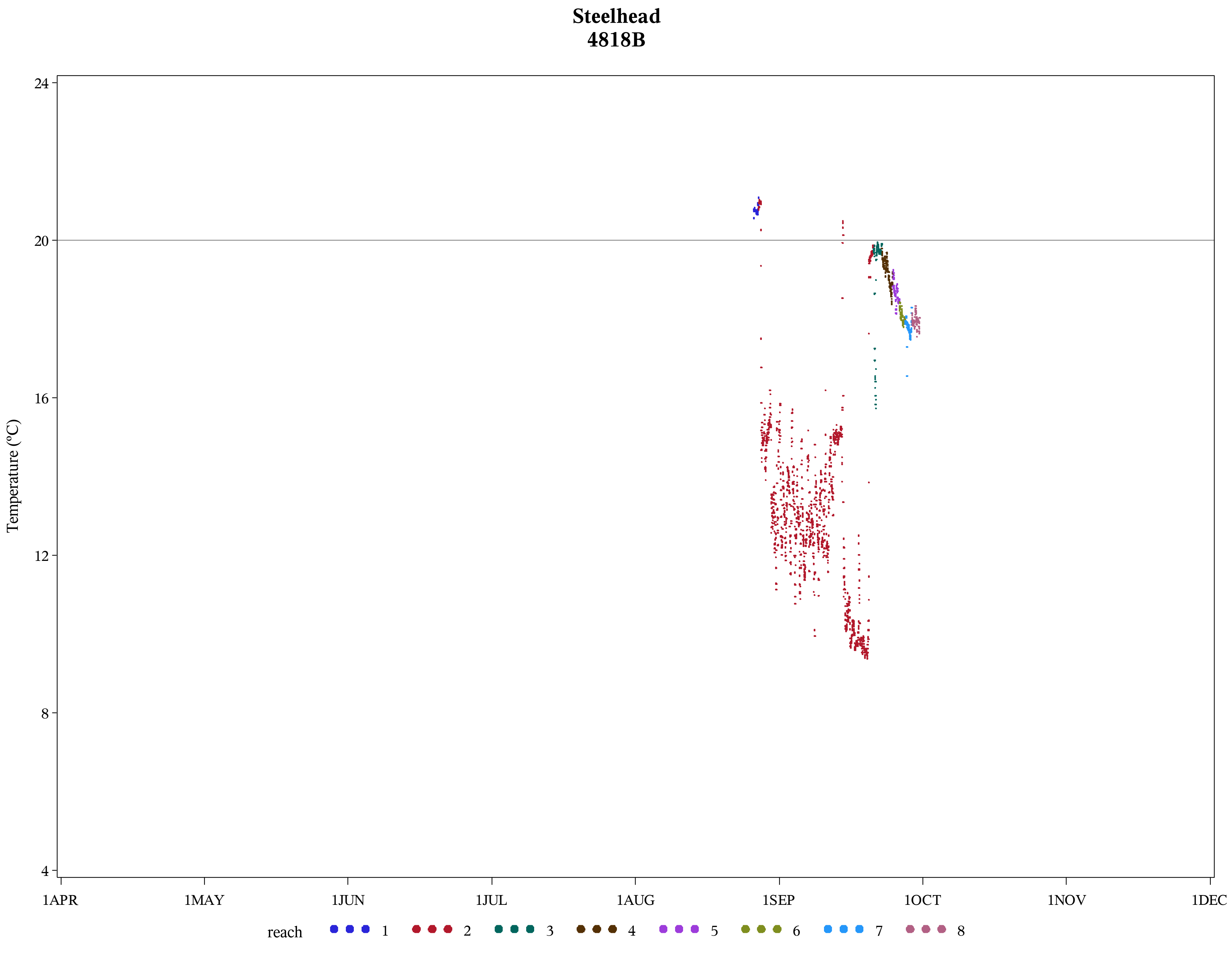

Steelhead
4818B
Temperature (°C)
24
20
16
12
8
4
1APR
1MAY
1JUN
1JUL
1AUG
1SEP
1OCT
1NOV
1DEC
reach
1
2
3
4
5
6
7
8

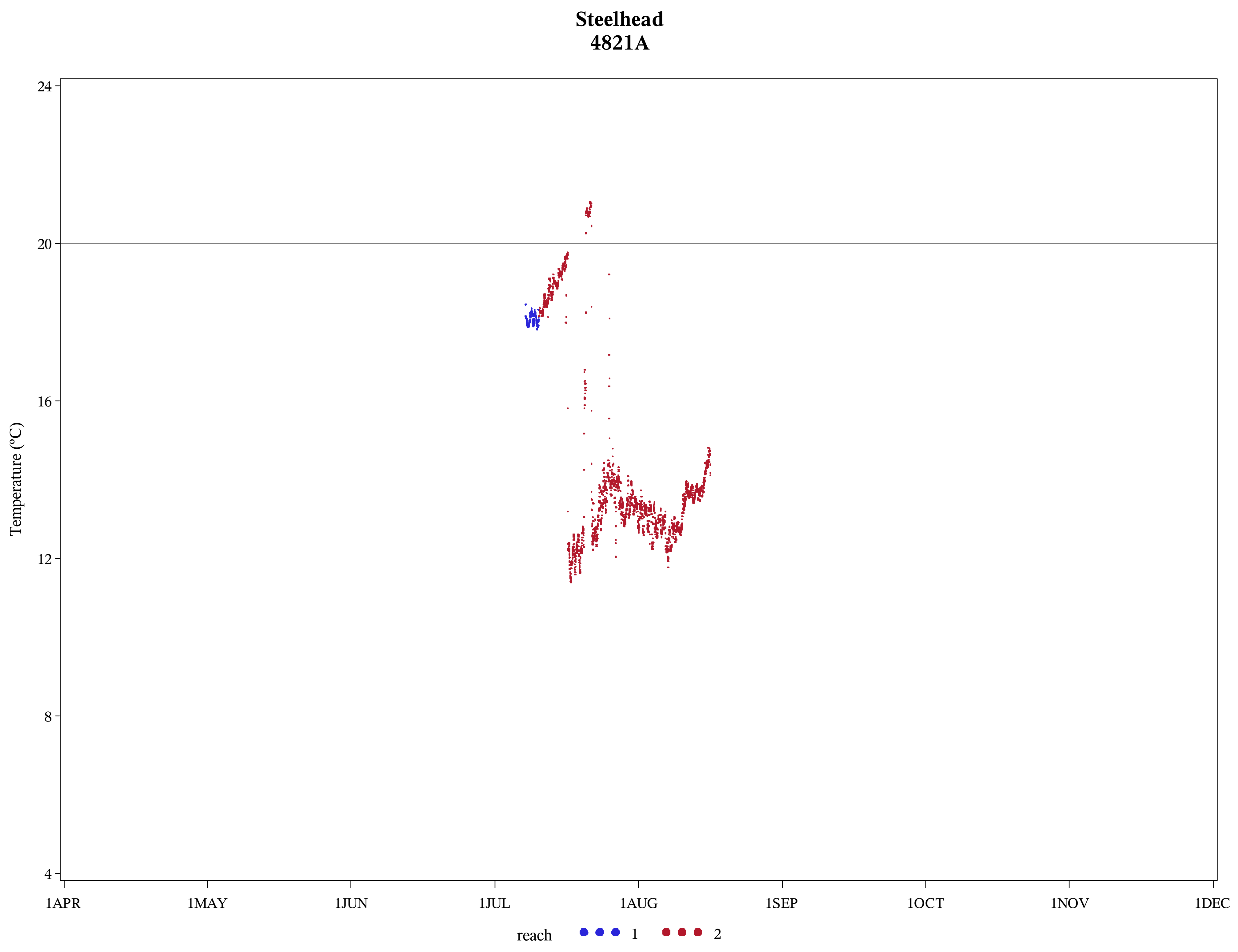

Steelhead
4821A
Temperature (°C)
24
20
16
12
8
4
1APR
1MAY
1JUN
1JUL
1AUG
1SEP
1OCT
1NOV
1DEC
reach
1
2

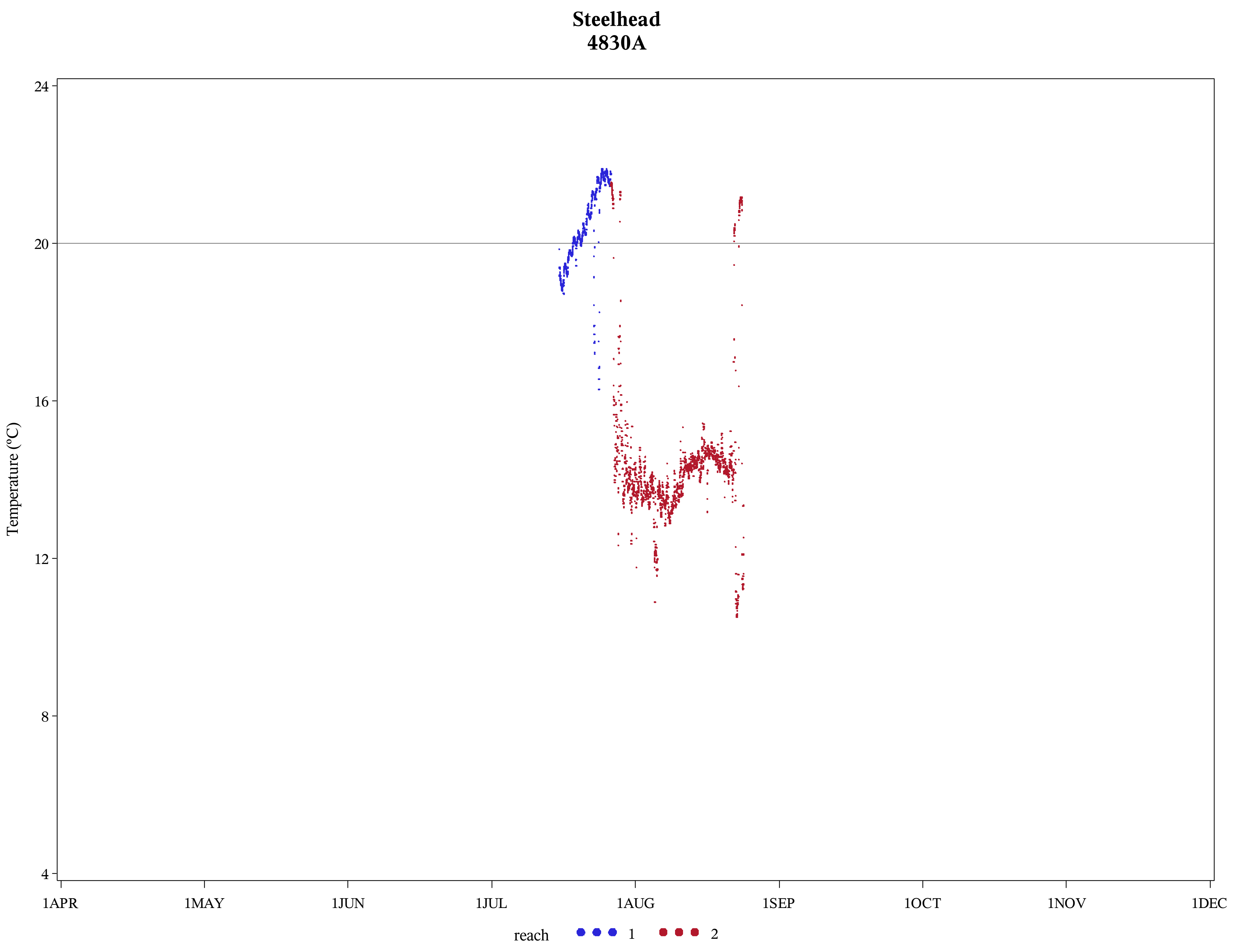

Steelhead
4830A
Temperature (°C)
24
20
16
12
8
4
1APR
1MAY
1JUN
1JUL
1AUG
1SEP
1OCT
1NOV
1DEC
reach
1
2

Steelhead
4833A

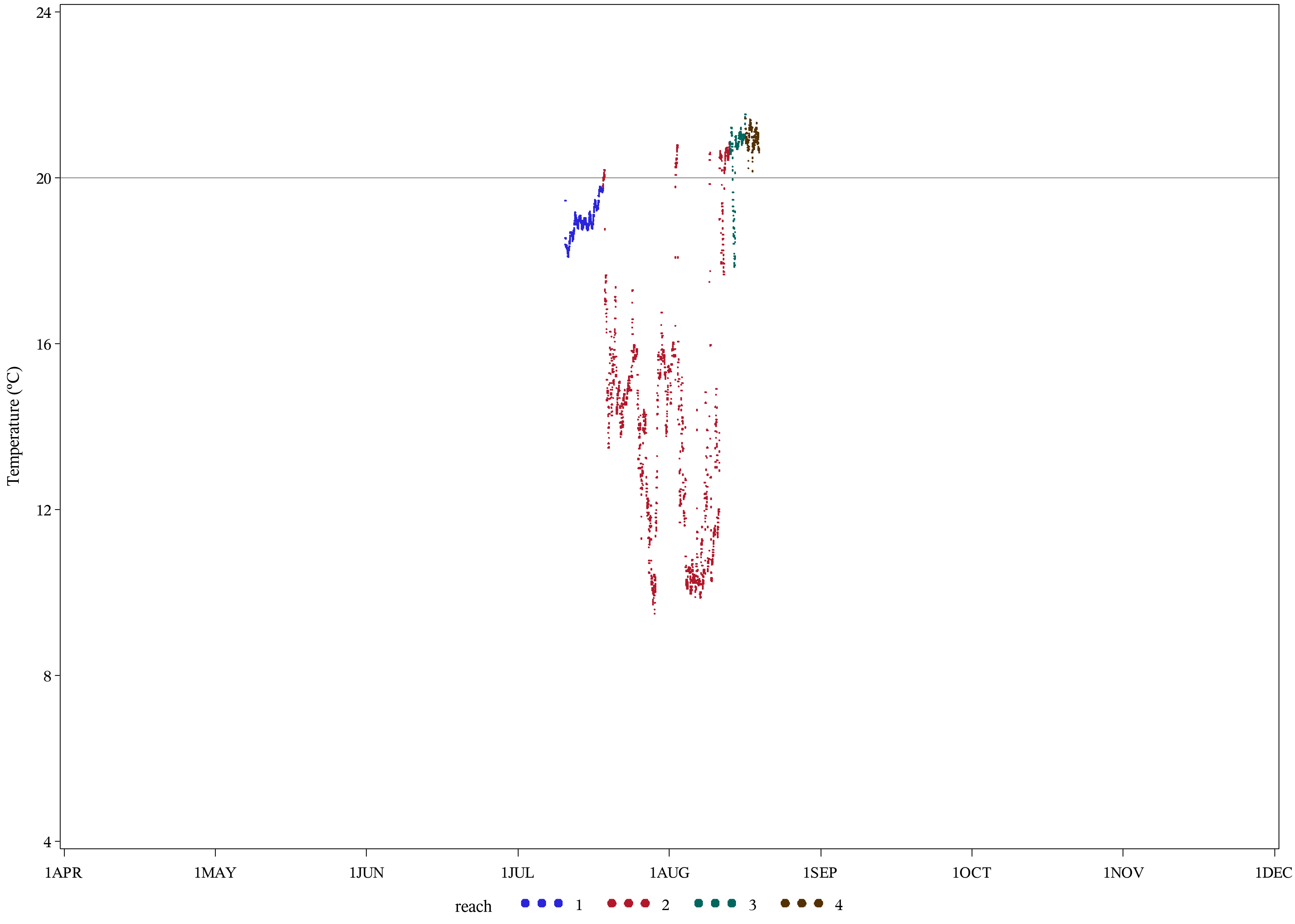

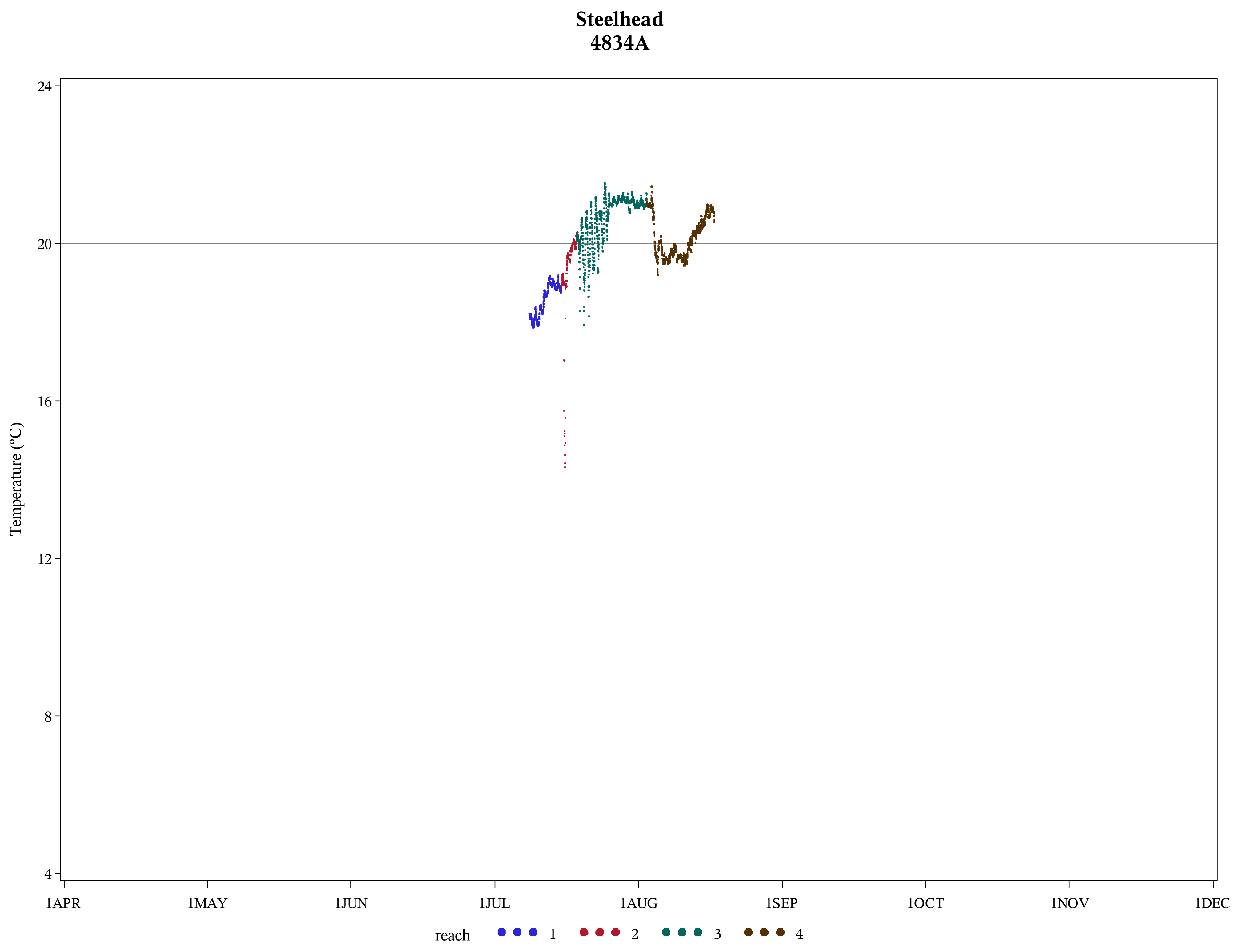

Steelhead
4834A
Temperature (°C)
24
20
16
12
8
4
1APR
1MAY
1JUN
1JUL
1AUG
1SEP
1OCT
1NOV
1DEC
reach
1
2
3
4

Steelhead
4837B

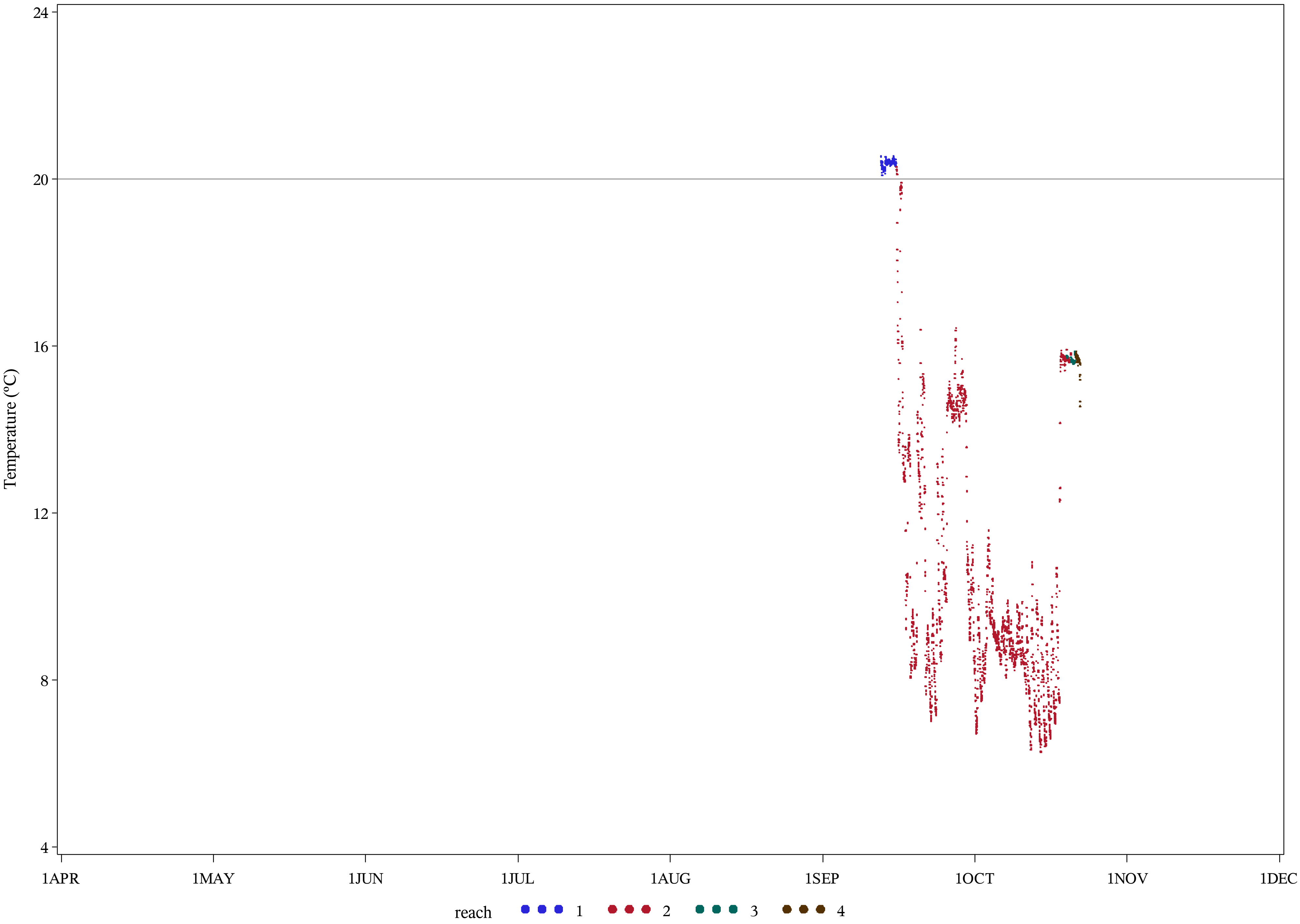

Steelhead
4838A

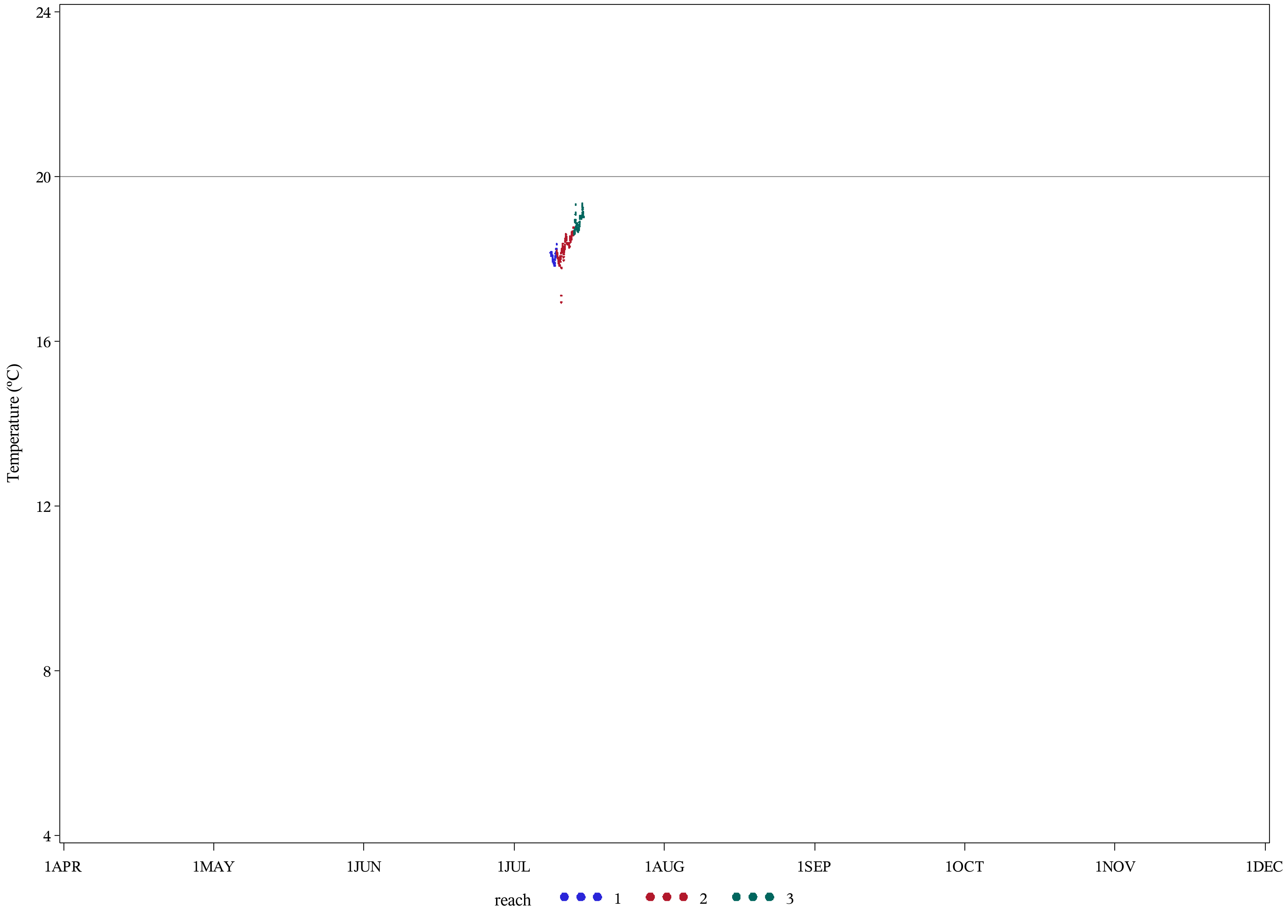

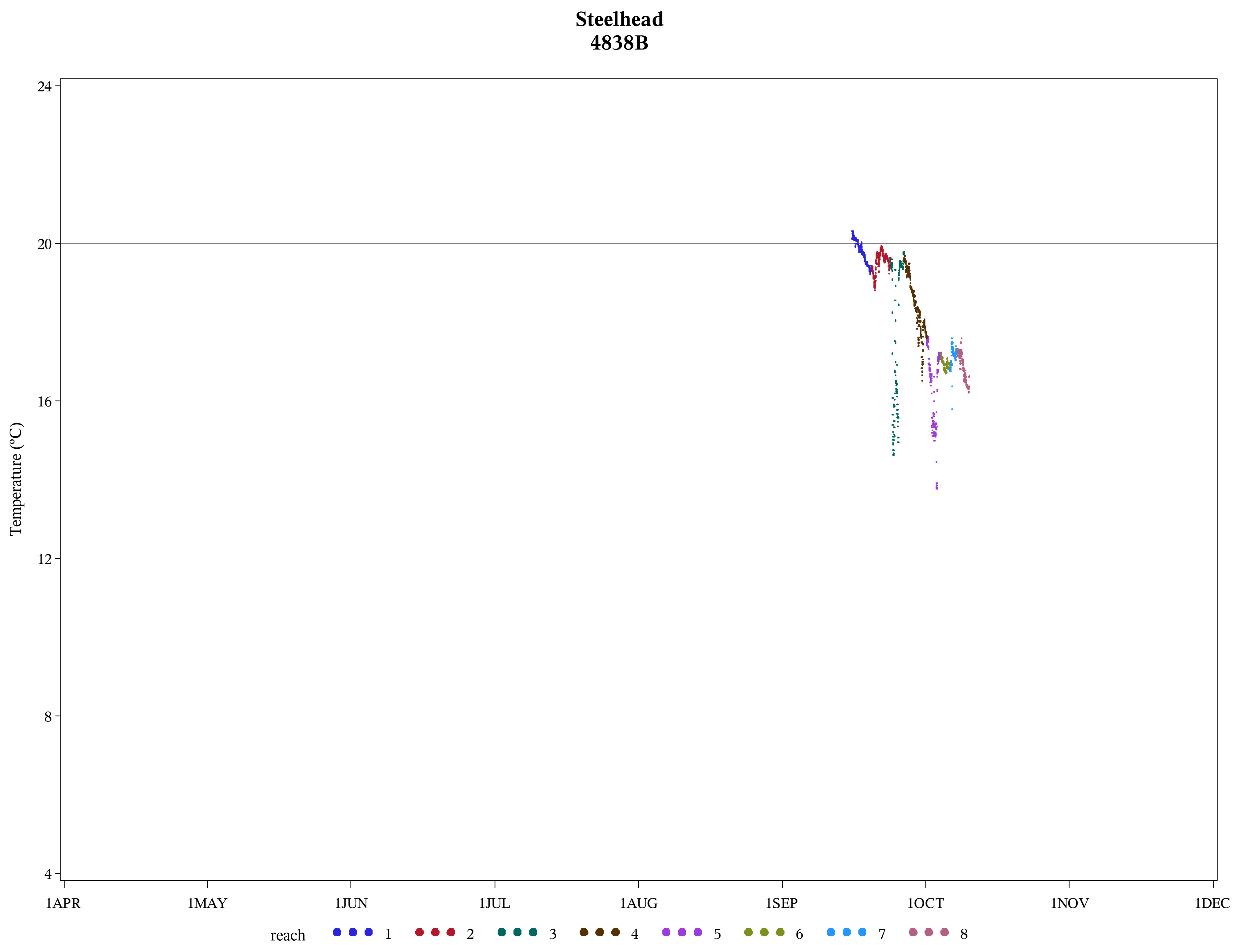
Steelhead
4838B
Temperature (°C)
24
20
16
12
8
4
1APR
1MAY
1JUN
1JUL
1AUG
1SEP
1OCT
1NOV
1DEC
reach
1
2
3
4
5
6
7
8

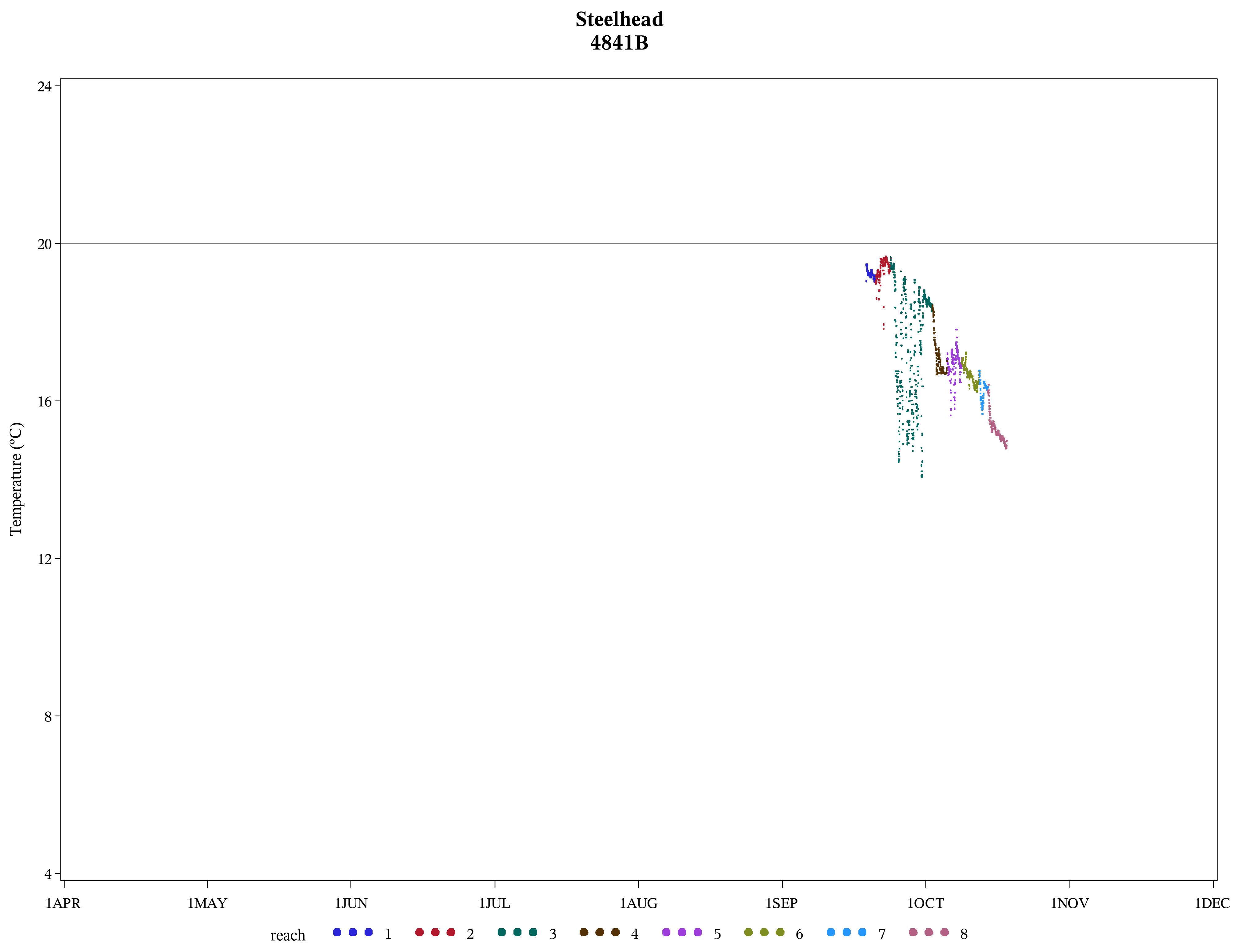
Steelhead
4841B
24
20
16
12
8
4
Temperature (°C)
1APR
1MAY
1JUN
1JUL
1AUG
1SEP
1OCT
1NOV
1DEC
reach
1
2
3
4
5
6
7
8

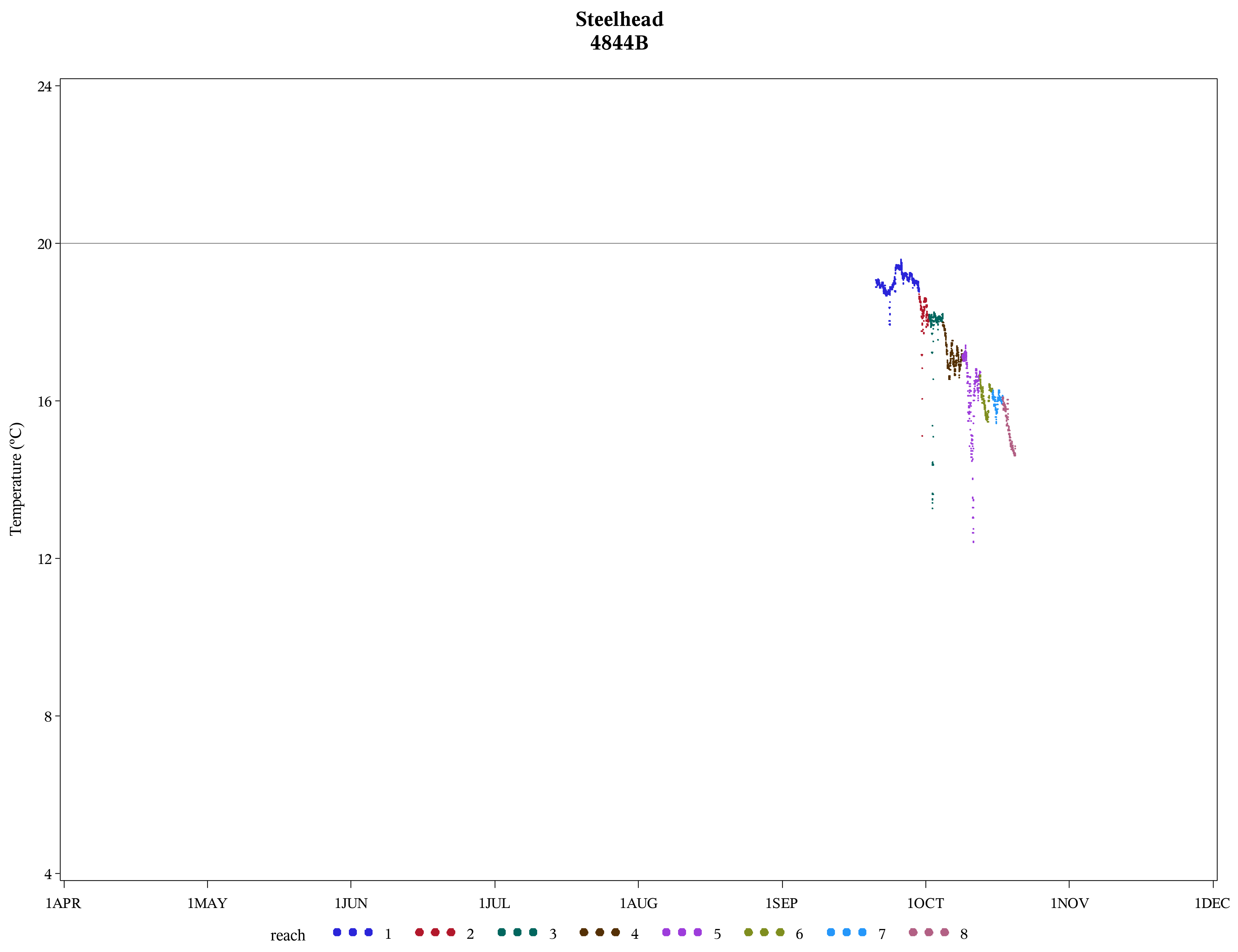

Steelhead
4844B
Temperature (°C)
24
20
16
12
8
4
1APR
1MAY
1JUN
1JUL
1AUG
1SEP
1OCT
1NOV
1DEC
reach
1
2
3
4
5
6
7
8

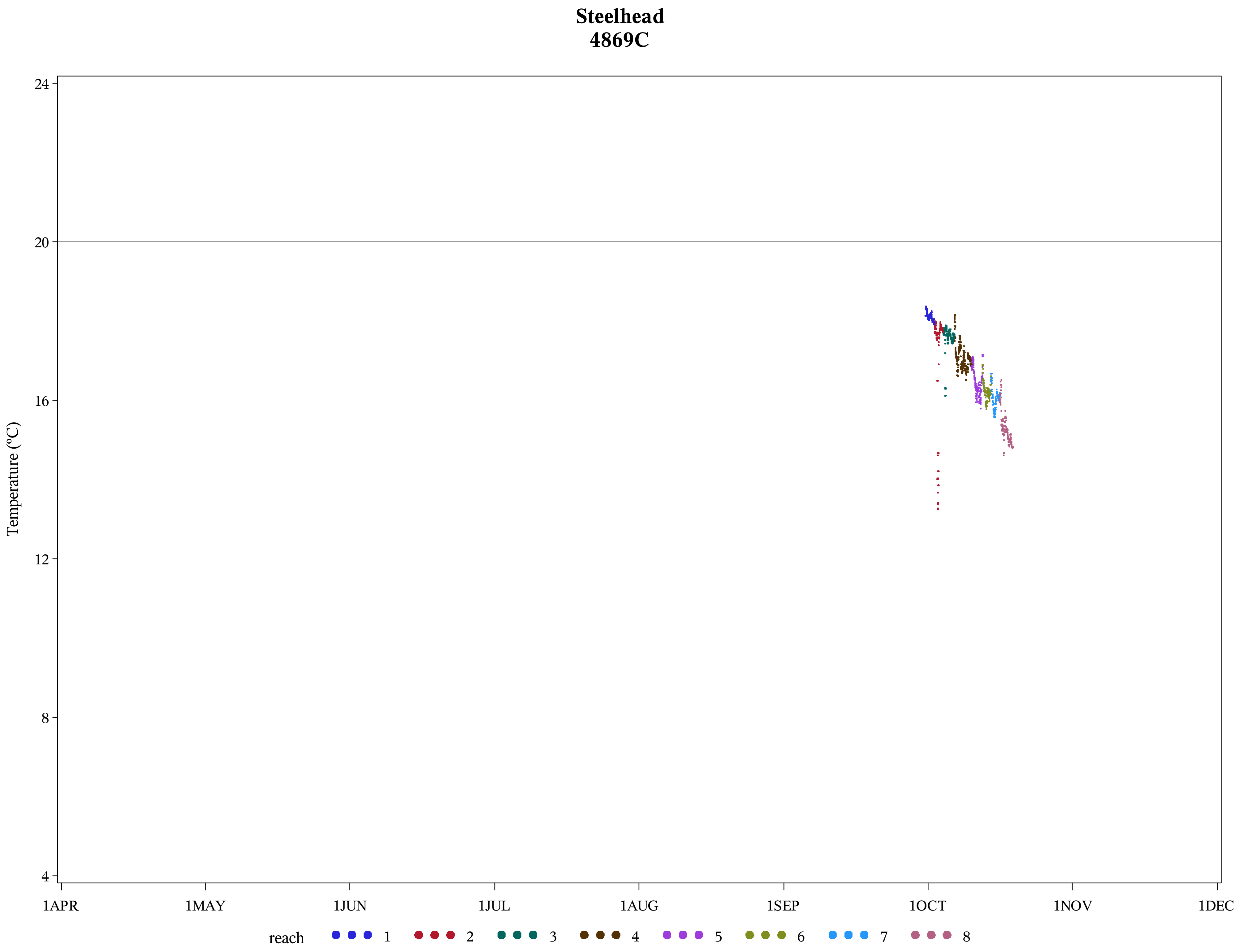

Steelhead
4869C
Temperature (°C)
24
20
16
12
8
4
1APR
1MAY
1JUN
1JUL
1AUG
1SEP
1OCT
1NOV
1DEC
reach
1
2
3
4
5
6
7
8

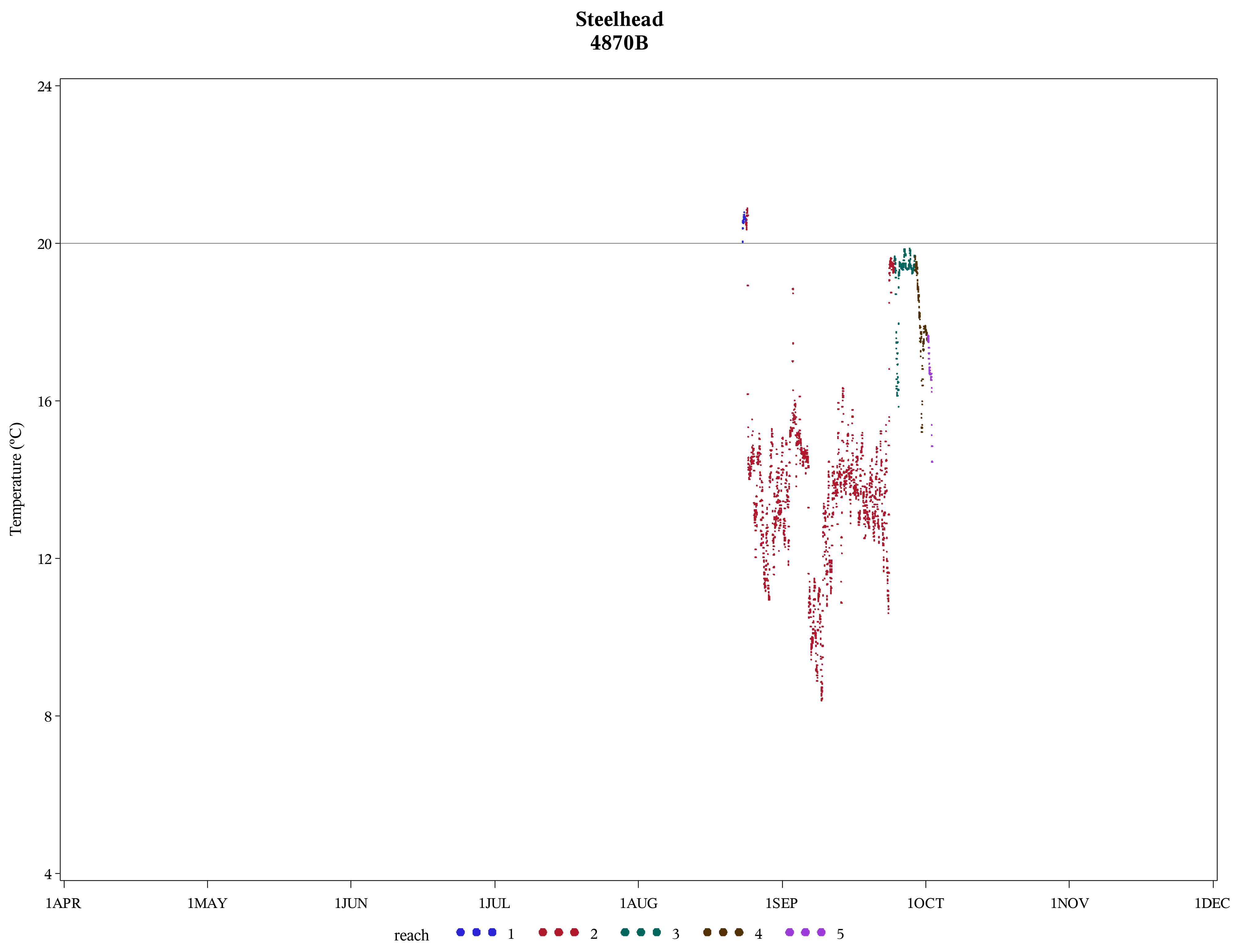

Steelhead
4870B
Temperature (°C)
24
20
16
12
8
4
1APR
1MAY
1JUN
1JUL
1AUG
1SEP
1OCT
1NOV
1DEC
reach
1
2
3
4
5

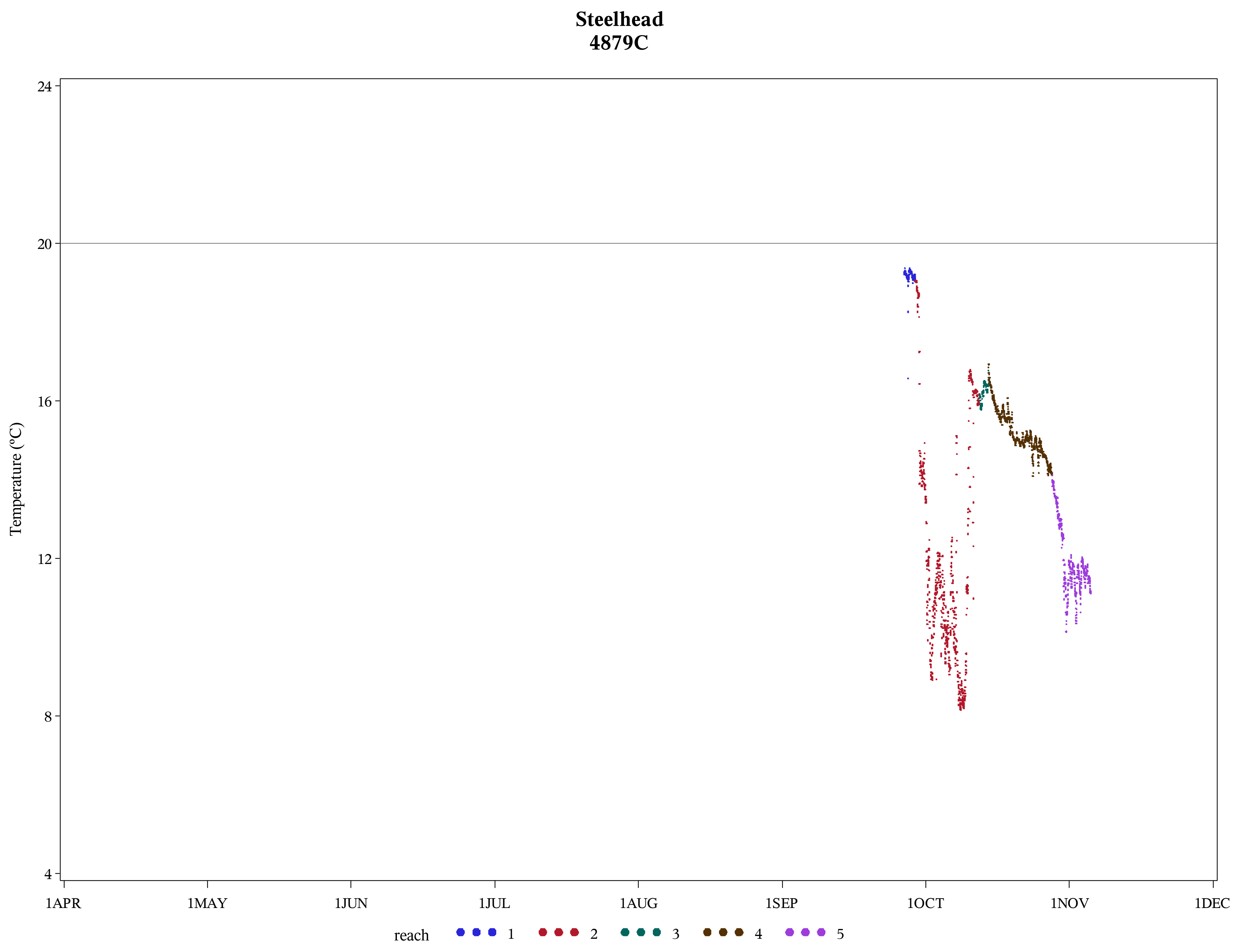

Steelhead
4879C
Temperature (°C)
24
20
16
12
8
4
1APR
1MAY
1JUN
1JUL
1AUG
1SEP
1OCT
1NOV
1DEC
reach
1
2
3
4
5

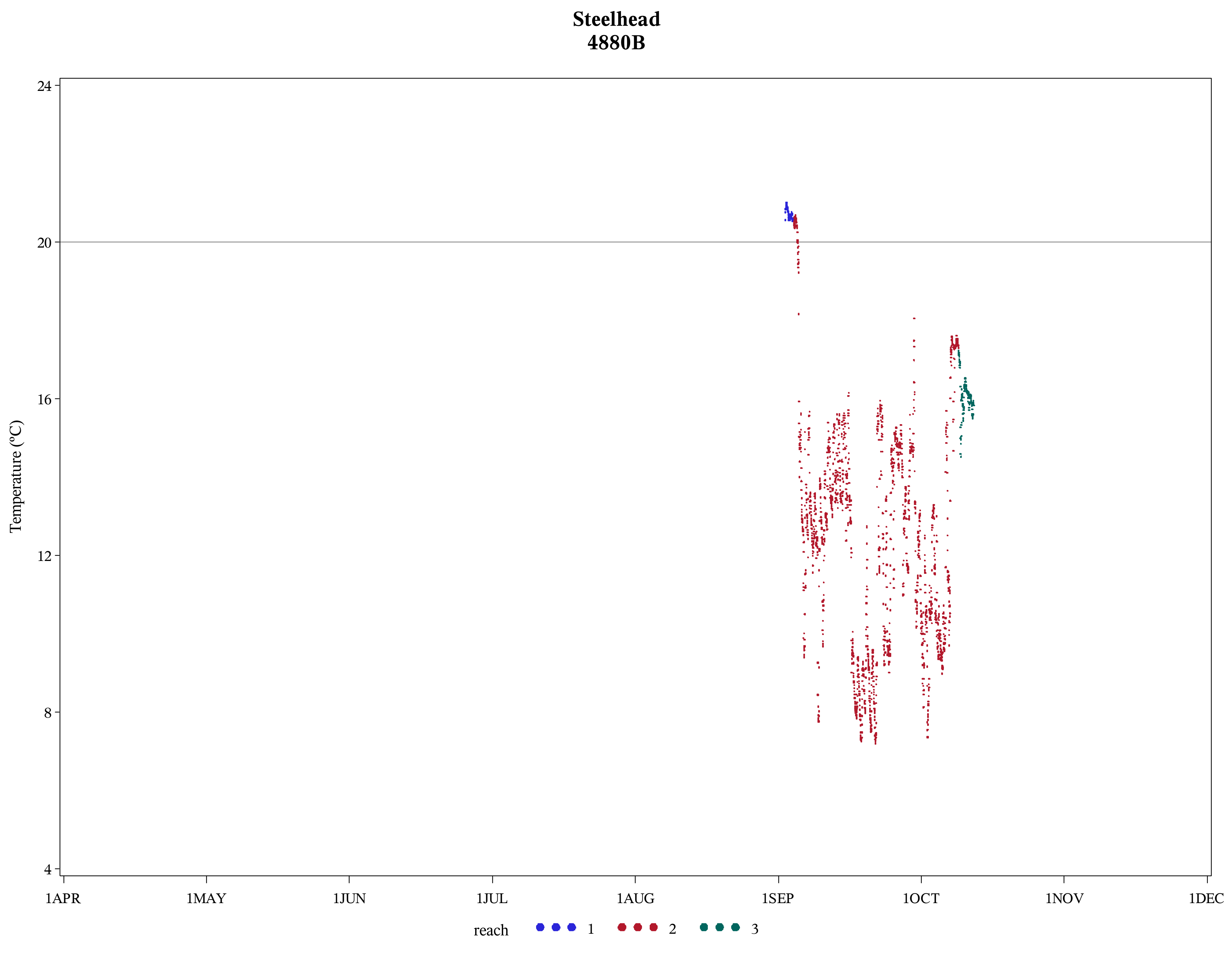
Steelhead
4880B
Temperature (°C)
24
20
16
12
8
4
1APR
1MAY
1JUN
1JUL
1AUG
1SEP
1OCT
1NOV
1DEC
reach
1
2
3

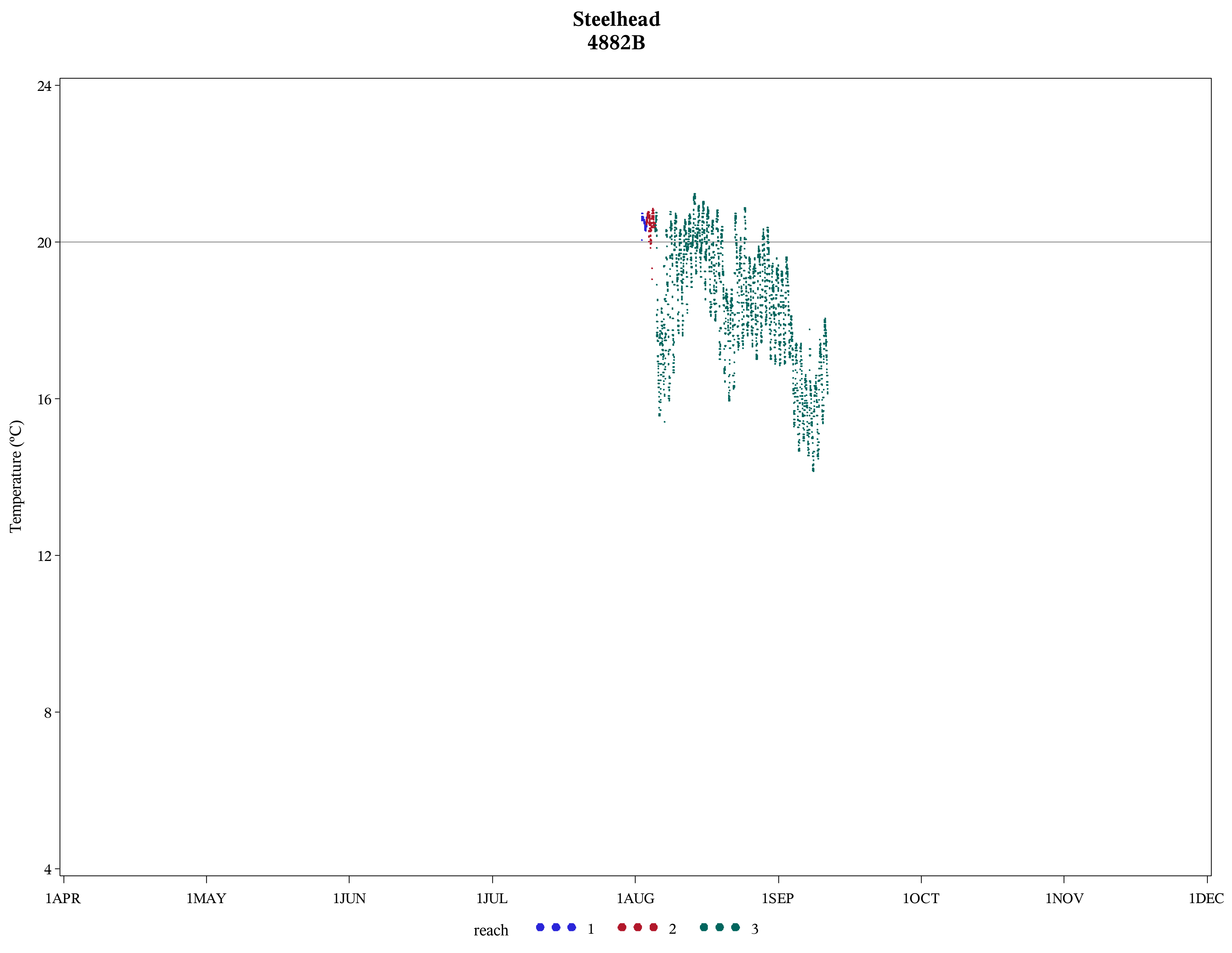

Steelhead
4882B
Temperature (°C)
24
20
16
12
8
4
1APR
1MAY
1JUN
1JUL
1AUG
1SEP
1OCT
1NOV
1DEC
reach
1
2
3

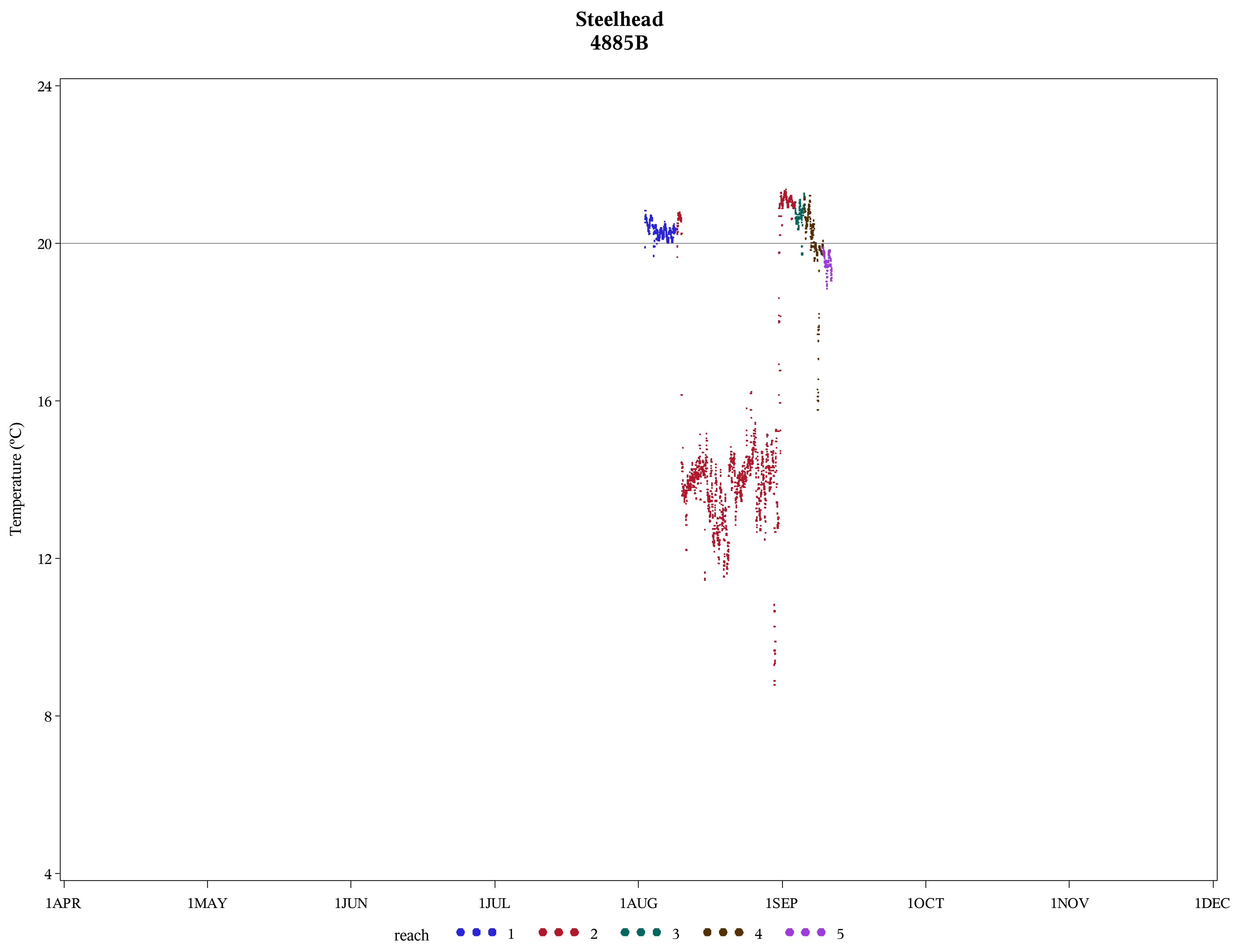
Steelhead
4885B
Temperature (°C)
24
20
16
12
8
4
1APR
1MAY
1JUN
1JUL
1AUG
1SEP
1OCT
1NOV
1DEC
reach
1
2
3
4
5

Steelhead
4886B

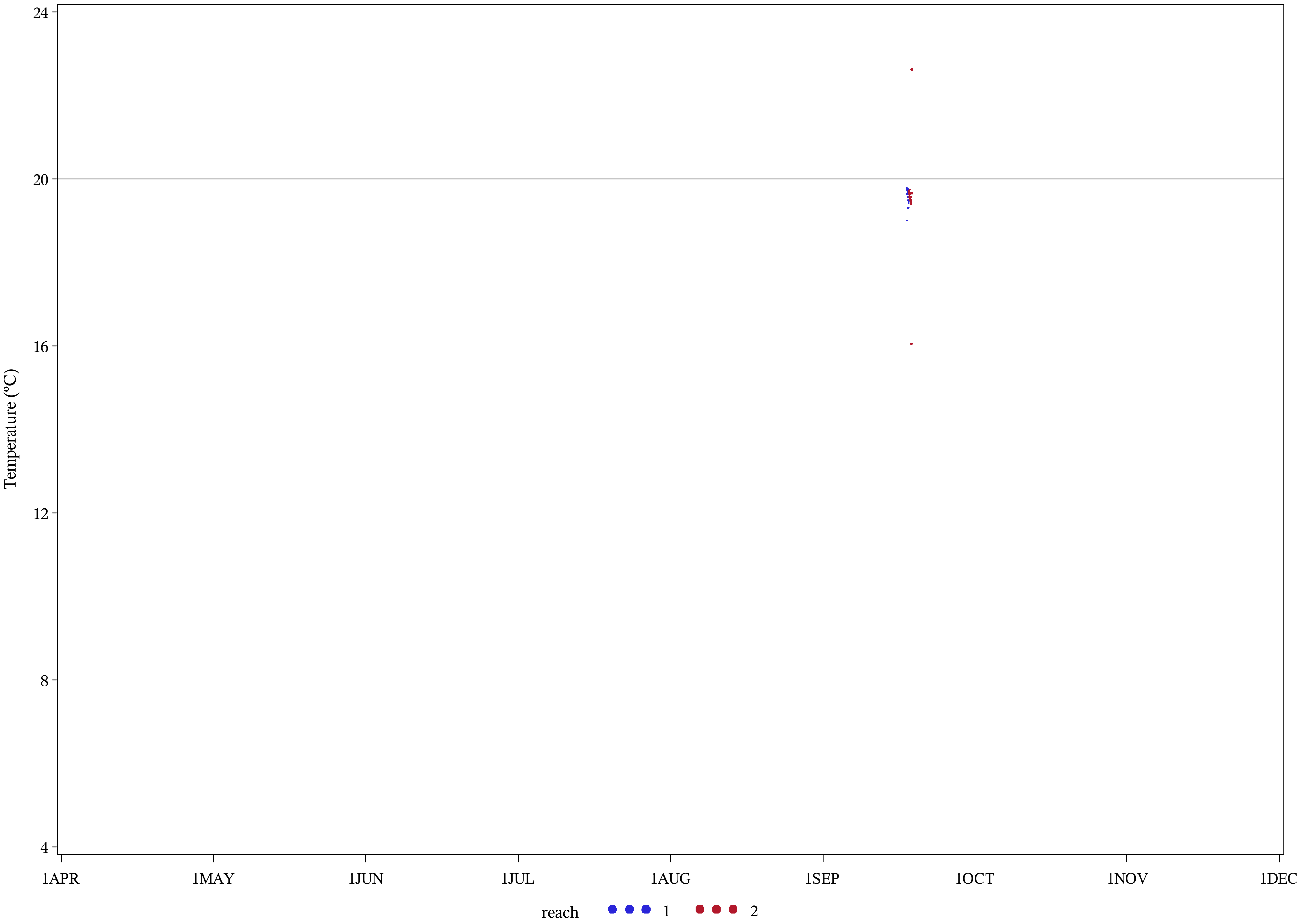

Steelhead
4886C

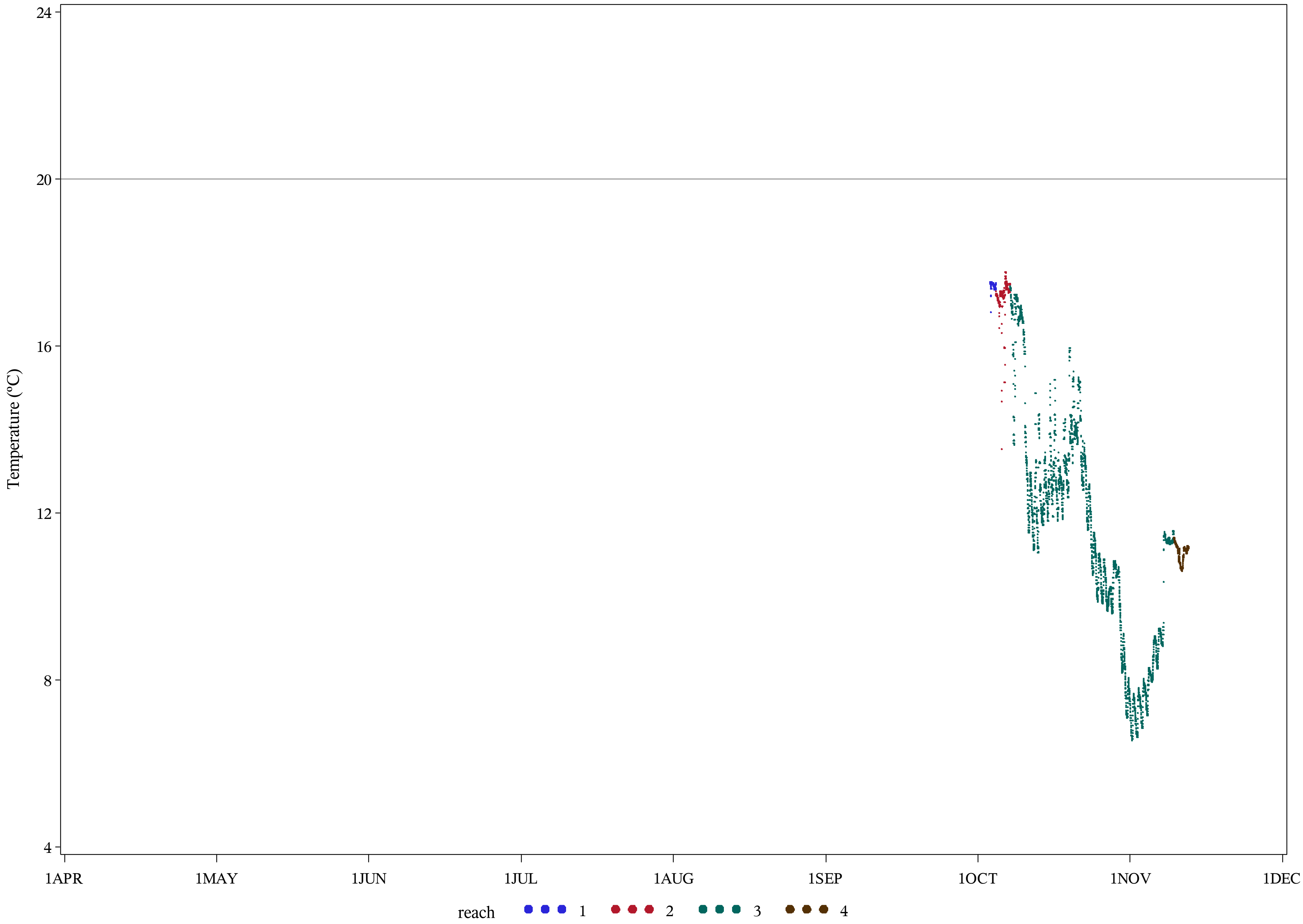

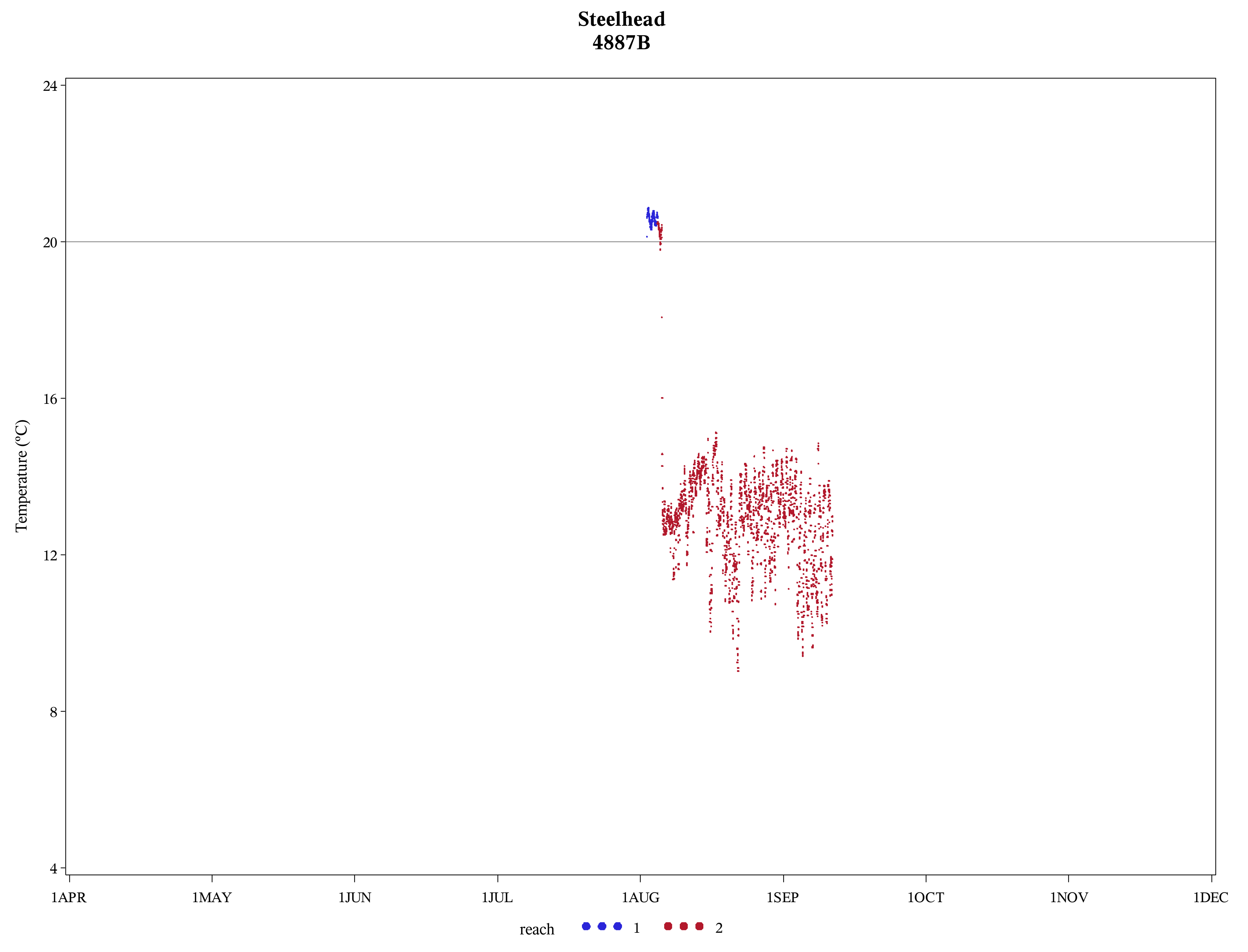

Steelhead
4887B
Temperature (°C)
24
20
16
12
8
4
1APR
1MAY
1JUN
1JUL
1AUG
1SEP
1OCT
1NOV
1DEC
reach
1
2

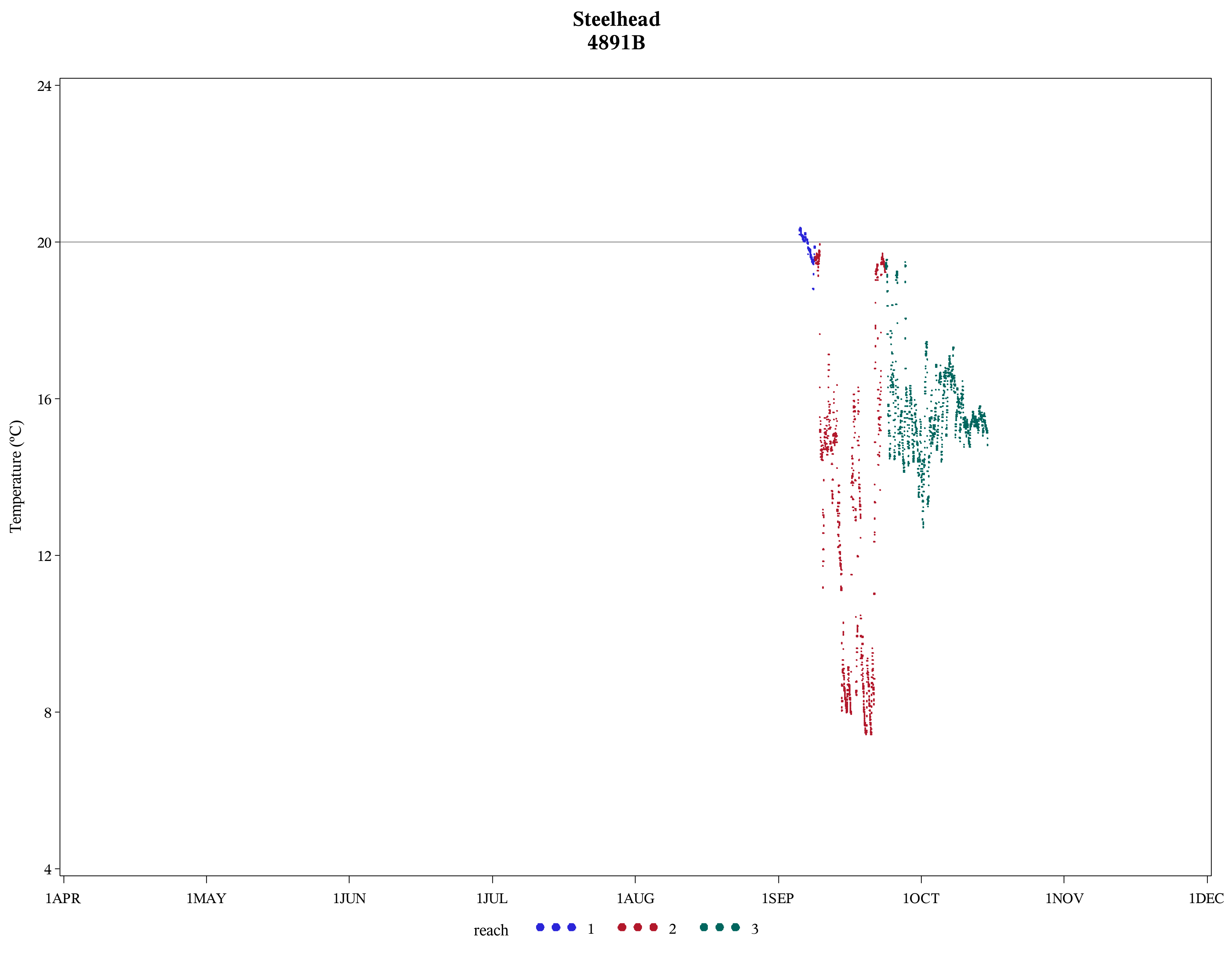
Steelhead
4891B
Temperature (°C)
24
20
16
12
8
4
1APR
1MAY
1JUN
1JUL
1AUG
1SEP
1OCT
1NOV
1DEC
reach
1
2
3

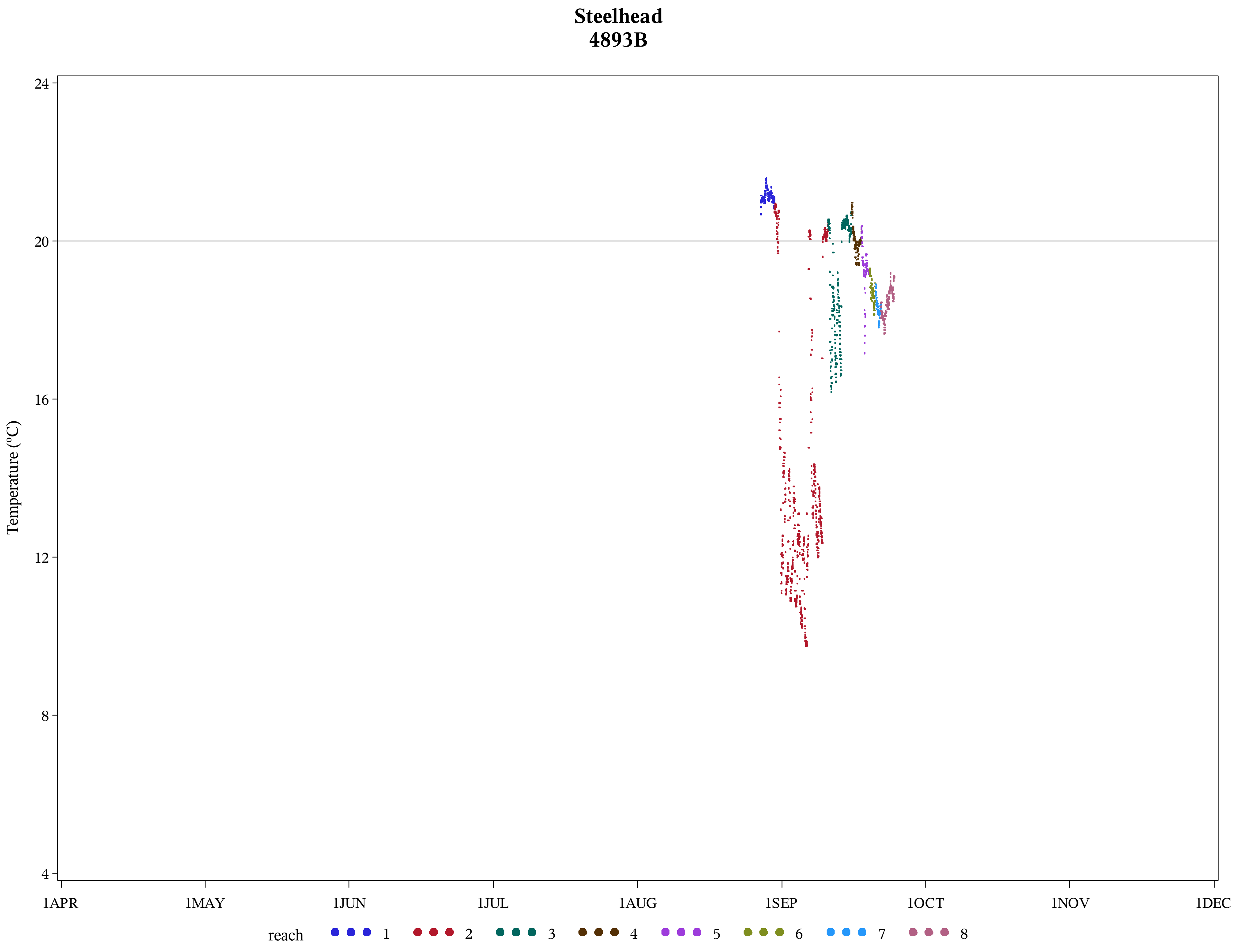
Steelhead
4893B
Temperature (°C)
24
20
16
12
8
4
1APR
1MAY
1JUN
1JUL
1AUG
1SEP
1OCT
1NOV
1DEC
reach
1
2
3
4
5
6
7
8

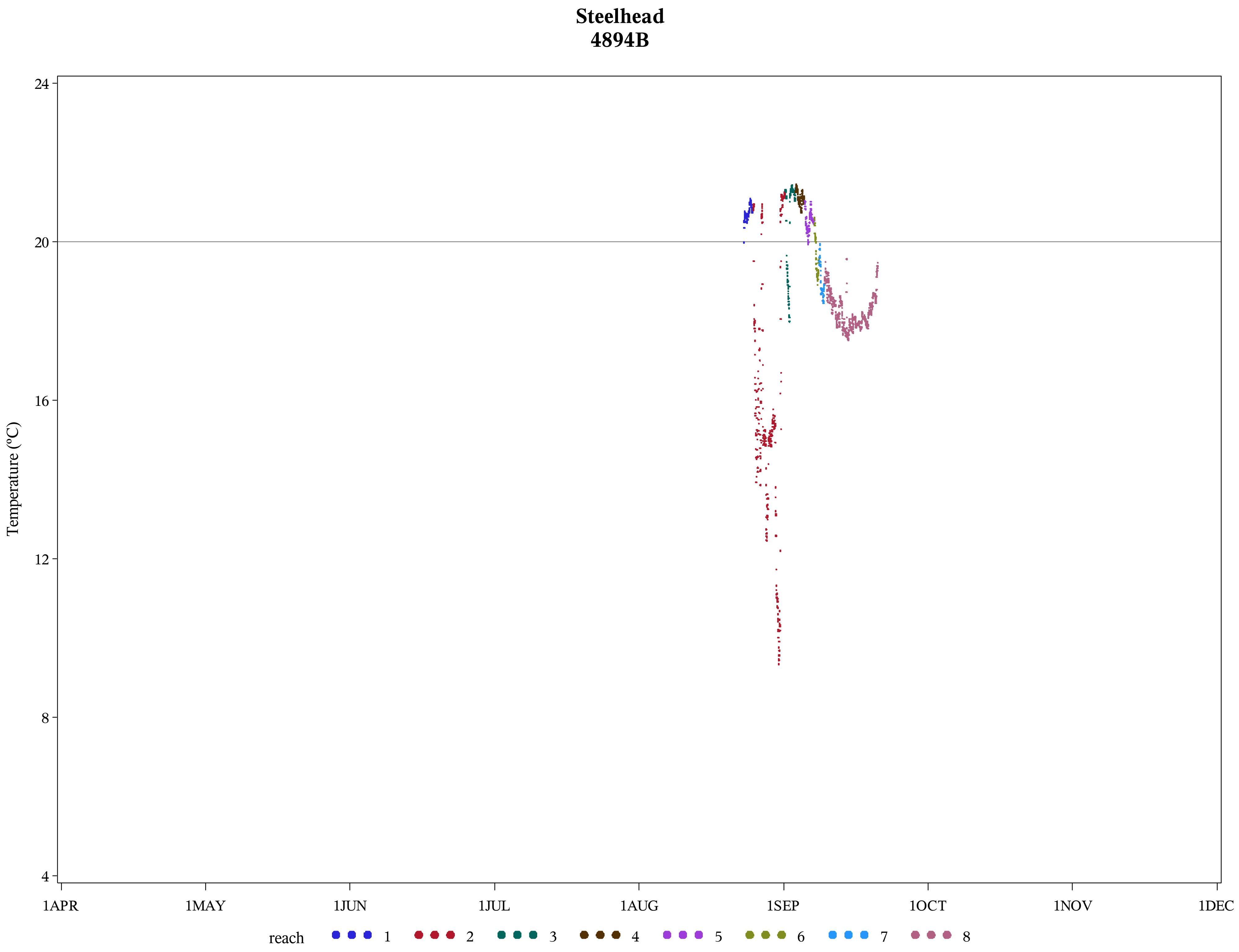

Steelhead
4894B
Temperature (°C)
24
20
16
12
8
4
1APR
1MAY
1JUN
1JUL
1AUG
1SEP
1OCT
1NOV
1DEC
reach
1
2
3
4
5
6
7
8

Steelhead
4897B

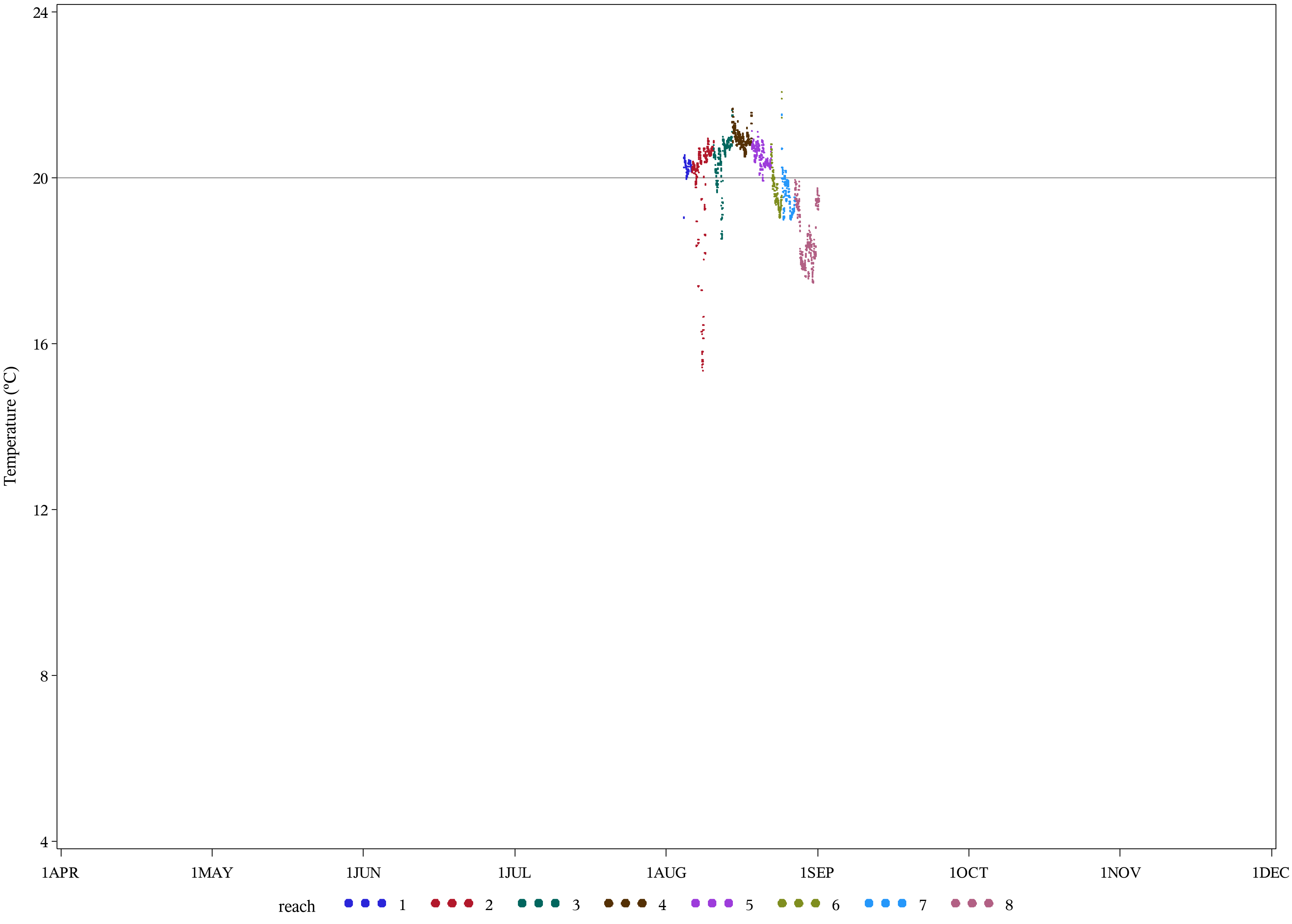

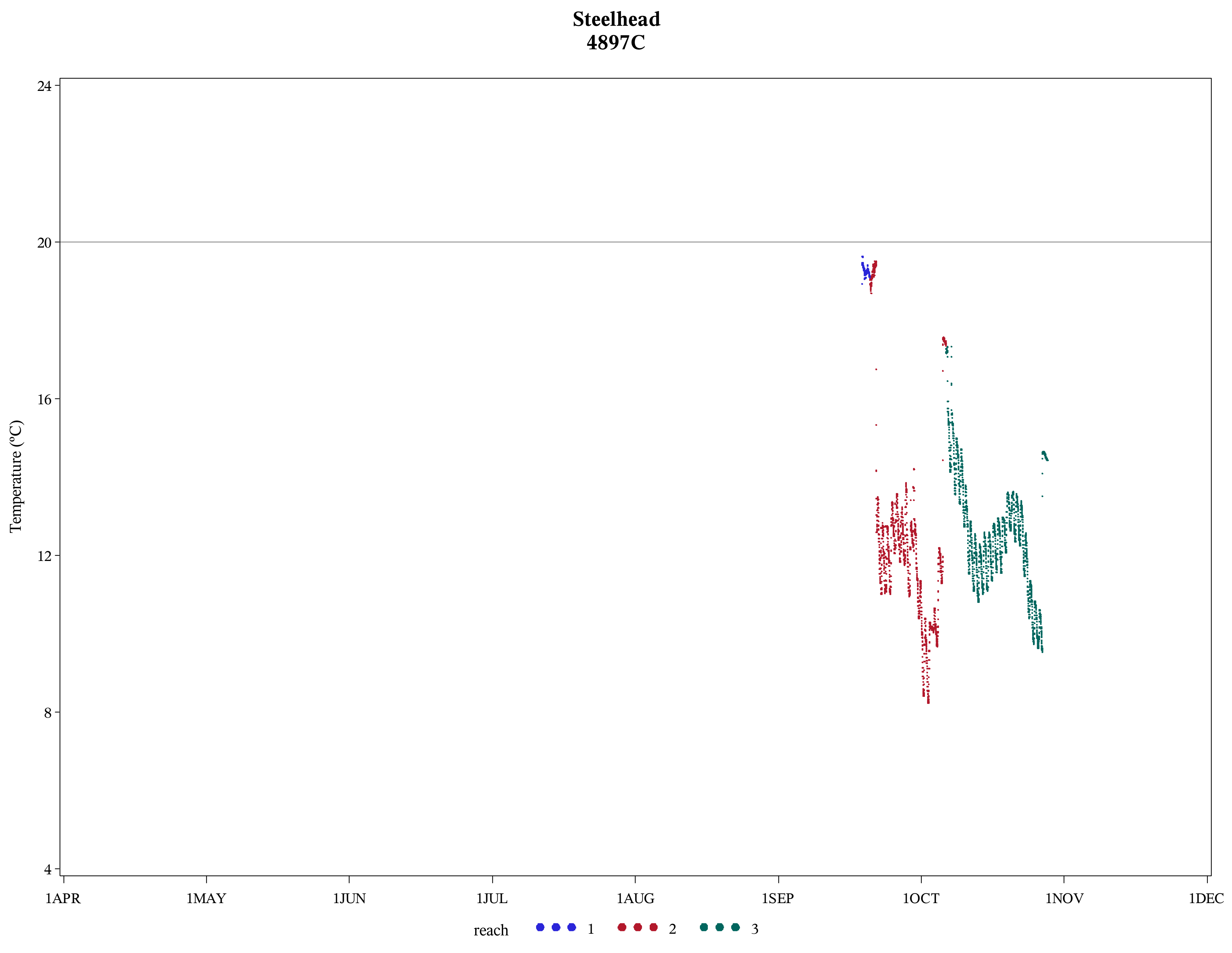

Steelhead
4897C
Temperature (°C)
24
20
16
12
8
4
1APR
1MAY
1JUN
1JUL
1AUG
1SEP
1OCT
1NOV
1DEC
reach
1
2
3

Steelhead
4906B

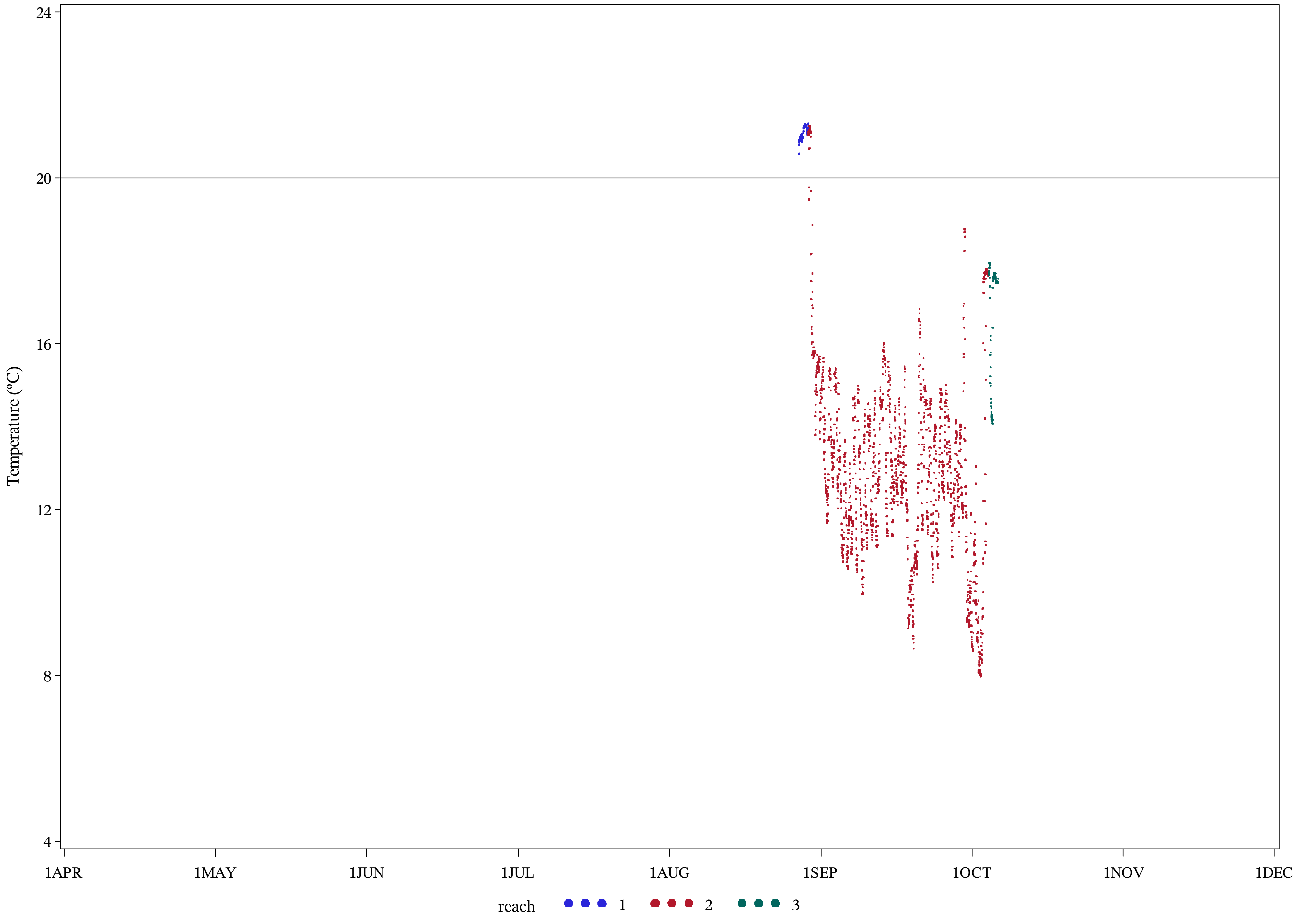

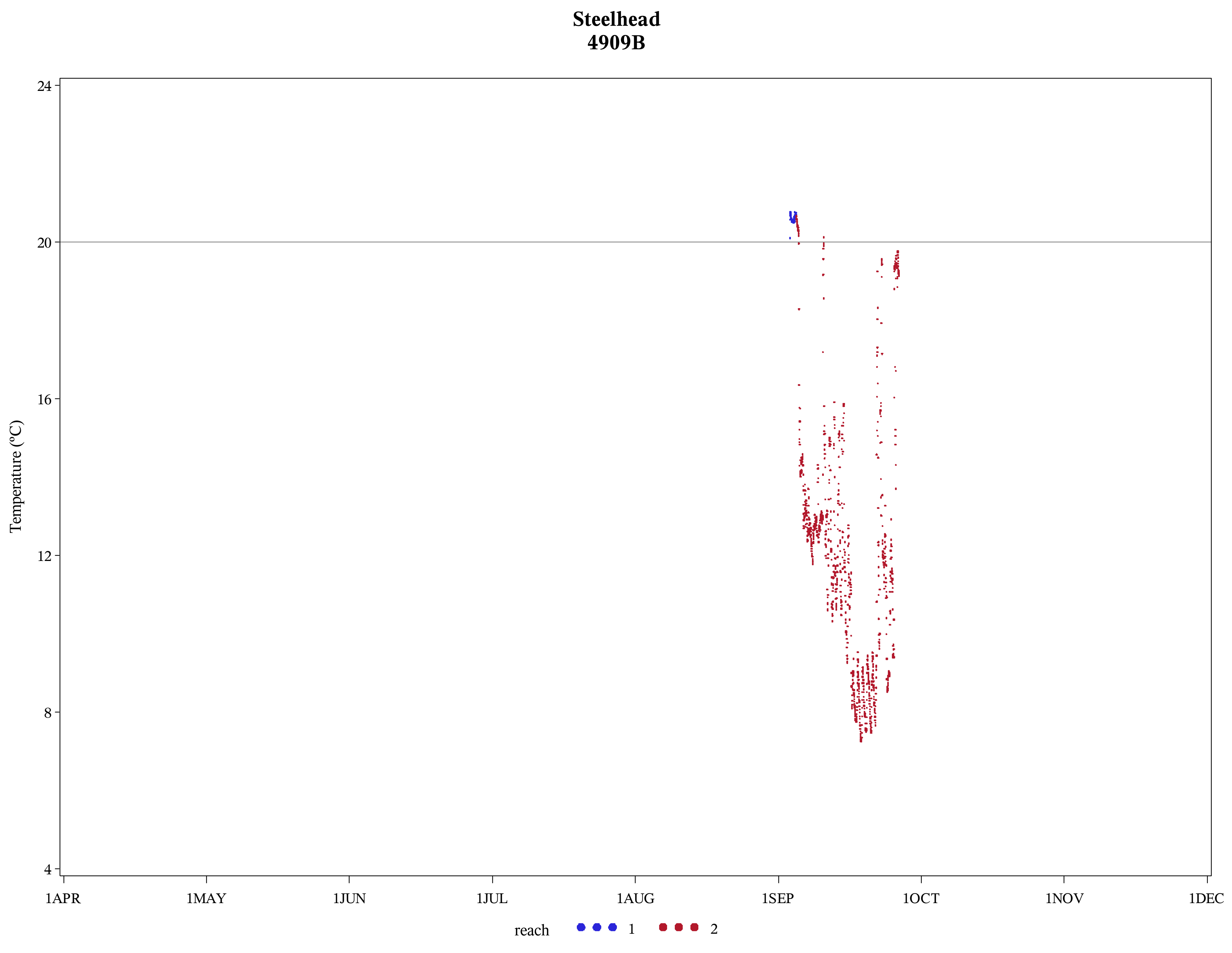
Steelhead
4909B
Temperature (°C)
24
20
16
12
8
4
1APR
1MAY
1JUN
1JUL
1AUG
1SEP
1OCT
1NOV
1DEC
reach
1
2

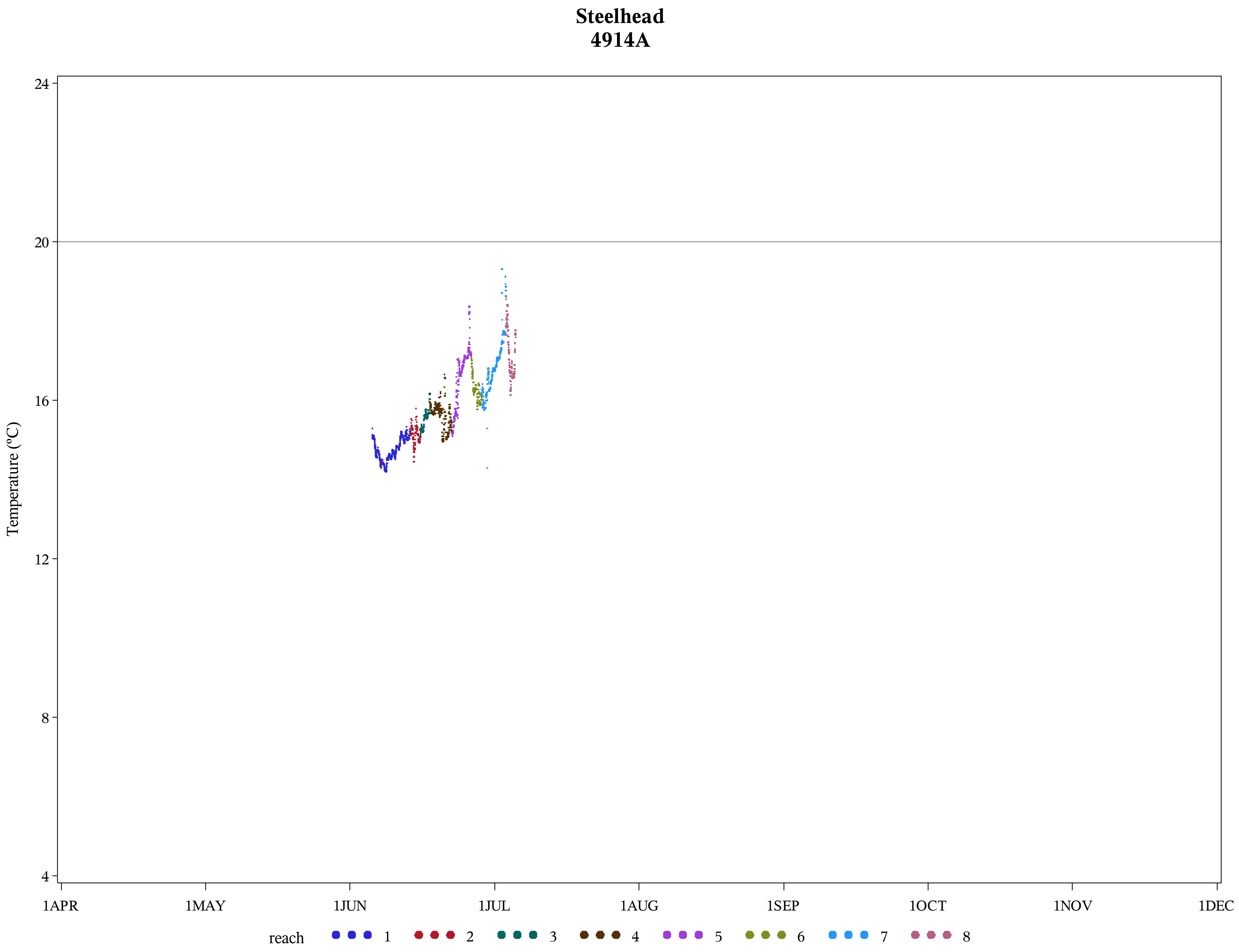

Steelhead
4914A
Temperature (°C)
24
20
16
12
8
4
1APR
1MAY
1JUN
1JUL
1AUG
1SEP
1OCT
1NOV
1DEC
reach
1
2
3
4
5
6
7
8

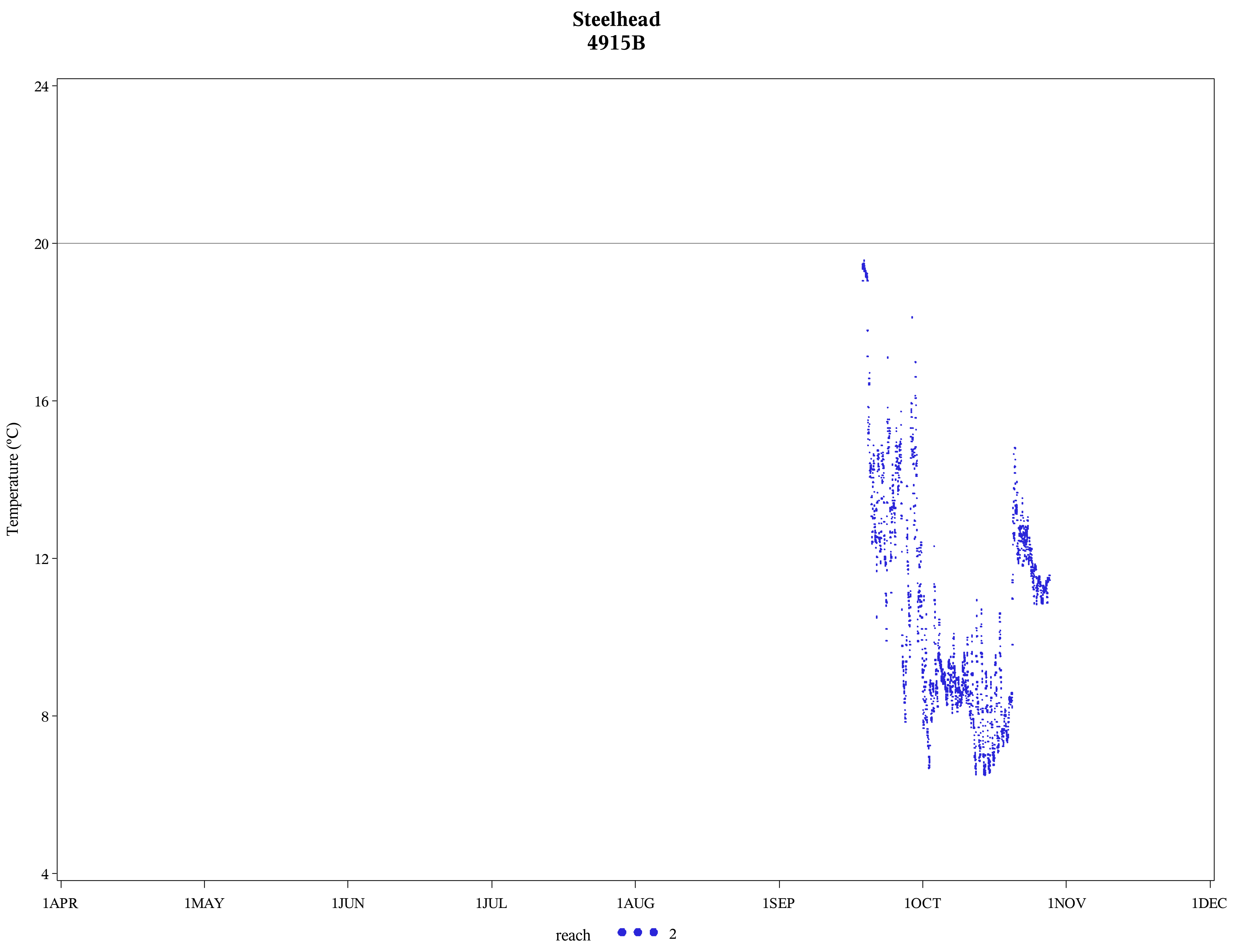
Steelhead
4915B
Temperature (°C)
24
20
16
12
8
4
1APR
1MAY
1JUN
1JUL
1AUG
1SEP
1OCT
1NOV
1DEC
reach
2

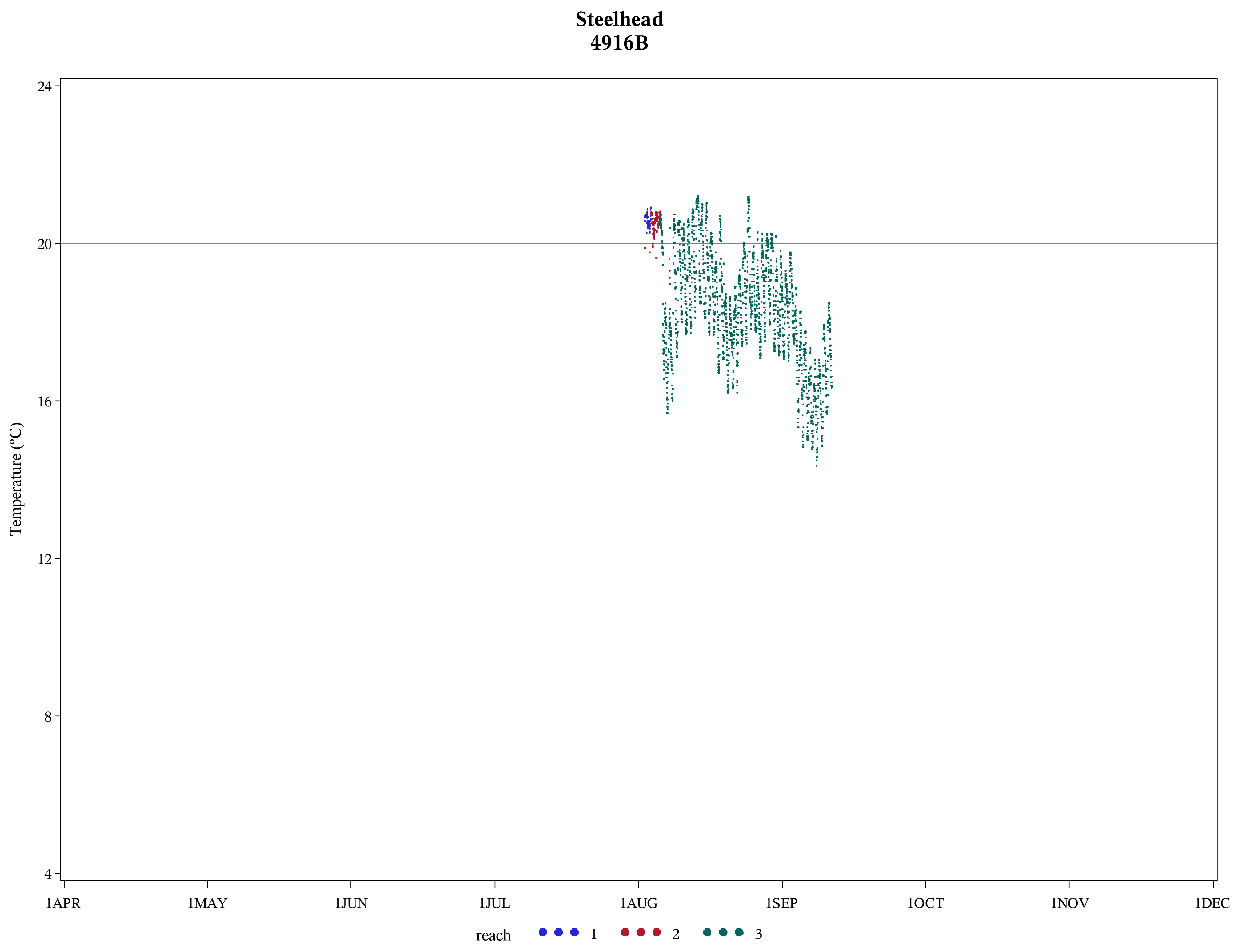

Steelhead
4916B
Temperature (°C)
24
20
16
12
8
4
1APR
1MAY
1JUN
1JUL
1AUG
1SEP
1OCT
1NOV
1DEC
reach
1
2
3

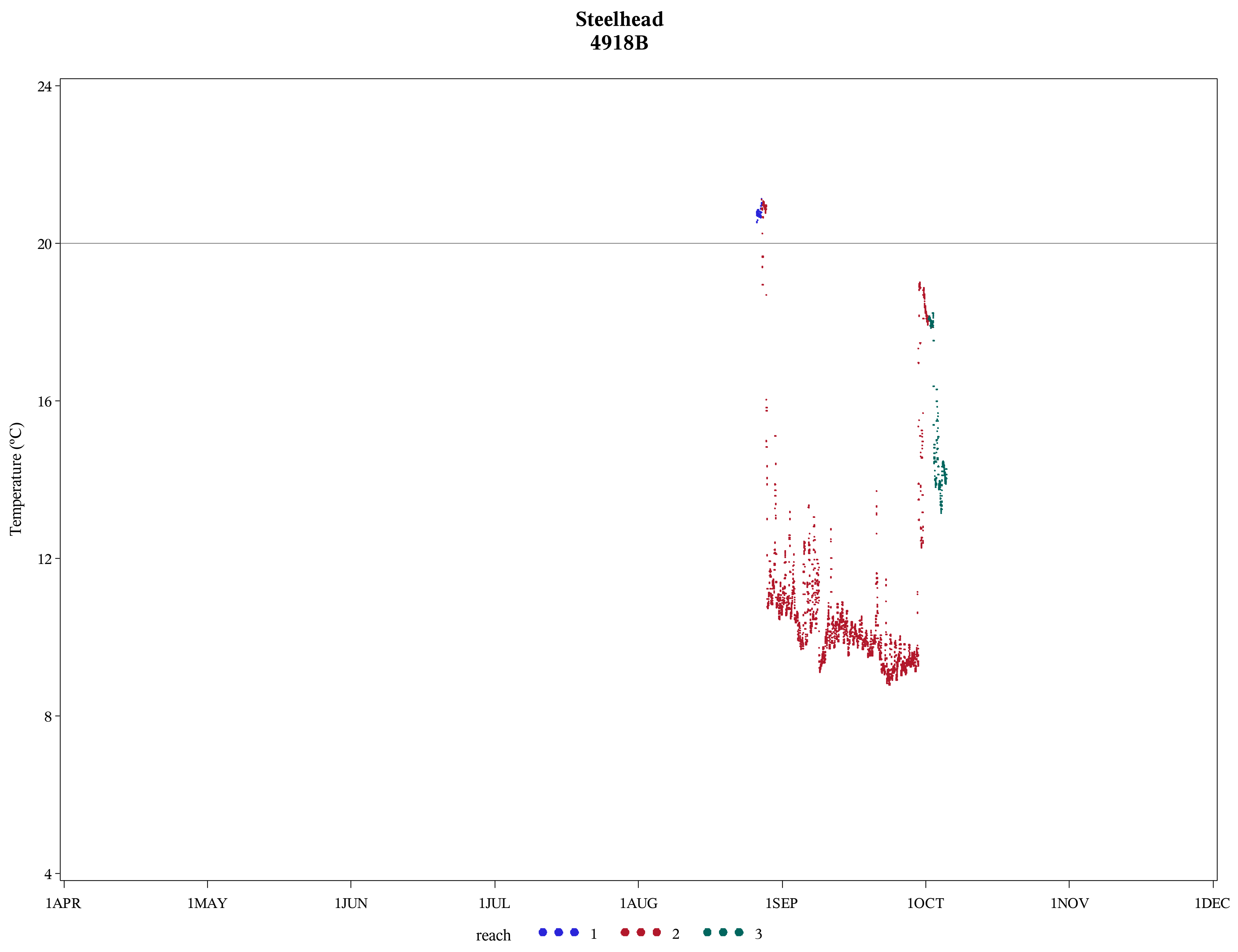

Steelhead
4918B
Temperature (°C)
24
20
16
12
8
4
1APR
1MAY
1JUN
1JUL
1AUG
1SEP
1OCT
1NOV
1DEC
reach
1
2
3

Steelhead
4922B

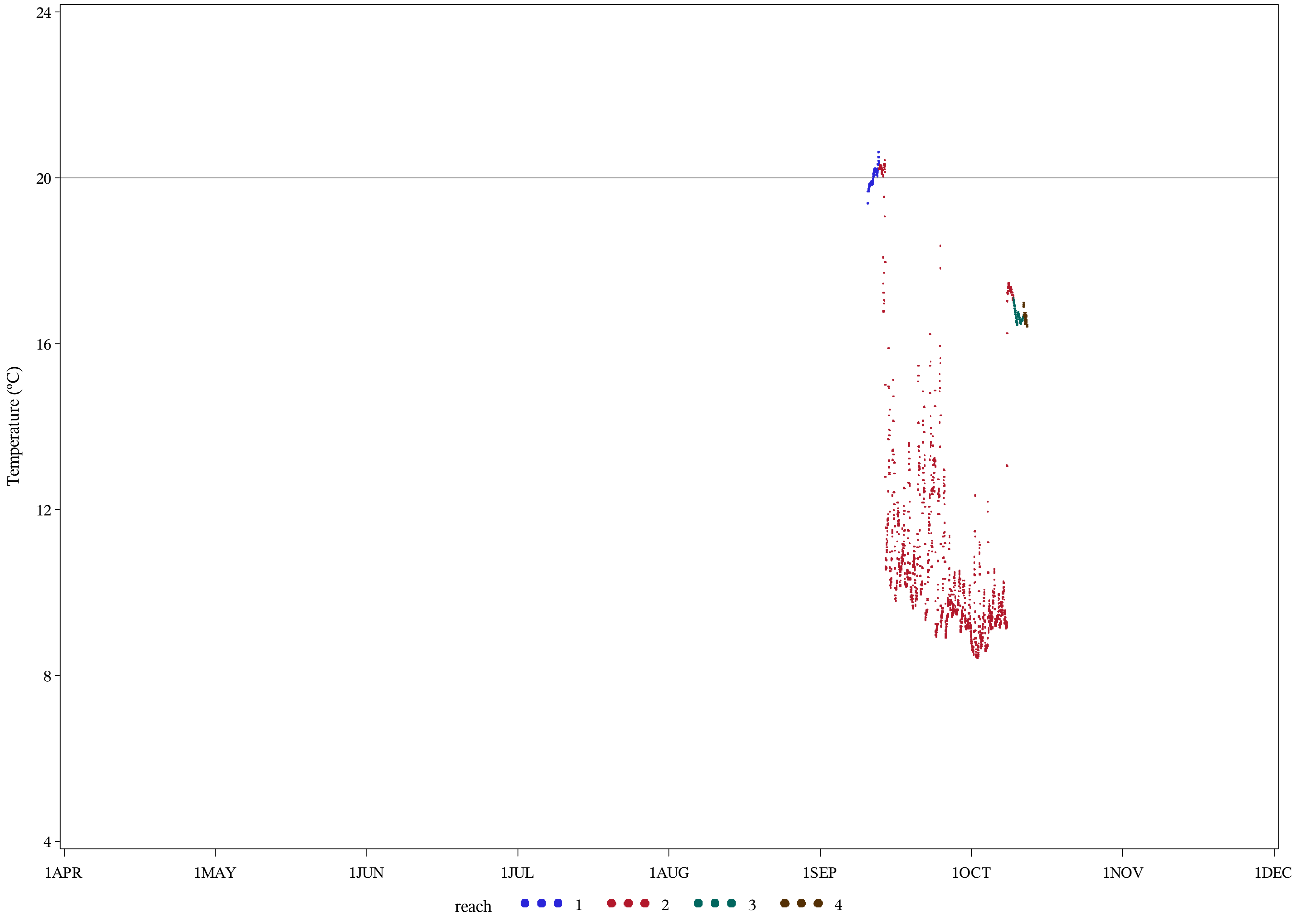

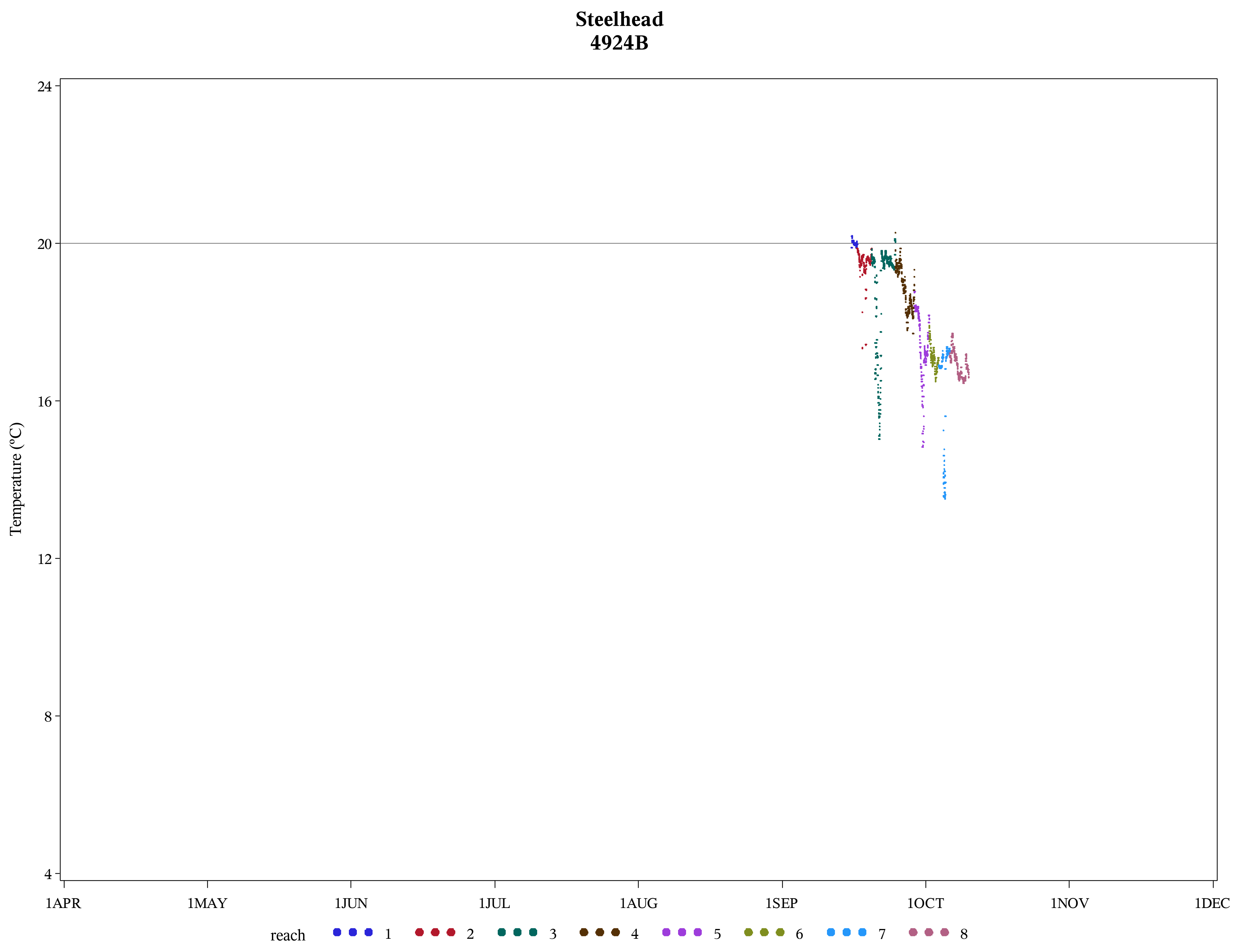
Steelhead
4924B
Temperature (°C)
24
20
16
12
8
4
1APR
1MAY
1JUN
1JUL
1AUG
1SEP
1OCT
1NOV
1DEC
reach
1
2
3
4
5
6
7
8

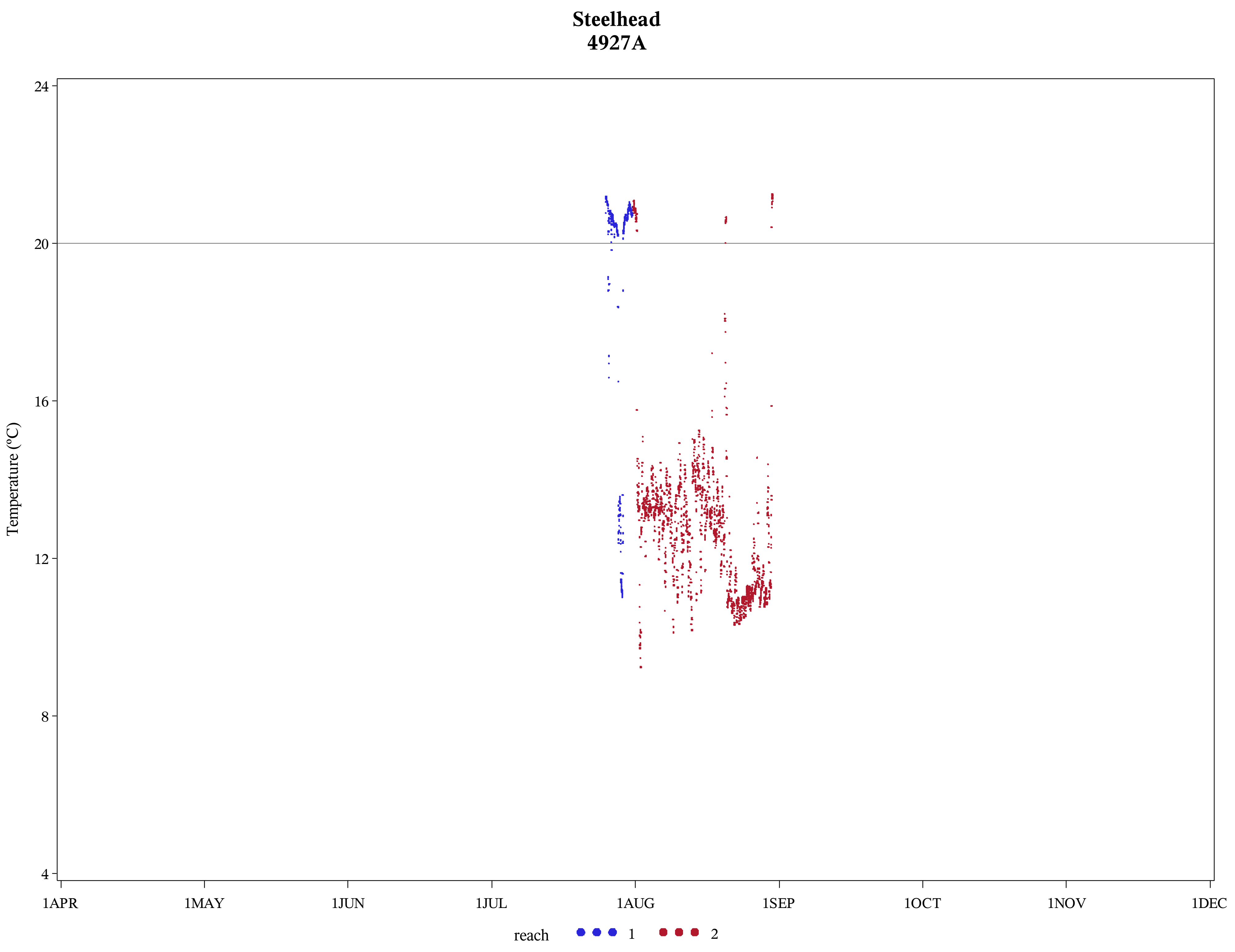
Steelhead
4927A
24
20
16
12
8
4
Temperature (°C)
1APR
1MAY
1JUN
1JUL
1AUG
1SEP
1OCT
1NOV
1DEC
reach
1
2

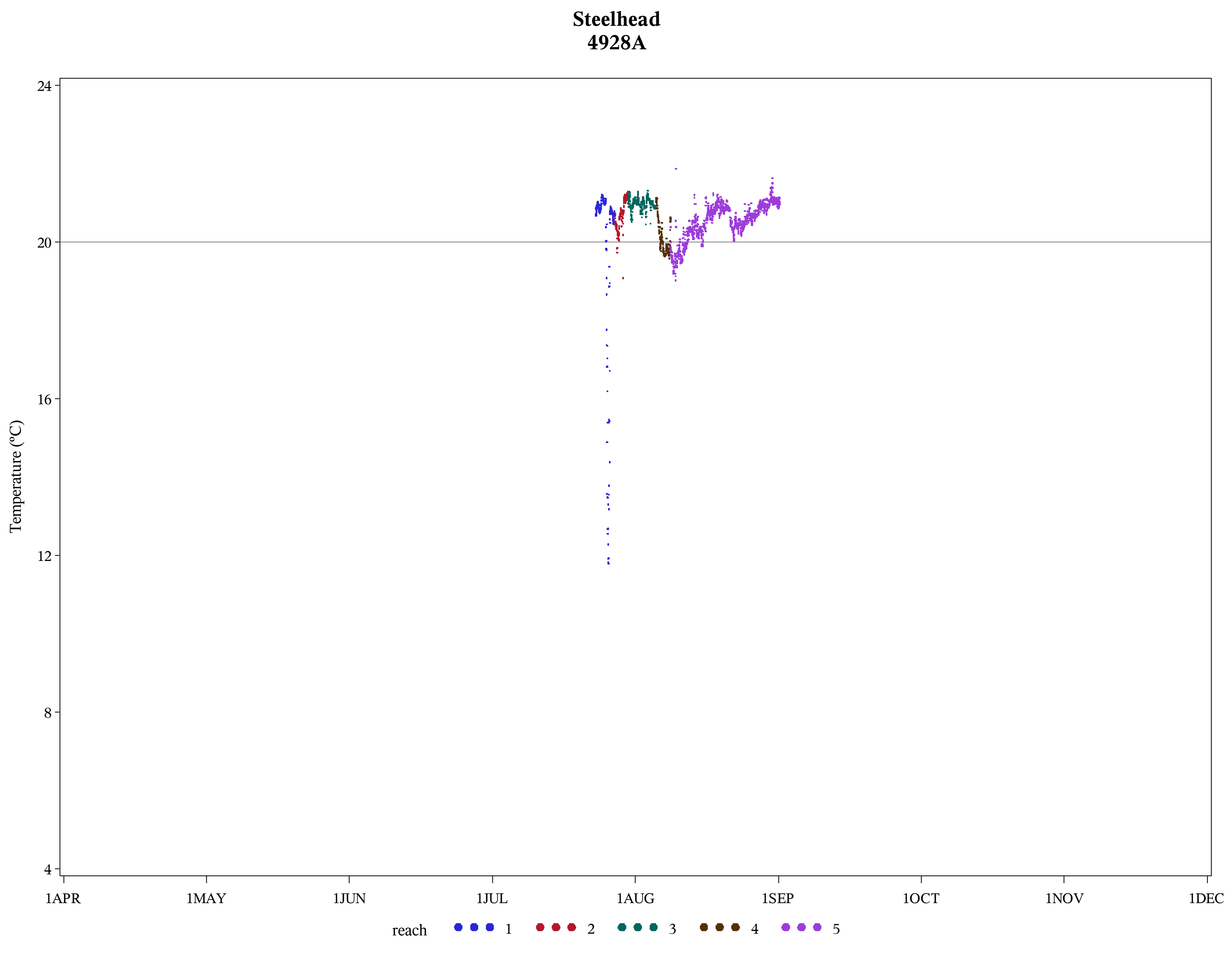

Steelhead
4928A
Temperature (°C)
24
20
16
12
8
4
1APR
1MAY
1JUN
1JUL
1AUG
1SEP
1OCT
1NOV
1DEC
reach
1
2
3
4
5

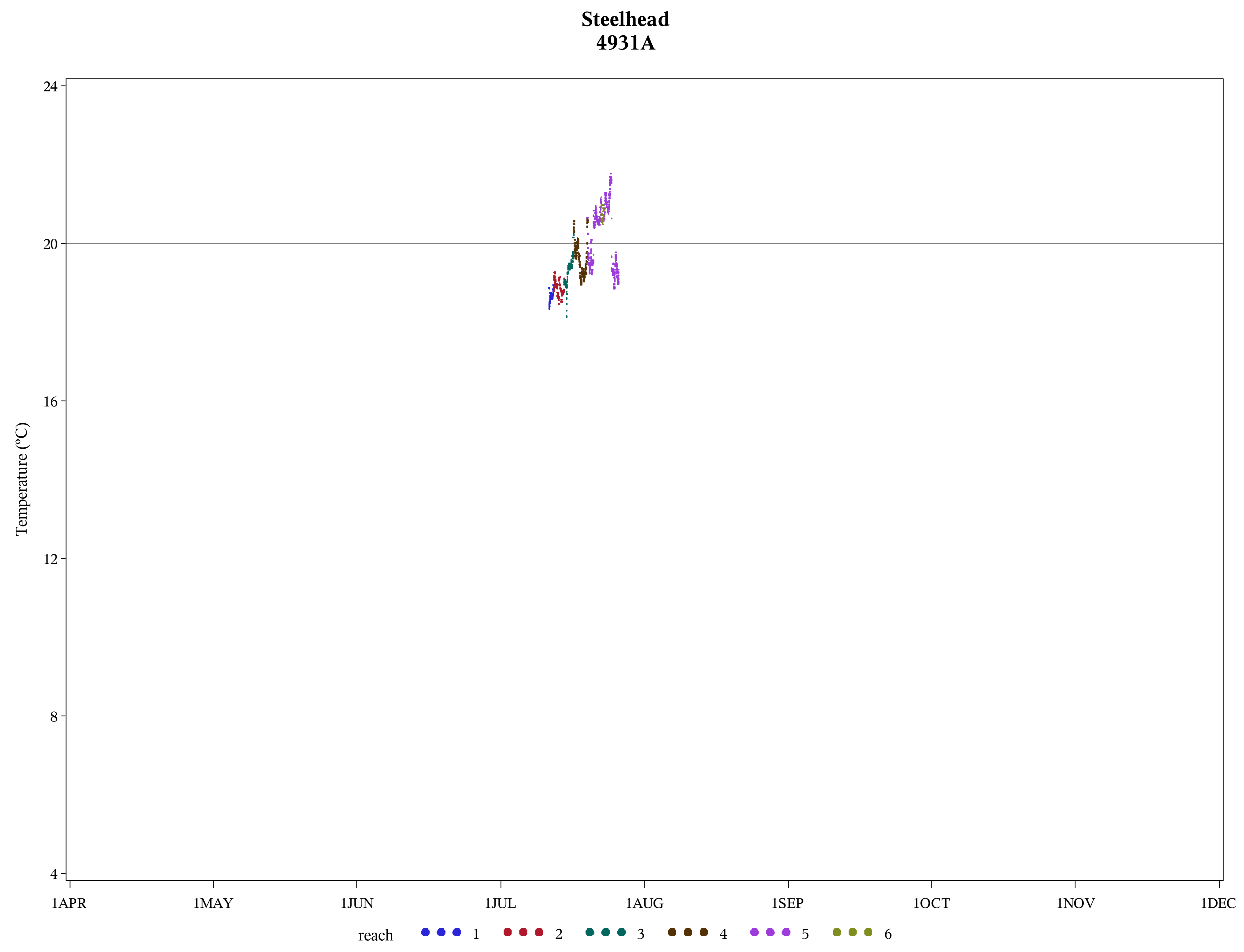
Steelhead
4931A
Temperature (°C)
24
20
16
12
8
4
1APR
1MAY
1JUN
1JUL
1AUG
1SEP
1OCT
1NOV
1DEC
reach
1
2
3
4
5
6

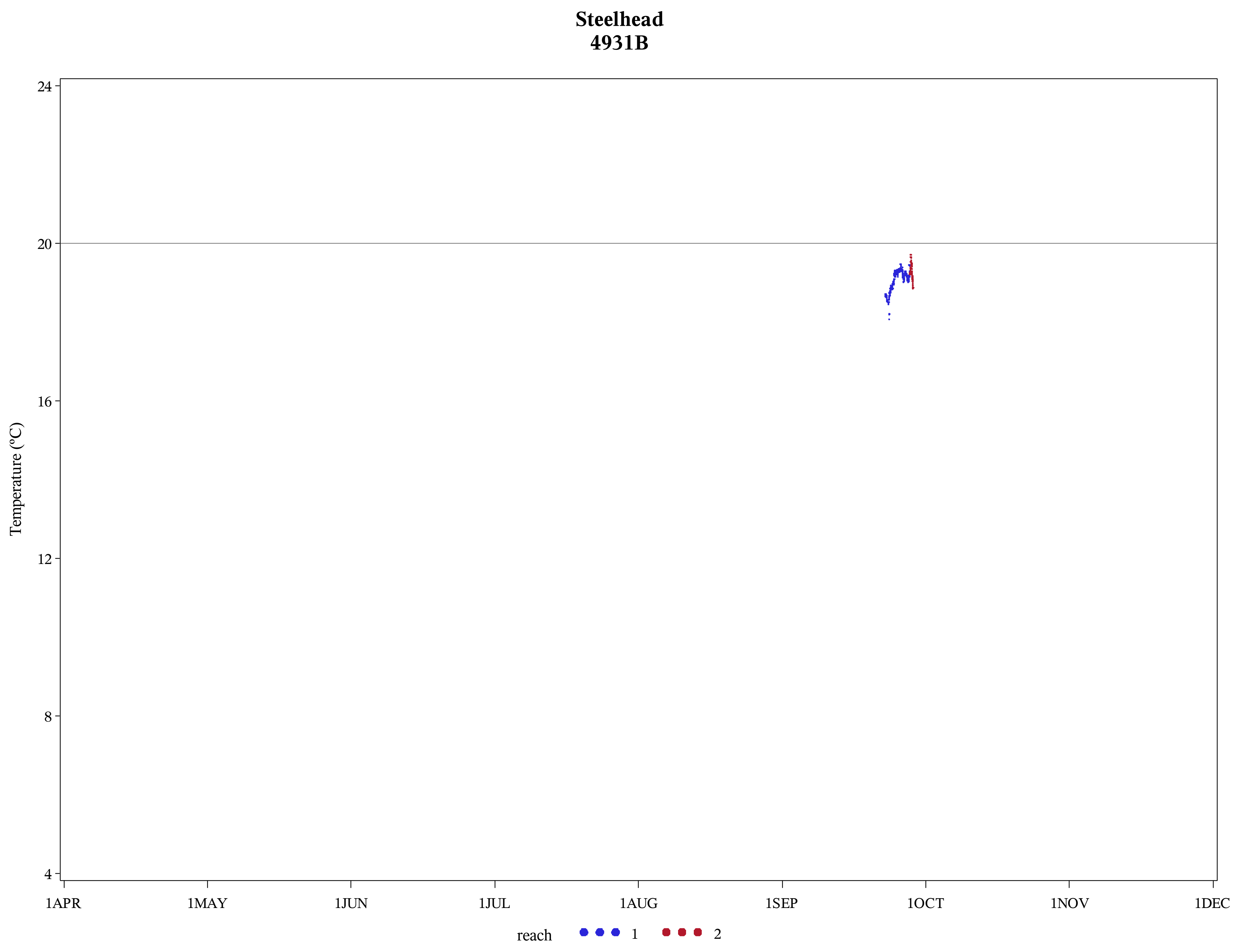

Steelhead
4931B
Temperature (°C)
24
20
16
12
8
4
1APR
1MAY
1JUN
1JUL
1AUG
1SEP
1OCT
1NOV
1DEC
reach
1
2

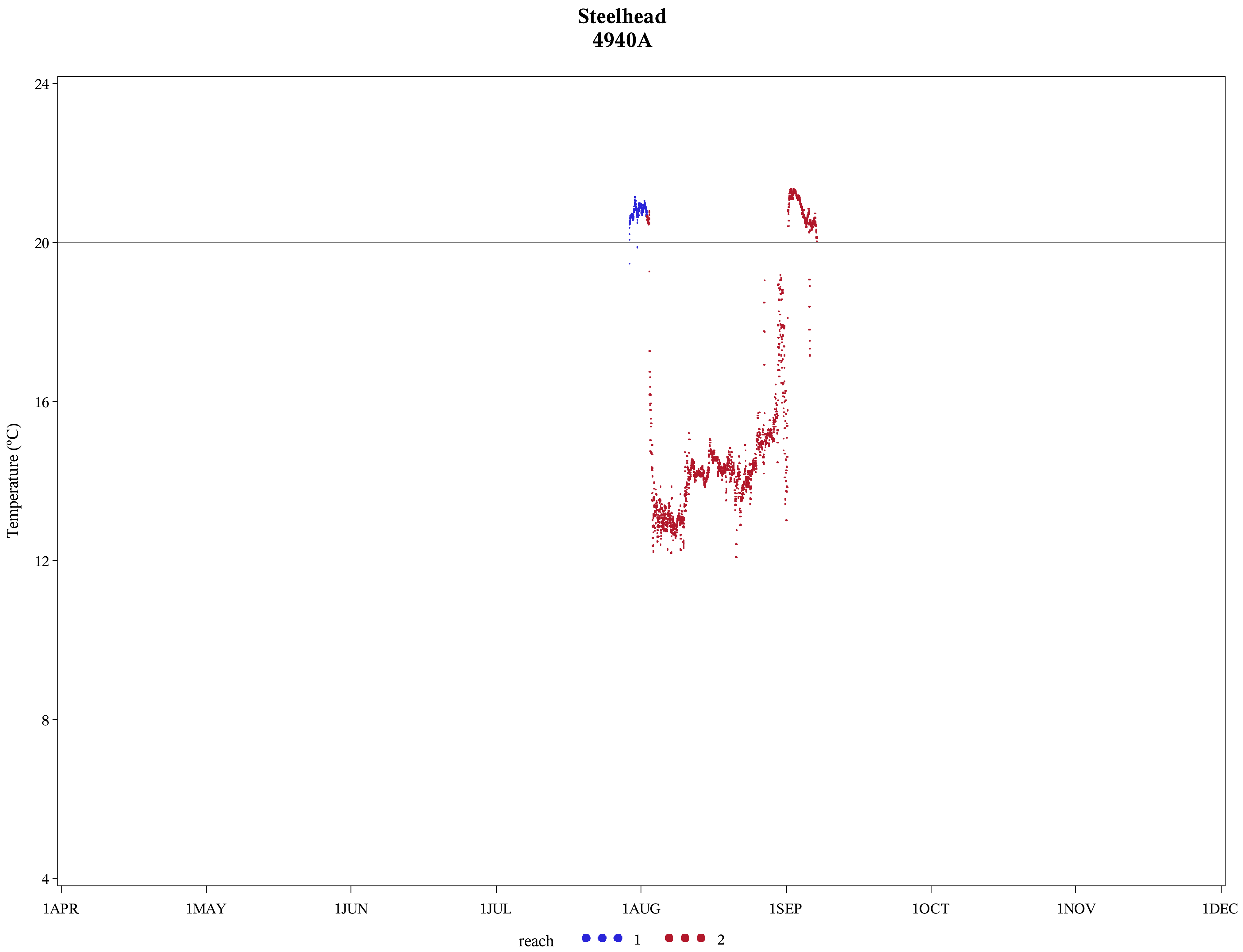
Steelhead
4940A
Temperature (°C)
24
20
16
12
8
4
1APR
1MAY
1JUN
1JUL
1AUG
1SEP
1OCT
1NOV
1DEC
reach
1
2

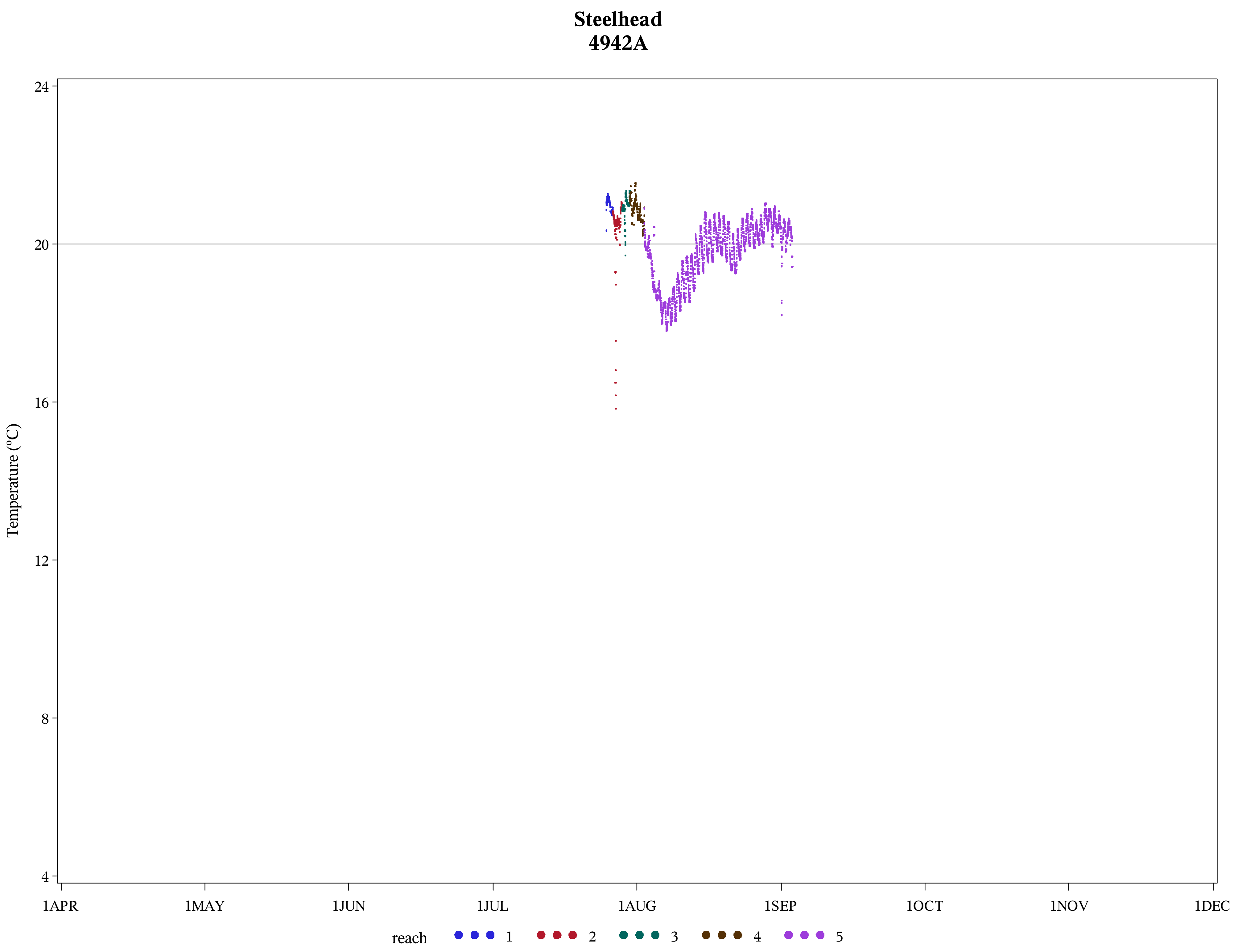

Steelhead
4942A
Temperature (°C)
24
20
16
12
8
4
1APR
1MAY
1JUN
1JUL
1AUG
1SEP
1OCT
1NOV
1DEC
reach
1
2
3
4
5

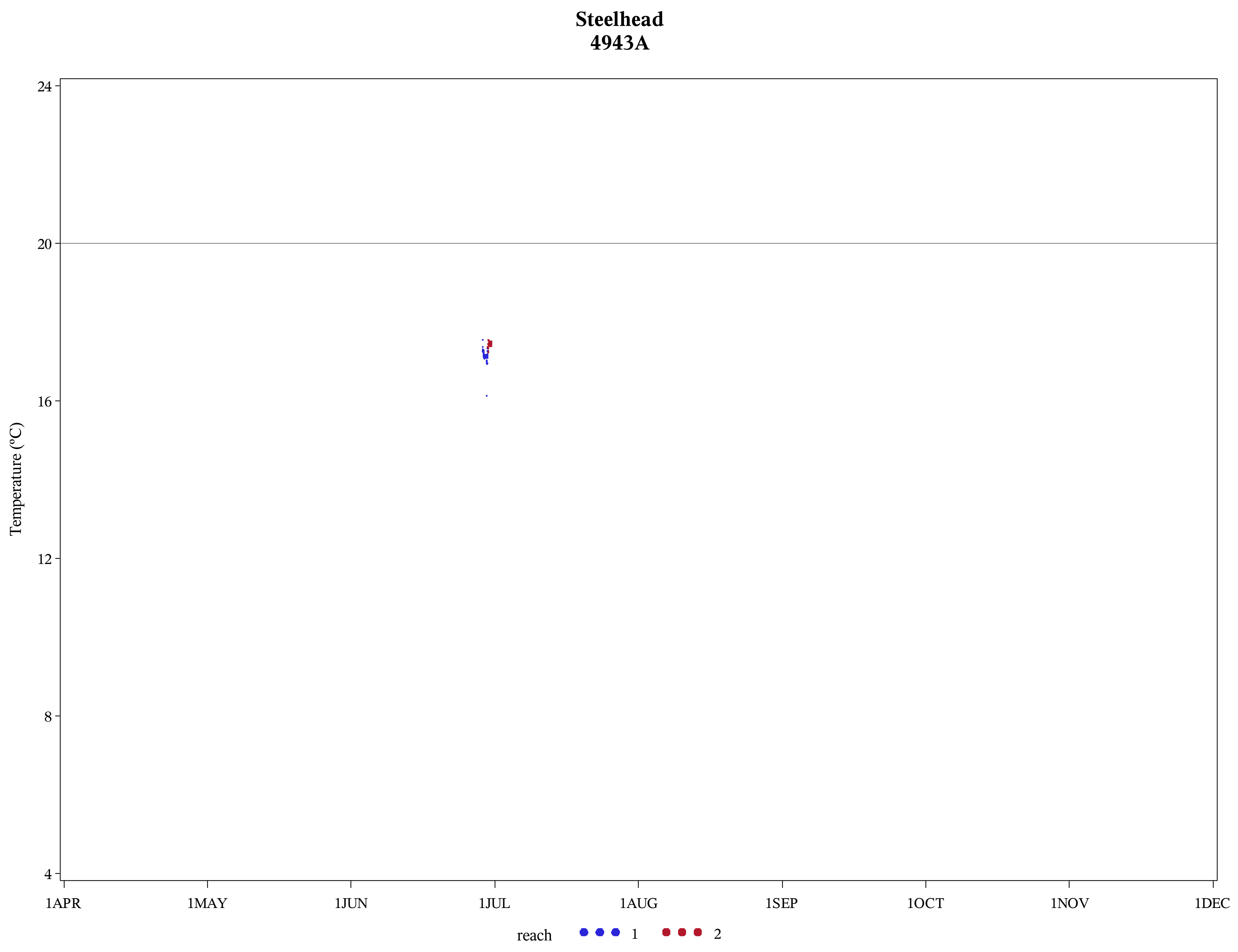

Steelhead
4943A
24
20
16
12
8
4
Temperature (°C)
1APR
1MAY
1JUN
1JUL
1AUG
1SEP
1OCT
1NOV
1DEC
reach
1
2

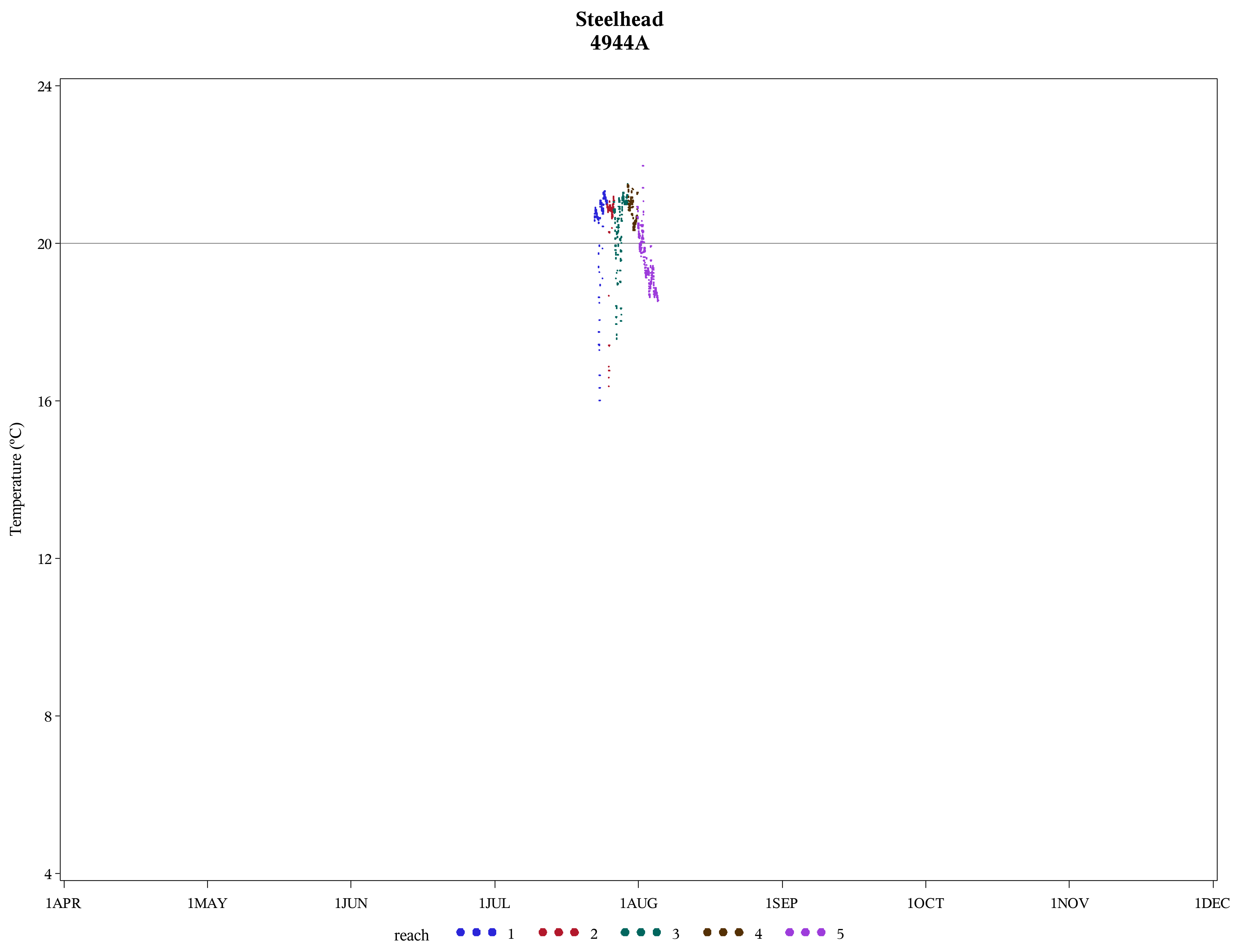

Steelhead
4944A
Temperature (°C)
24
20
16
12
8
4
1APR
1MAY
1JUN
1JUL
1AUG
1SEP
1OCT
1NOV
1DEC
reach
1
2
3
4
5

Steelhead
4947A

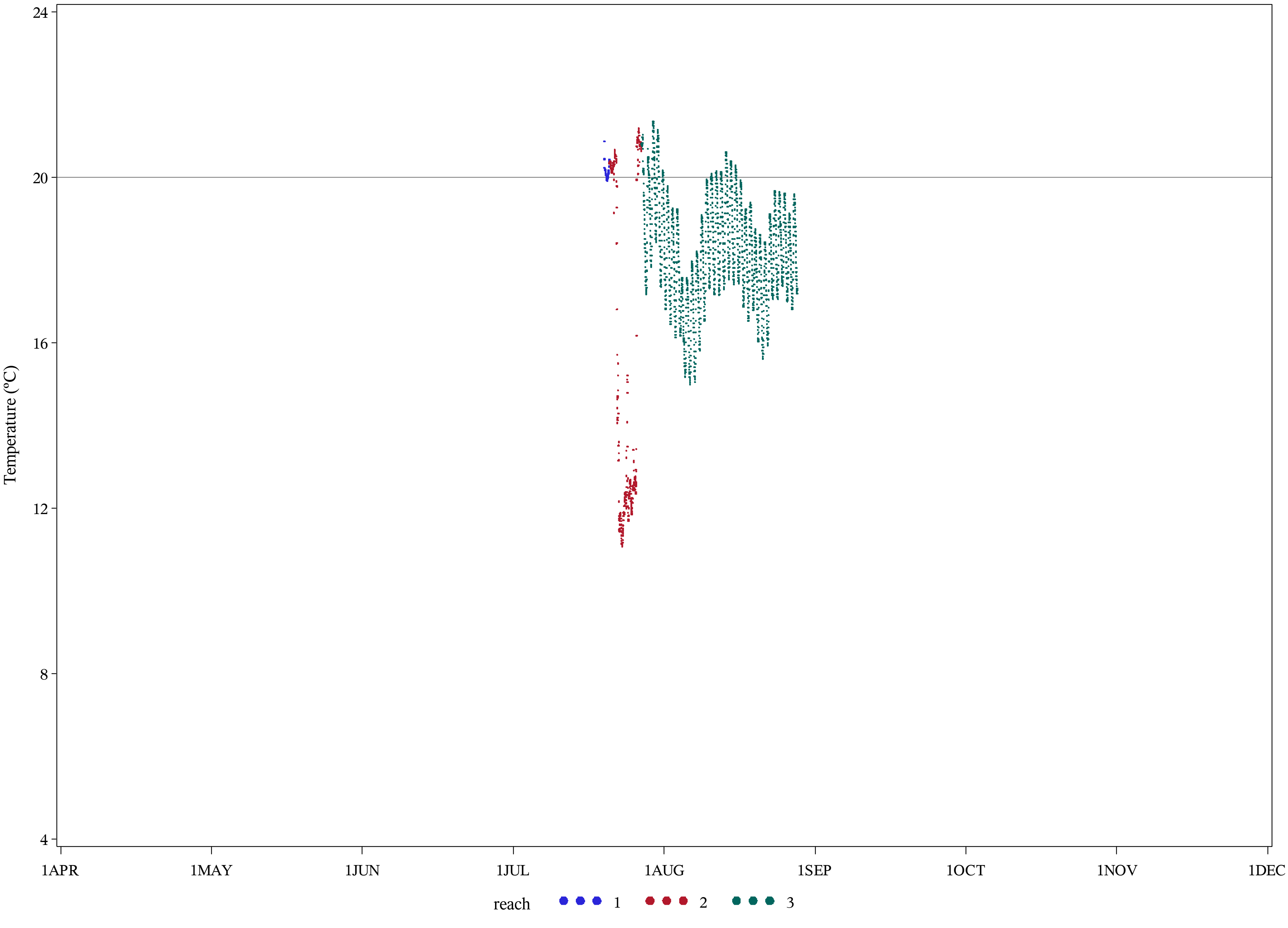

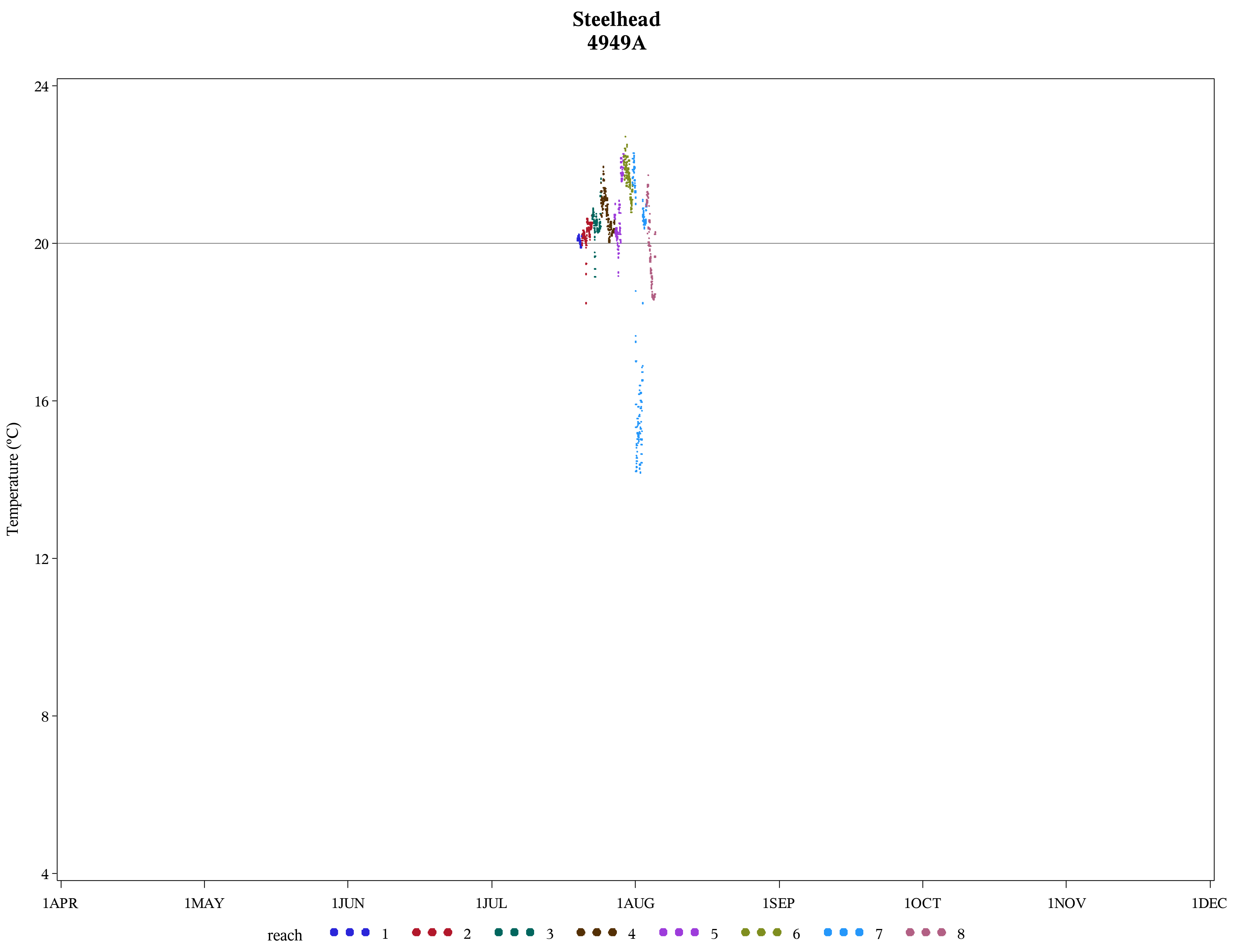

Steelhead
4949A
Temperature (°C)
24
20
16
12
8
4
1APR
1MAY
1JUN
1JUL
1AUG
1SEP
1OCT
1NOV
1DEC
reach
1
2
3
4
5
6
7
8

Steelhead
4949B

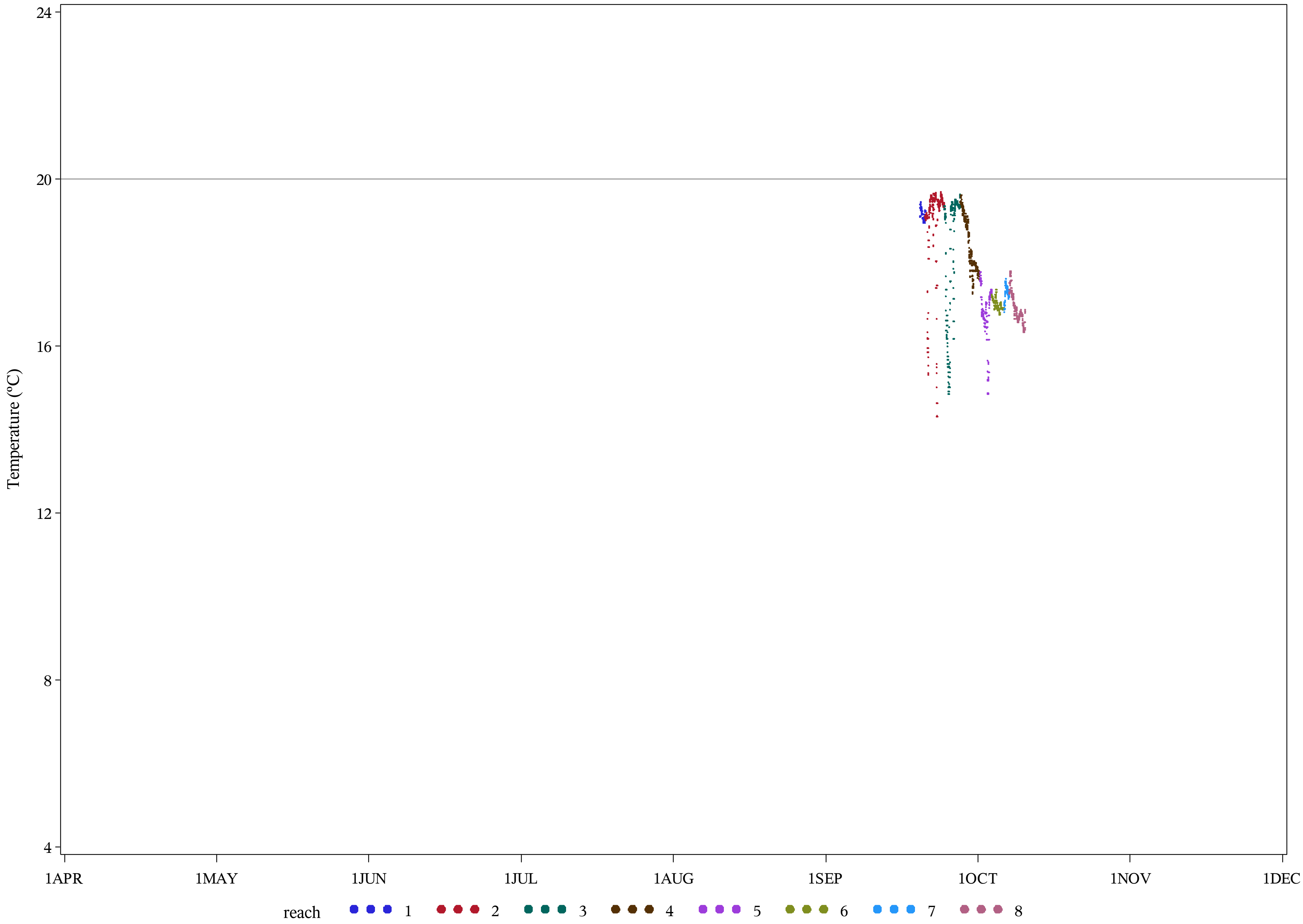

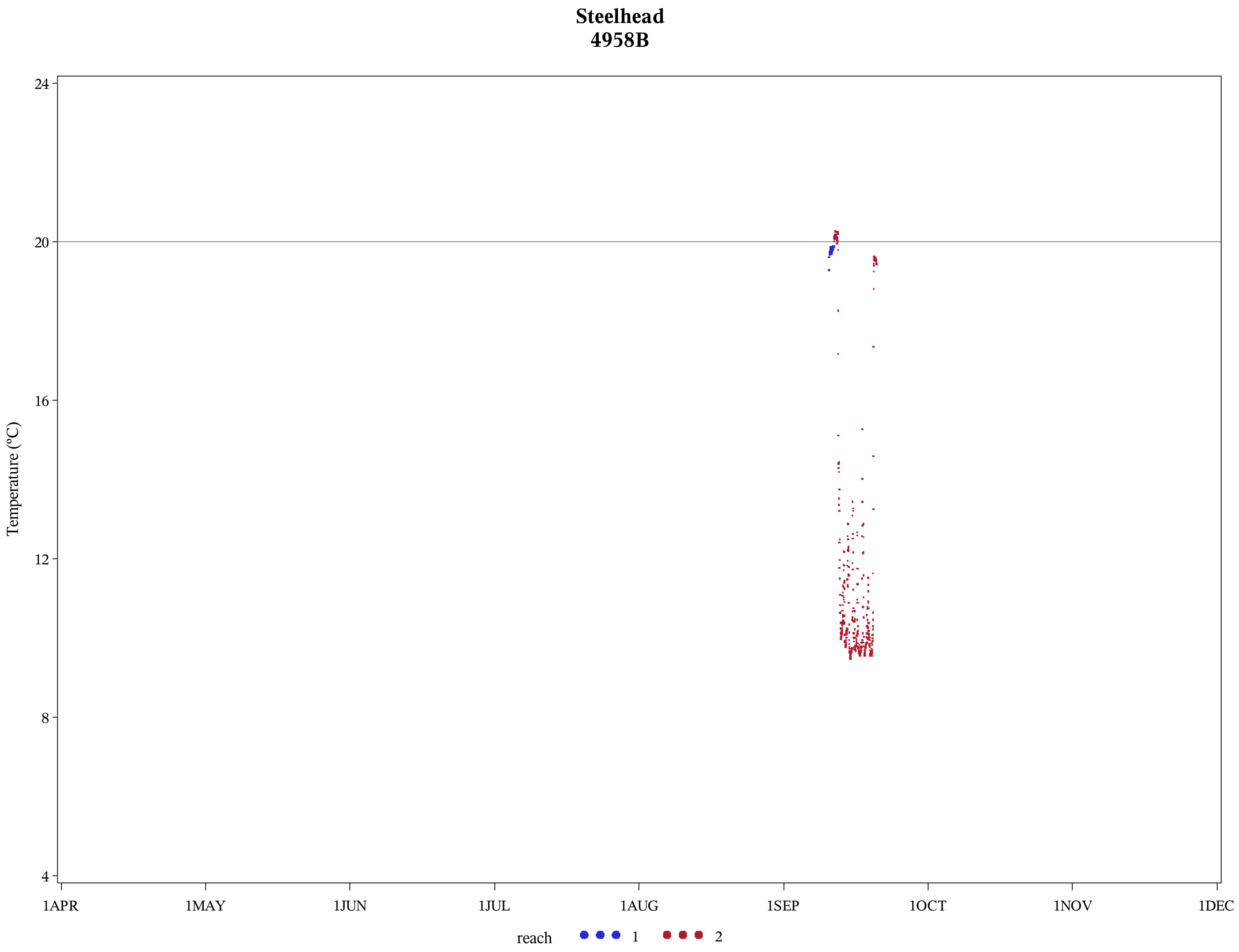

Steelhead
4958B
Temperature (°C)
24
20
16
12
8
4
1APR
1MAY
1JUN
1JUL
1AUG
1SEP
1OCT
1NOV
1DEC
reach
1
2

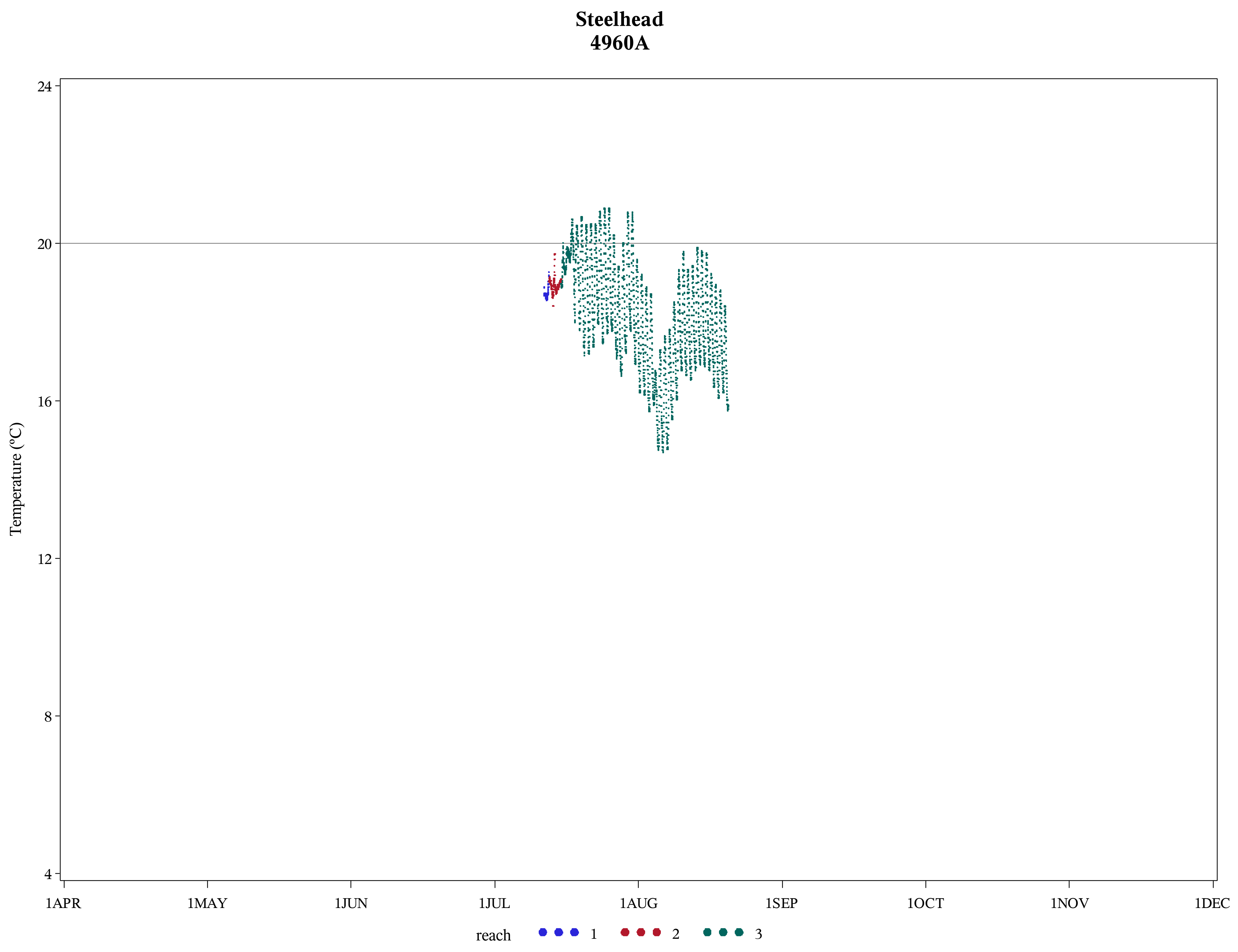
Steelhead
4960A
Temperature (°C)
24
20
16
12
8
4
1APR
1MAY
1JUN
1JUL
1AUG
1SEP
1OCT
1NOV
1DEC
reach
1
2
3

Steelhead
4962A

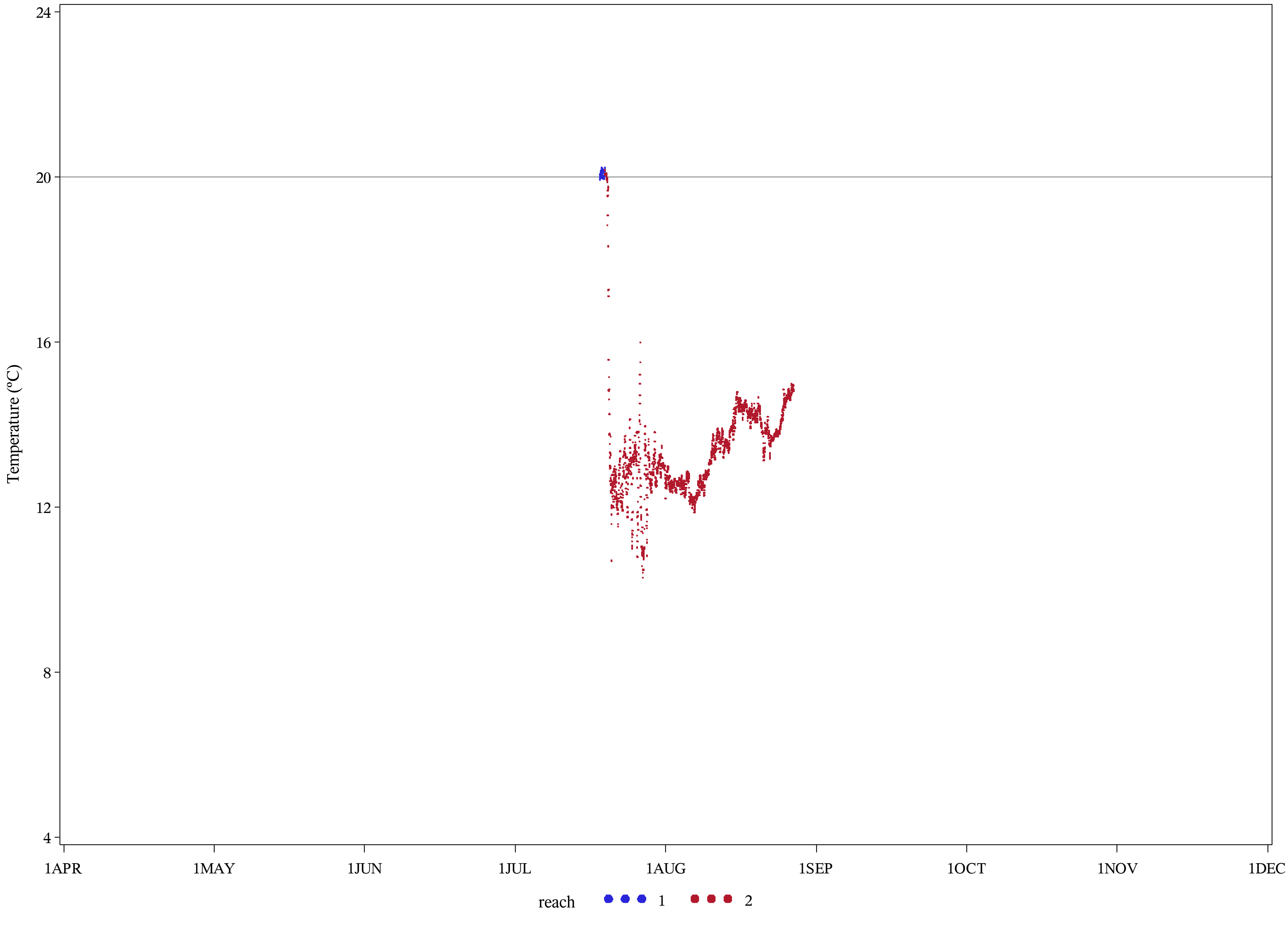

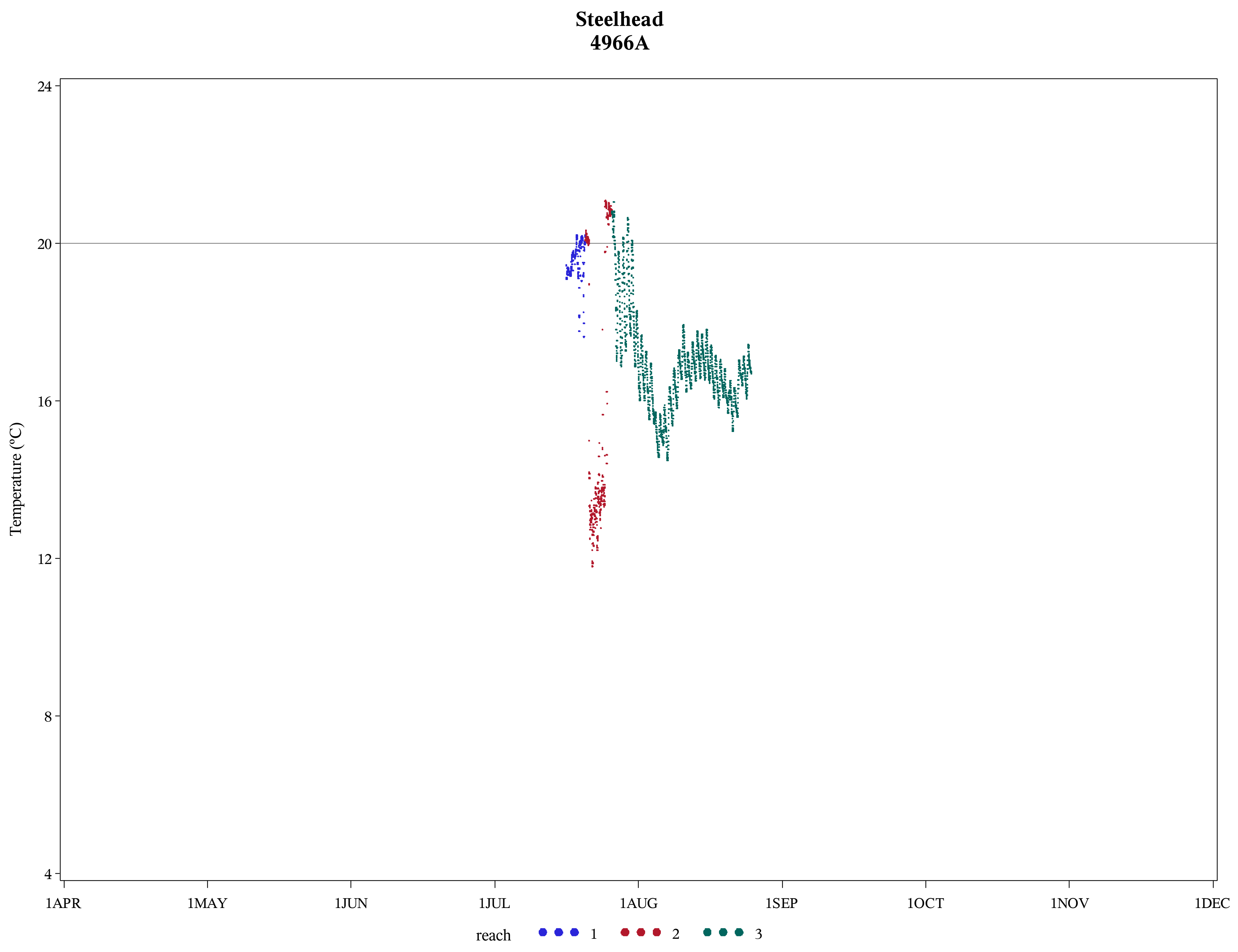
Steelhead
4966A
Temperature (°C)
24
20
16
12
8
4
1APR
1MAY
1JUN
1JUL
1AUG
1SEP
1OCT
1NOV
1DEC
reach
1
2
3

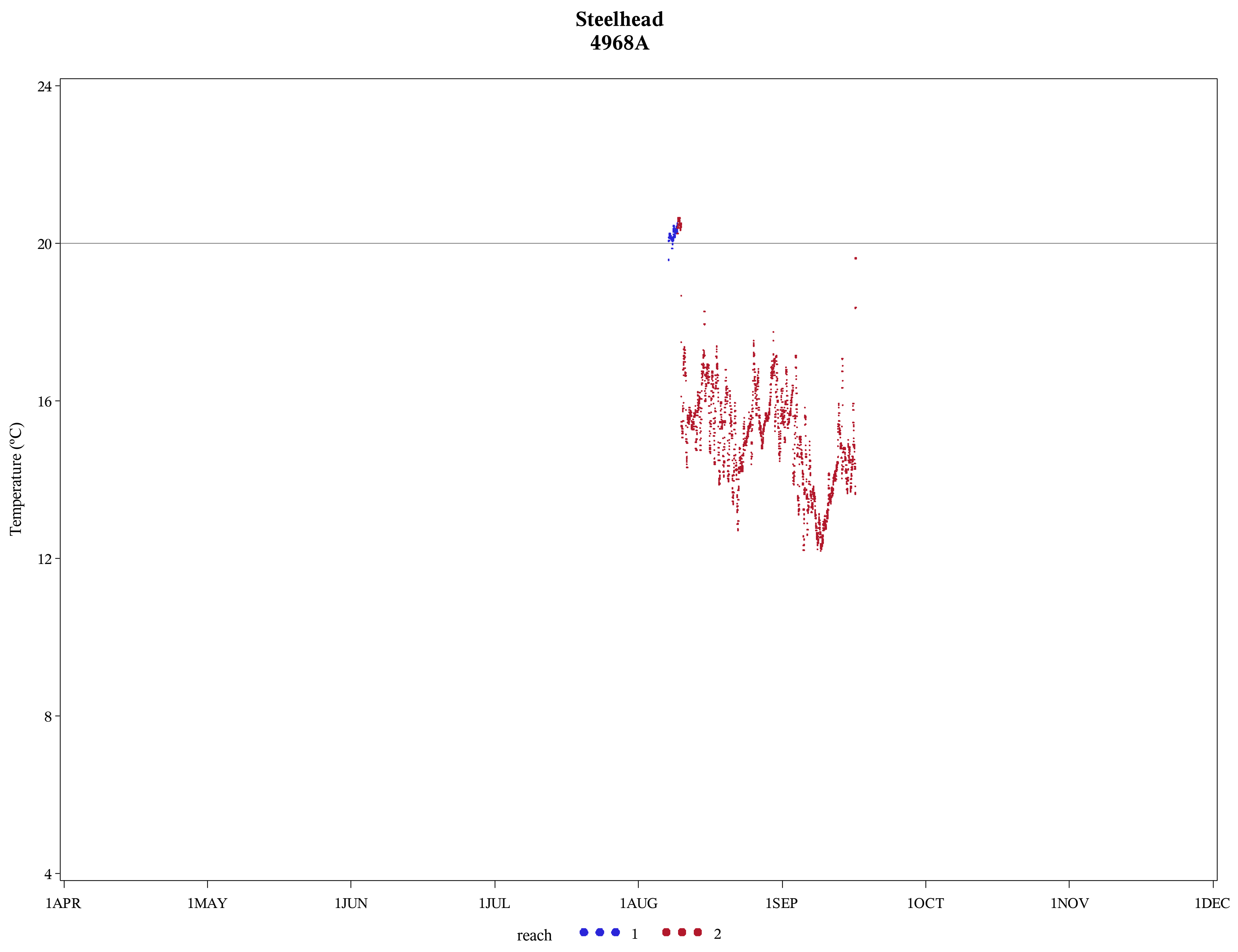

Steelhead
4968A
Temperature (°C)
24
20
16
12
8
4
1APR
1MAY
1JUN
1JUL
1AUG
1SEP
1OCT
1NOV
1DEC
reach
1
2

Steelhead
4969A

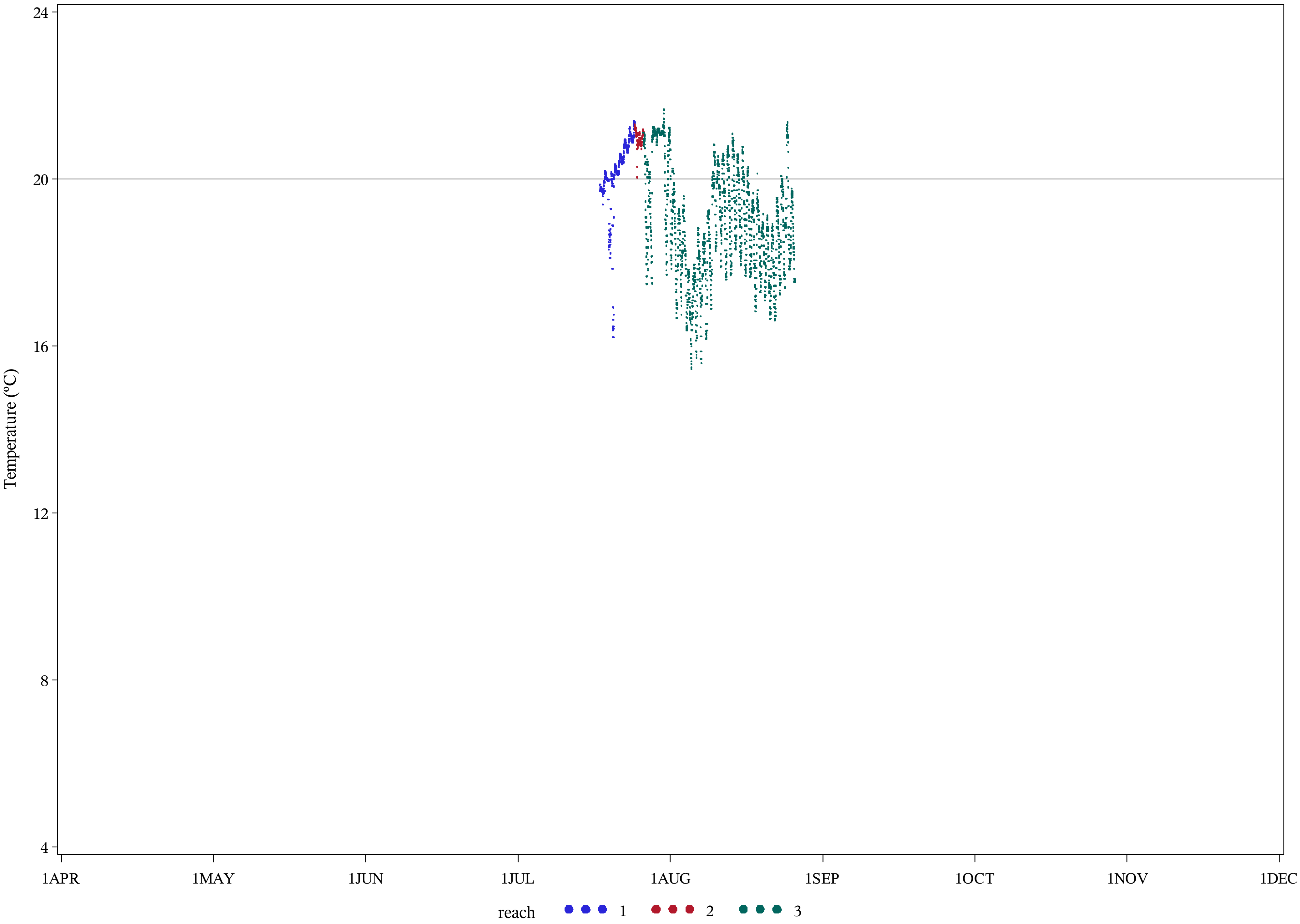

Steelhead
4972A

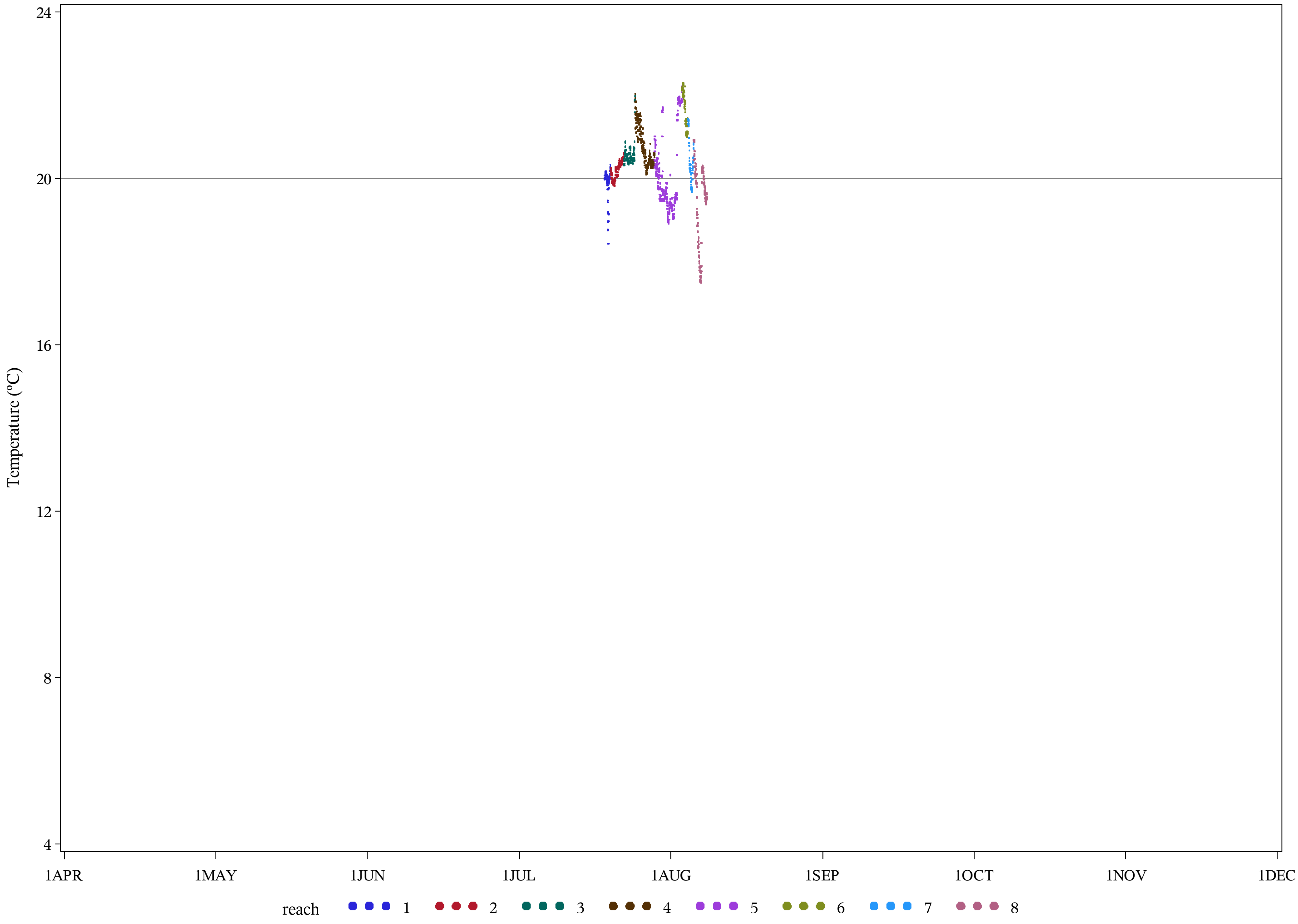

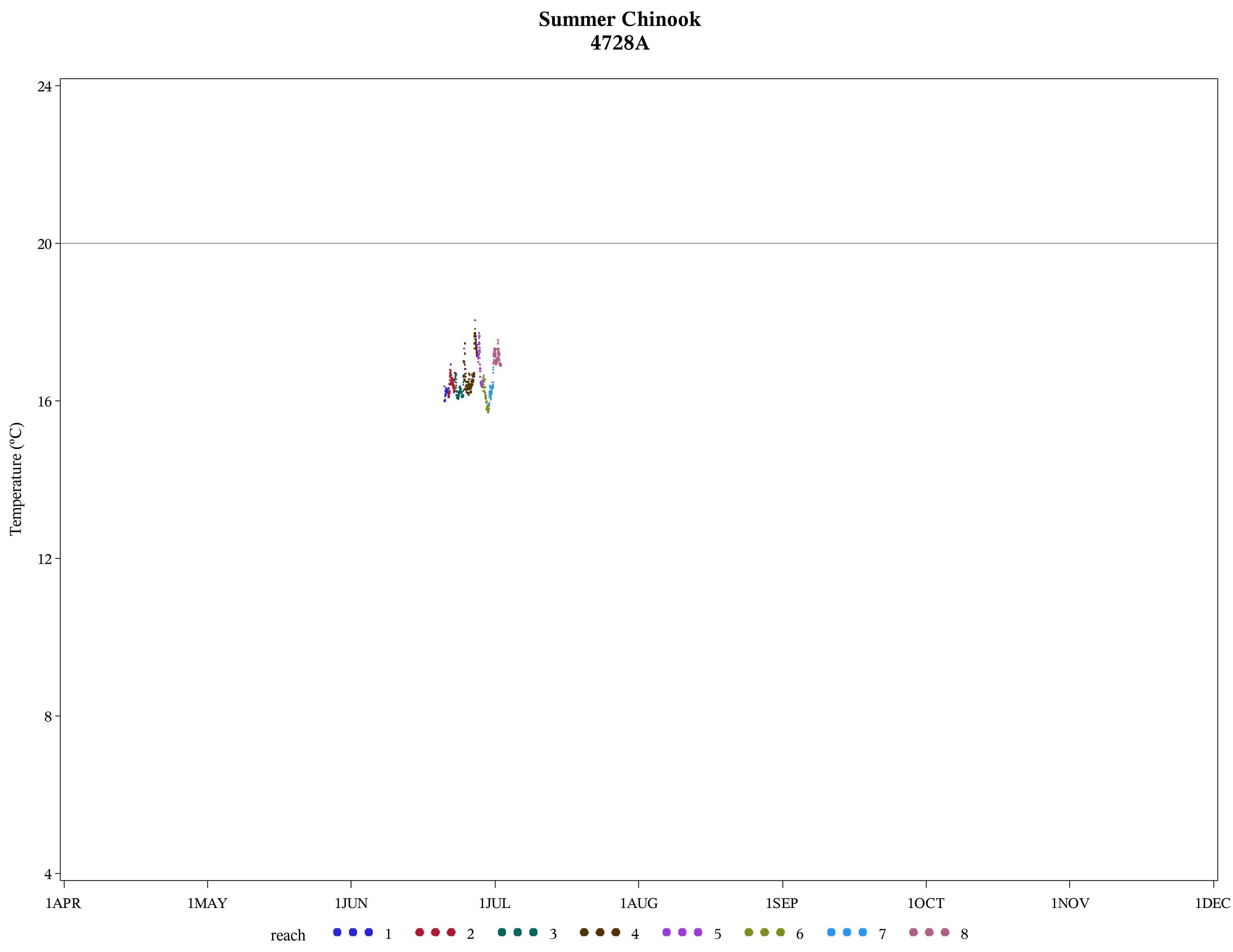

Summer Chinook
4728A
Temperature (°C)
24
20
16
12
8
4
1APR
1MAY
1JUN
1JUL
1AUG
1SEP
1OCT
1NOV
1DEC
reach
1
2
3
4
5
6
7
8

Summer Chinook
4730A

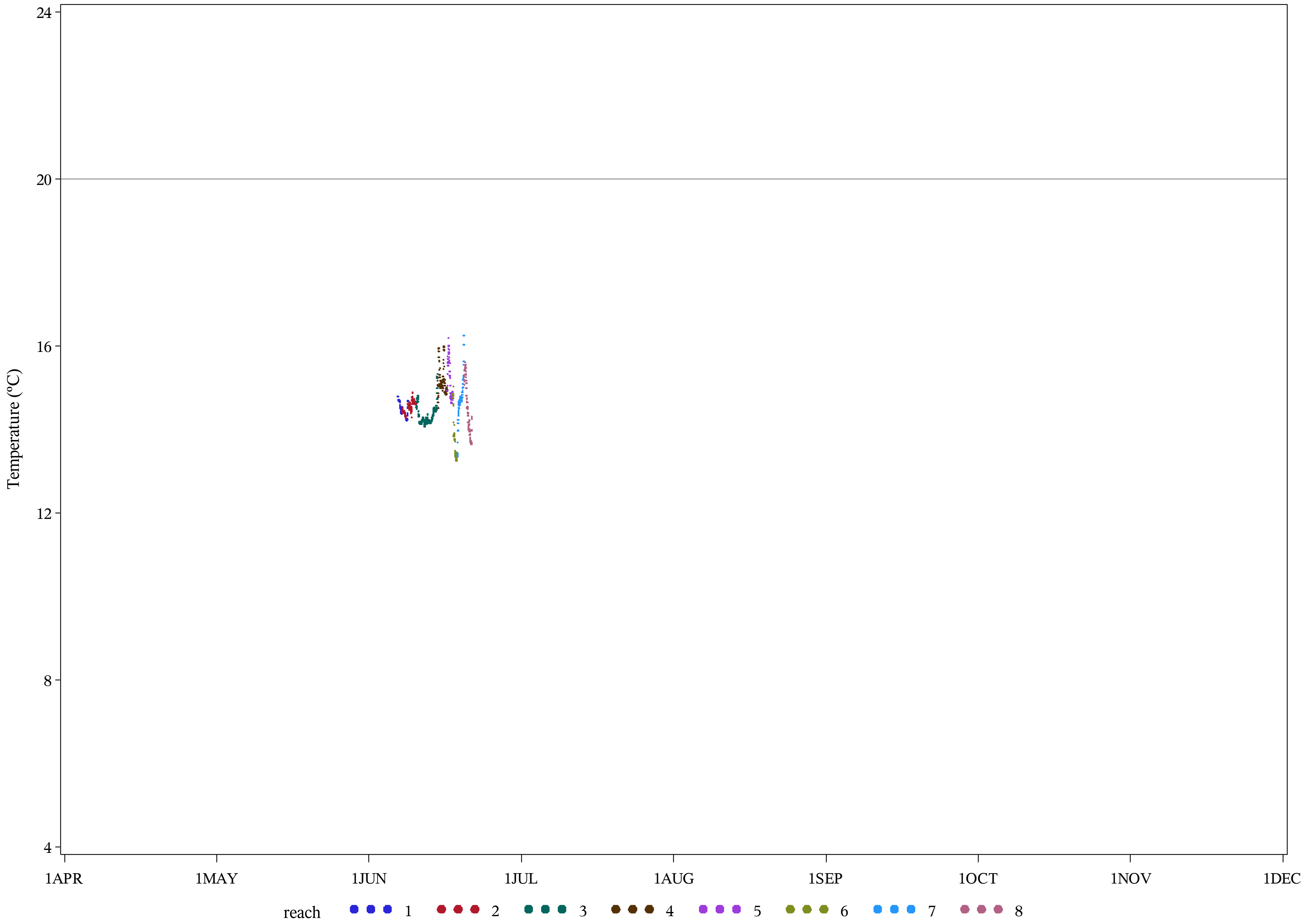

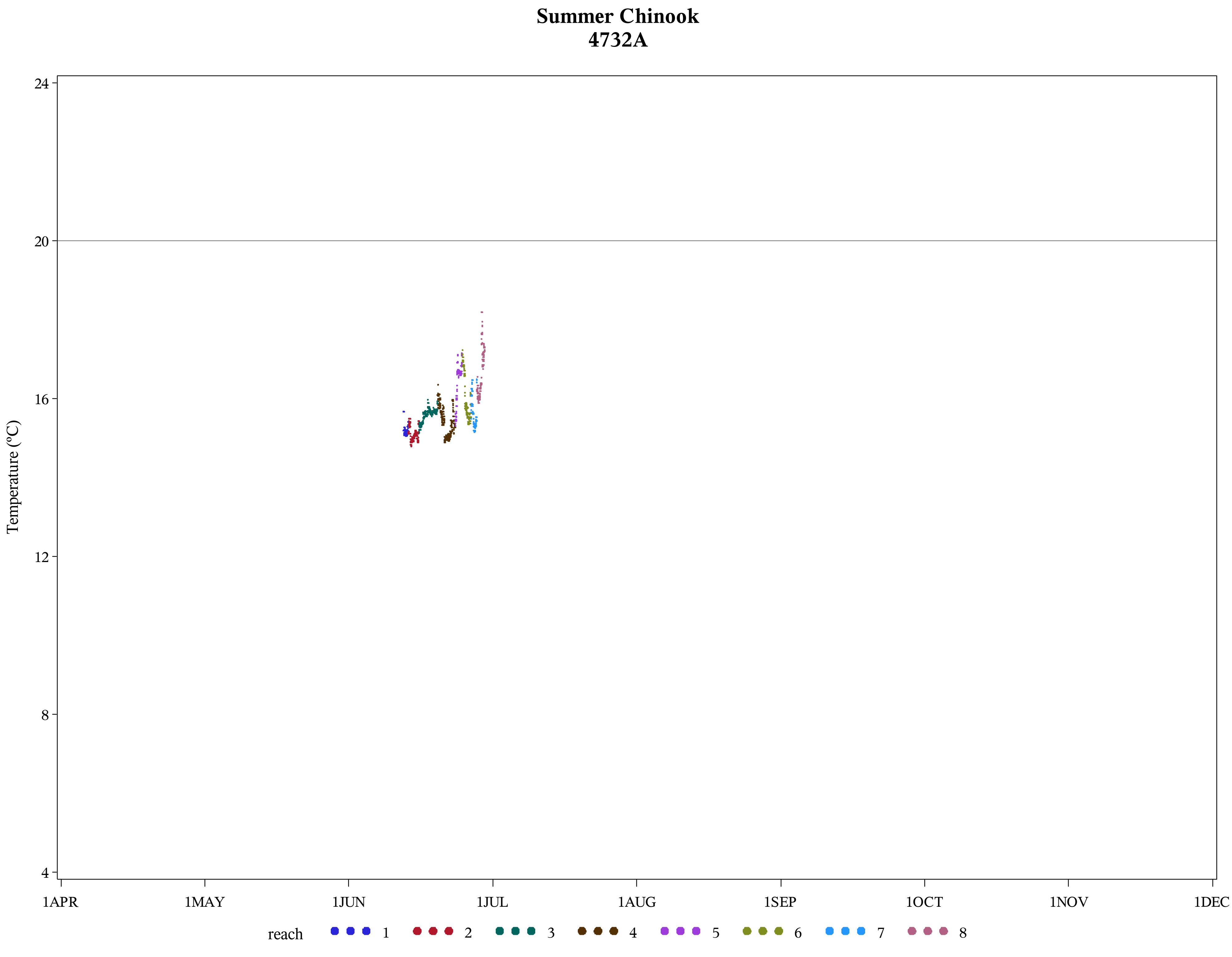

Summer Chinook
4732A
Temperature (°C)
24
20
16
12
8
4
1APR
1MAY
1JUN
1JUL
1AUG
1SEP
1OCT
1NOV
1DEC
reach
1
2
3
4
5
6
7
8

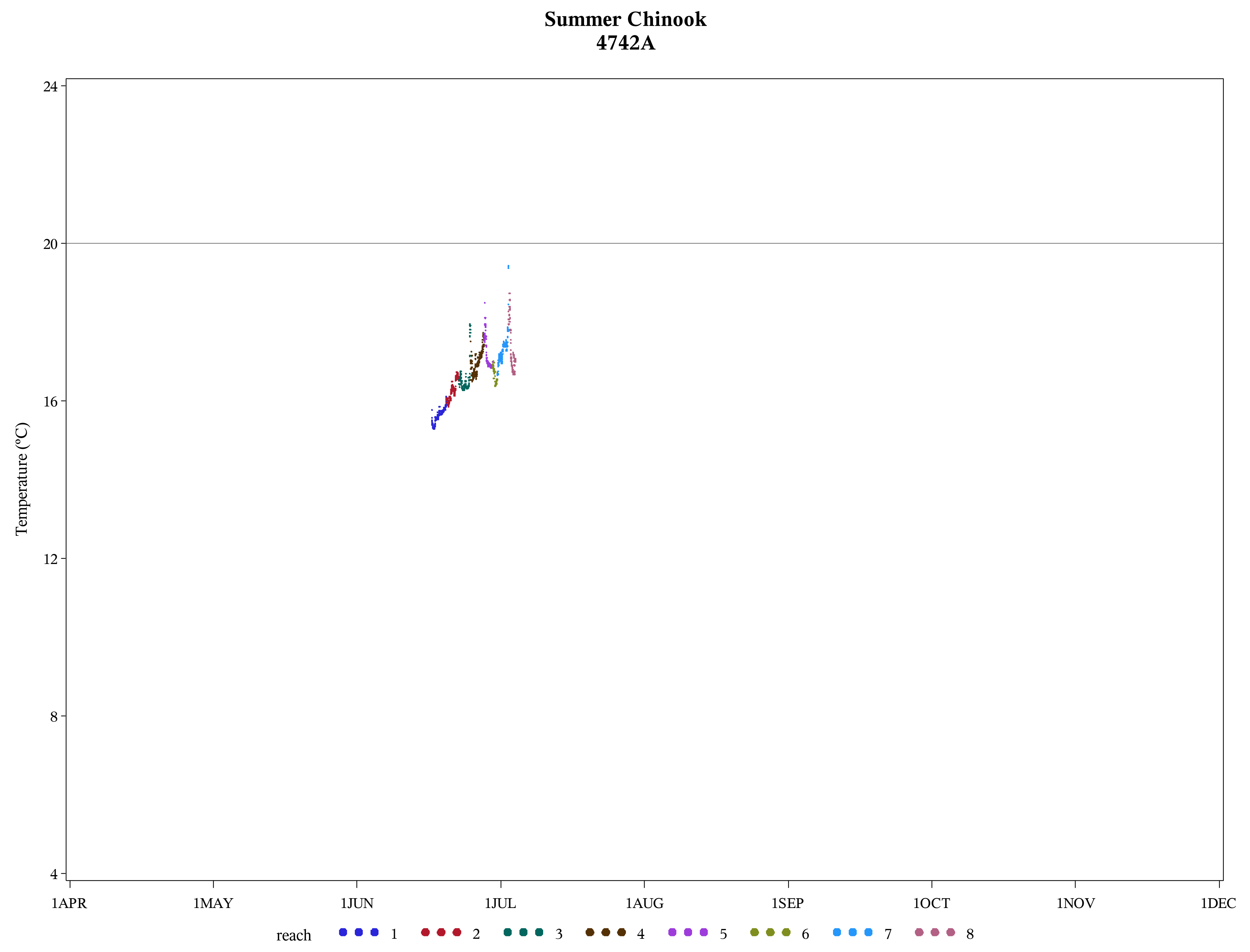

Summer Chinook
4742A
Temperature (°C)
24
20
16
12
8
4
1APR
1MAY
1JUN
1JUL
1AUG
1SEP
1OCT
1NOV
1DEC
reach
1
2
3
4
5
6
7
8

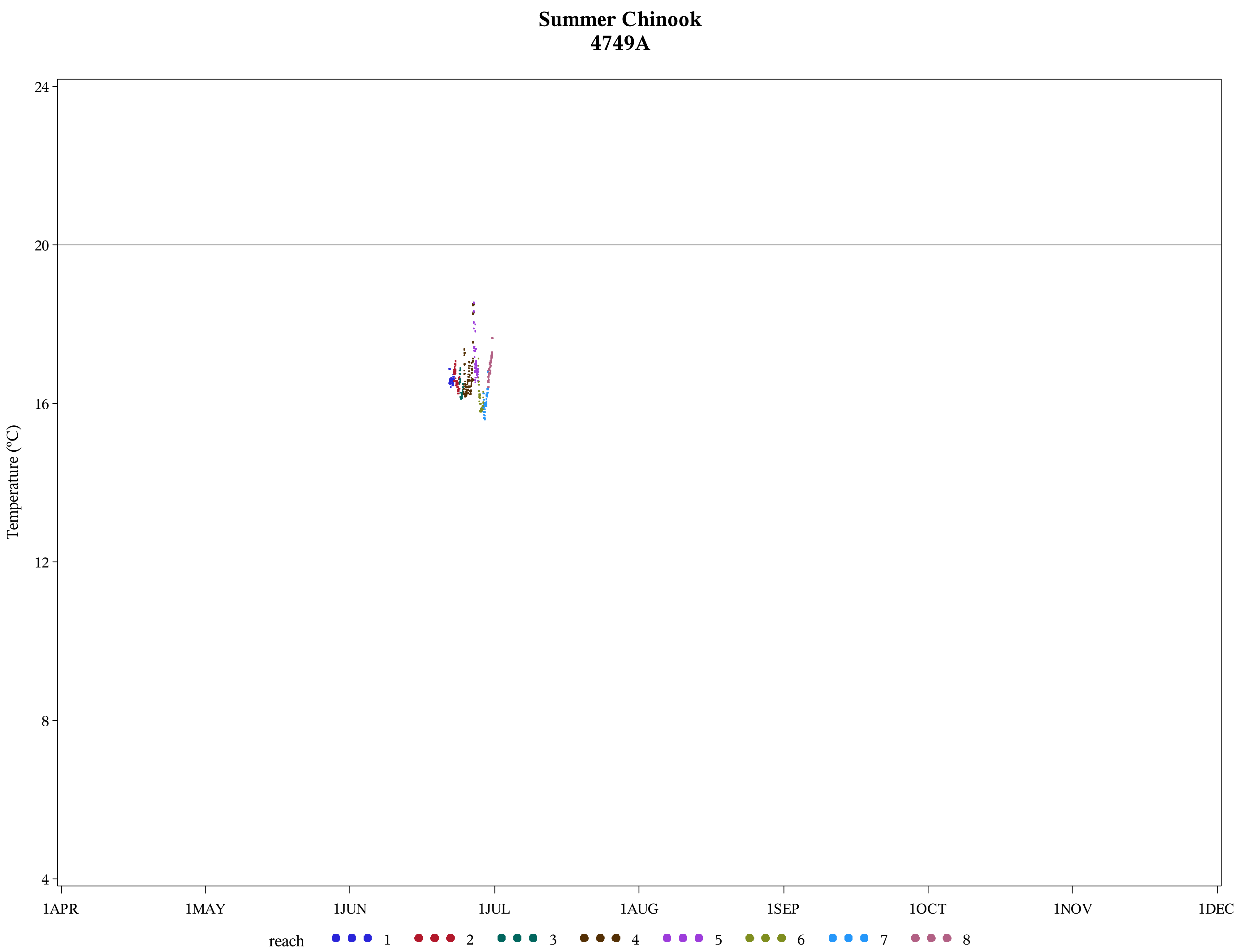
Summer Chinook
4749A
Temperature (°C)
24
20
16
12
8
4
1APR
1MAY
1JUN
1JUL
1AUG
1SEP
1OCT
1NOV
1DEC
reach
1
2
3
4
5
6
7
8

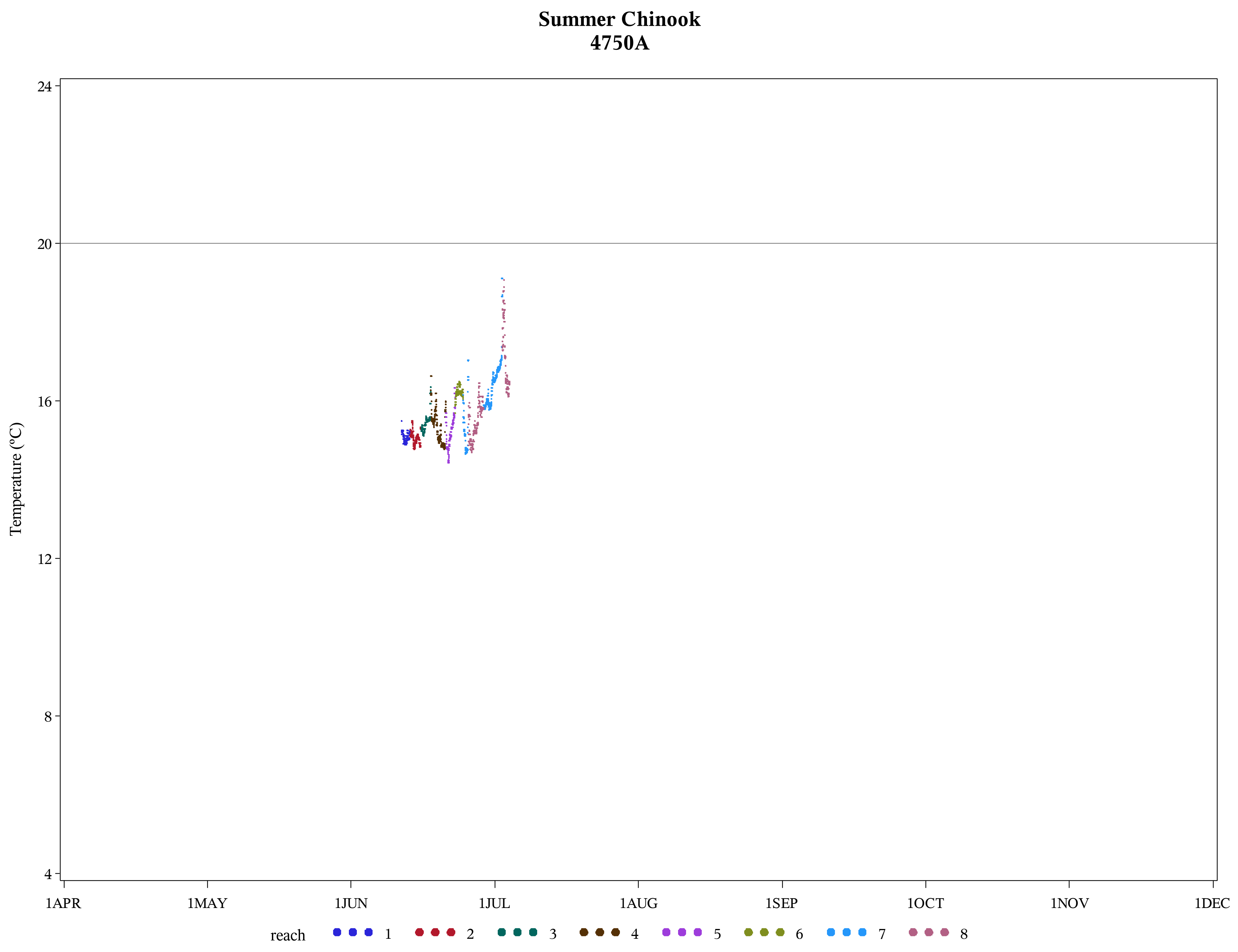
Summer Chinook
4750A
Temperature (°C)
24
20
16
12
8
4
1APR
1MAY
1JUN
1JUL
1AUG
1SEP
1OCT
1NOV
1DEC
reach
1
2
3
4
5
6
7
8

Summer Chinook
4752A

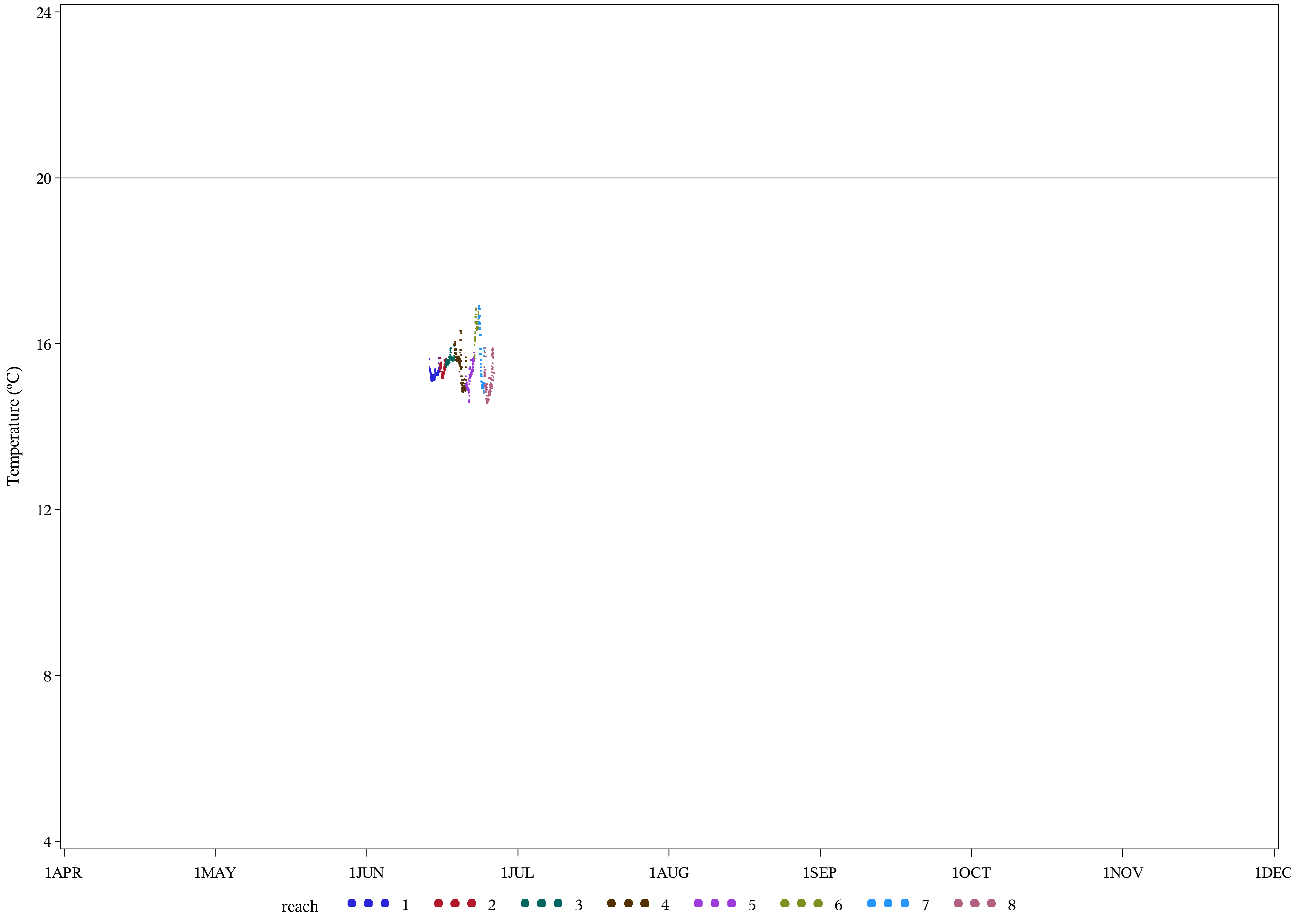

Summer Chinook
4757A

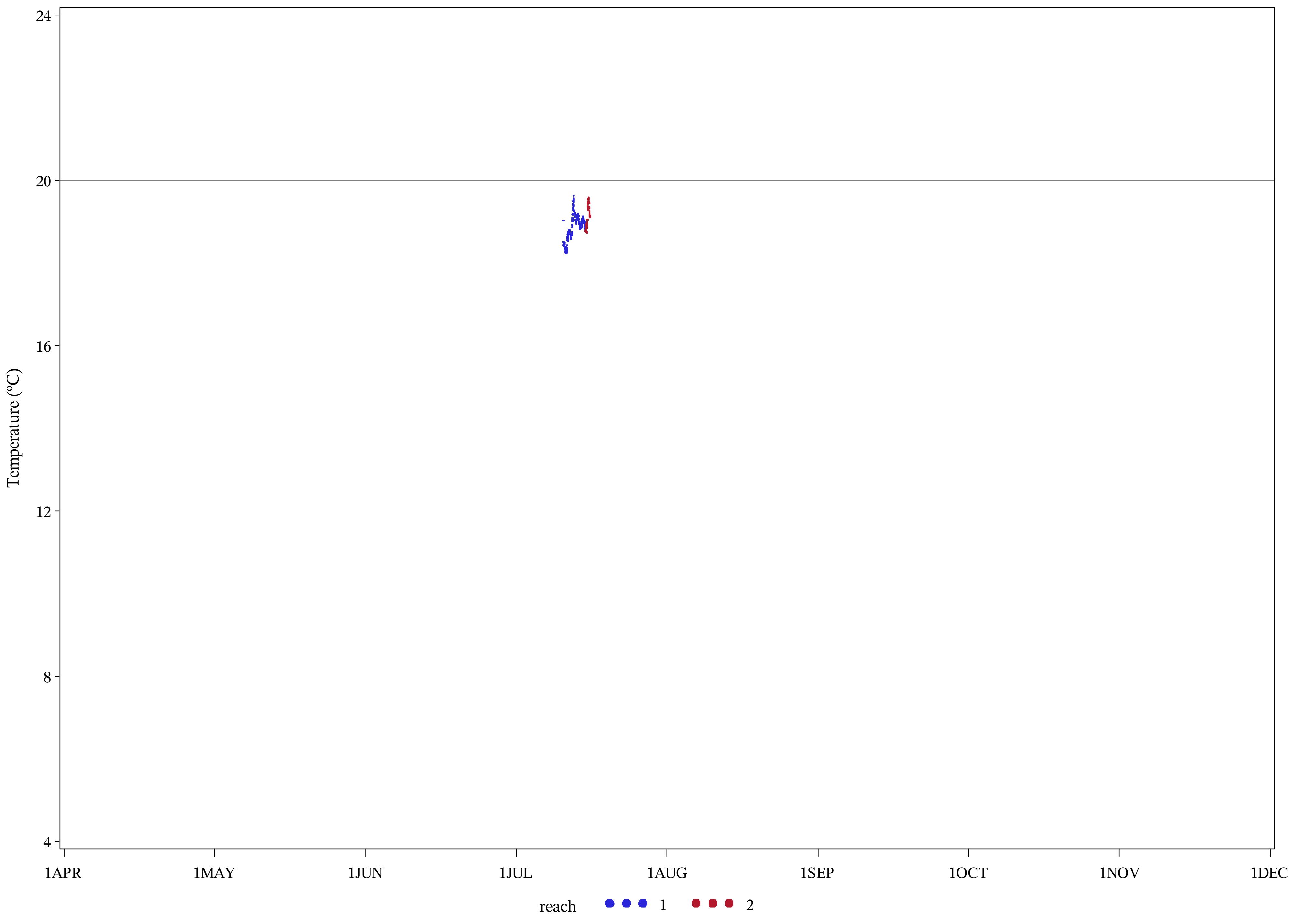

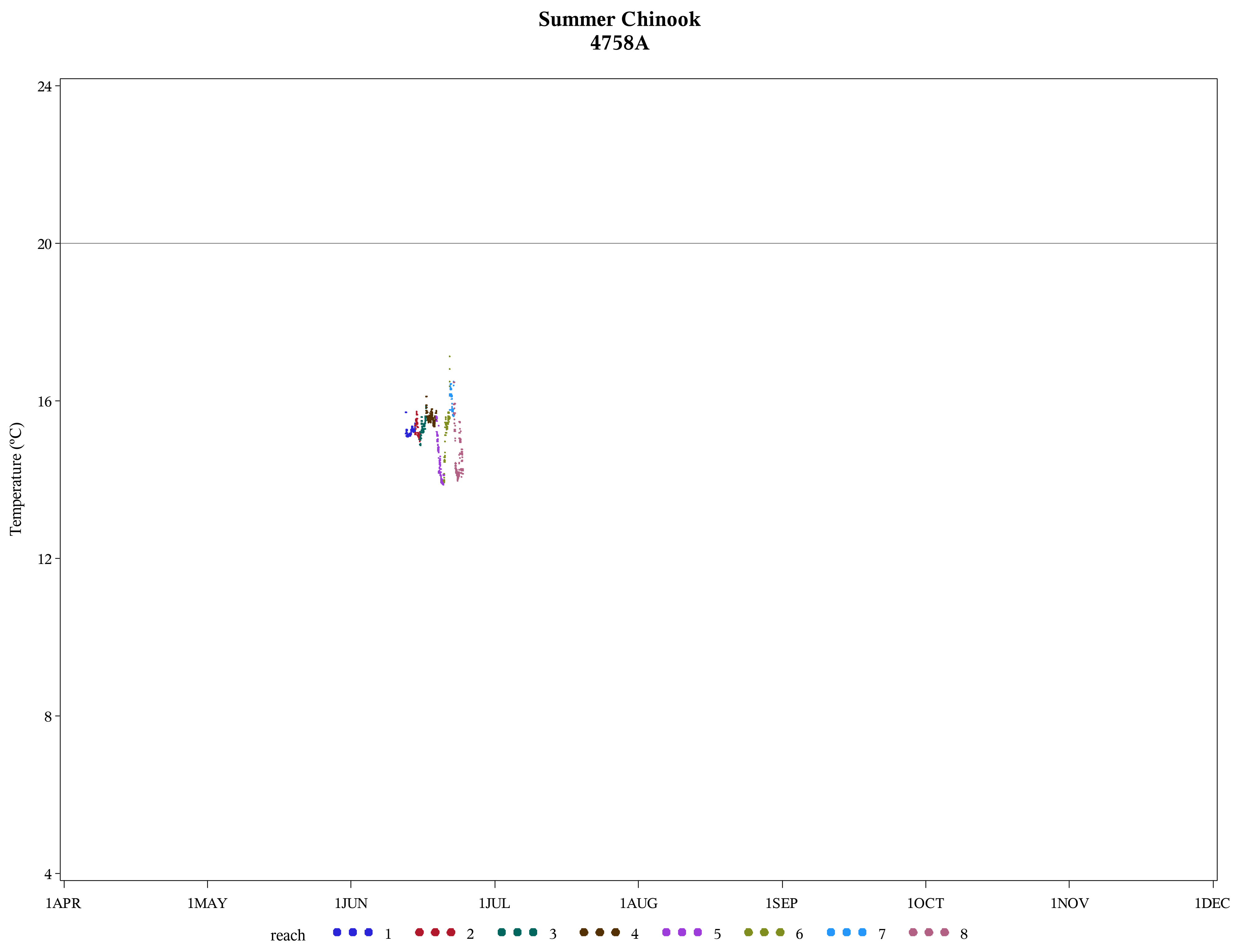

Summer Chinook
4758A
Temperature (°C)
24
20
16
12
8
4
1APR
1MAY
1JUN
1JUL
1AUG
1SEP
1OCT
1NOV
1DEC
reach
1
2
3
4
5
6
7
8

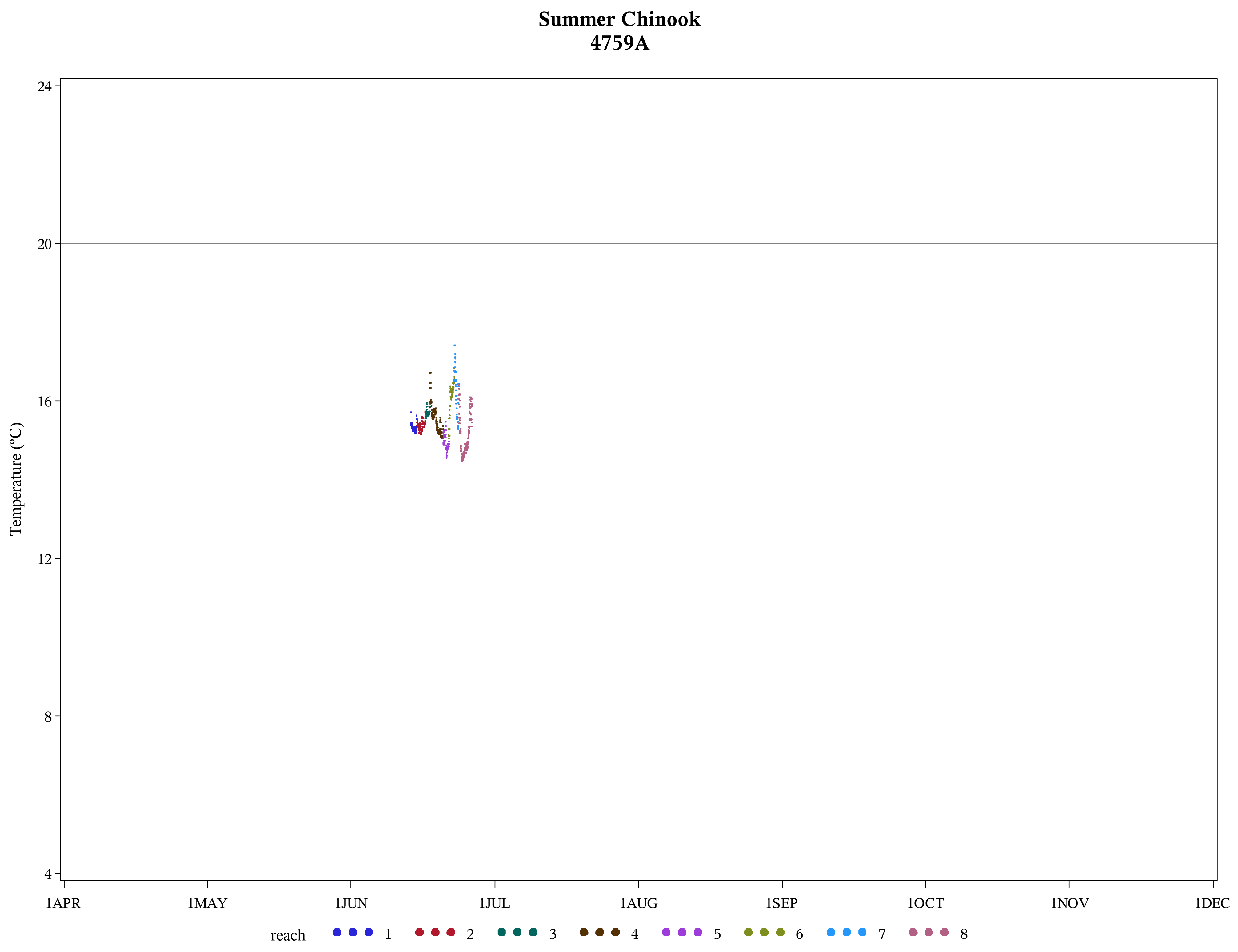
Summer Chinook
4759A
Temperature (°C)
24
20
16
12
8
4
1APR
1MAY
1JUN
1JUL
1AUG
1SEP
1OCT
1NOV
1DEC
reach
1
2
3
4
5
6
7
8

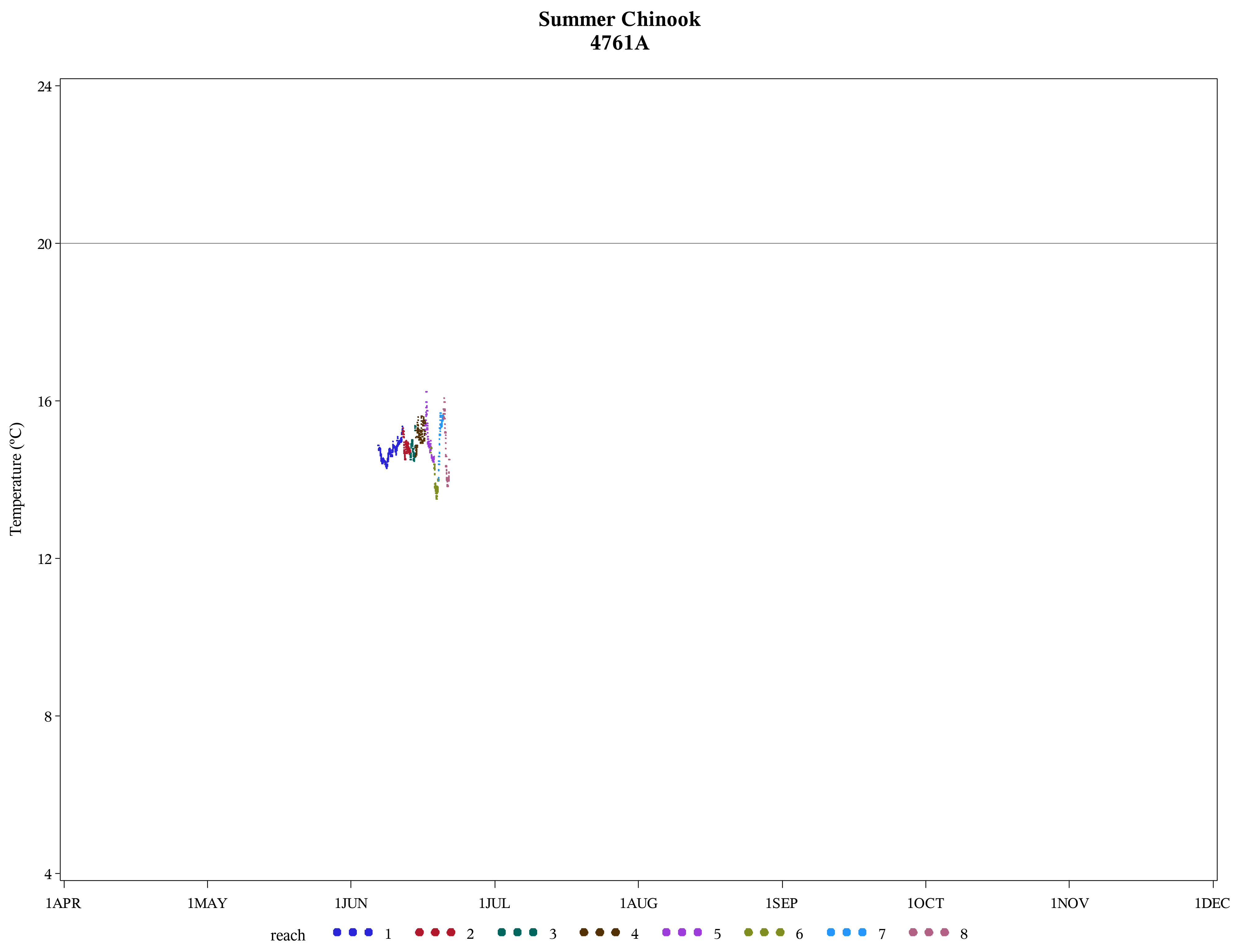
Summer Chinook
4761A
Temperature (°C)
24
20
16
12
8
4
1APR
1MAY
1JUN
1JUL
1AUG
1SEP
1OCT
1NOV
1DEC
reach
1
2
3
4
5
6
7
8

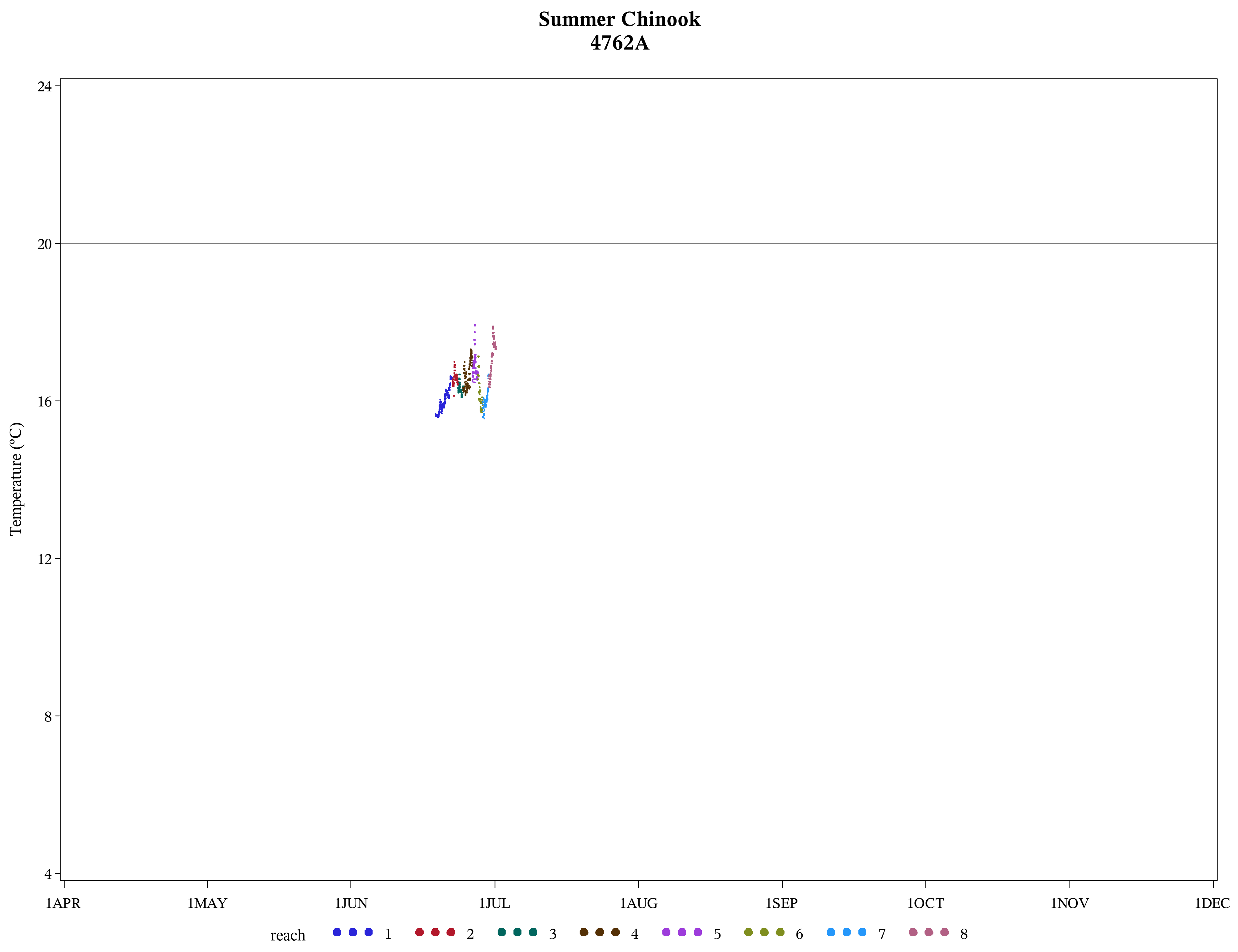

Summary Chinook
4762A
Temperature (°C)
24
20
16
12
8
4
1APR
1MAY
1JUN
1JUL
1AUG
1SEP
1OCT
1NOV
1DEC
reach
1
2
3
4
5
6
7
8

Summer Chinook
4775A

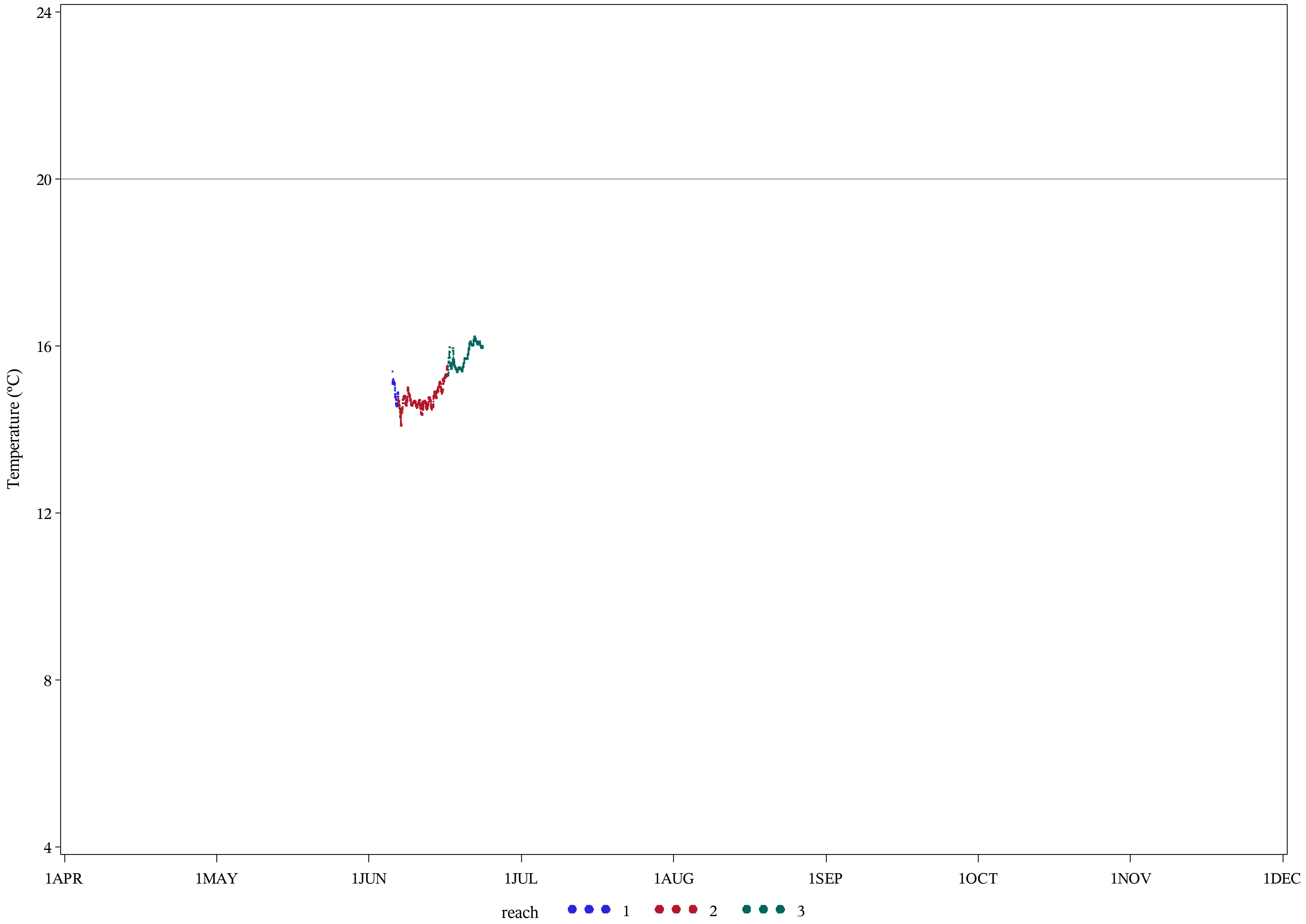

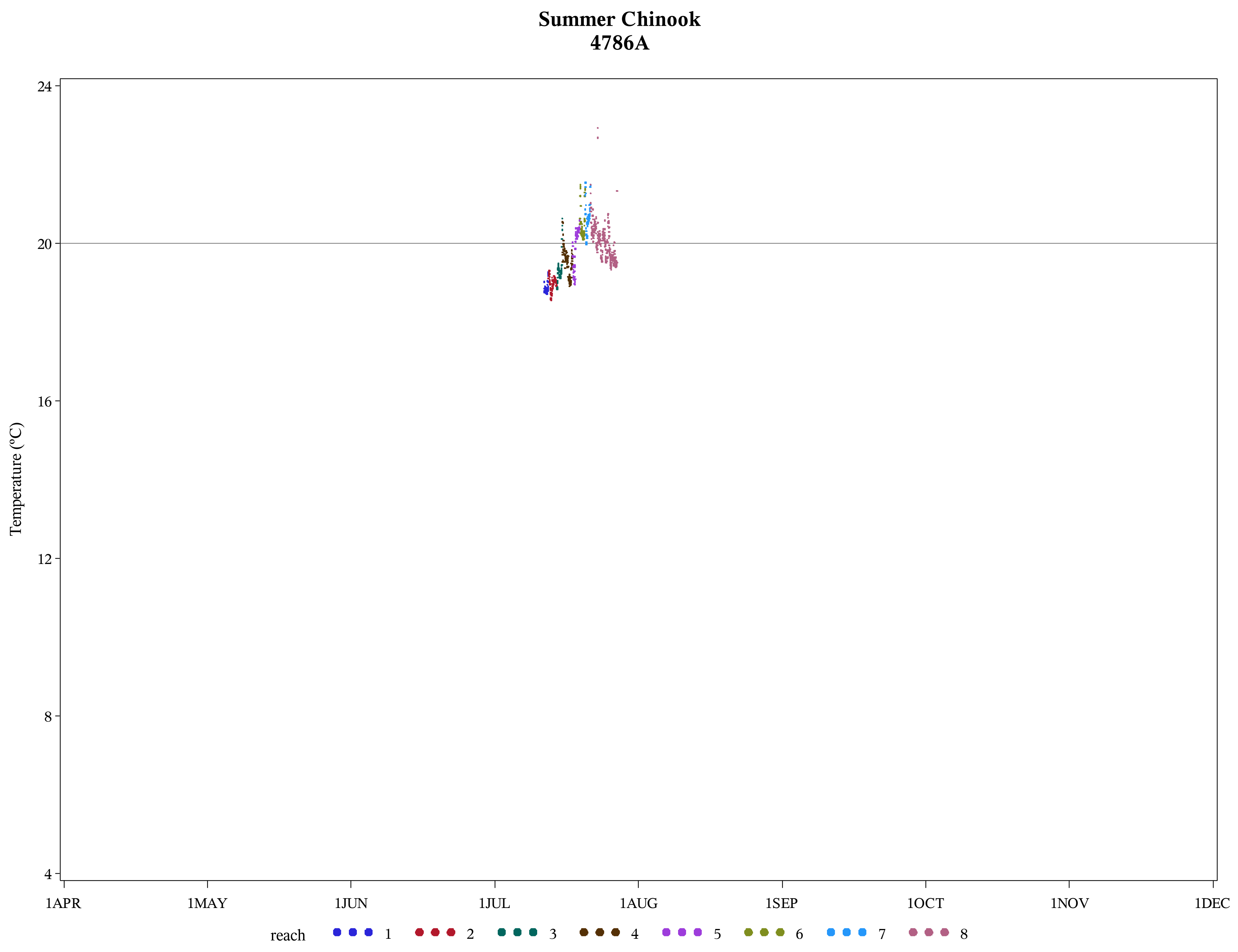
Summer Chinook
4786A
Temperature (°C)
24
20
16
12
8
4
1APR
1MAY
1JUN
1JUL
1AUG
1SEP
1OCT
1NOV
1DEC
reach
1
2
3
4
5
6
7
8

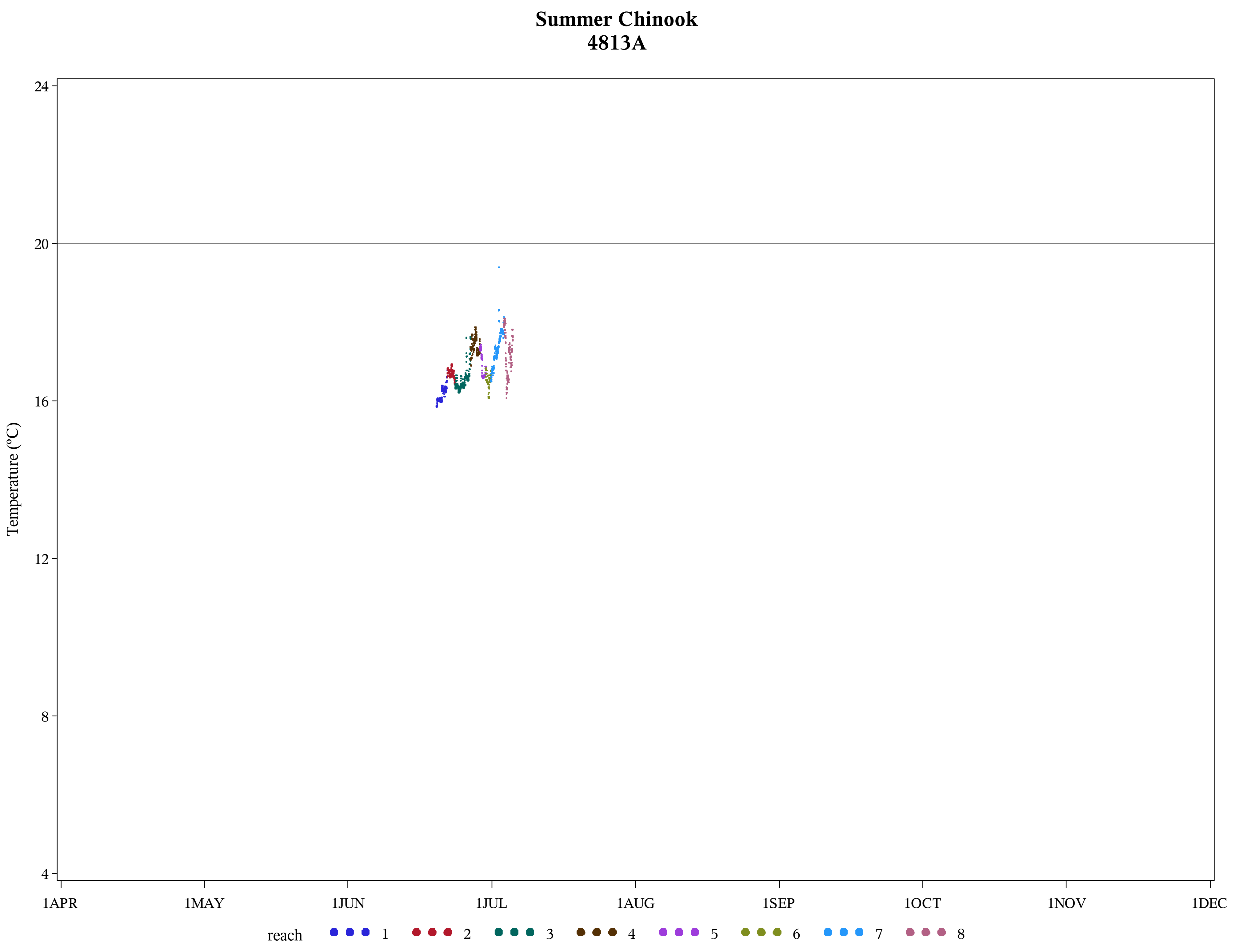
Summer Chinook
4813A
Temperature (°C)
24
20
16
12
8
4
1APR
1MAY
1JUN
1JUL
1AUG
1SEP
1OCT
1NOV
1DEC
reach
1
2
3
4
5
6
7
8

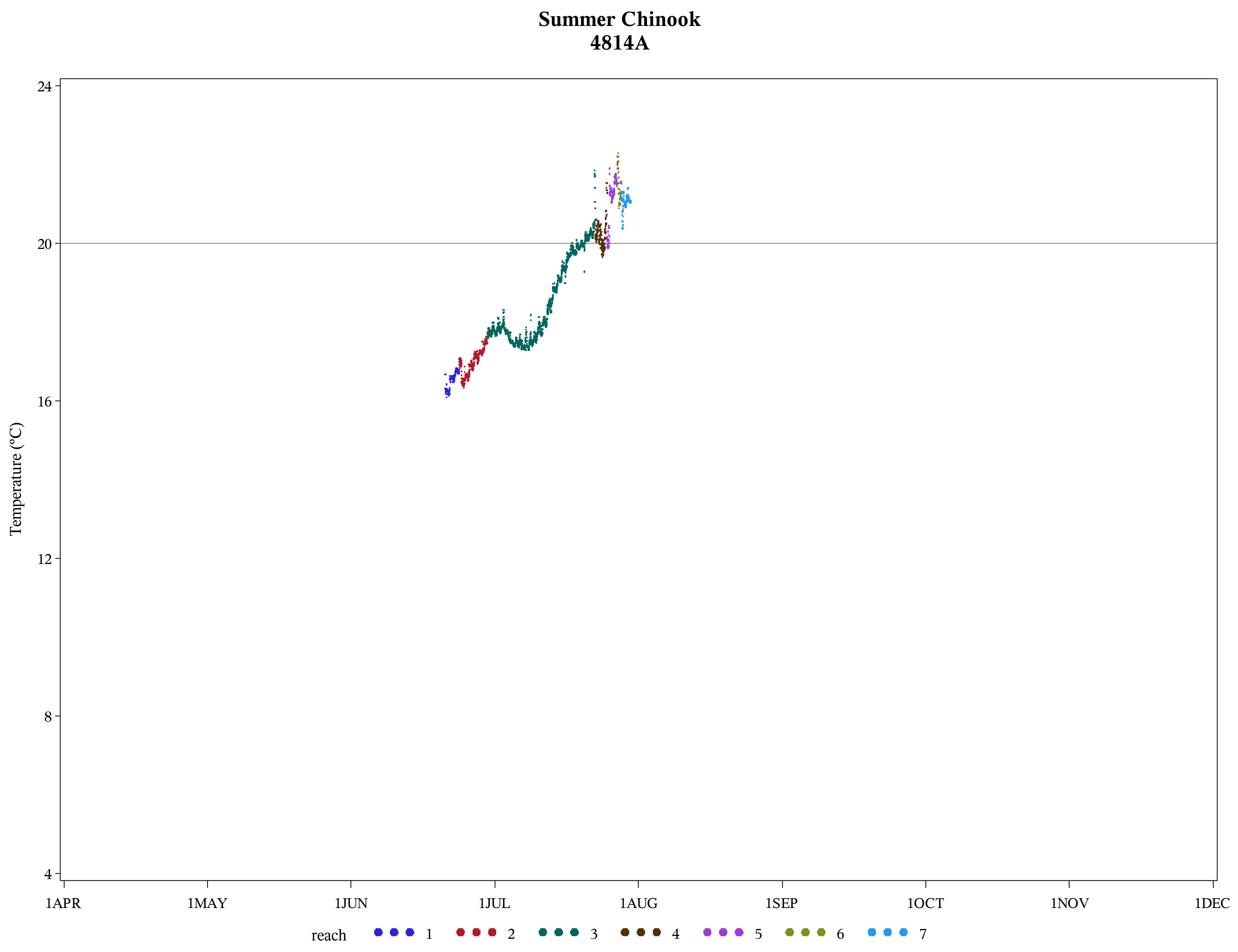
Summary Chinook
4814A
Temperature (°C)
24
20
16
12
8
4
1APR
1MAY
1JUN
1JUL
1AUG
1SEP
1OCT
1NOV
1DEC
reach
1
2
3
4
5
6
7

Summer Chinook
4817A

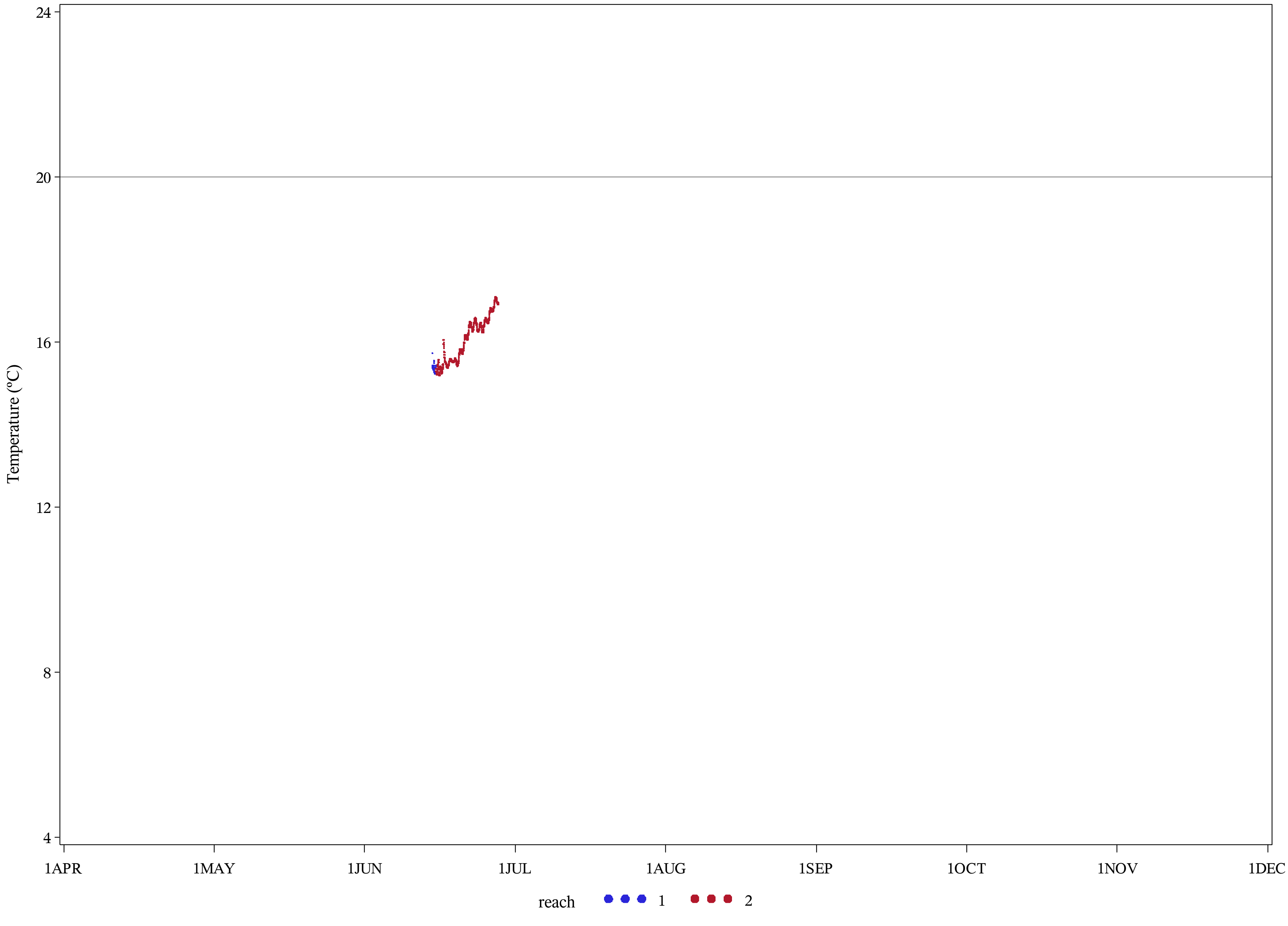

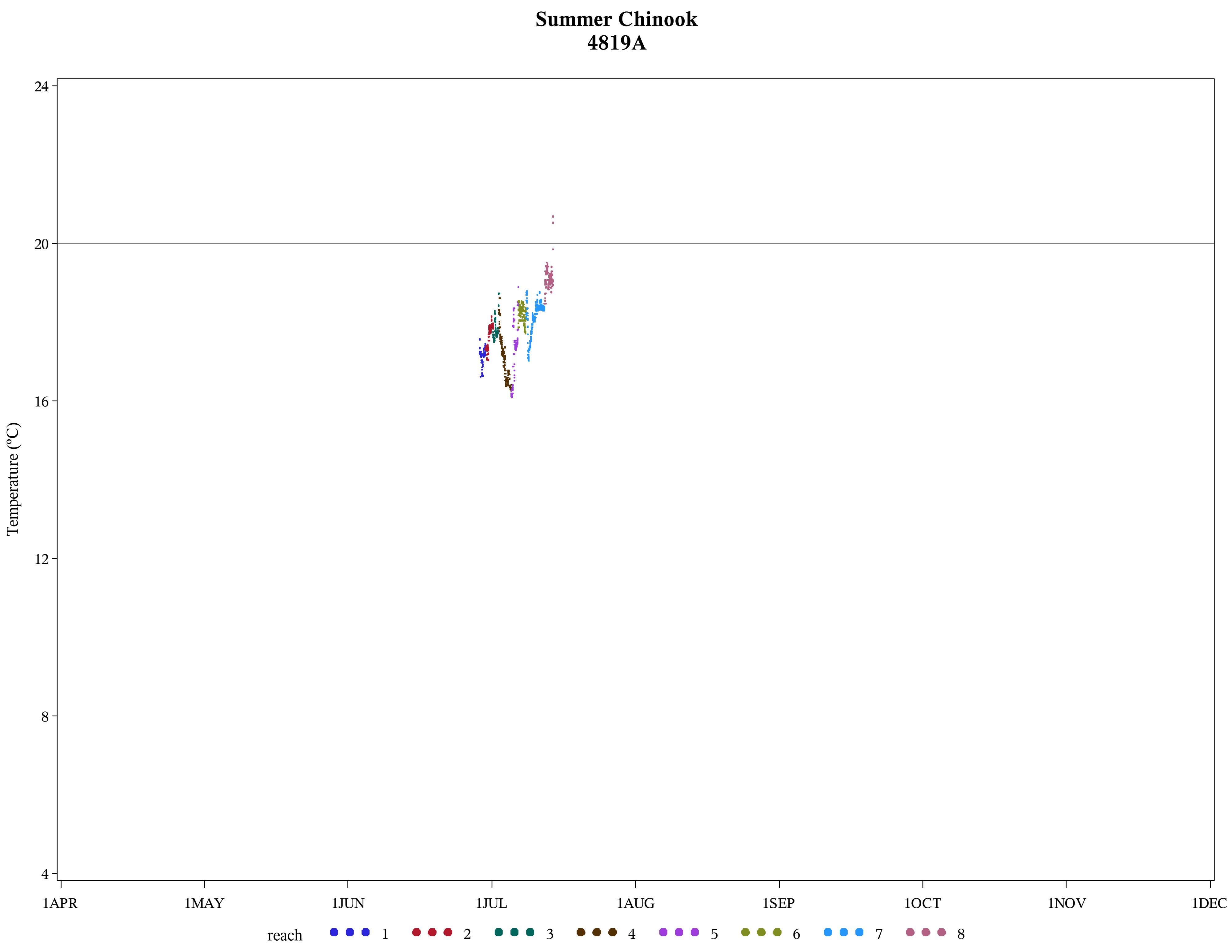
Summer Chinook
4819A
24
20
16
12
8
4
Temperature (°C)
1APR
1MAY
1JUN
1JUL
1AUG
1SEP
1OCT
1NOV
1DEC
reach
1
2
3
4
5
6
7
8

Summer Chinook
4829A

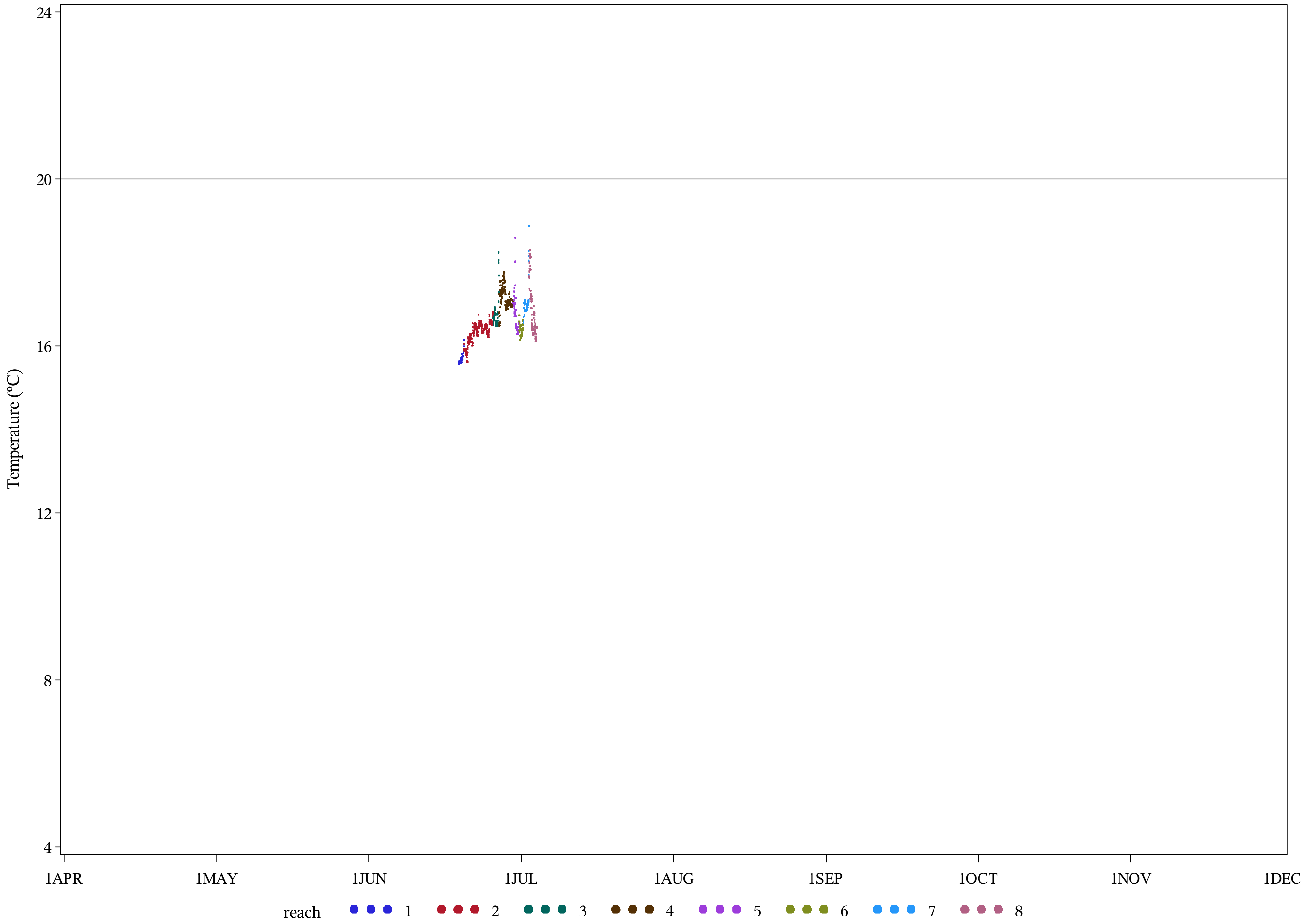

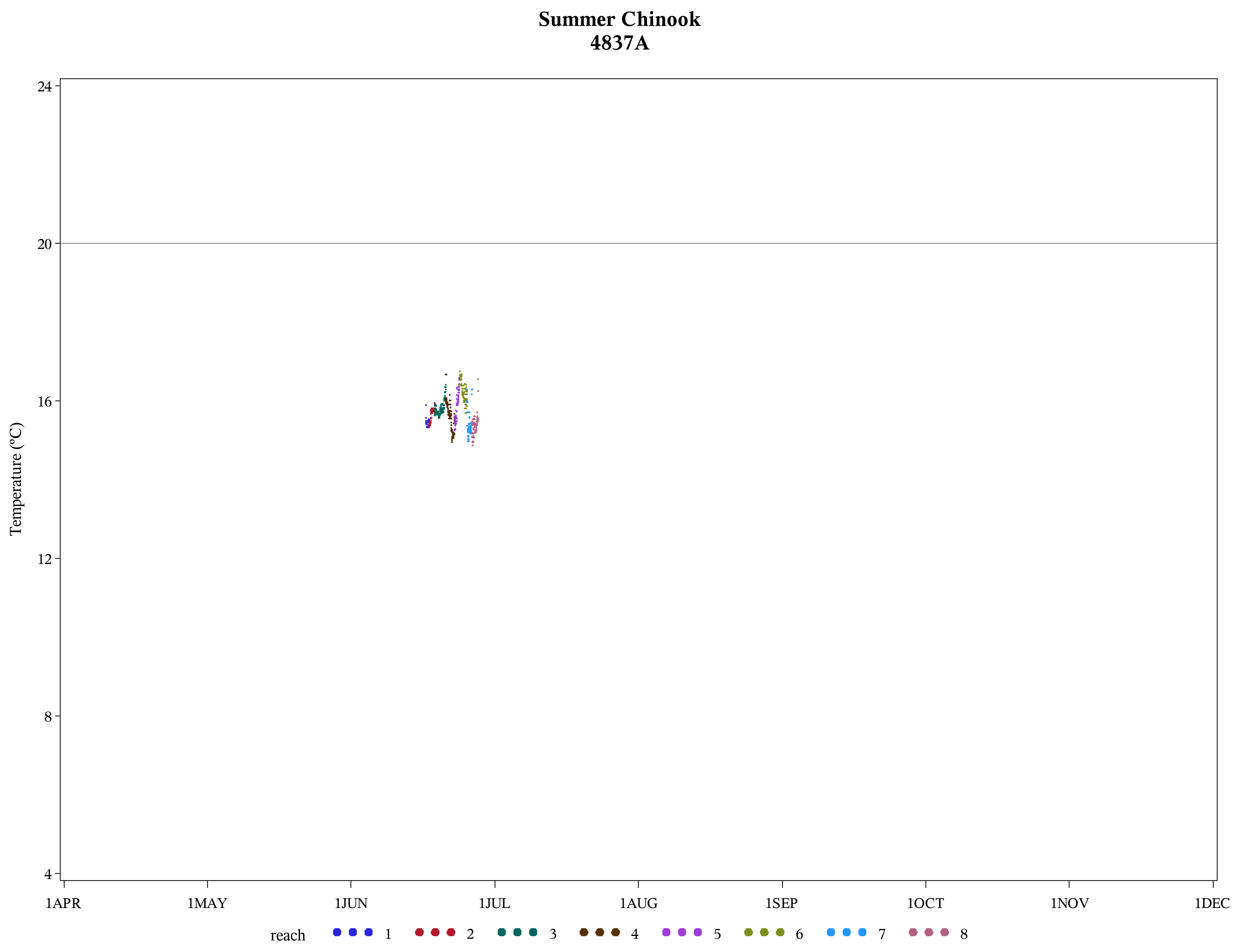

Summer Chinook
4837A
Temperature (°C)
24
20
16
12
8
4
1APR
1MAY
1JUN
1JUL
1AUG
1SEP
1OCT
1NOV
1DEC
reach
1
2
3
4
5
6
7
8

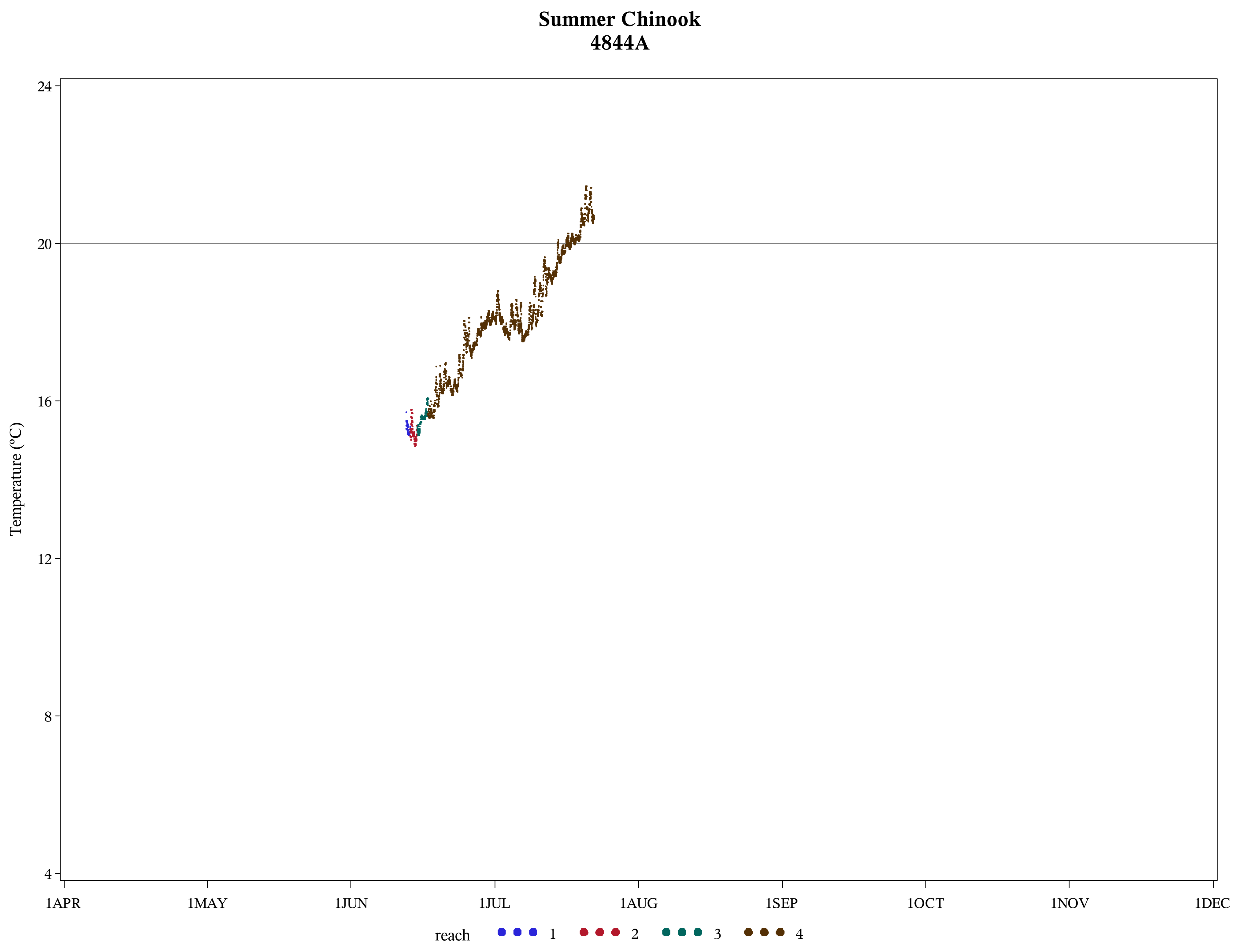

Summer Chinook
4844A
Temperature (°C)
24
20
16
12
8
4
1APR
1MAY
1JUN
1JUL
1AUG
1SEP
1OCT
1NOV
1DEC
reach
1
2
3
4

Summer Chinook
4869A

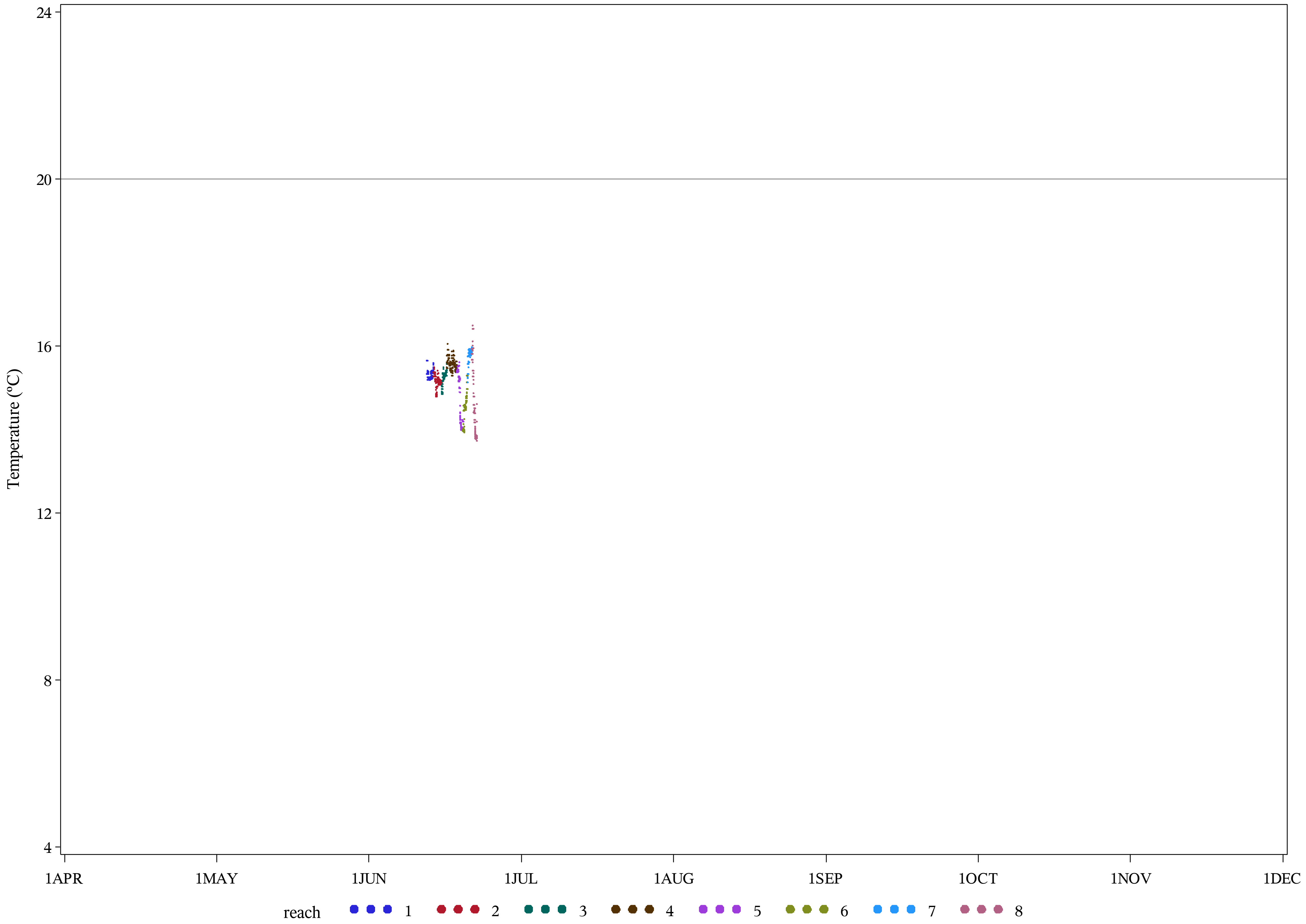

Summer Chinook
4878A

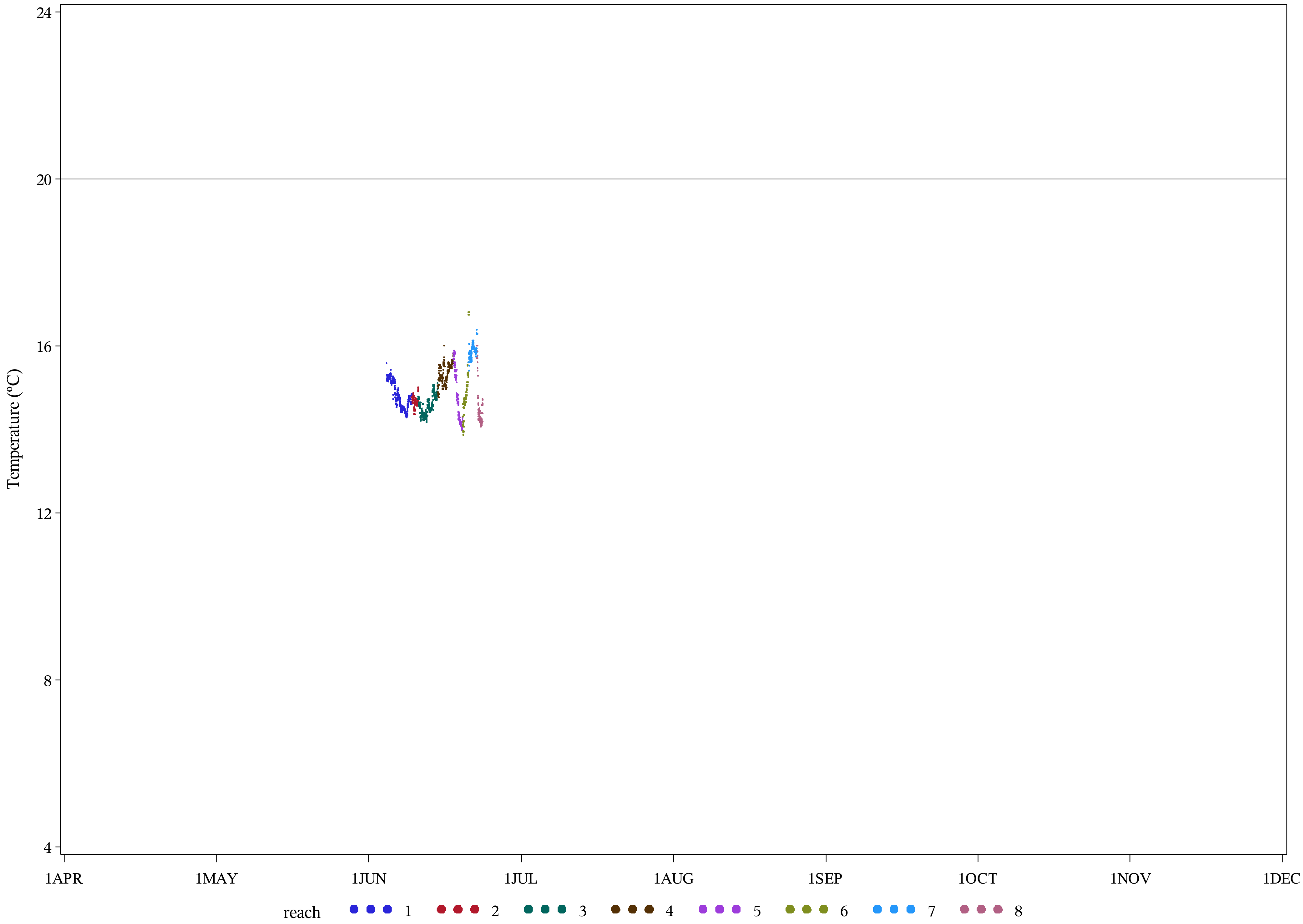

Summer Chinook
4882A

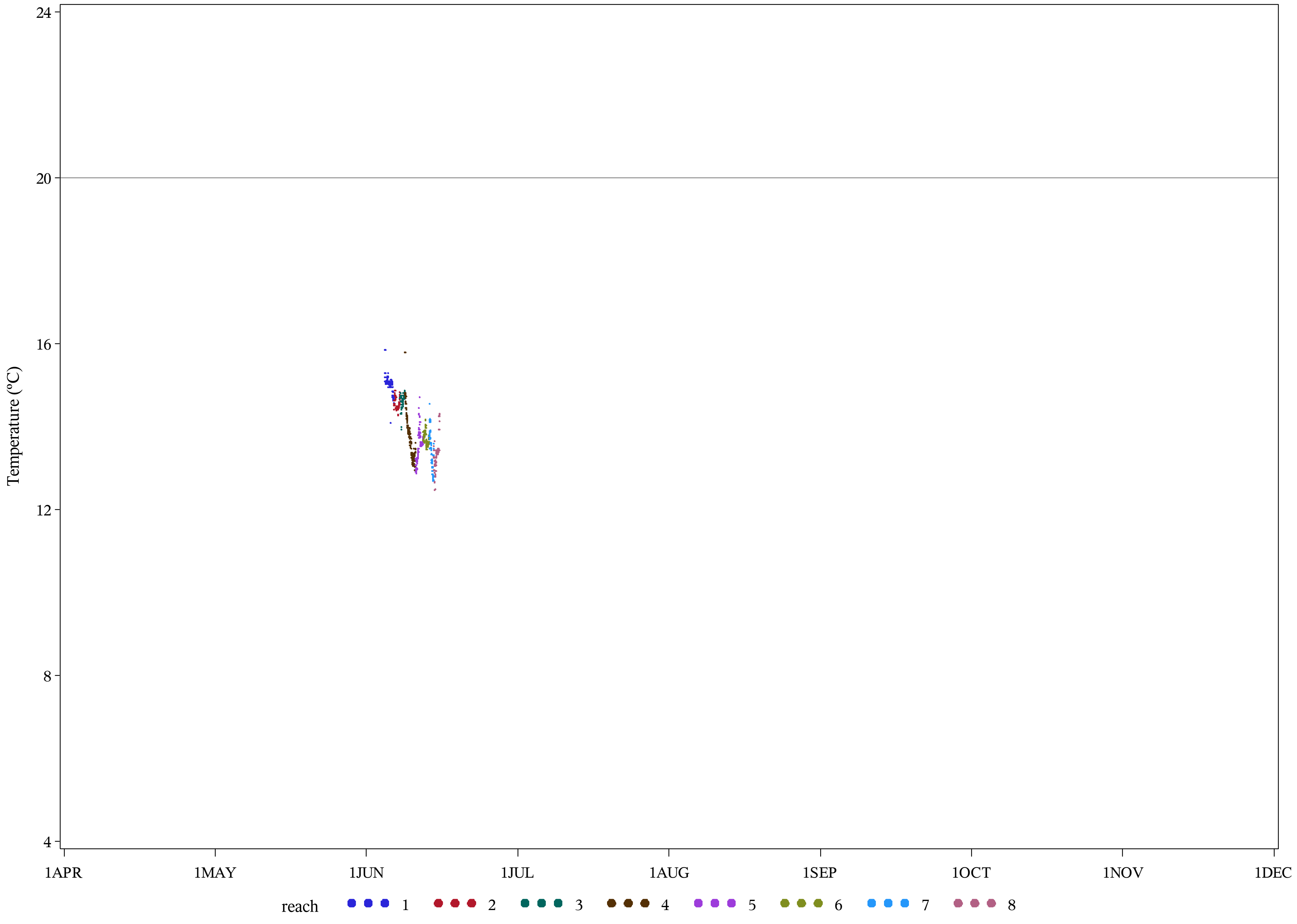

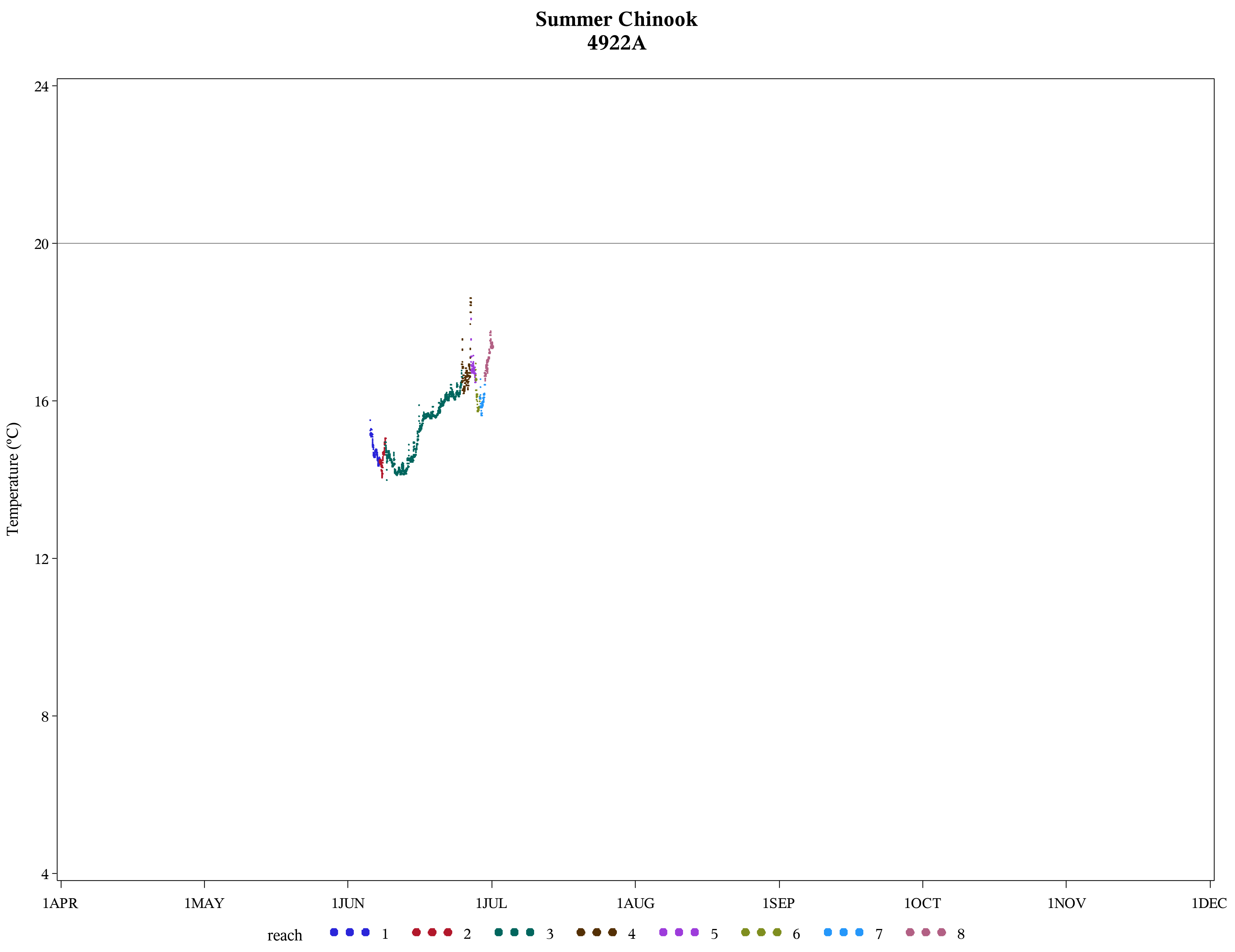
Summer Chinook
4922A
Temperature (°C)
24
20
16
12
8
4
1APR
1MAY
1JUN
1JUL
1AUG
1SEP
1OCT
1NOV
1DEC
reach
1
2
3
4
5
6
7
8

Summer Chinook
4934A

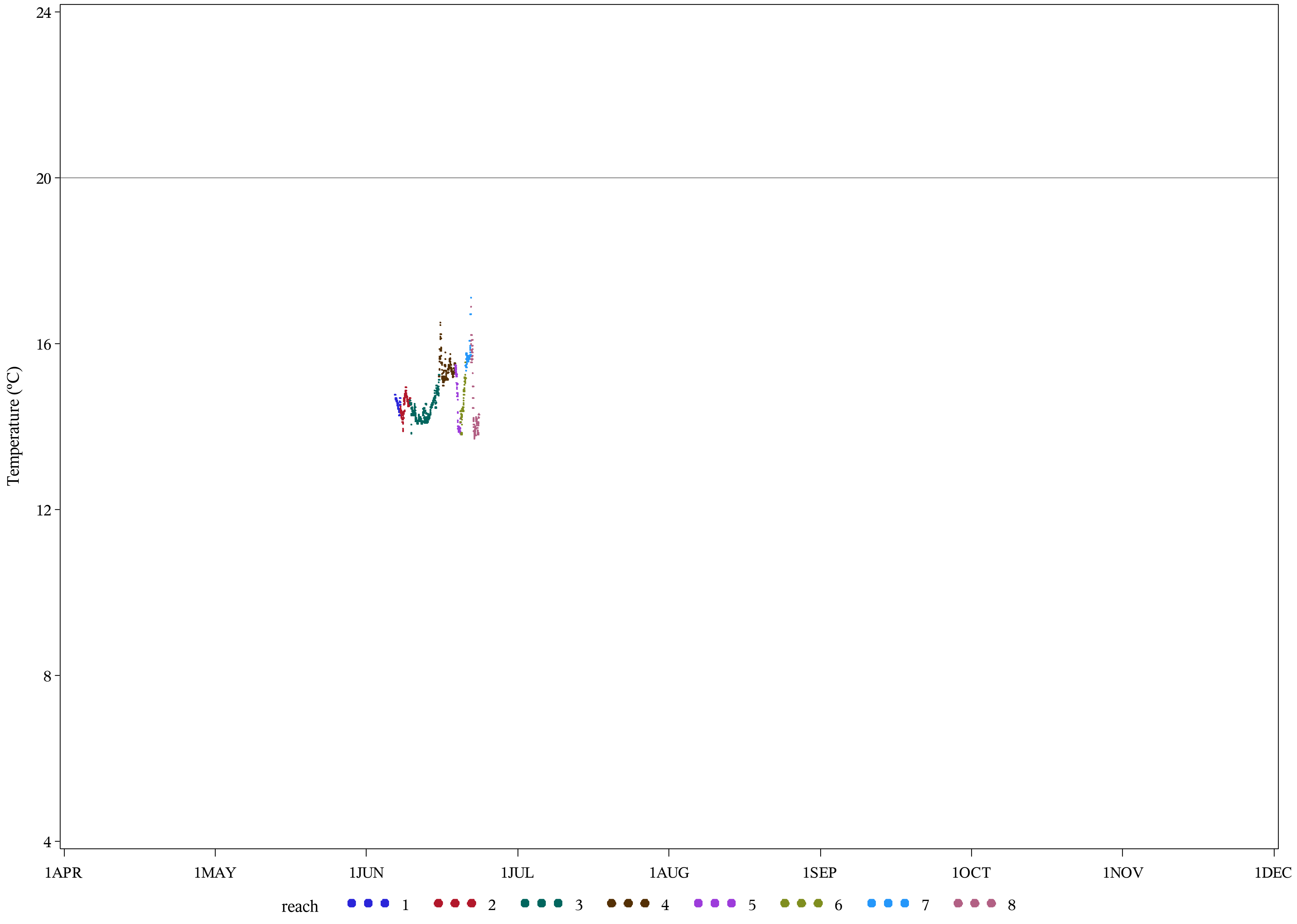

Summer Chinook
4958A

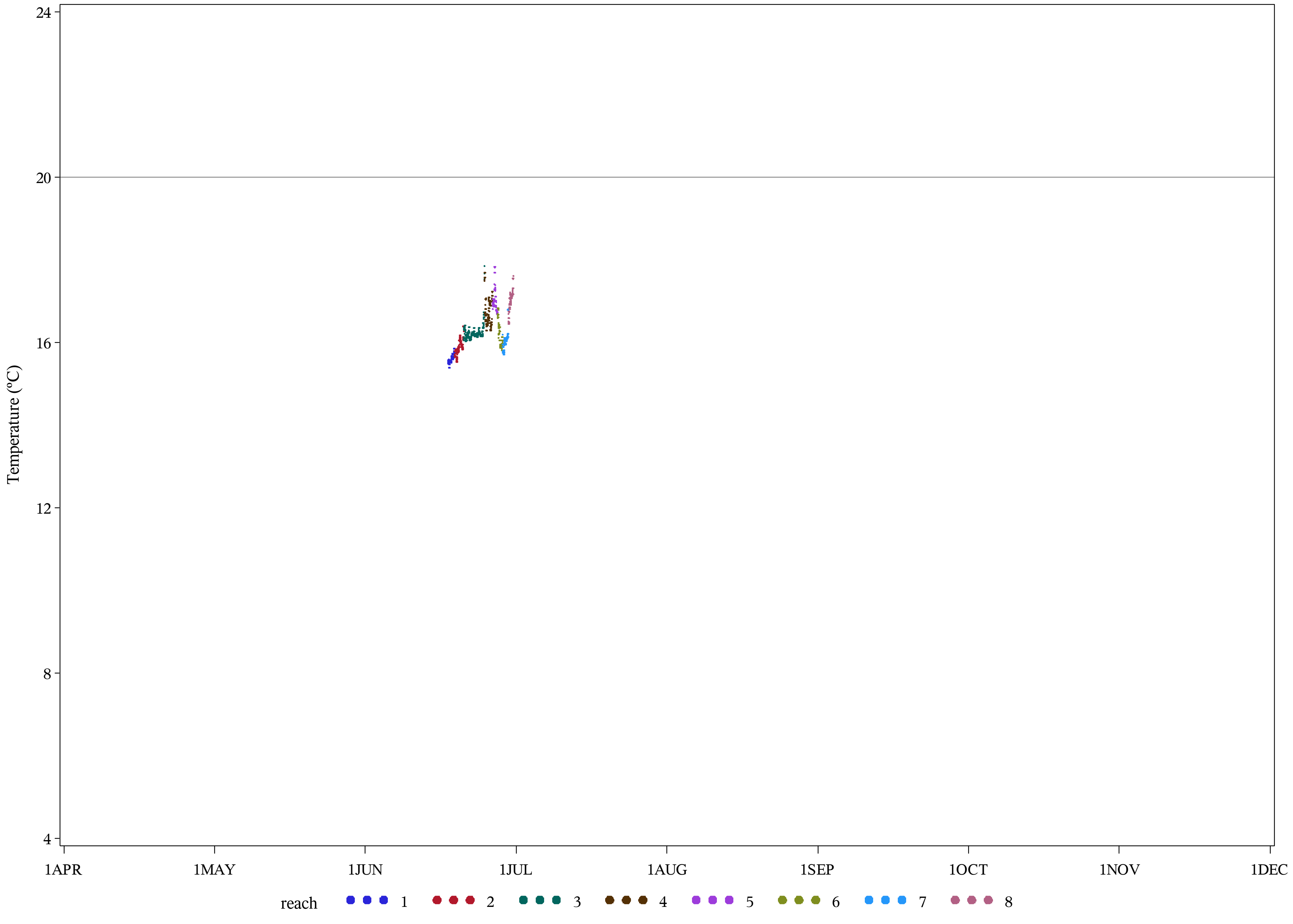

Supplement: S2 Appendix — Graphs show individual body-temperature histories, color coded by river reach. The run (spring Chinook salmon, summer Chinook salmon, fall Chinook salmon, steelhead) and RDST number are shown for each fish. (PDF) [file pone.0204274.s002.pdf]
