# Supplementary material for: Thermal exposure of adult Chinook salmon and steelhead: Diverse behavioral strategies in a large and warming river system
Source: PLoS One. 2018 Sep 21;13(9):e0204274. doi: 10.1371/journal.pone.0204274 (PMC6150539; doi:10.1371/journal.pone.0204274)
Supplement: S3 Appendix — Five tables summarizing the final adult Chinook salmon and steelhead detection locations (Tables S1-S4) and juvenile PIT-tag locations (Table S5) of all RDST-tagged Snake River study fish. (PDF) [file pone.0204274.s003.pdf]

S3 Appendix

Tables summarizing the final detection locations and juvenile PIT-tag locations of all Snake River study fish.

Table S1. Final detection sites for RDST-tagged spring Chinook salmon, their fin clip status, and whether they were or were not of known origin based on juvenile PIT tags.

		Total	Fin-clipped		Known origin	
Year	Final detection site	*n*	Yes	No	Yes	No
2000	Clearwater River	5	5	-	2	3
	North Fork Clearwater River	4	4	-	-	4
	Dworshak NFH	4	4	-	1	3
	Kooskia NFH	1	1	-	-	1
	South Fork Clearwater River	3	2	1	2	1
	Lochsa River	7	7	-	1	6
	Grande Ronde River	1	-	1	1	-
	Salmon River					
	Rapid River	8	8	-	1	7
	South Fork Salmon River	18	17	1	1	17
	Middle Fork Salmon River	2	-	2	2	-
	Pahsimeroi FH	1	1	-	-	1
	Sawtooth FH	1	-	1	1	-
	Imnaha River	4	2	2	2	2
	Hells Canyon Dam	4	4	-	3	1
2002	Clearwater River	4	3	1	3	1
	North Fork Clearwater River	2	2	-	-	2
	Dworshak NFH					
	Clear Creek	1	1	-	-	1
	Kooskia NFH	3	3	-	2	1
	South Fork Clearwater River	1	-	1	1	-
	Lolo Creek	2	-	2	2	1
	Lochsa River	3	-	3	3	-
	Grande Ronde River	6	1	5	5	1
	Lookingglass H	3	2	1	3	1
	Lostine River	1	1	-	1	1
	Wallowa River	1	-	1	1	-
	Salmon River	5	2	3	4	1
	Rapid River	6	6	-	2	4
	South Fork Salmon River	14	8	6	6	8
	Middle Fork Salmon River	8	-	8	8	1
	Lemhi River	1	-	1	1	-
	Pahsimeroi FH					
	Sawtooth FH	2	-	2	2	1
	Imnaha River	1	-	1	1	-
	Hells Canyon Dam					
	Lower Granite Dam	6	2	4	4	2
	Hydrosystem	11	2	9	11	-
**Total**	**All sites**	**144**	**88**	**56**	**77**	**67**

Table S2. Final detection sites for RDST-tagged summer Chinook salmon, their fin clip status, and whether they were or were not of known origin based on juvenile PIT tags.

		Total	Fin-clipped		Known origin	
Year	Final detection site	*n*	Yes	No	Yes	No
2000	Clearwater River	1	1	-	-	1
	Grande Ronde River	1	1	-	-	1
	Salmon River					
	South Fork Salmon River	6	6	-	-	6
	Pahsimeroi FH	2	2	-	-	2
	Imnaha River	2	2	-	-	2
	Lower Granite Dam	1	1	-	1	-
2002	Grande Ronde River	2	1	1	2	-
	Lostine River	4	3	1	4	-
	Imnaha River	1	-	1	1	-
	Salmon River					
	South Fork Salmon River	7	4	3	5	2
	Pahsimeroi FH	1	-	1	1	-
	Lower Granite Dam	9	3	6	7	2
	Hydrosystem	3	1	2	3	-
**Total**	**All sites**	**40**	**25**	**15**	**24**	**16**

Table S3. Final detection sites for RDST-tagged fall Chinook salmon, their fin clip status, and whether they were or were not of known origin based on juvenile PIT tags.

		Total	Fin-clipped		Known origin	
Year	Final detection site	*n*	Yes	No	Yes	No
2000	Clearwater River	3	3	-	1	2
	Snake River above L. Granite Dam	3	3	-	-	3
	Lower Granite Dam	2	2	-	-	2
2002	Clearwater River	9	-	9	8	1
	Snake River above L. Granite Dam	3	-	3	2	1
	Lower Granite Dam	1	1	-	-	1
	Hydrosystem	7	-	7	7	-
**Total**	**All sites**	**28**	**9**	**19**	**18**	**10**

Table S4. Final detection sites for RDST-tagged steelhead, their fin clip status, and whether they were or were not of known origin based on juvenile PIT tags.

		Total	Fin-clipped		Known origin	
Year	Final detection site	*n*	Yes	No	Yes	No
2000	Clearwater River	10	9	1	1	9
	North Fork Clearwater River	1	1	-	-	1
	Dworshak NFH	5	5	-	-	5
	Kooskia NFH	1	1	-	-	1
	South Fork Clearwater River	2	2	-	-	2
	Grande Ronde River	1	1	-	-	1
	Salmon River	11	11	-	1	10
	Little Salmon River	2	2	-	-	2
	Pahsimeroi FH	2	2	-	-	2
	Snake River above L. Granite Dam	3	3	-	-	3
	Lower Granite reservoir	2	2	-	-	2
	Hells Canyon Dam	1	1	-	-	1
	Oxbow FH	1	1	-	-	1
	Lower Granite Dam	4	4	-	-	4
2002	Clearwater River	5	-	5	5	-
	South Fork Clearwater River	4	-	4	4	-
	Lochsa River	9	-	9	9	-
	Grande Ronde River	4	2	2	4	-
	Catherine Creek	1	-	1	1	-
	Imnaha River	2	-	2	2	-
	Salmon River	25	2	23	25	-
	Little Salmon River	1	1	-	-	1
	South Fork Salmon River	3	-	3	3	-
	Snake River above L. Granite Dam	13	1	12	13	-
	Lower Granite reservoir	1	-	1	1	-
	Lower Granite Dam	61	9	52	59	2
	Hydrosystem	25	6	19	25	-
**Total**	**All sites**	**200**	**66**	**134**	**153**	**47**

Table S5. Locations where RDST-tagged Chinook salmon and steelhead were PIT-tagged as juveniles, including tagging codes and site descriptions as archived in PTAGIS.

Juvenile PIT tag site	Spring Chinook	Summer Chinook	Fall Chinook	Steelhead
**No juvenile PIT tag**	67	18	10	47
**Lower Granite Dam**				
LGRRBR: Barge transportation	12	1		5
LGRRRR: Return to river	29	9	1	109
**Snake River**				
SNAKER: Snake River			6	
SNKTRP: Snake Trap	2	1		2
**Clearwater River**				
BARGAC: Bargamin Creek				2
BCCAP: Big Canyon Creek Acclimation Facility			1	
CLEARC: Clear Creek	2			2
CLWR: Clearwater River			10	
CLWRSF: South Fork Clearwater River				1
DWORMS: Dworshak NFH				2
FISTRP: Fish Creek Trap				9
KOOS: Kooskia NFH	1			
LOLOC: Lolo Creek	1			
MEADOC: Meadow Creek	2			
NEWSOC: Newsome Creek				1
POWP: Powell Rearing Pond	1			
REDR: Red River				1
REDTRP: Red River Trap	2			1
**Grande Ronde River**				
CATHEC: Catherine Creek				1
COTP: Cottonwood Acclimation Pond				1
GRAND2: Grande Ronde River	1			
GRANDP: Grande Ronde River Pond	1			
GRANDR: Grande Ronde River	4			6
LOOKGC: Looking Glass Creek	2			
LOSTIP: Lostine River Pond	1	4		
LOSTIR: Lostine River		1		1
MINAMR: Minam River	1			
**Imnaha River**				
IMNAHW: Imnaha River Weir	1			
IMNTRP: Imnaha River Trap	2			2
LSHEEF: Little Sheep Facility				1
**Salmon River**				
CHAMBC: Chamberlain Creek				2
ELKC: Elk Creek		1		
HERDC: Herd Creek				1
HORSEC: Horse Creek				1
KNOXB: Knox Bridge, South Fork Salmon River		1		
LAKEC: Lake Creek				1
LEMHIW: Lemhi River Weir	1			
MARTRP: March Creek Trap	4			
PAHTRP: Pahsimeroi Trap		2		
SALTRP: Salmon River Trap	4	2		
SAWT: Sawtooth Hatchery				1
SAWTRP: Sawtooth Trap	2			
YANKWF: West Fork Yankee Fork Salmon River	1			
